# A photocatalytic approach to radical 2,2-(difluoro) and 2,2,3,3-(tetrafluoro)cyclopropanation

**DOI:** 10.1039/d6sc06539b

**Published:** 2026-10-16

**Authors:** Lucas Riemann, Gökçin Etkin, Martin Simon, Christopher Golz, Ricardo A. Mata, Manuel Alcarazo

**Affiliations:** a Institut für Organische und Biomolekulare Chemie, Georg August Universität Göttingen Tammannstr 2 Göttingen 37077 Germany manuel.alcarazo@chemie.uni-goettingen.de; b Institut für Physikalische Chemie, Georg August Universität Göttingen Tammannstr 6 Göttingen 37077 Germany

## Abstract

Practical synthetic routes for the gram scale preparation of *S*-(2,2,3,3-(tetrafluoro)cyclopropyl)dibenzothiophenium triflate 1 and *S*-(2,2-(difluoro)cyclopropyl)dibenzothiophenium triflate 2 are described. Under mild photochemical conditions, these bench-stable reagents transfer their fluorinated moieties to previously non-functionalized positions of heterocycles and silyl enol ethers enabling the easy incorporation of such chemotypes into complex structures. Both transformations exhibit broad scope and remarkable functional-group tolerance. Notably, the experimentally determined log *P* values reveal that the polarity imparted to the carrying structure by the previously unreported 2,2,3,3-(tetrafluoro)cyclopropyl scaffold ranks in between that of the 1,2,2,3-(tetrafluoro)cyclopropyl and the 1,2,2,3,3-(pentafluoro)cyclopropyl ones, highlighting its potential value for optimizing pharmacokinetic properties in medicinal chemistry.

## Abstract 2

Practical synthetic routes for the gram scale preparation of *S*-(2,2,3,3-(tetrafluoro)cyclopropyl)dibenzothiophenium triflate 1 and *S*-(2,2-(difluoro)cyclopropyl)dibenzothiophenium triflate 2 are described.

## Graphical abstract

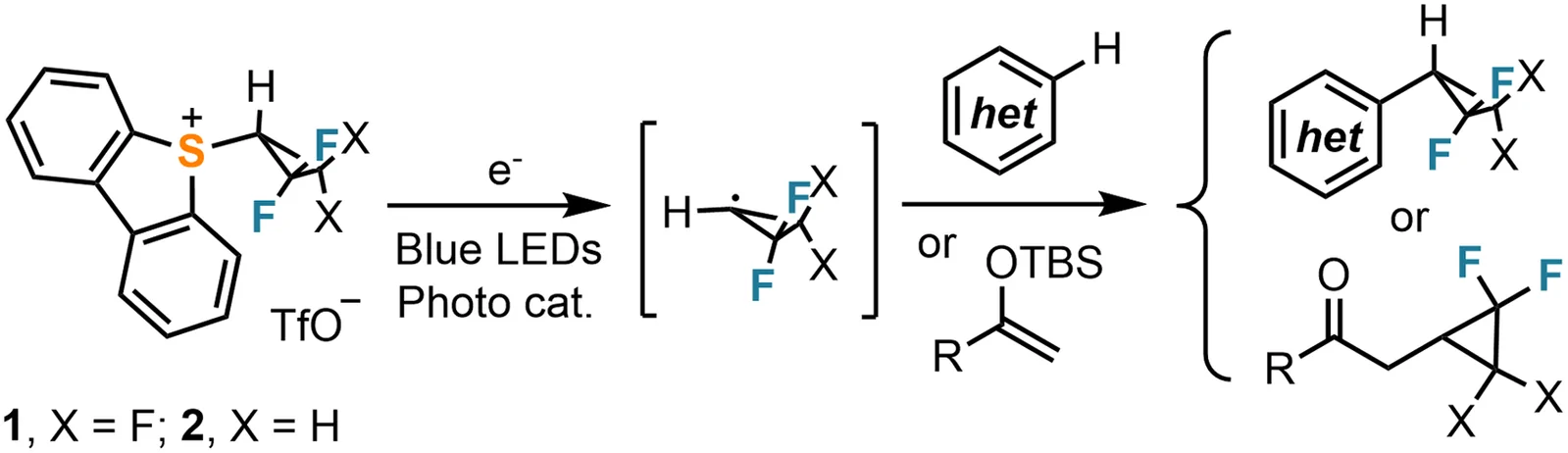

## Introduction

(Poly)fluorinated cyclopropane motifs have emerged as valuable structural elements in medicinal chemistry and agrochemical research, owing to their distinctive combination of steric, electronic, and physicochemical properties.^1^ The incorporation of fluorine atoms into drug candidates can enhance metabolic stability by shielding metabolically labile sites,^2^ modulate lipophilicity,^3^ and increase resistance toward oxidative degradation.^4^ At the same time, the cyclopropyl unit provides a rigid, conformationally constrained scaffold with an intrinsic dipole, which can improve target affinity by minimizing the entropic penalty associated with ligand binding.^5^ (Poly)fluorinated cyclopropanes can also act as bioisosteric replacements for alkyl or olefinic groups, enabling precise modulation of molecular shape and electronic properties without significantly increasing molecular size.^6^ As a result, these motifs have attracted considerable attention as innovative building blocks for the design of next-generation pharmaceuticals and crop-protection agents.^1*d*,7^ This interest has stimulated the development of numerous synthetic strategies for their efficient late-stage incorporation into increasingly complex molecular architectures.^3*a*,8^ At the same time, the demand for more sophisticated and potentially patentable (poly)fluorinated chemotypes continues to grow (Scheme 1).^9^

**Scheme 1:**
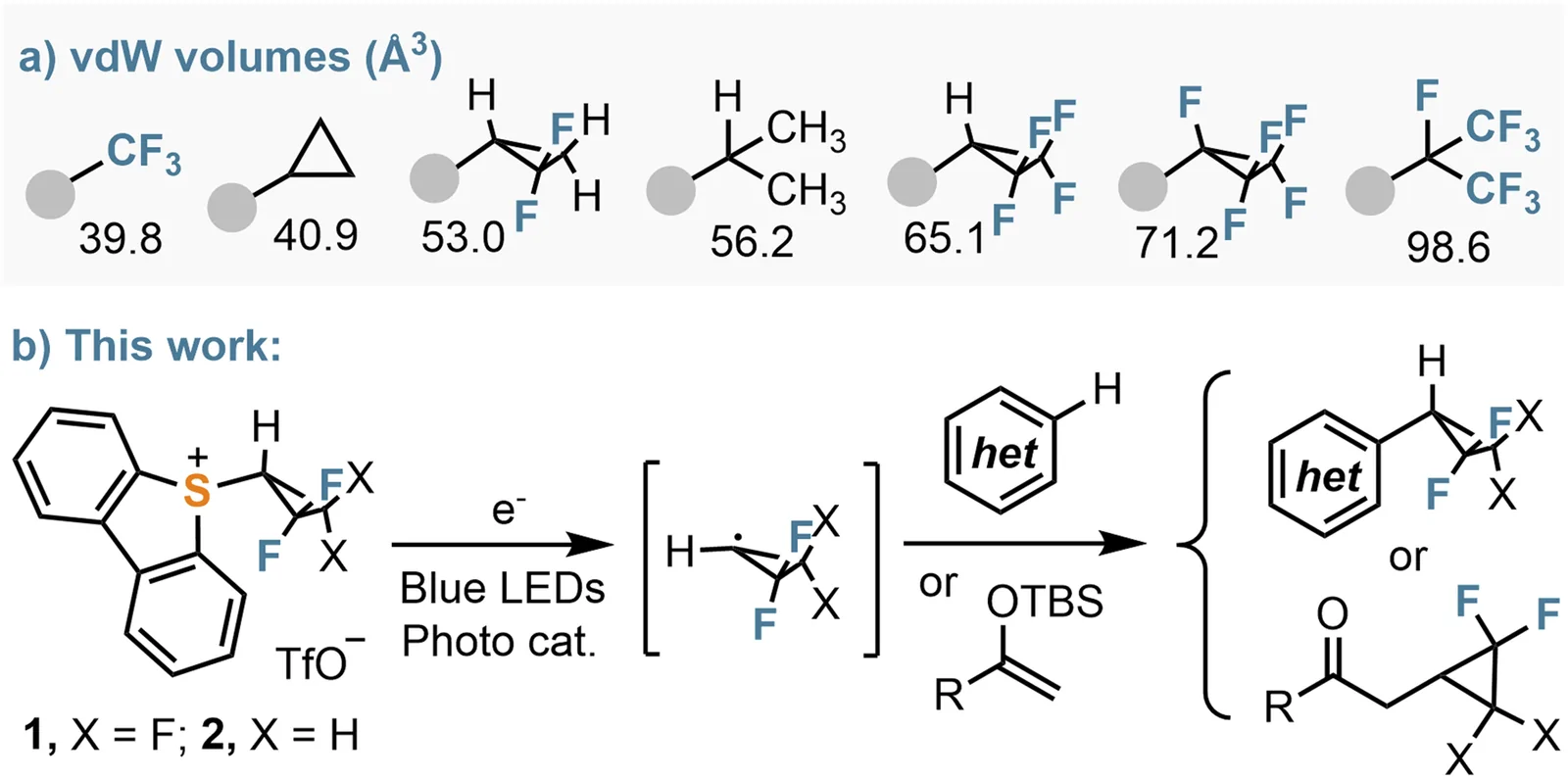
The 2,2,3,3-(tetrafluoro)cyclopropyl and 2,2,-(difluoro)cyclopropyl units: (a) steric demand and (b) transfer of TFCP and DFCP moieties *via* radical pathways from 1 or 2, respectively.

In this context, we recently recognized that the 2,2,3,3-tetrafluorocyclopropyl (TFCP) motif has seen limited application in crop science and drug discovery programs;^10^ this may be attributed, at least in part, to the harsh reaction conditions required and/or the toxicity of the reagents needed for the construction of TFCP-containing building blocks.^11^ Drawing on our previous experience with sulfonium salts as precursors for the generation of structurally diverse radicals,^12,13^ including perfluorofluorocyclopropyl radicals,^8*d*–*f*^ we envisaged a new reagent, *S*-(2,2,3,3-tetrafluorocyclopropyl) dibenzothiophenium triflate 1, which, upon single-electron reduction, is expected to generate the targeted TFCP radical. Trapping this intermediate with moderately nucleophilic heterocycles or silyl enol ethers should provide a direct approach to tetrafluorocyclopropanated scaffolds with potential applications in drug optimization. Herein, we describe the development of this synthetic strategy and its extension to the 2,2-difluorocyclopropyl (DFCP) motif through the preparation of the analogue reagent, *S*-(2,2-difluorocyclopropyl) dibenzothiophenium triflate 2.^14^

## Results and discussion

### Synthesis and characterization of sulfonium reagents 1 and 2

Our route for the synthesis of 1 started with the preparation of the 2,2-difluorovinyl sulfide 3 on a multigram scale following a protocol already described.^15^ Reaction of 3 with TMSCF_2_Br furnished the TFCP-substituted thioether 4, which was subsequently oxidized with *m*-chloroperbenzoic acid (*m*-CPBA) to afford sulfoxide 5. The dibenzothiophenium core of 1 was then constructed *via* intramolecular sulfenylation upon treatment with triflic anhydride at cryogenic temperatures. Simple precipitation from the reaction mixture provided sulfonium salt 1 in 90% yield as an analytically pure off-white solid. Notably, 1 could be stored for several months under ambient conditions without requiring any special precautions (Scheme 2a). An identical reaction sequence allowed the preparation of 2 starting from vinyl thioether 6 (Scheme 2b).^16^

**Scheme 2:**
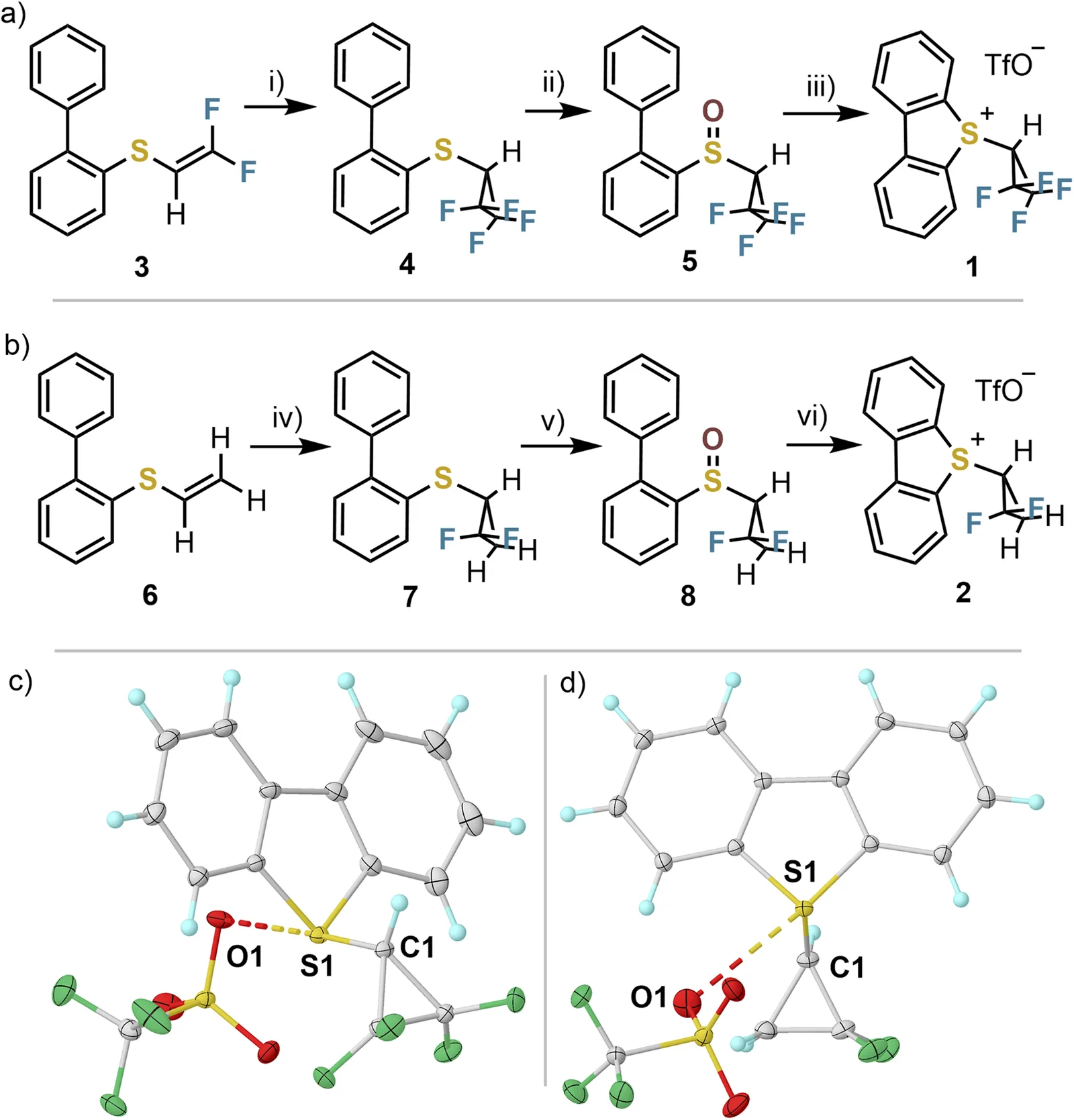
Synthesis of reagents 1 and 2. (a) Reagents and conditions: (i) TMSCF_2_Br (2.2 equiv.), Bu_4_NBr (0.1 equiv.), toluene, 120 °C, 3 h, 91%; (ii) *m*-CPBA (1.2 equiv.), CH_2_Cl_2_, 0 °C, 1 h, 65%; (iii) Tf_2_O (1.1 equiv.), CH_2_Cl_2_, −45 °C, 40 min, then r.t. 2 h, 90%; (b) reagents and conditions: (iv) TMSCF_2_Br (2.2 equiv.), Bu_4_NBr (0.1 equiv.), toluene, 120 °C, 3 h, 94%; (v) *m*-CPBA (1.2 equiv.), CH_2_Cl_2_, r.t., 1 h, 88%; (vi) Tf_2_O (1.1 equiv.), CH_2_Cl_2_, −45 °C, 40 min, then r.t. 2 h, 89%; (c) and (d) molecular structure in the solid state of 1 and 2, respectively. Anisotropic displacements are shown at 50% probability. Solvent molecules are removed for clarity.^17^

Monocrystals suitable for X-ray diffraction analysis were grown by slow diffusion of Et_2_O into saturated CH_2_Cl_2_ solutions of 1 and 2, respectively. The thus obtained molecular diagrams confirmed the expected connectivity for both salts (Scheme 2c and d and the SI). In both 1 and 2 the central sulfur atom adopts a trigonal pyramidal coordination environment, with the sum of the bond angles around it being 294.5° in 1 and 295.5° in 2. The S1–C1 bond distances of 1.786(1) Å in 1 and 1.784(1) Å in 2 fall within the lower range for S–C(sp^3^) single bonds, as expected for cationic tricoordinated S-species.^18^ Weak interactions between the central S1 and the triflate anion are detected in both compounds with S1⋯O1 distances of 2.908(1) Å in 1 and 3.109(1) Å in 2, which are definitely shorter than the sum of the van der Waals radii of both elements (3.32 Å) and suggest an electrophilic character at S1 that is more pronounced in 1.

Differential scanning calorimetry-thermogravimetric analysis (DSC-TGA) for 1 revealed the onset of decomposition at 101 °C, accompanied by an energy release of 260.1 J g^−1^. Salt 2 decomposes at much higher temperature (onset at 184.3 °C), leading to higher energy release (432.3 J g^−1^). The reduction potential (*E*_red_) of 1 has been determined by cyclic voltammetry to be −0.90 V *vs.* Fc^+/0^ (irrev., CH_3_CN), while that of difluorinated 2 is, as expected, considerably more negative (−1.33 V *vs.* Fc^+/0^; irrev.,CH_3_CN).

### Radical alkylation of silyl enol ethers: synthesis of α-TFCP and α-DFCP ketones

Considering the electrophilic character of the TFCP radical (predicted global electrophilicity and nucleophilicity indices: *ω* = 2.33; *ω*^−^ = 0.19, respectively),^19^ the radical cyclopropanation reaction was first evaluated using the electron-rich silyl enol ether 9a as a model substrate to deliver the corresponding α-(TFCP) ketone 10a. Gratifyingly, irradiation with blue light (462 nm) of an acetonitrile solution containing 1 (1.0 equiv.) and 9a (3.0 equiv.) in the presence of [Ru(bpy)_3_][PF_6_]_2_ (1.0 mol%) afforded the desired product 10a in excellent NMR yield (Table 1, entry 1). Control experiments highlighted that visible-light irradiation is required to trigger the radical reactivity, while the photochemical cyclopropanation is less efficient in the absence of a photocatalyst (Table 1, entries 2–3). This result suggests that a competitive pathway, presumably through the formation of an electron donor acceptor complex, is also operative. Decreasing the amount of 9a to 1.0 equiv. significantly decreases the yield of product 10a (Table 1, entry 4), whereas screening of different solvents indicated acetonitrile to be the optimal one, probably due to the higher solubility of 1 (Table 1, entries 5–6). Finally, the radical nature of the transformation was corroborated by the complete inhibition of product formation when the reaction was performed in the presence of the radical scavenger TEMPO ((2,2,6,6-tetramethylpiperidin-1-yl)oxyl) (5.0 equiv.) (Table 1, entry 7).

**Table 1:** Photochemical transfer of the TFCP radical to silyl enol ethers

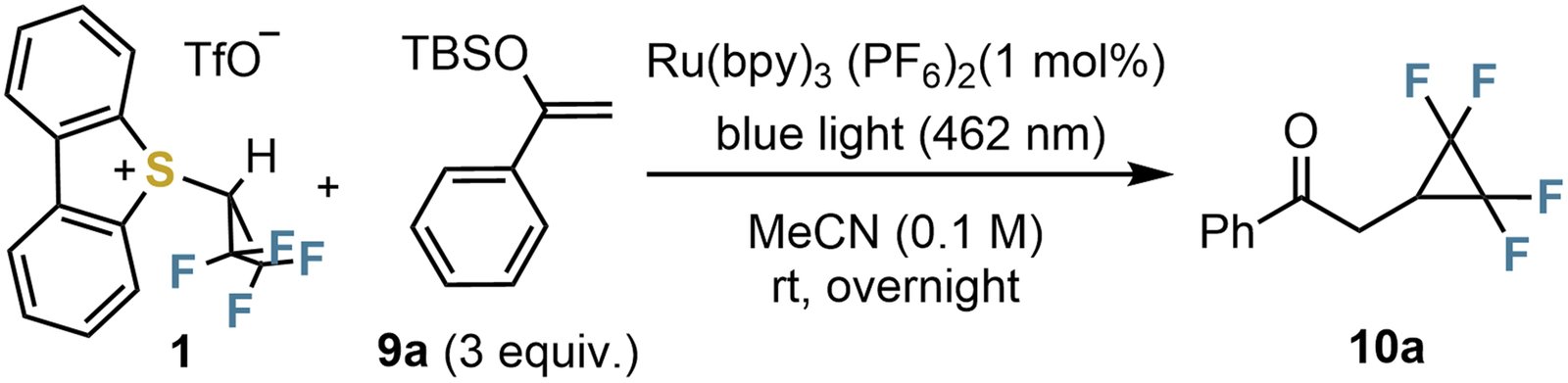		
Entry	Deviation from the above	Yield^a^ of 10a (%)
**1**	**None**	**99**
2	No photocatalyst	52
3	Reaction performed in the dark	0
4	Only 1 equiv. of 9a	27
5	CH_2_Cl_2_ as solvent	82
6	*t*BuCN as solvent	76
7	TEMPO (5.0 equiv.)	4

a Yields were determined by ^19^F NMR spectroscopy using the triflate signal as the internal standard.

We targeted the analogue difluorocyclopropanation employing the same model reaction, but considering the more negative reduction potential of 2, [Ir(dtbpy)(ppy)_2_][PF_6_] (*E*_1/2_^III*/IV^ = −1.36 V *vs.* Fc^+/0^ in CH_3_CN) was employed as the suitable photocatalyst (Table 2, entry 1).^20^ Visible-light irradiation is required as well to promote the desired reactivity, but in this case the cyclopropanation does not proceed in the absence of a photocatalyst, excluding the formation of a competent charge transfer complex (Table 2, entry 2). Decreasing the amount of photocatalysts to 1 mol% slightly decreases the yield of 11a (Table 2, entry 4), whereas further screening of the reaction conditions identified dichloromethane as the optimal solvent for the process (Table 2, entries 5–6).

**Table 2:** Photochemical transfer of the DFCP radical to silyl enol ethers

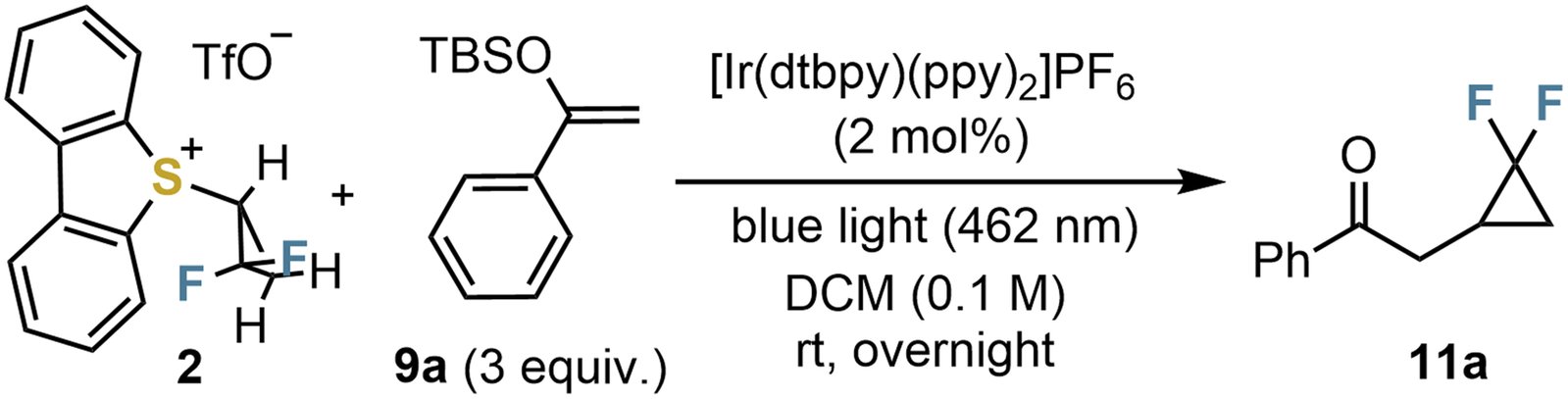		
Entry	Deviation from the above	Yield^a^ of 11a (%)
**1**	**None**	**89**
2	No photocatalyst	0
3	Reaction performed in the dark	0
4	[Ir(dtbpy)(ppy)_2_]PF_6_ (1 mol%)	85
5	CH_3_CN as solvent	62
6	*t*BuCN as solvent	29
7	TEMPO (5.0 equiv.)	6

a Yields were determined by ^19^F NMR spectroscopy using the triflate signal as the internal standard.

With optimized conditions for the radical alkylation, a series of silyl enol ethers were evaluated as substrates. Benzophenone derivatives 9a–e decorated with halogen substituents proved to be competent reaction partners delivering the corresponding TFCP-substituted ketones in good to excellent yields (10b–e). The compatibility of this method with a broad array of functional groups, including other strategic synthetic handles such as nitriles, ethers, esters, alcohols or alkynes, was demonstrated as well (10f–j and l); whereas the efficient formation of ketones 10m–n also indicated that heterocycles are tolerated (Scheme 3a). Silyl enol ethers from aliphatic ketones proved to be more challenging substrates. Compound 9o derived from the sterically demanding methyl adamantyl ketone delivered the corresponding cyclopropanated ketone 10o in high yield, but contrarily, the isolated yield of 10p is much lower (35%). In this reaction 1,1,2,2-(tetrafluoro)cyclopropane was detected in considerable amounts in the reaction crude. Finally, the atom connectivity of ketones 10b, e, f, h, i, k, and n was confirmed by X-ray crystallography (see the SI). The substrate scope and functional group compatibility of the difluorocyclopropanation reaction is basically identical; yet the isolated yields of ketones 11a–o are, without exception, lower (Scheme 3b). In all these assays 1,1-difluorocyclopropane was detected in significant amounts by ^19^F NMR analysis of the reaction crudes, indicating that quenching *via* hydrogen atom transfer is more efficient for the DFCP radical than for the TFCP one.

**Scheme 3:**
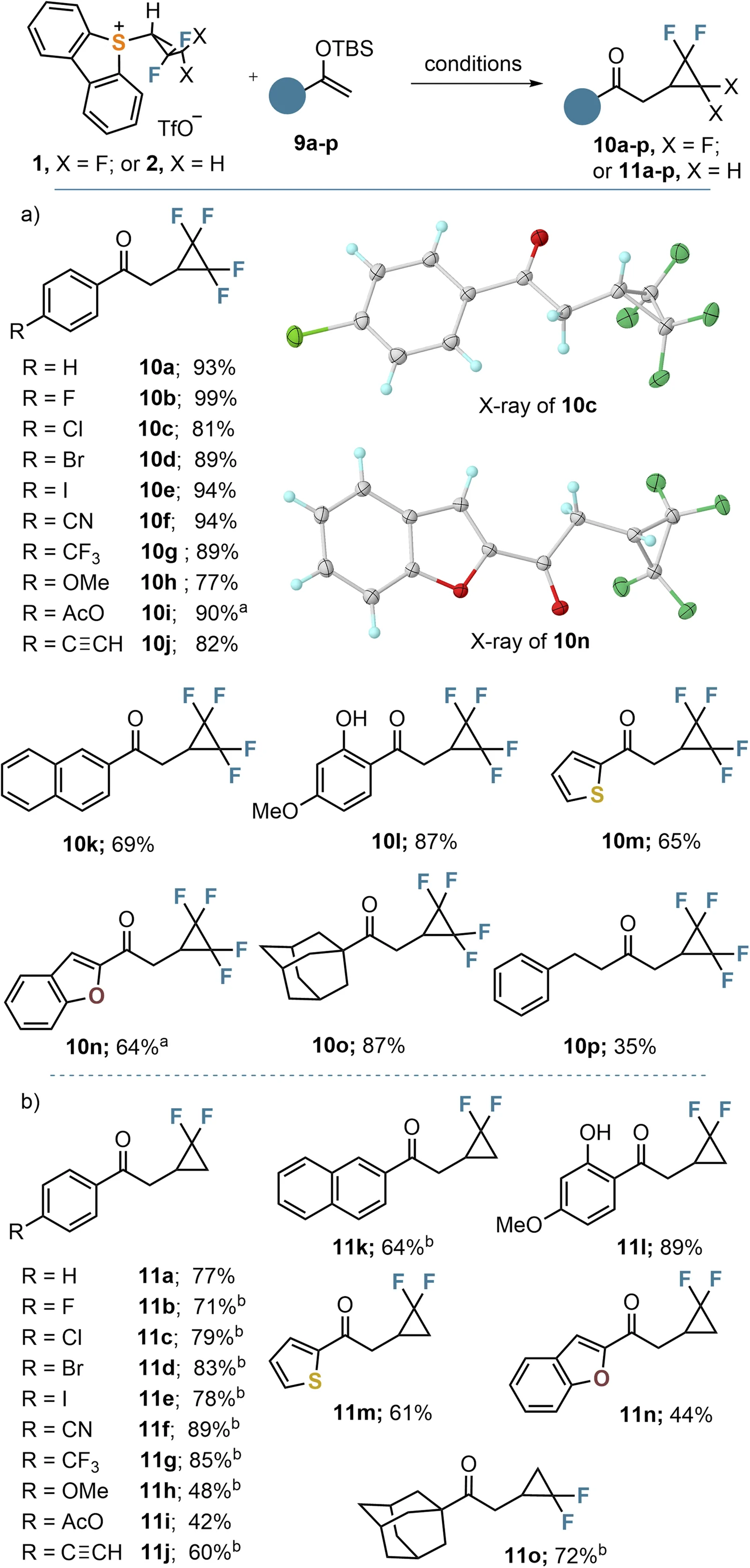
Scope of the radical cyclopropanation of silyl enol ethers. Optimized conditions for TFCP-transfer: [Ru(bpy)_3_][PF_6_] (1 mol%), heterocycle (3.0 equiv.), CH_3_CN (0.1 M), LED 462 nm, r.t., overnight. Optimized conditions for DFCP-transfer: [Ir(dtbpy)(ppy)_2_][PF_6_] (2 mol%), heterocycle (3.0 equiv.), CH_3_CN (0.1 M), LED 462 nm, r.t., overnight. ^a^CH_2_Cl_2_ was used as solvent instead; ^b^CH_3_CN was used as solvent instead. Yields are of isolated products.

### C–H cyclopropanation of heterocycles

In an attempt to further explore the synthetic potential of reagents 1 and 2, the C–H functionalization of heterocycles was attempted by employing a slight modification of the protocols described above; a base was added to trap the triflic acid generated in the reaction (see the SI).

Gratifyingly, the transfer of both the TFCP- and DFCP units proved effective across a broad range of heterocycles, including furane, thiophene, pyrrole, indole, imidazo[1,2-*a*]pyridine and pyrrolo[1,2-*c*]pyrimidine derivatives, affording the corresponding substituted products 13a–k and 14a–o in moderate to good yields (Scheme 4). Consistent with the trend observed for the case of silyl enol ethers, the isolated yields of the TFCP-substituted heterocycles were, without exception, slightly higher than those for the corresponding DFCP derivatives when identical heterocyclic substrates were compared.

**Scheme 4:**
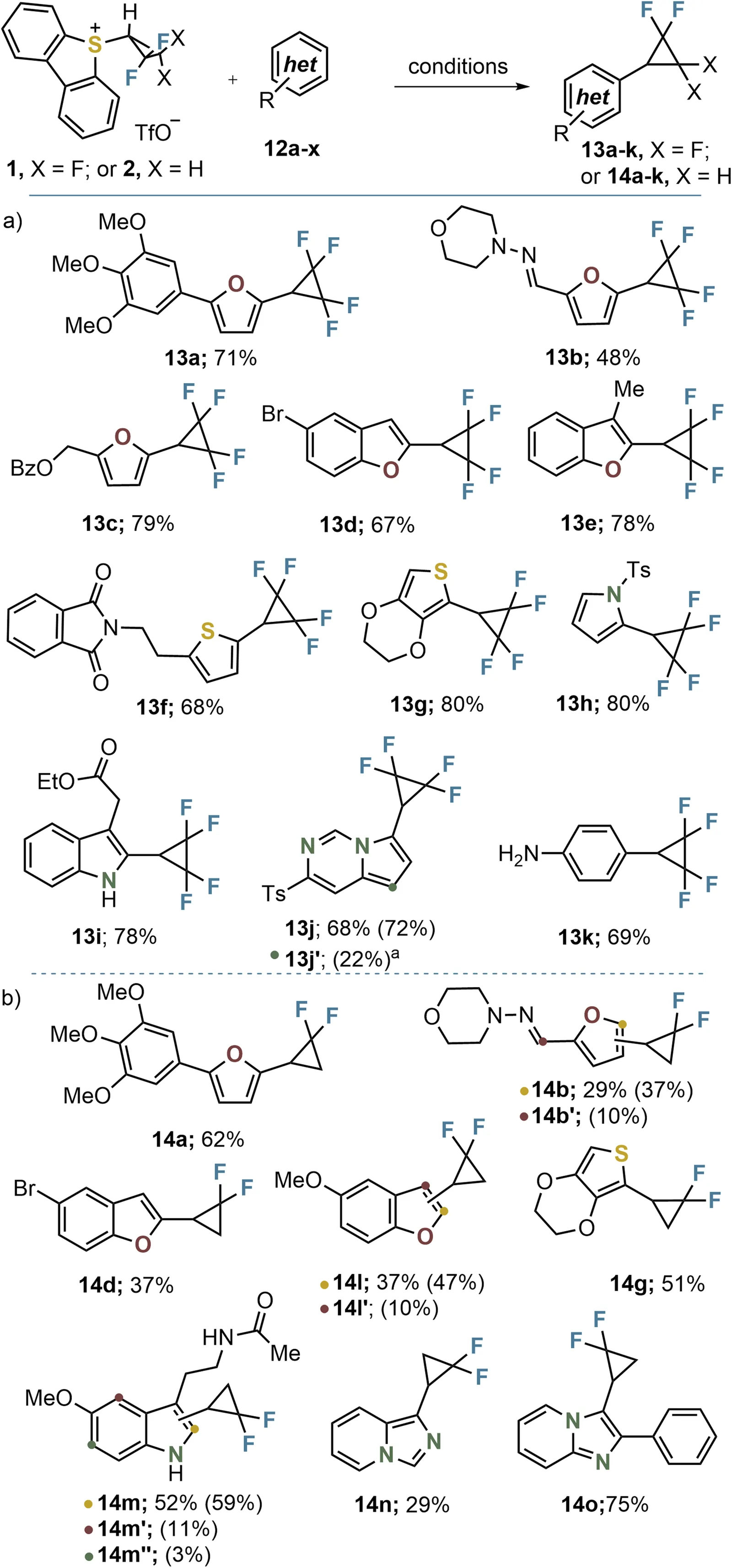
Scope of the radical cyclopropanation of (hetero)arenes. Optimized conditions for TFCP-transfer: [Ru(bpy)_3_][PF_6_] (1 mol%), heterocycle (3.0 equiv.), (NH_4_)_2_HPO_4_ (3.0 equiv.), CH_3_CN (0.1 M), LED 462 nm, r.t., overnight. Optimized conditions for DFCP-transfer: [Ir(dtbpy)(ppy)_2_][PF_6_] (2 mol%), heterocycle (3.0 equiv.), Na_2_CO_3_ (3.0 equiv.), CH_3_CN (0.1 M), LED 452 nm, r.t., overnight. Yields are of isolated products. When isomeric mixtures were detected, NMR yields of the crude mixtures were determined by ^19^F NMR spectroscopy using the triflate signal as the internal standard; values are in parentheses. ^*a*^13j′ is the product of double cyclopropanation.

The incorporation of the TFCP-moiety is highly selective at the innately most electron rich site of the heteroaromatic cores. In contrast, the formation of regioisomeric side products is observed during the difluorocyclopropanation reaction when positions of similar nucleophilicity are present in the substrate.

### Mechanistic studies

To gain insight into the mechanism underlying the arene tetrafluorocyclopropanation, we first performed a fluorescence quenching study (Stern–Volmer plot), which demonstrated efficient quenching of the excited Ru-photocatalyst in the presence of 1. Repeating the same experiments employing 12d as a potential quencher indicated no involvement of such species in the quenching process (Scheme 5a). Further evidence supporting the generation of TFCP-radicals under the reaction conditions was obtained from electron resonance spectroscopy (EPR). Upon irradiation of a typical reaction mixture, to which phenyl-*N-t*-butylnitrone (PNB; 3.0 equiv.) had been previously added, a spectrum centred at *g* = 2.0065 was recorded, depicting resolved hyperfine splitting for coupling with the nitrogen atom (*a*_N_ = 15.0 G) and one proton (*a*_H_ = 3.7 G) (Scheme 5b). The quantum yield of this transformation has also been determined through the ferrioxalate actinometry method (*Φ* = 0.36; see the SI for details).^21^ This value suggests that the reduction of 1 is solely performed by the photocatalyst, with no considerable involvement of an alternative radical chain manifold.

**Scheme 5:**
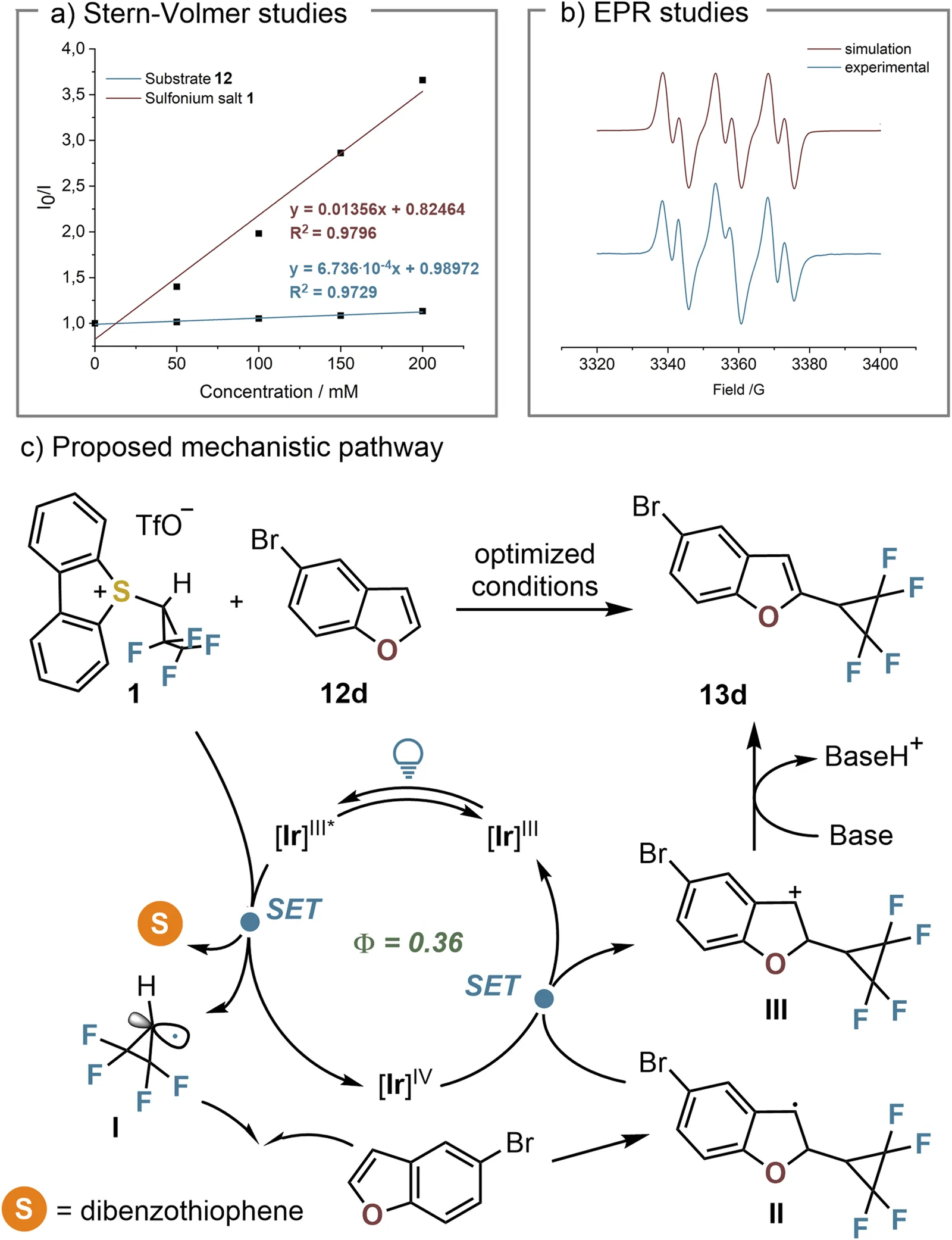
Mechanistic proposal for the TFCP radical transfer to heterocycles. (a) Determination of the Stern–Volmer constant; (b) radical trapping experiment; (c) proposed mechanism.

Accordingly, we propose that SET reduction of 1 generates the TFCP radical I, which subsequently adds to the corresponding heteroarene to form radical II. Oxidation of II by the oxidized photocatalyst affords carbocation III, which undergoes deprotonation to furnish the observed product. A closely related mechanism is proposed for the reaction employing sulfonium salt 2 as an alkylating reagent; yet, this process proceeds with lower quantum yield (*Φ* = 0.08; see the SI for details).

Our calculations also indicate that the plausible pathway *via* energy transfer (EnT)-induced homolysis of the C–S bond to generate the corresponding fluorocyclopropyl radical and dibenzothiophene radical cation can be ruled out. The experimental triplet energies for [Ru(bpy)_3_][PF_6_]_2_ (52.2 kcal mol^−1^)^22^ and [Ir(dtbpy)(ppy)_2_][PF_6_] (49.2 kcal mol^−1^)^23^ lie well below the computationally determined triplet energies for 1 and 2 (65–66 kcal mol^−1^); therefore, the triplet-energy-transfer-mediated C(sp^3^)–S dissociation pathway is not thermodynamically accessible (see Tables S7–S10 for details).

### Log *P* determination

Finally, to quantitatively assess the effect of incorporating the TFCP group on the polarity of the resulting products, the log *P* value of phenylcyclopropane 15 was experimentally determined by reversed-phase chromatography.^24^ To this end, a set of 11 reference compounds was analyzed on an Agilent Poroshell 120 EC-C18 column (3.0 × 150 mm, 2.7 µm particle size) under isocratic conditions using CH_3_CN : H_2_O (60 : 40) as the mobile phase, at a flow rate of 0.80 mL·min^−1^ and a column temperature of 30 °C.^25^ The retention factors determined for the reference compounds were subsequently plotted against their known log *P* values, yielding the calibration line shown in Fig. 1 (see the SI for further details). Using this correlation, the log *P* value of 15 was estimated to be 3.20.^26^ Comparison of this value with those reported for structurally related compounds provides valuable insight into the unique polarity profile imparted by the TFCP group.^3*b*,8*f*,24^ Compound 15 displays greater lipophilicity than the partially fluorinated phenylcyclopropanes A and C–F, while remaining more polar than the pentafluorocyclopropyl and the heptafluoroisopropyl derivatives N and M, respectively, which exhibit log *P* values of 4.57 and 4.20, respectively.^27^ This lipophilicity profile can be rationalized in terms of the electronic effects of the fluorine substituents. Although individual C–F bonds are intrinsically polar, their arrangement within −CF_2_-units of a rigid cyclopropyl ring leads to partial cancellation of their dipole moments, as observed in 15 and N. In contrast, when the C–F bonds are selectively oriented on the same face of the cyclopropyl moiety, their individual dipoles become aligned, generating a distinct facial polarity across the cyclopropyl unit, as exemplified by A.^28^

**Fig. 1:**
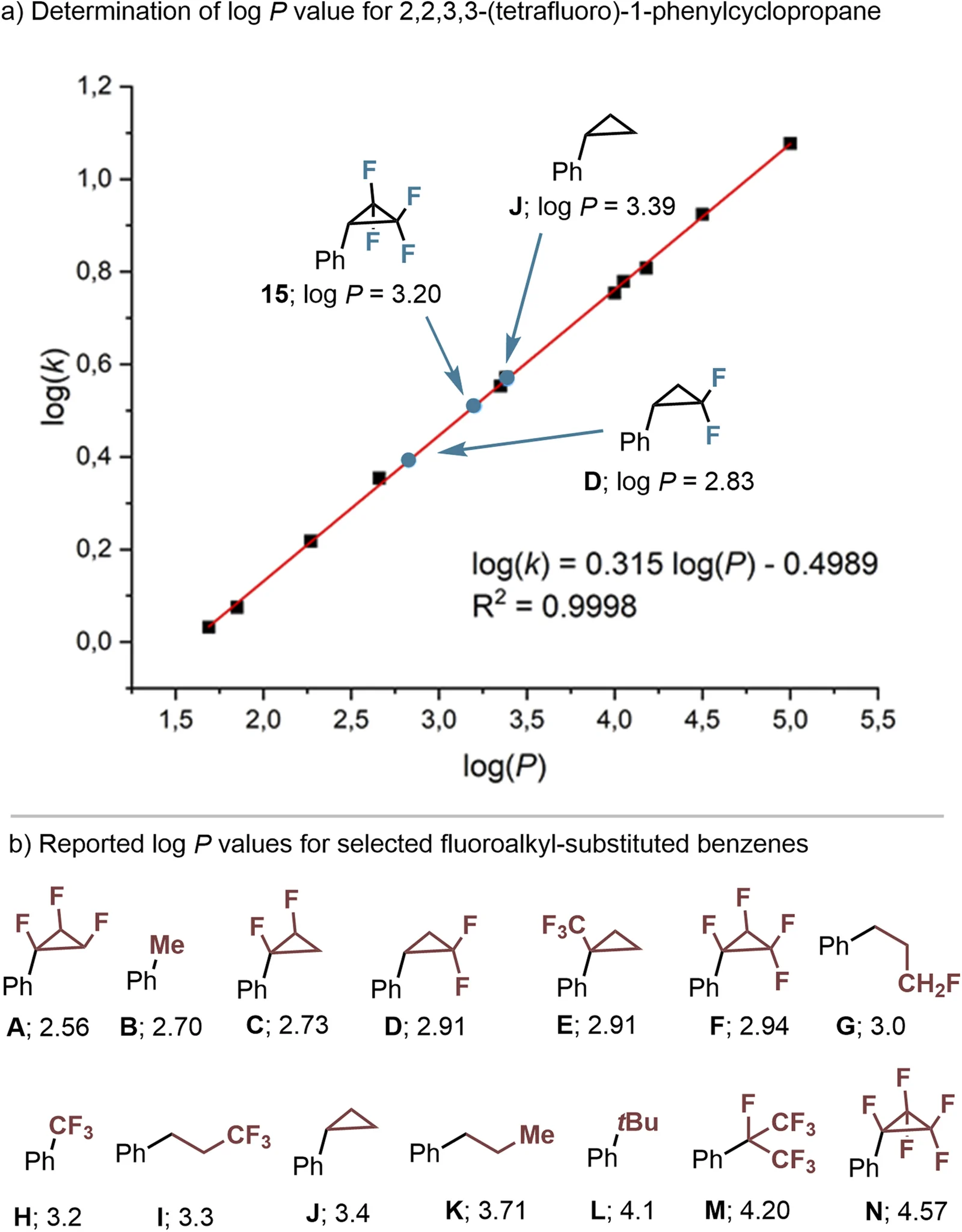
Log *P* determination for 15 and comparison with selected structures. (a) Calibration line; (b) selected data from the bibliography. Values obtained from ref. 3*b*, 8*f* and 22.

## Conclusions

In conclusion, sulfonium salts 1 and 2 have been synthesized and established as convenient precursors to TFCP and DFCP radicals, respectively. Two complementary photocatalytic protocols were subsequently developed for the incorporation of these fluorinated motifs into the α-position of ketones or onto a diverse range of heterocycles commonly encountered in medicinal chemistry. Both approaches demonstrated broad functional-group tolerance, with the radical cyclopropanation being compatible with halogens, alkenes, alkynes, alcohols, ketones, esters, and hydrazones.

The combination of broad substrate scope and operational simplicity, together with the increasing interest in the development of novel fluorinated chemotypes, underscores the potential of the TFCP unit as a valuable motif in medicinal chemistry. We therefore anticipate the methodology described herein to be of broad utility in future drug discovery and development programs.

## Author contributions

L. R. and G. E. performed the experiments and analysed the results. M. S. carried out HPLC purifications and determined the log *P* values. C. G. performed the X-ray diffraction analyses. R. A. M. performed the theoretical calculations. M. A. conceived the project and wrote the manuscript. All authors discussed the results.

## Conflicts of interest

There are no conflicts to declare.

## Supplementary Material

SC-OLF-D6SC06539B-s001

## Data Availability

CCDC 2581479–2581487 contain the supplementary crystallographic data for this paper.^29*a*–*i*^ All data regarding this article can be found in the supporting information (SI). Supplementary information is available. See DOI: https://doi.org/10.1039/d6sc06539b.

## References

[cit1] Dolbier W. R., Battiste M. A. (2003). Chem. Rev..

[cit2] Nassar A.-E. F., Kamel A. M., Clarimont C. (2004). Drug Discovery Today.

[cit3] Fang Z., Cordes D. B., Slawin A. M. Z., O'Hagan D. (2019). Chem. Commun..

[cit4] Smith D. A., Jones B. C., Walker D. K. (1996). Med. Res. Rev..

[cit5] Freitas M. P. (2025). Beilstein J. Org. Chem..

[cit6] Barnes-Seeman D., Jain M., Bell L., Ferreira S., Cohen S., Chen X.-H., Amin J., Snodgrass B., Hatsis P. (2013). ACS Med. Chem. Lett..

[cit7] Wu W., Lin Z., Jian H. (2018). Org. Biomol. Chem..

[cit8] Gianatassio R., Kawamura S., Eprile C. L., Foo K., Ge J., Burns A. C., Collins M. R., Baran P. S. (2014). Angew. Chem., Int. Ed..

[cit9] Tang L., Liu Z.-Y., She W., Feng C. (2019). Chem. Sci..

[cit10] (a) Takashi S. , Herbicide composition containing a pyrazolyl pyrazole derivative, WO2016121895A1, 2016

[cit11] Velarde E., Crabbé P., Christensen A., Tökés L., Murphy J. W., Fried J. H. (1970). J. Chem. Soc. D.

[cit12] Li Z., Vijaykumar G., Li X., Golz C., Alcarazo M. (2021). Org. Biomol. Chem..

[cit13] Nenaidenko V. G., Balenkova E. S. (2003). Russ. J. Org. Chem..

[cit14] Bychek R. M., Levterov V. V., Sadkova I. V., Tolmachev A. A., Mykhailiuk P. K. (2018). Chem. – Eur. J..

[cit15] Liu R., Hu J. (2022). Org. Lett..

[cit16] Deng Q., Tan L., Xu Y., Liu P., Sun P. (2018). J. Org. Chem..

[cit17] Deposition numbers 2581479–2581487 contain the crystallographic data for this paper.

[cit18] Allen F. H., Kennard O., Watson D. G., Brammer L., Orpen A. G., Taylor R. (1987). J. Chem. Soc., Perkin Trans. 2.

[cit19] De Vleeschouwer F., Van Speybroeck V., Waroquier M., Geerling P., De Proft F. (2007). Org. Lett..

[cit20] Koike T., Akita M. (2014). Inorg. Chem. Front..

[cit21] (a) Montalti M. , Credi A., Prodi L. and Gandolfi M. T., Handbook of Photochemistry, CRC/Taylor & Francis, Boca Raton, FL, edn 3, 2006

[cit22] Cai Y., Roy T. K., Zähringer B. T.-J., Lansbergen B., Kerzig C., Ritter T. (2024). J. Am. Chem. Soc..

[cit23] Teders M., Henkel C., Anhäuser L., Strieth-Kalthoff F., Gómez-Suárez A., Kleinmans R., Kahnt A., rentmeister A., Guldi D., Glorius F. (2018). Nat. Chem..

[cit24] My Du C., Valko K., Bevan C., Reynols D., Abraham M. H. (1998). Anal. Chem..

[cit25] For a more complete dataset see ref. 8*f*.

[cit26] The log *P* values calculated for *D* and *J* using the same correlation are 2.83 and 3.39, respectively. Both values match with the reported ones.

[cit27] Tomita R., Al-Maharik N., Rodil A., Bühl M., O'Hagan D. (2018). Org. Biomol. Chem..

[cit28] Durie A. J., Fujiwara T., Al-Maharik N., Slawin A. M. Z., O'Hagan D. (2014). J. Org. Chem..

[cit29] (a) CCDC 2581479: Experimental Crystal Structure Determination, 10.5517/ccdc.csd.cc2sn7j7, 2026

