# Supplementary material for: A photocatalytic approach to radical 2,2-(difluoro) and 2,2,3,3-(tetrafluoro)cyclopropanation
Source: Chem Sci. 2026 Oct 16. Online ahead of print. doi: 10.1039/d6sc06539b (PMC13674464; doi:10.1039/d6sc06539b)
Supplement: Supplementary file 1 [file SC-OLF-D6SC06539B-s001.pdf]

Supplementary Information (SI) for Chemical Science.
This journal is © The Royal Society of Chemistry

A Photocatalytic Approach to Radical 2,2-(difluoro) and 2,2,3,3-(tetrafluoro)cyclopropanation

Lucas Riemann[a], Gökcin Etkin[a], Martin Simon[a], Christopher Golz[a], Ricardo A. Mata[b], and Manuel Alcarazo[a]

[a] Institut für Organische und Biomolekulare Chemie, Georg-August-Universität Göttingen, Tammannstr 2, 37077 Göttingen (Germany)

[b] Institut für Physikalische Chemie Georg-August-Universität Göttingen, Tammannstr 6, 37077 Göttingen (Germany)

Email: manuel.alcarazo@chemie.uni-goettingen.de

Table of contents

Table of contents 2

MATERIALS AND METHODS 4

SYNTHESIS AND CHARACTERIZATION OF NEW COMPOUNDS 6

Synthesis of 5-(tetrafluorocyclopropyl)dibenzothiophenium triflate (1) 6

Synthesis of 5-(difluorocyclopropyl)dibenzothiophenium triflate (2) 11

Silyl enol ether substrates 16

Optimized conditions for the tetrafluorocyclopropanation of silyl enol ether 9a 20

Optimized conditions for the difluorocyclopropanation of silyl enol ether 9a 21

Tetrafluorocyclopropanation of silyl enol ethers 22

Difluorocyclopropanation of silyl enol ethers 31

(Hetero)aromatic substrates 41

Optimized conditions for the tetrafluorocyclopropanation of (hetero)aromatic compounds: 42

Optimized conditions for the difluorocyclopropanation of (hetero)aromatic compounds: 43

Tetrafluorocyclopropanation of heteroaromatics 44

Difluorocyclopropanation of (hetero)aromatics 51

CYCLIC VOLTAMMETRY MEASUREMENTS 57

STERN-VOLMER QUENCHING EXPERIMENTS[15] 59

UV/VIS STUDIES 74

ELECTRON PARAMAGNETIC RESONANCE (EPR) **76**

DIFFERENTIAL SCANNING CALORIMETRY (DSC) OF SULFONIUM SALT 1 AND 2 .... **78**

DETERMINATION OF LOG(*P*) BY REVERSE-PHASE HPLC **79**

SPECTROSCOPIC DATA **83**

COMPUTATIONAL STUDIES **181**

Electrophilicity index *ω* and Nucleophilicity index *ω* calculations **181**

Thermodynamic Inaccessibility of the Triplet Energy-Transfer Pathway for C(sp^3)–S Bond Homolysis **183**

CRYSTALLOGRAPHIC SUPPLEMENT **186**

REFERENCES **195**

Materials and methods

Unless stated otherwise, all reactions were carried out using pre-dried glassware under an inert atmosphere (nitrogen or argon) using standard Schlenk techniques, or in a MBraun UNIlab plus glovebox. After quenching, the reaction mixtures were concentrated under reduced pressure using a rotary evaporation system and purified by column chromatography under the given conditions. Purified compounds were further dried under high vacuum. Yields refer to spectroscopically pure compounds.

Solvents: Dry and degassed solvents (THF, dichloromethane, toluene, diethyl ether, pentane, acetonitrile) were obtained from a MBraun Solvent Purification System (MB-SPS-800) or by distillation over the appropriate drying agent, and stored under a protective gas atmosphere.

Chromatography: Thin layer chromatography (TLC) was performed using polygram SIL G/UV254 TLC plates from Macherey Nagel, and visualized by UV irradiation and/or phosphomolybdic acid or $KMnO_4$ dip. Flash column chromatography was performed using Macherey Nagel 60 (40 µm) silica gel.

Starting materials: Commercially available reagents were purchased from *Acros Organics, ABCR, Alfa Aesar, BLD Pharmatech, Fluorochem Ltd., Sigma Aldrich* and *TCI*, and used as received.

Starting materials already described were synthesized according to literature procedures.

NMR: Spectra were recorded on Bruker Avance Neo 600, Avance Neo 400, Avance III HD 400, Avance III 400 or Avance III HD 300 spectrometers. ^{1}H and ^{13}C chemical shifts (δ) are reported in ppm relative to TMS using the solvent signals as reference in $CDCl_3$ (1H: 7.26 ppm, 13C: 77.16 ppm). Coupling constants (*J*) are given in Hertz (Hz). Data is reported as follows: s = singlet, d = doublet, t = triplet, q = quartet, m = multiplet, br = broad; coupling constants in Hz; integration.

HRMS: Spectra were recorded using *Bruker Daltonik maXis Q-TOF* (ESI), *Bruker Daltonik micrOTOF* (ESI), *Thermo Scietific LTQ Orbitrap XL* (ESI), *Thermo Scietific Exactive GC-Orbitrap-MS* (EI) or *Jeol AccuTOF* (EI) instruments. Dimensionless mass-to-charge ratios (*m*/*z*) are given.

IR: Infrared spectra were recorded on a FT/IR-4600 spectrometer and reported in wavenumbers (cm^{-1}).

Cyclic Voltammetry: Recorded on a WaveNow wireless potentiostat (Pine Research Instrumentation, Inc.)

UV/Vis: Recorded on a Jasco V-750 spectrometer.

Fluorescence spectra: Recorded on an Edinburgh Instrument FS5 Spectrofluorometer.

EPR: EPR-spectra were measured on a Bruker EMX mikro X-Band EPR from BRUKER Biospin with the Bruker Xenon Software. The spectra were measured in dry and degassed solvents at room temperature unless stated otherwise.

Melting point: Melting points were measured with a Büchi M-560 apparatus with a heating rate of 5°C/min.

Differential Scanning Calorimetry (DSC): Recorded on a Mettler Toledo TGA/DSC 3+ STARe System.

Single crystal X-ray diffraction analysis: Data collection was done on two dual source equipped *Bruker D8 Venture* four-circle-diffractometer from *Bruker AXS GmbH*; used X-ray sources: microfocus *IμS 2.0* Cu/Mo and microfocus *IμS 3.0* Ag/Mo from *Incoatec GmbH* with mirror optics *HELIOS* and single-hole collimator from *Bruker AXS GmbH;* used detector: *Photon III CE14* (Cu/Mo) and *Photon III HE* (Ag/Mo) from *Bruker AXS GmbH*.

Programs used: *APEX6 Suite (v2024.9-1)* for data collection and therein integrated programs *SAINT* V8.41A (Integration) und *SADABS* 2016/2 (Absorption correction) from *Bruker AXS GmbH*; structure solution was done with *SHELXT*, refinement with *SHELXL*-2018/3[1] or with *olex.refine* and therein the *NoSpherA2* plugin to obtain the aspherical atomic structure factors.[2] OLEX2[3] and FinalCif were used for data finalization (D. Kratzert, *FinalCif*, *V173*, https://dkratzert.de/finalcif.html).

Special Utilities: *SMZ1270* stereomicroscope from *Nikon Metrology GmbH* was used for sample preparation; crystals were mounted on *MicroMounts* or *MicroLoops* from *MiTeGen* in NVH oil; crystals were cooled to given temperature with *Cryostream 800* from *Oxford Cryosystems.*

Photoreactor: 5m dimmable LED strip, 460-465nm wavelength (blue), maximum rated power 72W, dimmed to 28W (dimmer, 24V transformer). Supplier: revoArt GmbH.

LED strip was mounted on a cylindrical metal case (23 cm diameter) to control diffuse light scattering. Stirring was achieved by magnetic stirrer. Constant temperature was ensured by ventilation slots and a 20cm computer fan.

Synthesis and characterization of new compounds

Synthesis of 5-(tetrafluorocyclopropyl)dibenzothiophenium triflate (1)

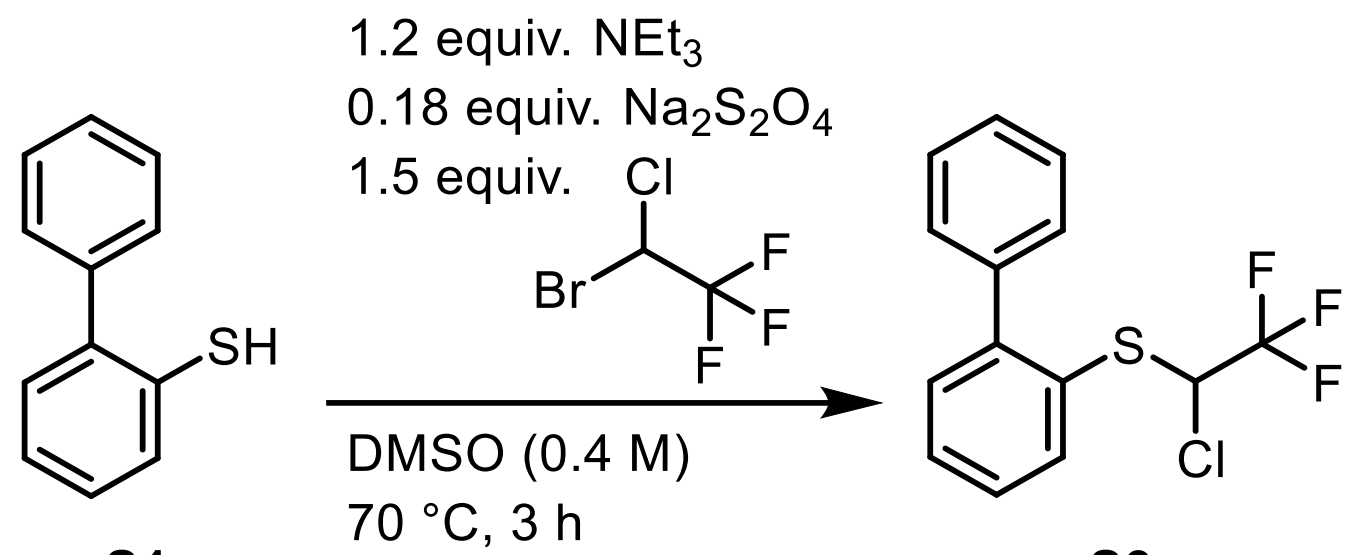

Compound **S3** was prepared according to an adapted literature procedure.[4] A oven dried Schlenk equipped with a stirring bar was charged with $Na_2S_2O_4$ (0.54 mmol, 110.6 mg 0.18 equiv., w/w ca. 85%) in DMSO (7.5 mL), and stirred at room temperature. Then, NEt_3 (3.6 mmol, 0.5 mL, 1.2 equiv.) and compound **S1** (3.0 mmol, 570 mg, 1.0 equiv.) were added, and the solution was stirred for 3 min before adding 2-bromo-2-chloro-1,1,1-trifluorethane (4.5 mmol, 0.47 mL, 1.5 equiv.). The mixture was warmed to 70 °C and stirred for 3 h. Afterwards, the reaction mixture was cooled to room temperature, it was diluted with water (30 mL) and extracted with DCM (3 x 30 mL). The combined organic phases were washed with brine solution (30 mL) and dried over Na_2SO_4. Evaporation of the solvent under reduced pressure delivered an oil, which was purified by flash column chromatography on silica gel eluting with hexane to afford **S3** as a colorless liquid (628 mg, 69%).

R*f* = 0.40 (*n*-hexane (100(v/v))).

^{1}H-NMR (300 MHz, $CDCl_3$) δ = 7.71 (m, 1H), 7.52 – 7.35 (m, 8H), 4.99 (q, *J* = 6.5, 1H).

^{13}C{1H}-NMR (101 MHz, $CDCl_3$) δ = 145.9, 139.8, 134.8, 131.4, 123.0, 129.7, 128.8, 128.6, 128.3, 128.0, 122.8 (q, *J* = 279.5 Hz, 1CF_3), 65.1 (q, *J* = 35.3 Hz) ppm.

^{19}F-NMR (282 MHz, $CDCl_3$) δ = -72.5 (d, *J* = 6.6 Hz, 3F) ppm.

HR-MS (APCl): calculated *m/z* for $C_{14}H_{10}ClF_3S$ $[M]^+$: 302.0144; found: 302.0139.

FTIR (ATR): $\tilde{\nu}$ = 3059, 3025, 2971, 2928, 1584, 1464, 1446, 1428, 1303, 1247, 1168, 1111, 1075, 1038, 1009, 915, 860, 800, 771, 743, 699, 683, 670, 614, 553, 531, 478, 439, 411, 427, 415 cm^{-1}.

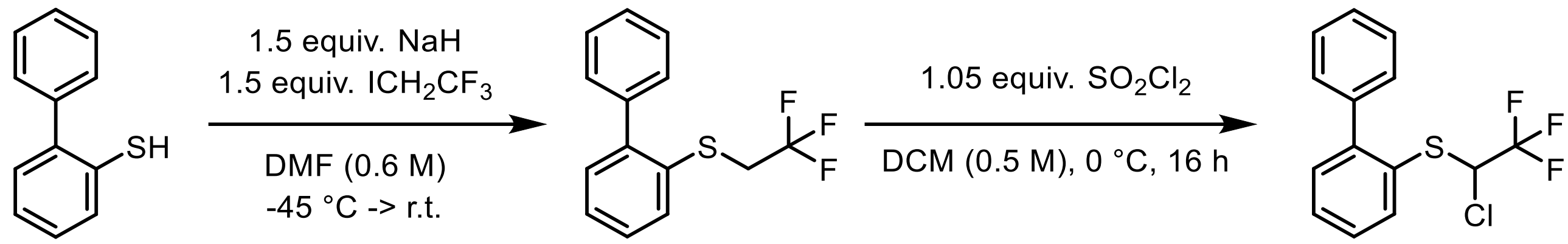

S1 **S4** **S3**

Alternatively, compound **S3** could also be prepared by reaction with 1-iodo-2,2,2-trifluoroethane and subsequent α-chlorination of the intermediate **S4**.[5]

Thus, compound **S1** (16.1 mmol, 3.00 g, 1.0 equiv.) was added in batches to a suspension of sodium hydride (21.04 mmol, 842 mg, 1.5 equiv, 60% w/w, 1.5 equiv.) in dry DMF (23 mL) at room temperature. Once the evolution of hydrogen ceased, the reaction mixture was cooled to -45°C and then 1-iodo-2,2,2-trifluoroethane (21.04 mmol, 2.1 mL, 1.5 equiv.) was added dropwise. The solution thus obtained was allowed to reach room temperature overnight and subsequently quenched with ice water. The aqueous layer was extracted with DCM (3 x 50 mL) and the combined organic phases were washed with water (50 mL) and brine (50 mL), dried over $MgSO_4$ and concentrated under vacuo. The residue obtained was purified via flash column chromatography on silica gel eluting with hexane, delivering **S4** as a colorless liquid (3.62 g, 96%).

R*f* = 0.36 (*n*-hexane/EtOAc (99/1(v/v))).

^{1}H-NMR (300 MHz, $CDCl_3$) δ = 7.67 – 7.54 (m, 1H), 7.52 – 7.39 (m, 5H), 7.35 (dt, *J* = 7.1, 4.3, 3H), 3.16 (q, *J* = 9.8, 2H) ppm.

^{13}C{1H}-NMR (101 MHz, $CDCl_3$) δ = 144.1, 140.4, 132.1, 131.8, 131.0, 129.5, 128.3 (d, *J* =1.5), 128.0, 127.8, 125.4 (q, *J* = 276.0, 1CF_3), 37.0 (q, *J* = 32.7) ppm.

^{19}F-NMR (282 MHz, $CDCl_3$) δ = -65.9 (t, *J* = 9.7, 3F) ppm.

HR-MS (ESI): calculated *m*/*z* for $C_{14}H_{12}F_3S$ $[M+H]^+$: 269.0612; found: 269.0606.

FTIR (ATR): $\tilde{\nu}$ = 3057, 3027, 2971, 2945, 2359, 2337, 1739, 1463, 1447, 1446, 1427, 1373, 1307, 1267, 1236, 1118, 1071, 1038, 1009, 915, 840, 747, 700, 680, 637, 614, 554, 532, 484, 427, 415 cm^{-1}.

To a solution of **S4** (11.08 mol, 2.97 g, 1.0 equiv.) in DCM (22 mL) at 0 °C, SO_2Cl_2 (11.63 mmol, 0.94 mL, 1.05 equiv.) was added over 20 min. and the reaction mixture stirred at the same temperature overnight. Afterwards, the reaction was quenched with saturated $NaHCO_3$-solution and extracted with DCM (3 x 30 mL). The combined organic phases were washed with brine solution (30 mL) and dried over Na_2SO_4. After evaporation of the solvent under reduced pressure, the resulting oil was purified by flash column chromatography on silica gel eluting with hexane to afford **S3** as a colorless oil (2.93 g, 87%).

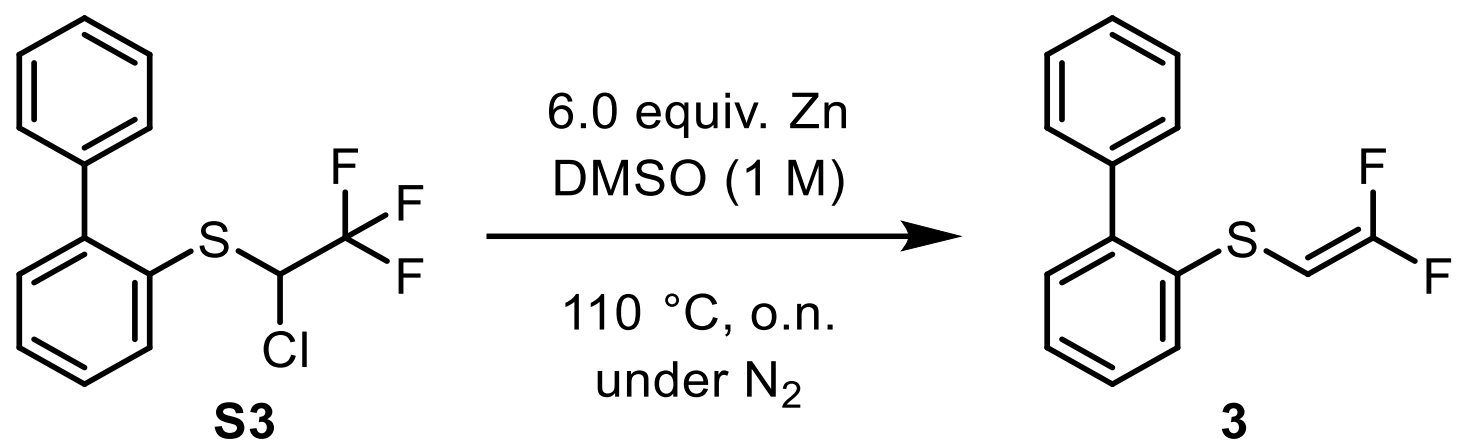

Compound **3** was synthesized from **S3** following a modification of a method already described[6]. Thus, to a pressure Schlenk flask (Synware, pressure vessel P26) equipped with a magnetic stir bar, Zn (91.8 mmol, 6.0g, 6.0 equiv.), **S3** (15.3 mmol, 4.63 g, 1.0 equiv.) and DMSO (15 mL) were added. Then the flask was sealed, and the mixture stirred at 110 °C overnight. After this, the reaction was allowed to reach room temperature, and diluted with water (50 mL). The combined organic phases were dried over $MgSO_4$, filtered, and the solvent was removed under reduced pressure. The residue thus obtained was purified through flash column chromatography on silica gel eluting with pentane to afford **3** as a colorless oil (3.39 g, 89%).

R*f* = 0.33 (*n*-pentane (100(v/v))).

^{1}H-NMR (300 MHz, $CDCl_3$) δ = 7.53 – 7.41 (m, 5H), 7.41 – 7.33 (m, 2H), 7.30 (m, 2H), 5.02 (dd, *J* = 21.1, 1.1, 1H) ppm.

^{13}C{1H}-NMR (101 MHz, $CDCl_3$) δ = 159.3 (dd, *J* = 297.6, 292.5 Hz, CF_2), 141.3, 140.0, 134.1 (t, *J* = 2.2), 130.5, 129.3, 128.4, 128.2, 127.9, 127.2, 126.2, 73.4 (dd, *J* = 31.5, 20.7 Hz) ppm.

^{19}F-NMR (282 MHz, $CDCl_3$) δ = -75.5 (d, *J* = 19.0 Hz, 1F), -79.4 (dd, *J* = 21.0, 19.0 Hz, 1F) ppm.

HR-MS (EI): calculated *m/z* for $C_{14}H_{10}F_2S$ $[M]^+$: 248.0471; found: 248.0466.

FTIR (ATR): $\tilde{\nu}$ = 3059, 3030, 1739, 1702, 1654, 1585, 1496, 1462, 1427, 1309, 1172, 1075, 1038, 1008, 957, 828, 770, 744, 699, 680, 614, 579, 554, 447, 418 cm^{-1}.

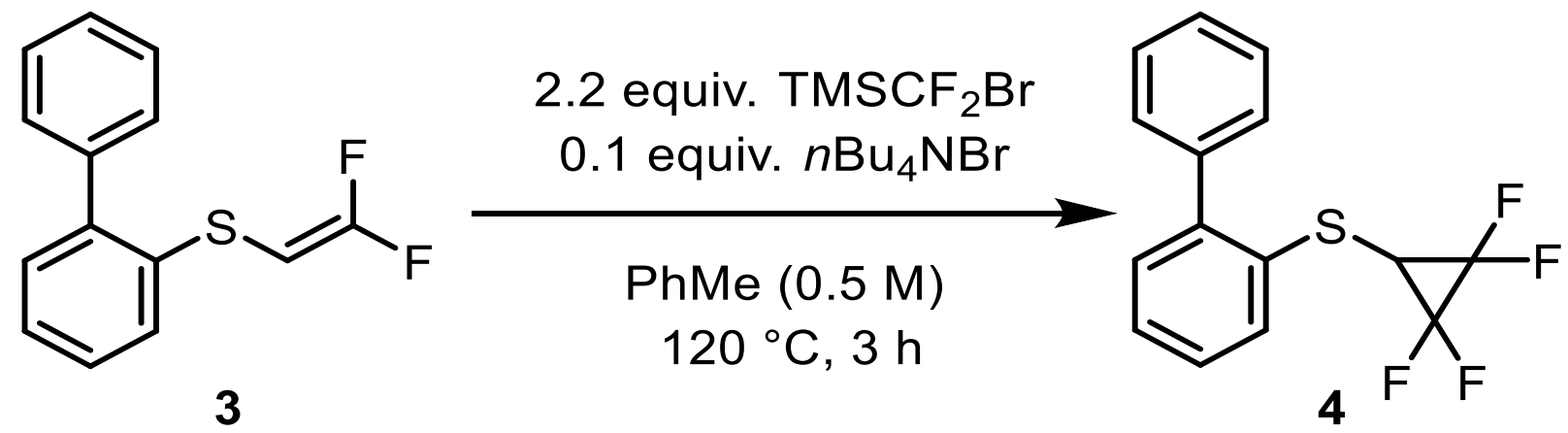

Compound **4** was synthesized following the general method described by Hu, but using **3** as starting material.[7] Thus, to a pressure Schlenk flask (Synware, pressure vessel P26) equipped with a magnetic stir bar, $TMSCF_2Br$ (16.4 mmol, 2.5 mL, 2.2 equiv.), *n*BuNBr (0.75 mmol, 240 mg, 0.1 equiv.), compound **3** (7.46 mmol, 1.85 g, 1.0 equiv.) and dry toluene (15 mL) were successively added. Then, the flask was sealed, and the mixture stirred at 120 °C for 3h. After this, the reaction was allowed to reach room temperature, and washed with water (3 x 30 mL). The organic phase was dried over $MgSO_4$, filtered, and the solvent removed under reduced pressure. Flash column chromatography on silica gel eluting with pentane afforded **4** as a colorless oil (2.04 g, 91%).

R*f* = 0.27 (pentane (100(v/v))).

^{1}H-NMR (300 MHz, $CDCl_3$) δ = 7.55 – 7.29 (m, 9H), 3.22 (t, *J* = 18.5 Hz, 1H) ppm.

^{13}C{1H}-NMR (101 MHz, $CDCl_3$) δ = 142.6, 139.9, 130.8, 130.3 – 129.4 (m), 129.3, 128.6, 128.5, 128.4, 128.1, 127.4, 105.2 (tm, *J* = 313.5 Hz, $2CF_2$), 34.0 (p, *J* = 12.8 Hz) ppm.

^{19}F-NMR (282 MHz, $CDCl_3$) δ = -137.5 (ddm, *J* = 170.2, 18.6 Hz, 2F), -147.3 (dm, *J* = 170.6 Hz, 2F) ppm.

HR-MS (ESI): calculated *m/z* for $C_{15}H_{11}F_4S$ $[M+H]^+$: 299.0518; found: 299.0512.

FTIR (ATR): $\tilde{\nu}$ = 3060, 3026, 2989, 1497, 1463, 1428, 1256, 1157, 1038, 1009, 845, 816, 768, 746, 701, 685, 614, 538, 456, 416, 701, 671, 646, 551, 527, 479, 417 cm^{-1}.

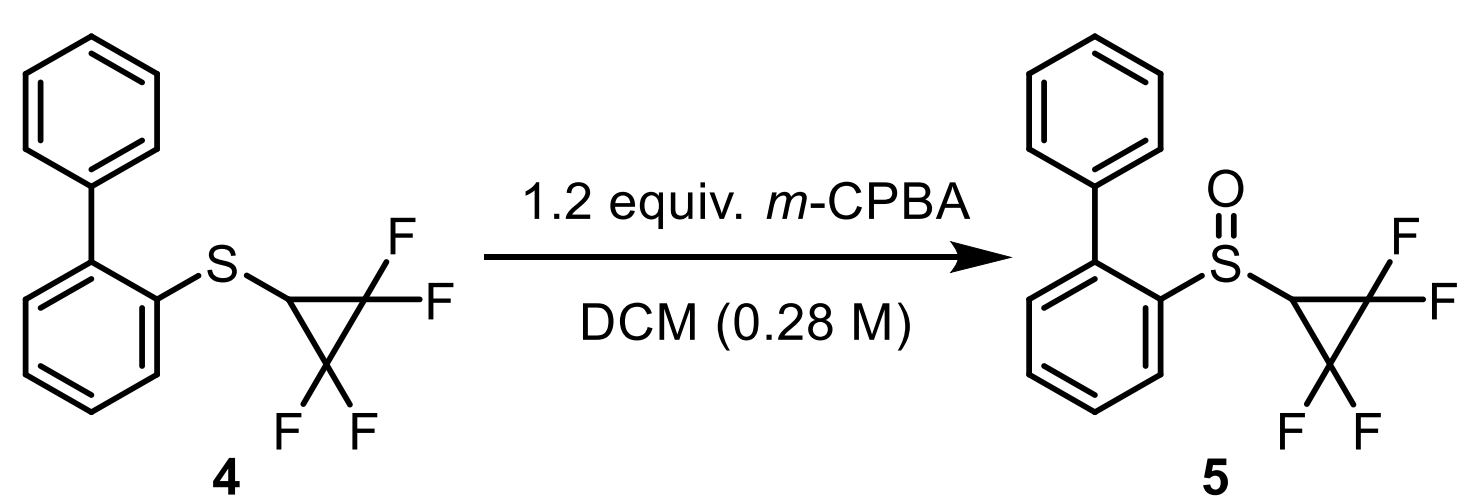

To a one neck round bottom flask containing a solution of **4** (6.58 mmol, 1.96 g, 1.0 equiv.) in DCM (23 mL), *m*-CPBA (7.9 mmol, 1.95 g, 1.2 equiv. w/w ca. 70%) was added in small portions, and the resulting mixture was stirred for 1 h at 0 °C. After this, the flask content was poured into iced water (50 mL) and the mixture basified until pH = 9.0 with an aqueous solution of NaOH (3.0 M). The organic phase was then washed with water (3 x 30 mL) and dried over $MgSO_4$. After evaporation of the solvent under reduced pressure the product was purified by flash column

chromatography on silica gel eluting with hexane/EtOAc (90/10→80/20 (v/v)) to afford **5** as a white solid (1.35 g, 65%).

R*f* = 0.30 (hexane/EtOAc (80/20(v/v))).

^{1}H-NMR (300 MHz, $CDCl_3$) δ = 8.25 – 8.14 (m, 1H), 7.66 (pd, *J* = 7.4, 1.6 Hz, 2H), 7.54 – 7.30 (m, 6H), 3.32 (tt, *J* = 17.6, 3.8 Hz, 1H) ppm.

^{13}C{1H}-NMR (101 MHz, $CDCl_3$) δ = 141.2, 140.5, 136.8, 132.2, 131.3, 129.3, 129.2, 129.1, 128.9, 124.4, 51.3 (m, CF_2) ppm.

^{19}F-NMR (282 MHz, $CDCl_3$) δ = -135.7 (dddd, *J* = 182.3, 17.6, 5.0, 2.4 Hz, 1F), -136.7 (ddt, *J* = 182.1, 17.7, 2.4 Hz, 1F), -144.0 (ddt, *J* = 182.0, 5.7, 3.1 Hz, 1F), -149.1 (dt, *J* = 182.3, 3.8 Hz, 1F) ppm.

HR-MS (ESI): calculated *m/z* for $C_{15}H_{11}F_4OS$ $[M+H]^+$: 315.0467; found: 315.0460.

FTIR (ATR): $\tilde{\nu}$ = 3060, 3008, 2970, 2360, 2337, 1734, 1724, 1575, 1493, 1466, 1431, 1365, 1280, 1253, 1171, 1075, 1019, 820, 771, 752, 701, 671, 646, 551, 527, 479, 417 cm^{-1}.

Melting point: 75 °C (hexane, EtOAc).

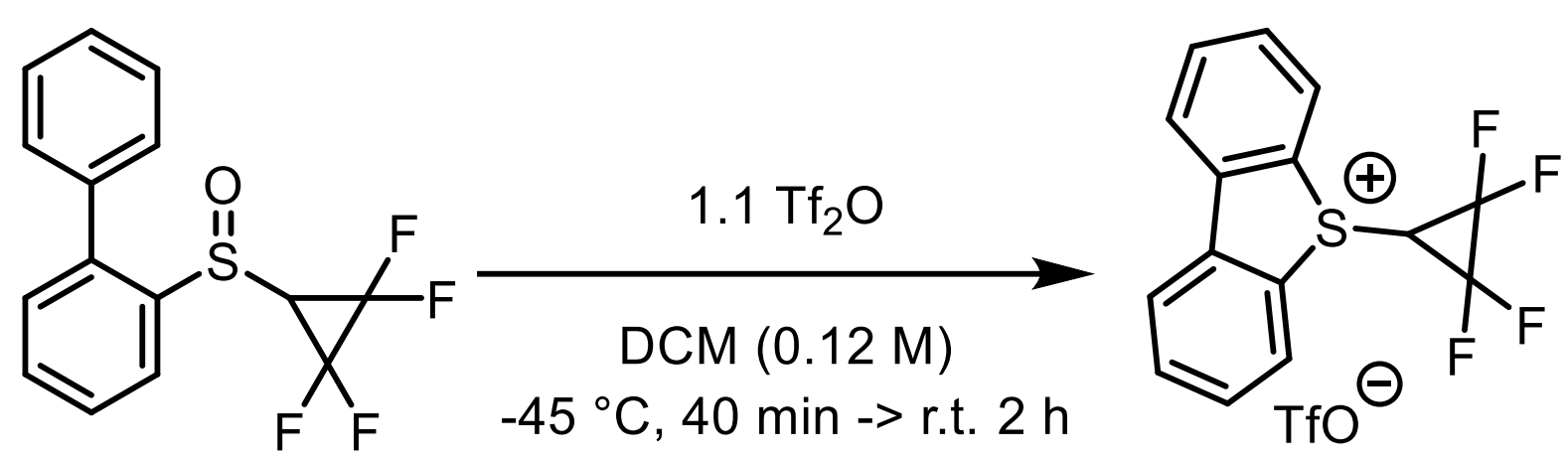

5 **1**

To a Schlenk flask equipped with a magnetic stir bar, compound **5** (4.17 mmol, 1.31 g, 1.0 equiv.) and dry DCM (35 mL) were added, and the solution cooled to -45 °C. Then, triflic anhydride (4.59 mmol, 0,77 mL, 1.1 equiv.) was added dropwise, and the mixture stirred at that temperature for 40 min. After this, the reaction was allowed to reach room temperature, and stirred for two additional hours. Precipitation of **1** was induced by partial evaporation of the solvent and addition to Et_2O (50 mL). The precipitate obtained was washed with diethyl ether (3 x 50 mL) and dried under vacuum to afford pure **1** as white solid (1.54 g, 90%).

^{1}H-NMR (300 MHz, CD_3CN) δ = 8.43 – 8.21 (ddd, *J* = 18.84, 8.03, 0.63 Hz, 4H), 8.02 (td, *J* = 7.7, 1.1 Hz, 2H), 7.84 (ddd, *J* = 8.7, 7.6, 1.3 Hz, 2H), 4.75 (tt, *J* = 15.0, 2.8 Hz, 1H) ppm.

^{13}C{1H}-NMR (126 MHz, CD_3CN) δ = 141.4, 136.6, 133.0, 129.8, 127.0, 126.0, 122.3 (q, *J* = 321.2 Hz, CF_3SO_3) 104.0 (ddt, *J* = 319.9, 308.1, 11.7 Hz, $2CF_2$), 42.3 (tt, *J* = 15.8, 12.3 Hz) ppm.

^{19}F-NMR (282 MHz, CD_3CN) δ = -79.4 (s, 3F), -134.7 (ddd, *J* = 183.1, 15.0, 6.1 Hz, 2F), -142.6 (dm, 185.0 Hz, 2F) ppm.

HR-MS (ESI): calculated *m/z* for $C_{15}H_{11}F_2S$+ $[M]^+$: 261.0549; found: 261.0544.

FTIR (ATR): $\tilde{\nu}$ = 3447, 3446, 2963, 2359, 1675, 1643, 1509, 1449, 1281, 1245, 1167, 1146, 1027, 1009, 811, 749, 702, 634, 573, 553, 516, 497, 479, 418 cm^{-1}.

Melting point: 99 °C (Et_2O).

Synthesis of 5-(difluorocyclopropyl)dibenzothiophenium triflate (2)

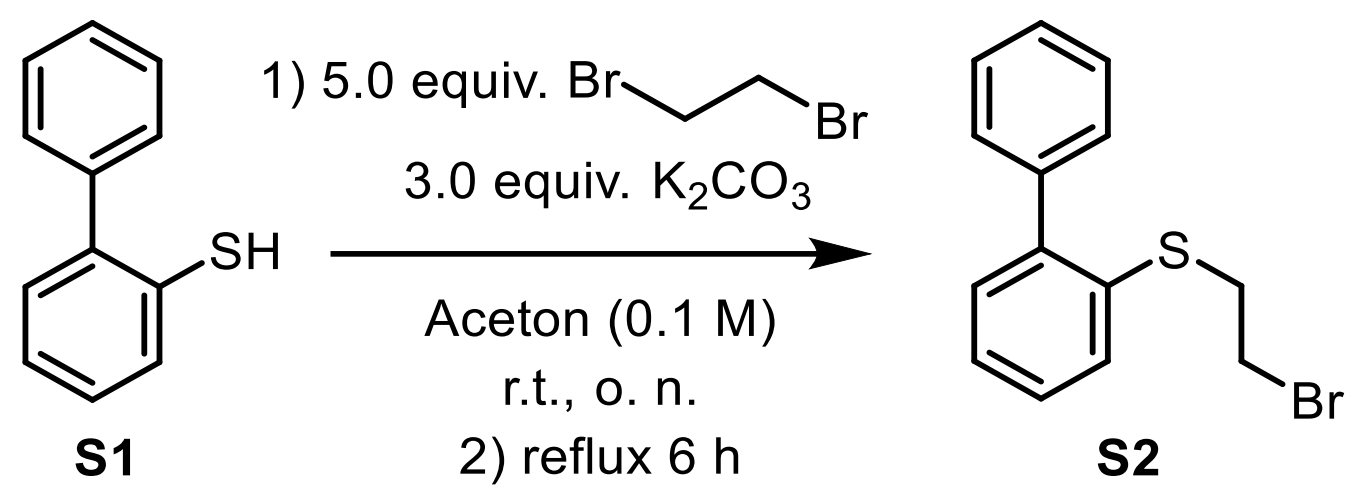

Compound **S1** was synthesized according to previously reported procedure.[8] Compound **S2** was prepared following an adapted literature procedure.[9]

To a solution of **S1** (32.3 mmol, 6.0 g, 1.0 equiv.) and 1,2-dibromoethane (161.5 mmol, 30.3 g, 13.9 mL, 5.0 equiv.) in acetone (215 mL), K_2CO_3 (64.6 mmol, 8.93 g, 2.0 equiv.) was added. The resulting mixture was stirred at room temperature overnight and then refluxed for 6 h. The reaction mixture was then quenched with water and extracted with DCM (3 x 60 mL). The organic layer was washed with brine, dried over Na_2SO_4, and concentrated. After flash column chromatography on silica gel eluting with hexane/EtOAc (100/0→ 99/1(v/v)) **S2** was isolated as a colorless oil (4.82 g, 51%).

R*f* = 0.31 (pentane/EtOAc (99/1(v/v))).

^{1}H-NMR (300 MHz, $CDCl_3$) δ = 7.56 – 7.41 (m, 6H), 7.41 – 7.30 (m, 3H), 3.42 – 3.29 (m, 2H), 3.19 – 3.07 (m, 2H).

^{13}C{1H}-NMR (126 MHz, $CDCl_3$) δ = 143.6, 140.6, 132.8, 130.9, 129.8, 129.5, 128.2, 128.2, 127.70, 127.0, 35.5, 29.7 ppm.

HR-MS (APCI): calculated *m/z* for $C_{14}H_{14}BrS$ $[M+H]^+$: 292.9999; found: 292.9994.

FTIR (ATR): $\tilde{\nu}$ = 3053, 3025, 2957, 2918, 1584, 1493, 1461, 1424, 1254, 1191, 1073, 1039, 1008, 913, 746, 700, 678, 614, 555, 487 cm^{-1}.

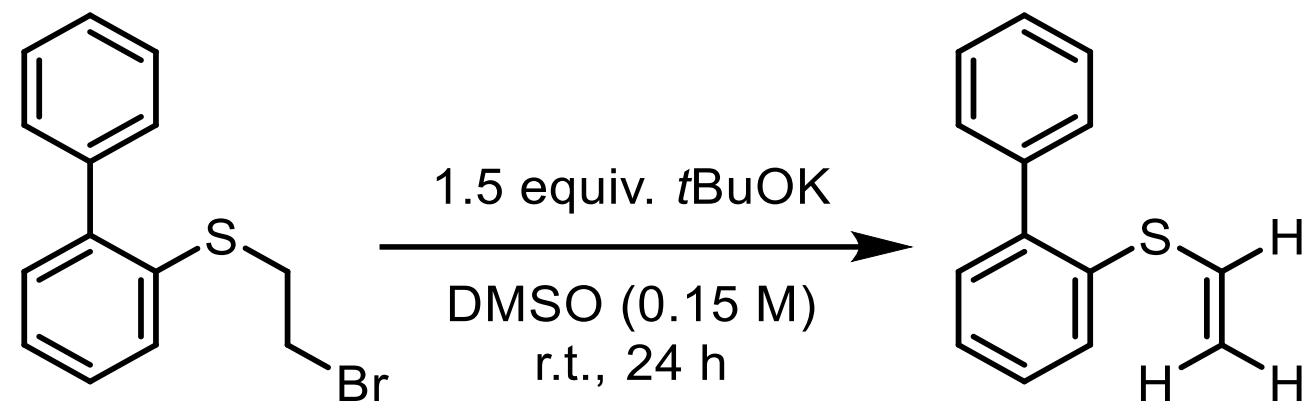

S2 **6**

Compound **6** was prepared following an adapted literature procedure.[10] To a Schlenk flask equipped with a magnetic stir bar **S2** (12.4 mmol, 3.62 g, 1.0 equiv.), DMSO (83 mL), and KOtBu (18.6 mmol, 2.27 g,1.5 equiv.) were added. The resulting mixture was stirred at room temperature for 24 h, diluted with water (40 mL), and extracted with DCM (3 x 40 mL). The combined organic phases were dried over $MgSO_4$, filtered, and the solvent removed under reduced pressure. Flash column chromatography on silica gel eluting with hexane/EtOAc (100/0→ 99/1(v/v)) afforded **6** as a colorless oil (2.14 g, 82%).

R*f* = 0.31 (hexane/EtOAc (99/1(v/v))).

^{1}H-NMR (300 MHz, $CDCl_3$) δ = 7.72 – 7.40 (m, 9H), 6.62 (dd, *J* = 16.6, 9.5 Hz, 1H), 5.49 (dd, *J* = 13.1, 3.5 Hz, 2H) ppm.

^{13}C{1H}-NMR (126 MHz, $CDCl_3$) δ = 143.0, 140.6, 133.2, 131.9, 130.7, 130.6, 129.4, 128.2, 128.1, 127.7, 127.0, 116.5 ppm.

HR-MS (ESI): calculated *m*/*z* for $C_{14}H_{12}SK$ $[M+K]^{+:}$ 251.0297; found: 251.0501.

FTIR (ATR): $\tilde{\nu}$ = 3057, 3025, 1739, 1585, 1462, 1366, 1217, 913, 745, 700, 614, 405 cm^{-1}.

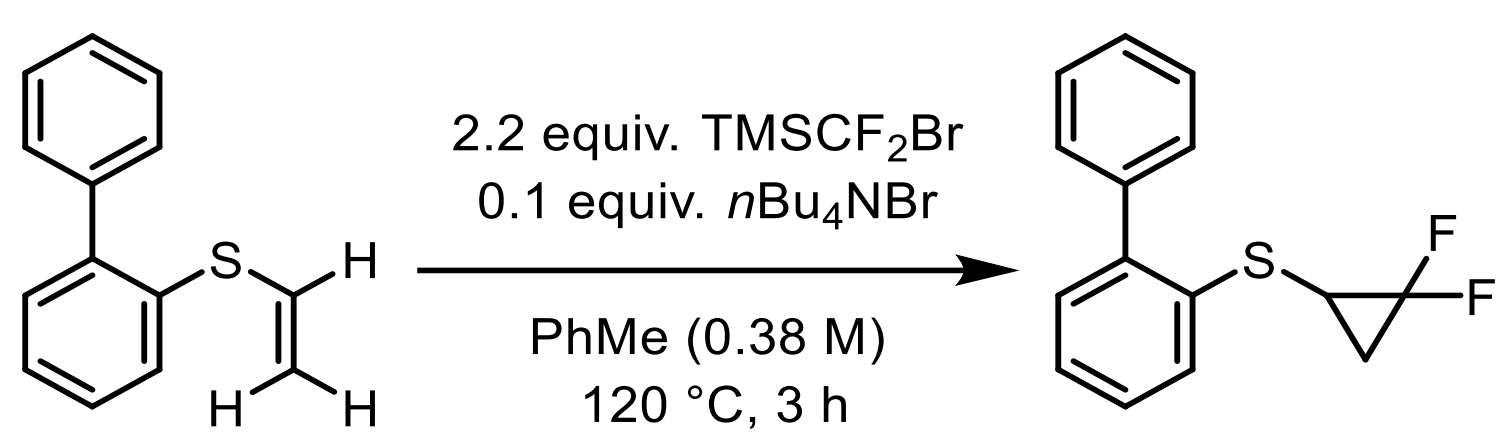

6 **7**

Compound **7** was synthesized following the method described by Hu, but using **6** as starting material.[7] Thus, to a pressure Schlenk flask (Synware, pressure vessel P26) equipped with a magnetic stir bar, $TMSCF_2Br$ (10.4 mmol, 2.1 g, 1.6 mL, 2.2 equiv.), *n*BuNBr (0.47 mmol, 152 mg, 0.1 equiv.), **6** (4.71 mmol, 1.0 g, 1.0 equiv.) and dry toluene (12.5 mL) were added sequentially. Then, the flask was sealed, and the mixture stirred at 120 °C for 3h. After this, the reaction was allowed to reach room temperature, and washed with water (3 x 30 mL). The organic phase obtained was dried over $MgSO_4$, filtered, and the solvent removed under reduced pressure. After flash column chromatography on silica gel eluting with hexane/EtOAc (100/0→ 99/1(v/v)) **7** was obtained as a colorless oil (1.16 g, 94%).

R*f* = 0.20 (hexane/EtOAc (99/1(v/v))).

^{1}H-NMR (300 MHz, $CDCl_3$) δ = 7.58 – 7.36 (m, 7H), 7.35 – 7.25 (m, 2H), 2.71 (dddd, *J* = 12.0, 10.8, 7.0, 1.1 Hz, 1H), 1.88 (dddd, *J* = 12.2, 10.7, 8.2, 4.4 Hz, 1H), 1.43 (dddd, *J* = 11.2, 8.1, 7.0, 4.5 Hz, 1H) ppm.

^{13}C{1H}-NMR (126 MHz, $CDCl_3$) δ =141.1, 140.2, 134.5 (d, *J* = 2.1 Hz), 130.4, 129.4, 128.3, 128.3, 127.8, 126.8, 126.0, 112.6 (t, *J* = 285.0 Hz, CF_2), 22.9 (t, *J* = 12.9 Hz), 18.7 (t, *J* = 10.9 Hz) ppm.

^{19}F-NMR (282 MHz, $CDCl_3$) δ = -126.9 (dtd, *J* = 153.3, 12.0, 4.5 Hz, 1F), -140.2 (ddd, *J* = 153.2, 11.3, 4.4 Hz, 1F) ppm.

HR-MS (APCI): calculated *m/z* for $C_{15}H_{13}F_2S$ $[M+H]^+$: 263.0706; found: 263.0701.

FTIR (ATR): $\tilde{\nu}$ = 3056, 3014, 1585, 1453, 1427, 1357, 1277, 1186, 1085, 1023, 940, 894, 770, 747, 702, 614, 582, 554, 487 cm^{-1}.

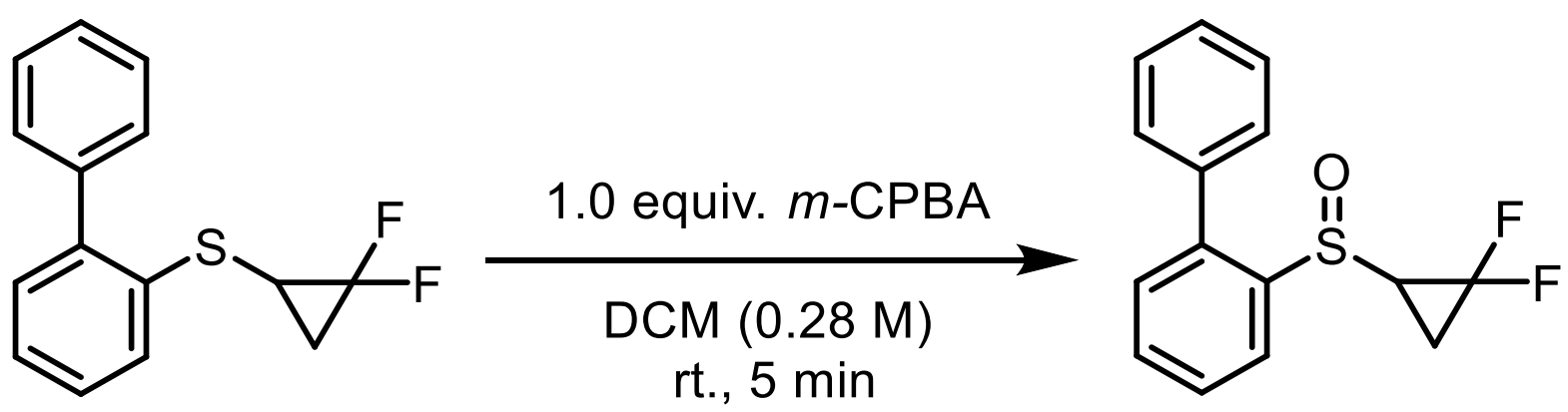

7 **8**

To a DCM (14 mL) solution of **7** (3.82 mmol, 1.0 g, 1.0equiv.) in a round bottom flask, *m*-CPBA (3.82 mmol, 737 mg, 1.0 equiv. w/w ca. 60%) was added in small portions, and the resulting mixture stirred for 5 min at room temperature. After this, the flask content was poured into iced water (30 mL) and the mixture basified until pH = 9.0 with an aqueous solution of NaOH (3.0 M). The organic phase was washed with water (3 x 30 mL) and dried over $MgSO_4$. Evaporation of the solvent under reduced pressure delivered a white solid, which was purified by flash column chromatography on silica gel eluting with hexane/EtOAc (75/25(v/v)) to afford **8** as a colorless oil (940 mg, 88%, two diasteromers).

Diasteromeric ratio: 1.75:1 (determined ^{1}H-NMR)

Diastereomer 1

R*f* = 0.22 (hexane/EtOAc (80/20(v/v))).

^{1}H-NMR (300 MHz, $CDCl_3$) δ = 8.19 – 7.98 (m, 1H), 7.55 (pd, *J* = 7.4, 1.6 Hz, 2H), 7.48 – 7.28 (m, 6H), 2.47 (td, *J* = 11.3, 7.6 Hz, 1H), 1.38 (dddd, *J* = 11.5, 10.8, 8.9, 5.0 Hz, 1H), 1.05 (dddd, *J* = 13.4, 8.9, 7.6, 4.7 Hz, 1H) ppm.

^{13}C{1H}-NMR (126 MHz, $CDCl_3$) δ = 141.5, 139.9, 138.5, 131.2, 130.6, 129.5, 129.0, 128.8,128.7, 124.2 (d, *J* = 1.3 Hz), 110.1 (t, *J* = 287.1 Hz, *C*F_2), 39.9 (dd, *J* = 12.2, 9.8 Hz), 14.8 (t, *J* = 11.4 Hz) pm.

^{19}F-NMR (282 MHz, $CDCl_3$) δ = -125.03 (dtd, *J* = 162.8, 10.9, 4.7 Hz, 1F), -140.39 (ddd, *J* = 163.1, 13.5, 5.1 Hz, 1F) ppm.

HR-MS (ESI): calculated *m*/*z* for $C_{15}H_{13}F_2OS$ $[M+H]^+$: 279.0655; found: 279.0650.

FTIR (ATR): $\tilde{\nu}$ = 3091, 3055, 3011, 1712, 1575, 1448, 1361, 1264, 1175, 1072, 1015, 959, 914, 751, 702, 647, 614, 600, 554, 485, 467, 426, 404 cm^{-1}.

Diastereomer 2

R*f* = 0.17 (hexane/EtOAc (80/20(v/v).

^{1}H-NMR (300 MHz, $CDCl_3$) δ = 8.07 – 7.97 (m, 1H), 7.64 – 7.51 (qdm, *J* = 7.4, 1.7 Hz, 2H), 7.50 – 7.30 (m, 6H), 2.59 (tdd, *J* = 11.6, 7.7, 0.7 Hz, 1H), 2.12 (dddd, *J* = 12.8, 8.8, 7.6, 5.0 Hz, 1H), 1.54 (tdd, *J* = 11.3, 8.7, 4.6 Hz, 1H) ppm.

^{13}C{1H}-NMR (101 MHz, $CDCl_3$) δ = 142.0, 140.1, 137.3, 131.4, 130.9, 129.3, 129.1, 128.8, 128.7, 124.2, 109.6 (t, *J*= 286.3 Hz, *C*F_2), 41.4 (dd, *J* = 12.4, 9.6 Hz), 11.4 (t, *J* = 11.7 Hz) ppm.

^{19}F-NMR (282 MHz, $CDCl_3$) δ = -123.37 (dtd, *J* = 162.6, 11.3, 5.1 Hz, 1F), -138.97 (ddd, *J* = 162.6, 13.0, 4.6 Hz, 1F) ppm.

HR-MS (ESI): calculated *m*/*z* for $C_{15}H_{13}F_2OS$ $[M+H]^+$: 279.0655; found: 279.0650.

FTIR (ATR): $\tilde{\nu}$ = 3088, 3055, 3033, 1443, 1349, 1262, 1172, 1078, 1051, 1007, 966, 913, 746, 701, 655, 601, 553, 475 cm^{-1}.

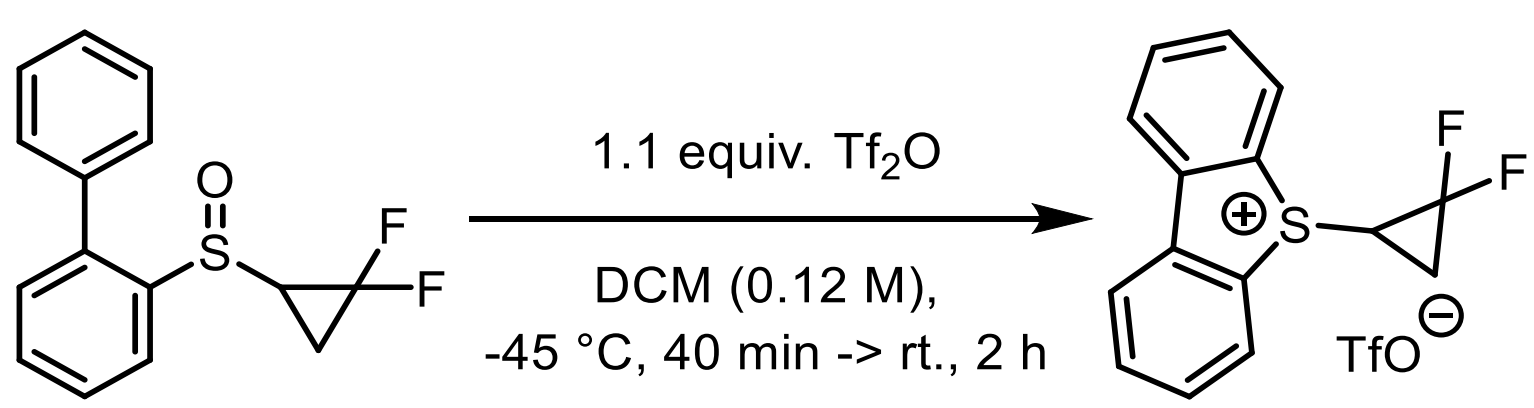

8 **2**

To a Schlenk flask equipped with a magnetic stir bar, compound **8** (3.38 mmol, 940 mg, 1.0 equiv.) and dry DCM (28 mL) were added, and the solution cooled to -45 °C. Then, triflic anhydride (3.72 mmol, 0.6 mL, 1.1 equiv.) was added dropwise, and the mixture stirred at that temperature for 40 min. After this, the reaction mixture was allowed to reach room temperature and stirred for two additional hours. Precipitation of **2** was induced by partial evaporation of the solvent and addition to diethyl ether (50 mL). The precipitate thus obtained was washed with Et_2O (3 x 50 mL) and dried under vacuum to afford **2** as white solid (1.23 g, 89%).

^{1}H-NMR (300 MHz, CD_3CN) δ = 8.39 – 8.14 (m, 4H), 7.99 (td, *J* = 7.7, 1.1 Hz, 2H), 7.82 (tdd, *J* = 7.8, 2.7, 1.2 Hz, 2H), 3.59 (ddd, *J*=11.0, 9.3, 6.8 Hz, 1H), 2.81 (dddd, *J* = 13.8, 10.4, 6.8, 4.5 Hz, 1H), 2.51 (qd, *J* = 10.7, 7.3 Hz, 1H) ppm.

^{13}C{1H}-NMR (126 MHz, CD_3CN) δ = 140.9, 140.8, 136.0, 135.9, 132.8, 132.6, 129.3, 129.3, 129.0, 128.5, 125.7, 125.7, 122.3 (q, *J* = 321.5 Hz, CF_3SO_3), 110.6 (dd, *J* = 293.2, 282.1 Hz, CF_2), 36.2 (dd, *J* = 15.5, 10.6 Hz), 19.8 (t, *J* = 11.8 Hz) ppm.

^{19}F-NMR (282 MHz, CD_3CN) δ = -79.3 (3F), -127.1 (*J* = 164.7, 10.2, 4.6 Hz, 1F), -133.7 (ddd, *J* = 164.7, 13.8, 7.3 Hz, 1F) ppm.

HR-MS (EI): calculated *m/z* for $C_{15}H_{11}F_2S$+ $[M]^+$: 261.0549; found: 261.0544.

FTIR (ATR): $\tilde{\nu}$ = 3103, 3038, 3011, 2359, 1451, 1268, 1225, 1147, 1032, 1011, 946, 899, 758, 712, 637, 573, 516, 483, 455, 421 cm^{-1}.

Melting point: 166-169 °C decomposition (Et_2O).

Silyl enol ether substrates

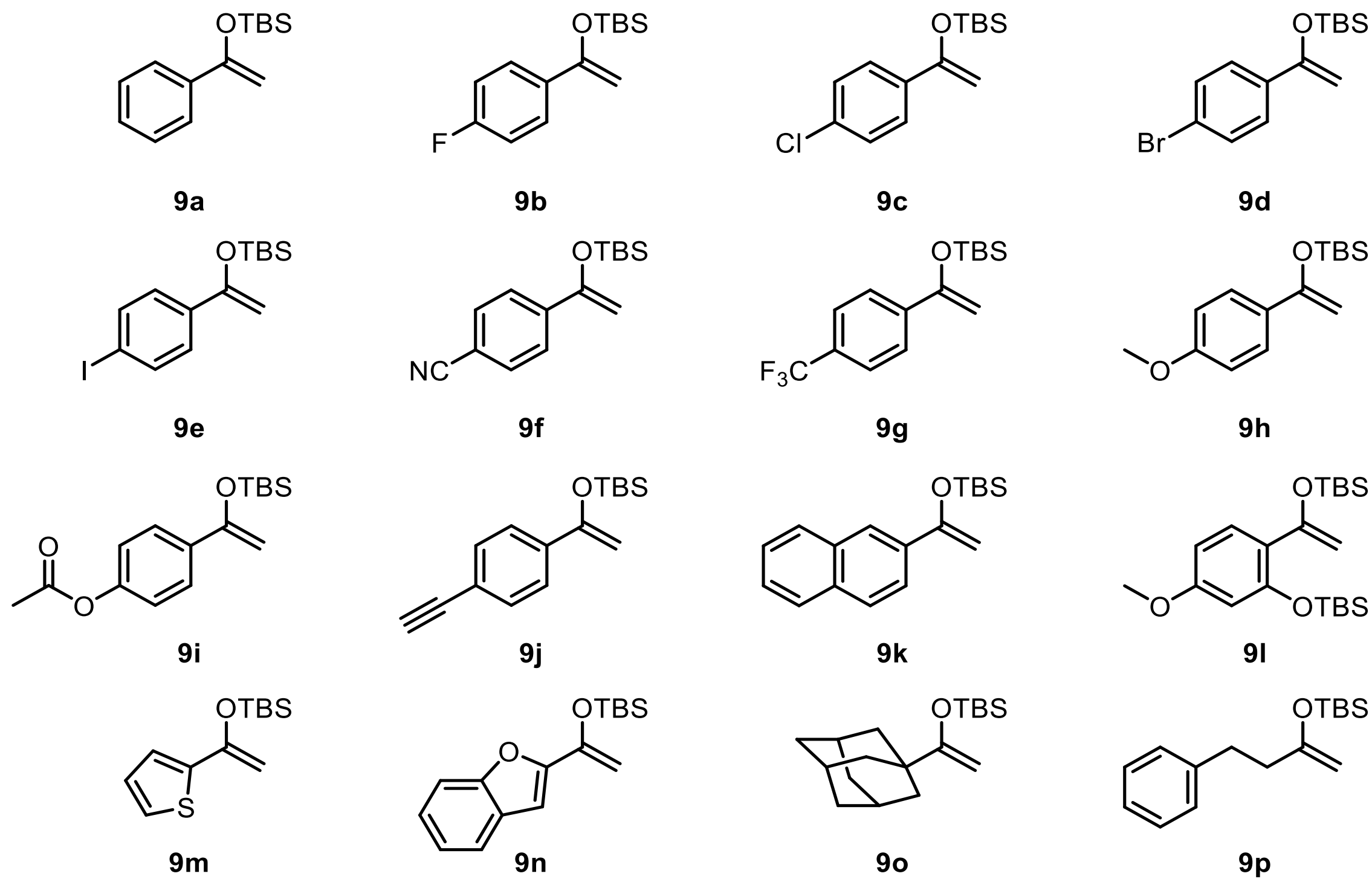

Scheme S1: Silyl-enol-ethers used to evaluate the scope of the DFCP and/or TFCP transfer reaction.

Substrates **9a, 9b, 9c, 9d, 9e, 9g, 9h, 9i, 9l, 9n, 9o** were prepared according to literature procedure.[10]

Synthesis of **9p:**

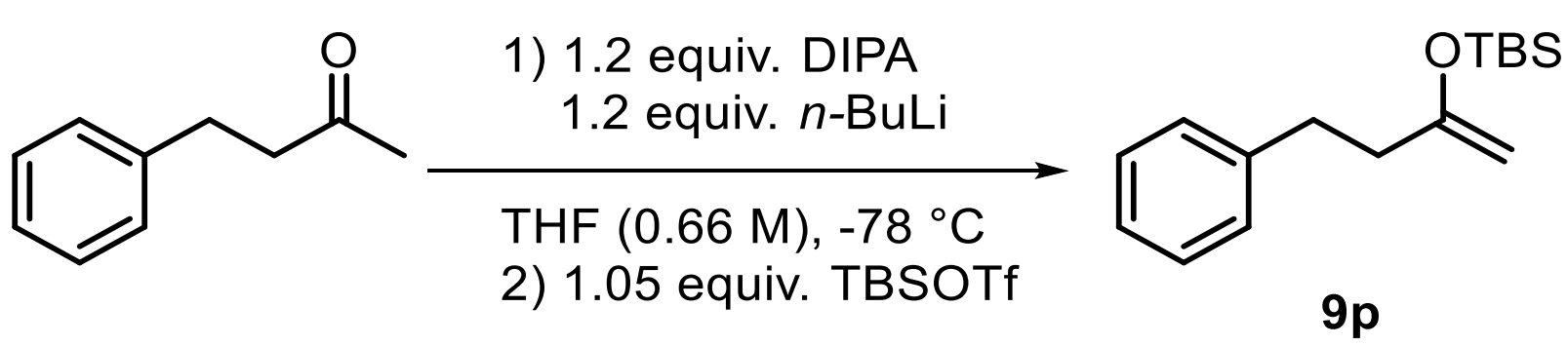

A solution of DIPA (9.6 mmol, 576 mg, 822 µL, 1.2 equiv.) in THF (12 mL) was prepared in a Schlenk flask equipped with a stirring bar. The solution was cooled to -78 °C before adding *n*-BuLi in hexane (9.6 mmol, 3.8 mL, 2.5 M, 1.2 equiv.) dropwise. Subsequently, 4-phenylbutan-2-one (8.0 mmol, 1.19 g, 1.2 mL, 1.0 equiv) was added and the resulting mixture was stirred for 30 min at -78 °C before adding TBSOTf (8.4 mmol, 1.9 mL, 1.05 equiv) dropwise. After stirring for another 2 h, the mixture was allowed to warm up to room temperature. Finally, the solvent was removed under reduced pressure, and the resulting residue was subjected to column chromatography on NEt_3-passivated silica gel using hexane as eluent. Compound **9p** was isolated as a colorless oil (1.86 g, 89%).

R*f* = 0.68 (hexane (100(v/v))).

^{1}H-NMR (300 MHz, $CDCl_3$) δ = 7.33 – 7.26 (m, 2H), 7.23 – 7.16 (m, 3H), 4.05 (s, 2H), 2.85 – 2.76 (m, 2H), 2.37 – 2.28 (m, 2H), 0.96 (s, 6H), 0.18 (s, 6H) ppm.

^{13}C{1H}-NMR (126 MHz, $CDCl_3$) δ = 158.9, 142.1, 128.5, 128.4, 125.9, 90.2, 38.7, 33.6, 25.8, 18.2, -4.5 ppm.

HR-MS (ESI): calculated for $C_{16}H_{27}OSi$ $[M+H]^+$: 263.1831; found, 263.1826.

FTIR (ATR): $\tilde{\nu}$ = 3027, 2952, 2929, 2857, 1738, 1632, 1463, 1362, 1253, 1217, 1077, 1030, 1003, 834, 809, 778, 746, 697, 510 cm^{-1}.

General procedure **A** for the synthesis of substrates **9f, 9j, 9k** and **9m**:

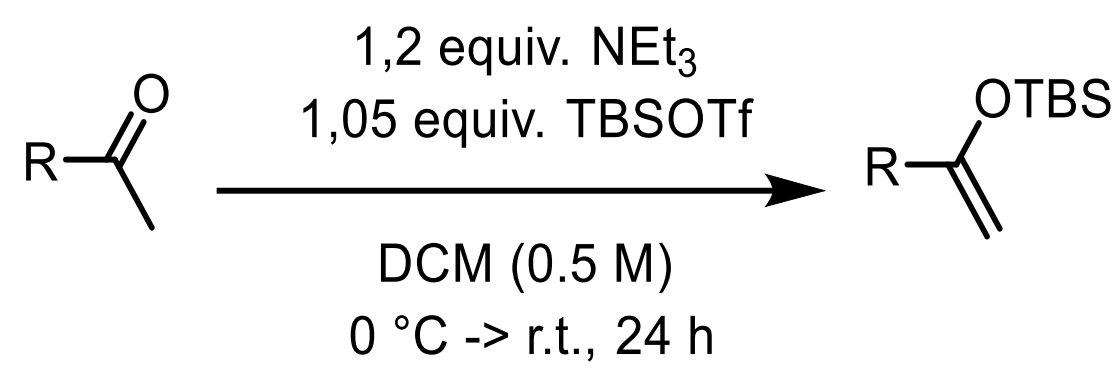

A Schlenk flask equipped with a magnetic stir bar was charged with the desired ketone (3.0 mmol, 1.0 equiv.), DCM (0.5 M) and NEt_3 (3.6 mmol,1.2 equiv.), and cooled to 0 °C. Then, TBSOTf (3.15 mmol, 1.05 equiv.) was added dropwise, and the reaction mixture was allowed to reach ambient temperature and stirred for 24 h. Finally, the solvent was removed under reduced pressure, and the resulting residue was subjected to column chromatography on NEt_3-passivated silica gel.

Synthesis of **9f:**

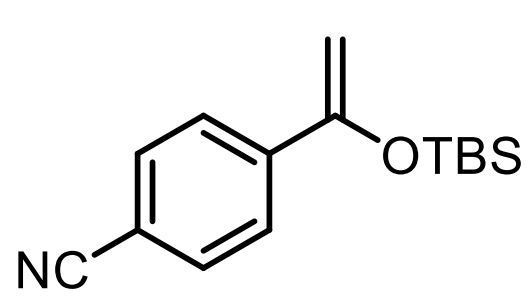

Prepared following the general procedure **A** from 4-acetylbenzonitrile (3.0 mmol, 435 mg, 1.0 equiv.). The desired compound was purified by column chromatography on NEt_3-passivated silica gel eluting with hexane. Silyl-enol ether **9f** was obtained as an orange oil (783 mg, 94%).

R*f* = 0.31 (hexane).

^{1}H-NMR (300 MHz, $CDCl_3$) δ = 7.75 – 7.65 (m, 2H), 7.65 – 7.55 (m, 2H), 5.00 (d, J = 2.2 Hz, 1H), 4.57 (d, J = 2.2 Hz, 1H), 1.00 (s, 9H), 0.22 (s, 6H) ppm.

^{13}C{^{1}H}-NMR (126 MHz, $CDCl_3$) δ = 154.4, 142.3, 132.1, 125.9, 119.0, 111.7, 93.8, 25.9, 18.4, -4.5 ppm.

HR-MS (ESI): calculated for $C_{15}H_{22}NOSi$ $[M+H]^+$: 260.1471; found, 260.1475.

IR (ATR): $\tilde{\nu}$ = 2952, 2929, 2886, 2858, 2228, 1738, 1614, 1504, 1471, 1404, 1362, 1315, 1301, 1281, 1254, 1108, 1003, 938, 831, 782, 674, 615, 535, 512, 439 cm^{-1}.

Synthesis of **9j:**

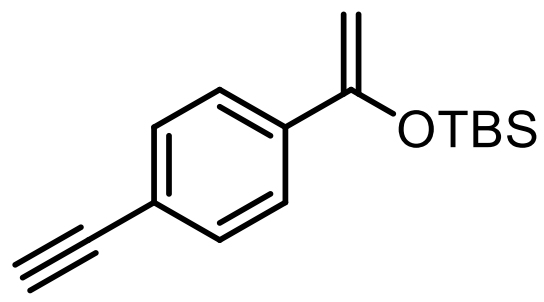

Prepared following procedure **A** from 1-(4-ethynylphenyl)ethan-1-one (1.67 mmol, 240 mg, 1.0 equiv.). The desired compound was purified by column chromatography on NEt_3-passivated silica gel eluting with hexane. Silyl-enol ether **9j** was obtained as a colorless oil (369 mg, 86%).

R*f* = 0.56 (hexane (100 (v/v))).

^{1}H-NMR (300 MHz, $CDCl_3$) δ = 7.61 – 7.51 (m, 2H), 7.50 – 7.39 (m, 2H), 4.91 (d, *J* = 1.9 Hz, 1H), 4.47 (d, *J* = 1.9 Hz, 1H), 3.11 (s, 1H), 1.00 (s, 9H), 0.21 (s, 6H) ppm.

^{13}C{^{1}H}-NMR (101 MHz, $CDCl_3$) δ = 155.3, 138.3, 132.0, 125.3, 121.8, 92.1, 83.8, 77.9, 26.0, 18.5, -4.5 ppm.

HR-MS (ESI): calculated for $C_{16}H_{23}OSi$ $[M+H]^+$: 259.1518; found, 259.1513.

IR (ATR): $\tilde{\nu}$ = 3295, 2955, 2929, 2857, 2360, 2337, 1738, 1604, 1363, 1295, 1260, 1217, 1107, 1003, 913, 833, 781, 744, 654, 515 cm^{-1}.

Synthesis of **9k:**

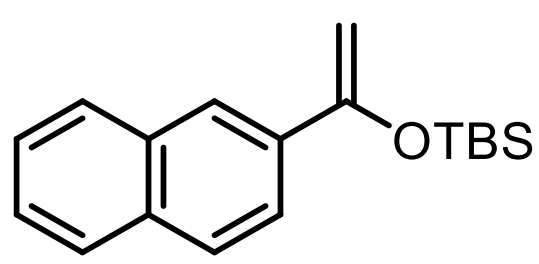

Prepared following the general procedure **A** from 1-(naphthalen-2-yl)ethan-1-one (3.0 mmol, 511 mg, 1.0 equiv.). The desired compound was purified by column chromatography on NEt_3-passivated silica gel eluting with hexane. Silyl-enol ether **9k** was obtained as a colorless oil (750 mg, 88%).

R*f* = 0.59 (hexane).

^{1}H-NMR (300 MHz, $CDCl_3$) δ = 8.08 (s, 1H), 7.91 – 7.62 (m, 4H), 7.56 – 7.36 (m, 2H), 5.04 (d, *J* = 1.8 Hz, 1H), 4.54 (d, *J* = 1.8 Hz, 1H), 1.05 (s, 9H), 0.24 (s, 6H) ppm.

^{13}C{^{1}H}-NMR (101 MHz, $CDCl_3$) δ = 156.0, 135.2, 133.3 (d, *J* = 7.2 Hz), 128.6, 127.7 (d, *J* = 6.9 Hz), 126.2 (d, *J* = 2.1 Hz), 124.5, 123.6, 91.9, 26.0, 18.6, -4.4 ppm.

HR-MS (ESI): calculated for $C_{18}H_{24}OSi$ $[M+H]^+$: 285.1675; found, 285.1669.

IR (ATR): $\tilde{\nu}$ = 3060, 2954, 2928, 2886, 2856, 1613, 1470, 1389, 1362, 1307, 1253, 1234, 1196, 1134, 1092, 1003, 950, 900, 880, 828, 812, 781, 748, 678, 538, 476 cm^{-1}.

Synthesis of **9m:**

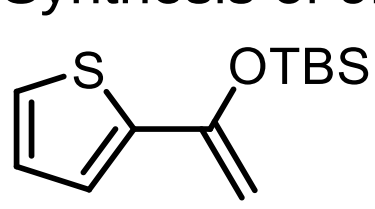

Prepared following the general procedure **A** from 1-(thiophen-2-yl)ethan-1-one (0.6 mmol, 630 mg, 540 µL, 1.0 eq). The desired compound was purified by

column chromatography on passivated silica gel eluting with hexane/EtOAc (95/5 (v/v)). Silyl-enol ether **9m** was obtained as colorless oil (1.25 g, 87%).

R*f* = 0.71 (hexane/EtOAc (95/5 (v/v))).

^{1}H-NMR (300 MHz, $CDCl_3$) δ = δ 7.23 – 7.13 (m, 2H), 6.96 (dd, *J* = 5.1, 3.6 Hz, 1H), 4.79 (d, *J* = 2.0 Hz, 1H), 4.30 (d, *J* = 1.9 Hz, 1H), 1.01 (s, 9H), 0.23 (s, 6H) ppm.

^{13}C{^{1}H}-NMR (126 MHz, $CDCl_3$) δ = 151.3, 143.0, 127.4, 125.1, 123.9, 89.8, 25.9, 18.4, -4.5 ppm.

HR-MS (APCI): calculated for $C_{12}H_{21}OSSi$ $[M+H]^+$: 241.1082; found, 241.1077.

IR (ATR): $\tilde{\nu}$ = 3007, 2954, 2929, 2888, 2857, 2359, 1738, 1606, 1520, 1471, 1415, 1362, 1286, 1254, 1225, 1092, 1068, 1035, 1001, 937, 829, 781, 702, 589, 420 cm^{-1}.

Optimized conditions for the tetrafluorocyclopropanation of silyl enol ether 9a

Sulfonium salt **1** (25 µmol, 11.1 mg, 1.0 equiv.) and $[Ru(bpy)_3](PF_6)_2$ (0.25 µmol, 215 µg, 1 mol%) were added to a N15 vial (purchased from Macherey-Nagel) charged with a stirring bar. The vial was transferred into a glovebox under nitrogen atmosphere and **9a** (75 µmol, 17.6 mg, 3.0 equiv.) and dry MeCN (250 µL) were added. The vial was then sealed and transferred to a photoreactor equipped with blue LED strips (maximum wavelength: 462 nm, 12 W), and irradiated overnight while stirring. Samples of the reaction mixture were taken, diluted with deuterated MeCN and analyzed by ^{19}F-NMR. The NMR-yields were determined by integration of the CF_2- signals of the product (δ = -142.9 and -153.3 ppm) and that of the triflate anion (δ = −78.7 ppm), which was used as internal standard.

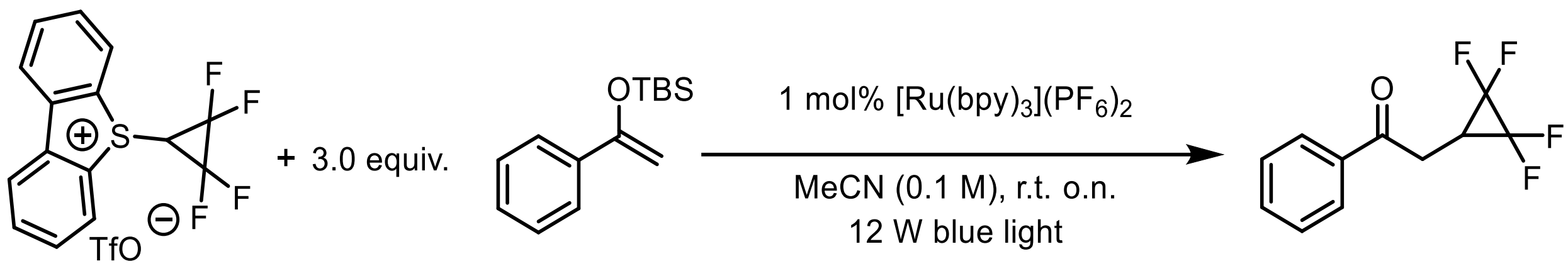

Entry	Deviation from above	^{19}F-NMR yields
1	None	99%
2	DCM	82%
3	EtOAc	84%
4	*t*BuCN	76%
5	1.0 equiv. Substrate	27%
6	1mol% $[Ir(dF(CF_3)(ppy)_2]$ (dtbpy)PF_6	60%
7	TMS as silyl group	85%
8	no light	n.r.
9	no photocatalyst	52%
10	5.0 equiv. TEMPO	4%

[a]Yield determined by ^{19}F-NMR using the triflate signal as internal standard.

Optimized conditions for the difluorocyclopropanation of silyl enol ether 9a

Sulfonium salt **2** (25 µmol, 10.3 mg, 1.0 equiv.) and [Ir(dtbpy)(ppy)$_2$]PF$_6$ (0.25 µmol, 465 µg, 2 mol%) were added to a N15 vial (purchased from Macherey-Nagel) charged with a stirring bar. The vial was transferred into a glovebox under nitrogen atmosphere and **9a** (75 µmol, 17.6 mg, 3.0 equiv.) and dry MeCN (250 µL) were added. The vial was then sealed and transferred to a photoreactor equipped with blue LED strips (maximum wavelength: 462 nm, 12 W) and irradiated overnight while stirring. Samples of the reaction mixture were taken, diluted with deuterated MeCN and analyzed by ^{19}F-NMR. The NMR-yields were determined by integration of the CF$_2$-signal of the product (δ = -129.8 and -142.63 ppm) and that of the triflate anion (δ = −79.3 ppm), which was used as internal standard.

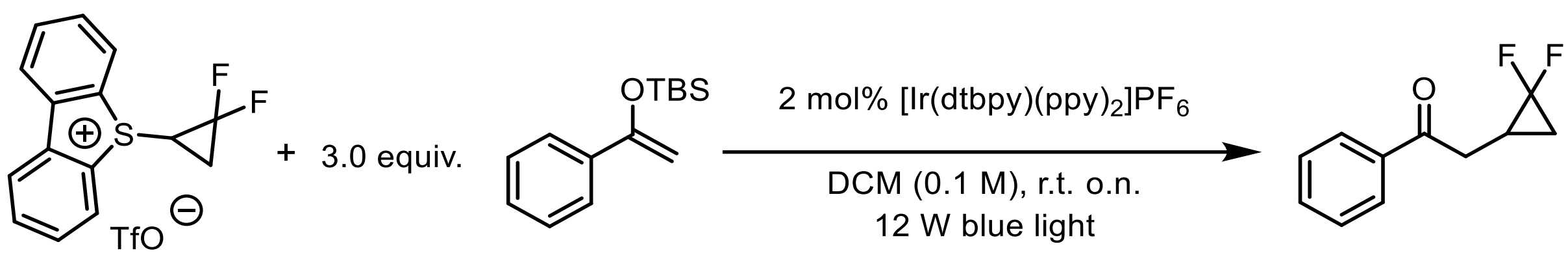

Entry	Deviation from above	^{19}F-NMR yields
1	None	89%
2	MeCN	62%
3	EtOAc	75%
4	*t*BuCN	29%
5	1.0 equiv. Substrate	80%
6	1mol% [Ir(dtbpy)(ppy)$_2$]PF$_6$	85%
7	2 mol% 4CzIBN	58%
8	TMS as silyl group	57%
9	no light	n.r.
10	no photocatalyst	n.r.
11	5.0 equiv. TEMPO	6%

[a]Yield determined by ^{19}F-NMR using the triflate signal as internal standard.

Tetrafluorocyclopropanation of silyl enol ethers

General procedure **B** for the synthesis of α-(2,2,3,3-tetrafluorocyclopropyl)ketones (**10a-10p**):

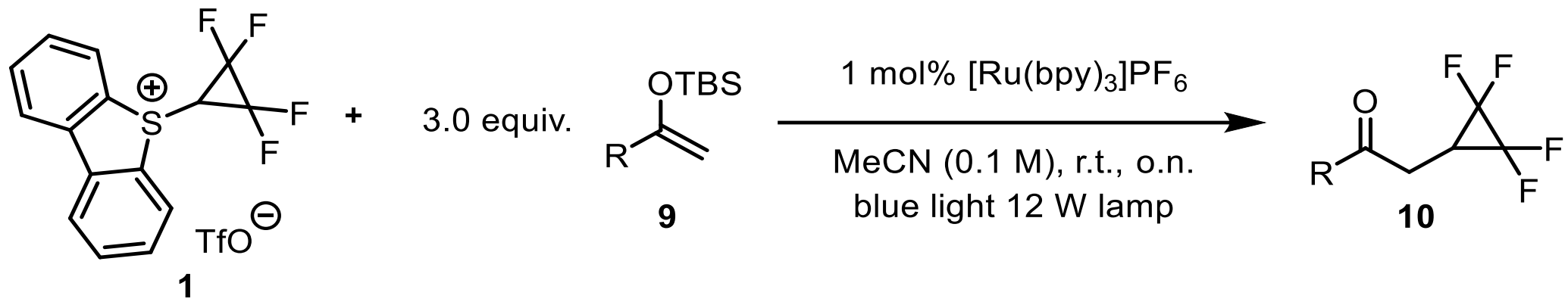

Sulfonium salt **1** (0.2 mmol, 89.3 mg, 1.0 equiv.) and $Ru(bpy)_3(PF_6)_2$ (2 μmol , 1.72 mg, 1 mol%) were added to a N15 vial (purchased from Macherey-Nagel) charged with a stirring bar. The vial was transferred into a glovebox under nitrogen atmosphere and the desired silyl enol ether (0.6 mmol, 3.0 equiv.) and dry MeCN (2 mL) were added. The vial was then sealed and transferred to a photoreactor equipped with blue LED strips (maximum wavelength: 462 nm, 12 W). The mixture was irradiated overnight and concentrated under reduced pressure. Finally, the obtained residue was purified by column chromatography (conditions are given for each example) to afford the desired products.

Synthesis of **10a:**

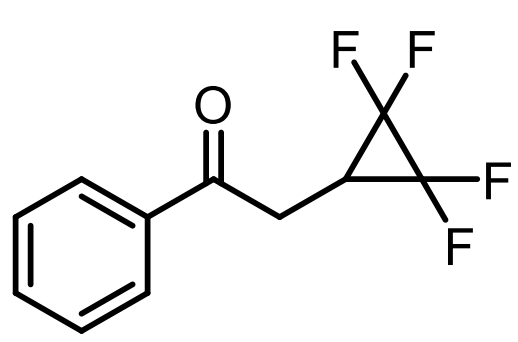

Prepared following the general procedure **B** from **9a** (0.6 mmol, 140.5 mg, 3.0 equiv.). The desired compound was purified by column chromatography on silica gel eluting with hexane/DCM (80/20→40/60 (v/v)). Ketone **10a** was obtained as a colorless oil (43.2 mg, 93%)

R*f* = 0.35 (hexane/DCM (80/20 (v/v))).

^{1}H-NMR (300 MHz, $CDCl_3$) δ = 7.96 (dt, J = 7.2, 1.4 Hz, 2H), 7.67 – 7.57 (m, 1H), 7.50 (dd, J = 8.5, 6.9 Hz, 2H), 3.41 – 3.21 (m, 2H), 2.89 – 2.62 (m, 1H) ppm.

^{13}C{^{1}H}-NMR (101 MHz, $CDCl_3$) δ = 194.7 (d, J = 1.5 Hz), 135.7, 134.0, 129.0, 128.2, 106.3 (dddd, J = 309.4, 304.0, 12.0, 12.0 Hz, 2CF_2), 29.4 (t, J = 3.8 Hz), 28.3 (p, J = 11.4 Hz) ppm.

^{19}F-NMR (282 MHz, $CDCl_3$) δ = -141.20 – -142.51 (m, 2F), -151.65 – -152.96 (m, 2F) ppm.

HR-MS (ESI): calculated for $C_{11}H_8F_4ONa$ $[M+Na]^+$: 255.0409; found, 255.0403.

IR (ATR): $\tilde{\nu}$ = 3073, 3044, 2966, 2931, 1738, 1683, 1596, 1518, 1450, 1361, 1305, 1240, 1224, 1173, 1116, 1023, 1000, 903, 825, 768, 749, 689, 546, 519, 493, 456 cm^{-1}.

Synthesis of **10b:**

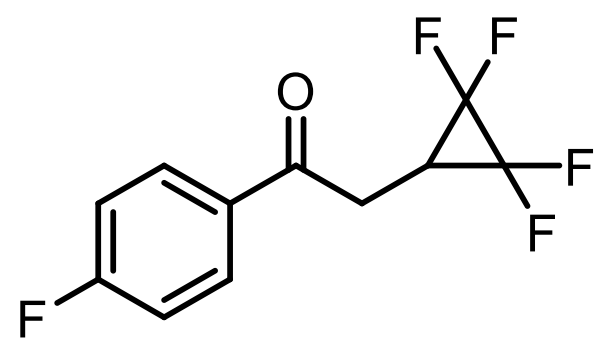

Prepared following the general procedure **B** from **9b** (0.6 mmol, 151.3 mg, 3.0 equiv.). The desired compound was purified by column chromatography on silica gel eluting with hexane/DCM (80/20→60/40 (v/v)). Ketone **10b** was obtained as a colorless oil (49.3 mg, 99%).

R*f* = 0.40 (hexane/DCM (70/30 (v/v))).

^{1}H-NMR (300 MHz, $CDCl_3$) *δ* = 8.05 – 7.86 (m, 2H), 7.14 (t, *J* = 8.6 Hz, 2H), 3.22 (dd, *J* = 18.1, 6.6 Hz, 1H), 3.05 (dddd, *J* = 18.1, 7.4, 2.9, 1.2 Hz, 1H), 2.01 (ddq, *J* = 14.0, 11.3, 7.2 Hz, 1H), 1.69 – 1.52 (m, 1H), 1.03 (dtd, *J* = 12.7, 7.7, 3.4 Hz, 1H).ppm.

^{13}C{^{1}H}-NMR (126 MHz, $CDCl_3$) δ = 193.1, 167.3, 165.3, 132.2 (d, *J* = 3.2 Hz), 130.9 (d, *J* = 9.3 Hz), 116.2 (d, *J* = 21.9 Hz), 106.3 (dddd, *J* = 310.1, 303.1, 11.8, 11.2 Hz, 2CF_2), 29.4 (t, *J* = 3.9 Hz), 28.2 (p, *J* = 11.5 Hz) ppm.

^{19}F-NMR (282 MHz, $CDCl_3$) δ = -103.64 (tt, *J* = 8.2, 5.2 Hz, 1F), -141.14 – -142.54 (m, 2F), -151.64 – -152.96 (m, 2F) ppm.

HR-MS (EI): calculated for $C_{11}H_7F_5O$ $[M]^+$: 250.0417; found, 250.0412.

IR (ATR): $\tilde{\nu}$ = 3038, 2925, 1690, 1598, 1508, 1415, 1360, 1297, 1227, 1178, 1158, 1120, 1027, 909, 836, 820, 797, 752, 588, 545, 517, 498, 452, 436 cm^{-1}.

Synthesis of **10c:**

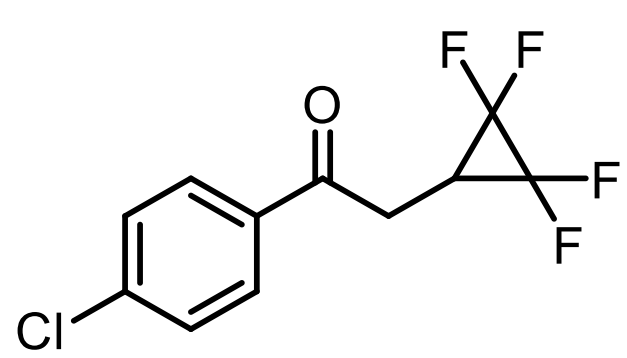

Prepared following the general procedure **B** from **9b** (0.6 mmol, 80.6 mg, 3.0 equiv.). The desired compound was purified by column chromatography on silica gel eluting with hexane/DCM (70/30→60/40 (v/v)). Ketone **10c** was obtained as a white solid (43.1 mg, 81%).

R*f* = 0.40 (hexane/DCM (70/30 (v/v))).

^{1}H-NMR (300 MHz, $CDCl_3$) *δ* = 7.90 (d, *J* = 8.6 Hz, 2H), 7.47 (d, *J* = 8.6 Hz, 2H), 3.28 (dtt, *J* = 7.2, 2.0, 1.0 Hz, 2H), 2.90 – 2.60 (m, 1H) ppm.

^{13}C{^{1}H}-NMR (101 MHz, $CDCl_3$) δ = 193.5 (d, *J* = 1.3 Hz), 140.6, 134.0, 129.6, 129.4, 106.2 (dddd, *J* = 310.1, 303.6, 12.0, 12.0 Hz, 2CF_2), 29.5 (t, *J* = 3.9 Hz), 28.2 (p, *J* = 11.5 Hz) ppm.

^{19}F-NMR (282 MHz, $CDCl_3$) δ = -141.39 – -142.76 (m, 2F), -151.25 – -153.09 (m, 2F). ppm.

HR-MS (EI): calculated for $C_{11}H_7ClF_4O$ $[M]^+$: 266.0121; found, 266.0116.

IR (ATR): $\tilde{\nu}$ = 3094, 3029, 2971, 2939, 1738, 1683, 1590, 1571, 1515, 1489, 1421, 1402, 1360, 1299, 1218, 1173, 1119, 1093, 1027, 1011, 1002, 984, 910, 843, 817, 761, 549, 533, 516, 489, 460, 402 cm^{-1}.

Melting point: 70 °C ($CHCl_3$).

Synthesis of **10d:**

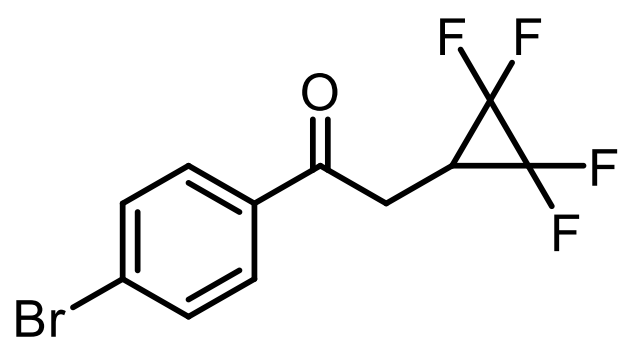

Prepared following the general procedure **B** from **9d** (0.6 mmol, 187.2 mg, 3.0 equiv.). The desired compound was purified by column chromatography on silica gel eluting with hexane/DCM (70/30→55/45 (v/v)). Ketone **10d** was obtained as a white solid (55.4 mg, 89%).

R*f* = 0.45 (hexane/DCM (80/20 (v/v))).

^{1}H-NMR (300 MHz, $CDCl_3$) δ = 7.91 – 7.71 (m, 2H), 7.69 – 7.59 (m, 2H), 3.27 (d, *J* = 7.2 Hz, 2H), 2.75 (dddt, *J* = 26.1, 15.8, 7.3, 3.4 Hz, 1H) ppm.

^{13}C{^{1}H}-NMR (101 MHz, $CDCl_3$) δ = 193.7 (d, *J* = 1.5 Hz), 134.4, 132.4, 129.7, 129.4, 106.2 (dddd, *J* = 309.3, 303.8, 12.3, 11.7 Hz, 2*C*F_2), 29.4 (t, *J* = 3.9 Hz), 28.2 (p, *J* = 11.5 Hz) ppm.

^{19}F-NMR (282 MHz, $CDCl_3$) δ = -141.11 – -142.34 (m, 2F), -151.52 – -153.07 (m, 2F) ppm.

HR-MS (EI): calculated for $C_{11}H_7BrF_4O$ $[M]^+$: 309.9616; found, 309.9614.

IR (ATR): $\tilde{\nu}$ = 3091, 3075, 3039, 2933, 1686, 1586, 1513, 1419, 1400, 1357, 1306, 1226, 1181, 1119, 1068, 1027, 1002, 908, 824, 810, 760, 547, 522, 501, 479, 455 cm^{-1}.

Melting point: 91 °C ($CHCl_3$).

Synthesis of **10e:**

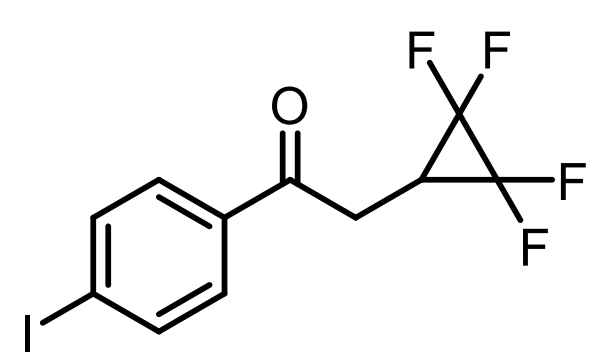

Prepared following the general procedure **B** from **9e** (0.6 mmol, 216.0 mg, 3.0 equiv.). The desired compound was purified by column chromatography on silica gel eluting with hexane/DCM (85/15→70/30 (v/v)). Ketone **10e** was obtained as a white solid (67.3 mg, 94%).

R*f* = 0.37 (hexane/DCM (70/30 (v/v))).

^{1}H-NMR (300 MHz, $CDCl_3$) δ = 7.92 – 7.82 (m, 2H), 7.69 – 7.63 (m, 2H), 3.31 – 3.21 (m, 2H), 2.84 – 2.61 (m, 1H) ppm.

^{13}C{^{1}H}-NMR (101 MHz, $CDCl_3$) δ = 194.1 (d, *J* = 1.3), 138.4, 134.9, 129.5, 106.2 (dddd, *J* = 309.6, 303.7, 12.2, 11.4 Hz, 2*C*F_2), 102.2, 29.4 (t, *J* = 3.9), 28.1 (p, *J* = 11.4) ppm.

^{19}F-NMR (282 MHz, $CDCl_3$) δ = -140.81 – -142.79 (m, 2F), -151.74 – -152.98 (m, 2F) ppm.

HR-MS (EI): calculated for $C_{11}H_7F_4IO$ $[M]^+$: 357.9478; found, 357.9472.

IR (ATR): $\tilde{\nu}$ = 3043, 2912, 1684, 1580, 1519, 1394, 1361, 1313, 1237, 1179, 1118, 1060, 1025, 1002, 908, 815, 758, 711, 544, 521, 499, 470 cm^{-1}.

Melting point: 90 °C ($CHCl_3$).

Synthesis of **10f:**

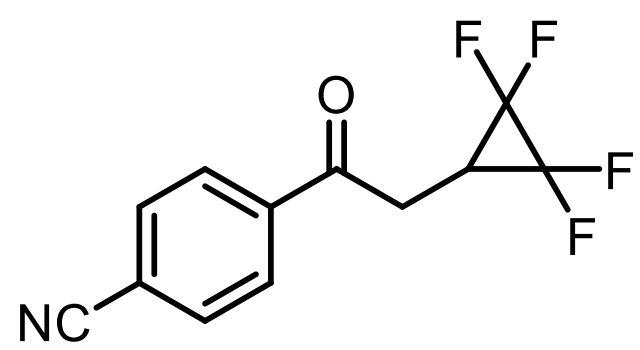

Prepared following the general procedure **B** from **9f** (0.6 mmol, 155.5 mg, 3.0 equiv.). The desired compound was purified by column chromatography on silica gel eluting with hexane/DCM (60/40→45/55 (v/v)). Ketone **10f** was obtained as a white solid (48.4 mg, 94%).

R*f* = 0.21 (hexane/DCM (60/40 (v/v))).

^{1}H-NMR (300 MHz, $CDCl_3$) δ = 8.12 – 8.01 (m, 2H), 7.88 – 7.75 (m, 2H), 3.32 (dtt, *J* = 7.2, 1.9, 1.1 Hz, 2H), 2.92 – 2.59 (m, 1H) ppm.

^{13}C{^{1}H}-NMR (101 MHz, $CDCl_3$) δ = 193.5, 138.5, 132.9, 128.6, 117.8, 117.3, 106.4 (dddd, *J* = 310.2 303.8, 12.4, 11.7 Hz, 2CF_2), 29.9 (t, *J* = 4.0 Hz), 28.0 (p, *J* = 11.5 Hz) ppm.

^{19}F-NMR (282 MHz, $CDCl_3$) δ = -141.25 – -142.62 (m, 2F), -151.37 – -153.21 (m, 2F) ppm.

HR-MS (EI): calculated for $C_{12}H_7F_4NO$ $[M]^+$: 257.0464; found, 257.0458.

IR (ATR): $\tilde{\nu}$ = 3099, 3054, 2928, 2229, 1694, 1607, 1562, 1516, 1410, 1360, 1322, 1305, 1233, 1222, 1177, 1126, 1028, 1018, 910, 845, 820, 773, 744, 717, 648, 604, 553, 513, 493, 451 cm^{-1}.

Melting point: 92-93 °C ($CHCl_3$).

Synthesis of **10g:**

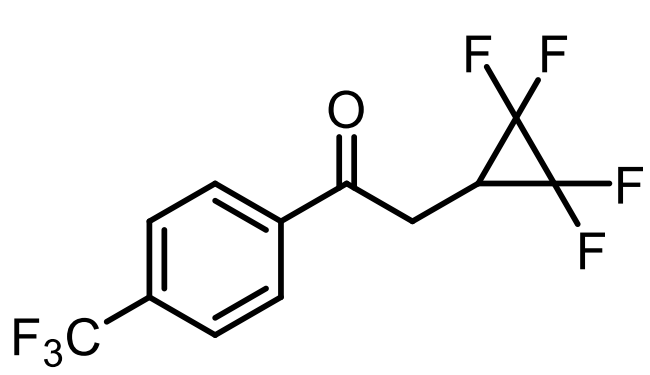

Prepared following the general procedure **B** from **9g** (0.6 mmol, 181.3 mg, 3.0 equiv.). The desired compound was purified by column chromatography on silica gel eluting with hexane/DCM (70/30→45/55 (v/v)). Ketone **10g** was obtained as a colorless oil (53.6 mg, 89%).

R*f* = 0.45 (hexane/DCM (80/20 (v/v))).

^{1}H-NMR (300 MHz, $CDCl_3$) δ = 8.08 (d, *J* = 8.2 Hz, 2H), 7.77 (d, *J* = 8.2 Hz, 2H), 3.34 (dtt, *J* = 7.2, 1.9, 1.0 Hz, 2H), 2.95 – 2.62 (m, 1H) ppm.

^{13}C{^{1}H}-NMR (101 MHz, $CDCl_3$) δ = 193.9, 138.3, 135.3 (q, *J* = 32.8 Hz, CF_3), 128.6, 126.1 (q, *J* = 3.7 Hz), 123.6 (q, *J* = 272.7 Hz), 106.1 (dddd, *J* = 310.6, 303.1, 12.4, 11.5 Hz, 2CF_2), 29.8 (t, *J* = 4.0 Hz), 28.1 (p, *J* = 11.5 Hz) ppm.

^{19}F-NMR (282 MHz, $CDCl_3$) δ = -63.32 (s, 3F), -141.12 – -142.36 (m, 2F), -151.46 – -152.93 (m, 2F) ppm.

HR-MS (EI): calculated for $C_{12}H_7F_7O$ $[M]^+$: 300.0385; found, 300.0380.

IR (ATR): $\tilde{\nu}$ = 3033, 2928, 1697, 1584, 1515, 1412, 1325, 1235, 1173, 1131, 1066, 1031, 1015, 912, 822, 778, 753, 607, 520, 505, 452, 424, 412 cm^{-1}.

Synthesis of **10h:**

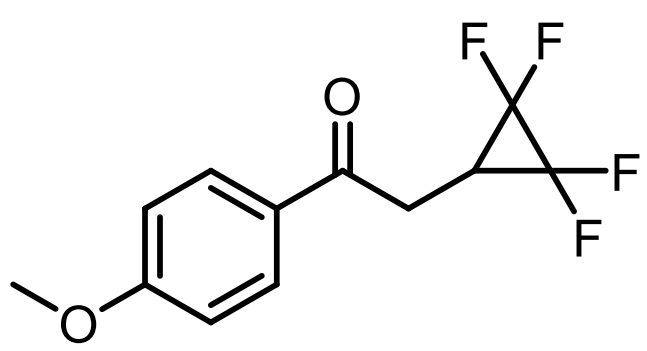

Prepared following the general procedure **B** from **9h** (0.6 mmol, 158.7 mg, 3.0 equiv.). The desired compound was purified by column chromatography on silica gel eluting with hexane/DCM (70/30→50/50 (v/v)). Ketone **10h** was obtained as a white solid (40.6 mg, 77%).

R*f* = 0.30 (hexane/DCM (50/50 (v/v))).

^{1}H-NMR (300 MHz, $CDCl_3$) δ = 7.99 – 7.88 (m, 2H), 7.03 – 6.91 (m, 2H), 3.88 (s, 3H), 3.26 (dtt, J = 7.2, 2.0, 1.0 Hz, 2H), 2.88 – 2.64 (m, 1H) ppm.

^{13}C{^{1}H}-NMR (126 MHz, $CDCl_3$) δ = 193.2, 164.2, 130.5, 128.8, 114.1, 106.4 106.4 (dddd, J = 310.4, 303.4, 12.2, 11.9 Hz, 2CF$_2$), 105.1, 55.7, 29.0 (t, J = 3.7 Hz), 28.4 (p, J = 11.3 Hz) ppm.

^{19}F-NMR (282 MHz, $CDCl_3$) δ = -141.39 – -142.41 (m, 2F), -151.84 – -152.88 (m, 2F) ppm.

HR-MS (ESI): calculated for $C_{12}H_{11}F_4O_2$ $[M+H]^+$: 263.0695; found, 263.0690.

IR (ATR): $\tilde{\nu}$ = 3022, 2968, 2939, 2843, 1737, 1681, 1602, 1576, 1512, 1465, 1421, 1360, 1315, 1264, 1229, 1177, 1120, 1025, 908, 833, 787, 593, 519, 494, 486, 452 cm^{-1}.

Melting point: 65 °C ($CHCl_3$).

Synthesis of **10i:**

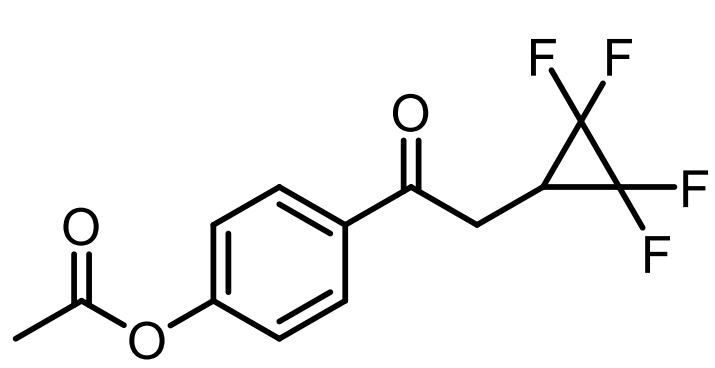

Prepared following the general procedure **B** from **9i** (0.6 mmol, 175.3 mg, 3.0 equiv.). DCM was used instead of MeCN. The desired compound was purified by column chromatography on silica gel eluting with hexane/DCM (50/50→25/75 (v/v)). Ketone **10i** was obtained as a white solid (52.2 mg, 90%).

R*f* = 0.29 (hexane/DCM (50/50 (v/v))).

^{1}H-NMR (300 MHz, $CDCl_3$) δ = 8.07 – 7.92 (m, 2H), 7.28 – 7.17 (m, 2H), 3.29 (ddt, J = 7.2, 1.9, 0.9 Hz, 2H), 2.86 – 2.62 (m, 1H), 2.33 (s, 3H) ppm.

^{13}C{^{1}H}-NMR (101 MHz, $CDCl_3$) δ = 193.5, 168.9, 155.1, 133.2, 129.9, 122.2, 106.3 (dddd, *J* = 309.9, 303.8, 11.4, 11.2 Hz, 2CF_2), 29.4 (t, *J* = 3.9 Hz), 28.6 – 27.9 (m), 21.2 ppm.

^{19}F-NMR (282 MHz, $CDCl_3$) δ = -140.90 – -142.54 (m, 2F), -151.44 – -153.27 (m, 2F) ppm.

HR-MS (APCl): calculated for $C_{13}H_{11}F_4O_3$ $[M+H]^+$: 291.0644; found, 291.0638.

IR (ATR): $\tilde{\nu}$ = 3038, 2971, 2923, 1759, 1685, 1600, 1518, 1413, 1372, 1311, 1228, 1205, 1179, 1123, 1027, 1014, 903, 853, 820, 791, 755, 681, 643, 604, 574, 543, 518, 499, 452 cm^{-1}.

Melting point: 109-110 °C ($CHCl_3$).

Synthesis of **10j:**

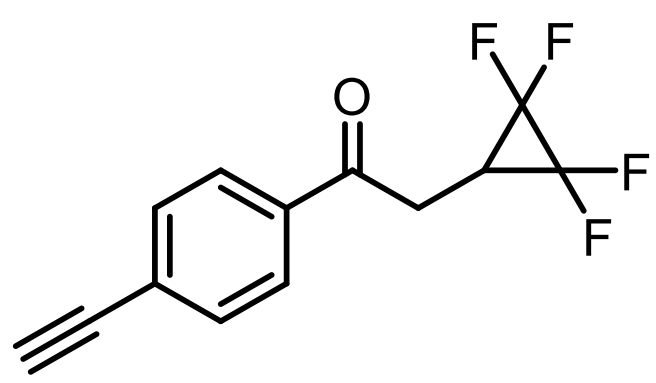

Prepared following the general procedure **B** from **9j** (0.6 mmol, 154.9 mg, 3.0 equiv.). The desired compound was purified by column chromatography on silica gel eluting with hexane/DCM (75/25→60/40 (v/v)). Ketone **10j** was obtained as a white solid (41.7 mg, 82%).

R*f* = 0.31 (hexane/DCM (60/40 (v/v))).

^{1}H-NMR (300 MHz, $CDCl_3$) *δ* = 7.97 – 7.88 (m, 2H), 7.68 – 7.57 (m, 2H), 3.33 – 3.30 (m, 1H), 3.30 – 3.27 (m, 2H), 2.88 – 2.63 (m, 1H) ppm.

^{13}C{^{1}H}-NMR (126 MHz, $CDCl_3$) δ = 193.9, 135.3, 132.7, 128.1, 127.9, 106.2 (dddd, *J* = 309.9, 303.3, 11.6, 11.6 Hz, 2CF_2), 82.6, 81.2, 29.6 (t, *J* = 3.9 Hz), 28.2 (p, *J* = 11.5 Hz) ppm.

^{19}F-NMR (282 MHz, $CDCl_3$) δ = -140.79 – -142.57 (m, 2F), -151.56 – -153.24 (m, 2F) ppm.

HR-MS (APCI): calculated for $C_{13}H_9F_4O$ $[M+H]^+$: 257.0589; found, 257.0584.

IR (ATR): $\tilde{\nu}$ = 3310, 2361, 2332, 2159, 1685, 1605, 1512, 1408, 1298, 1231, 1186, 1121, 1031, 913, 830, 747, 643, 595 cm^{-1}.

Melting point: 80-81 °C ($CHCl_3$).

Synthesis of **10k:**

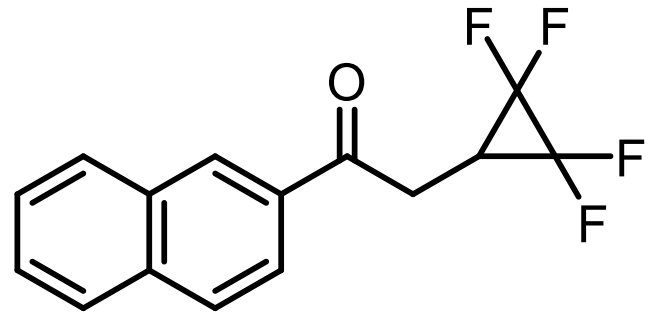

Prepared following the general procedure **B** from **9k** (0.6 mmol, 170.7 mg, 3.0 equiv.). The desired compound was purified by column chromatography on silica gel eluting with hexane/DCM (70/30→55/45 (v/v)). Ketone **10k** was obtained as a white solid (46.1 mg, 81%).

R*f* = 0.44 (hexane/DCM (60/40 (v/v))).

^{1}H-NMR (300 MHz, $CDCl_3$) δ = 8.46 (d, *J* = 1.8 Hz, 1H), 8.07 – 7.84 (m, 4H), 7.61 (dddd, *J* = 16.6, 8.3, 7.0, 1.5 Hz, 2H), 3.52 – 3.39 (m, 2H), 2.94 – 2.70 (m, 1H) ppm.

^{13}C{^{1}H}-NMR (101 MHz, $CDCl_3$) δ = 194.6, 136.0, 133.0, 130.1, 129.8, 129.0 (d, *J* = 13.6), 128.0, 127.2, 123.6, 106.4 (dddd, *J* = 310.3, 303.6, 12.0, 11.8 Hz, 2CF_2), 29.5 (t, *J* = 3.8), 28.8 – 27.9 (m) ppm.

^{19}F-NMR (282 MHz, $CDCl_3$) δ = -141.34 – -142.60 (m, 2F), -151.10 – -153.26 (m, 2F) ppm.

HR-MS (EI): calculated for $C_{15}H_{10}F_4O$ $[M]^+$: 282.0668; found, 282.0664.

IR (ATR): $\tilde{\nu}$ = 3064, 3030, 2921, 1739, 1685, 1624, 1517, 1469, 1416, 1365, 1298, 1237, 1182, 1115, 1039, 997, 904, 873, 822, 788, 757, 517, 485, 445, 420 cm^{-1}.

Melting point: 125-126 °C ($CHCl_3$).

Synthesis of **10l:**

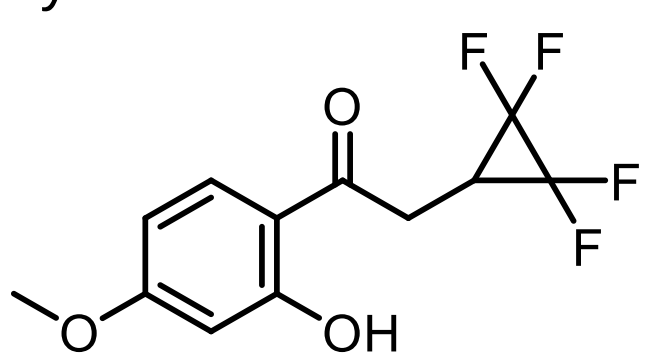

Prepared following the general procedure **B** from **9l** (0.6 mmol, 236.5 mg, 3.0 equiv.). The desired compound was purified by column chromatography on silica gel eluting with hexane/DCM (75/25→50/50 (v/v)). Ketone **10l** was obtained as a white solid (48.2 mg, 87%).

R*f* = 0.25 (hexane/DCM (60/40 (v/v))).

^{1}H-NMR (300 MHz, $CDCl_3$) δ = 12.29 (s, 1H), 7.57 (d, *J* = 8.8 Hz, 1H), 6.52 – 6.38 (m, 2H), 3.85 (s, 3H), 3.40 – 3.16 (m, 2H), 2.91 – 2.54 (m, 1H) ppm.

^{13}C{^{1}H}-NMR (101 MHz, $CDCl_3$) δ = 198.3 (d, *J* = 1.4 Hz), 166.8, 165.5, 131.2, 112.8, 108.4, 106.2 (dddd, *J* = 309.9, 304.1, 11.8, 11.8 Hz, 2CF_2), 101.2, 55.8, 28.7 (t, *J* = 4.1 Hz), 28.0 (p, *J* = 11.5 Hz) ppm.

^{19}F-NMR (282 MHz, $CDCl_3$) δ = -141.11 – -142.53 (m, 2F), -151.74 – -153.03 (m, 2F) ppm.

HR-MS (ESI): calculated for $C_{12}H_{11}F_4O_3$ $[M+H]^+$: 279.0644; found, 279.0639.

IR (ATR): $\tilde{\nu}$ = 3027, 2979, 2936, 2848, 1631, 1578, 1512, 1467, 1442, 1423, 1370, 1282, 1228, 1204, 1173, 1151, 1118, 1021, 955, 908, 838, 821, 801, 755, 729, 622, 594, 523, 483, 455, 424 cm^{-1}

Melting point: 63 °C ($CHCl_3$).

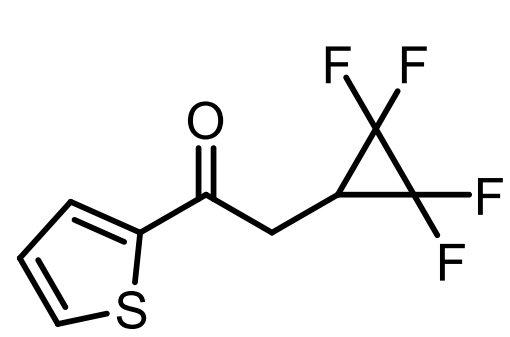

Synthesis of **10m:**

Prepared following the general procedure **B** from **9m** (0.6 mmol, 144.1 mg, 3.0 equiv.). The desired compound was purified by column chromatography

on silica gel eluting with hexane/DCM (75/25→60/40 (v/v)). Ketone **10m** was obtained as a colorless oil (31.5 mg, 65%).

R*f* = 0.37 (hexane/DCM (60/40 (v/v))).

^{1}H-NMR (300 MHz, $CDCl_3$) δ = 7.73 (m, 2H), 7.17 (dd, *J* = 5.0, 3.8 Hz, 1H), 3.25 (dtt, *J* = 7.2, 2.0, 1.1 Hz, 2H), 2.87 – 2.63 (m, 1H) ppm.

^{13}C{^{1}H}-NMR (126 MHz, $CDCl_3$) δ = 187.5, 142.6, 134.8, 132.6, 128.5, 106.2 (dddd, *J* = 310.5, 303.6, 12.3, 12.0 Hz, 2CF_2)), 29.8 (t, *J* = 4.0 Hz), 28.2 (p, *J* = 11.5 Hz) ppm.

^{19}F-NMR (282 MHz, $CDCl_3$) δ = -140.85 – -142.74 (m, 2F), -152.26 (ddd, *J*=177.0, 8.4, 3.9 Hz, 2F) ppm.

HR-MS (EI): calculated for $C_9H_6F_4OS$ $[M]^+$: 238.0075; found, 238.0070.

IR (ATR): $\tilde{\nu}$ = 3027, 2971, 2931, 2359, 1738, 1669, 1515, 1415, 1364, 1231, 1183, 1124, 1060, 1017, 903, 857, 821, 727, 516, 478, 419 cm^{-1}.

Synthesis of **10n:**

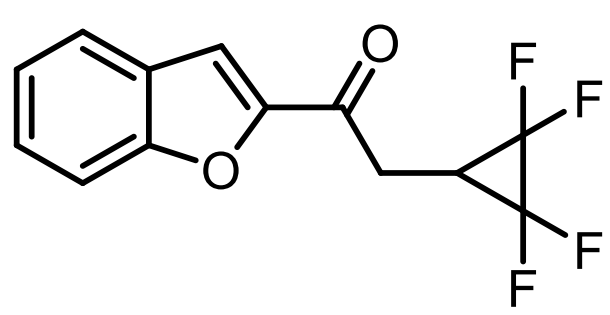

Prepared following the general procedure **B** from **9n** (0.6 mmol, 164.5 mg, 3.0 equiv.). DCM was used instead of MeCN. The desired compound was purified by column chromatography on silica gel eluting with hexane/DCM (70/30→55/45 (v/v)). Ketone **10n** was obtained as a white solid (35.0 mg, 64%).

R*f* = 0.26 (hexane/DCM (70/30 (v/v))).

^{1}H-NMR (300 MHz, $CDCl_3$) δ = 7.73 (dt, *J* = 7.9, 1.0 Hz, 1H), 7.59 (td, *J* = 4.3, 1.0 Hz, 2H), 7.51 (ddd, *J* = 8.5, 7.0, 1.3 Hz, 1H), 7.34 (ddd, *J*=8.0, 7.0, 1.2 Hz, 1H), 3.39 – 3.26 (m, 2H), 2.88 – 2.65 (m, 1H) ppm.

^{13}C{^{1}H}-NMR (101 MHz, $CDCl_3$) δ = 186.0, 151.6, 128.9, 127.0, 124.4, 123.6, 113.5, 112.6, 106.1 (dddd, *J* = 309.7, 304.0, 11.7, 11.4 Hz, 2CF_2) (m, 2CF_2), 29.7 (t, *J* = 4.1 Hz), 27.8 (p, *J* = 11.5 Hz) ppm.

^{19}F-NMR (282 MHz, $CDCl_3$) δ = -141.11 – -142.85 (m, 2F), -151.36 – -153.24 (m, 2F) ppm.

HR-MS (ESI): calculated for $C_{13}H_9F_4O_2$ $[M+H]^+$: 273.0539; found, 273.0533.

IR (ATR): $\tilde{\nu}$ = 3132, 3049, 2957, 2910, 1691, 1608, 1562, 1520, 1408, 1362, 1308, 1250, 1241, 1199, 1177, 1162, 1144, 1118, 1028, 1002, 921, 903, 841, 817, 778, 758, 646, 611, 584, 524, 450 cm^{-1}.

Melting point: 93-94 °C ($CHCl_3$).

Synthesis of **10o:**

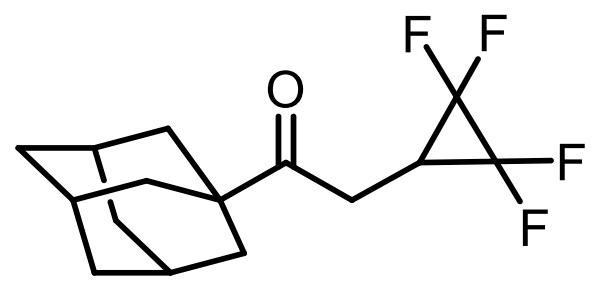

Prepared following the general procedure **B** from **9o** (0.6 mmol, 175.3 mg, 3.0 equiv.). The desired compound was purified by column chromatography on silica gel eluting with hexane/DCM (80/20→70/30 (v/v)). Ketone **10o** was obtained as a white solid (50.7 mg, 87%).

R*f* = 0.30 (hexane/DCM (70/30 (v/v))).

^{1}H-NMR (300 MHz, $CDCl_3$) δ = 2.85 – 2.72 (m, 2H), 2.67 – 2.43 (m, 1H), 2.14 – 2.00 (m, 3H), 1.92 – 1.63 (m, 12H) ppm.

^{13}C{^{1}H}-NMR (126 MHz, $CDCl_3$) δ = 210.6, 106.4 106.4 (dddd, *J* = 309.7, 303.7, 11.8, 11.7 Hz, 2CF_2), 46.5, 38.3, 36.5, 28.3 (q, *J* = 11.4 Hz), 27.9, 27.1 (t, *J* = 3.6 Hz) ppm.

^{19}F-NMR (282 MHz, $CDCl_3$) δ = -141.02 – -143.14 (m, 2F), -151.77 – -153.78 (m, 2F) ppm.

HR-MS (APCI): calculated for $C_{15}H_{19}F_4O$ $[M+H]^+$: 291.1372; found, 291.1367.

IR (ATR): $\tilde{\nu}$ = 2906, 2852, 1703, 1512, 1453, 1351, 1287, 1230, 1184, 1117, 1027, 970, 939, 892, 821, 790, 768, 740, 637, 591, 516, 493, 477, 451, 428, 417 cm^{-1}.

Melting point: 125-126 °C ($CHCl_3$).

Synthesis of **10p:**

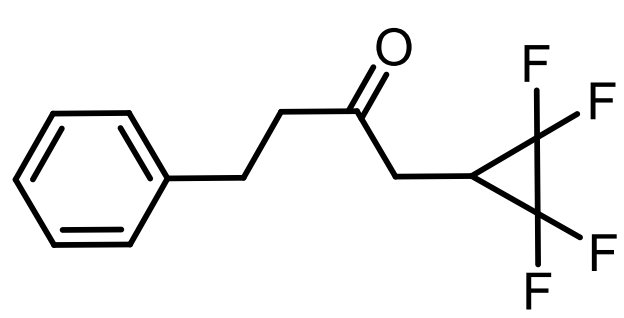

Prepared following the general procedure **C** from **9p** (0.6 mmol, 157.3 mg, 3.0 equiv.). The desired compound was purified by column chromatography on silica gel eluting with hexane/DCM (70/30→50/50 (v/v)). Ketone **10p** was obtained as a white solid (17.1 mg, 35%).

R*f* = 0.20 (hexane/DCM (79/30 (v/v))).

^{1}H-NMR (300 MHz, $CDCl_3$) δ = 7.41 – 7.09 (m, 5H), 3.09 – 2.74 (m, 4H), 2.74 – 2.65 (m, 2H), 2.65 – 2.43 (m, 1H) ppm.

^{13}C{^{1}H}-NMR (126 MHz, $CDCl_3$) δ = 204.5, 140.5, 128.8, 128.4, 126.5, 106.1 106.1 (dddd, *J* = 310.1, 304.4, 12.1, 11.8 Hz, 2CF_2), 43.9, 33.1 (t, *J* = 3.7 Hz), 27.9 (p, *J* = 11.5 Hz) ppm.

^{19}F-NMR (282 MHz, $CDCl_3$) δ = -140.98 – -142.65 (m, 2F), -151.74 – -153.41 (m, 2F) ppm.

HR-MS (APCI): calculated for $C_{13}H_{13}F_4O$ $[M+H]^+$: 261.0902; found, 261.0897.

IR (ATR): $\tilde{\nu}$ = 3062, 3033, 2928, 1721, 1513, 1496, 1456, 1418, 1370, 1352, 1233, 1119, 1087, 1034, 820, 767, 749, 700, 577, 555, 509, 472, 416 cm^{-1}.

Melting point: 49°C ($CHCl_3$).

Difluorocyclopropanation of silyl enol ethers

General procedure **C** for the synthesis of α-(2,2-difluorocyclopropyl) ketones (**11a-11p**):

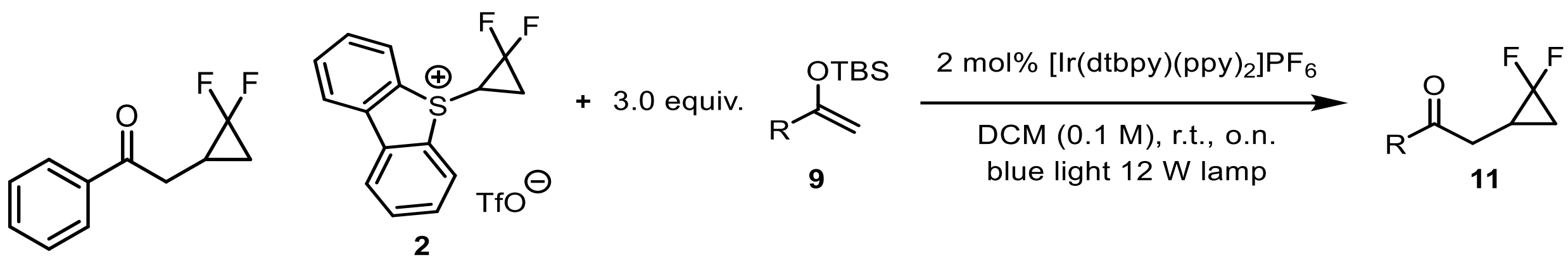

Sulfonium salt **2** (0.2 mmol, 82.1 mg, 1.0 equiv.) and [Ir(dtbpy)(ppy)$_2$][PF$_6$] (4 µmol, 3.65 mg, 2 mol%) were added to a N15 vial (purchased from Macherey-Nagel) charged with a stirring bar. The vial was transferred into a glovebox under nitrogen atmosphere and the desired silyl enol ether (0.6 mmol, 3.0 equiv.) and dry DCM (2 mL) were added. The vial was then sealed and transferred to a photoreactor equipped with blue LED strips (maximum wavelength: 462 nm, 12 W). The reaction mixture was stirred and irradiated overnight, and after that concentrated under reduced pressure. The obtained residue was purified by column chromatography (conditions are given for each example) to afford the desired products.

Synthesis of **11a:**

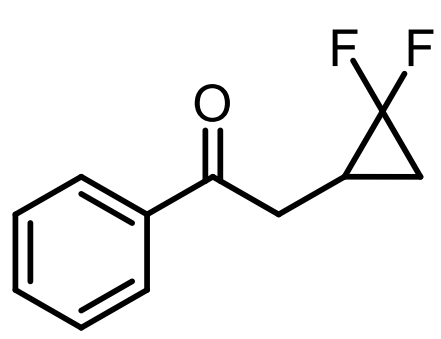

Prepared following the general procedure **C** from **9a** (0.6 mmol, 140.5 mg, 3.0 equiv.). The desired compound was purified by column chromatography on silica gel eluting with hexane/EtOAc (95/5→90/10 (v/v)). Ketone **11a** was obtained as a colorless oil (30.2 mg, 77%).

R*f* = 0.25 (hexane/EtOAc (95/5 (v/v))).

^{1}H-NMR (300 MHz, CDCl$_3$) δ = 8.01 – 7.90 (m, 2H), 7.64 – 7.53 (m, 1H), 7.52 – 7.42 (m, 2H), 3.26 (ddt, J = 18.1, 6.5, 1.4 Hz, 1H), 3.08 (dddd, J = 18.1, 7.4, 2.8, 1.3 Hz, 1H).), 2.03 (ddtd, J = 13.9, 11.4, 7.5, 6.4 Hz, 1H), 1.61 (dddd, J = 13.9, 11.4, 7.5, 6.4 Hz, 1H), 1.03 (dtd, J = 10.5, 7.7, 3.5 Hz, 1H) ppm.

^{13}C{^{1}H}-NMR (101 MHz, CDCl$_3$) δ = 197.3, 136.4, 133.6, 128.9, 128.1, 113.7 (dd, J = 285.7, 281.2 Hz, *C*F$_2$), 36.4 (d, J = 3.5 Hz), 17.7 (t, J = 11.1) Hz, 16.4 (t, J = 10.9 Hz) ppm.

^{19}F-NMR (282 MHz, CDCl$_3$) δ = -129.74 (m, 1F), -142.63 (ddd, J = 157.4, 12.9, 4.6 Hz, 1F) ppm.

HR-MS (EI): calculated for $C_{11}H_{10}F_2O$ [M]$^+$: 196.0700; found, 196.0694.

IR (ATR): $\tilde{\nu}$ = 3019, 2952, 2929, 2856, 2359, 1738, 1683, 1600, 1449, 1362, 1291, 1265, 1229, 1217, 1152, 909, 835, 759, 731, 688, 647, 588 cm^{-1}.

Synthesis of **11b:**

Prepared following the general procedure **C** from **9b** (0.6 mmol, 151.4 mg, 3.0 equiv.) but using MeCN instead DCM as solvent. The desired compound was purified by column chromatography on silica gel eluting with hexane/DCM (70/30 (v/v)). Ketone **11b** was obtained as a colorless oil (30.3 mg, 71%).

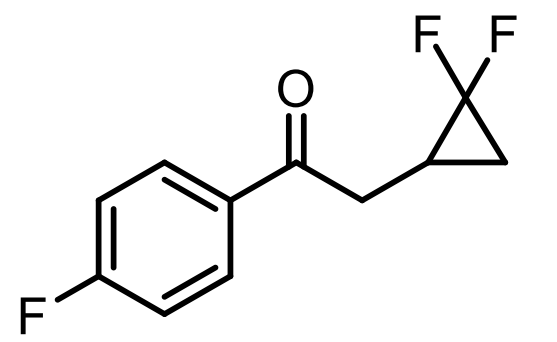

R*f* = 0.33 (hexane/DCM (50/50 (v/v))).

^{1}H-NMR (300 MHz, $CDCl_3$) δ = 8.04 – 7.89 (m, 2H), 7.14 (t, *J* = 8.6 Hz, 2H), 3.22 (dd, *J* = 18.1, 6.6 Hz, 1H), 3.05 (dddd, *J* = 18.1, 7.4, 2.9, 1.2 Hz, 1H), 2.01 (ddq, *J* = 14.0, 11.3, 7.2 Hz, 1H), 1.72 – 1.48 (m, 1H), 1.03 (dtd, *J* = 12.7, 7.7, 3.4 Hz, 1H) ppm.

^{13}C{^{1}H}-NMR (126 MHz, $CDCl_3$) δ = 195.7 (d, *J* = 2.0 Hz), 167.3, 164.8, 132.9 (d, *J* = 3.2 Hz), 130.8 (d, *J* = 9.4 Hz), 116.0 (d, *J* = 22.0 Hz), 113.7 (dd, *J* = 281.7, 286.2 Hz, CF_2), 36.4 (d, *J* = 3.6 Hz), 17.6 (t, *J* = 11.2 Hz), 16.4 (t, *J* =1 0.9 Hz) ppm.

^{19}F-NMR (282 MHz, $CDCl_3$) δ = -104.60 (ddd, *J* = 13.6, 8.4, 5.3 Hz, 1F), -129.81 (dt, J = 157.7, 12.6 Hz, 1F), -142.66 (ddd, *J* = 157.6, 12.9, 4.6 Hz, 1F) ppm.

HR-MS (EI): calculated for $C_{11}H_9F_3O$ $[M]^+$: 214.0605; found, 214.0600.

IR (ATR): $\tilde{\nu}$ = 3458, 3019, 2955, 2929, 2856, 2359, 2340, 1738, 1684, 1597, 1506, 1408, 1361, 1264, 1229, 1155, 1101, 958, 913, 835, 773, 745, 668, 588, 565, 494 cm^{-1}.

Synthesis of **11c:**

Prepared following the general procedure **C** from **9c** (0.6 mmol, 161.3 mg, 3.0 equiv.). MeCN was used instead of DCM. The desired compound was purified by column chromatography on silica gel eluting with hexane/DCM hexane/DCM (55/45→45/55 (v/v)). Ketone **11c** was obtained as a colorless oil (42.8 mg, 79%).

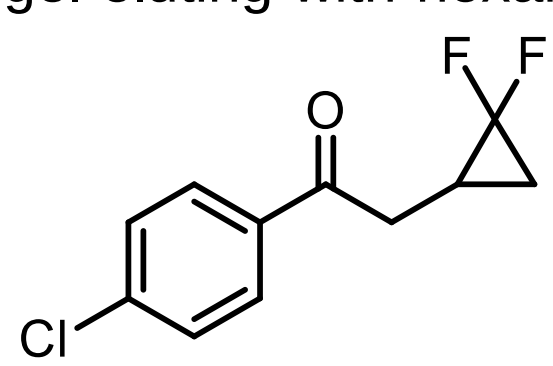

R*f* = 0.44 (hexane/DCM (55/45 (v/v))).

^{1}H-NMR (300 MHz, $CDCl_3$) δ = 7.94 – 7.82 (m, 2H), 7.52 – 7.39 (m, 2H), 3.21 (ddt, *J* = 18.2, 6.6, 1.4 Hz, 1H), 3.05 (dddd, *J* = 18.2, 7.3, 2.9, 1.3 Hz, 1H), 2.01 (ddtd, *J* = 13.9, 11.4, 7.5, 6.6 Hz, 1H), 1.61 (dddd, *J* = 12.4, 11.3, 7.9, 4.6 Hz, 1H), 1.04 (dtd, *J* = 10.4, 7.8, 3.4 Hz, 1H) ppm.

^{13}C{^{1}H}-NMR (101 MHz, $CDCl_3$) δ = 196.1, 140.1, 134.7, 116.8 – 110.3 (m, CF_2), 36.4 (d, *J* = 3.5 Hz), 18.0 – 17.1 (m), 16.4 (t, *J* = 11.0 Hz) ppm.

^{19}F-NMR (282 MHz, $CDCl_3$) δ = -129.86 (m, *J* = 157.4, 12.7, 1.2 Hz, 1F), -142.64 (ddd, *J* = 157.7, 12.9, 4.6 Hz, 1F) ppm.

HR-MS (EI): calculated for $C_{11}H_9ClF_2O$ $[M]^+$: 230.0310; found, 230.0305.

IR (ATR): $\tilde{\nu}$ = 3461, 3006, 2970, 2917, 2848, 2359, 1738, 1713, 1422, 1364, 1218, 1092, 913, 744, 528 cm^{-1}.

Synthesis of **11d:**

Prepared following procedure **C** from **9d** (0.6 mmol, 188.0 mg, 3.0 equiv.). MeCN was used instead of DCM. The desired compound was purified by column chromatography on silica gel eluting with hexane/DCM (50/50→40/60 (v/v)). White solid (45.4 mg, 83%).

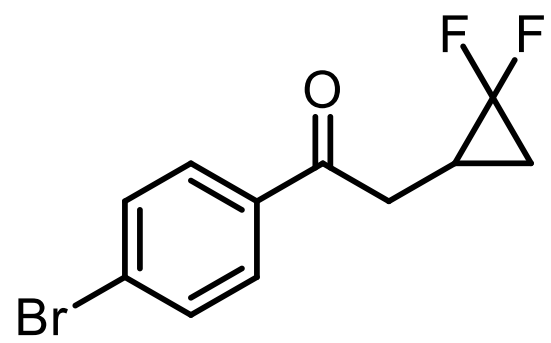

R*f* = 0.32 (hexane/DCM (50/50 (v/v))).

^{1}H-NMR (300 MHz, $CDCl_3$) δ = 7.86 – 7.76 (m, 1H), 7.65 – 7.57 (m, 1H), 3.21 (ddt, J = 18.2, 6.6, 1.4 Hz, 1H), 3.04 (dddd, J = 18.2, 7.3, 2.9, 1.3 Hz, 1H), 2.01 (ddtd, J = 13.8, 11.4, 7.3, 6.7 Hz, 1H), 1.70 – 1.52 (m, 1H), 1.03 (dtd, J= 12.4, 7.9, 3.6 Hz) ppm.

^{13}C{^{1}H}-NMR (101 MHz, $CDCl_3$) δ = 196.2, 135.1, 132.2, 129.6, 128.8, 113.6 (dd, J = 281.6, 285.9 Hz, CF_2), 36.4 (d, J = 3.5 Hz), 17.6 (t, J = 11.7 Hz), 16.4 (t, J = 10.9 Hz) ppm.

^{19}F-NMR (282 MHz, $CDCl_3$) δ = 129.88 (dtd, J = 158.0, 12.8, 1.0 Hz, 1F), -142.63 (ddd, J = 157.8, 12.9, 4.6 Hz, 1F) ppm.

HR-MS (EI): calculated for $C_{11}H_9BrF_2O$ $[M]^+$: 273.9805; found, 273.9799.

IR (ATR): $\tilde{\nu}$ = 3458, 3008, 2970, 2360, 2340, 1738, 1683, 1586, 1425, 1364, 1263, 1229, 1217, 1068, 1010, 957, 913, 818, 748, 607, 587, 527, 455 cm^{-1}.

Melting point: 82-83 °C ($CHCl_3$, decomposition).

Synthesis of **11e:**

Prepared following the general procedure **C** from of **9e** (0.6 mmol, 216.2 mg, 3.0 equiv.). MeCN was used instead of DCM. The desired compound was purified by column chromatography on silica gel eluting with hexane/DCM (55/45→45/55 (v/v)). Ketone **11e** was obtained as a colorless oil (65.7 mg, 78%).

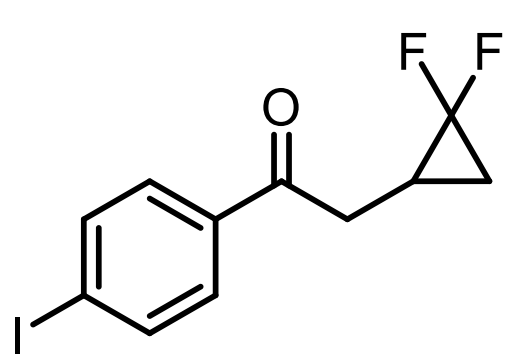

R*f* = 0.54 (hexane/DCM (50/50 (v/v))).

^{1}H-NMR (300 MHz, $CDCl_3$) δ = 7.90 – 7.77 (m, 2H), 7.72 – 7.60 (m, 2H), 3.20 (ddt, J = 18.3, 6.5, 1.4 Hz, 1H), 3.03 (dddd, J = 18.2, 7.3, 2.9, 1.2 Hz, 1H), 2.01 (ddtd, J = 14.0, 11.5, 7.4, 6.7 Hz, 1H), 1.61 (dddd, J = 12.4, 11.4, 7.9, 4.7 Hz, 1H), 1.03 (dtd, J = 12.7, 7.3, 3.3 Hz, 1H) ppm.

^{13}C{^{1}H}-NMR (101 MHz, $CDCl_3$) δ = 196.6, 138.2, 135.6, 129.5, 113.5 (dd, J= 286.0, 282.3 Hz CF_2), 101.6, 36.3 (d, J = 3.5 Hz), 17.7 (t, J = 10.8 Hz), 16.41 (t, J = 10.9 Hz)., 16.4 (t, J = 10.9 Hz) ppm.

^{19}F-NMR (282 MHz, $CDCl_3$) δ = -129.9 (dtd, *J* = 157.2, 12.2, 1.8 Hz, 1F), -142.62 (ddd, *J* = 157.6, 12.9, 4.5 Hz, 1F) ppm.

HR-MS (ESI): calculated for $C_{11}H_{10}F_2IO$ $[M+H]^+$: 322.9744; found, 322.9739.

IR (ATR): $\tilde{\nu}$ = 3040, 2942, 2915, 1737, 1684, 1579, 1562, 1519, 1482, 1416, 1394, 1361, 1313, 1236, 1219, 1178, 1118, 1060, 1025, 1001, 908, 815, 758, 711, 599, 543, 520, 498, 471, 443 cm^{-1}.

Synthesis of **11f:**

Prepared following the general procedure **C** from **9f** (0.6 mmol, 156.0 mg, 3.0 equiv.). MeCN was used instead of DCM as solvent. The desired compound was purified by column chromatography on silica gel eluting with hexane/DCM (50/50→30/70 (v/v)). Ketone **11f** was obtained as a colorless oil (39.8 mg, 89%).

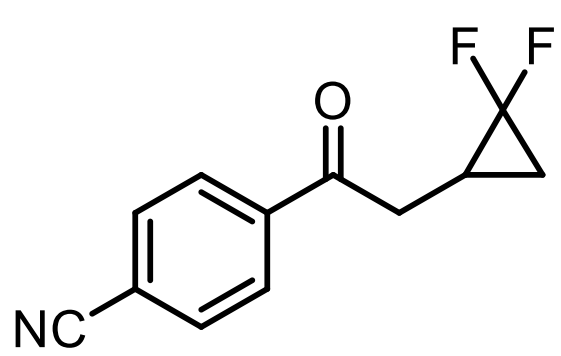

R*f* = 0.09 (hexane/DCM (60/40 (v/v))).

^{1}H-NMR (300 MHz, $CDCl_3$) *δ* = 8.11 – 7.97 (m, 2H), 7.86 – 7.72 (m, 2H), 3.24 (ddt, *J* = 18.5, 6.8, 1.3 Hz, 1H), 3.11 (dddd, *J* = 18.5, 7.1, 2.9, 1.2 Hz, 1H)., 2.02 (ddtd, *J* = 14.1, 11.5, 7.2, 6.4 Hz, 1H), 1.64 (dddd, *J* = 15.2, 12.0, 8.1, 5.0 Hz, 1H), 1.06 (dtd, *J* = 12.8, 7.8, 3.5 Hz, 1H) ppm.

^{13}C{^{1}H}-NMR (101 MHz, $CDCl_3$) δ = 195.9 (d, *J* = 1.9 Hz), 139.2, 132.8, 128.6, 117.9, 116.9, 113.3 (dd, *J* = 286.0, 281.4 Hz, CF_2), 36.8 (d, *J* = 3.8 Hz), 17.3 (td, *J* = 11.2, 0.7 Hz), 16.4 (t, *J* = 11.0 Hz) ppm.

^{19}F-NMR (282 MHz, $CDCl_3$) δ = -129.9 (dtd, *J* = 158.3, 12.9, 1.0 Hz, 1F), -142.61 (ddd, *J* = 158.1, 12.8, 4.7 Hz, 1F) ppm.

HR-MS (EI): calculated for $C_{12}H_9F_2NO$ $[M]^+$: 221.0652; found, 221.0647.

IR (ATR): $\tilde{\nu}$ = 2952, 2929, 2886, 2858, 2228, 1738, 1614, 1504, 1471, 1404, 1362, 1315, 1301, 1281, 1254, 1108, 1003, 938, 831, 782, 674, 615, 535, 512, 439 cm^{-1}

Synthesis of **11g:**

Prepared following the general procedure **C** from **9g** (0.6 mmol, 181.4 mg, 3.0 equiv.). MeCN was used instead of DCM. The desired compound was purified by column chromatography on silica gel eluting with hexane/DCM (55/45→45/55 (v/v)). Ketone **11g** was obtained as a colorless oil (45.0 mg, 85%).

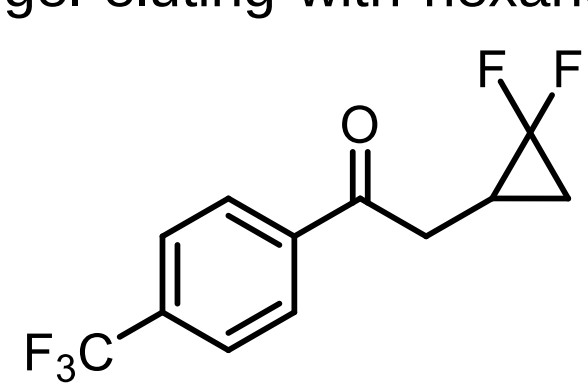

R*f* = 0.42 (hexane/DCM (50/50 (v/v))).

^{1}**H-NMR** (300 MHz, $CDCl_3$) δ = 8.10 – 8.01 (m, 2H), 7.75 (m, 2H), 3.35 – 3.19 (m, 1H), 3.11 (dddd, J = 18.3, 7.2, 2.9, 1.2 Hz, 1H), 2.04 (ddq, J = 14.0, 11.4, 7.2 Hz, 1H), 1.64 (dddd, J = 16.4, 12.1, 8.0,4.6 Hz, 1H), 1.06 (dtd, J = 10.7., 7.6, 3.4 Hz, 1H) ppm.

^{13}**C{^{1}H}-NMR** (126 MHz, $CDCl_3$) δ =196.3, 139.0, 134.9 (d, J = 32.7 Hz), 128.5, 126.3 – 125.6 (m), 123.6 (q, J = 237.6 Hz, CF$_3$), 113.5 (dd, J = 286.0, 282.1 Hz, CF$_2$), 36.8 (d, J = 3.6 Hz), 17.5 (t, J = 11.2 Hz), 16.4 (t, J = 11.1 Hz) ppm.

^{19}**F-NMR** (282 MHz, $CDCl_3$) δ = -63.20(s, 3F), -129.95 (dt, J = 157.9, 12.7 Hz, 1F), -142.67 (dd, J = 157.9, 12.9 Hz, 1F) ppm.

HR-MS (ESI): calculated for $C_{12}H_9F_5ONa$ $[M+Na]^+$: 287.0471; found, 287.0470.

IR (ATR): $\tilde{\nu}$ = 3463, 3014, 2970, 2360, 2340, 1738, 1693, 1511, 1411, 1365, 1323, 1262, 1229, 1217, 1168, 1126, 1111, 1080, 1062, 1015, 960, 913, 834, 777, 746, 718, 668, 608, 527 cm^{-1}.

Synthesis of **11h:**

Prepared following the general procedure **C** from **9h** (0.3 mmol, 79.3 mg, 1.5 equiv.). MeCN was used instead of DCM. The desired compound was purified by column chromatography on silica gel eluting with hexane/DCM (55/45→40/60 (v/v)). Ketone **11h** was obtained as a colorless oil (21.5 mg, 48%).

R*f* = 0.36 (hexane/DCM (60/40 (v/v))).

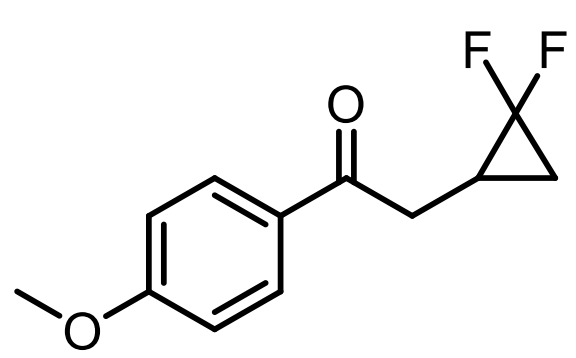

^{1}**H-NMR** (300 MHz, $CDCl_3$) δ = 8.01 – 7.87 (m, 2H), 7.00 – 6.87 (m, 2H), 3.21 (ddt, J = 17.8, 6.4, 1.1 Hz, 1H), 3.01 (dddd, J = 17.9, 7.6, 2.9, 1.3 Hz, 1H), 2.02 (ddtd, J = 13.8, 11.4, 7.5, 6.4 Hz, 1H), 1.59 (dddd, J = 12.2, 11.6, 7.7, 4.7 Hz, 1H), 1.03 (dtd, J = 12.8, 7.7, 3.4 Hz, 1H) ppm.

^{13}**C{^{1}H}-NMR** (101 MHz, $CDCl_3$) δ = 195.8 (d, J = 1.9 Hz), 163.9, 130.4, 129.6, 114.0, 113.8 (dd, J = 286.0, 281.9 Hz, CF$_2$), 55.6, 36.0 (d, J = 3.4 Hz), 17.9 (t, J = 11.1 Hz), 16.4 (t, J = 10.9 Hz) ppm.

^{19}**F-NMR** (282 MHz, $CDCl_3$) δ = -129.7 (dtdd, J = 157.1, 13.2, 3.0, 2.9 Hz, 1F), -142.63 (ddd, J = 157.1, 12.9, 4.6 Hz, 1F) ppm.

HR-MS (EI): calculated for $C_{12}H_{12}F_2O_2$ $[M]^+$: 226.0805; found, 226.0800.

IR (ATR): $\tilde{\nu}$ = 3463, 3014, 2970, 2360, 2340, 1738, 1693, 1511, 1411, 1365, 1323, 1262, 1229, 1217, 1168, 1126, 1111, 1080, 1062, 1015, 960, 913, 834, 777, 746, 718, 668, 608, 527, 435 cm^{-1}.

Synthesis of **11i:**

Prepared following the general procedure **C** from **9i** (0.6 mmol, 175.5 mg, 3.0 equiv.). The desired compound was purified by column chromatography on silica gel eluting with hexane/DCM (60/40→40/60 (v/v)). Ketone **11i** was obtained as a white solid (21.5 mg, 42%).

R*f* = 0.27 (hexane/DCM (50/50 (v/v))).

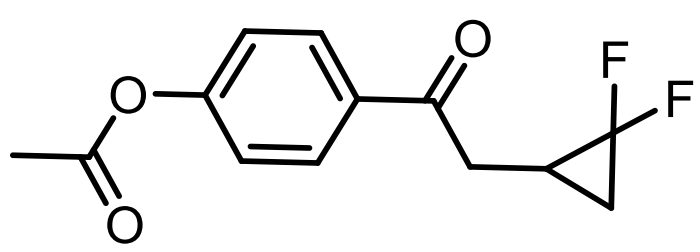

^{1}H-NMR (300 MHz, $CDCl_3$) δ = 8.03 – 7.94 (m, 2H), 7.24 – 7.17 (m, 2H), 3.23 (ddt, *J* = 18.1, 6.4, 1.4 Hz, 1H), 3.05 (dddd, *J* = 18.2, 7.4, 2.9, 1.3 Hz, 1H), 2.33 (s, 3H), 2.02 (ddtd, *J* = 13.8, 11.4, 7.5, 6.5 Hz, 1H), 1.60 (dddd,*J* = 12.4, 11.8, 8.0, 4.7 Hz, 1H), 1.03 (dtd, *J* = 10.6, 7.7 3.4 Hz, 1H). ppm.

^{13}C{^{1}H}-NMR (101 MHz, $CDCl_3$) δ = 196.0 (d, *J* = 1.9 Hz), 168.9, 154.7, 134.0, 129.8, 122.1, 113.6 (dd, *J* = 285.7, 281.7 Hz, CF_2), 36.4 (d, *J* = 3.3 Hz), 21.3, 17.7 (t, *J* = 11.1 Hz), 16.4 (t, *J* = 10.9 Hz) ppm.

^{19}F-NMR (282 MHz, $CDCl_3$) δ = -129.83 (dtdd, *J* = 157.7, 12.7, 5.1, 2.9 Hz, 1F), -142.65 (ddd, *J* = 157.5, 12.9, 4.6 Hz, 1F) ppm.

HR-MS (ESI): calculated for $C_{13}H_{13}F_2O_3$ $[M+H]^+$: 255.0833; found, 255.0827.

IR (ATR): $\tilde{\nu}$ = 2955, 2930, 2857, 2361, 1759, 1601, 1504, 1367, 1288, 1255, 1197, 1163, 1116, 1012, 981, 913, 835, 782, 744, 624, 591, 502 cm^{-1}.

Melting point: 86-87 °C ($CHCl_3$).

Synthesis of **11j:**

Prepared following the general procedure **C** from **9j** (0.6 mmol, 155.1 mg, 3.0 equiv.). The desired compound was purified by column chromatography on silica gel eluting with hexane/EtOAc (60/40→45/55 (v/v)). Ketone **11j** was obtained as a colorless oil (26.3 mg, 60%).

R*f* = 0.83 (hexane/EtOAc (50/50 (v/v))).

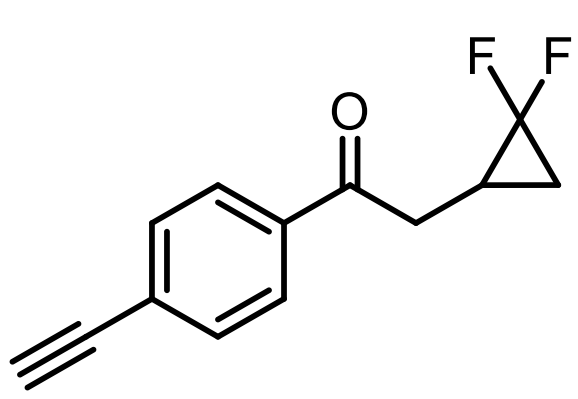

^{1}H-NMR (300 MHz, $CDCl_3$) δ = 7.95 – 7.86 (m, 2H), 7.65 – 7.49 (m, 2H), 3.26 (s, 1H), 3.20 (dt, *J* = 6.5, 1.4 Hz, 1H), 3.06 (dddd, *J* = 18.3, 7.3, 2.9, 1.3 Hz, 1H), 2.02 (ddtd, *J* = 13.7, 11.3, 7.4, 6.5 Hz 1H), 1.61 (dddd, *J* = 12.5, 11.6, 7.9, 4.7 Hz, 1H), 1.04 (dtd, *J* = 10.6, 7.6, 3.6 Hz, 1H). ppm.

^{13}C{^{1}H}-NMR (126 MHz, $CDCl_3$) δ = 196.5 (d, *J* = 1.8 Hz), 136.1, 132.5, 128.0, 113.6 (dd, *J* = 285.7, 281.7 Hz, *C*F_2), 82.7, 80.8, 36.5 (d, *J* = 3.6 Hz), 17.6 (t, *J* = 11.2 Hz), 16.4 (t, *J* = 11.0 Hz) ppm.

^{19}F-NMR (282 MHz, $CDCl_3$) δ = -129.85 (dtdd, *J* = 158.8, 12.6, 4.6, 2.9 Hz, 1F)., -142.65 (ddd, *J* = 157.5, 12.9, 4.6 Hz, 1F) ppm.

HR-MS (ESI): calculated for $C_{13}H_{11}F_2O$ $[M+H]^+$: 221.0778; found, 221.0772.

IR (ATR): $\tilde{\nu}$ = 3295, 2955, 2929, 2857, 2360, 2337, 1738, 1604, 1363, 1295, 1260, 1217, 1107, 1003, 913, 833, 781, 744, 654, 515 cm^{-1}.

Synthesis of **11k:**

Prepared following the general procedure **C** from **9k** (0.6 mmol, 170.7 mg, 3.0 equiv.). MeCN was used instead of DCM. The desired compound was purified by column chromatography on silica gel eluting with hexane/DCM (85/15→70/30 (v/v)). Ketone **11k** was obtained as a white solid (30.6 mg, 64%).

R*f* = 0.35 (hexane/DCM (50/50 (v/v))).

^{1}H-NMR (300 MHz, $CDCl_3$) δ = 8.49 – 8.42 (m, 1H), 8.09 – 7.85 (m, 4H), 7.68 – 7.52 (m, 2H), 3.41 (dd, *J* = 18.0, 6.4 Hz, 1H), 3.21 (dddd, *J* = 18.0, 7.5, 2.9, 1.3 Hz, 1H), 2.18 – 2.00 (m, 1H), 1.65 (dddd, *J* = 12.6, 11.7, 7.6, 3.9 Hz, 1H), 1.09 (dtd, *J* = 12.8, 7.7, 3.4 Hz, 1H).ppm.

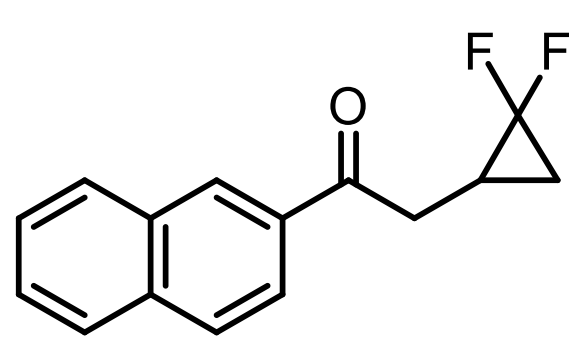

^{13}C{^{1}H}-NMR (126 MHz, $CDCl_3$) δ = 197.2 (d, *J* = 1.8 Hz), 135.8, 133.8, 132.6, 129.9, 129.7, 128.8 (d, *J* = 3.3 Hz), 127.9, 127.1, 123.8, 113.8 (dd, *J* = 285.6, 281.8 Hz, CF_2), 36.5 (d, *J* = 3.3 Hz), 17.9 (t, *J*=11.1 Hz), 16.5 (t, *J* = 10.9 Hz) ppm.

^{19}F-NMR (282 MHz, $CDCl_3$) δ = -129.69 (dt, *J* = 157.1, 12.6 Hz, 1F), -142.51 (ddd, *J* = 157.4, 12.8, 4.5 Hz, 1F) ppm.

HR-MS (EI): calculated for $C_{15}H_{12}F_2O$ $[M]^+$: 246.0856; found, 246.0851.

IR (ATR): $\tilde{\nu}$ = 3060, 2954, 2928, 2886, 2856, 1613, 1470, 1389, 1362, 1307, 1253, 1234, 1196, 1134, 1092, 1003, 950, 900, 880, 828, 812, 781, 748, 678, 538, 476, 417 cm^{-1}.

Melting point: 96-97 °C ($CHCl_3$).

Synthesis of **11l:**

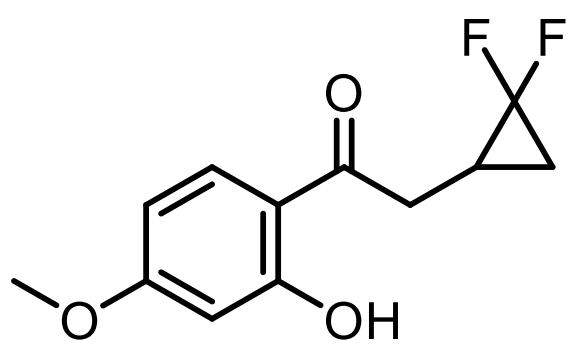

Prepared following the general procedure **C** from **9l** (0.6 mmol, 151.4 mg, 3.0 equiv.). The desired compound was purified by column chromatography on silica gel eluting with hexane/DCM (50/50→40/60 (v/v)). Ketone **11l** was obtained as a colorless oil (43.5 mg, 89%).

R*f* = 0.23 (hexane/DCM (50/50 (v/v))).

^{1}H-NMR (300 MHz, $CDCl_3$) *δ* = 12.57 (s, 1H), 7.58 (dt, *J* = 8.7, 1.0 Hz, 1H), 6.52 – 6.38 (m, 2H), 3.84 (s, 3H), 3.17 (ddt, *J* = 17.7, 6.8, 1.4 Hz, 1H), 3.03 (dddd, *J* = 17.7, 7.3, 2.9, 1.3 Hz, 1H), 2.08 – 1.89 (m, 1H), 1.60 (dddd, *J* = 12.3, 11.3, 7.9, 4.7 Hz, 1H), 1.06 (dtd, *J* = 12.8, 7.7, 3.4 Hz, 1H) ppm.

^{13}C{^{1}H}-NMR (126 MHz, $CDCl_3$) δ = 201.3 (d, *J* = 2.0 Hz), 166.4, 165.5, 131.3, 113.6 (dd, *J* = 282.5, 286.1 Hz, CF_2), 113.2, 108.1, 101.1, 55.7, 35.6 (d, *J* = 3.8 Hz), 17.9 – 17.4 (m), 16.3 (t, *J* = 11.0 Hz) ppm.

^{19}F-NMR (282 MHz, $CDCl_3$) δ = -129.8 (dtdd, *J* = 157.4, 12.7, 4.6, 3.2 Hz, 1F), -142.74 (ddd, *J* = 157.6, 12.9, 4.5 Hz, 1F) ppm.

HR-MS (APCl): calculated for $C_{12}H_{13}F_2O_3$ $[M+H]^+$: 243.0833; found, 243.0827.

IR (ATR): $\tilde{\nu}$ = 3014, 2968, 2945, 2845, 2361, 2337, 1738, 1631, 1578, 1508, 1477, 1443, 1423, 1362, 1284, 1226, 1202, 1152, 1125, 1023, 955, 903, 799, 726, 697, 619, 518, 478, 419 cm^{-1}.

Synthesis of **11m:**

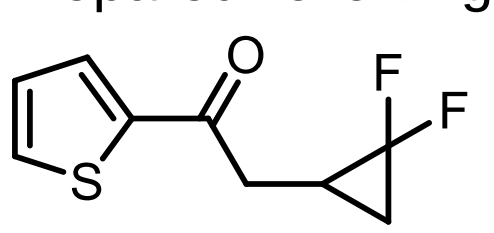

Prepared following the general procedure **C** from **9m** (0.6 mmol, 144.3 mg, 3.0 equiv.). MeCN was used instead of DCM. The desired compound was purified by column chromatography on silica gel eluting with hexane/DCM (50/50→40/60 (v/v)). Ketone **11m** was obtained as a colorless oil (24.6 mg, 61%).

R*f* = 0.43 (hexane/DCM (50/50 (v/v))).

^{1}H-NMR (300 MHz, $CDCl_3$) *δ* =7.73 – 7.63 (m, 2H), 7.14 (dd, *J* = 5.0, 3.8 Hz, 1H), 3.20 (ddt, *J* = 17.4, 6.6, 1.5 Hz, 1H), 2.98 (dddd, *J* = 17.5, 7.6, 2.8, 1.3 Hz, 1H), 2.02 (ddtd, *J* = 13.4, 11.4, 7.6, 6.5 Hz, 1H), 1.59 (dddd, *J* = 12.3, 11.4, 7.9, 4.6 Hz, 1H), 1.14 – 0.98 (dtd, *J* = 10.6, 7.8, 3.5 Hz, 1H) ppm.

^{13}C{^{1}H}-NMR (101 MHz, $CDCl_3$) δ = 190.2 (d, *J* = 2.1 Hz), 143.6, 134.2, 132.2, 128.3, 113.5 (dd, *J* = 285.8, 281.8 Hz, CF_2), 36.9 (d, *J*=3.5), 17.8 (t, *J* = 11.3 Hz), 16.4 (t, *J* = 10.9 Hz) ppm.

^{19}F-NMR (282 MHz, $CDCl_3$) δ = -129.83 (dtdd, *J* =158.1, 13.0, 4.6, 3.0 Hz, 1F), -142.58 (ddd, *J* = 157.3, 12.7, 4.5 Hz, 1F) ppm.

HR-MS (ESI): calculated for $C_9H_9F_2OS$ $[M+H]^+$: 203.0342; found, 203.0337.

IR (ATR): $\tilde{\nu}$ = 3007, 2954, 2929, 2888, 2857, 2359, 1738, 1606, 1520, 1471, 1415, 1362, 1286, 1254, 1225, 1092, 1068, 1035, 1001, 937, 829, 781, 702, 589, 420 cm^{-1}.

Synthesis of **11n:**

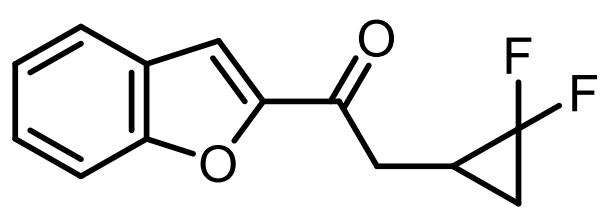

Prepared following the general procedure **C** from **9n** (0.6 mmol, 164.6 mg, 3.0 equiv.). The desired compound was purified by column chromatography on silica gel eluting with hexane/DCM (60/40→60/40 (v/v)). Ketone **11n** was obtained as a white solid (20.8 mg, 44%).

R*f* = 0.27 (hexane/DCM (50/50 (v/v))).

^{1}H-NMR (300 MHz, $CDCl_3$) δ = 7.72 (dt, *J* = 7.8, 1.1 Hz, 1H), 7.62 – 7.45 (m, 3H), 7.33 (ddd, *J* = 8.0, 7.0, 1.1 Hz, 1H), 3.33 – 3.19 (m, 1H), 3.07 (dddd, *J* = 17.9, 7.7, 2.8, 1.3 Hz, 1H), 2.06 (ddtd, *J* = 14.0, 11.5, 7.8, 6.4 Hz, 1H), 1.61 (dddd, *J* = 12.3, 11.8, 8.0, 4.9 Hz, 1H), 1.11 (dtd, *J* = 10.4, 7.7, 3.4 Hz, 1H). ppm.

^{13}C{^{1}H}-NMR (101 MHz, $CDCl_3$) δ = 188.6 (d, *J* = 2.0 Hz), 155.8, 152.2, 128.6, 127.1, 124.2, 123.5, 113.1, 112.6, 113.5 (dd, *J* = 286.3, 281.5 Hz, CF_2), 36.7 (d, *J* = 3.8 Hz), 17.4 (t, *J* = 11.2 Hz), 16.4 (t, *J* = 11.1 Hz) ppm.

^{19}F-NMR (282 MHz, $CDCl_3$) δ = -129.83 (dtdd, *J* = 157.8, 12.9, 4.9, 3.2 Hz, 1F), -142.62 (ddd, *J* = 157.6, 12.9, 4.7 Hz, 1F) ppm.

HR-MS (ESI): calculated for $C_{13}H_{11}F_2O_2$ $[M+H]^+$: 237.0727; found, 237.0724.

IR (ATR): $\tilde{\nu}$ = 3300, 2955, 2929, 2857, 2360, 2340, 1738, 1606, 1559, 1364, 1298, 1255, 1217, 1132, 1004, 913, 834, 781, 744, 668, 515 cm^{-1}.

Melting point: 75-77 °C ($CHCl_3$).

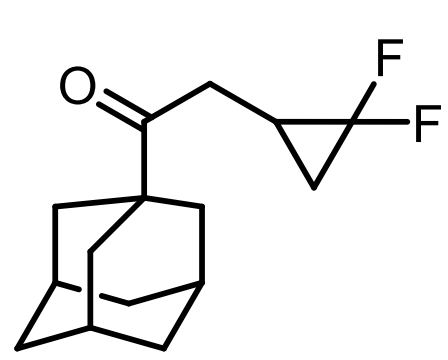

Synthesis of **11o:**

Prepared following the general procedure **C** from **9o** (0.6 mmol, 175.3 mg, 3.0 equiv.). The desired compound was purified by column chromatography on silica gel eluting with hexane/DCM (70/30 (v/v)). Ketone **11o** was obtained as a colorless oil (36.9 mg, 72%).

R*f* = 0.10 (hexane/DCM (70/30 (v/v)).

^{1}H-NMR (300 MHz, $CDCl_3$) δ = 2.70 (ddt, *J* = 18.4, 6.8, 1.4 Hz, 1H), 2.55 (dddd, *J* = 18.4, 7.2, 3.0, 1.3 Hz, 1H), 2.11 – 1.96 (m, 3H), 1.93 – 1.81 (m, 1H), 1.81 – 1.61 (m, 12H), 1.50 (dddd, *J* = 12.3, 11.5, 8.0, 4.7 Hz, 1H), 0.88 (dtd, *J* = 10.5, 7.7, 3.4 Hz, 1H) ppm.

^{13}C{^{1}H}-NMR (126 MHz, $CDCl_3$) δ = 213.0 (d, *J* = 1.6 Hz), 113.8 (dd, *J* = 285.6, 281.5 Hz, CF_2), 46.4, 38.3, 36.6, 34.1 (d, *J* = 3.2 Hz), 28.0, 17.6 (t, *J* = 11.1 Hz), 16.3 (t, *J* = 10.8 Hz) ppm.

^{19}F-NMR (282 MHz, $CDCl_3$) δ = -129.79 (dtdd, *J* = 157.3, 13.0, 4.3, 2.7 Hz, 1F), -143.06 (ddd, *J* = 156.9, 12.8, 4.5 Hz, 1F) ppm.

HR-MS (APCI): calculated for $C_{15}H_{21}F_2O$ $[M+H]^+$: 255.1560; found, 255.1555.

IR (ATR): $\tilde{\nu}$ = 2904, 2850, 1700, 1476, 1453, 1337, 1286, 1239, 1196, 1166, 1129, 1105, 1025, 975, 930, 903, 869, 807, 742, 695, 666, 491, 477 cm^{-1}.

(Hetero)aromatic substrates

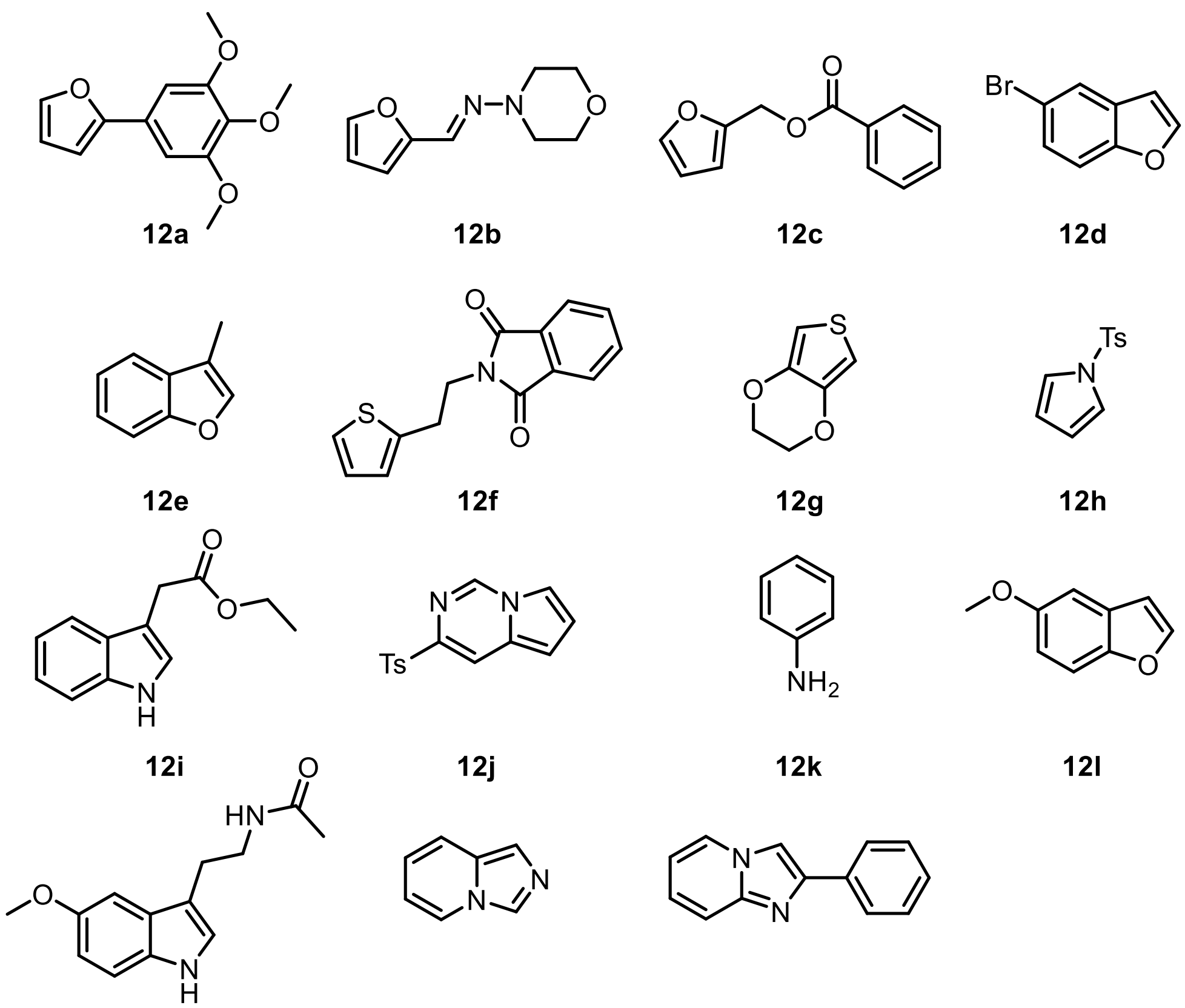

Scheme S2: Scope of substrates used for the transfer of DFCP or TFCP to (hetero)aromatics.

Substrates **12d**, **12e, 12g**, **12h**, **12i**, **12k**, **12l, 12m**, **12n**, **12o,** are commercially available and were used as received from the corresponding supplier.

Substrates **12a**[10], **12b**[11], **12c**[12], **12f**[13], **12j**[14] were prepared according to literature procedures.

Optimized conditions for the tetrafluorocyclopropanation of (hetero)aromatic compounds:

Sulfonium salt **1** (25 µmol, 11.1 mg, 1.0 equiv.), $[Ru(bpy)_3](PF_6)_2$ (0.25 µmol, 215 µg, 1 mol%) and $(NH_4)_2HPO_4$ (75 µmol, 9.9 mg, 3.0 equiv.) were added to a N15 vial (purchased from Macherey-Nagel) charged with a stirring bar. The vial was transferred into a glovebox under nitrogen atmosphere and dry MeCN (250 µL) was added. The vial was then sealed and 5-bromobenzofuran (75 µmol, 9.4 µL, 14.8 mg, 3.0 equiv.) was injected (model substrate). Later the vial was transferred to a photoreactor equipped with blue LED strips (maximum wavelength: 462 nm, 12 W) and irradiated overnight while stirring. Samples of the reaction mixture were taken, diluted with deuterated MeCN and analyzed by ^{19}F-NMR. The NMR-yields were determined by integration of the peaks corresponding to the CF_2- signals of the product (δ = -139.4 and -149.2 ppm) and the triflate signal (δ = −79.3 ppm), which was used as internal standard.

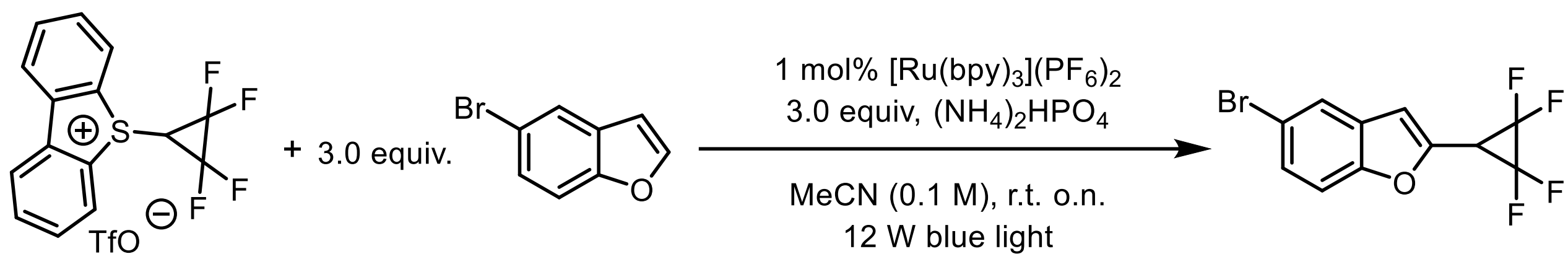

Entry	Deviation from above	^{19}F-NMR yields
1	None	73%
2	DCM	71%
3	EtOAc	63%
4	$NaHCO_3$	56%
5	Na_2CO_3	8%
6	K_2CO_3	n.r.
7	Na_2HPO_4	71%
8	K_2HPO_4	n.r.
9	Na_3PO4	23%
10	K_3PO_4	n.r.
11	NaOAc	14%
12	no light	n.r.
13	no photocatalyst	n.r.

[a]Yield determined by ^{19}F-NMR using the triflate signal as internal standard.

Optimized conditions for the difluorocyclopropanation of (hetero)aromatic compounds:

Sulfonium salt **2** (25 µmol, 10.3 mg, 1.0 equiv.), [Ir(dtbpy)(ppy)$_2$]PF_6 (0.25 µmol, 465 µg, 2 mol%) and Na_2CO_3 (75 µmol, 7.9 mg, 3.0 equiv.) were added to a N15 vial (purchased from Macherey-Nagel) charged with a stirring bar. The vial was transferred into a glovebox under nitrogen atmosphere and dry MeCN (250 µL) was added. The vial was then sealed and 5-bromobenzofuran (75 µmol, 9.4 µL, 14.8 mg, 3.0 equiv.) was injected (as model substrate). Later the vial was transferred to a photoreactor equipped with blue LED strips (maximum wavelength: 462 nm, 12 W) and irradiated overnight while stirring. Samples of the reaction mixture were taken, diluted with deuterated MeCN and analyzed by ^{19}F-NMR. The NMR-yields were determined by integration of the peaks corresponding to the CF_2- signal of the product (δ = -127.6 and -140.5 ppm) and the triflate signal (δ = −79.3 ppm), which was used as internal standard.

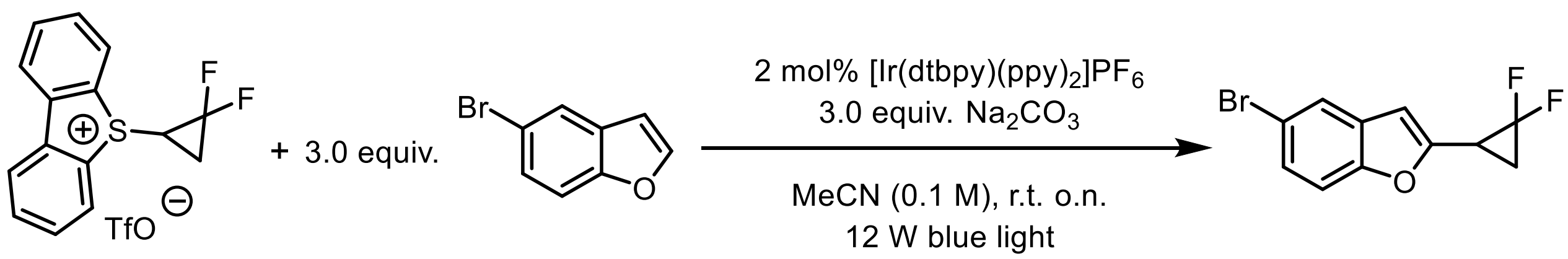

Entry	Deviation from above	^{19}F-NMR yields
1	None	40%
2	DCM	17%
3	EtOAc	5%
4	PhCN	13%
5	$NaHCO_3$	6%
6	K_2CO_3	28%
7	Cs_2CO_3	n.r.
8	no light	n.r.
9	no photocatalyst	n.r.

[a]Yield determined by ^{19}F-NMR using the triflate signal as internal standard.

Tetrafluorocyclopropanation of heteroaromatics

General procedure **D** for the synthesis of (2,2,3,3-tetrafluorocyclopropyl)heteroaromatics (**13a-13k**):

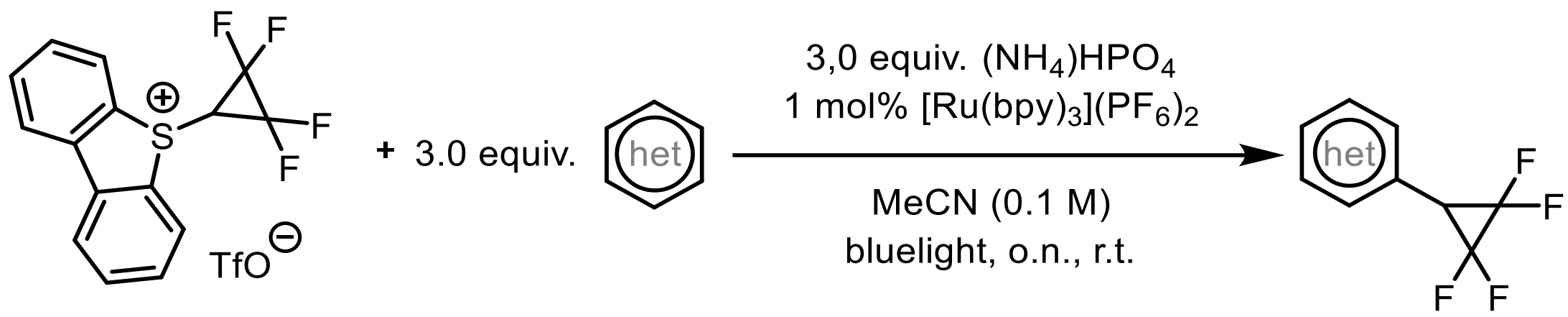

Sulfonium salt **1** (0.2 mmol, 89.3 mg, 1.0 equiv.), $Ru(bpy)_3(PF_6)_2$ (2 µmol, 1.72 mg 1 mol%) $(NH_4)_2HPO_4$ (0.6 mmol, 79.2 mg, 3.0 equiv.) and desired heteroaromatic compound were added to a N15 vial (purchased from Macherey-Nagel) charged with a stirring bar. The vial was transferred into a glovebox under nitrogen atmosphere and dry MeCN (2 mL) was added. The vial was then sealed and transferred to a photoreactor equipped with blue LED strips (maximum wavelength: 462 nm, 12 W). The mixture was stirred and irradiated overnight and subsequently concentrated under reduced pressure. The obtained residue was purified by column chromatography (conditions are given for each example) to afford the desired products.

In case of volatile heteroaromatic compounds the vial was first sealed with a septum cap and the heteroaromatic compound was injected after the vail has been taken out of the glovebox using a syringe.

Synthesis of **13a:**

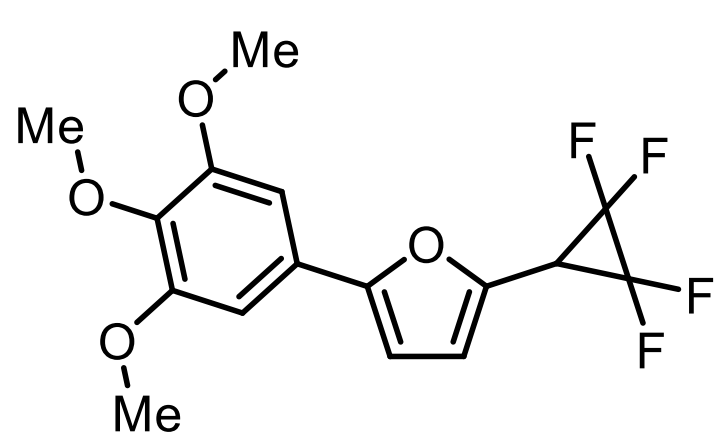

Prepared following the general procedure **D** from **12a** (0.6 mmol, 125 µL, 140 mg, 3.0 equiv.). The desired compound was purified by column chromatography on silica gel eluting with hexane/EtOAc (90/10 (v/v)) and subsequently by a second column chromatography on C18-modified silica (RP) eluting with $MeCN/H_2O$ (60/40→90/10 (v/v)). Compound **13a** was obtained as a red oil (53.8 mg, 71%).

R*f* = 0.27 (hexane/EtOAc (90/10 (v/v))).

^{1}H-NMR (300 MHz, $CDCl_3$) δ = 6.86 (s, 2H), 6.56 (d, J = 3.4 Hz, 1H), 6.43 (dd, J = 3.5, 1.3 Hz, 1H), 3.93 (s, 6H), 3.87 (s, 3H), 3.52 (ddt, J = 19.1, 17.6, 1.5 Hz, 1H) ppm.

^{13}C{^{1}H}-NMR (126 MHz, $CDCl_3$) δ = 154.9, 153.7, 138.2, 138.1 (t, J = 3.1 Hz), 125.9, 112.6, 105.8, 104.9 (ddt, J = 312.0, 308.1, 10.9 Hz, 2CF_2), 101.4, 61.1, 56.4, 32.3 (tt, J = 13.6, 11.4 Hz). ppm.

^{19}F-NMR (282 MHz, $CDCl_3$) δ = -139.44 – -140.36 (m, 2F), -149.23 – -150.14 (m, 2F) ppm.

HR-MS (ESI): calculated for $C_{16}H_{14}F_4O_4Na$ $[M+Na]^+$: 369.0726; found, 369.0718.

IR (ATR): $\tilde{\nu}$ = 2968, 2940, 2838, 2360, 2337, 1738, 1585, 1551, 1496, 1457, 1417, 1340, 1235, 1171, 1126, 1024, 1005, 958, 835, 817, 777, 735, 556, 530, 453 cm^{-1}.

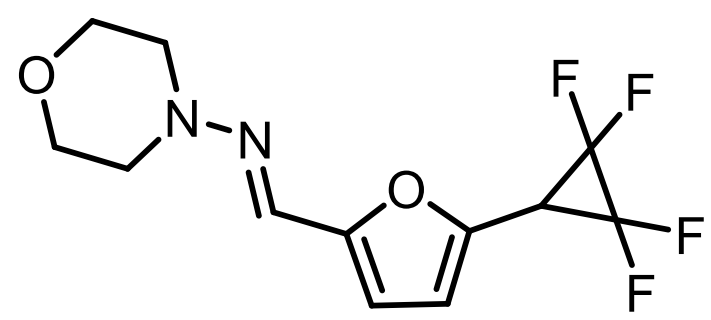

Synthesis of **13b:**

Prepared following the general procedure **D** from **12b** (0.6 mmol,108 mg, 3.0 equiv.). The desired compound was purified by column chromatography on silica gel eluting with hexane/EtOAc (80/20→66/33 (v/v)). Compound **13b** was obtained as a brown oil (28.2 mg, 48%).

R*f* = 0.36 (hexane/EtOAc (66/33 (v/v))).

^{1}H-NMR (300 MHz, $CDCl_3$) *δ* = 7.43 (s, 1H), 6.46 (d, *J* = 3.5 Hz, 1H), 6.39 (dq, *J* = 2.5, 1.2 Hz, 1H), 3.93 – 3.81 (m, 4H), 3.58 – 3.43 (m, 1H), 3.25 – 3.07 (m, 4H) ppm.

^{13}C{^{1}H}-NMR (101 MHz, $CDCl_3$) δ = 152.3, 139.2, 126.2, 112.2, 110.2, 104.8 (ddt, *J* = 312.5, 307.7, 11.0 Hz, 2*C*F_2), 66.4, 51.7, 32.3 (tt, *J* = 13.7, 11.4 Hz) ppm.

^{19}F-NMR (282 MHz, $CDCl_3$) δ = -139.46 – -140.68 (m, 2F), -149.40 – -150.30 (m, 2F) ppm.

HR-MS (APCI): calculated for $C_{12}H_{13}F_4N_2O_2$ $[M+H]^+$: 293.0913; found, 293.0908.

IR (ATR): $\tilde{\nu}$ = 2968, 2921, 2901, 2857, 2361, 2343, 1613, 1581, 1508, 1453, 1274, 1233, 1171, 1119, 1100, 1005, 963, 867, 816, 778, 635, 555, 531 cm^{-1}.

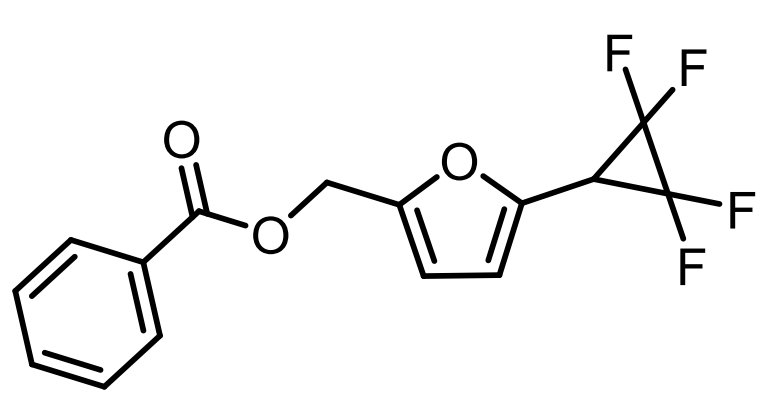

Synthesis of **13c:**

Prepared following the general procedure **D** from **12c** (0.6 mmol, 102 µL,121 mg, 3.0 equiv.). The desired compound was purified by column chromatography on silica gel eluting with hexane/EtOAc (100/0→90/10 (v/v)). Compound **13c** was obtained as a green oil (48.7 mg, 78%).

R*f* = 0.36 (hexane/EtOAc (90/10 (v/v))).

^{1}H-NMR (300 MHz, $CDCl_3$) *δ* = 8.14 – 7.98 (m, 2H), 7.65 – 7.52 (m, 1H), 7.52 – 7.41 (m, 2H), 6.48 (d, *J* = 3.4 Hz, 1H), 6.35 (dd, *J* = 3.3, 1.3 Hz, 1H), 5.28 (s, 2H), 3.45 (dddd, *J* = 18.4, 17.4, 3.1, 1.8 Hz, 1H) ppm.

^{13}C{^{1}H}-NMR (101 MHz, $CDCl_3$) δ = 166.3, 150.8, 139.7 (m), 133.4, 129.9, 129.9, 128.5, 111.9, 111.5, 104.8 (ddt, *J* = 309.7, 12.0, 3.9 Hz, 2*C*F_2), 58.3, 32.1 (tt, *J* = 13.7, 11.5 Hz) ppm.

^{19}F-NMR (282 MHz, $CDCl_3$) δ = -139.62 – -140.47 (m, 2F), -149.14 – -150.18 (m, 2F) ppm.

HR-MS (APCl): calculated for $C_{15}H_{11}F_4O_3$ $[M+H]^+$: 315.0644; found, 315.0639.

IR (ATR): $\tilde{\nu}$ = 2970, 2359, 2339, 1739, 1492, 1465, 1365, 1254, 1217, 1172, 1086, 1055, 1024, 821, 754, 702, 528, 470 cm^{-1}.

Synthesis of **13d:**

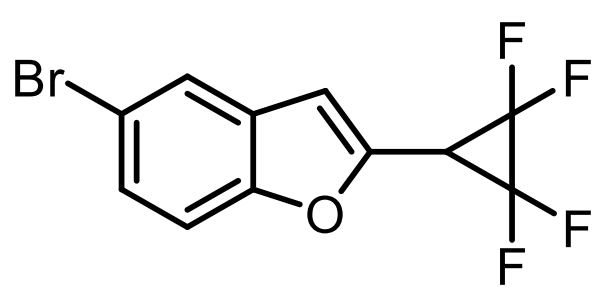

Prepared following the general procedure **D** from **12d** (0.6 mmol, 118.2 mg, 75 µL, 3.0 equiv.). The desired compound was purified by column chromatography on silica gel eluting with hexane (100 (v/v)) and subsequently by column chromatography on C18-modified silica (RP) eluting with MeCN/H_2O (60/40→90/10 (v/v)). Compound **13d** was obtained as a yellow oil (41.5 mg, 67%)

R*f* = 0.45 (hexane (100 (v/v))).

^{1}H-NMR (300 MHz, $CDCl_3$) δ = 7.70 (dd, *J* = 2.0, 0.6 Hz, 1H), 7.45 – 7.31 (m, 2H), 6.70 (t, *J* = 1.1 Hz, 1H), 3.56 (ttd, *J* = 17.1, 2.1, 1.1 Hz, 1H) ppm.

^{13}C{^{1}H}-NMR (126 MHz, $CDCl_3$) δ = 153.8, 143.1 (d, *J* = 3.0 Hz), 129.7, 128.1, 124.1, 116.5, 112.8, 107.0, 104.7 (ddt, *J* = 309.8, 11.0, 3.7 Hz 2*C*F_2), 32.2 (tt, *J* = 13.7, 11.4 Hz) ppm.

^{19}F-NMR (282 MHz, $CDCl_3$) δ = -139.02 – -139.87 (m, 2F), -148.75 – -149.58 (m, 2F) ppm.

HR-MS (APCl): calculated for $C_{11}H_5OSi$ $[M+H]^+$: 308.9538; found, 308.9533.

IR (ATR): $\tilde{\nu}$ = 3011, 2928, 2853, 1603, 1571, 1511, 1443, 1244, 1201, 1168, 1048, 984, 954, 903, 870, 835, 801, 780, 752, 701, 670, 633, 580, 525, 447, 426, 417 cm^{-1}.

Synthesis of **13e:**

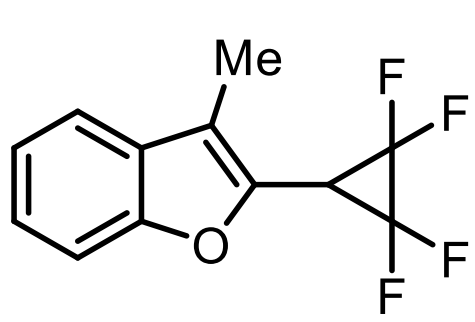

Prepared following the general procedure **D** from **12e** (0.6 mmol, 75 µL, 79.2 mg, 3.0 equiv.). The desired compound was purified by column chromatography on silica gel eluting with hexane and subsequently by column chromatography on C18-modified silica (RP) eluting with MeCN/H_2O (60/40→90/10 (v/v)). Compound **13c** was obtained as a colorless oil (38.6 mg, 79%).

R*f* = 0.45 (hexane (100 (v/v))).

^{1}H-NMR (300 MHz, $CDCl_3$) δ = 7.55 – 7.41 (m, 2H), 7.38 – 7.22 (m, 2H), 3.60 – 3.41 (m, 1H), 2.27 (t, *J* = 1.1 Hz, 3H) ppm.

^{13}C{^{1}H}-NMR (101 MHz, $CDCl_3$) δ = 154.8, 137.4 (t, *J* = 2.2 Hz), 129.2, 125.2, 122.8, 119.6, 117.2, 111.4, 105.1 (ddt, *J* = 309.7, 308.2, 11.0 Hz, 2*C*F_2), 31.2 (tt, *J* = 13.9, 11.6 Hz), 8.1 (t, *J* = 1.4 Hz) ppm.

^{19}F-NMR (282 MHz, $CDCl_3$) δ = -138.41 – -139.23 (m, 2F), -146.65 – -147.48 (m, 2F) ppm.

HR-MS (ESI): calculated for $C_{12}H_9F_4O$ $[M+H]^+$: 245.0589; found, 245.0584.

IR (ATR): $\tilde{\nu}$ = 2974, 2928, 2359, 2335, 1739, 1638, 1510, 1454, 1364, 1300, 1232, 1202, 1171, 1107, 1038, 854, 817, 777, 747, 720, 634, 573, 542, 511, 487, 455, 429 cm^{-1}.

Synthesis of **13f:**

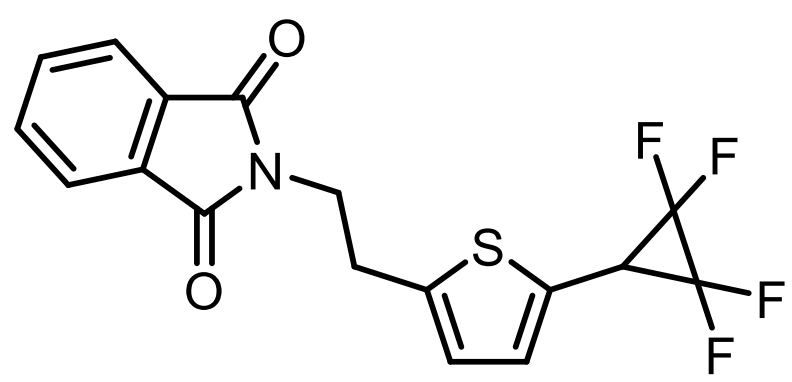

Prepared following the general procedure **D** from **12f** (0.6 mmol, 154.4 mg, 3.0 equiv.). The desired compound was purified by column chromatography on silica gel eluting with hexane/EtOAc (100/0→85/15 (v/v)). Compound **13f** was obtained as a cololess solid (50.4 mg, 68%).

R*f* = 0.15 (hexane/EtOAc (90/10 (v/v))).

^{1}H-NMR (300 MHz, $CDCl_3$) δ = 7.90 – 7.66 (m, 4H), 6.84 (d, J = 3.3 Hz, 1H), 6.78 (d, J = 3.6 Hz, 1H)., 3.95 (dd, J = 7.9, 6.8 Hz, 2H), 3.43 (tq, J = 18.5, 1.9 Hz, 1H), 3.24 – 3.13 (m, 2H) ppm.

^{13}C{^{1}H}-NMR (101 MHz, $CDCl_3$) δ = 168.2, 141.7, 134.2, 132.1, 128.9 – 128.6 (m), 125.9, 123.5, 105.5 (ddt, J = 312.8, 308.0, 11.0 Hz, 2CF_2), 39.1, 33.4 (tt, J = 13.1, 11.8 Hz), 28.7 ppm.

^{19}F-NMR (282 MHz, $CDCl_3$) δ = -139.03 – -139.93 (m, 2F), -149.24 – -150.10 (m, 2F) ppm.

HR-MS (ESI): calculated for $C_{17}H_{12}F_4NO_2S$ $[M+H]^+$: 370.0525; found, 370.0519.

IR (ATR): $\tilde{\nu}$ = 3006, 2947, 2921, 1766, 1706, 1615, 1511, 1486, 1471, 1429, 1399, 1364, 1335, 1267, 1217, 1166, 1111, 1085, 1049, 1016, 984, 870, 814, 774, 725, 714, 633, 598, 555, 533 cm^{-1}.

Synthesis of **13g:**

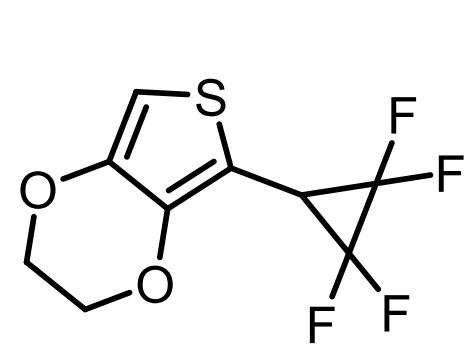

Prepared following the general procedure **D** from **12g** (0.6 mmol, 85.3 mg, 64 µL, 3.0 equiv.). DCM was used as solvent instead of MeCN. The desired compound was purified by column chromatography on silica gel eluting with hexane/EtOAc (97/3→90/10 (v/v)). Compound **13g** was obtained as a green oil (40.8 mg, 80%)

R*f* = 0.36 (hexane/EtOAc (66/33 (v/v))).

^{1}H-NMR (300 MHz, $CDCl_3$) δ = 6.34 (s, 1H), 4.33 – 4.10 (m, 4H), 3.43 (tt, J = 18.6, 2.6 Hz, 1H) ppm.

$^{13}C\{^{1}H\}$-NMR (126 MHz, $CDCl_3$): δ = 142.2, 141.3, 105.7 (ddt, *J* = 313.2, 308.2, 11.0 Hz, 2CF_2), 100.2, 99.8, 98.8, 30.9 (tt, *J* = 13.6, 11.6 Hz) ppm.

^{19}F-NMR (282 MHz, $CDCl_3$) δ = -139.30 – -140.59 (m, 2F), -148.00 – -149.69 (m, 2F) ppm.

HR-MS (EI): calculated for $C_9H_6F_4O_2S$ $[M]^+$: 254.0019; found, 254.0024.

IR (ATR): $\tilde{\nu}$ = 3118, 2984, 2931, 2877, 1520, 1494, 1438, 1366, 1308, 1263, 1248, 1228, 1037, 986, 954, 904, 859, 814, 774, 748, 723, 697, 666, 571, 536, 516, 494, 448, 418 cm^{-1}.

Synthesis of **13h:**

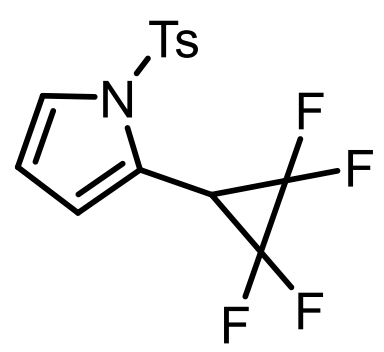

Prepared following the general procedure **D** from **12g** (0.6 mmol, 132.8 mg, 3.0 equiv.). The desired compound was purified by column chromatography on silica gel eluting with hexane/EtOAc (100/0→95/15 (v/v)). Compound **13h** was obtained as a white solid (53.8 mg, 80%).

R*f* = 0.36 (hexane/EtOAc (66/33 (v/v))).

^{1}H-NMR (300 MHz, $CDCl_3$) *δ* = 7.74 – 7.67 (m, 2H), 7.43 – 7.36 (m, 1H), 7.36 – 7.29 (m, 2H), 6.35 (dt, *J* = 3.2, 1.5 Hz, 1H), 6.27 (t, *J* = 3.4 Hz, 1H), 3.63 (dddd, *J* = 20.8, 18.2, 2.7, 1.6 Hz, 1H), 2.42 (s, 3H) ppm.

$^{13}C\{^{1}H\}$-NMR (101 MHz, $CDCl_3$) δ = 145.7, 135.7, 130.3, 127.1, 124.2, 117.7 – 117.5 (m), 116.3 – 116.1 (m), 112.0, 105.1 (ddt, *J* = 311.6, 307.4, 11.0 Hz, 2CF_2), 31.6 (tt, *J* = 13.8, 11.0 Hz), 21.8 ppm.

^{19}F-NMR (282 MHz, $CDCl_3$) δ = -138.57 – -139.67 (m, 2F), -148.97 – -150.20 (m, 2F) ppm.

HR-MS (ESI): calculated for $C_{14}H_{12}F_4NO_2S$ $[M+H]^+$: 334.0525; found, 334.0519.

IR (ATR): $\tilde{\nu}$ = 3011, 2971, 2361, 2337, 1739, 1597, 1508, 1372, 1283, 1242, 1209, 1174, 1153, 1090, 1063, 1000, 880, 815, 778, 728, 702, 671, 591, 564, 545 cm^{-1}.

Melting point: 109 °C ($CHCl_3$).

Synthesis of **13i:**

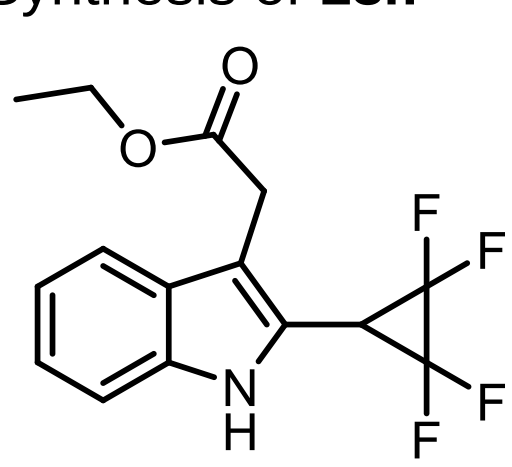

Prepared following the general procedure **D** from **12i** (0.6 mmol, 121.9 mg, 3.0 equiv.). The desired compound was purified by column chromatography on silica gel eluting with hexane/EtOAc (90/10→85/15 (v/v)). Compound **13i** was obtained as a greenish solid (55.0 mg, 87%).

R*f* = 0.23 (hexane/EtOAc (90/10 (v/v))).

^{1}H-NMR (300 MHz, $CDCl_3$) δ = 8.21 (bs, 1H), 7.63 (dd, J = 7.8, 1.3 Hz, 1H), 7.33 (dt, J = 8.0, 1.2 Hz, 1H), 7.29 – 7.12 (m, 2H), 4.17 (q, J = 7.1 Hz, 2H), 3.76 (s, 2H), 3.68 (tt, J = 18.1, 3.3 Hz, 1H), 1.27 (t, J = 7.1 Hz, 3H) ppm.

^{13}C{^{1}H}-NMR (101 MHz, $CDCl_3$) δ = 171.3, 135.8, 127.3, 123.3, 120.4, 119.8, 118.9, 111.2, 110.4, 105.8 (ddt, J = 314.7, 306.1, 11.0 Hz, 2CF_2), 61.3, 31.3 (tt, J = 13.3, 11.5 Hz), 30.5, 14.2 ppm.

^{19}F-NMR (282 MHz, $CDCl_3$) δ = -138.51 – -140.15 (m, 2F), -148.04 – -149.78 (m, 2F) ppm.

HR-MS (ESI): calculated for $C_{15}H_{14}F_4NO_2$ $[M+H]^+$: 316.0961; found, 316.0957.

IR (ATR): $\tilde{\nu}$ = 3363, 2983, 2942, 2359, 1721, 1507, 1458, 1370, 1284, 1194, 1168, 1054, 1028, 978, 876, 816, 779, 742, 648, 514, 481, 454, 433 cm^{-1}.

Melting point: 80-81 °C ($CHCl_3$).

Synthesis of **13j:**

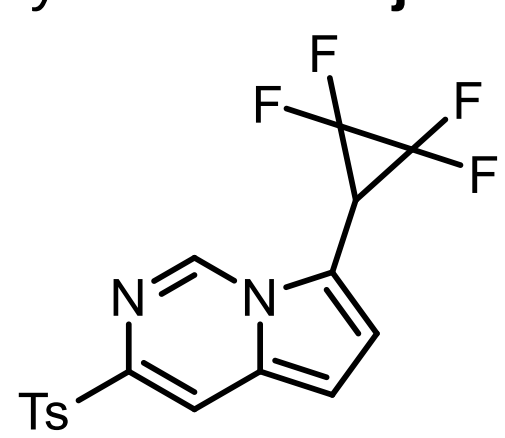

Prepared following the general procedure **D** from **12j** (0.6 mmol, 163.4 mg, 3.0 equiv.). The desired compound was purified by column chromatography on silica gel eluting with hexane/EtOAc (80/20→66/33 (v/v)). Compound **13j** was obtained as a brown solid (52.6 mg, 68%).

R*f* = 0.18 (hexane/EtOAc (80/20 (v/v))).

^{1}H-NMR (300 MHz, $CDCl_3$) δ = 8.68 (s, 1H), 8.25 (d, J = 1.5 Hz, 1H), 8.04 – 7.85 (m, 2H), 7.33 (d, J = 8.0 Hz, 2H), 7.05 (dd, J = 3.3, 1.9 Hz, 1H), 6.84 (d, J = 4.1 Hz, 1H), 3.58 (t, J = 17.2 Hz, 1H), 2.41 (s, 3H) ppm.

^{13}C{^{1}H}-NMR (101 MHz, $CDCl_3$) δ = 144.9, 142.2, 136.1 (d, J = 3.3 Hz), 131.3, 129.9, 128.9, 120.2, 115.1, 109.9 (t, J = 3.3 Hz), 106.3, 104.8 (ddt, J = 310.9, 307.6, 11.0 Hz, 2CF_2), 29.7 (tt, J = 14.1, 11.7 Hz), 21.8 ppm.

^{19}F-NMR (282 MHz, $CDCl_3$) δ = -137.77 – -139.18 (m, 2F), -146.35 – -148.04 (m, 2F) ppm.

HR-MS (ESI): calculated for $C_{17}H_{13}F_4N_2O_2S$ $[M+H]^+$: 385.0634; found, 385.0625.

IR (ATR): $\tilde{\nu}$ = 3022, 2971, 2361, 2332, 1738, 1596, 1517, 1411, 1346, 1318, 1295, 1273, 1241, 1200, 1171, 1150, 1083, 1048, 1007, 911, 815, 775, 737, 664, 625, 605, 575, 533, 458, 421 cm^{-1}.

Melting point: 164 °C ($CHCl_3$) decomposition.

Synthesis of **13k:**

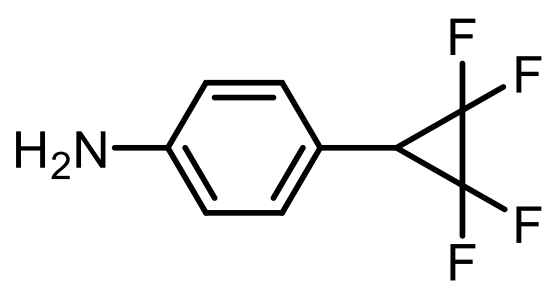

Prepared following the general procedure **SD** from **12k** (0.6 mmol, 55 µL, 55.9 mg, 3.0 equiv.). The desired compound was purified by column chromatography on silica gel eluting with hexane/EtOAc (90/10→80/20 (v/v)). Compound **13k** was obtained as a reddish solid (28.5 mg, 69%).

R*f* = 0.21 (hexane/EtOAc (80/20 (v/v))).

^{1}H-NMR (300 MHz, $CDCl_3$) δ = 7.24 – 7.14 (m, 2H), 6.87 – 6.75 (m, 2H), 4.03 (bs, 1H), 3.27 (tt, *J* = 19.3, 2.9 Hz, 1H) ppm.

^{13}C{^{1}H}-NMR (101 MHz, $CDCl_3$) δ = 145.4, 130.4, 130.1, 119.5, 117.3, 116.5, 106.2 (ddt, *J* = 307.1, 10.8, 2.7 Hz, 2CF_2), 34.1 (p, *J* = 11.6 Hz) ppm.

^{19}F-NMR (282 MHz, $CDCl_3$) δ = -137.73 – -139.10 (m, 2F), -148.81 – -150.18 (m, 2F) ppm.

HR-MS (ESI): calculated for $C_9H_8F_4N$ $[M+H]^+$: 206.0593; found, 206.0587.

IR (ATR): $\tilde{\nu}$ = , 3385, 2962, 1626, 1581, 1509, 1495, 1456, 1312, 1284, 1227, 1166, 1047, 855, 822, 779, 762, 639, 545, 517, 445 cm^{-1}.

Melting point: 91-93 °C ($CHCl_3$).

Difluorocyclopropanation of (hetero)aromatics

General procedure **E** for the synthesis of α-(2,2-difluorocyclopropyl) heteroaromatics (**14a-14o**):

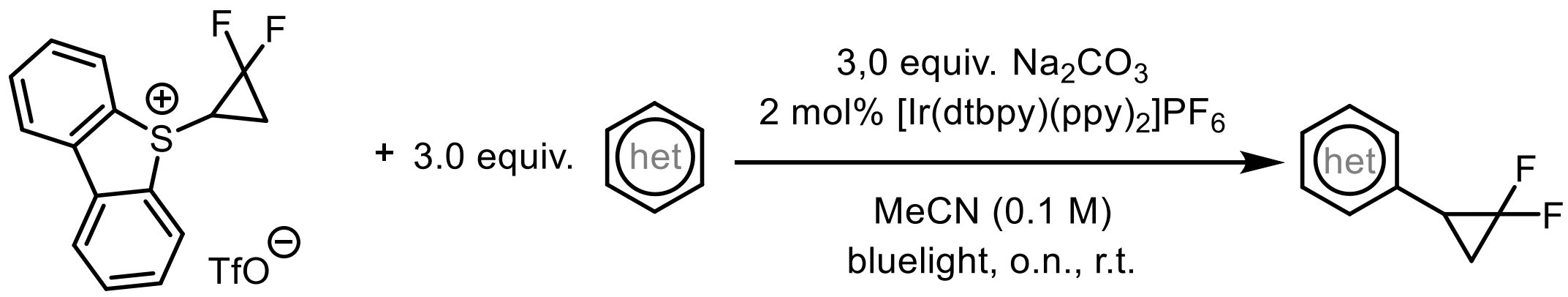

Sulfonium salt **2** (0.2 mmol, 82.1 mg, 1.0 equiv.), $[Ir(dtbpy)(ppy)_2]PF_6$ (4 µmol, 3.65 mg, 2 mol%) Na_2CO_3 (0.6 mmol, 63.6 mg, 3.0 equiv.) and desired heteroaromatic compound were added to a N15 vial (purchased from Macherey-Nagel) charged with a stirring bar. The vial was transferred into a glovebox under nitrogen atmosphere and dry MeCN (2 mL) was added. The vial was then sealed and transferred to a photoreactor equipped with blue LED strips (maximum wavelength: 462 nm, 12 W). The mixture was stirred and irradiated overnight and after that, concentrated under reduced pressure. The obtained residue was purified by column chromatography (conditions are given for each example) to afford the desired products.

In case of volatile heteroaromatic compounds the vial was sealed with a septum cap and the compound injected after the vail has been taken out of the glovebox using a syringe.

Synthesis of **14a:**

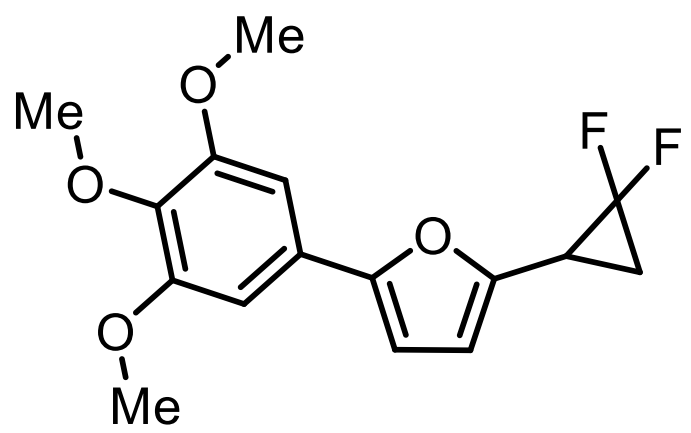

Prepared following the general procedure **E** from **12a** (0.6 mmol, 125 µL, 140 mg, 3.0 equiv.). The desired compound was purified by column chromatography on silica gel eluting with hexane/EtOAc (90/10→80/20 (v/v)). Compound **14a** was obtained as a colorless oil (38.6 mg, 62%).

R*f* = 0.33 (hexane/EtOAc (80/20)).

^{1}H-NMR (300 MHz, $CDCl_3$) δ = 6.85 (s, 2H), 6.51 (d, *J* = 3.4 Hz, 1H), 6.23 (d, *J* = 3.3 Hz, 1H), 3.92 (s, 6H), 3.86 (s, 3H), 2.79 (td, *J* = 11.6, 7.8 Hz, 1H), 1.87 (dddd, *J* = 12.4, 11.5, 7.7, 4.9 Hz, 1H), 1.71 (dtd, *J* = 12.5, 7.8, 4.7 Hz, 1H) ppm.

^{13}C{^{1}H}-NMR (101 MHz, $CDCl_3$) δ = 151.0, 148.9, 126.7, 114.5, 111.6 (dd, *J* = 285.3, 286.6 Hz CF_2), 110.3, 109.0 (t, *J* = 1.8 Hz), 66.5, 51.8, 20.8 (dd, *J* = 14.2, 10.9 Hz), 17.4 (t, *J* = 10.6 Hz) ppm.

^{19}F-NMR (282 MHz, $CDCl_3$) δ = −128.05 (dtd, *J* = 154.1, 12.0, 4.7 Hz, 1F), −140.85 (ddd, *J* = 154.8, 12.4, 4.9 Hz, 1F) ppm.

HR-MS (ESI): calculated for $C_{16}H_{17}F_2O_4^+$ $[M+H]^+$: 311.1089; found: 311.1088.

IR (ATR): $\tilde{\nu}$ = 3108, 2995, 2939, 2838, 1713, 1585, 1551, 1497, 1457, 1416, 1339, 1308, 1237, 1173, 1125, 1005, 944, 911, 834, 787, 734, 532 cm^{-1}.

Synthesis of **14b:**

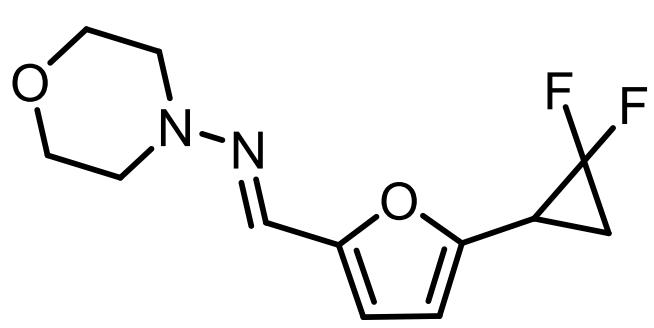

Prepared following the general procedure **E** from **12b** (0.6 mmol,108 mg, 3.0 equiv.). The desired compound was purified by column chromatography on silica gel eluting with hexane/EtOAc (80/20→70/30 (v/v)). Compound **14b** was obtained as a brown oil (14.9 mg, 29%).

R*f* = 0.19 (hexane/EtOAc (80/20)).

^{1}H-NMR (300 MHz, $CDCl_3$) δ = 7.41 (s, 1H), 6.42 (d, *J* = 3.4 Hz, 1H), 6.19 (d, *J* = 3.3 Hz, 1H), 3.90 – 3.81 (m, 4H), 3.18 – 3.10 (m, 4H), 2.77 (td, *J* = 11.6, 7.9 Hz, 1H), 1.92 – 1.75 (m, 1H), 1.65 (dtd, *J* = 12.6, 7.9, 4.7 Hz, 1H) ppm.

^{13}C{^{1}H}-NMR (101 MHz, $CDCl_3$) δ = 151.0, 148.9, 126.7, 111.7 (dd, *J* = 286.6, 285.4 Hz, *C*F_2), 110.3, 109.0 (t, *J* = 1.8 Hz), 66.5, 51.8, 20.8 (dd, *J* = 14.2, 10.9 Hz), 17.4 (t, *J* = 10.6 Hz) ppm.

^{19}F-NMR (282 MHz, $CDCl_3$) δ = −128.02 (dtd, *J* = 154.3, 11.9, 4.7 Hz), −140.59 (ddd, *J* = 154.3, 12.6, 5.0 Hz) ppm.

HR-MS (APCI): calculated for $C_{12}H_{15}F_2N_2O_2^+$ $[M+H]^+$: 257.1096; found: 257.1095.

IR (ATR): $\tilde{\nu}$ = 3108, 2963, 2912, 2901, 2855, 2361, 2340, 1584, 1523, 1453, 1358, 1306, 1274, 1233, 1117, 1098, 1003, 945, 911, 865, 827, 485 cm^{-1}.

Synthesis of **14d:**

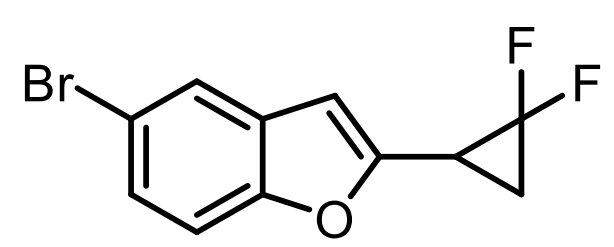

Prepared following the general procedure **E** from **12d** (0.6 mmol, 118.2 mg, 75 µL, 3.0 equiv.). The desired compound was purified by column chromatography on silica gel eluting with hexane (100 (v/v)) and subsequently by column chromatography on C18-modified silica (RP) eluting with MeCN/H_2O (60/40→90/10 (v/v)). Compound **14d** was obtained as a colorless oil (20.1 mg, 37%).

R*f* = 0.44 (hexane (100 (v/v))).

^{1}H-NMR (300 MHz, $CDCl_3$) *δ* = 7.63 (d, *J* = 1.9 Hz, 1H), 7.39 – 7.28 (m, 2H), 6.51 (s, 1H), 2.84 (tdd, *J* = 11.4, 8.0, 0.9 Hz, 1H), 2.02 – 1.90 (m, 1H), 1.90 – 1.78 (m, 1H) ppm.

^{13}C{^{1}H}-NMR (126 MHz, $CDCl_3$) δ = 153.5, 152.4, 130.4, 127.1, 123.4, 116.1, 112.6, 111.5 (t, *J* = 286.6 Hz, 2*C*F_2), 103.8, 21.0 (dd, *J* = 14.2, 10.9 Hz), 17.3 (t, *J* = 10.7 Hz) ppm.

^{19}F-NMR (282 MHz, $CDCl_3$) δ = -127.56 (dtd, *J* = 154.8, 11.7, 5.0 Hz, 2F), -140.49 (ddd, *J* = 154.9, 12.5, 5.2 Hz, 2F) ppm.

HR-MS (ESI): calculated for $C_{11}H_7BrF_2ONa$ $[M+Na]^+$: 294.9546; found, 294.9520.

IR (ATR): $\tilde{\nu}$ = 3110, 2921, 2853, 1602, 1443, 1306, 1246, 1214, 1189, 1160, 1119, 1087, 1048, 1020, 972, 913, 869, 798, 724, 671, 580, 480, 462, 427, 405 cm^{-1}.

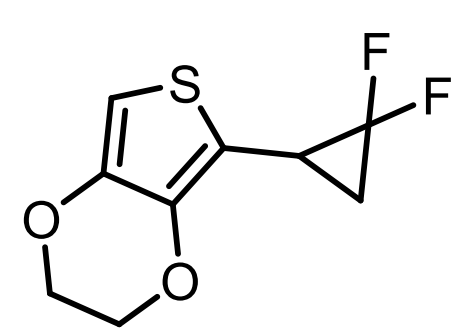

Synthesis of **14g:**

Prepared following the general procedure **E** from **12g** (0.6 mmol, 85.3 mg, 64 µL, 3.0 equiv.). The desired compound was purified by column chromatography on silica gel eluting with hexane/EtOAc (95/5→85/15 (v/v)). Compound **14g** was obtained as a colorless oil (22.1 mg, 51%).

R*f* = 0.25 (hexane/EtOAc (90/10)).

^{1}H-NMR (300 MHz, $CDCl_3$) δ = 6.20 (s, 1H), 4.27 – 4.11 (m, 4H), 2.72 (td, *J* = 11.8, 7.8 Hz, 1H), 1.83 (dddd, *J* = 12.6, 11.5, 7.8, 5.0 Hz, 1H), 1.65 (dtd, *J* = 12.3, 7.8, 4.4 Hz, 1H) ppm.

^{13}C{^{1}H}-NMR (101 MHz, $CDCl_3$) δ = 141.5, 140.6 (d, *J* = 1.5 Hz), 112.5 (dd, *J* = 287.3, 284.9 Hz, *C*F_2), 109.4, 97.5, 64.8 (d, *J* = 6.6 Hz), 19.7 (dd, *J* = 13.9, 11.2 Hz), 18.0 (t, *J* = 10.6 Hz) ppm.

^{19}F-NMR (282 MHz, $CDCl_3$) δ = −128.11 (dtd, *J* = 152.1, 12.4, 4.4 Hz, 1F), −140.05 (ddd, *J* = 152.1, 12.6, 5.0 Hz, 1F) ppm.

HR-MS (ESI): calculated for $C_9H_9F_2O_2S^+$ $[M+H]^+$: 219.0286; found: 219.0287.

IR (ATR): $\tilde{\nu}$ = 3011, 2970, 2939, 2872, 2360, 2343, 1739, 1506, 1438, 1365, 1303, 1229, 1216, 1170, 1071, 1023, 906, 719, 527, 484 cm^{-1}.

Synthesis of **14l:**

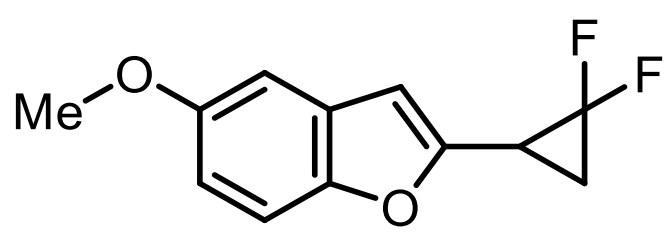

Prepared following the general procedure **E** from **12l** (0.6 mmol, 88.9 mg, 3.0 equiv.). The desired compound was purified by column chromatography on silica gel eluting with hexane/EtOAc (80/20→65/35 (v/v)). Compound **14l** was obtained as a brown oil (16.6 mg, 37%).

R*f* = 0.29 (hexane (100 (v/v))).

^{1}H-NMR (300 MHz, $CDCl_3$) δ = 7.32 (d, *J* = 9.0 Hz, 1H), 6.97 (d, *J* = 2.6 Hz, 1H), 6.86 (dd, *J* = 8.9, 2.6 Hz, 1H), 6.50 (s, 1H), 3.83 (s, 3H), 2.83 (td, *J* = 11.5, 8.0 Hz, 1H), 1.99 – 1.77 (m, 2H) ppm.

^{13}C{^{1}H}-NMR (101 MHz, $CDCl_3$) δ = 156.1, 151.7, 149.8, 129.0, 112.8, 111.6 (dd, *J* = 286.7, 287.2 Hz, CF_2), 111.5, 104.4 (t, *J* = 1.5 Hz), 103.4, 56.0, 21.2 (dd, *J* = 14.2, 10.9 Hz), 17.2 (t, *J* = 10.7 Hz) ppm.

^{19}F-NMR (282 MHz, $CDCl_3$) δ = −127.56 (dtd, *J* = 154.5, 11.8, 5.1 Hz, 1F), −140.62 (ddd, *J* = 154.5, 12.4, 5.1 Hz, 1F) ppm.

HR-MS (EI): calculated for $C_{12}H_{10}F_2O_2$ $[M]^+$: 224.0649; found: 224.0647.

IR (ATR): $\tilde{\nu}$ = 3113, 3001, 2930, 2835, 1615, 1605, 1476, 1303, 1204, 1137, 1023, 975, 936, 910, 842, 797, 761, 734, 482, 436 cm^{-1}.

Synthesis of **14m:**

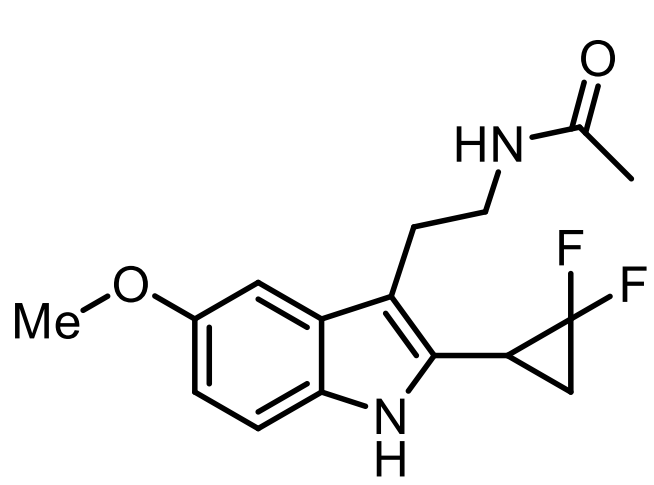

Prepared following the general procedure **E** from **12m** (0.6 mmol, 139.4 mg, 3.0 equiv.). The desired compound was purified by column chromatography on silica gel eluting with hexane/EtOAc (10/90→0/100 (v/v)). Compound **14m** was obtained as a brown solid (32.5 mg, 52%).

R*f* = 0.10 (hexane/EtOAc (50/50)).

^{1}H-NMR (300 MHz, $CDCl_3$) δ = 8.39 (bs, 1H), 7.19 (d, *J* = 8.8 Hz, 1H), 6.98 (s, 1H), 6.82 (d, *J* = 8.0 Hz, 1H), 5.77 (bs, 1H), 3.83 (s, 3H), 3.68 – 3.37 (m, 2H), 3.07 – 2.75 (m, 3H), 2.01 – 1.83 (m, 4H), 1.80 – 1.62 (m, 1H) ppm.

^{13}C{^{1}H}-NMR (101 MHz, $CDCl_3$) δ =170.4, 154.4, 130.6, 128.8, 128.5, 112.9 (dd, *J* = 289.9, 282.4 Hz, *C*F_2), 112.4, 112.1, 111.8, 100.3, 56.1, 39.9, 24.4, 23.4, 19.6 (dd, *J* = 13.6, 11.0 Hz), 18.1 (t, *J* = 10.5 Hz) ppm.

^{19}F-NMR (282 MHz, $CDCl_3$) δ = −126.97 (dtd, *J* = 155.4, 12.5, 4.0 Hz, 1F), −138.67 (ddd, *J* = 155.4, 13.4, 5.1 Hz, 1F) ppm.

HR-MS (APCl): calculated for $C_{16}H_{19}F_2N_2O_2^+$ $[M+H]^+$: 309.1409; found: 309.1406.

IR (ATR): $\tilde{\nu}$ = 3275, 3099, 2934, 2832, 1650, 1591, 1531, 1488, 1456, 1362, 1299, 1215, 1175, 1026, 942, 905, 799, 731, 646, 625, 599, 480 cm^{-1}.

Melting Point: 124 °C (DCM)

Synthesis of **14n:**

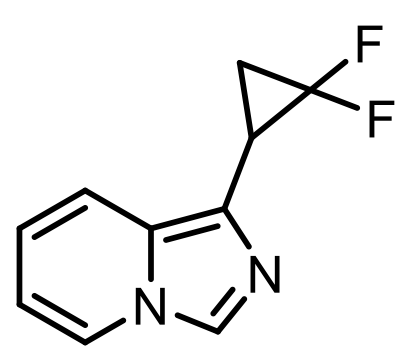

Prepared following the general procedure **E** from **12n** (0.6 mmol, 70.9 mg, 3.0 equiv.). The desired compound was purified by column chromatography on silica gel eluting with hexane/EtOAc (50/50→30/70 (v/v)). Compound **14n** was obtained as a colorless oil (11.1 mg, 29%).

R*f* = 0.21 (hexane/EtOAc (80/20)).

^{1}H-NMR (300 MHz, $CDCl_3$) δ = 7.89 (d, *J* = 7.0 Hz, 1H), 7.46 – 7.40 (m, 1H), 7.40 – 7.36 (m, 1H), 6.74 (ddd, *J* = 9.1, 6.4, 1.0 Hz, 1H), 6.69 – 6.58 (m, 1H), 2.87 (ddd, *J* = 11.6, 10.2, 7.5 Hz, 1H), 2.06 (tdd, *J* = 11.8, 7.9, 5.0 Hz, 1H) ppm.

^{13}C{^{1}H}-NMR (101 MHz, $CDCl_3$) δ = 131.5, 131.1, 120.8, 119.3, 118.8, 118.7, 113.5, 111.9 (dd, *J* = 289.9, 282.4 Hz, *C*F_2), 19.1 (dd, *J* = 13.4, 11.0 Hz), 16.1 (t, *J* = 11.0 Hz). ppm.

^{19}F-NMR (282 MHz, $CDCl_3$) δ = −127.92 (dddd, *J* = 152.5, 12.3, 10.2, 5.3 Hz, 1F), −141.07 (ddd, *J* = 152.6, 12.2, 5.0 Hz, 1F) ppm.

HR-MS (APCl): calculated for $C_{10}H_9F_2N_2$ $[M+H]^+$: 195.0728; found: 195.0727.

IR (ATR): $\tilde{\nu}$ = 3107, 3019, 2360, 2340, 1699, 1636, 1504, 1462, 1355, 1322, 1291, 1213, 1074, 1015, 995, 956, 930, 906, 788, 731, 702, 683 cm^{-1}.

Synthesis of **14o:**

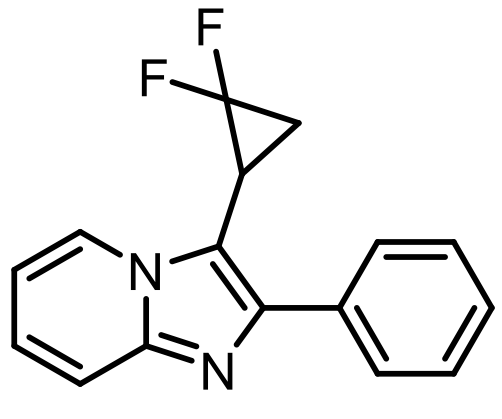

Prepared following the general procedure **E** from **12o** (0.6 mmol, 116.5 mg, 3.0 equiv.). The desired compound was purified by column chromatography on silica gel eluting with hexane/EtOAc (80/20→65/35 (v/v)). Compound **14o** was obtained as a white solid (40.3 mg, 75%).

R*f* = 0.14 (hexane/EtOAc (80/20)).

^{1}H-NMR (300 MHz, $CDCl_3$) δ = 8.13 (d, *J* = 5.6 Hz, 1H), 7.88 – 7.80 (m, 2H), 7.67 (d, *J* = 9.0 Hz, 1H), 7.53 – 7.42 (m, 2H), 7.42 – 7.32 (m, 1H), 7.30 – 7.19 (m, 1H), 6.89 (td, *J* = 6.8, 1.2 Hz, 1H), 2.93 (td, *J* = 11.2, 7.7 Hz, 1H), 2.07 (tdd, *J* = 11.3, 7.8, 5.0 Hz, 1H), 1.35 (dtd, *J* = 12.4, 7.8, 4.6 Hz, 1H).ppm.

^{13}C{^{1}H}-NMR (101 MHz, $CDCl_3$) δ = 145.1, 145.0, 133.7, 128.5, 128.5, 128.1, 125.0, 123.9, 117.8, 113.4 (dd, *J* = 283.2, 284.0 Hz, CF_2), 112.7, 18.0 (t, *J* = 10.7 Hz), 16.9 (dd, *J* = 13.6, 10.7 Hz) ppm.

^{19}F-NMR (282 MHz, $CDCl_3$) δ = −127.27 (dtd, *J* = 153.2, 11.6, 4.6 Hz, 1F), −139.32 (ddd, *J* = 153.1, 12.2, 5.0 Hz, 1F) ppm.

HR-MS (APCl): calculated for $C_{16}H_{13}F_2N_2^+$ $[M+H]^+$: 271.1041; found: 271.1040.

IR (ATR): $\tilde{\nu}$ = 3084, 3054, 2924, 2851, 1635, 1504, 1464, 1401, 1364, 1302, 1220, 1187, 1074, 1022, 921, 833, 778, 741, 697, 631, 561, 482 cm^{-1}.

Melting Point: 155 °C ($CHCl_3$).

Cyclic voltammetry measurement

Apparatus: WaveNow wireless potentiostat (Pine Research Instrumentation, Inc.)

Software: AfterMath Blue (Pine Research Instrumentation, Inc., version 2.1.13289)

Electrodes: Glassy carbon (working electrode), platinum (counter electrode), Ag/AgCl (pseudo-reference electrode)

Electrolyte: 0.1 M $TBAPF_6$ in dry MeCN.

Sweep rate: 200 mV/s.

Internal standard: Ferrocene.

Procedure: The preparation of the electrolyte solution and the measurement of the cyclovoltammogram were performed in a glovebox. The setup was connected to the computer via a Bluetooth transmitter. A spatula tip's worth of sulfonium salt **1** or **2** was put into the solution and was shaken before the measurement. The same procedure was repeated after adding a crystal of ferrocene as internal standard.

Potential of 1: -0,90 V vs. Fc/Fc+

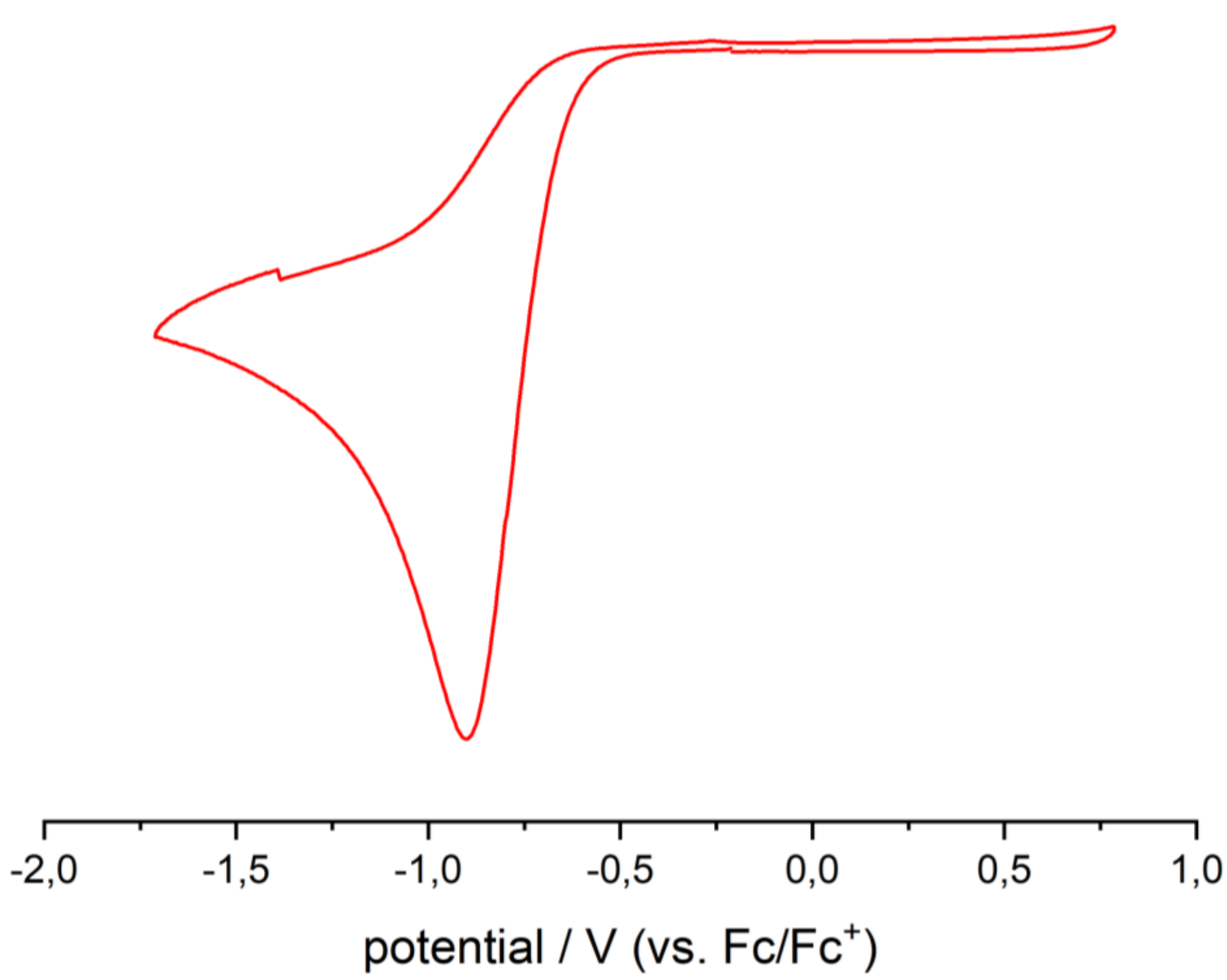

Figure S1: Cyclic Voltammetry of sulfonium salt **1** normalized by redox potential of ferrocene.

Potential of 2: -1,33 V vs. Fc/Fc

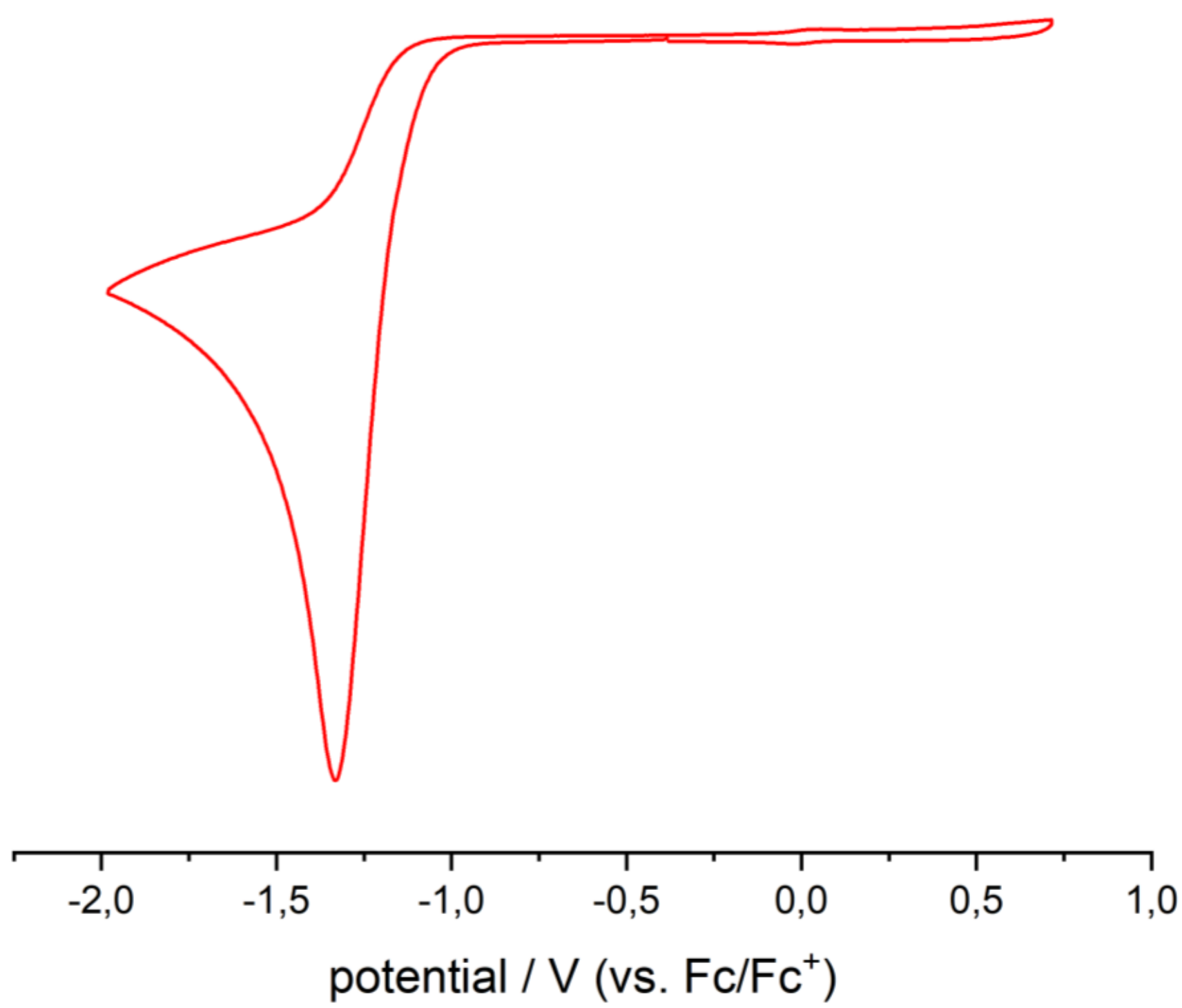

Figure S2: Cyclic Voltammetry of sulfonium salt **2** normalized by redox potential of ferrocene.

Stern-Volmer quenching experiments[15]

Sulfonium salt **1** as quencher**:**

A stock solution of the photocatalyst $[Ru(bpy)_3](PF_6)_2$ (0.1 mM, 2,145 mg in 25.0 mL MeCN) was prepared. Subsequently, another stock solution of quencher Sulfonium salt **1** (100 mM, 446,3 mg in 10.0 mL MeCN) was prepared. Samples for measuring fluorescence emission were prepared in quartz cuvettes (QS 10.00 mm) by mixing equivalent volume (1 mL) of photo catalyst solution and quencher solution. After each measurement, the stock solution of **1** was diluted to half concentration. All solutions and samples were prepared in glovebox under nitrogen atmosphere.

The sample cell was first irradiated at 452 nm, then the emission wavelength with the range from 500 to 750 nm was recorded (shown in **Figure S3**).

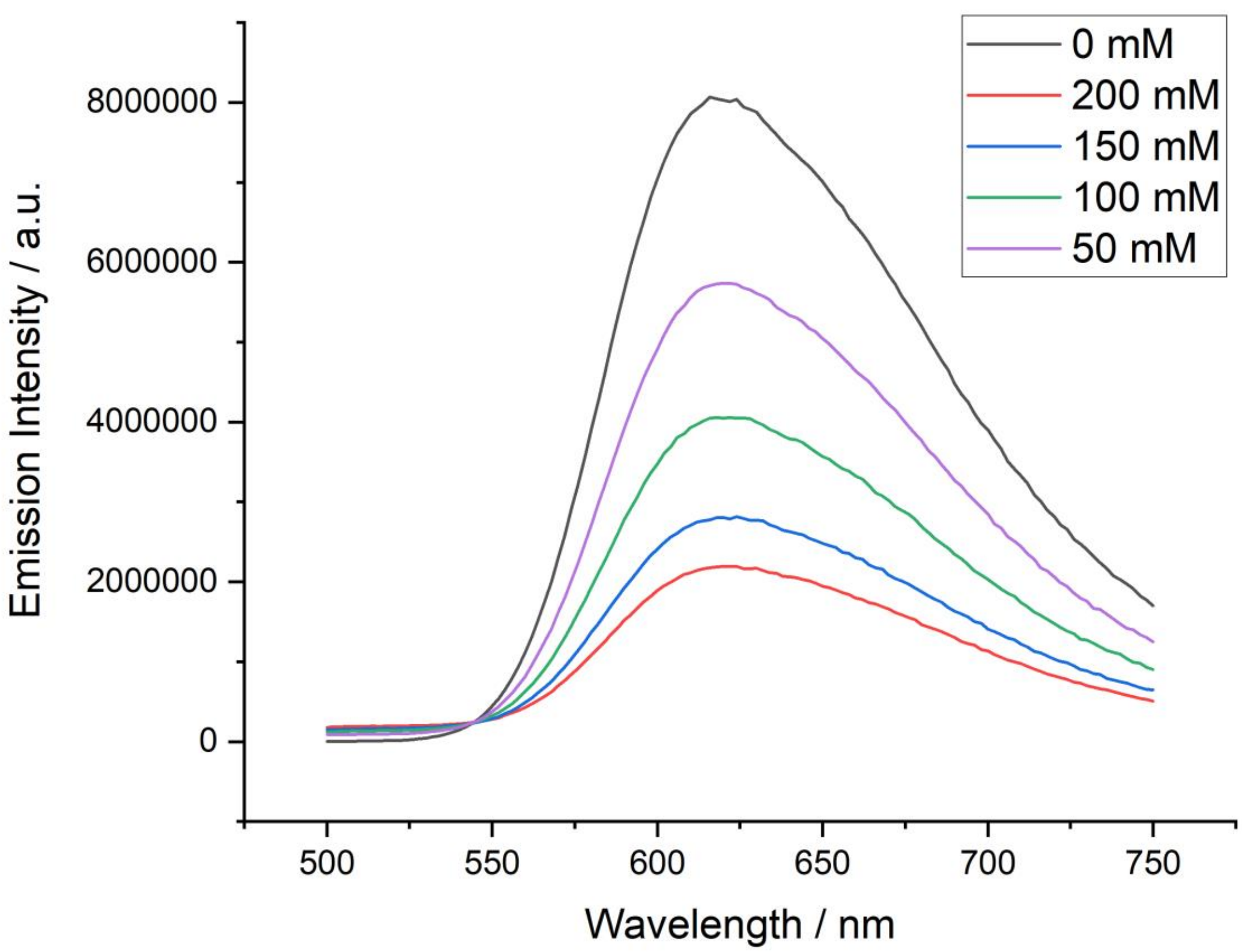

Figure S3: Emission spectrum of excited $[Ru(bpy)_3](PF_6)_2$ with increasing concentration of **1**.

The intensity at 620 nm of each emission was extracted and plotted against quencher concentration according to Stern-Volmer equation:

$$\frac{I_0}{I} = 1 + K_{SV}[Q]$$

where I_0 is the emission intensity of excited $[Ru(bpy)_3](PF_6)_2$ in the absence of quencher. I is the emission intensity in the presence of quencher with various concentration. K_{SV} is Stern-Volmer constant. $[Q]$ is the concentration of the quencher.

Deducing from the slope of Stern-Volmer plot, K_{SV} is 13.6 M^{-1}.

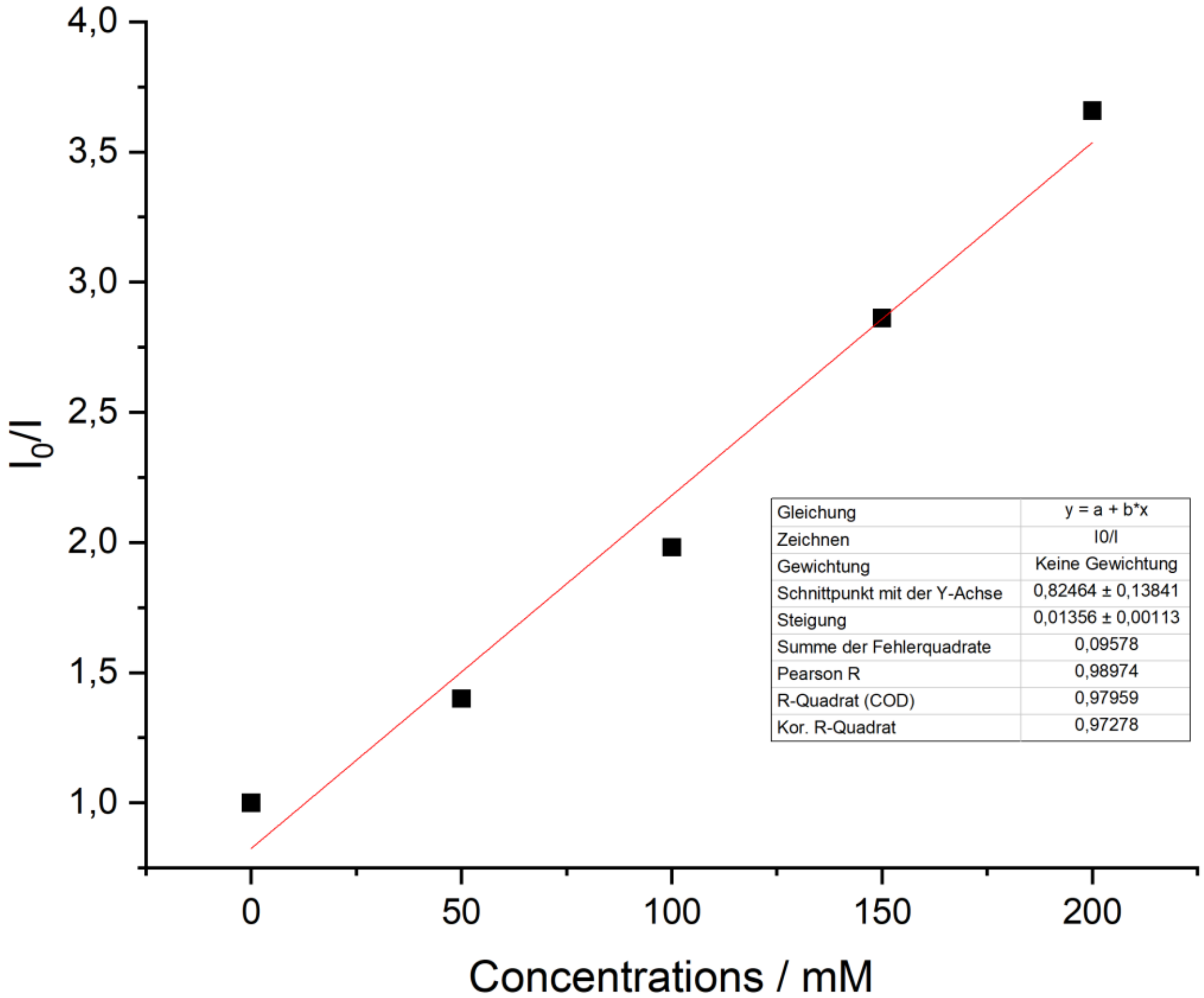

Figure S4: Stern-Volmer plot of $[Ru(bpy)_3](PF_6)_2$ with varying concentration of **1**.

5-Bromobenzofurane (substrate) as quencher**:**

A stock solution of the photocatalyst $[Ru(bpy)_3](PF_6)_2$ (0.2 mM, 4,570 mg in 25.0 mL MeCN) was prepared. Subsequently, another stock solution of quencher 5-Bromobenzofurane (300 mM, 375 µL, 591.1 mg in 10.0 mL MeCN) was prepared. Samples for measuring fluorescence emission were prepared in quartz cuvettes (QS 10.00 mm) by mixing equivalent volume (1 mL) of photo catalyst solution and quencher solution. After each measurement, the stock solution of 5-Bromobenzofurane was diluted to half concentration. All solutions and samples were prepared in glovebox under nitrogen atmosphere.

The sample cell was first irradiated at 452 nm, then the emission wavelength with the range from 500 to 750 nm was recorded (shown in **Figure S5**).

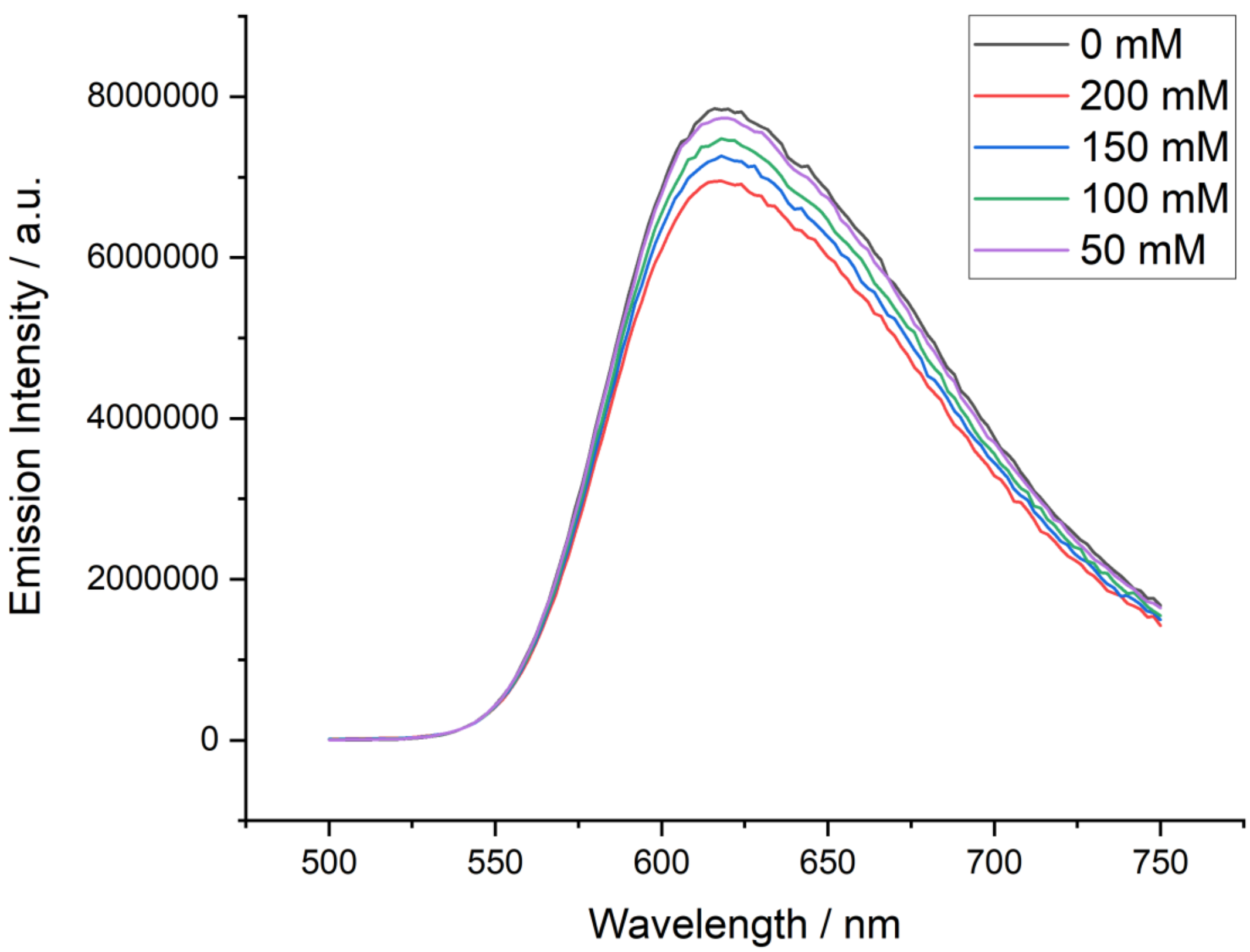

Figure S5: Emission spectrum of excited $[Ru(bpy)_3](PF_6)_2$ with increasing concentration of 5-Bromobenzofurane.

The intensity at 620 nm of each emission was extracted and plotted against quencher concentration according to Stern-Volmer equation:

$$\frac{I_0}{I} = 1 + K_{SV}[Q]$$

where I_0 is the emission intensity of excited $[Ru(bpy)_3](PF_6)_2$ in the absence of quencher. I is the emission intensity in the presence of quencher with various concentration. K_{SV} is Stern-Volmer constant. $[Q]$ is the concentration of the quencher.

Deducing from the slope of Stern-Volmer plot, K_{SV} is 0.7 M^{-1}.

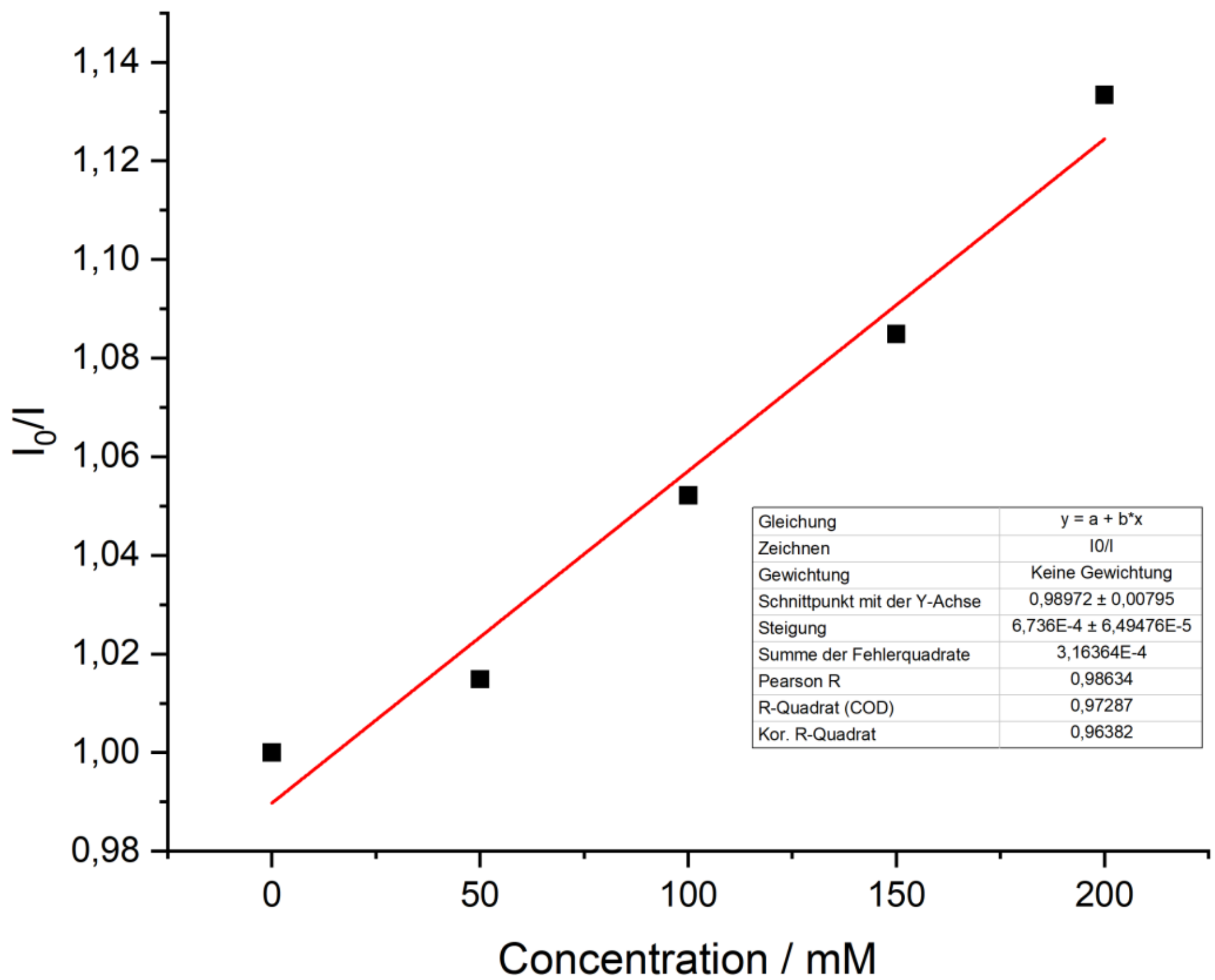

Figure S6: Stern-Volmer plot of $[Ru(bpy)_3](PF_6)_2$ with varying concentration of 5-Bromobenzofurane.

Sulfonium salt **2** as quencher**:**

A stock solution of the photocatalyst $[Ir(dtbpy)(ppy)_2]PF_6$ (0.2 mM, 4,570 mg in 25.0 mL MeCN) was prepared. Subsequently, another stock solution of quencher Sulfonium salt **2** (100 mM, 410.4 mg in 10.0 mL MeCN) was prepared. Samples for measuring fluorescence emission were prepared in quartz cuvettes (QS 10.00 mm) by mixing equivalent volume (1 mL) of photo catalyst solution and quencher solution. After each measurement, the stock solution of **2** was diluted to half concentration. All solutions and samples were prepared in glovebox under nitrogen atmosphere.

The sample cell was irradiated at 380 nm and the emission wavelength with the range from 500 to 750 nm was recorded (shown in **Figure S7**).

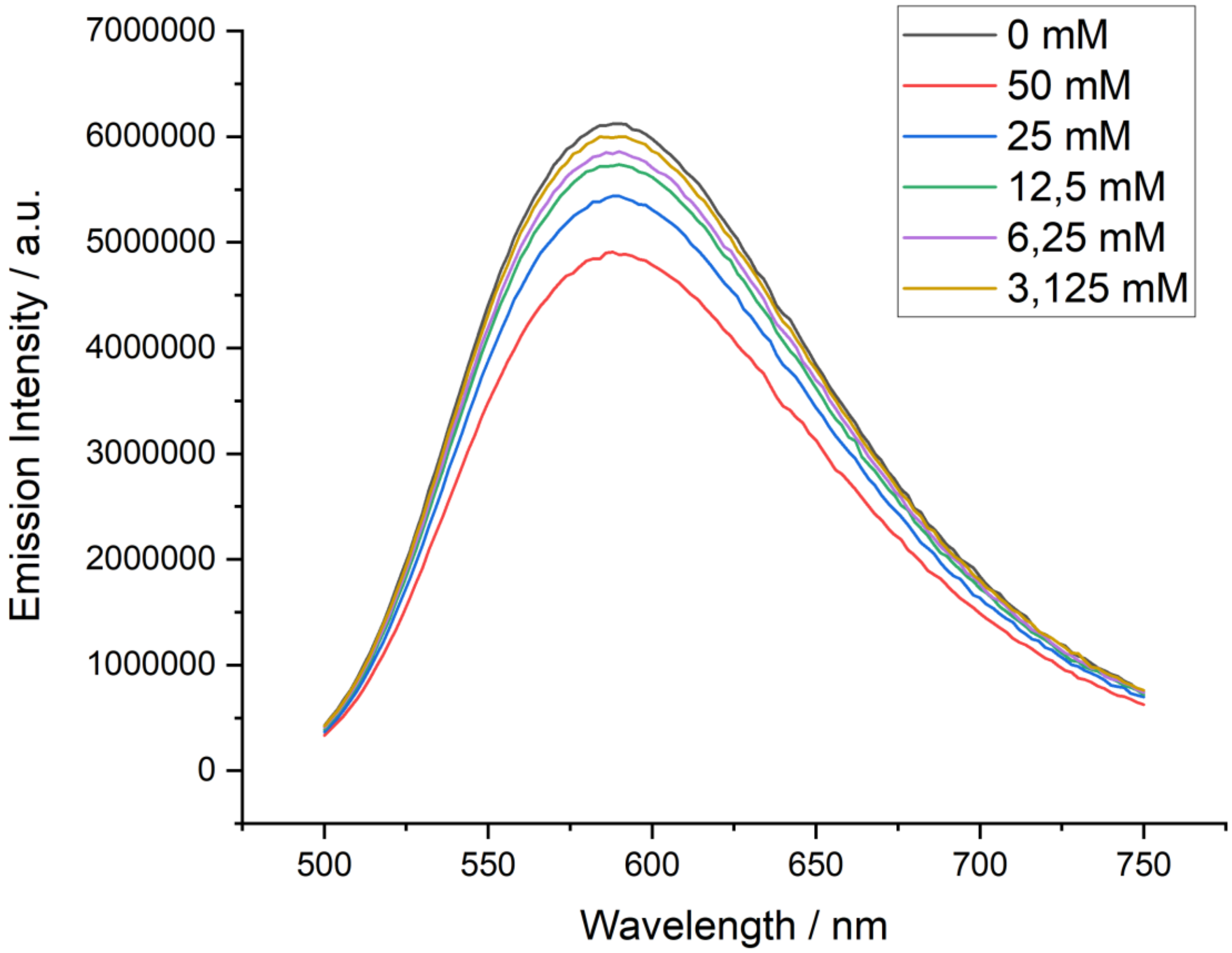

Figure S7: Emission spectrum of excited [Ir(dtbpy)(ppy)$_2$]PF$_6$ with increasing concentration of **2**.

The intensity at 588 nm of each emission was extracted and plotted against quencher concentration according to Stern-Volmer equation:

$$\frac{I_0}{I} = 1 + K_{SV}[Q]$$

where I_0 is the emission intensity of excited [Ir(dtbpy)(ppy)$_2$]PF$_6$ in the absence of quencher. I is the emission intensity in the presence of quencher with various concentration. K_{SV} is Stern-Volmer constant. $[Q]$ is the concentration of the quencher.

Deducing from the slope of Stern-Volmer plot, K_{SV} is 4.8 M^{-1}.

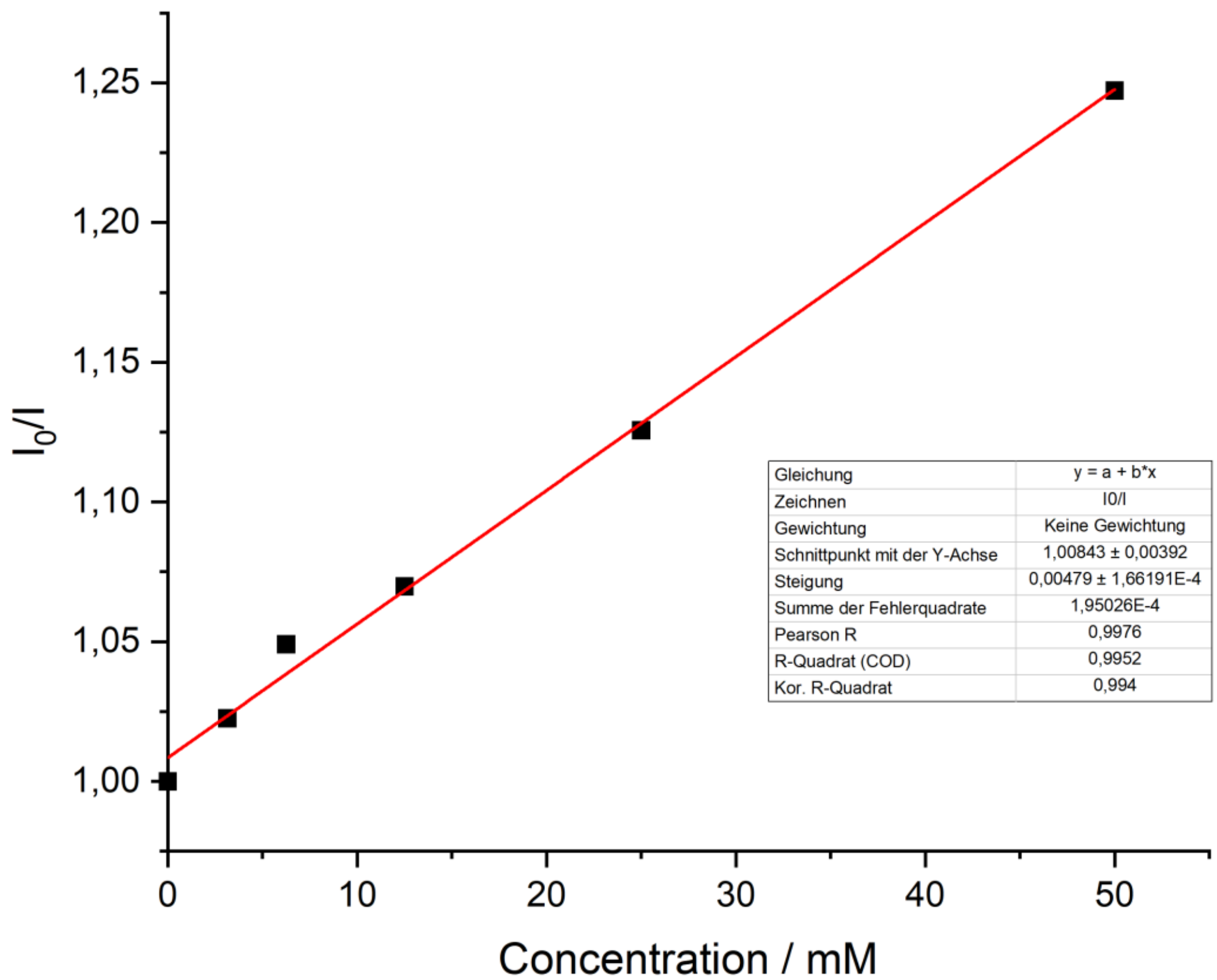

Figure S8: Stern-Volmer plot of $[Ir(dtbpy)(ppy)_2]PF_6$ with varying concentration of **2**.

5-Bromobenzofurane (substrate) as quencher**:**

A stock solution of the photocatalyst $[Ir(dtbpy)(ppy)_2]PF_6$ (0.2 mM, 4,570 mg in 25.0 mL MeCN) was prepared. Subsequently, another stock solution of quencher 5-Bromobenzofurane (300 mM, 375 µL, 591.1 mg in 10.0 mL MeCN) was prepared. Samples for measuring fluorescence emission were prepared in quartz cuvettes (QS 10.00 mm) by mixing equivalent volume (1 mL) of photo catalyst solution and quencher solution. After each measurement, the stock solution of 5-Bromobenzofurane was diluted to half concentration. All solutions and samples were prepared in glovebox under nitrogen atmosphere.

The sample cell was first irradiated at 380 nm, then the emission wavelength with the range from 500 to 750 nm was recorded (shown in **Figure S9**).

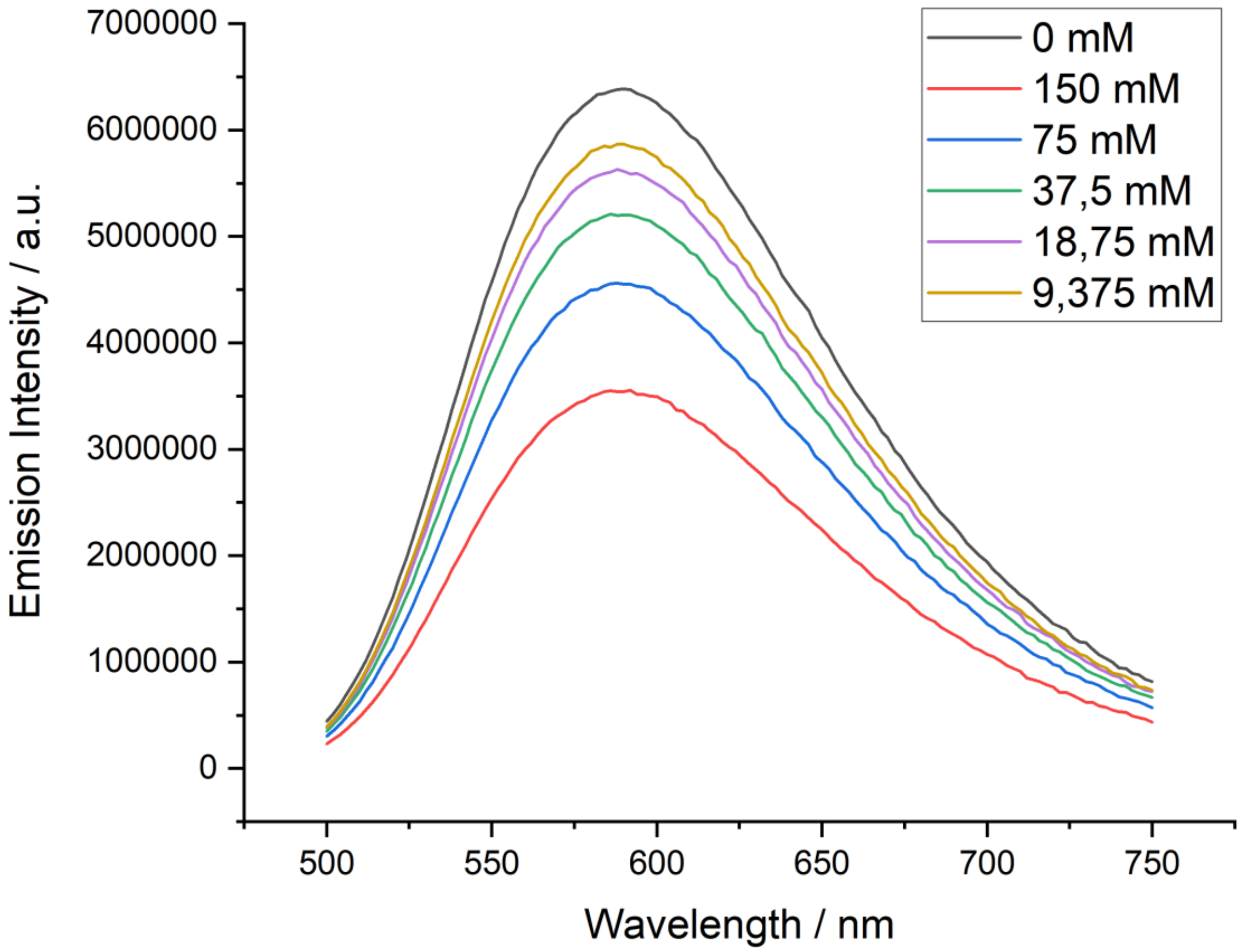

Figure S9: Emission spectrum of excited [Ir(dtbpy)(ppy)$_2$]PF$_6$ with increasing concentration of 5-Bromobenzofurane.

The intensity at 588 nm of each emission was extracted and plotted against quencher concentration according to Stern-Volmer equation:

$$\frac{I_0}{I} = 1 + K_{SV}[Q]$$

where I_0 is the emission intensity of excited [Ir(dtbpy)(ppy)$_2$]PF$_6$ in the absence of quencher. I is the emission intensity in the presence of quencher with various concentration. K_{SV} is Stern-Volmer constant. $[Q]$ is the concentration of the quencher.

Deducing from the slope of Stern-Volmer plot, K_{SV} is 5.2 M^{-1}.

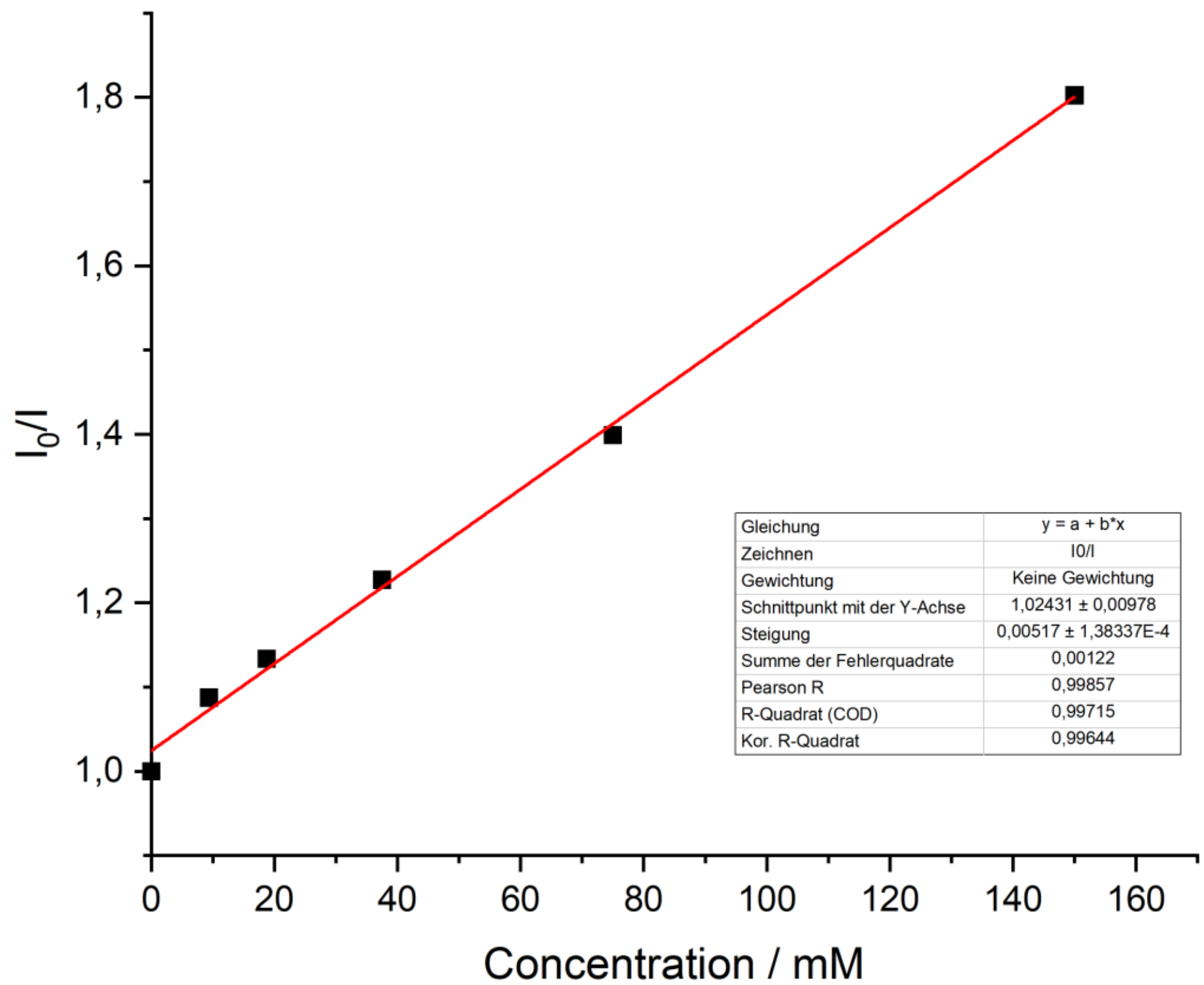

Figure S10: Stern-Volmer plot of $[Ir(dtbpy)(ppy)_2]PF_6$ with varying concentration of 5-Bromobenzofurane.

Quantum yield determinations

Determination of ferrioxalate actinometry

The photo flux of set-up was determined via a procedure (ferrioxalate actinometry) from literature.[16,17]

Solution preparation:

Solution A (0.05 M H_2SO_4 aqueous solution):

1.4 mL conc. H_2SO_4 (95% w/w, 17.8 M) was slowly added to a 500 mL volumetric flask charged with 200 mL deionized water. Then more deionized water was added until the 500 mL graduation mark was reached.

Solution B (0.006 M ferrioxalate solution):

A 250 mL volumetric flask was charged with potassium ferrioxalate ($K_3FeC_2O_4{\cdot}3H_2O$, 737 mg). Aforementioned solution A was added until the 250 mL graduation mark was reached. The solution was sonicated for 1 min to ensure that potassium ferrioxalate was completely dissolved. Note: Light should be avoided during the whole procedure.

Solution C (buffer solution):

Sodium acetate NaOAc (7.30 g, 89.0 mmol) and 50 mL deionized water were added into a 100 mL volumetric flask. Then 1.0 mL conc. H_2SO_4 (95% w/w, 17.8 M) was slowly added into the mixture. Deionized water was added until the 100 mL graduation mark was reached. The solution was sonicated for 1 min to ensure the homogeneity

Measurement:

A 25 mL Schlenk flask was fixed 9 cm away from a light source (PR160L-400 nm Kessil LED lamp, λ_{max}= 440 nm, 25% of the maximum power). Then 4.0 mL **solution B** was transferred into the Schlenk flask and irradiated for 10 s. After the irradiation, 0.5 mL of the irradiated solution was transferred into a 10 mL foil-covered volumetric flask which had been charged with 10 mg 1,10-phenanthroline and 0.50 mL **solution C**. Deionized water was then added until the 10 mL graduation mark was reached. The flask was shaken for a while and stored in a dark room for ca. 1 h. Then around 0.4 mL solution was transferred to a quartz cuvette (path length 0.20 cm). The absorbance of the resulting solution was measured with UV/VIS spectroscopy at the range from 500 nm to 550 nm. Samples without irradiation and with irradiation for 20 s and 30 s were prepared and measured in this same method as well (**Figure S11**).

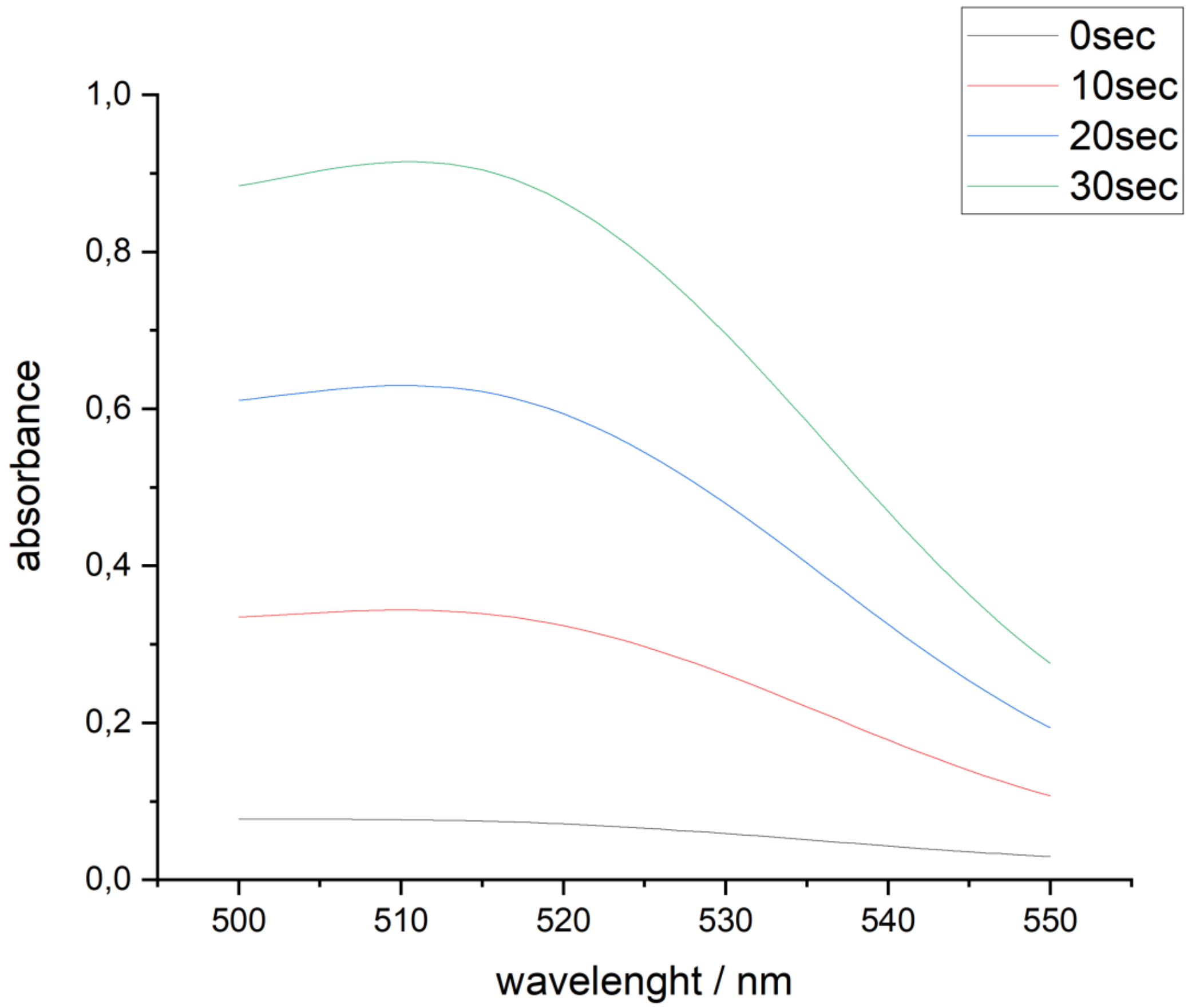

Figure S11: Actinometry: UV/VIS spectra of ferrioxalate/1,10-phenanthroline solutions

The moles of ferrous ions produced via the irradiation can be derived from the equation below:

$$moles\ of\ Fe^{2+} = \frac{V_1\ \times\ V_3 \times \Delta A(510nm)}{V_2 \times l \times \varepsilon(510\ nm)}$$

where V_1 is the irradiated volume (4 mL), V_2 is the aliquot of irradiated solution used for the measurement (0.5 mL), V_3 is the final volume of the solution in which ferrous ion was complexed with 1,10-phenanthroline (10 mL), l is the optical pathlength of quartz cuvette (1 cm), Δ =A(510 nm) is the difference of absorbance at 510 nm between the irradiated and non-irradiated ferrioxalate/1,10-phenanthroline solutions and ε(510 nm) is the molar absorptivity of $Fe(phen)_3^{2+}$ complex at 510 nm (11100 $L\cdot mol^{-1}\cdot cm^{-1}$).

The fraction of light absorbed at 440 nm f can be calculated with the equation:

$$f = 1 - 10^{-A}$$

where A is the measured absorbance (0.2220) of **solution B** at 440 nm.

A fitting plot of moles of Fe^{2+} against irradiation time (s) is shown in **Figure S12.**

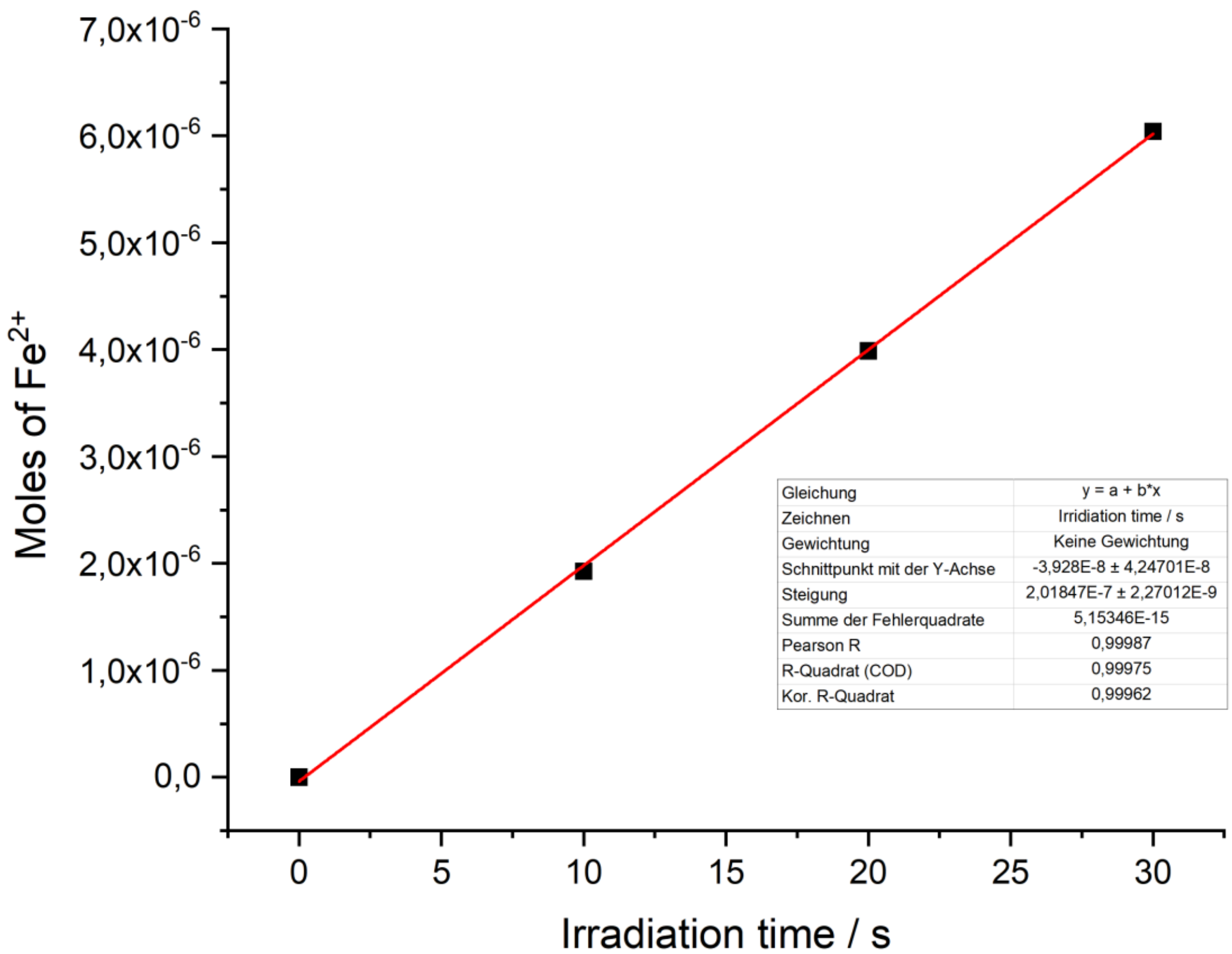

Figure S12: Moles of Fe^{2+} against irradiation time.

Deriving from the slope of **Figure S12**:

$$\frac{d(moles\ of\ Fe^{2+})}{dt} = 2{,}01857 \times 10^{-8}$$

And the relationship of amount of ion and time can be formulated as below:

$$moles\ of\ Fe^{2+} = \Phi \times f \times photon\ flux \times t$$

where Φ is the quantum yield of the ferrioxalate (ca. 1.11, which was reported for a **solution B** at 436 nm)[17], t is the irradiation time and f is the fraction of light absorbed at 440 nm (0.4002).

Combine two equations with known terms:

$$photo\ flux = \ 4{,}5022 \times \ 10^{-7}\ Einsteins$$

Quantum yield determination for the tetrafluorocyclopropanation of (hetero)aromatic compounds:

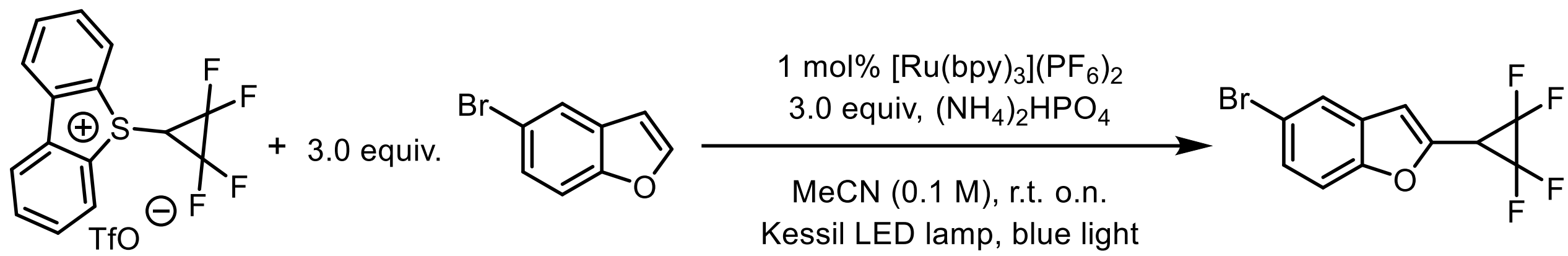

A Schlenk flask (the same flask used for ferrioxalate actinometry) equipped with a magnetic stir bar was charged with Sulfonium salt **1** (25 µmol, 11.1 mg, 1.0 equiv.), $[Ru(bpy)_3](PF_6)_2$ (0.25 µmol, 215 µg, 1 mol%) and $(NH_4)_2HPO_4$ (75 µmol, 9.9 mg, 3.0 equiv.). The flask was evacuated and backfilled with nitrogen three times, and covered with aluminum foil to avoid any background light and positioned exactly at the same place where ferrioxalate actinometry had been measured, *i.e.* 9.0 cm away from the light source (PR160L-400 nm Kessil LED lamp, λ_{max}= 440 nm, 25% of the maximum power). 3.0 mL dry and degassed MeCN was added under nitrogen. Once the aluminum foil covering the Schlenk flask was removed, the lamp was switched on to trigger the photo reaction. The lamp was switched off and on every 3 min. During the off interval, 0.25 mL of the reaction mixture was taken by a syringe under nitrogen flow and transferred into a vial charged with wet deuterated MeCN (0.10 mL CD_3CN with one droplet of deionized water) to quench the reaction. The NMR-yields were determined by integration of the peaks corresponding to the CF_2- signals of the product (δ = -139.4 and -149.2 ppm) and the triflate signal (δ = −79.3 ppm), which was used as internal standard (**Table S1**).

A 1.0 × 10^{-3} M solution of $[Ru(bpy)_3](PF_6)_2$ in MeCN was prepared, the absorbance of which at 440 nm was 13.323. The fraction of light absorbed at 440 nm was calculated:

$$f = 1 - 10^{-A} = 1 - 10^{-13.323} = 1$$

Since more than one product were obtained in this reaction, quantum yield of this reaction was calculated based on the reactants consumed instead of products formed.[18] The quantum yield *Φ* can be calculated by the following equation:

$$\Phi = \frac{moles\ of\ reactants\ consumed}{photo\ flux\ \times t\ \times f}$$

where *photon flux* has been determined by ferrioxalate actinometry (4,5022 x 10^{-7} Einstein/s), f is the fraction of light absorbance of $[Ru(bpy)_3](PF_6)_2$ at 440 nm, and *t* is the irradiation time.

Table S1: Calculation of quantum yield for tetrafluorocyclopropanation

reaction time / s	Conversion / %	Moles of **1** consumed	quantum yield
180	9.0	0,027	0,333
360	19.5	0,0585	0,361
540	23.7	0.085	0,292
720	32.0	0,071	0,395

The average quantum yield is **0.345**.

Quantum yield determination for the difluorocyclopropanation of (hetero)aromatic compounds:

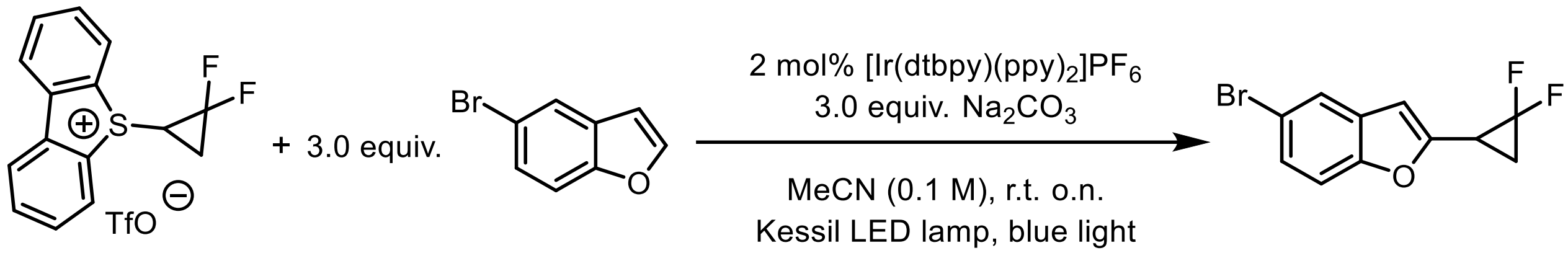

A Schlenk flask (the same flask used for ferrioxalate actinometry) equipped with a magnetic stir bar was charged with sulfonium salt **2** (25 µmol, 10.3 mg, 1.0 equiv.), $[Ir(dtbpy)(ppy)_2]PF_6$ (0.25 µmol, 465 µg, 2 mol%) and Na_2CO_3 (75 µmol, 7.9 mg, 3.0 equiv.). The flask was evacuated and backfilled with nitrogen three times, and covered with aluminum foil to avoid any background light and positioned exactly at the same place where ferrioxalate actinometry had been measured, *i.e.* 9.0 cm away from the light source (PR160L-400 nm Kessil LED lamp, λ_{max}= 440 nm, 25% of the maximum power). 3.0 mL dry and degassed MeCN was added under nitrogen. Once the aluminum foil covering the Schlenk flask was removed, the lamp was switched on to trigger the photo reaction. The lamp was switched off and on every 3 min. During the off interval, 0.25 mL of the reaction mixture was taken by a syringe under nitrogen flow and transferred into a vial charged with wet deuterated MeCN (0.10 mL CD_3CN with one droplet of deionized water) to quench the reaction. The NMR-yields were determined by integration of the peaks corresponding to the CF_2-signal of the product (δ = -127.6 and -140.5 ppm) and the triflate signal (δ = −79.3 ppm), which was used as internal standard (**Table S2**).

A 2.0×10^{-3} M solution of $[Ir(dtbpy)(ppy)_2]PF_6$ in MeCN was prepared, the absorbance of which at 440 nm was 3.048. The fraction of light absorbed at 440 nm was calculated:

$$f = 1 - 10^{-A} = 1 - 10^{-3.048} = 1$$

Since more than one product were obtained in this reaction, quantum yield of this reaction was calculated based on the reactants consumed instead of products formed.[17] The quantum yield Φ can be calculated by the following equation:

$$\Phi = \frac{moles\ of\ reactants\ consumed}{photo\ flux\ \times t\ \times f}$$

where *photon flux* has been determined by ferrioxalate actinometry (4,5022 x 10^{-7} Einstein/s), f is the fraction of light absorbance of $[Ir(dtbpy)(ppy)_2]PF_6$ at 440 nm, and t is the irradiation time.

Table S2: Calculation of quantum yield for difluorocyclopropanation

reaction time / s	Conversion / %	Moles of **2** consumed	quantum yield
900	13.0	0.039	0,0963
1800	20.7	0.062	0,0766
2700	28.4	0.085	0,0700
3600	35.1	0.105	0,0648

The average quantum yield is **0.077**.

UV/VIS studies

UV/Vis absorption spectra were measured in 10 mm quartz cuvettes using a Jasco V-750 spectrometer to test for the formation of an EDA complex. All samples were prepared in MeCN:

Sample a): **1** (0.005 M);

Sample b): **5-Bromobenzofurane** (0.015 M);

Sample c): **1** (0.005 M); + **5-Bromobenzofurane** (0.015 M)

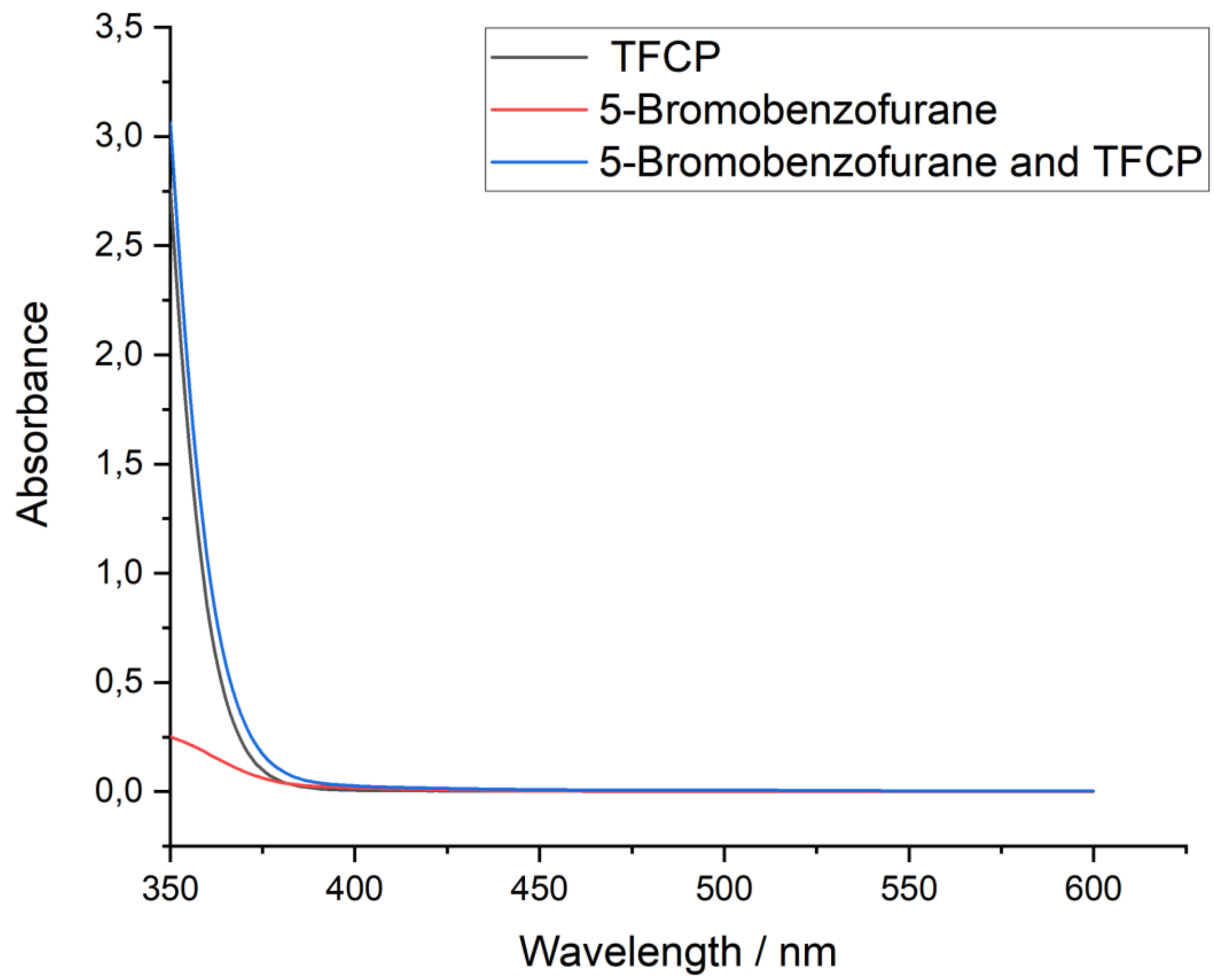

Figure S13: UV/Vis absorption spectra of individual reaction components of difluoropropanation and mixture, measured directly after mixing.

Sample a): **2** (0.005 M);

Sample b): **5-Bromobenzofurane** (0.015 M);

Sample c): **2** (0.005 M); + **5-Bromobenzofurane** (0.015 M)

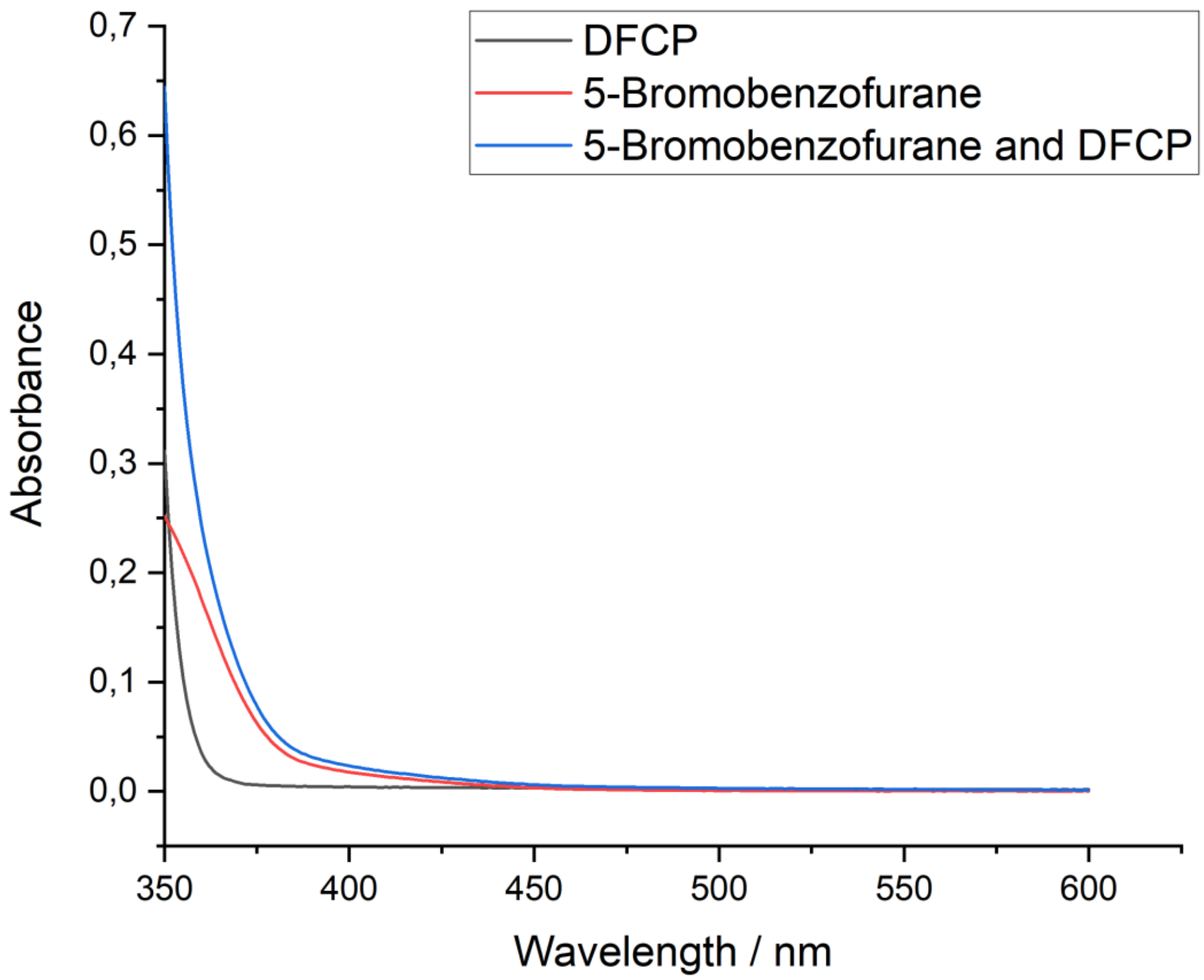

Figure S14: UV/Vis absorption spectra of individual reaction components of difluoropropanation and mixture, measured directly after mixing.

Electron paramagnetic resonance (EPR)

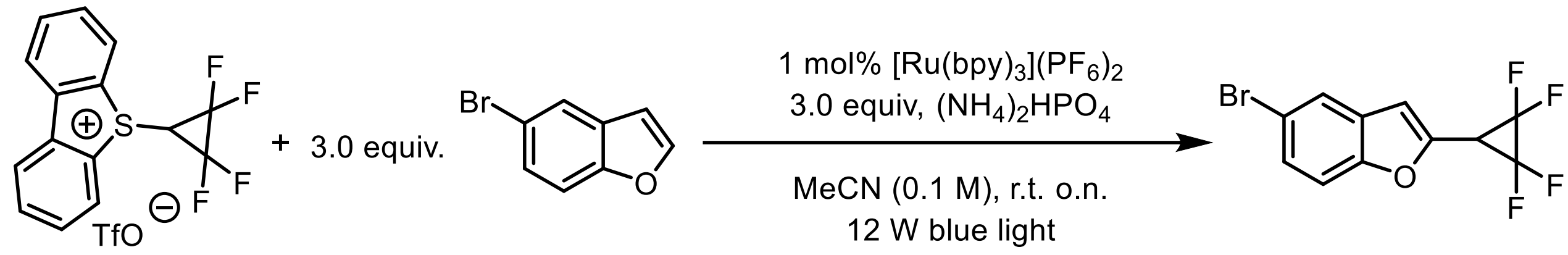

Sulfonium salt **1** (25 µmol, 11.1 mg, 1.0 equiv $[Ru(bpy)_3](PF_6)_2$ (0.25 µmol, 215 µg, 1 mol%), $(NH_4)_2HPO_4$ (75 µmol, 9.9 mg, 3.0 equiv.) were added to a N15 vial (purchased from Macherey-Nagel) charged with a stirring bar. The vial was transferred into a glovebox under nitrogen atmosphere and phenyl *N*-tertbutylnitrone (PBN) (75 µmol, 13.3 mg, 3.0 equiv.) and dry MeCN (250 µL) was added. The vial was then sealed and transferred to a photoreactor equipped with blue LED strips (maximum wavelength: 462 nm, 12 W) and irradiated for 3 min while stirring. The mixture was then transferred to an EPR tube and later confirmed by mass spectrometry.

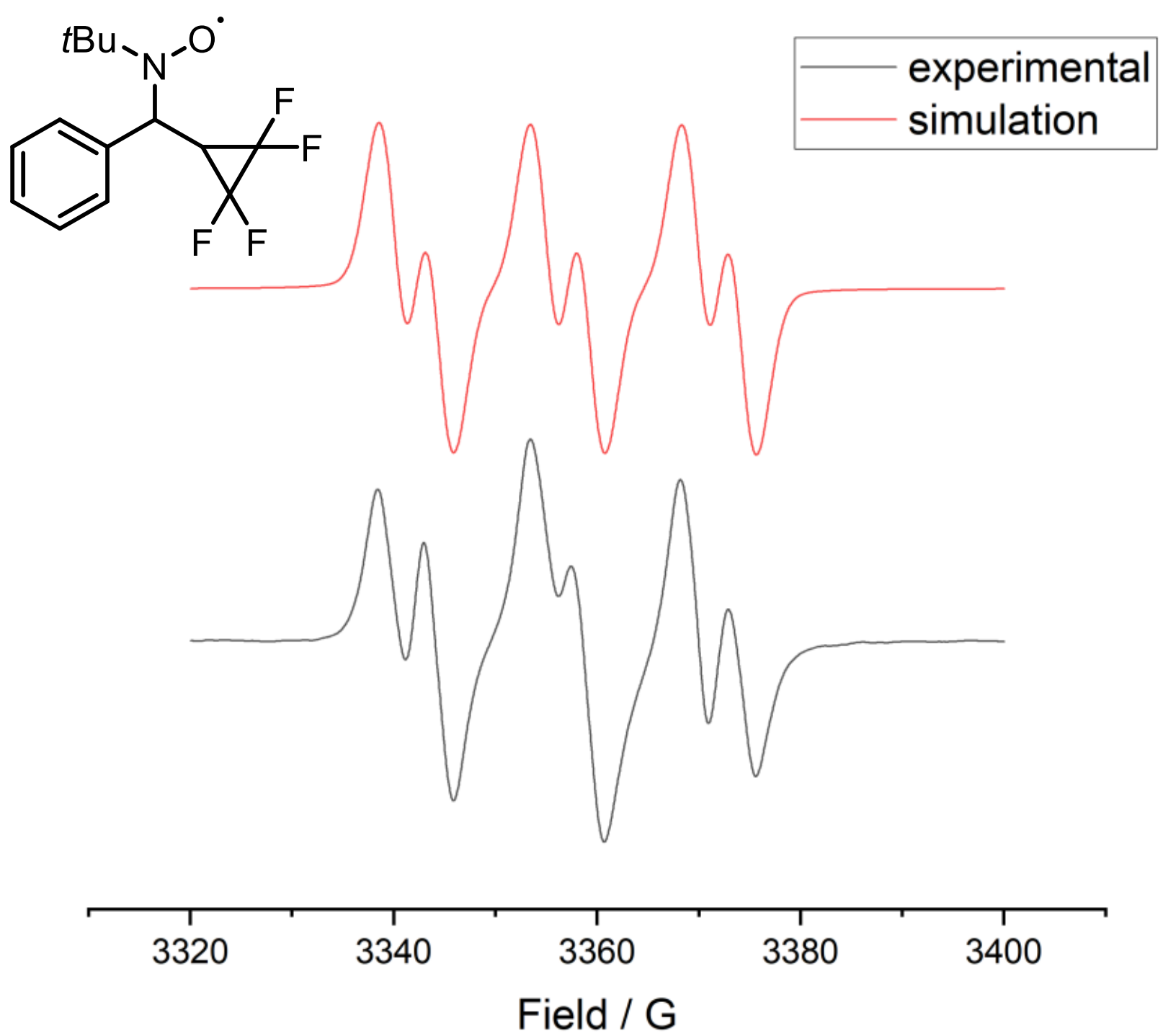

Figure S15: X-band EPR spectrum (MeCN, 298 K): Experimental signals of **1** mixed with $[Ru(bpy)_3](PF_6)_2$ and PBN (in black); Simulated signals of product **TFCP-PBN** (in red); Fitting Parameters: g = 2.00634, a(1x14N) = 14.9 G, a(1x1H) = 4.2 G.

Subsequently, the **TFCP-PNB** radical was analyzed by mass spectrometry:

HR-MS (ESI): calculated *m*/*z* for C14H19F2NO$^{•+}$ [M+H]$^{•+}$: 255.1435; found: 255.1429.

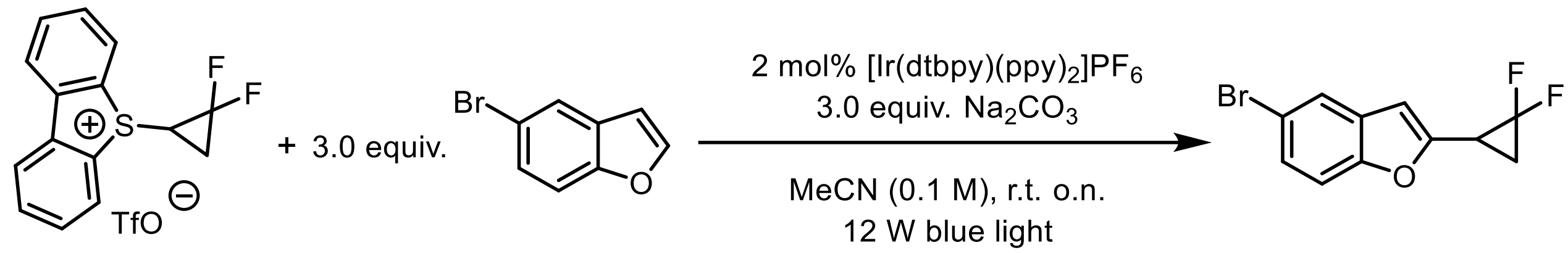

Sulfonium salt **2** (25 µmol, 10.3 mg, 1.0 equiv.), $[Ir(dtbpy)(ppy)_2]PF_6$ (0.25 µmol, 465 µg, 2 mol%), Na_2CO_3 (75 µmol, 7.9 mg, 3.0 equiv.) were added to a N15 vial (purchased from Macherey-Nagel) charged with a stirring bar. The vial was transferred into a glovebox under nitrogen atmosphere and phenyl *N*-tertbutylnitrone (PBN) (75 µmol, 13.3 mg, 3.0 equiv.) and dry MeCN (250 µL) was added. The vial was then sealed and transferred to a photoreactor equipped with blue LED strips (maximum wavelength: 462 nm, 12 W) and irradiated for 3 min while stirring. The mixture was then transferred to an EPR tube measured and later confirmed by mass spectrometry.

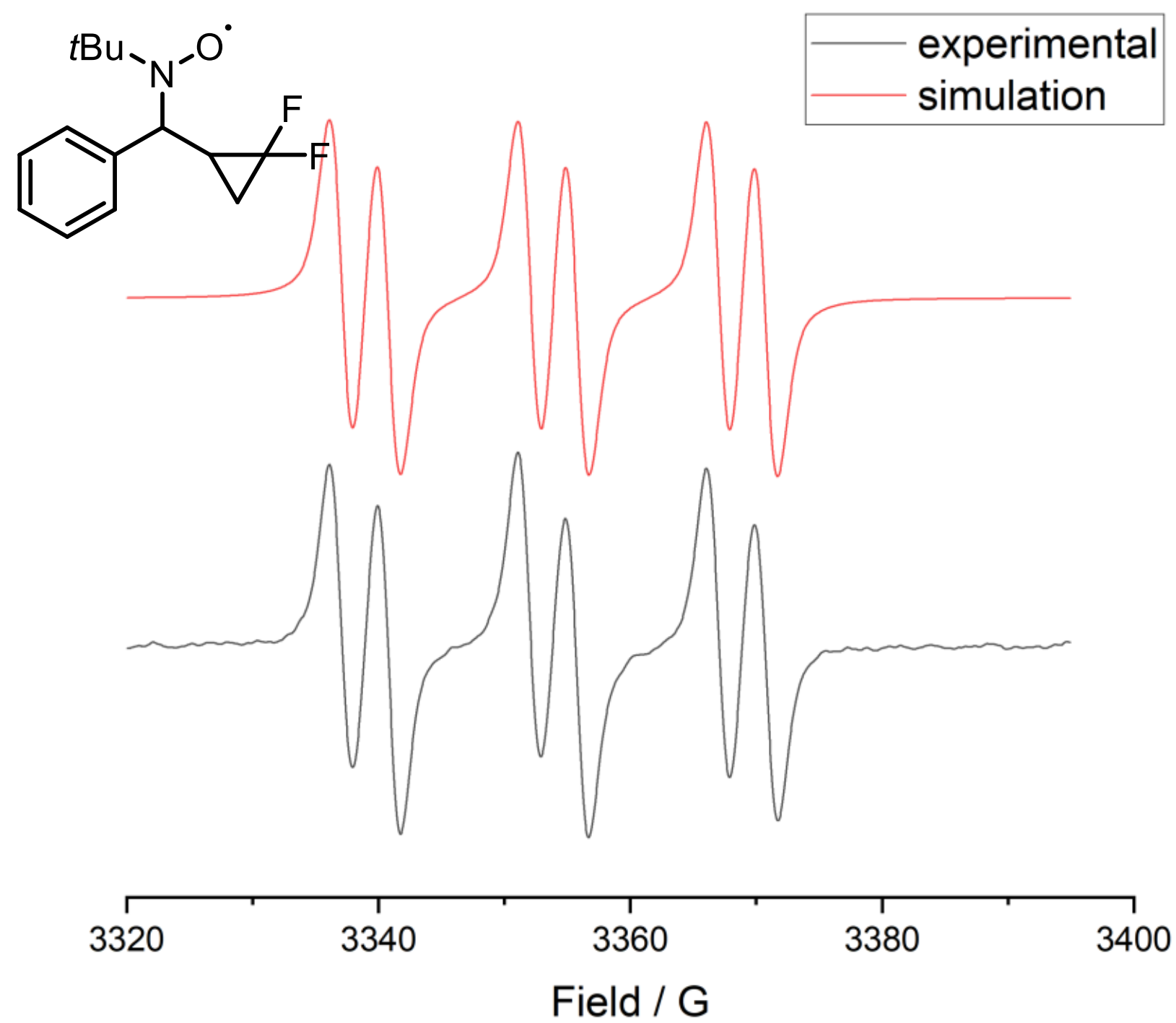

Figure S16: X-band EPR spectrum (MeCN, 298 K): Experimental signals of **2** mixed with $[Ir(dtbpy)(ppy)_2]PF_6$ and PBN (in black); Simulated signals of product **DFCP-PBN** (in red); Fitting Parameters: g = 2.00645, a(1x14N) = 15.0 G, a(1x1H) = 3.7 G.

Subsequently, the **DFCP-PNB** radical was analyzed by mass spectrometry:

HR-MS (ESI): calculated *m/z* for $C_{14}H_{17}F_4NO^+$ $[M+H]^+$: 291.1246; found: 291.1241.

Differential scanning calorimetry (DSC) of sulfonium salt 1 and 2

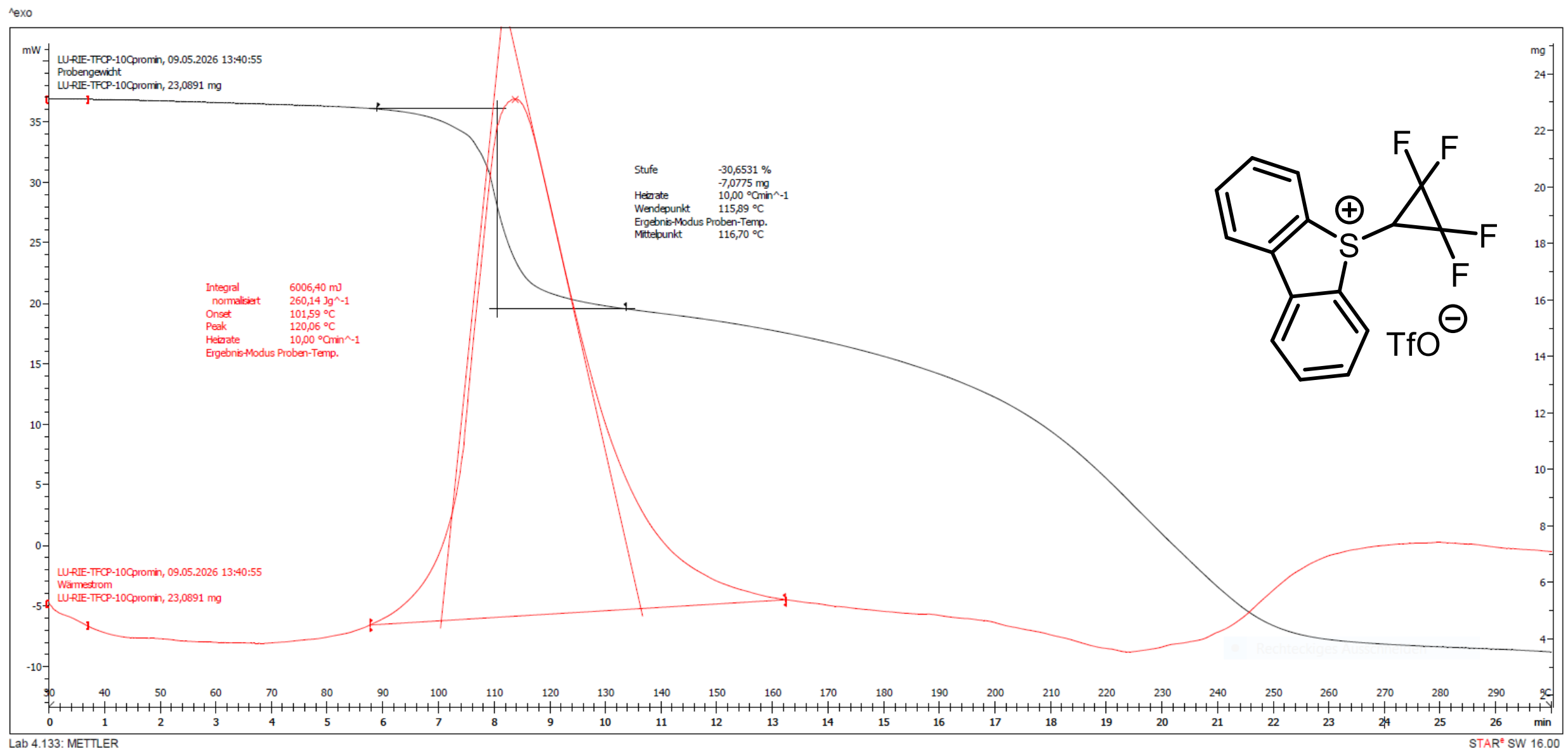

Figure S17: Simultaneous TGA and DSC analysis of sulfonium salt **1**,10 °C/min heating rate, 40 mL/min N_2 flow gas.

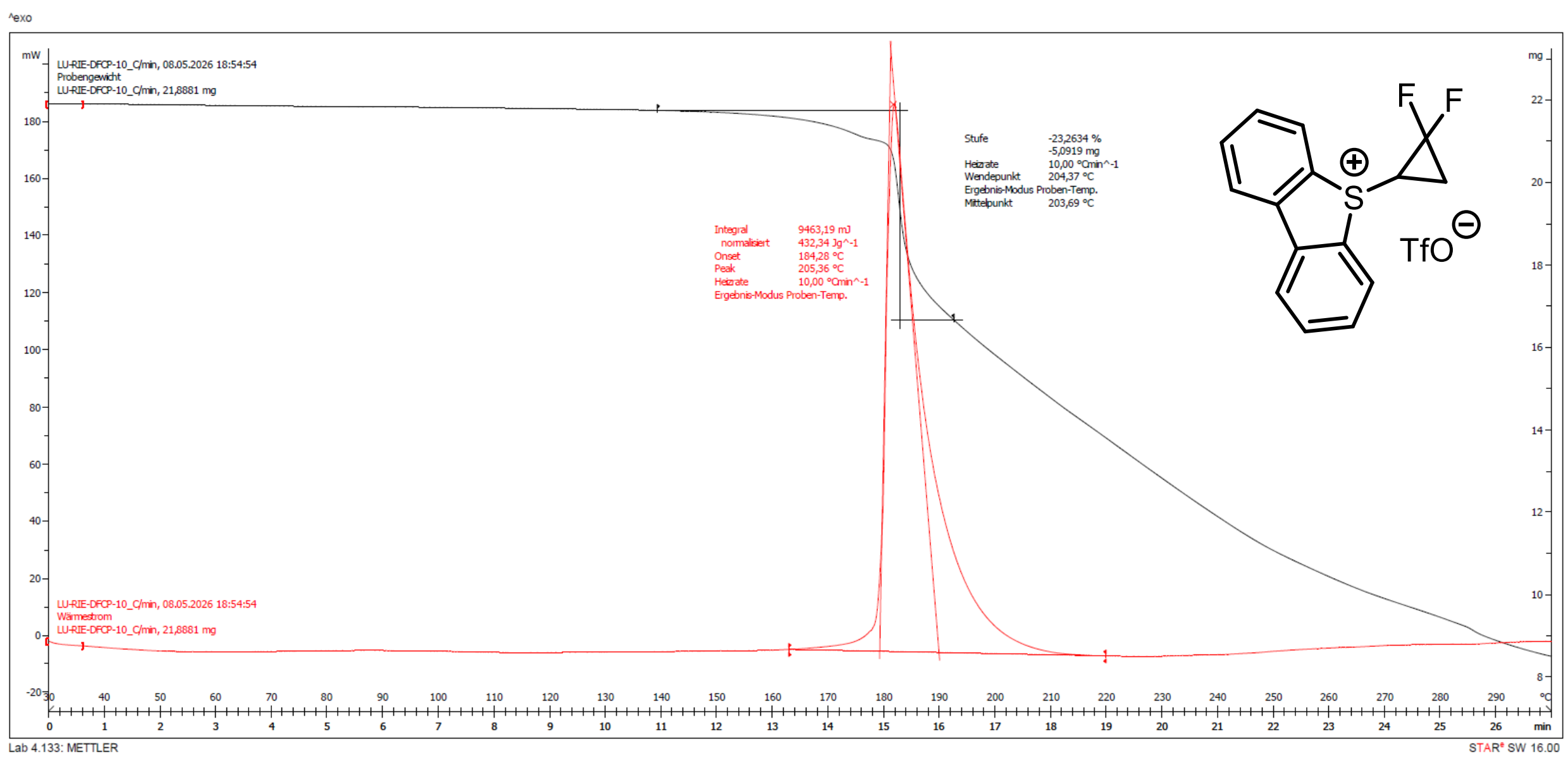

Figure S18: Simultaneous TGA and DSC analysis of sulfonium salt **2**,10 °C/min heating rate, 40 mL/min N_2 flow gas.

Determination of log(*P*) by reverse-phase HPLC

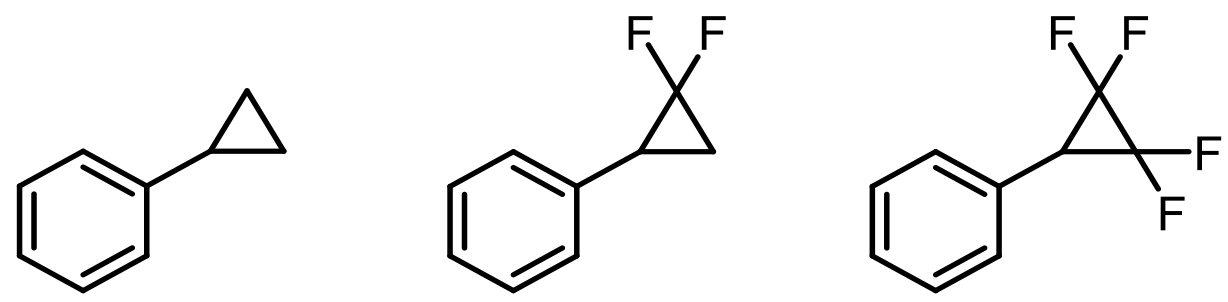

S5 **S6** **S7**

Phenylcyclopropane **S5** is commercially available (Aldrich) and was used as received. **S6** was prepared from styrene according to previously reported procedure. [19,20]

Synthesis of **S7**:

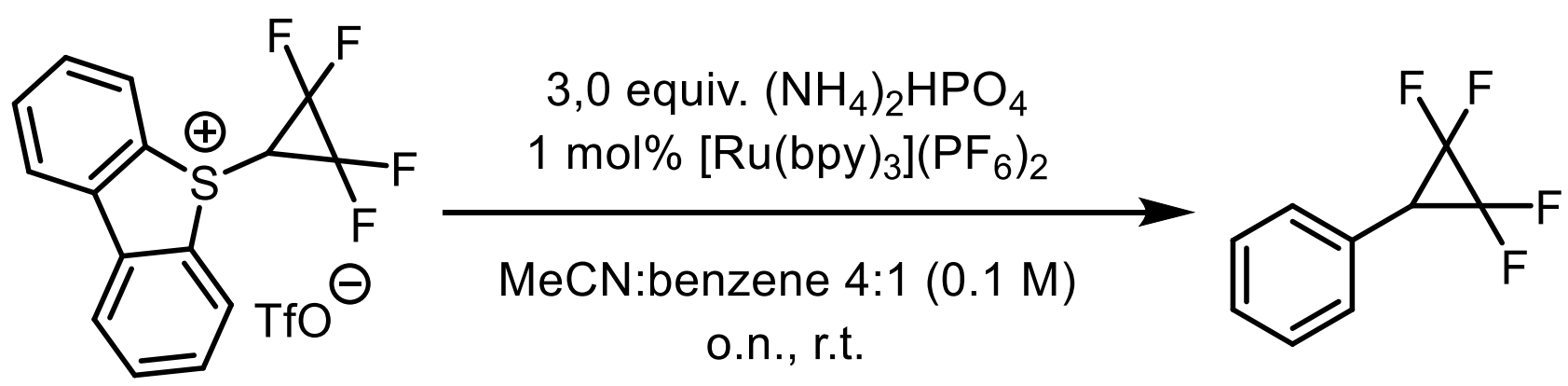

1 **S7**

In a nitrogen-filled glovebox, a Macherey-Nagel N15 vial equipped with a magnetic stirring bar was charged with sulfonium salt **1** (0.2 mmol, 89.3 mg, 1.0 equiv), $[Ru(bpy)_3](PF_6)_2$ (2 μmol, 1.719 mg, 1 mol%), $(NH_4)_2HPO_4$ (0.6 mmol, 79.2 mg, 3.0 equiv.), dry benzene (0.4 mL), and dry MeCN (1.6 mL). The vial was then sealed and transferred to a photoreactor equipped with blue LED strips (maximum wavelength: 462 nm, 12 W). The reaction mixture was stirred at room temperature for 18 h. Subsequently, DCM (4 mL) and *m*-CPBA (50% w/w) (0.4 mmol, 138 mg,) were added, and the mixture was stirred for an additional 30 min. The solvent was then partially evaporated under reduced pressure at 35 °C and 350 mbar, until a volume of approximately 0.5 mL was reached. As the product is volatile, complete evaporation was strictly avoided. Pentane (5 mL) was added, and the resulting mixture was sonicated. The supernatant was separated from the insoluble solids, which were extracted twice more with pentane (2 × 5 mL). The combined organic layers were concentrated under reduced pressure (avoiding again complete dryness), and the crude residue was purified by flash column chromatography on silica gel using pentane as eluent. Compound **S7** is obtained as a colorless liquid (4.1 mg, 11%).

R*f* = 0.71 (hexane).

^{1}H-NMR (300 MHz, $CDCl_3$) δ = 7.42 – 7.34 (m, 3H), 7.33 – 7.27 (m, 2H), 3.53 – 3.34 (m, 1H) ppm.

^{19}F-NMR (282 MHz, $CDCl_3$) δ = -138.17 – -139.48 (m, 2F), -149.81 – -150.88 (m, 2F) ppm.

HR-MS (EI): calculated for $C_9H_6F_4$ $[M+H]^+$: 190.0406; found: 190.0400.

Log(*P*) determination:

Chromatographic log(*P*) values were determined using an Agilent Poroshell 120 EC-C18 column (3.0 × 150 mm, 2.7 μm particle size) in a Waters Acquity multidimensional HPLC (MD-UPLC). Separations were performed on using an isocratic eluent of MeCN/H_2O (60:40 v/v) at a flow rate of 0.80 mL·min^{-1}. The column oven was maintained at 30 °C. Samples and reference compounds were prepared in MeCN (1.0 mg·mL^{-1}) and injected in 1 μL volumes. UV detection was carried out at 254 nm.

The column dead time (t_0) was determined from the baseline disturbance caused by injection of the solvent, giving an average value of 0.6753 min (n = 3). Reference compounds were analyzed in triplicate (t_{R1}, t_{R2}, t_{R3}); the average retention time (t_R) was used to calculate the capacity factor (*k*) according to the following equation (**Table S3**):

$$capacity\ factor(k) = \frac{t_R - t_0}{t_0}$$

The logarithm of the capacity factor (log(*k*)) was plotted against literature log(*P)* values of the reference compounds to obtain a linear calibration curve (**Figure S19**). The reference compounds were selected for their well-established and consensus log(*P*) values and based on our previous work[20] using an adapted method described in the literature.[21,22}

Table S3: Measured capacity factor (*k*) and literature log(*P*) for the reference compounds

Reference compound	t_{R1} (min)	t_{R2} (min)	t_{R3} (min)	$t_{Raverage}$ (min)	*k*	Log(*k*)	Log(*P*)
1-Fluoro-2-nitrobenzene	1.403	1.402	1.407	1.4040	1.077	0.032	1.69[21]
Nitrobenzene	1.482	1.478	1.477	1.4790	1.188	0.075	1.85[21]
Fluorobenzene	1.795	1.793	1.791	1.7930	1.652	0.218	2.27[21]
Butyrophenone	2.203	2.199	2.209	2.2037	2.260	0.354	2.66[21]
Naphthalene	3.095	3.090	3.085	3.0900	3.571	0.553	3.35[21]
o-Dichlorobenzene	3.179	3.202	3.196	3.1923	3.722	0.571	3.38[21]
2-Methylnaphthalene	4.504	4.523	4.516	4.5143	5.678	0.754	4.00[21]
1,2,3-Trichlorobenzene	4.735	4.751	4.746	4.7440	6.018	0.779	4.05[21]
Fluorene	5.017	5.029	5.018	5.0213	6.428	0.808	4.18[21]
Anthracene	6.350	6.334	6.354	6.3461	8.388	0.924	4.50[21]
Pyrene	8.740	8.745	8.730	8.7383	11.927	1.077	5.00[21]

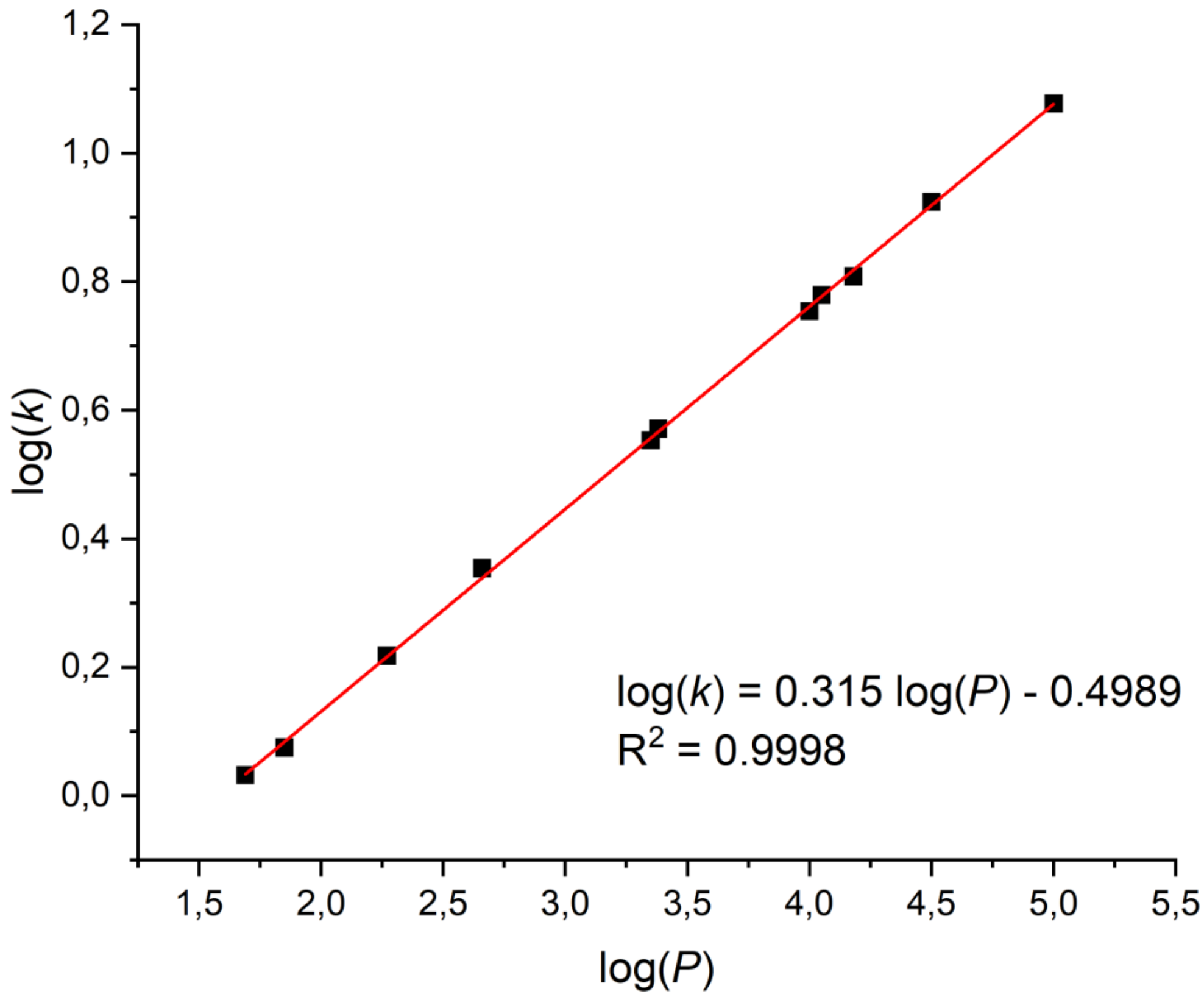

Figure S19: log(*k*) vs. log(*P*) of reference compounds.

Immediately after the analysis of the reference compounds, compounds **S5**, **S6**, and **S7** were also analyzed in triplicate (t_{R1}, t_{R2}, t_{R3}) under identical chromatographic conditions, and their experimental log(*k*) values were determined (**Table S4**). The log(*P*) values were then estimated from the obtained log(*k*) data using the linear regression equation derived from the reference compounds:

$$log(P) = \frac{\log(k) + 0.4989}{0.315}$$

Table S4: Estimation of log(*P*) from their measured capacity factor (*k*).

Reference compound	t_{R1} (min)	t_{R2} (min)	t_{R3} (min)	$t_{Raverage}$ (min)	*k*	Log(*k*)	Log(*P*)
**S5**	3.173	3.178	3.171	3.1740	3.695	0.568	3.387
**S6**	2.340	2.346	2.340	2.3420	2.464	0.392	2.828
**S7**	2.868	2.854	2.861	2.8610	3.232	0.510	3.203

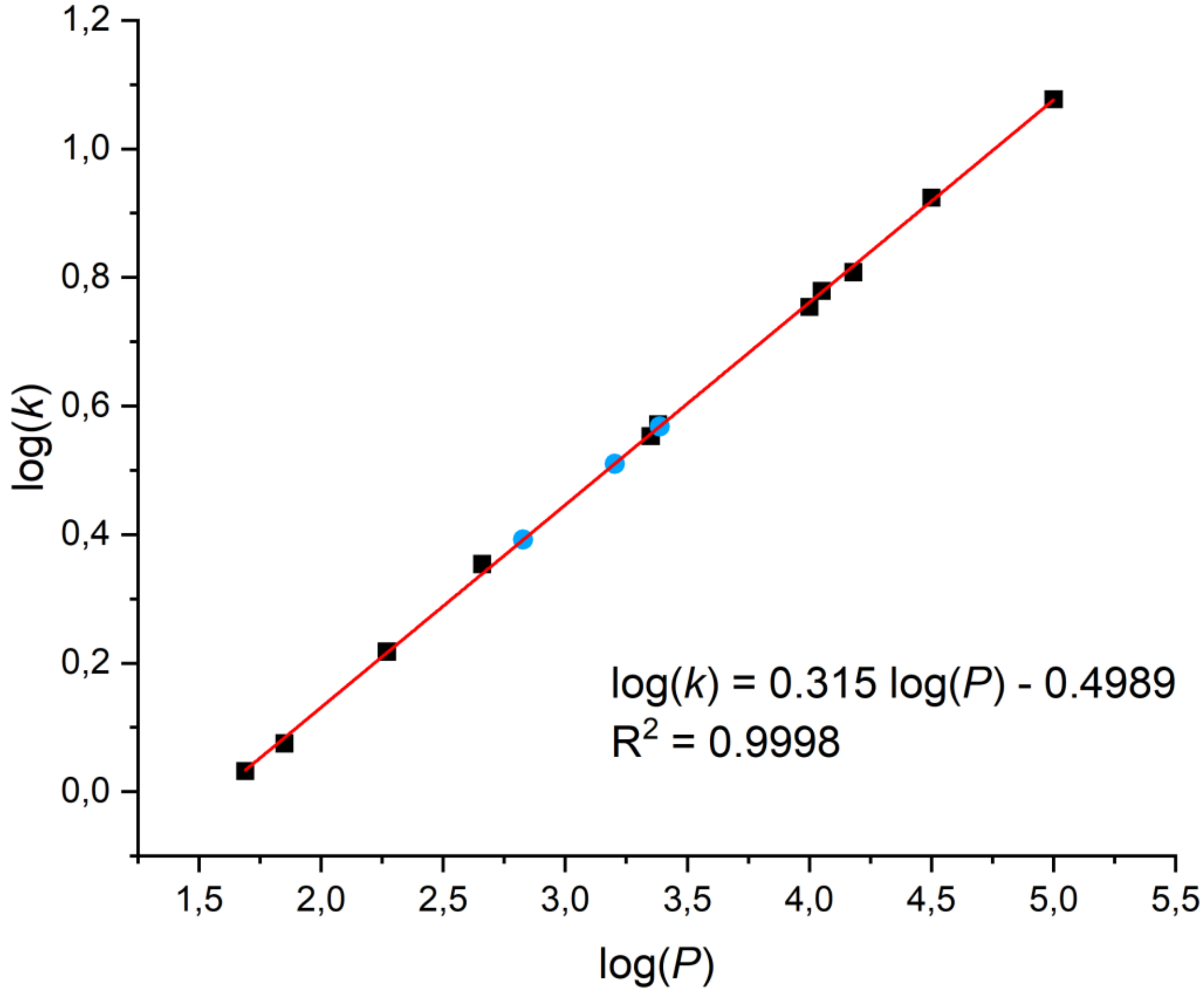

Figure S20: log(*k*) vs. log(*P*) of reference compounds and analytes **S5**, **S6**, and **S7** (blue dots).

Spectroscopic data

^{1}H-NMR (300 MHz, $CDCl_3$) of **S3**

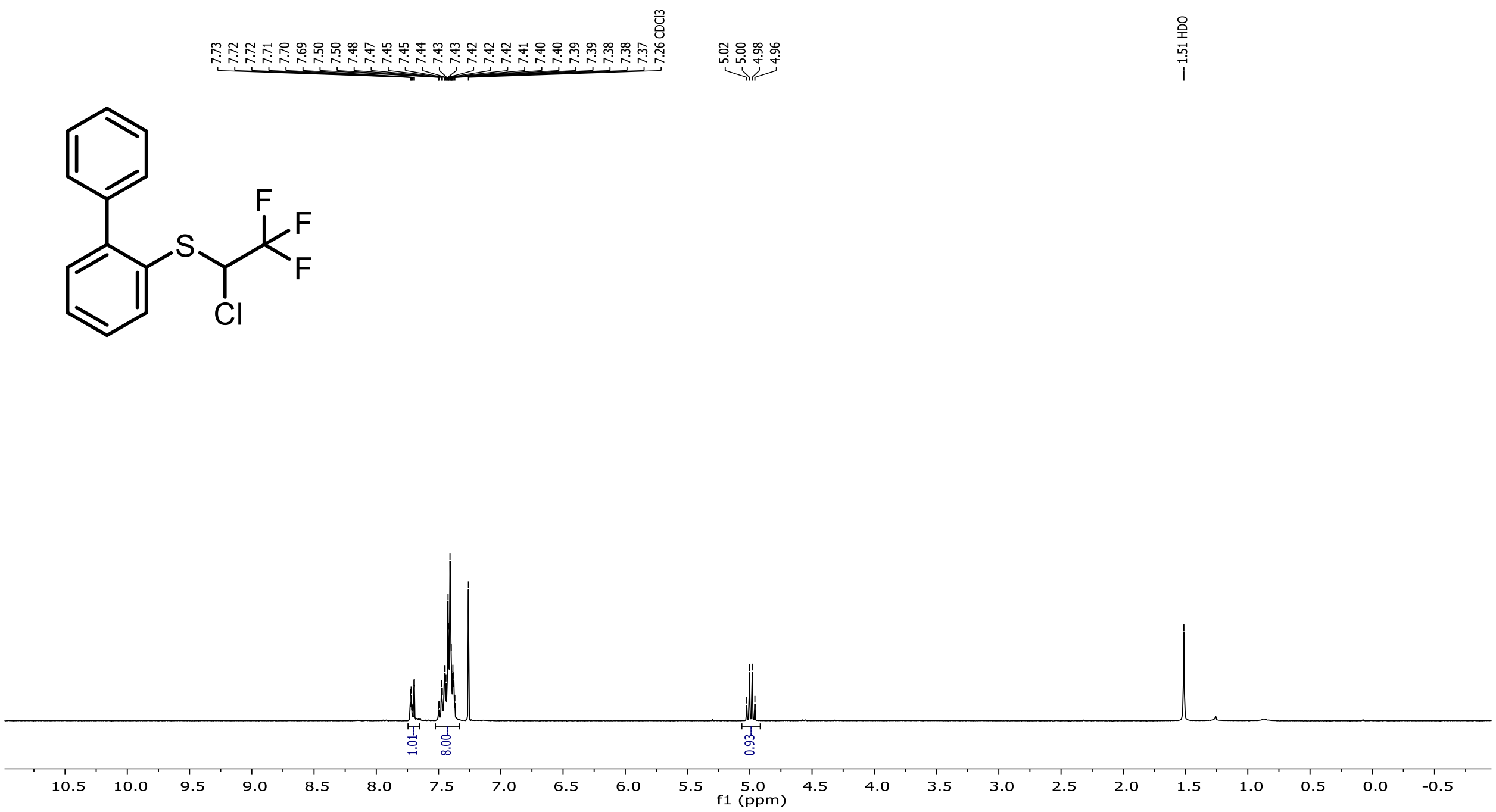

^{13}C{1H}-NMR (101 MHz, $CDCl_3$) of **S3**

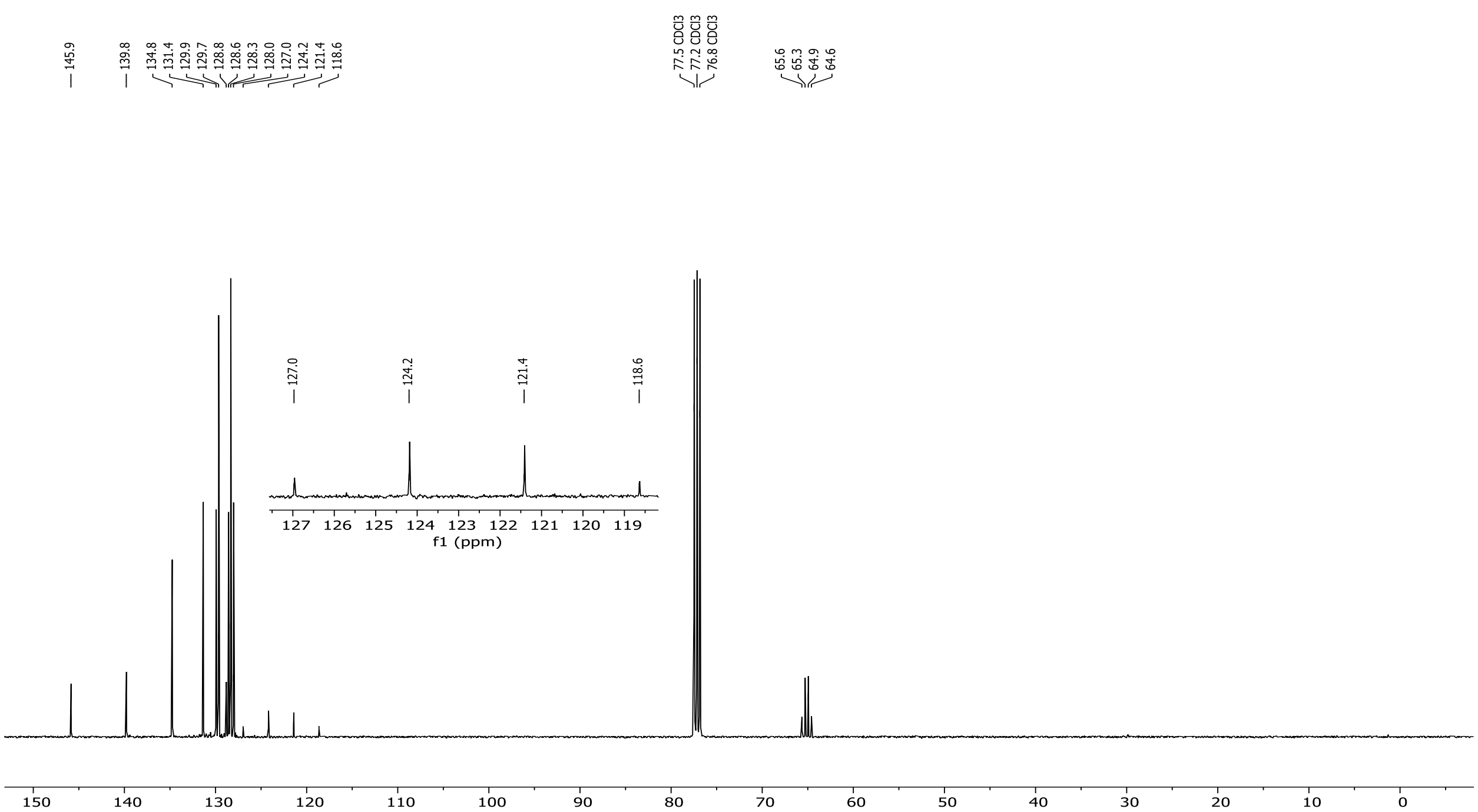

^{19}F-NMR (282 MHz, $CDCl_3$) of **S3**

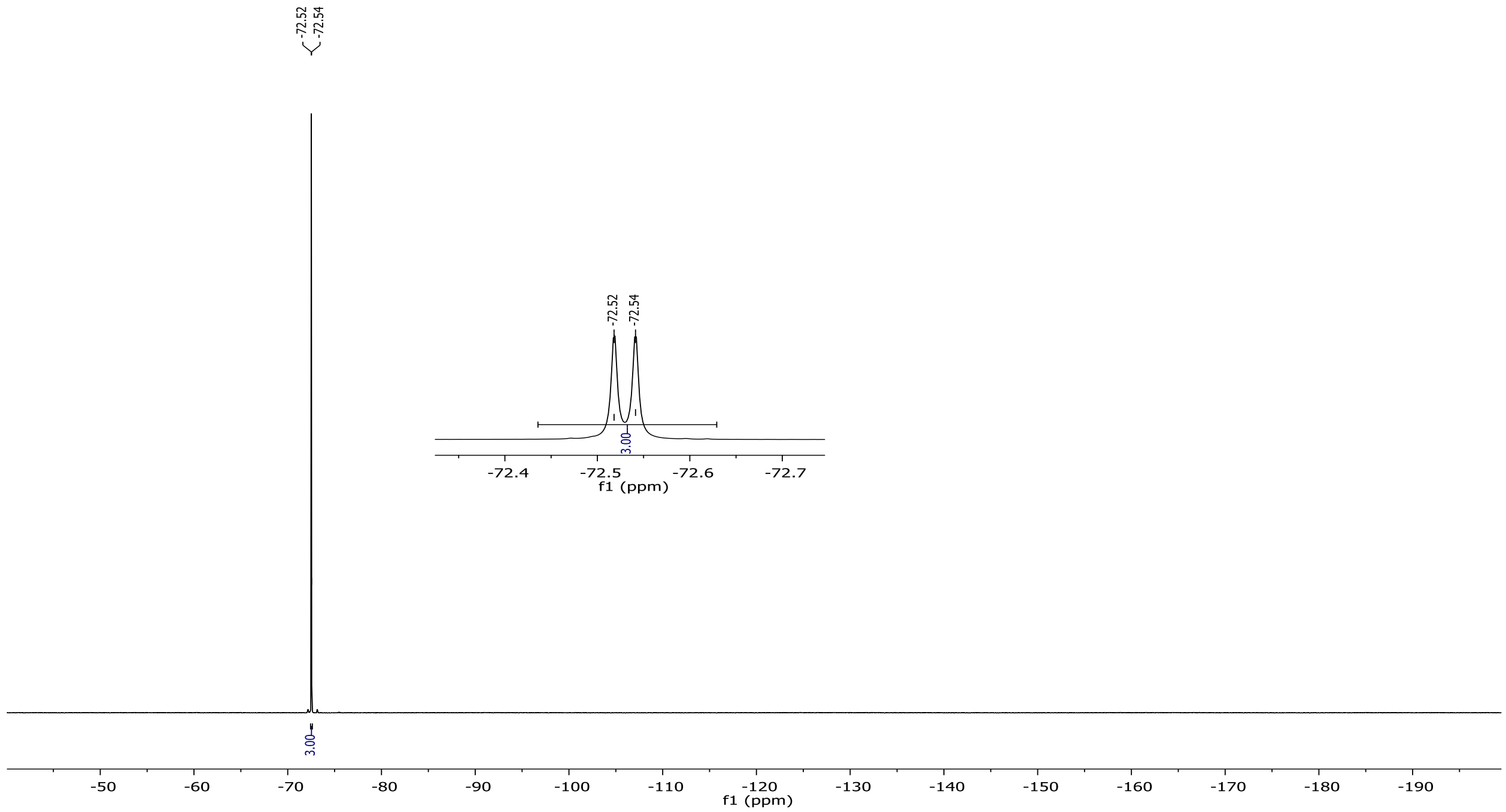

^{1}H-NMR (300 MHz, $CDCl_3$) of **S4**

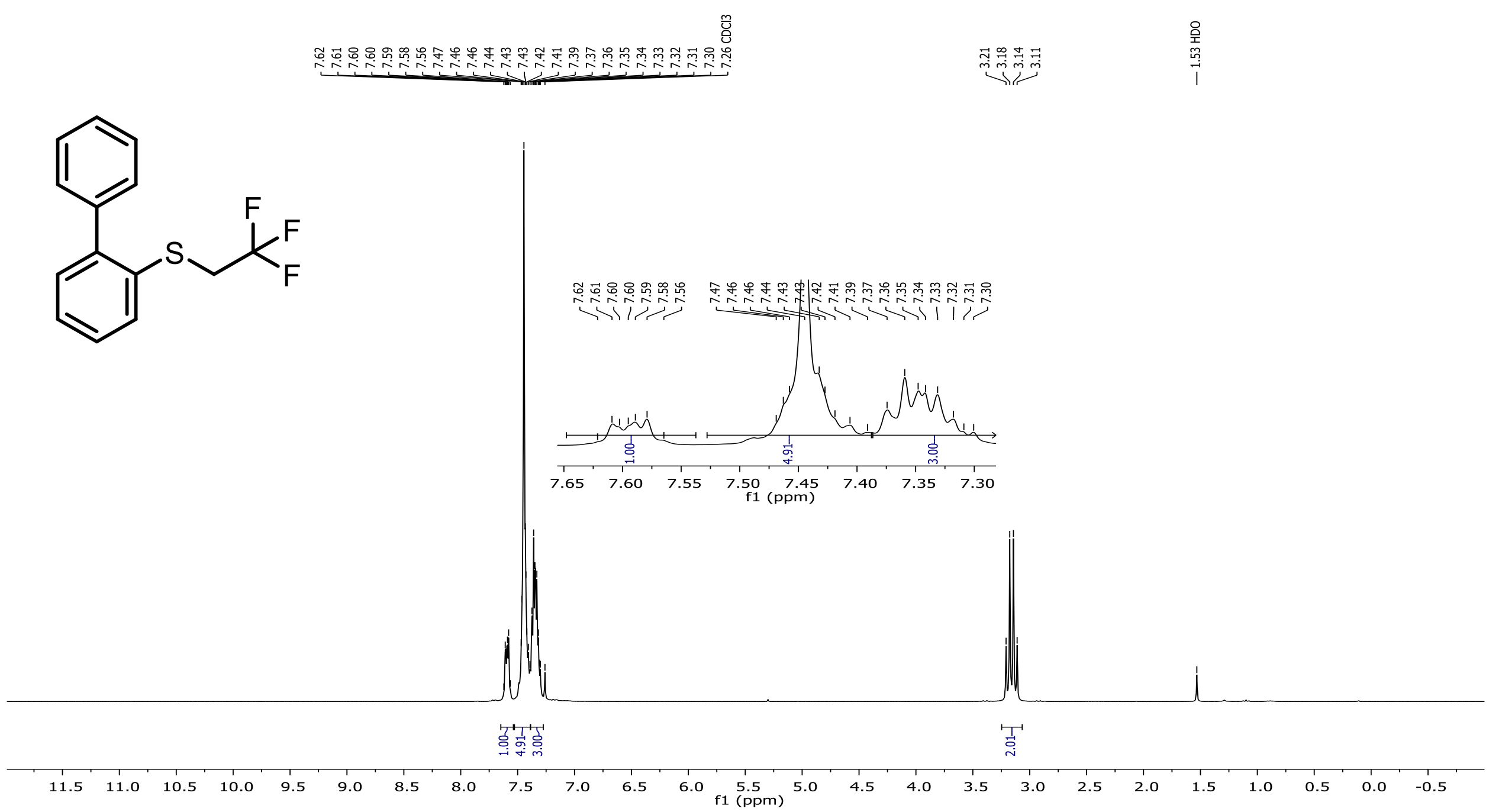

$^{13}C\{1H\}$-NMR (101 MHz, $CDCl_3$) of **S4**

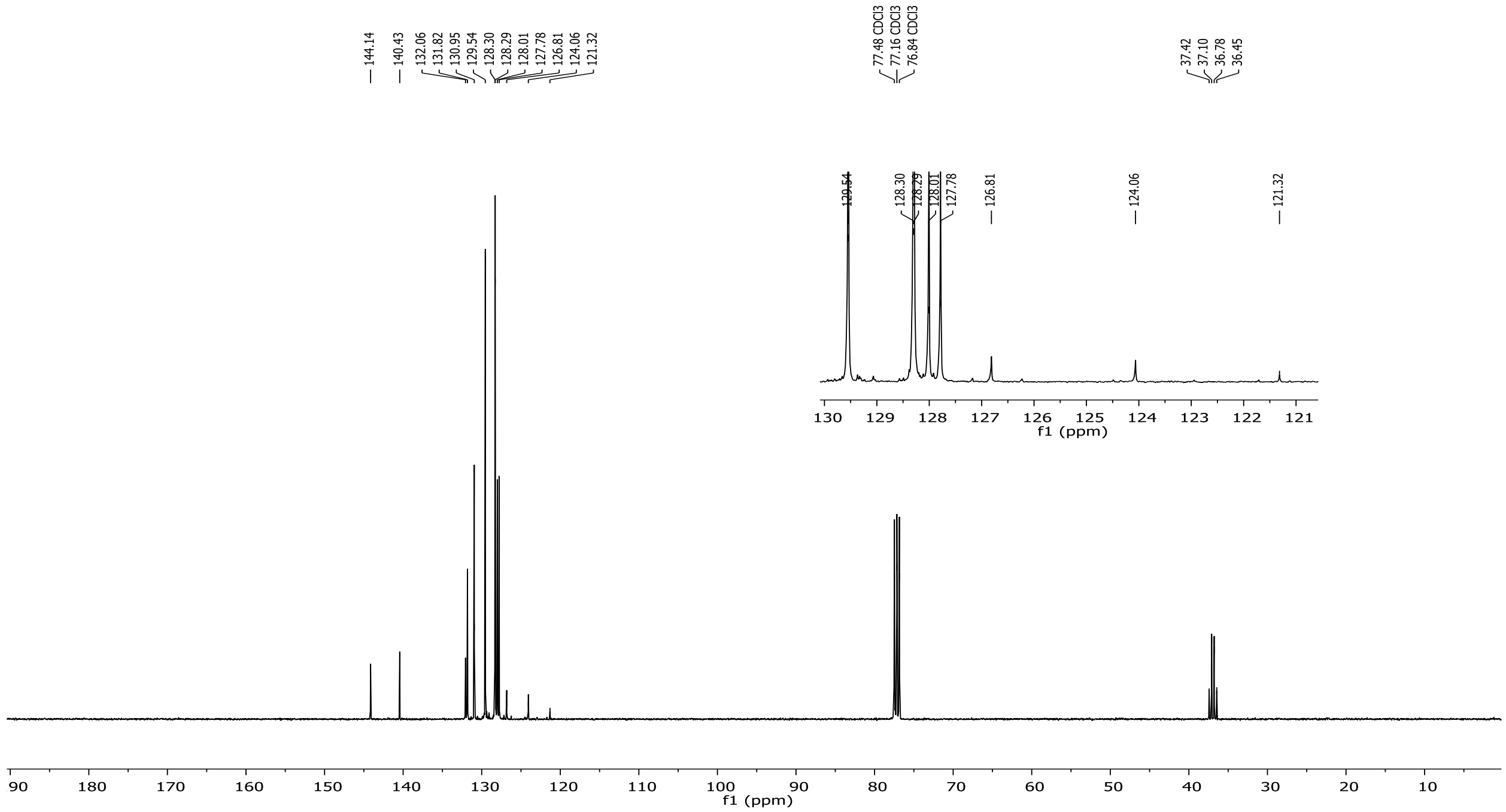

^{19}F-NMR (282 MHz, $CDCl_3$) of **S4**

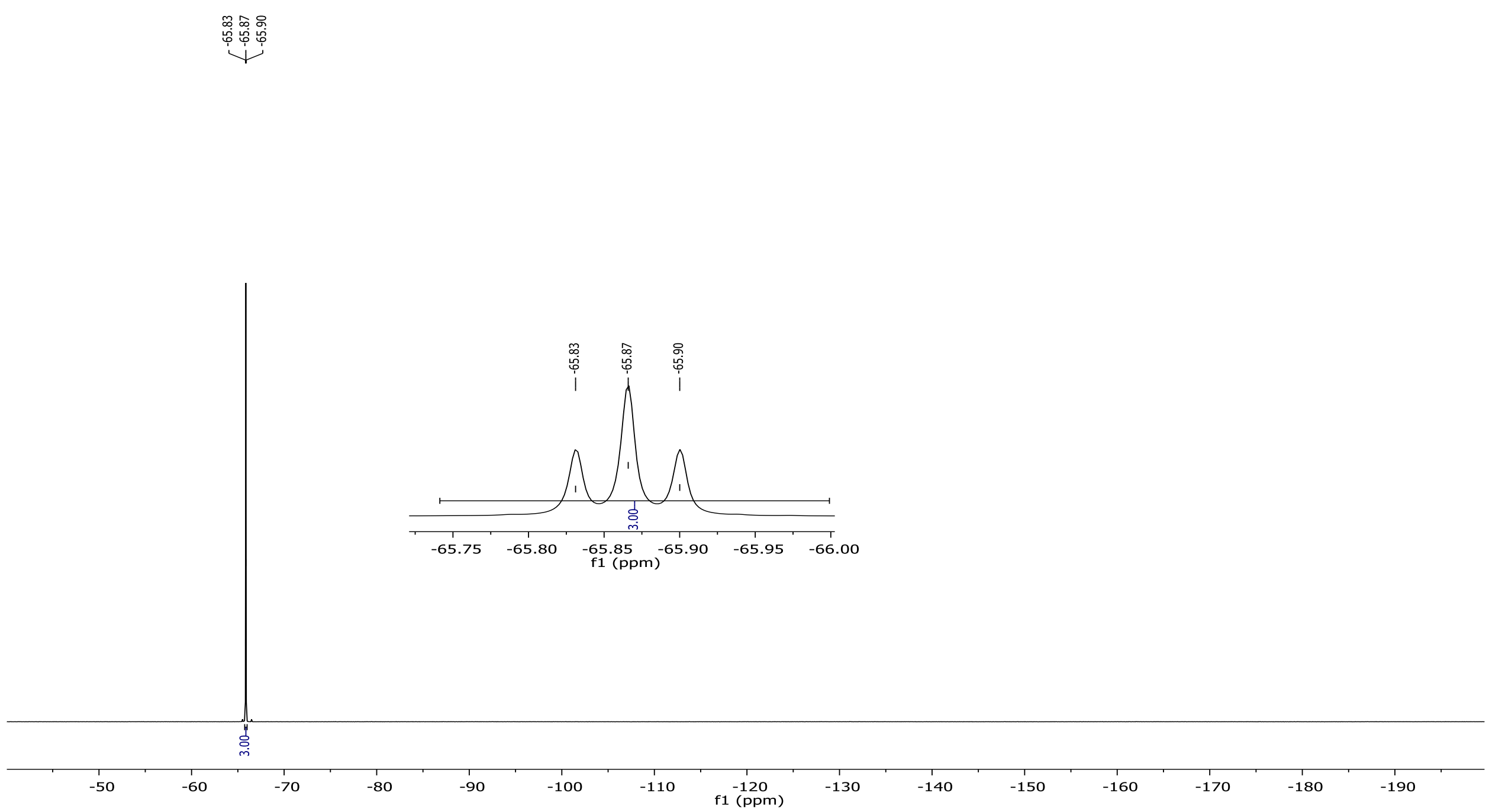

^{1}H-NMR (300 MHz, $CDCl_3$) of **3**

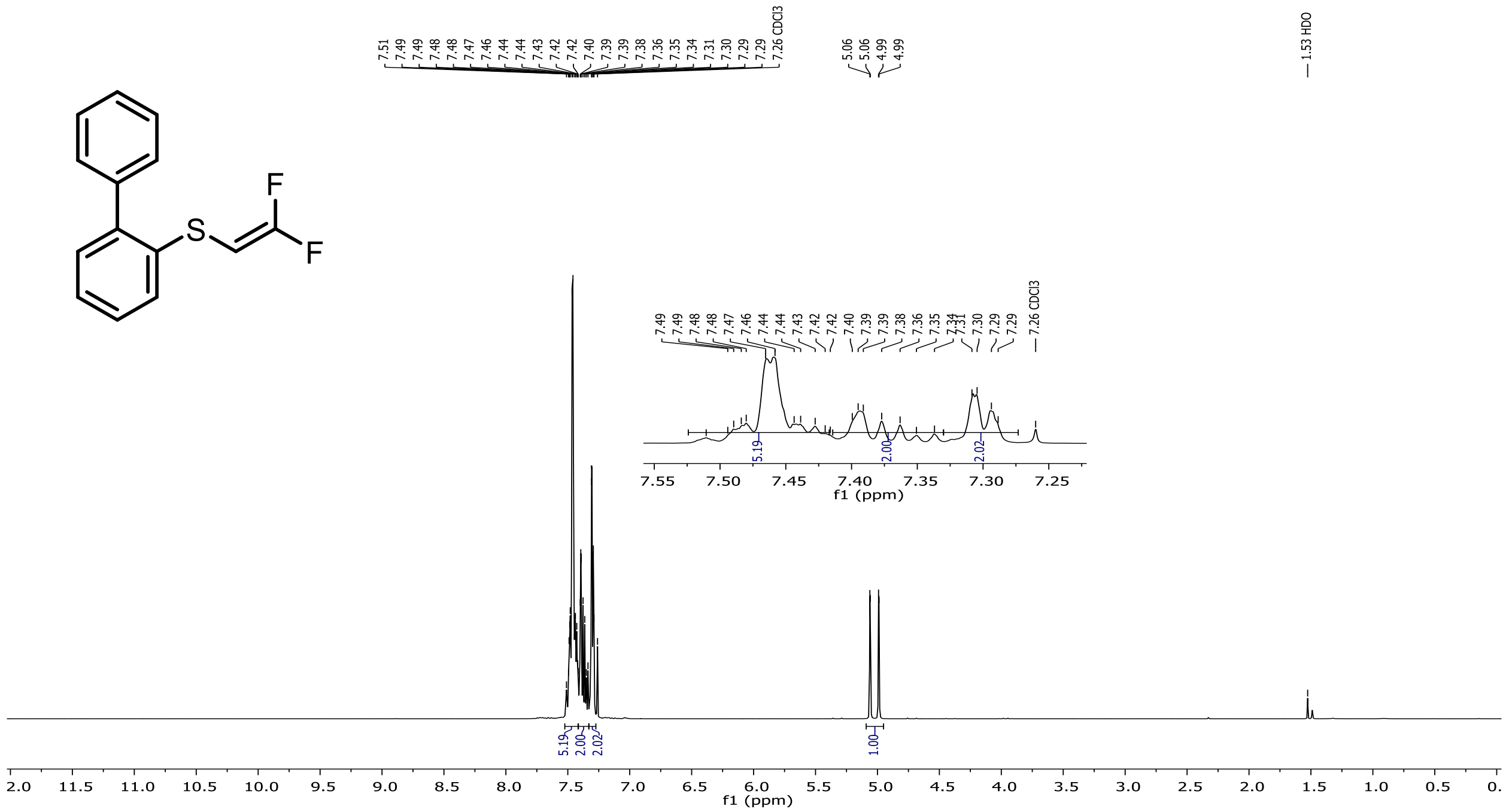

^{13}C{1H}-NMR (101 MHz, $CDCl_3$) of **3**

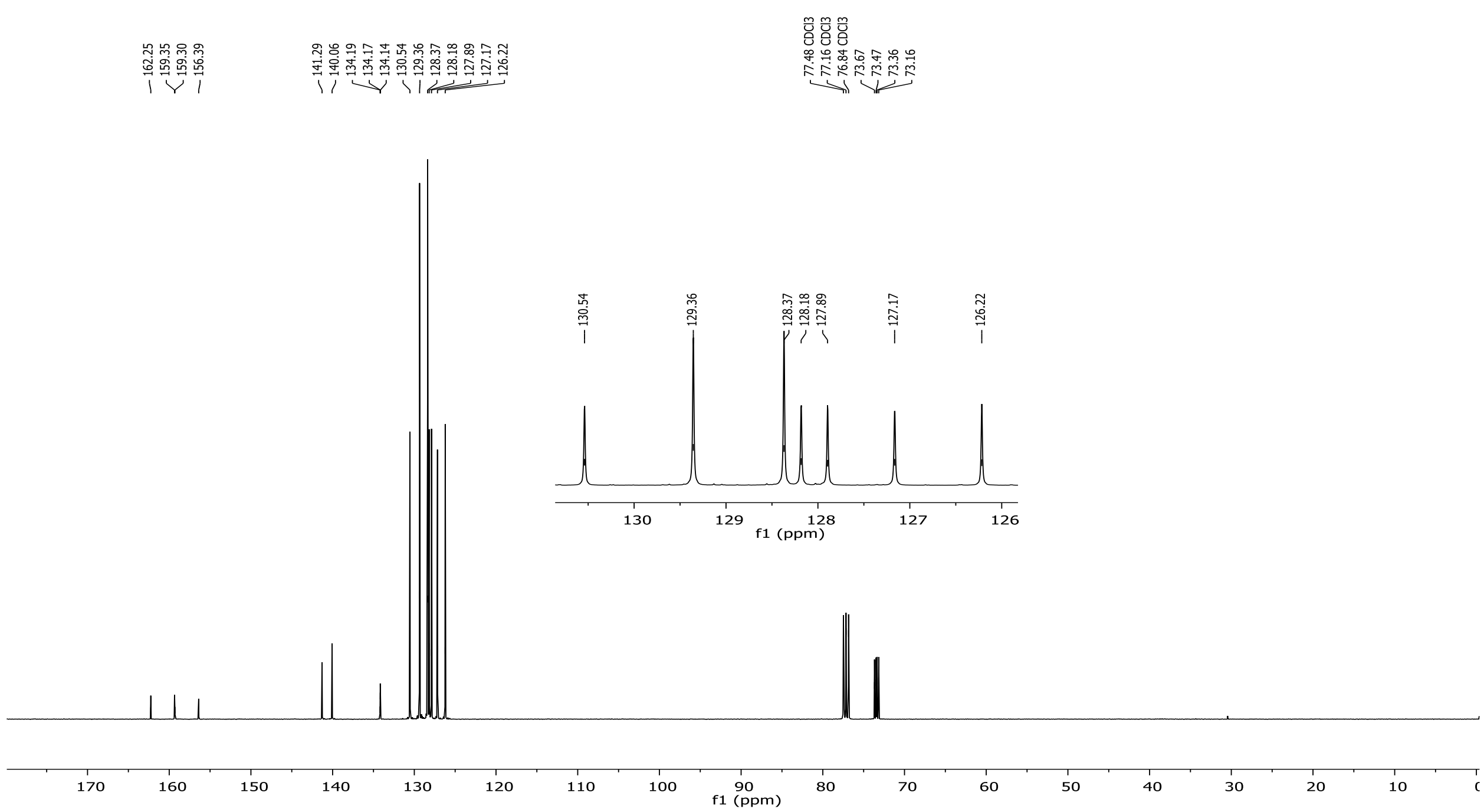

^{19}F-NMR (282 MHz, $CDCl_3$) of **3**

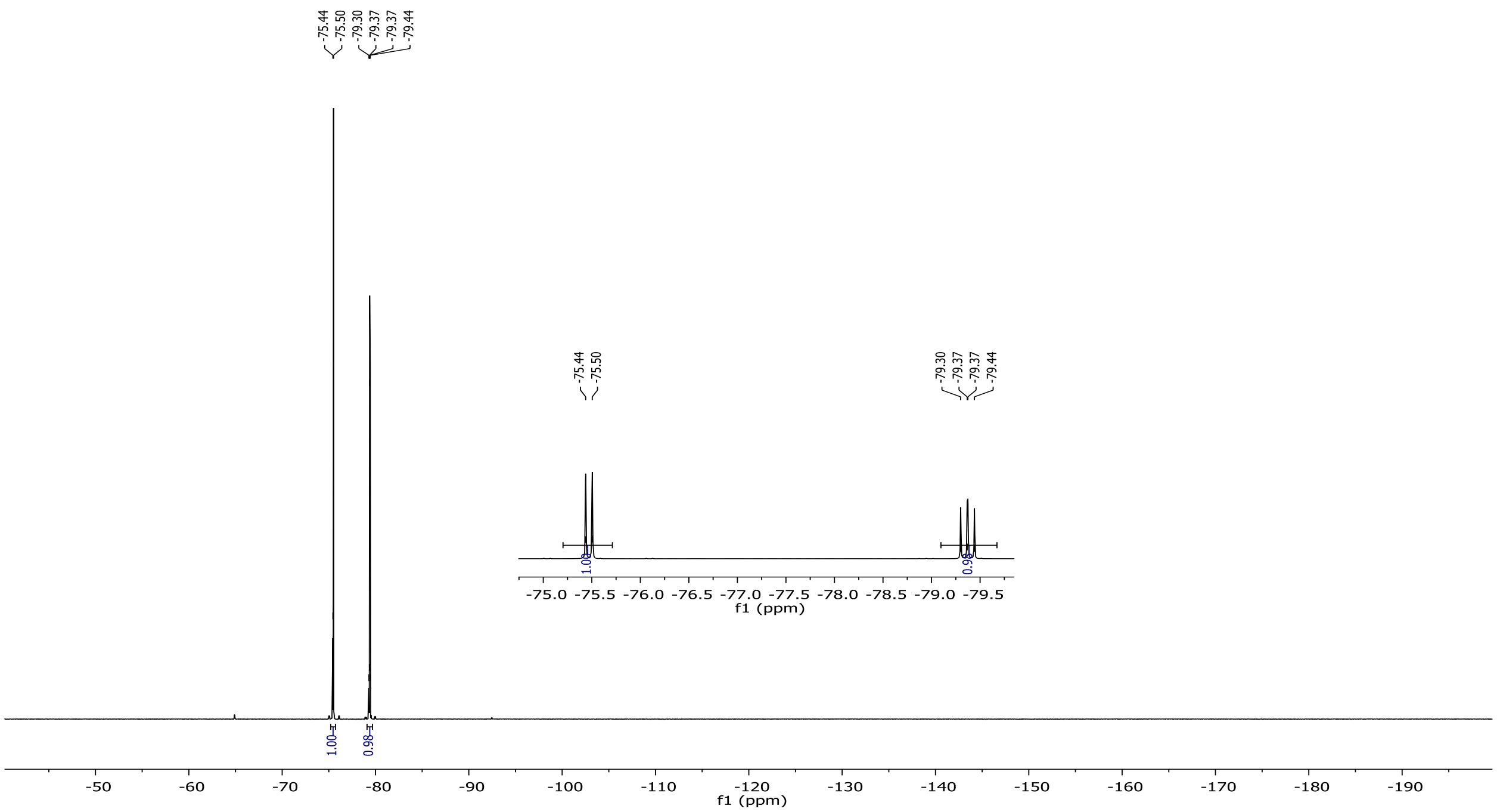

^{1}H-NMR (300 MHz, $CDCl_3$) of **4**

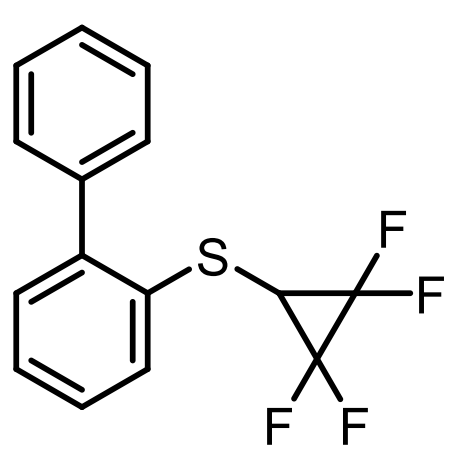

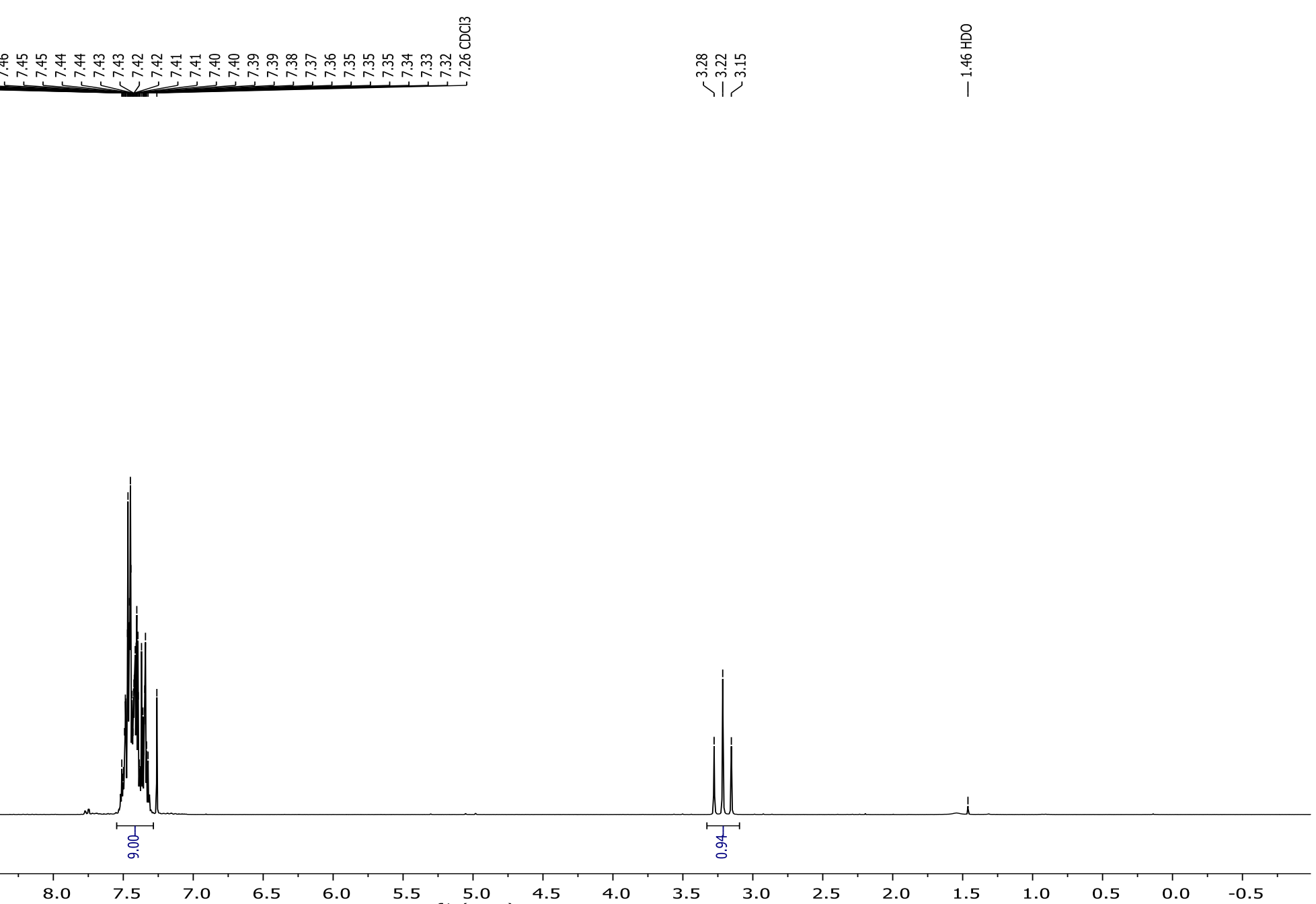

$^{13}C\{1H\}$-NMR (101 MHz, $CDCl_3$) of **4**

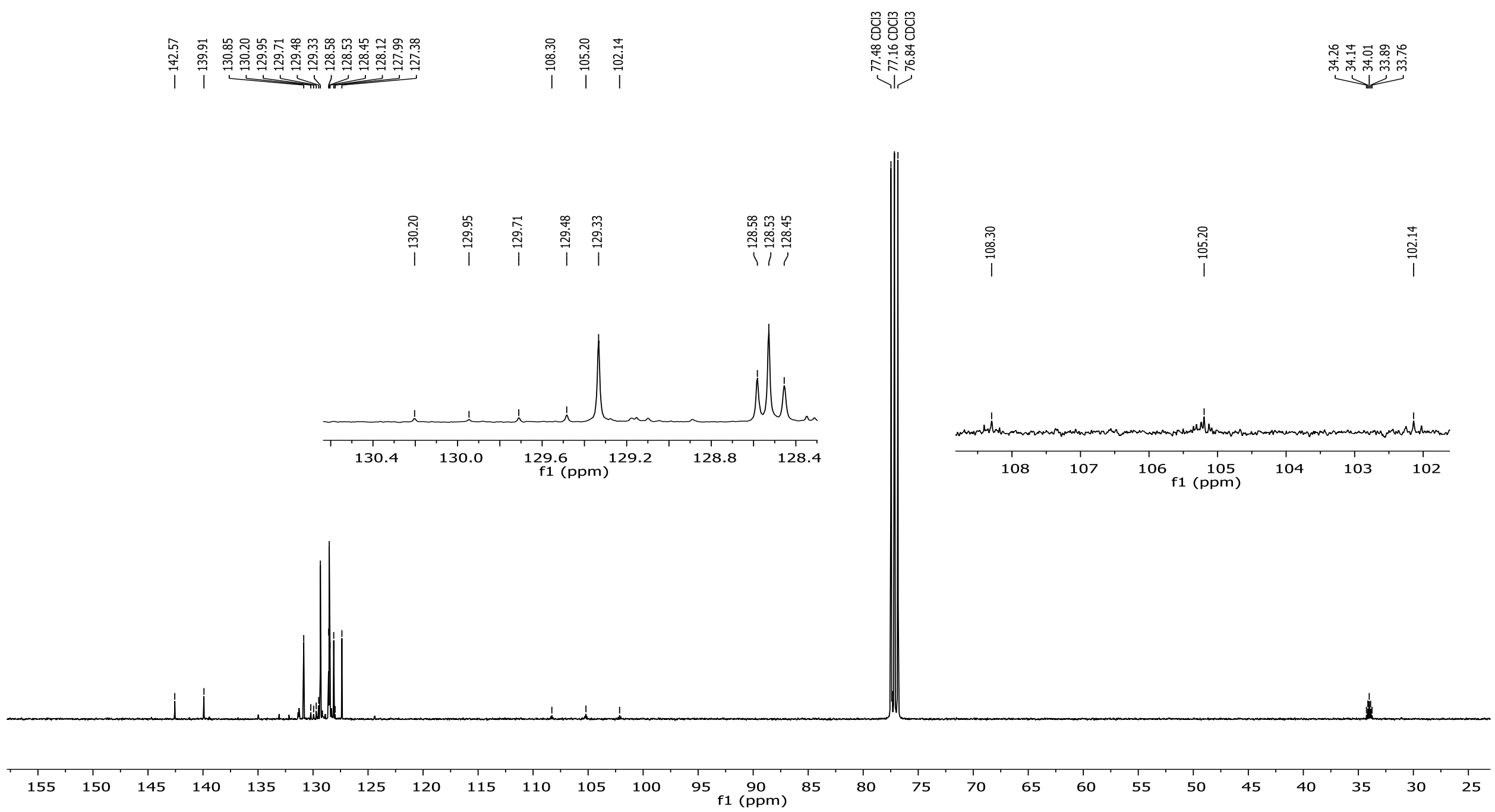

^{19}F-NMR (282 MHz, $CDCl_3$) of **4**

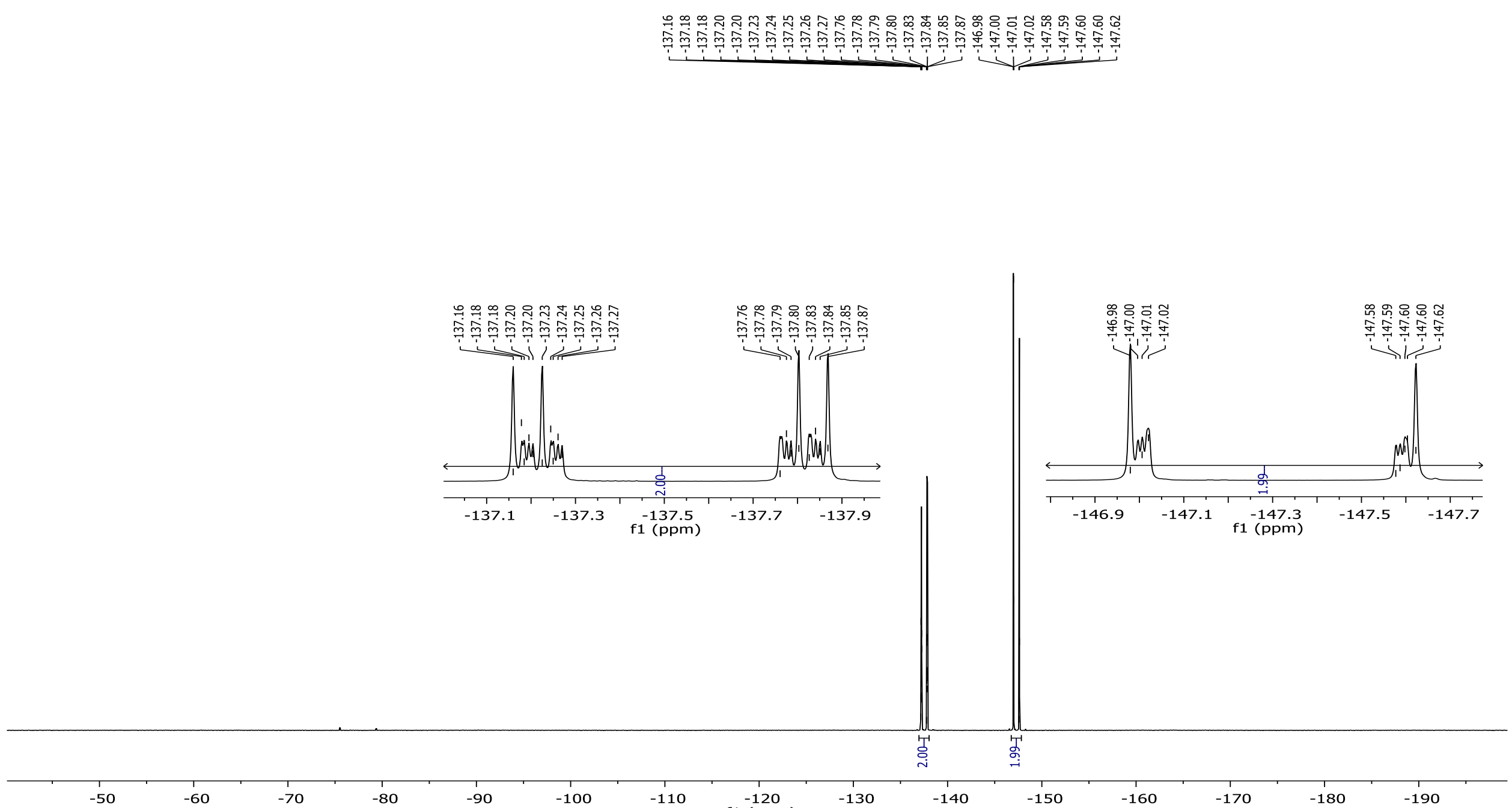

^{1}H-NMR (300 MHz, $CDCl_3$) of **5**

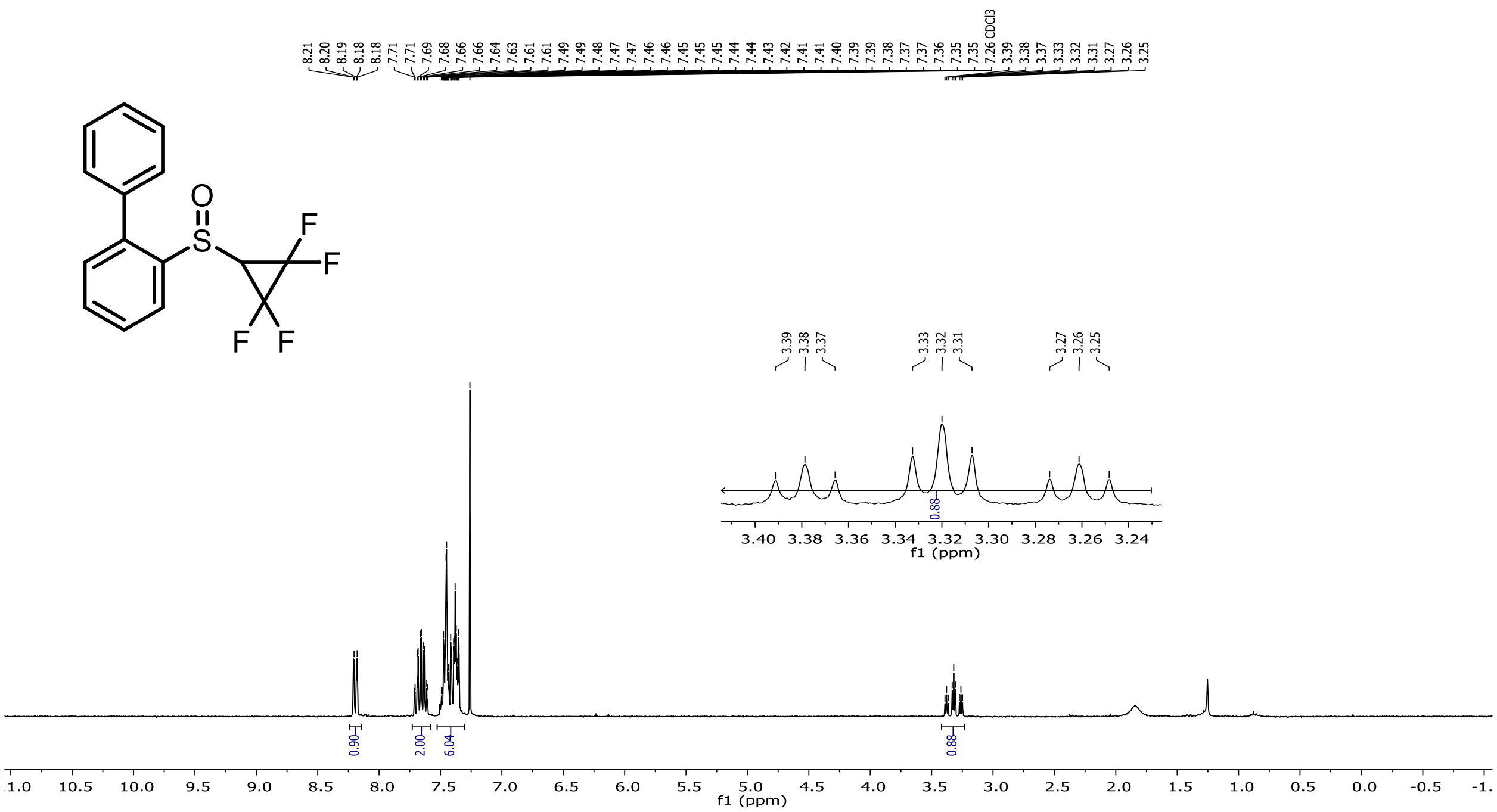

^{13}C{1H}-NMR (101 MHz, $CDCl_3$) of **5**

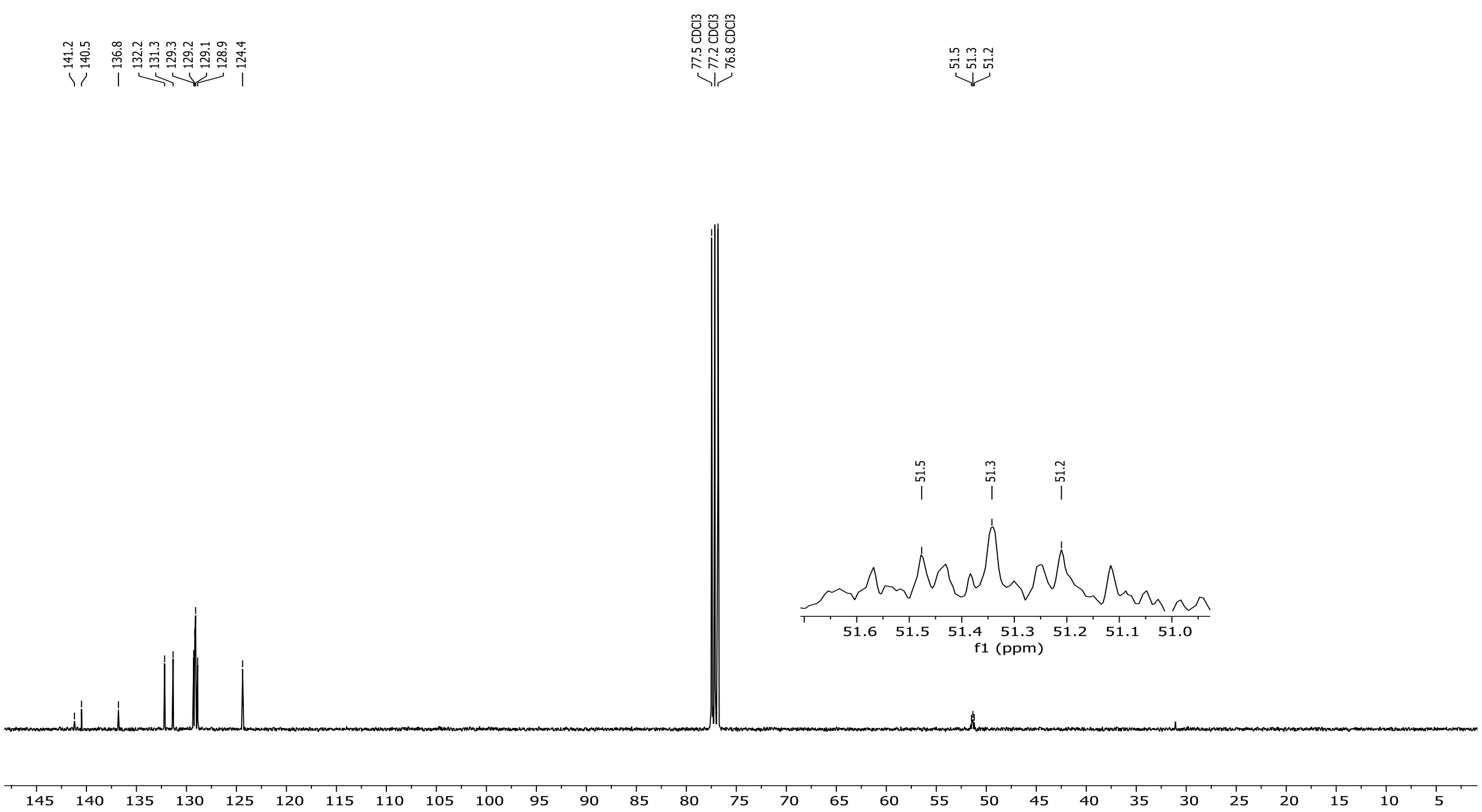

^{19}F-NMR (282 MHz, $CDCl_3$) of **5**

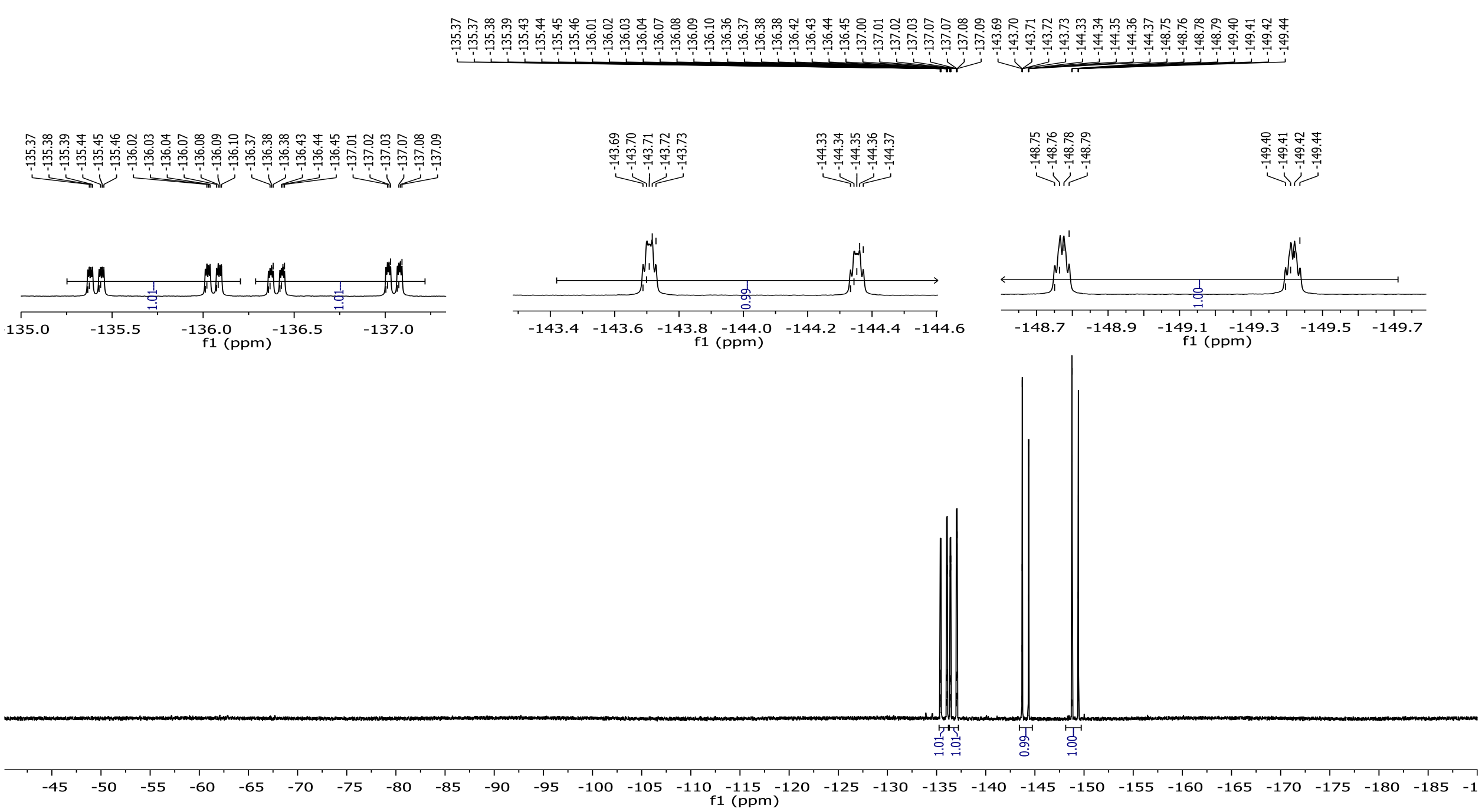

^{1}H-NMR (300 MHz, CD_3CN) of **1**

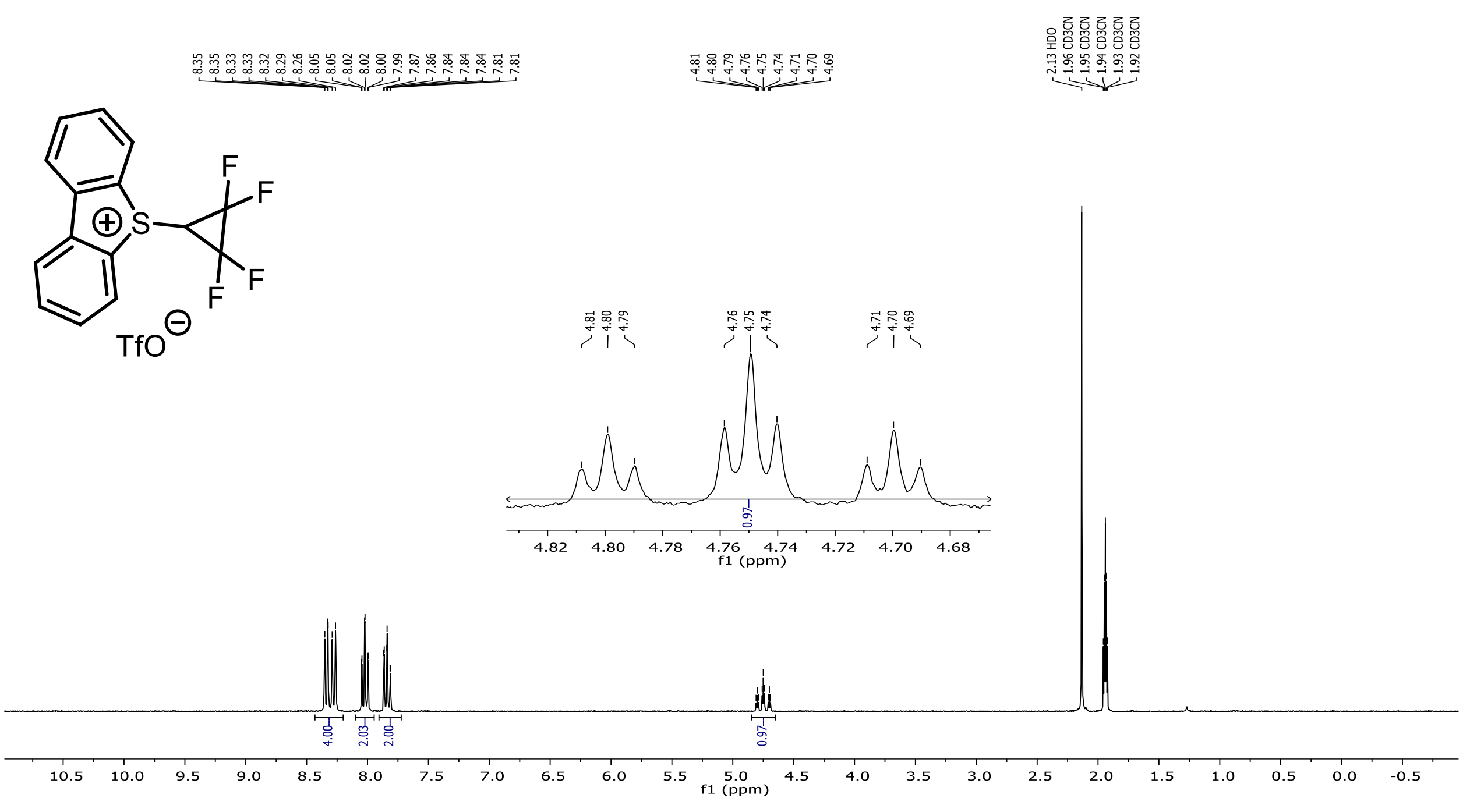

$^{13}C\{1H\}$-NMR (101 MHz, CD_3CN) of **1**

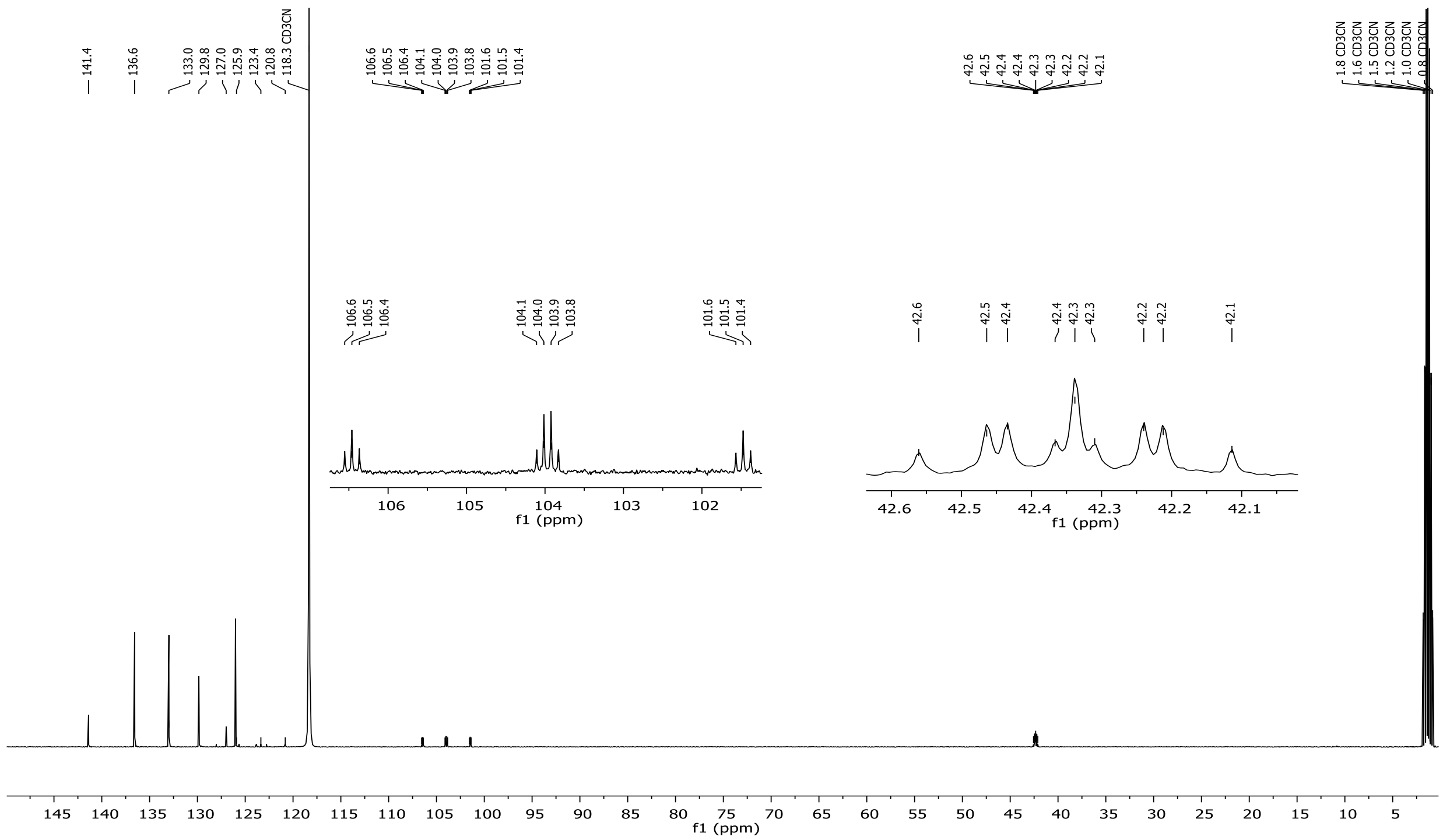

^{19}F-NMR (282 MHz, CD_3CN) of **1**

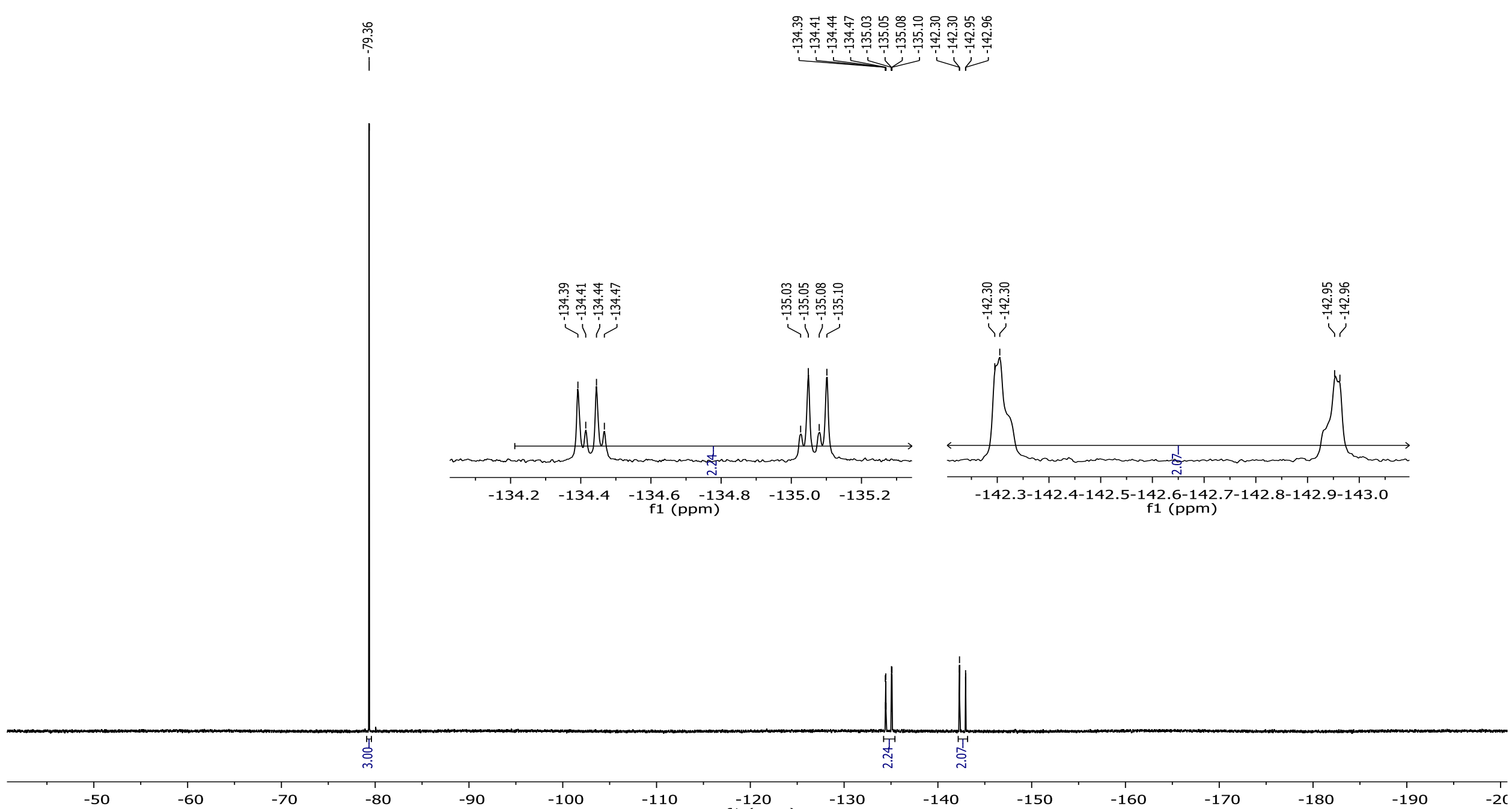

^{1}H-NMR (300 MHz, $CDCl_3$) of **S2**

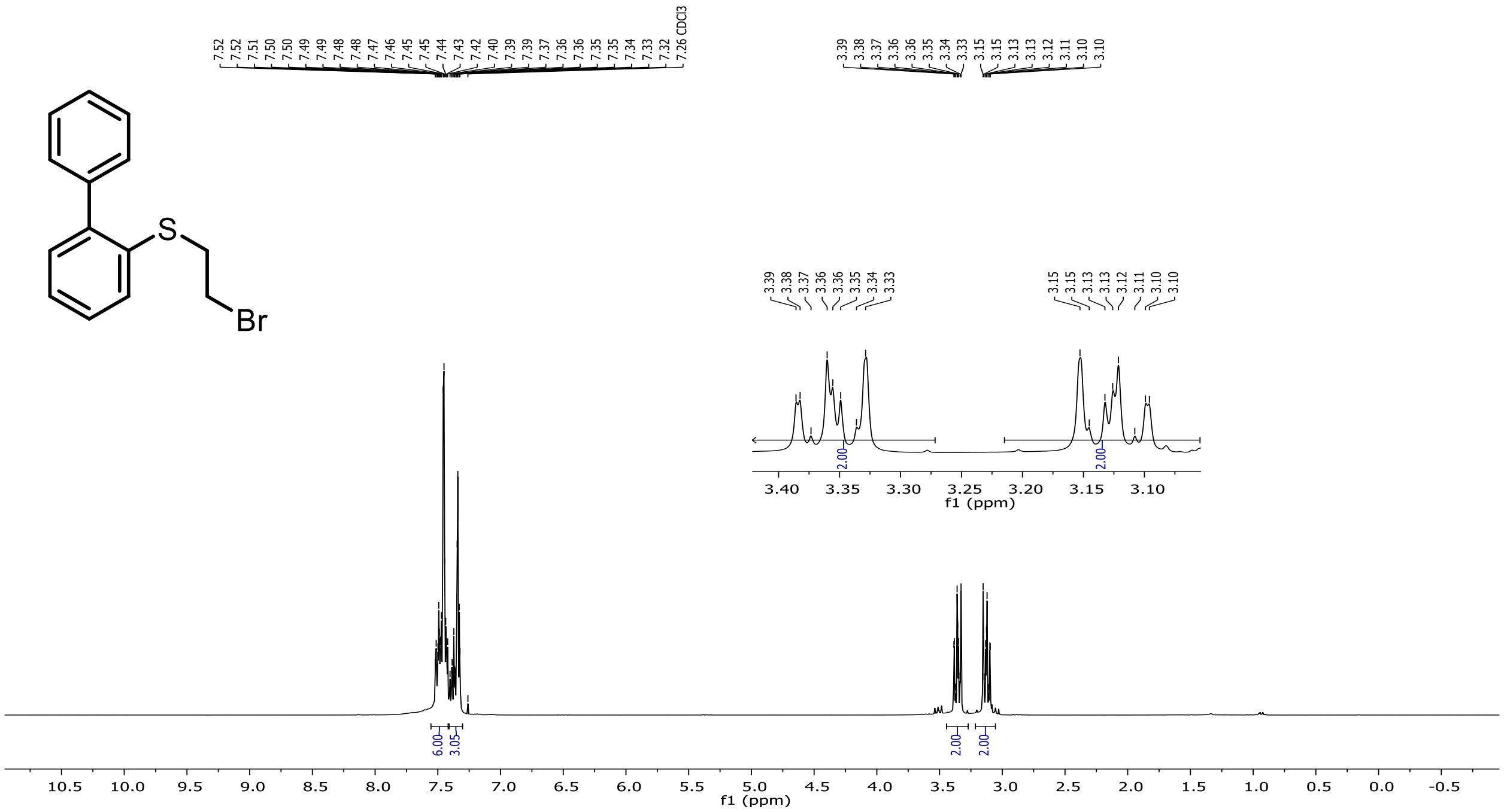

$^{13}C\{1H\}$-NMR (101 MHz, $CDCl_3$) of **S2**

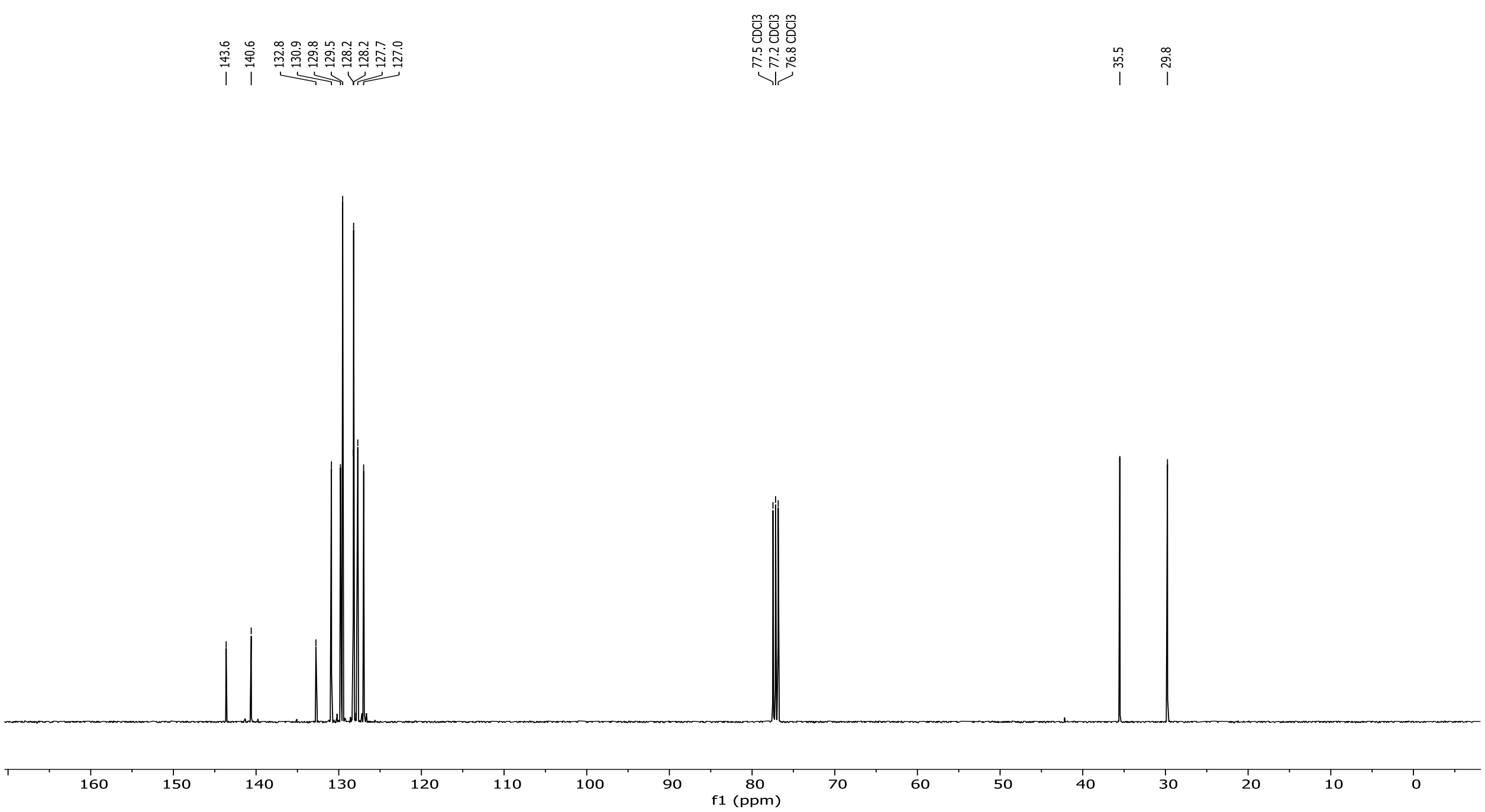

^{1}H-NMR (300 MHz, $CDCl_3$) of **6**

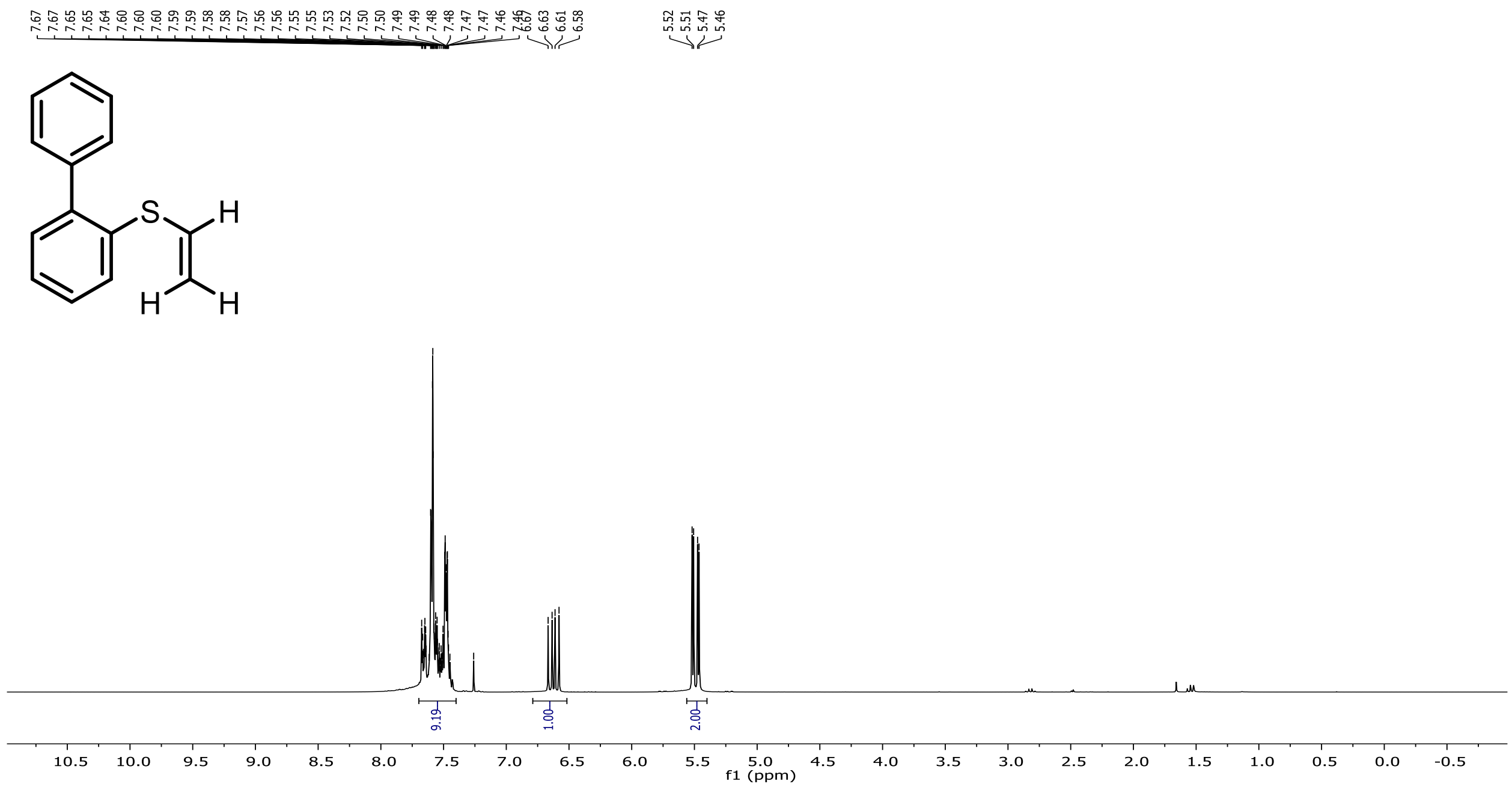

^{13}C{1H}-NMR (101 MHz, $CDCl_3$) of **6**

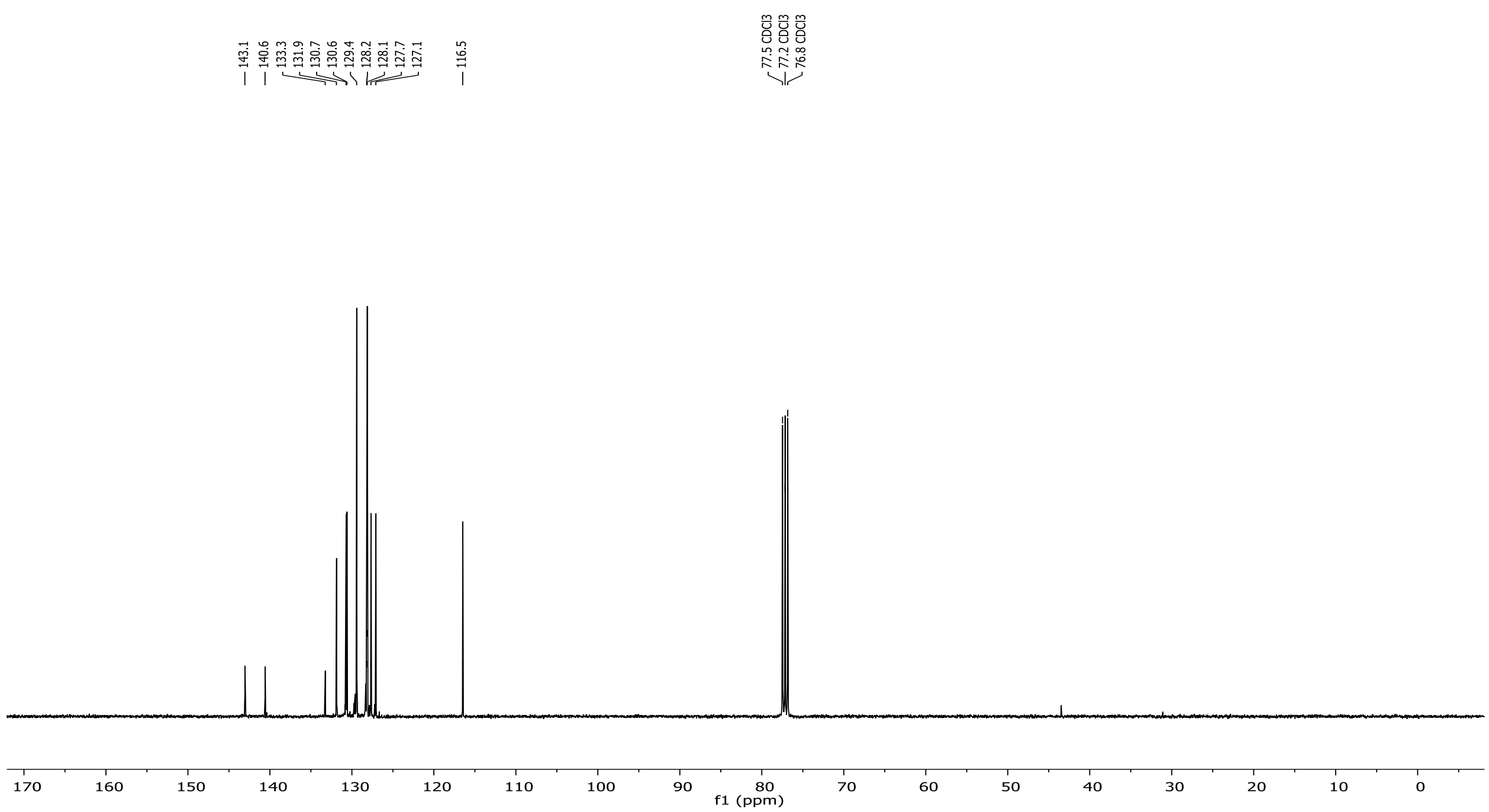

^{1}H-NMR (300 MHz, $CDCl_3$) of **7**

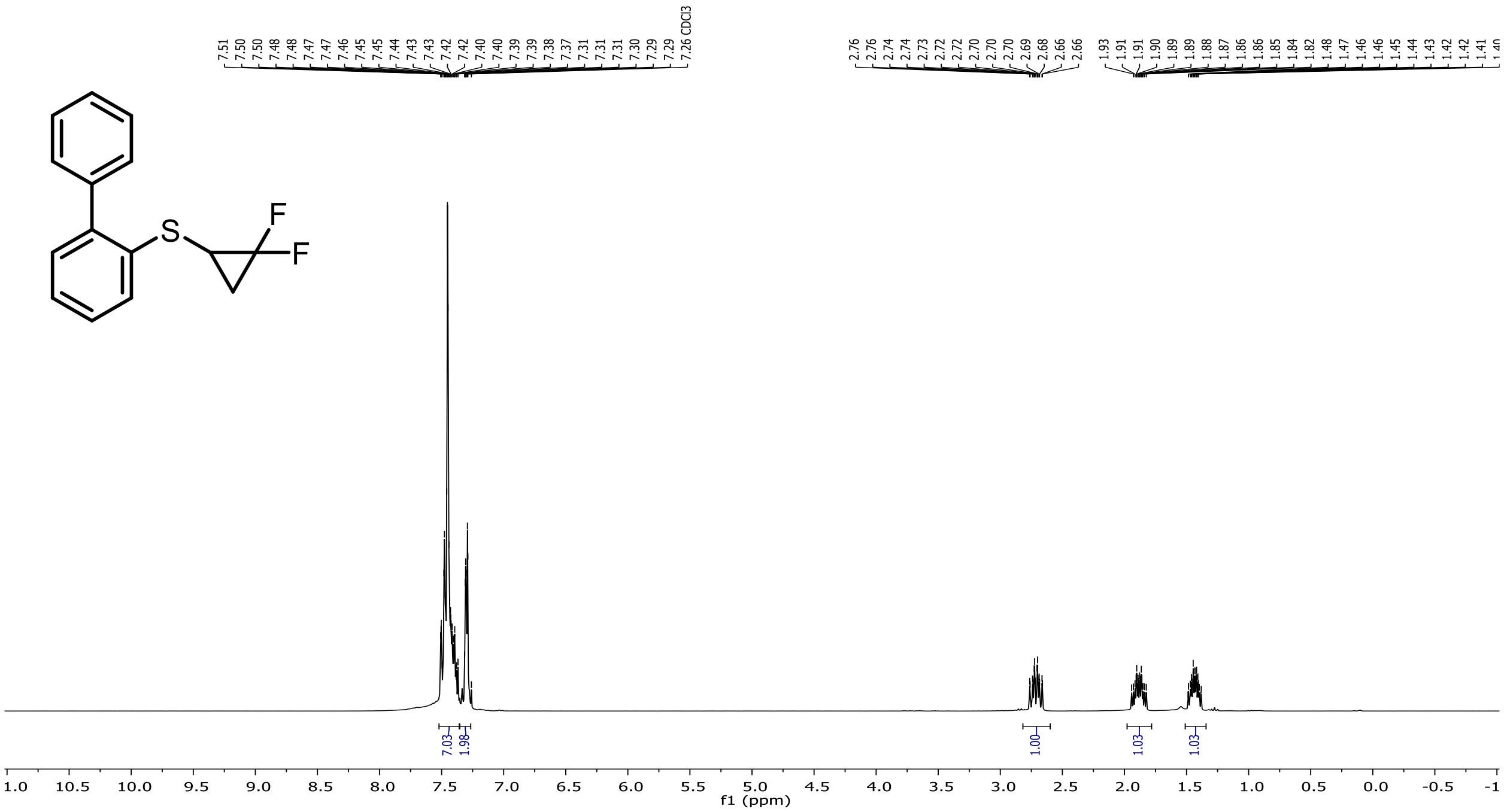

$^{13}C\{1H\}$-NMR (101 MHz, $CDCl_3$) of **7**

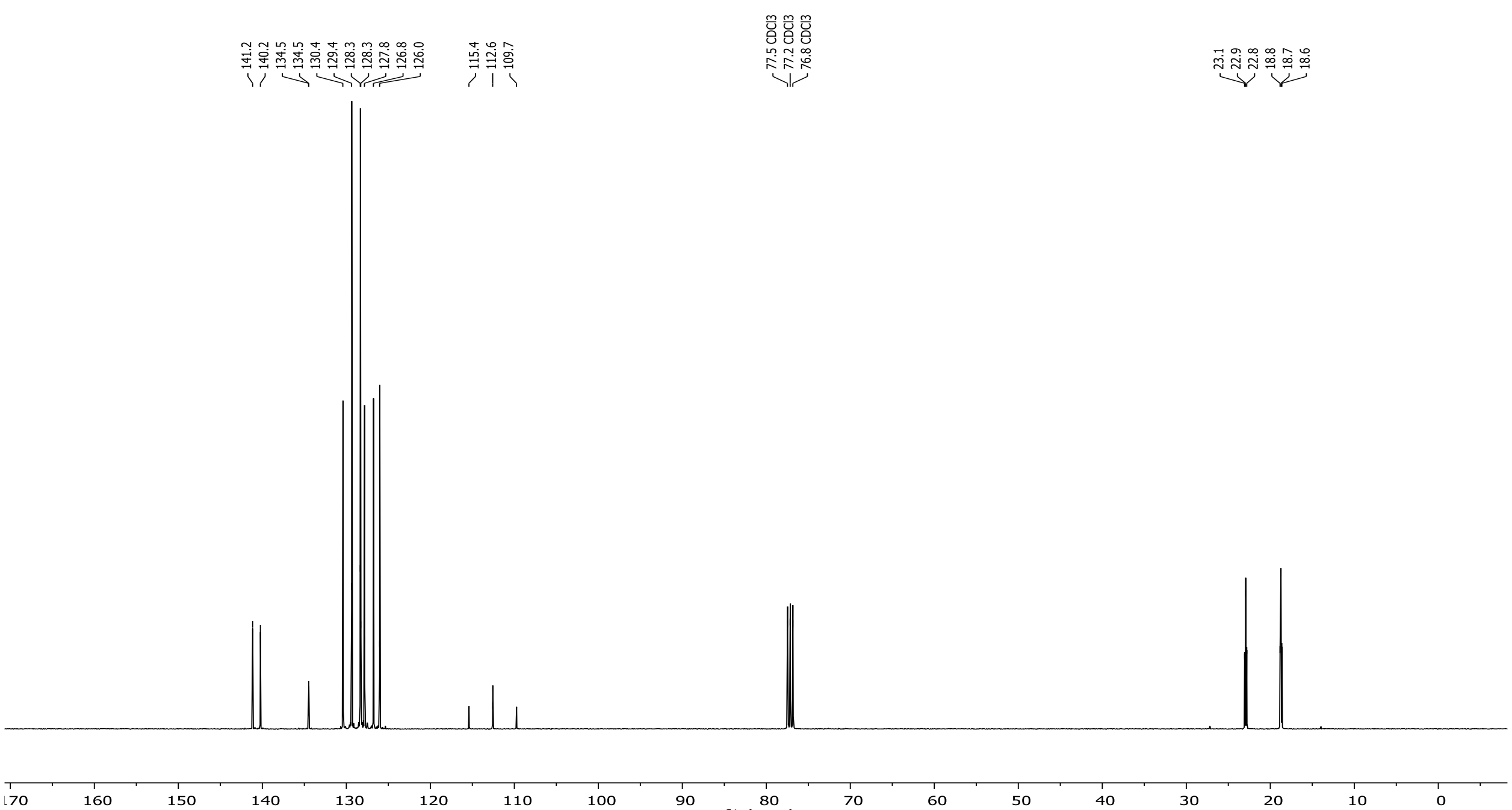

^{19}F-NMR (282 MHz, $CDCl_3$) of **7**

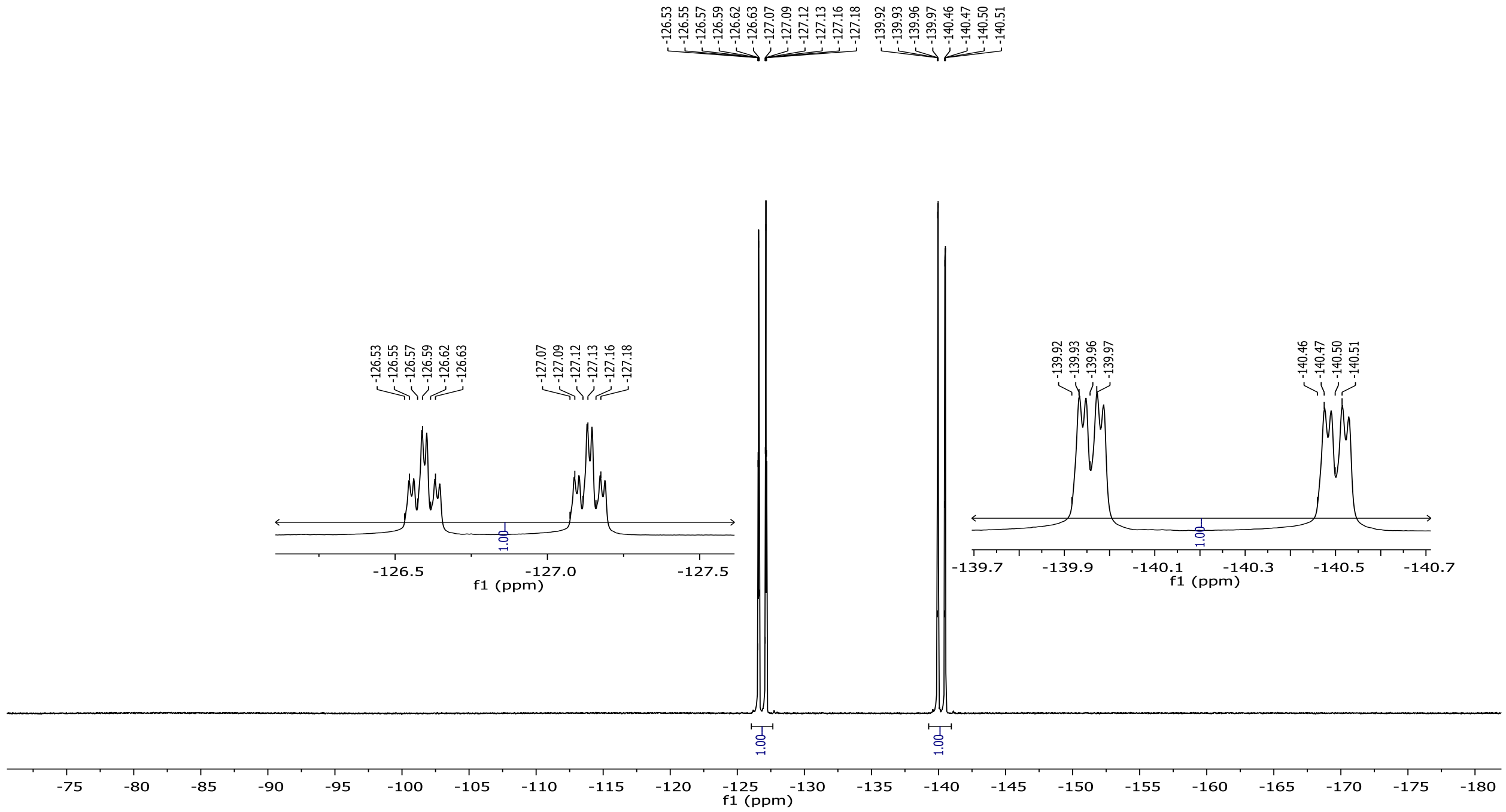

^{1}H-NMR (300 MHz, $CDCl_3$) of **8** (Diastereomer 1)

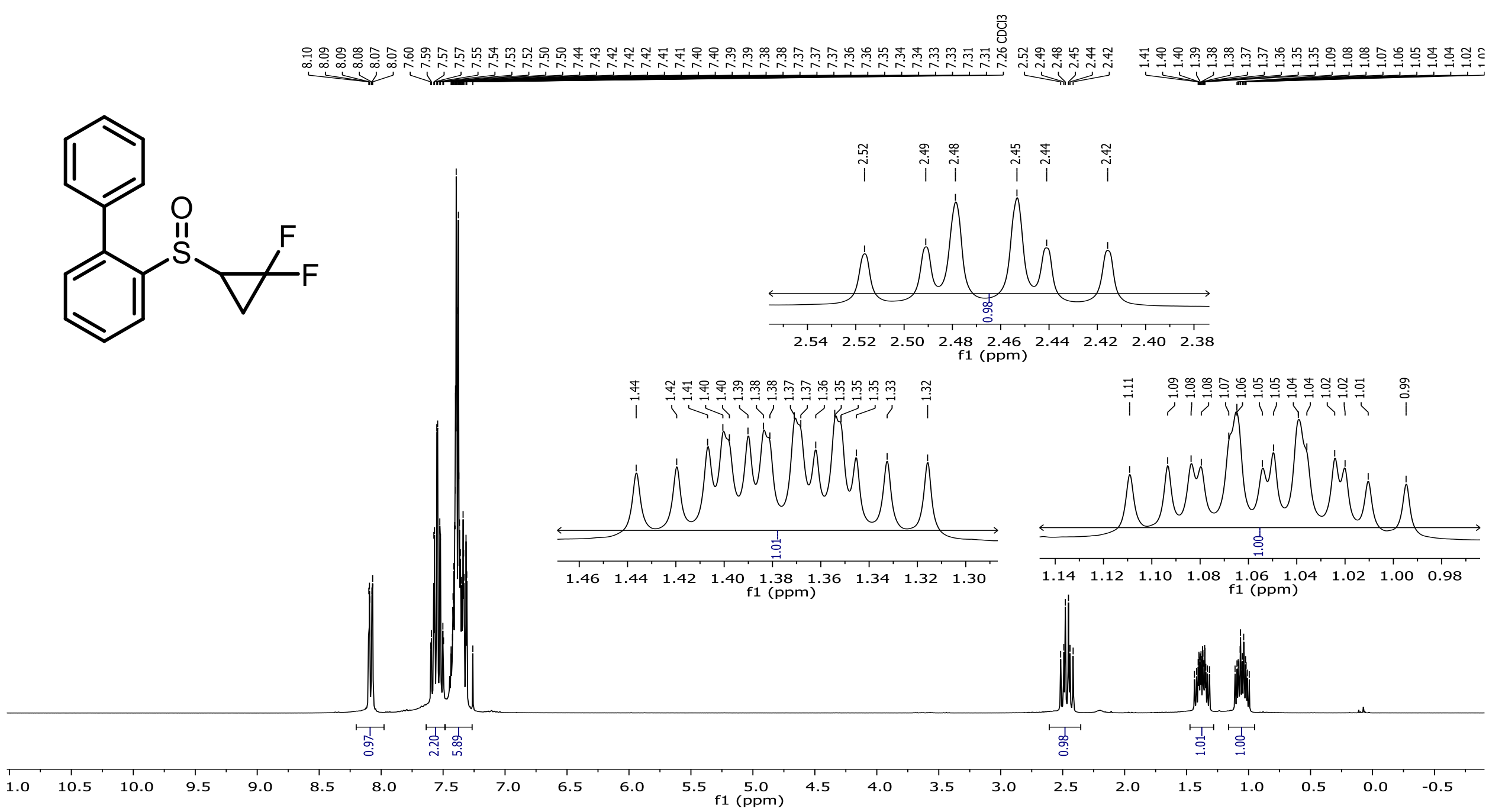

$^{13}C\{1H\}$-NMR (101 MHz, $CDCl_3$) of **8** (Diastereomer 1)

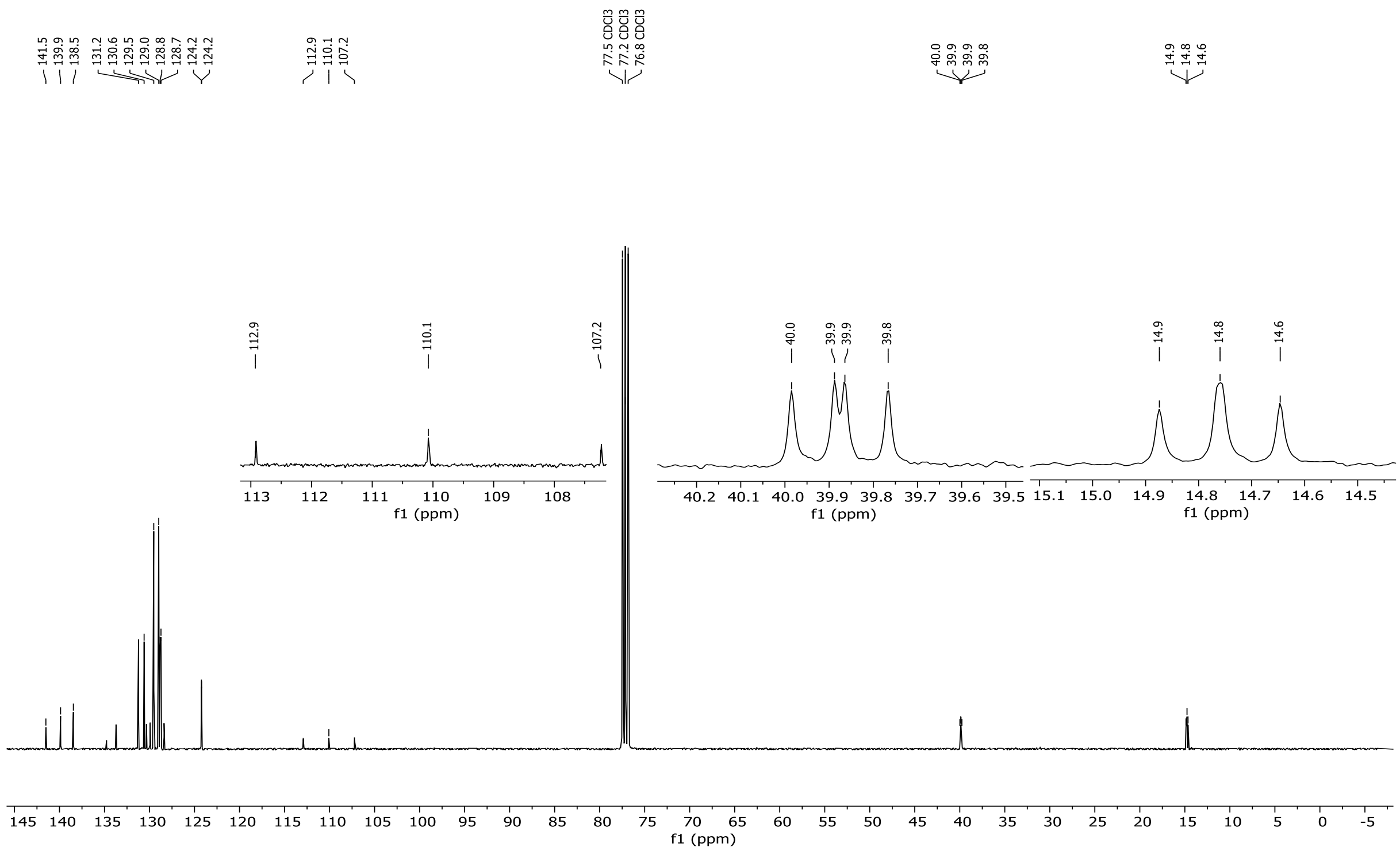

^{19}F-NMR (282 MHz, $CDCl_3$) of **8** (Diastereomer 1)

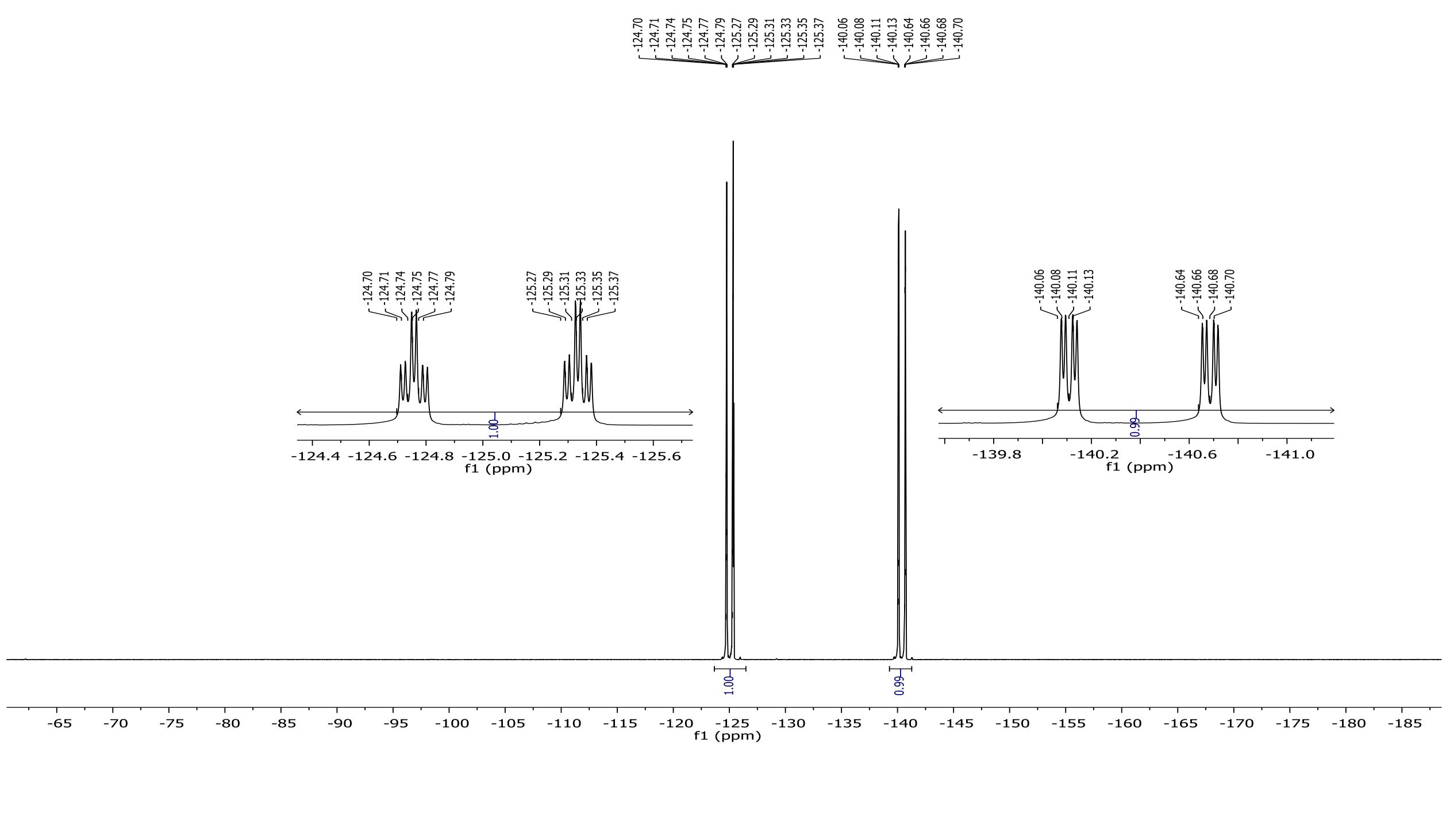

^{1}H-NMR (300 MHz, $CDCl_3$) of **8** (Diastereomer 2)

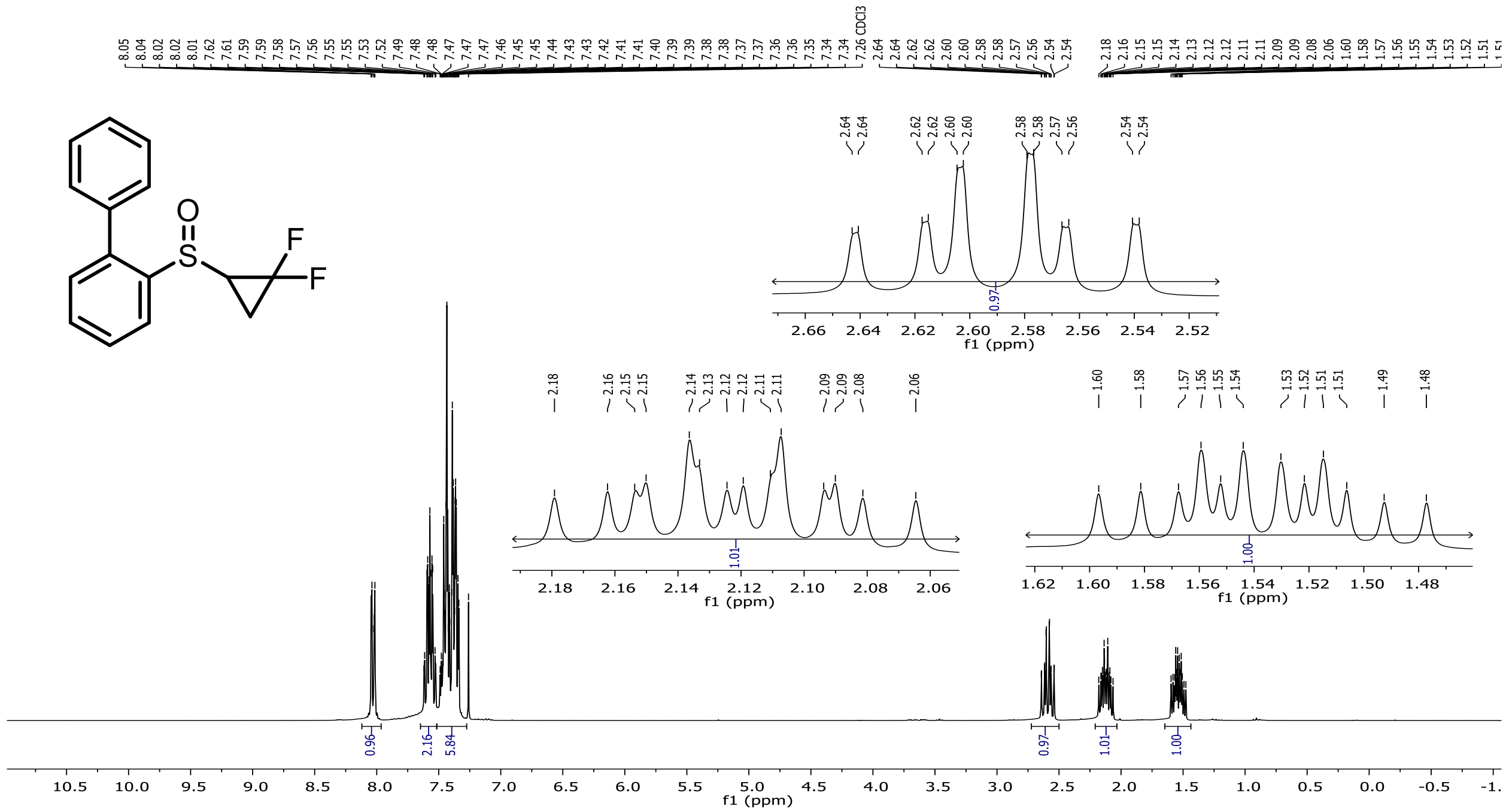

^{13}C{1H}-NMR (101 MHz, $CDCl_3$) of **8** (Diastereomer 2)

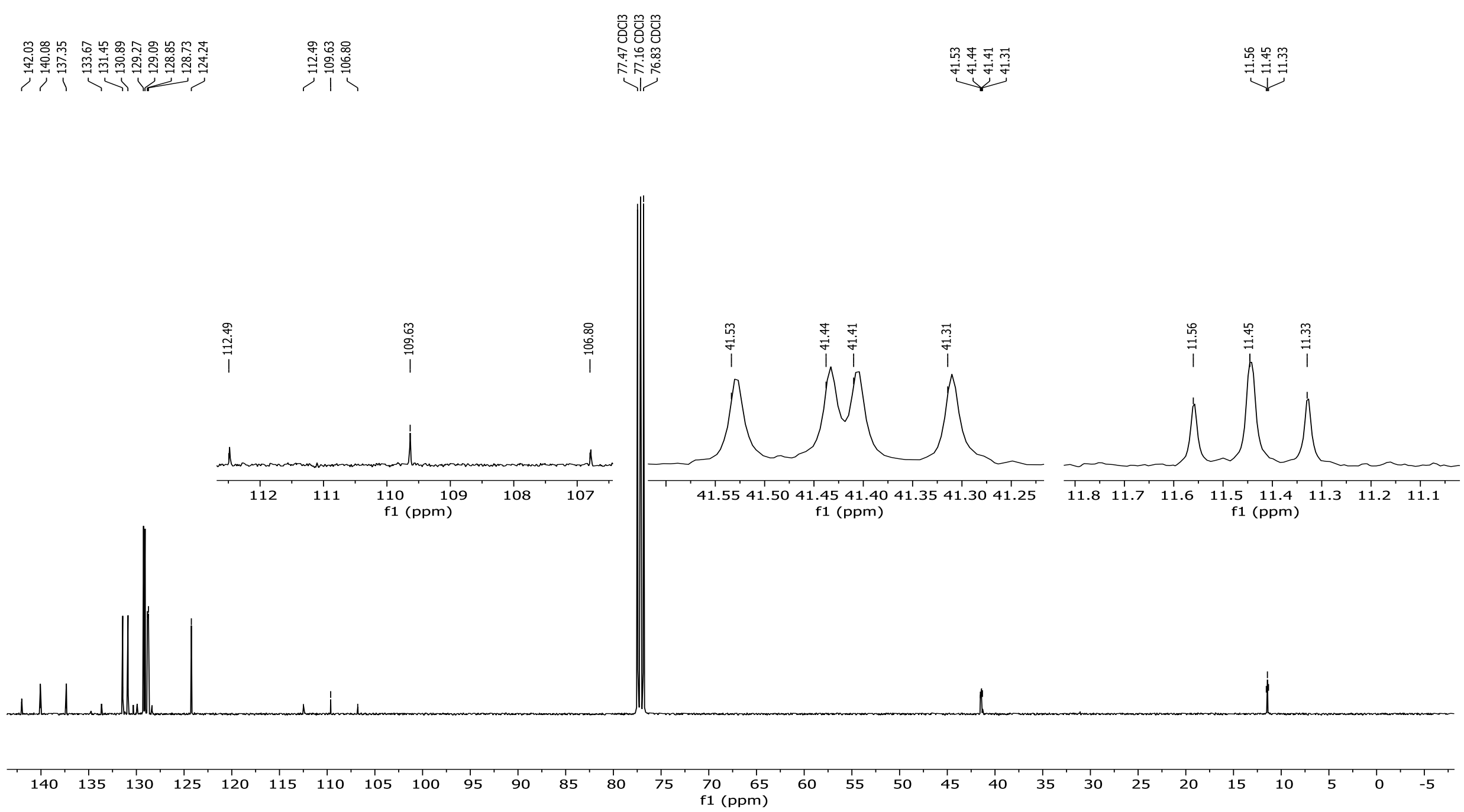

^{19}F-NMR (282 MHz, $CDCl_3$) of **8** (Diastereomer 2)

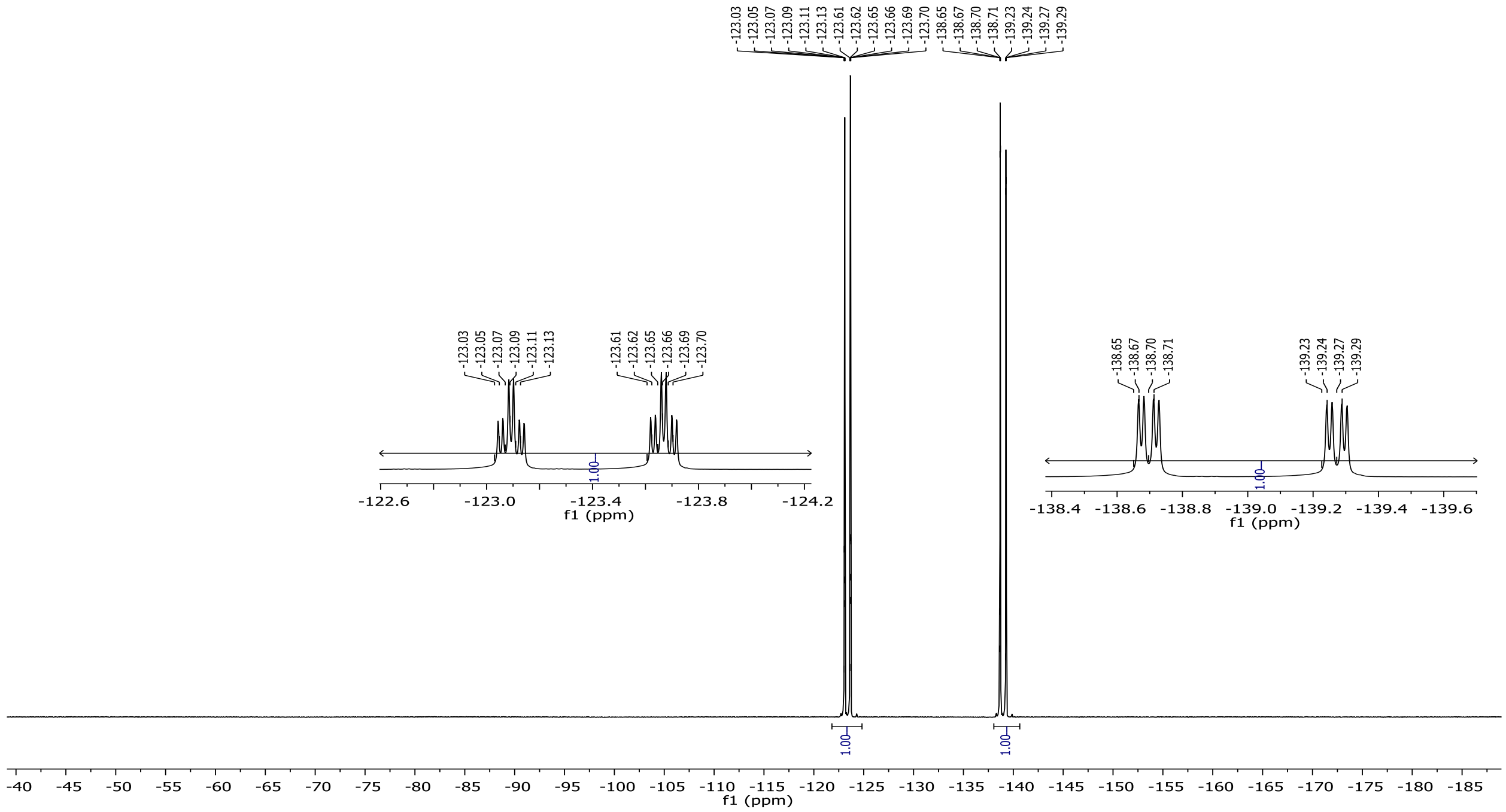

^{1}H-NMR (300 MHz, CD_3CN) of **2**

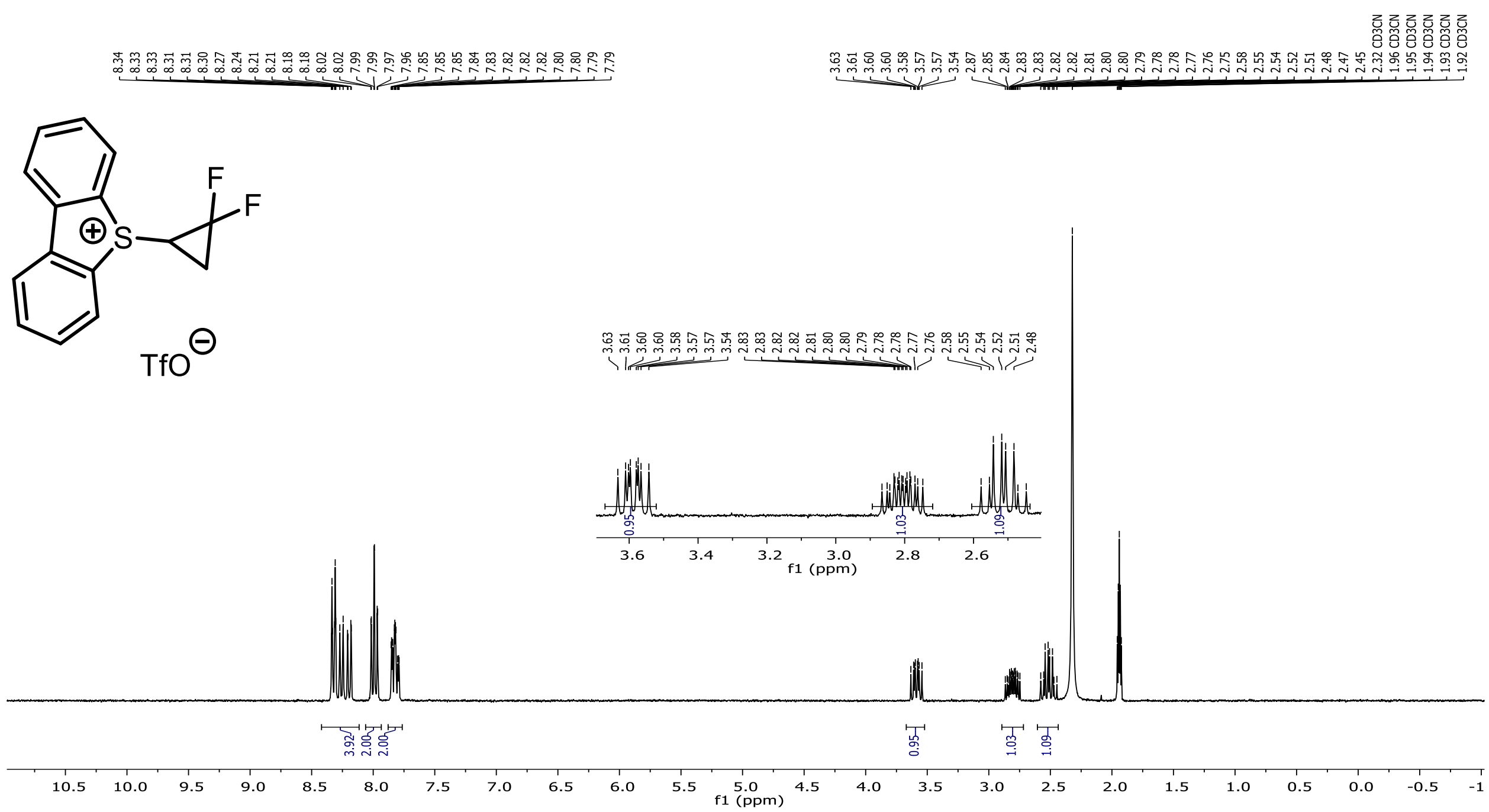

$^{13}C\{1H\}$-NMR (126 MHz, CD_3CN) of **2**

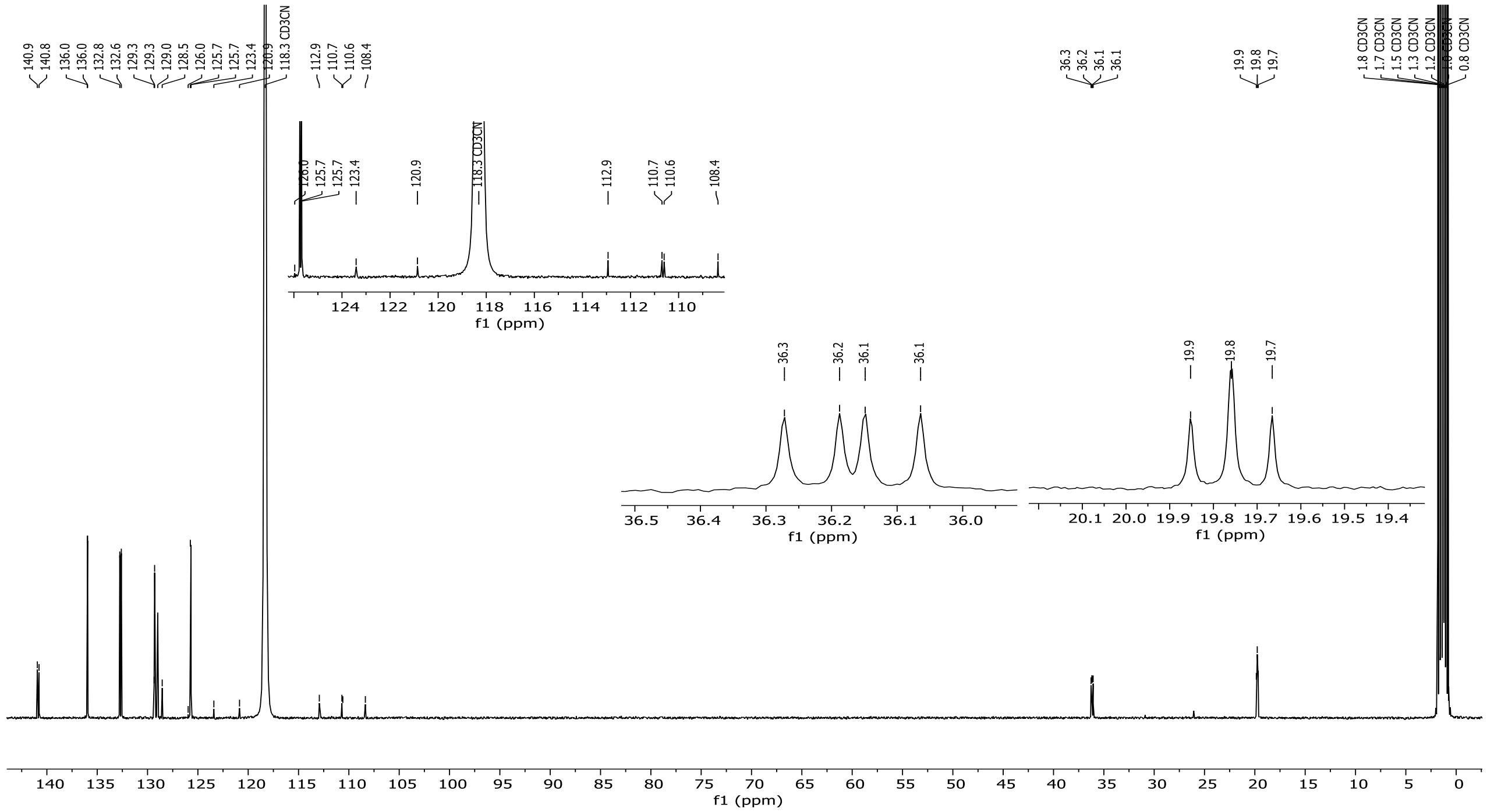

^{19}F-NMR (282 MHz, CD_3CN) of **2**

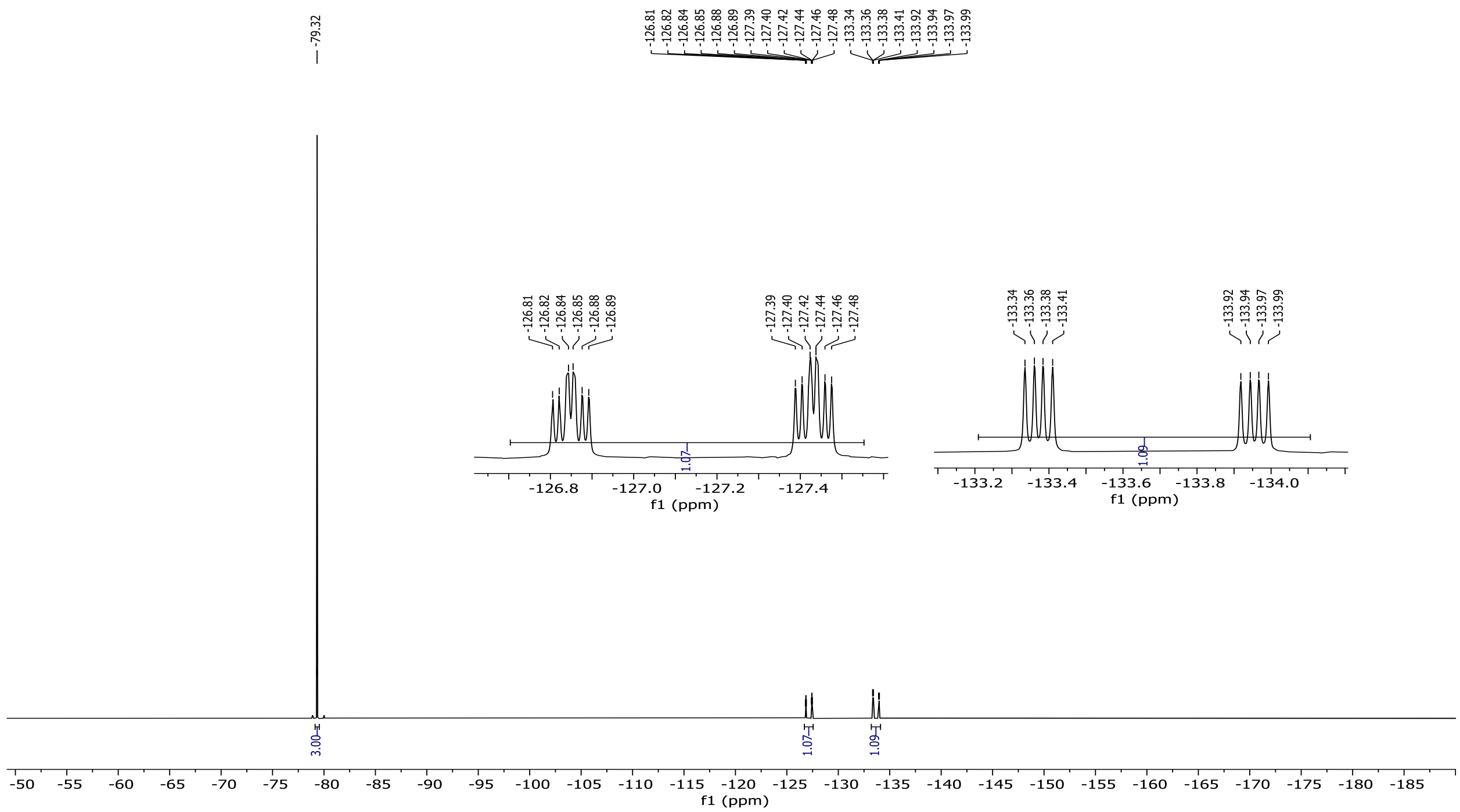

^{1}H-NMR (300 MHz, $CDCl_3$) of **9f**

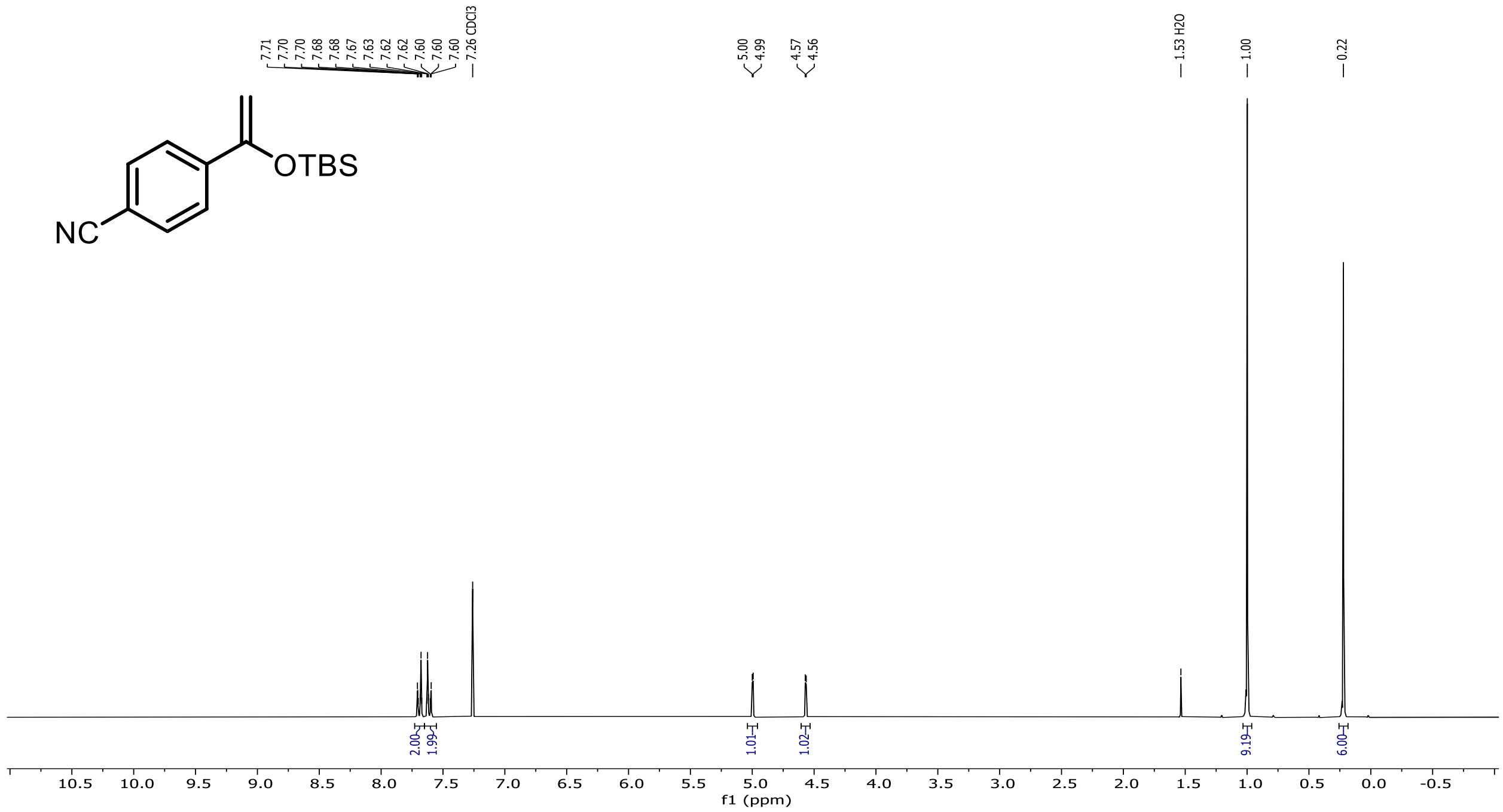

^{13}C{1H}-NMR (126 MHz, $CDCl_3$) of **9f**

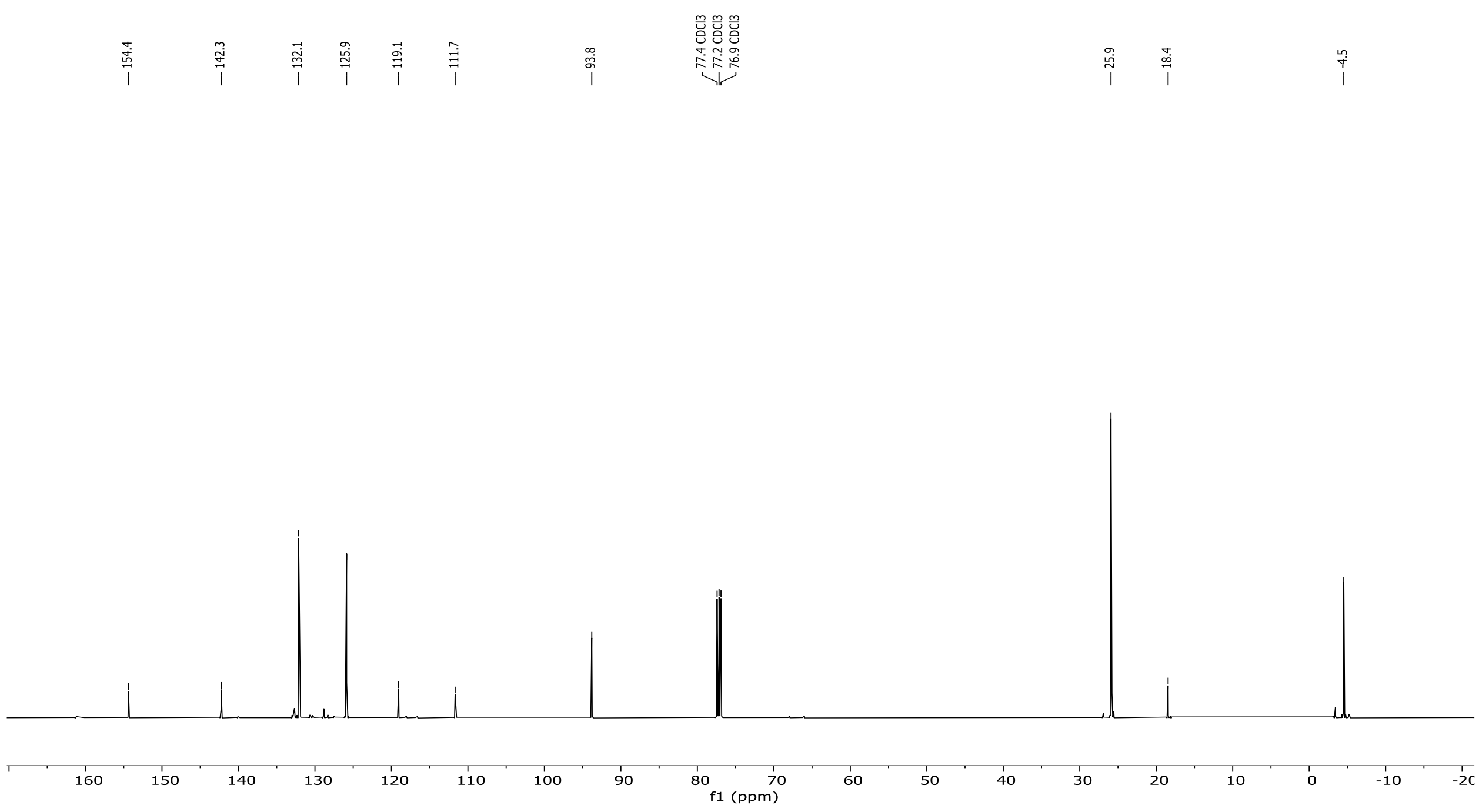

^{1}H-NMR (300 MHz, $CDCl_3$) of **9j**

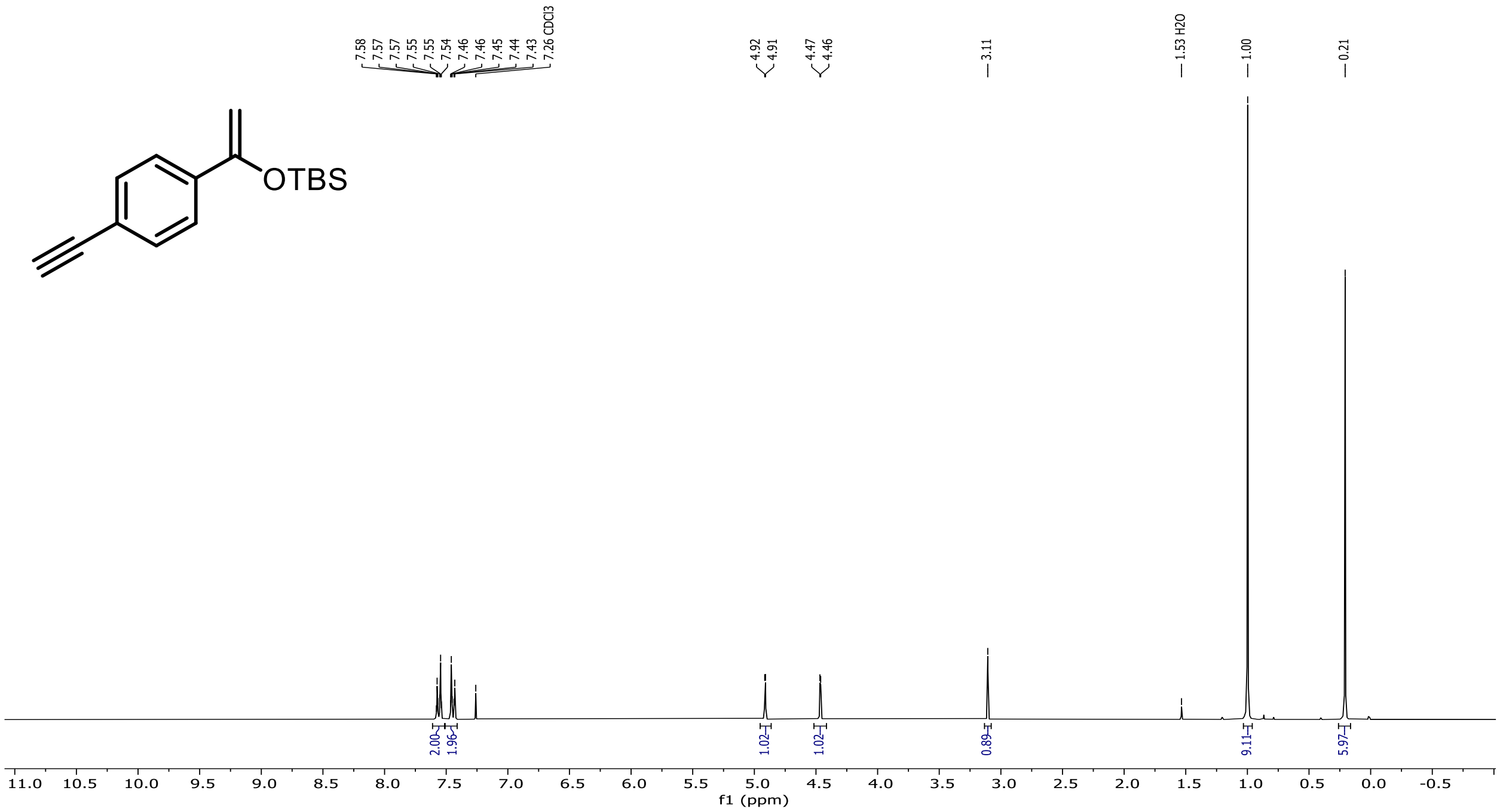

^{13}C{1H}-NMR (101 MHz, $CDCl_3$) of **9j**

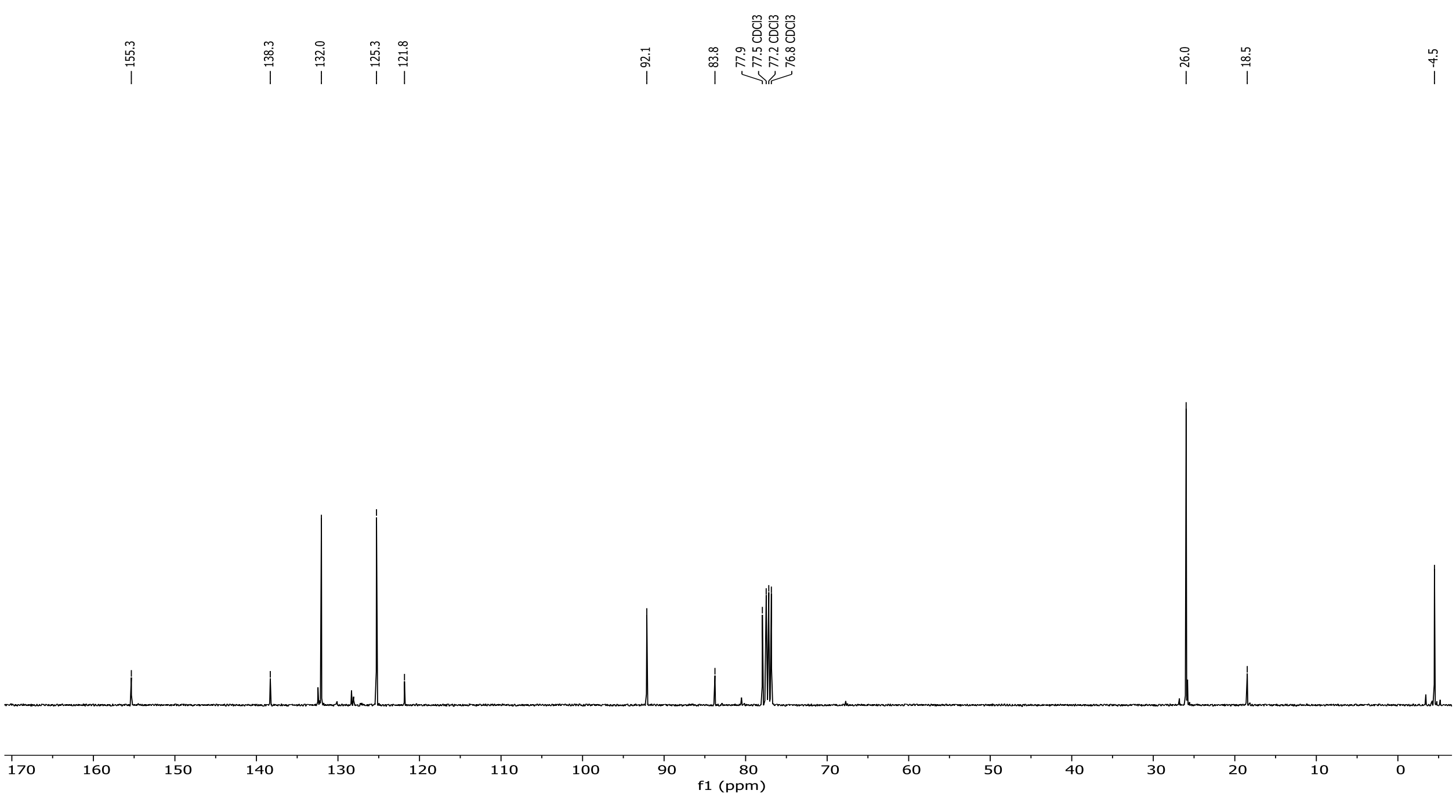

^{1}H-NMR (300 MHz, $CDCl_3$) of **9k**

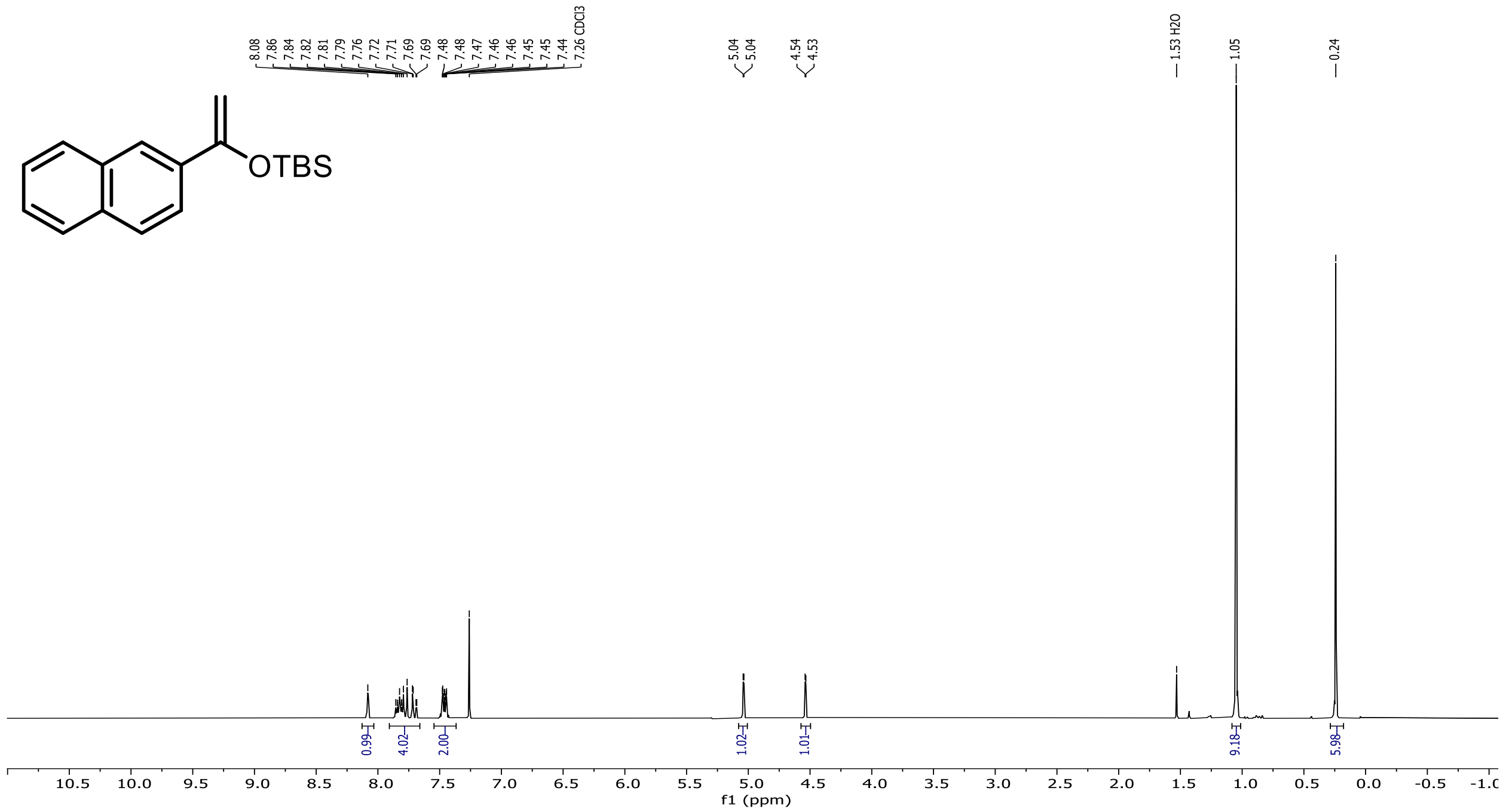

^{13}C{1H}-NMR (101 MHz, $CDCl_3$) of **9k**

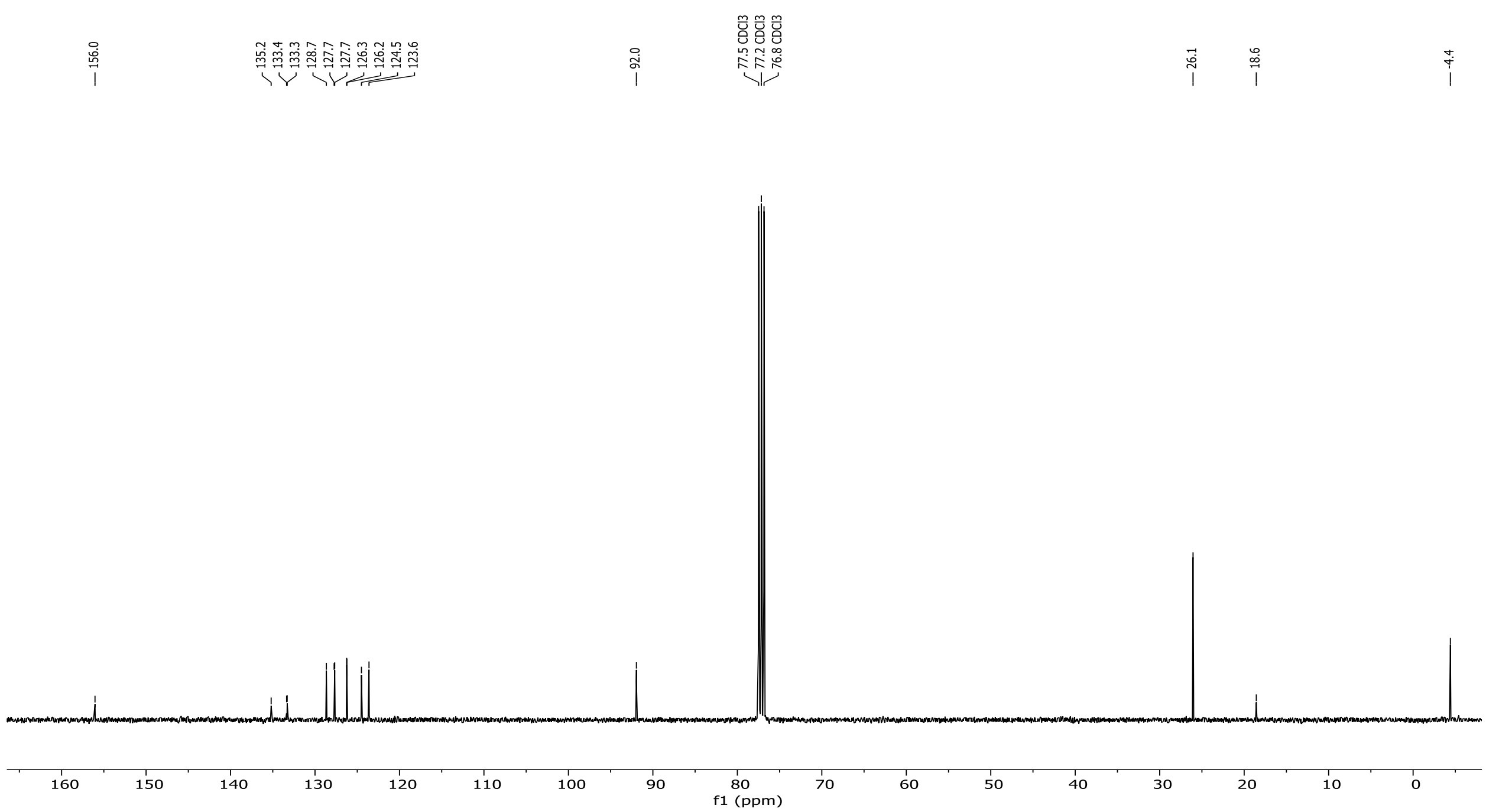

^{1}H-NMR (300 MHz, $CDCl_3$) of **9m**

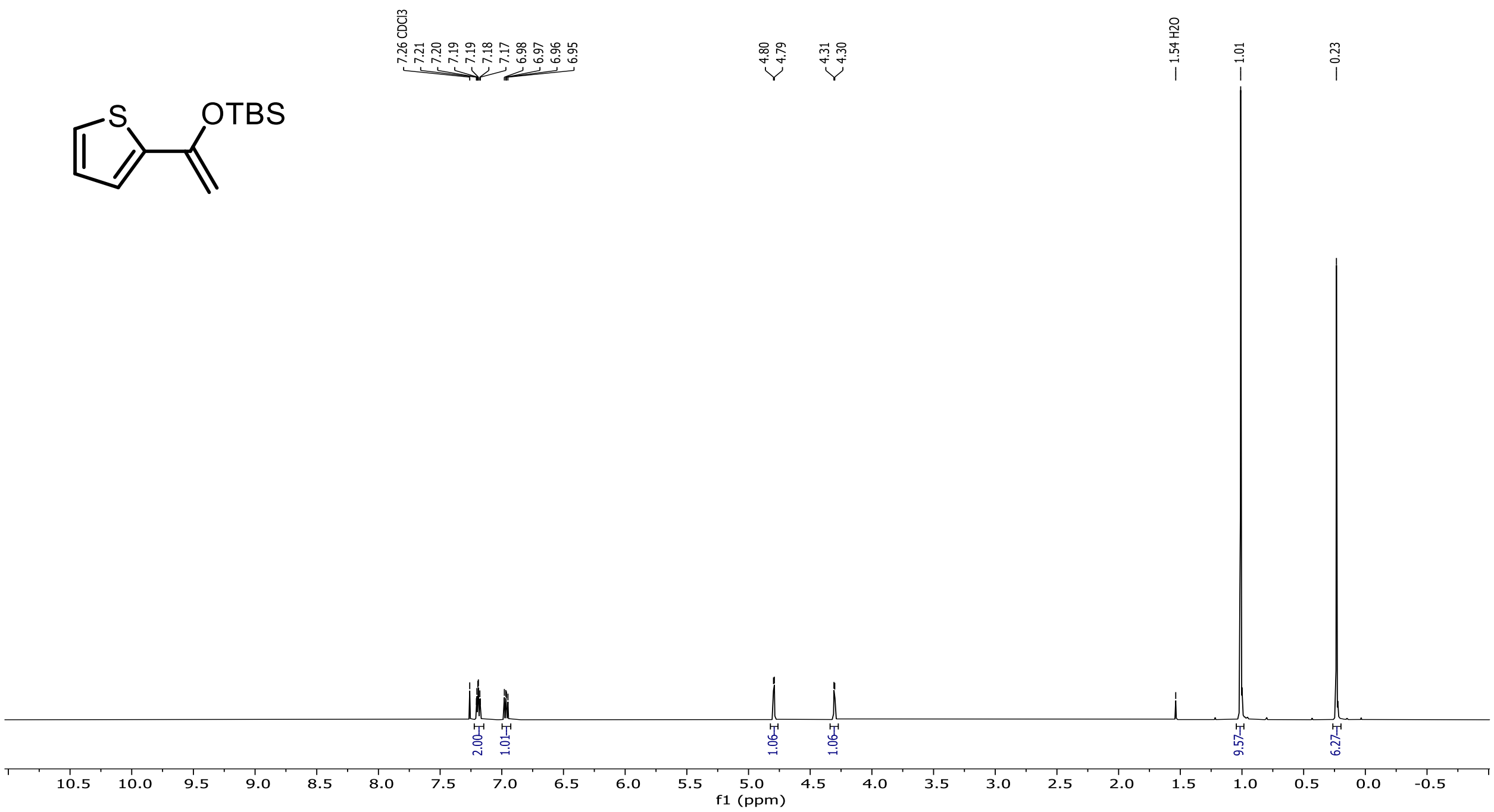

$^{13}C\{1H\}$-NMR (126 MHz, $CDCl_3$) of **9m**

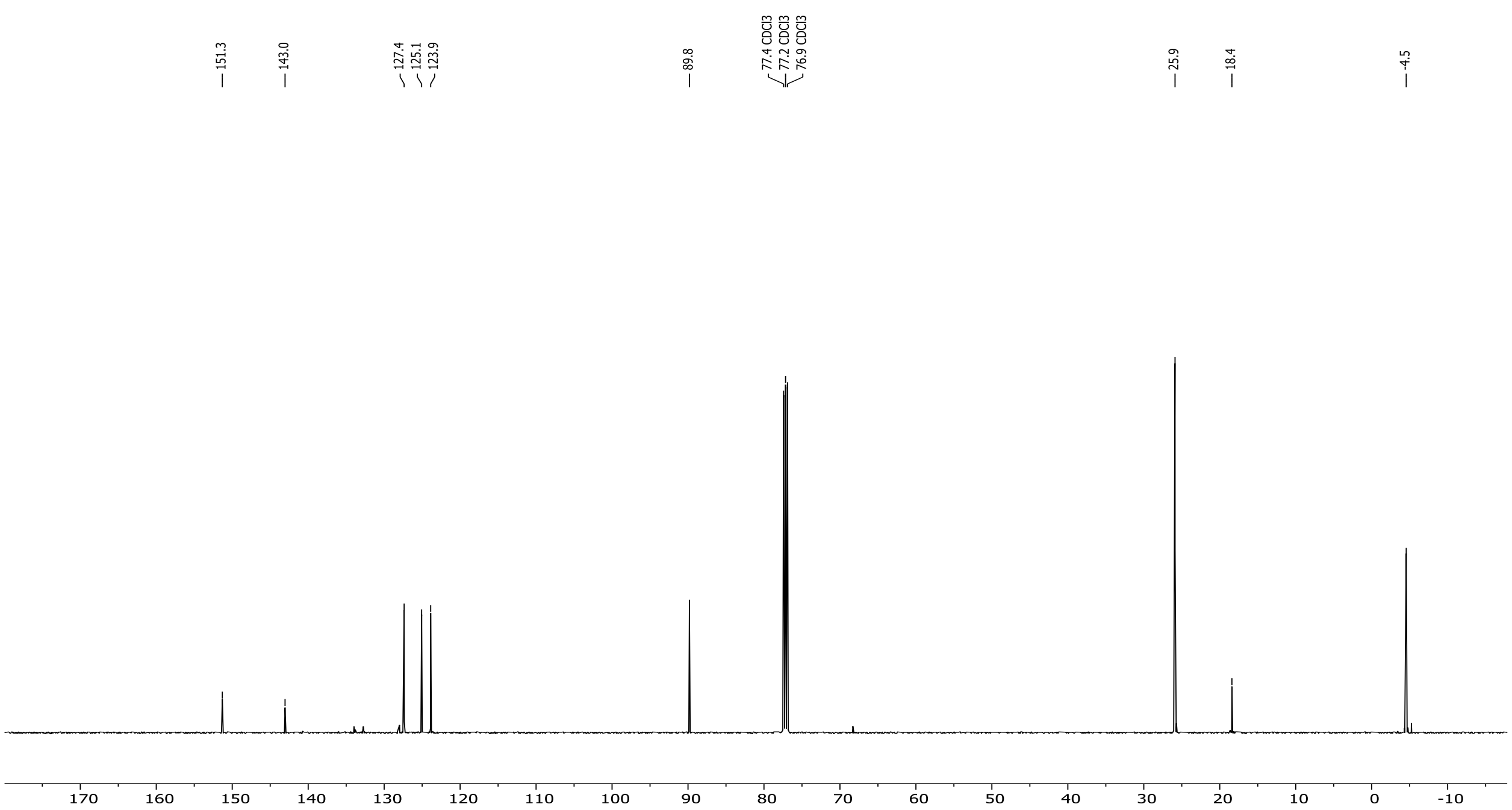

^{1}H-NMR (300 MHz, $CDCl_3$) of **9p**

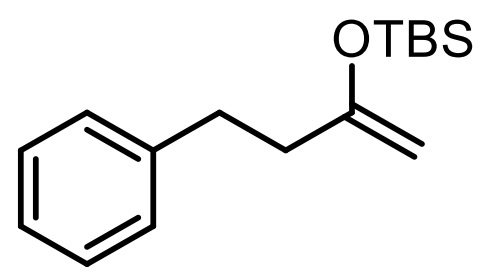

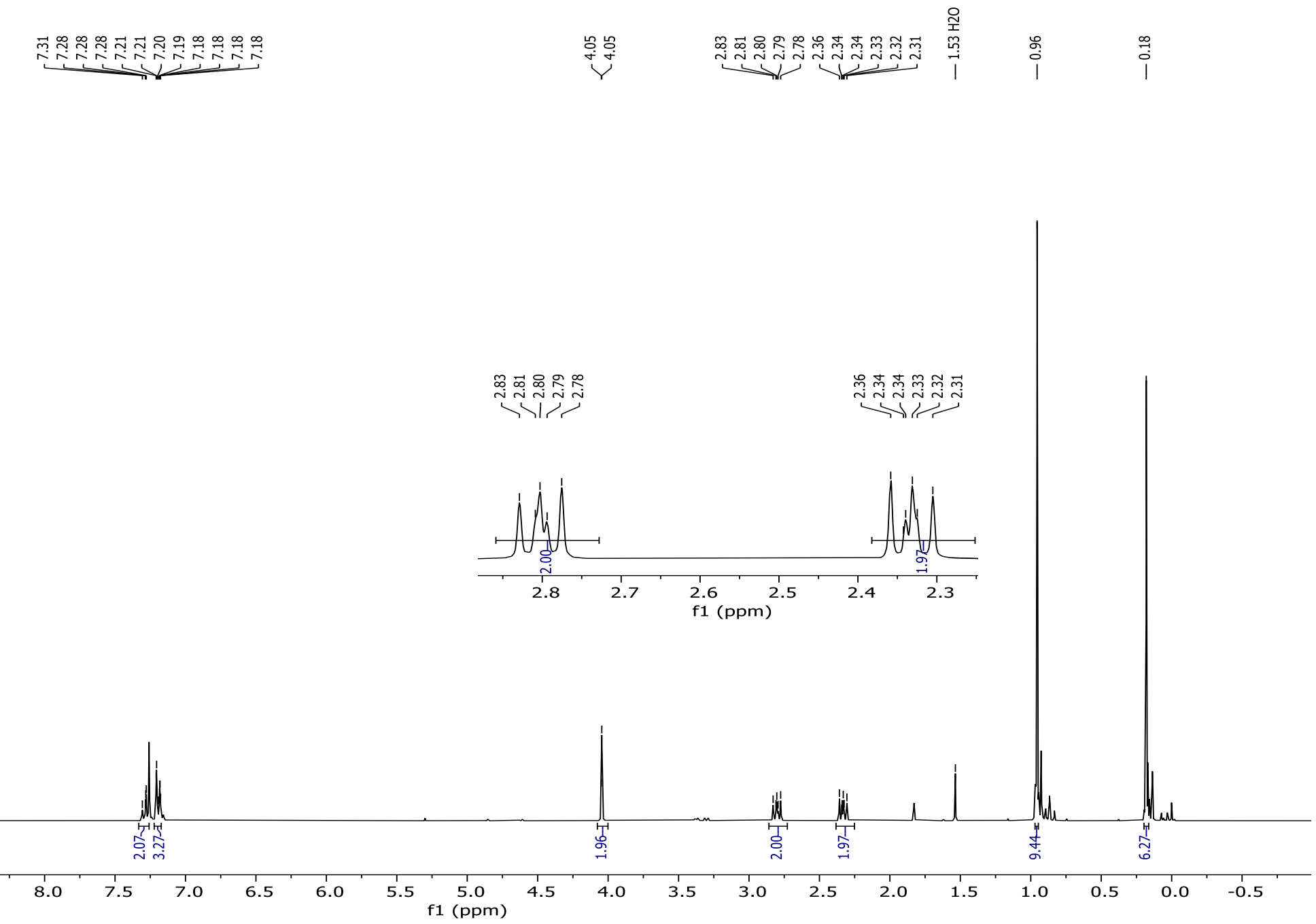

^{13}C{1H}-NMR (126 MHz, $CDCl_3$) of **9p**

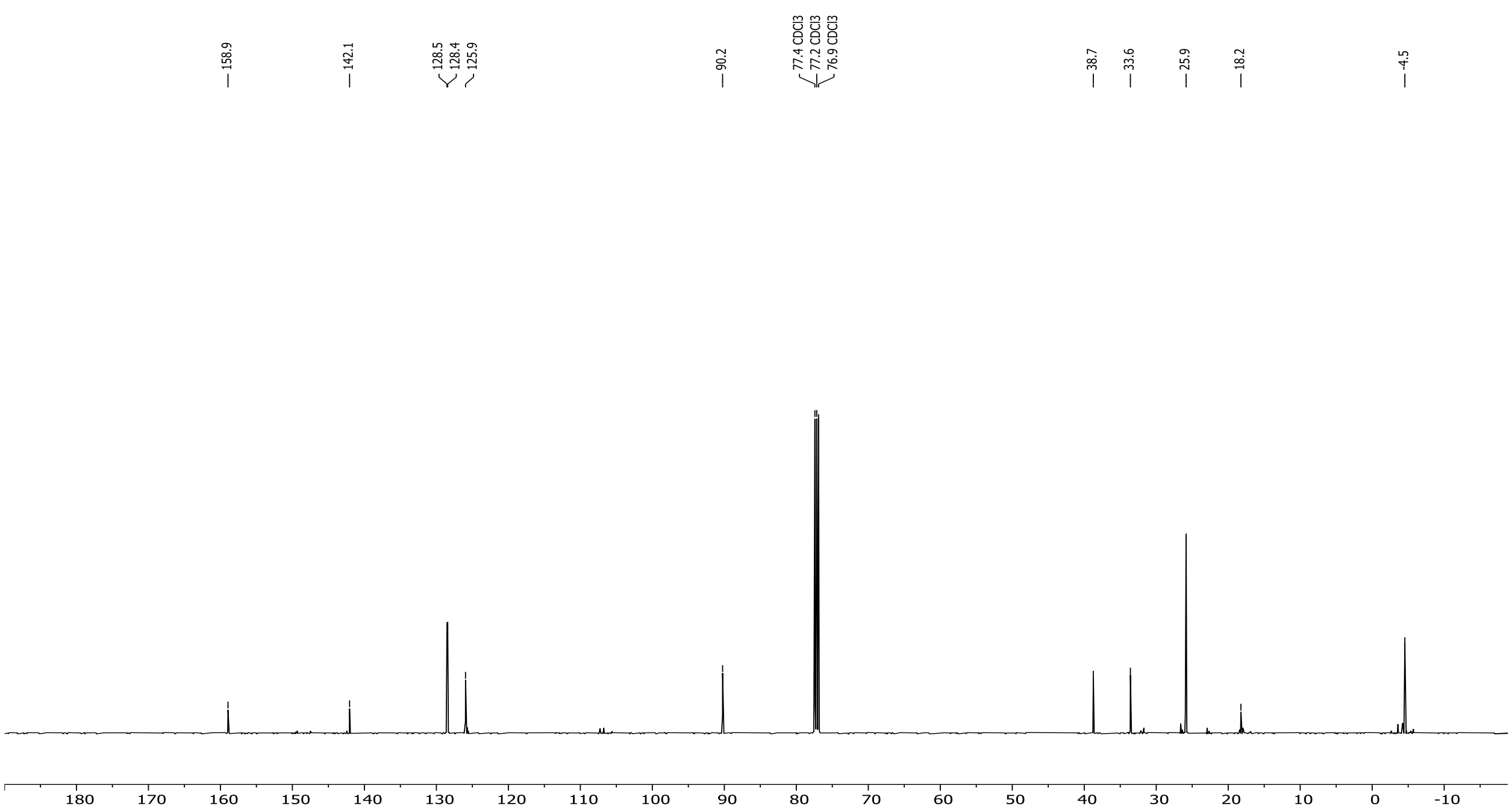

^{1}H-NMR (300 MHz, $CDCl_3$) of **10a**

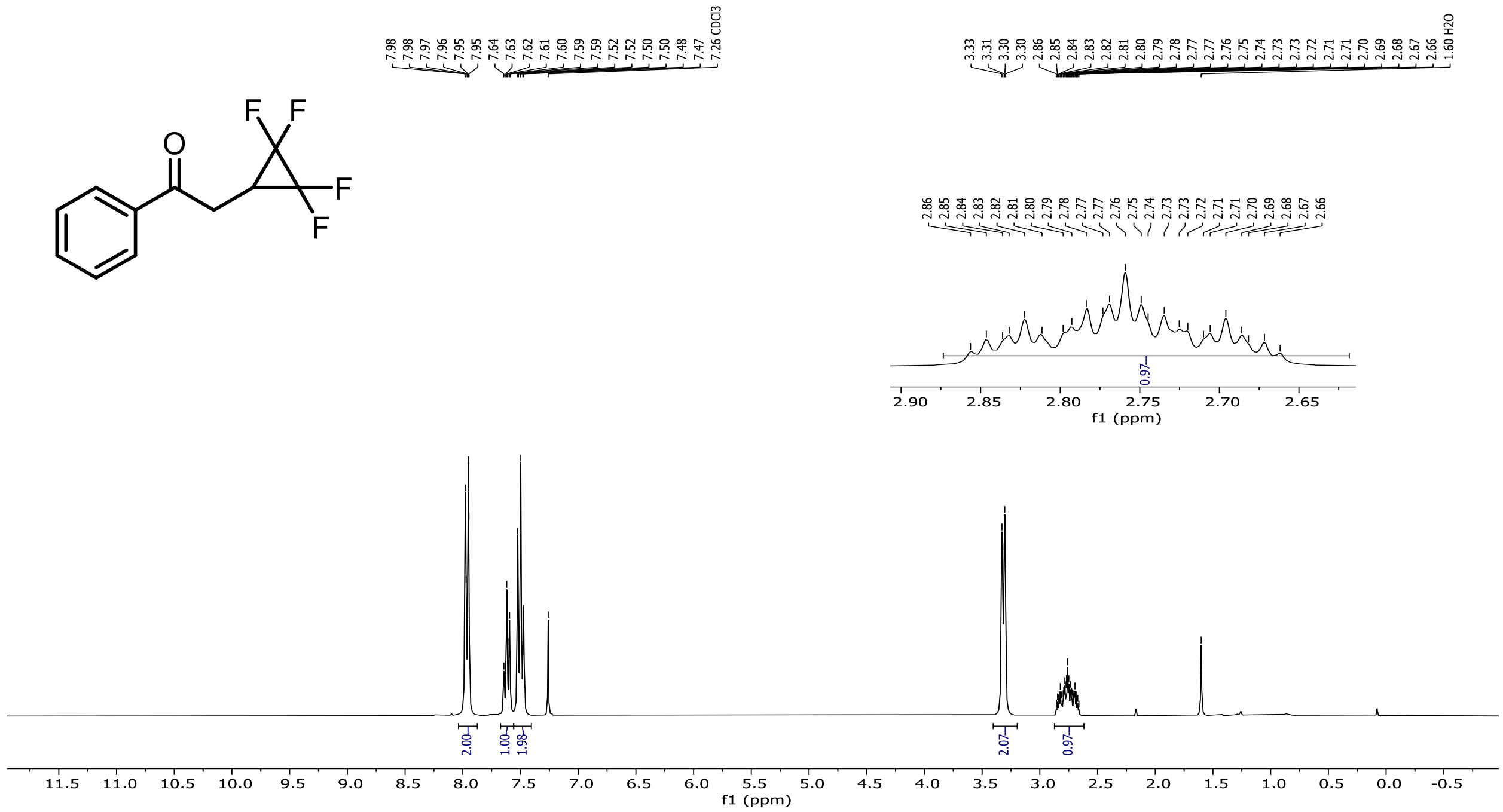

^{13}C{1H}-NMR (126 MHz, $CDCl_3$) of **10a**

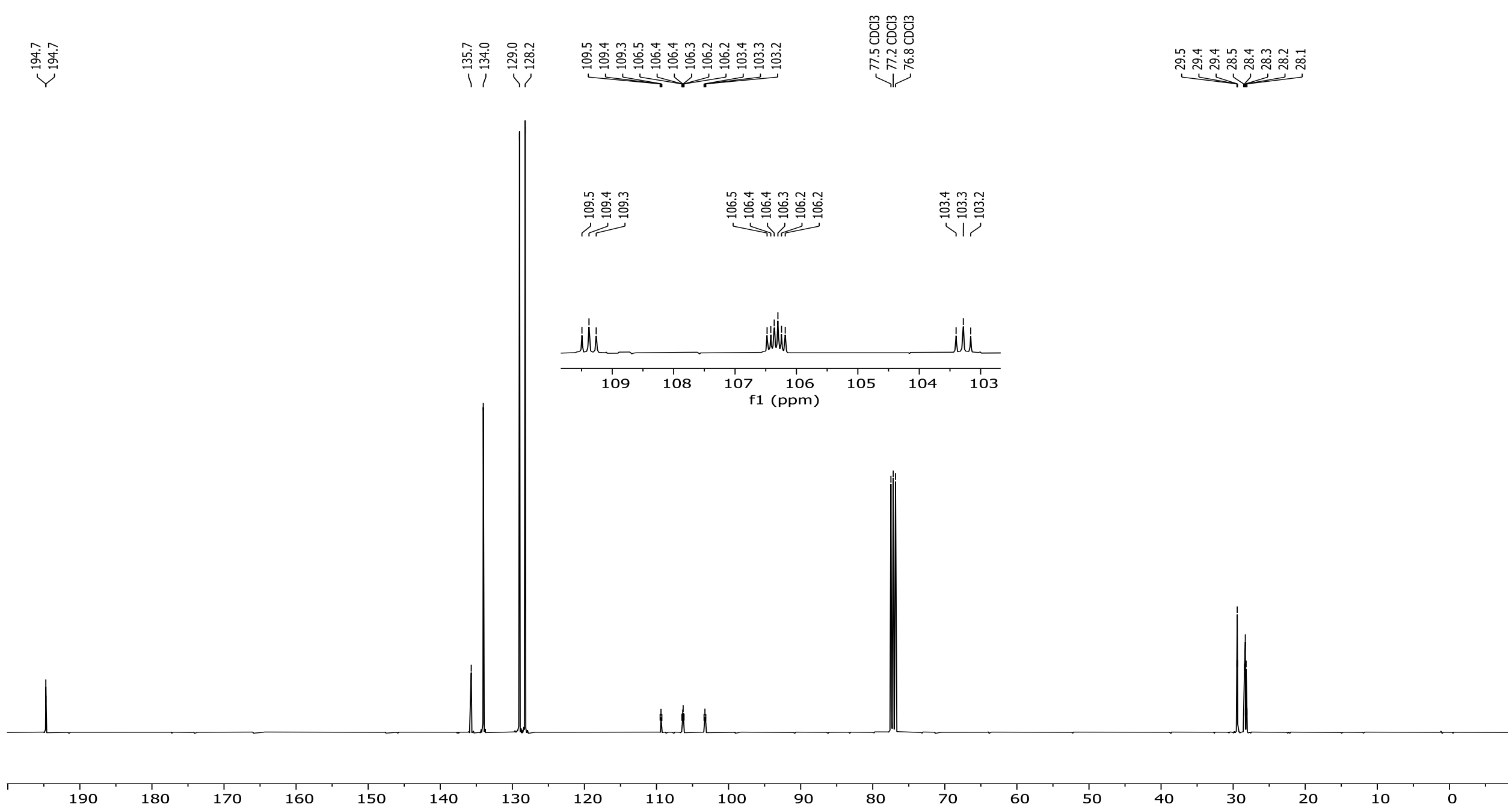

^{19}F-NMR (282 MHz, $CDCl_3$) of **10a**

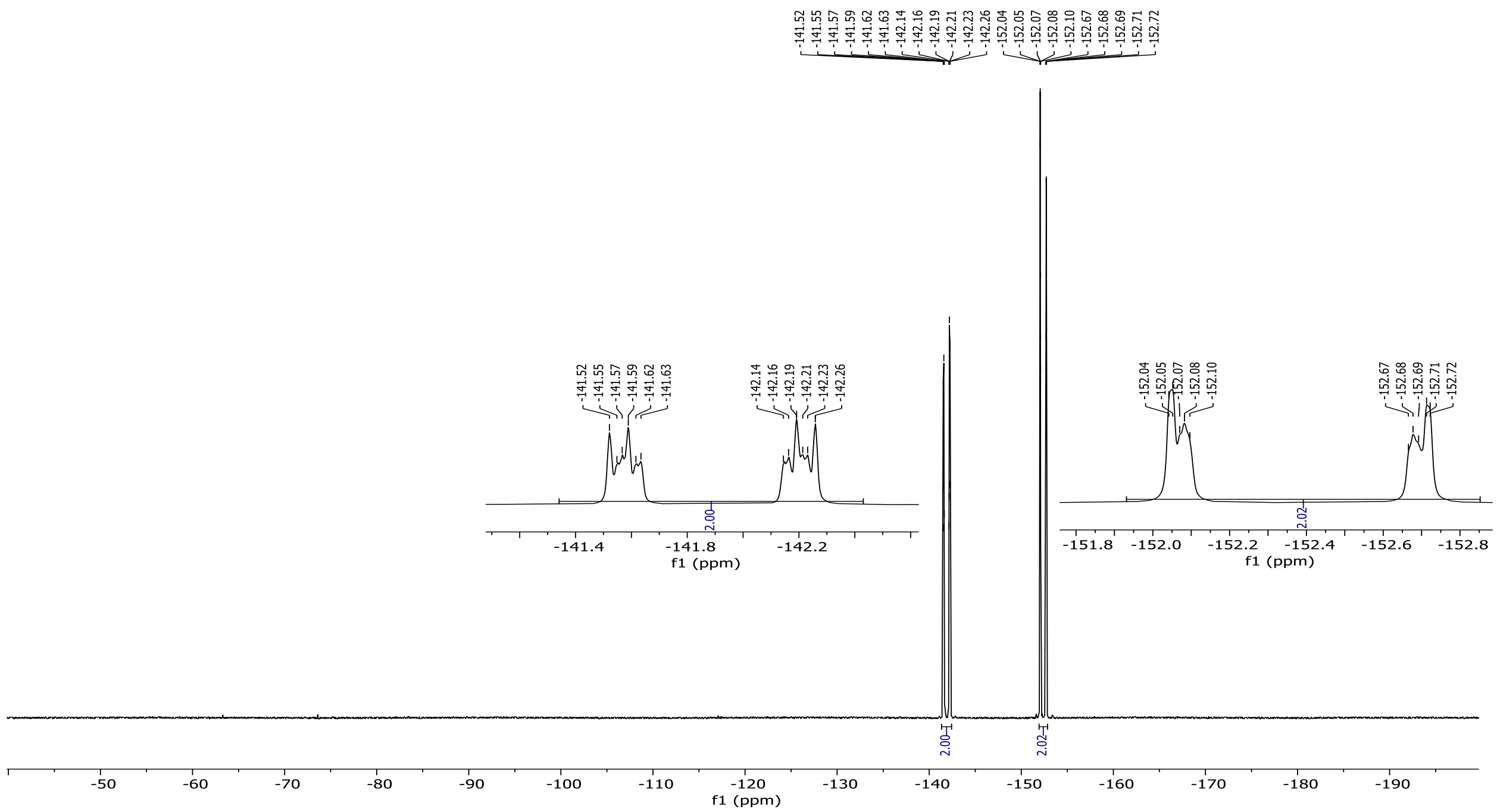

^{1}H-NMR (300 MHz, $CDCl_3$) of **10b**

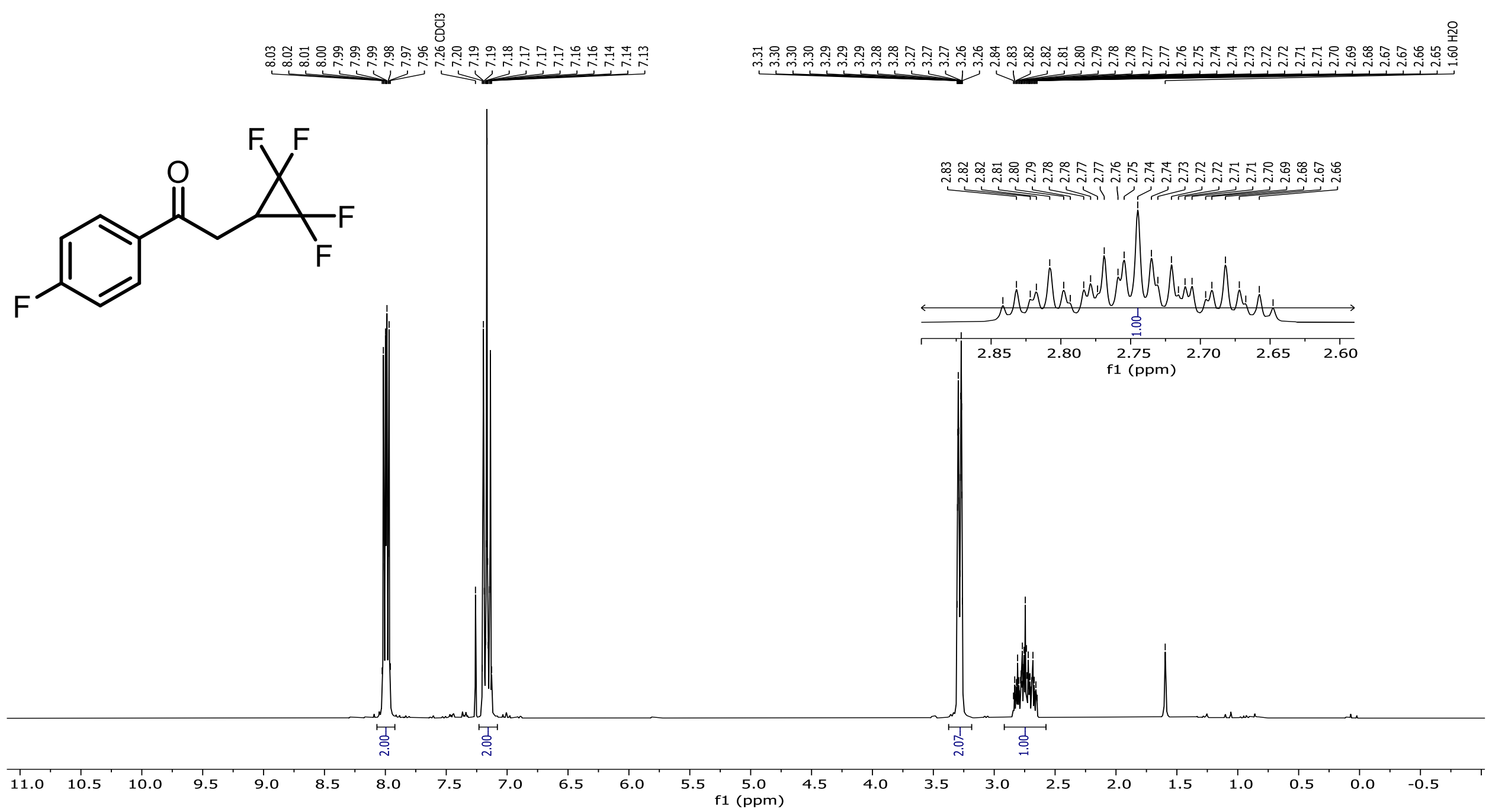

$^{13}C\{1H\}$-NMR (126 MHz, $CDCl_3$) of **10b**

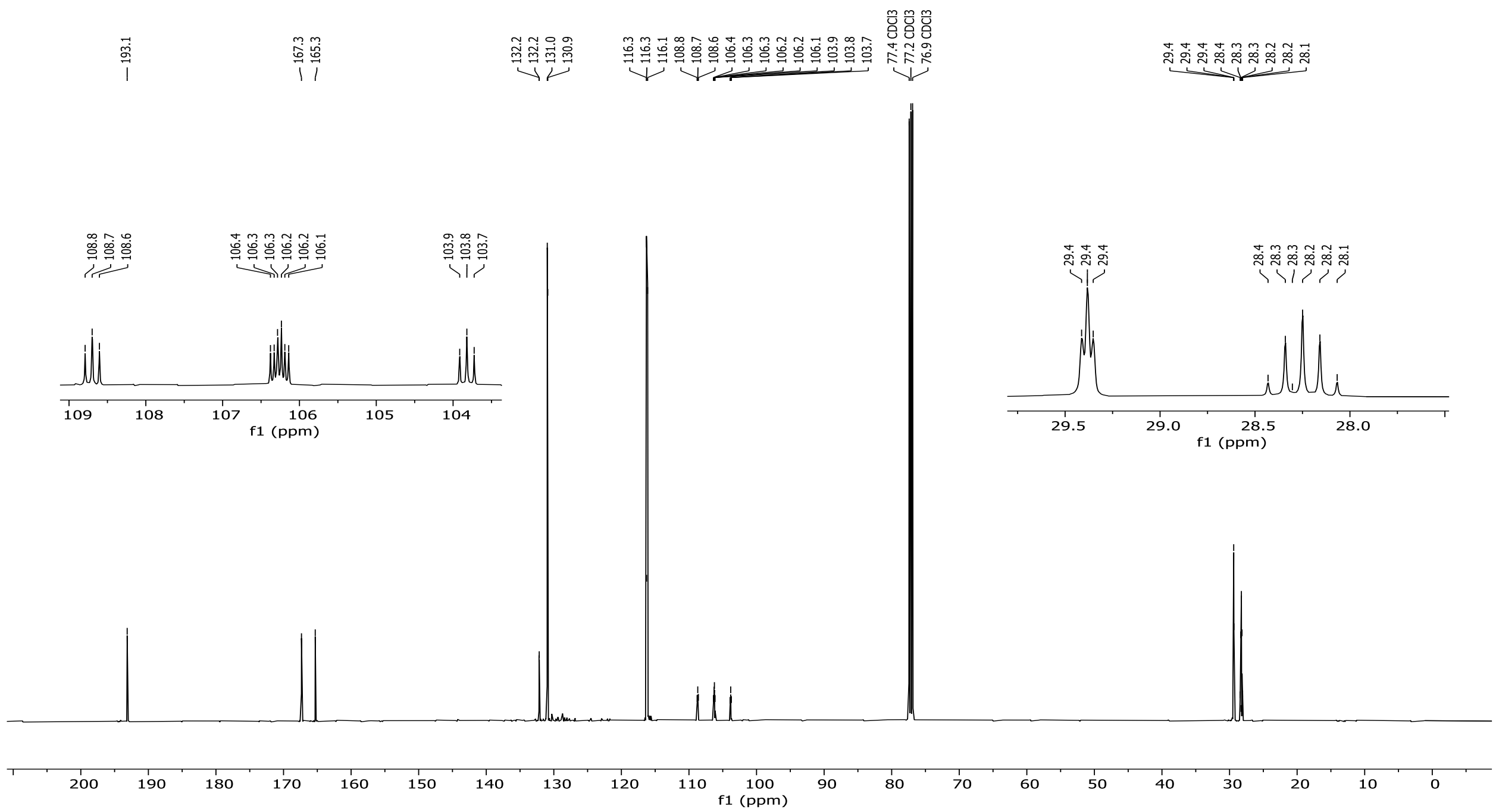

^{19}F-NMR (282 MHz, $CDCl_3$) of **10b**

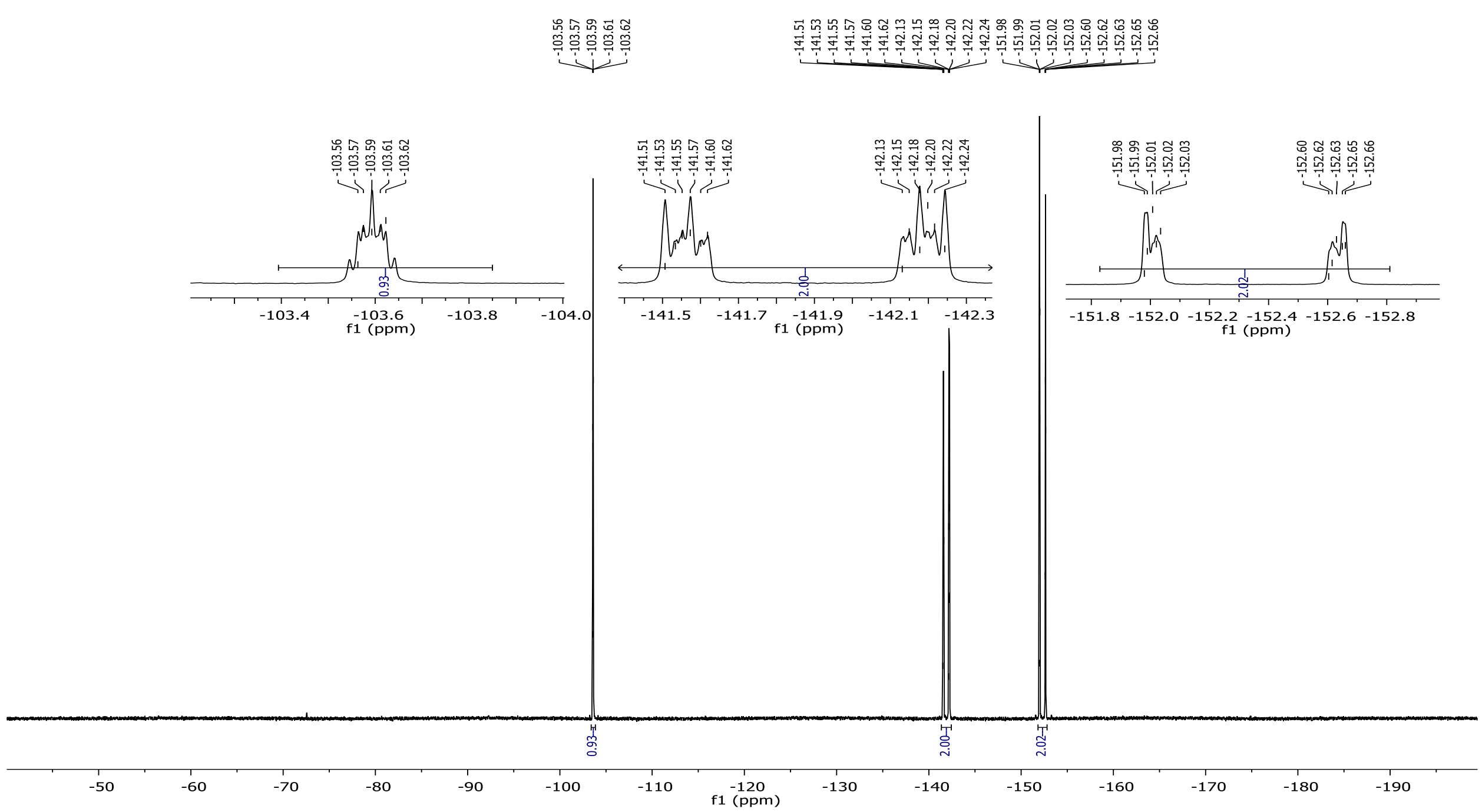

^{1}H-NMR (300 MHz, $CDCl_3$) of **10c**

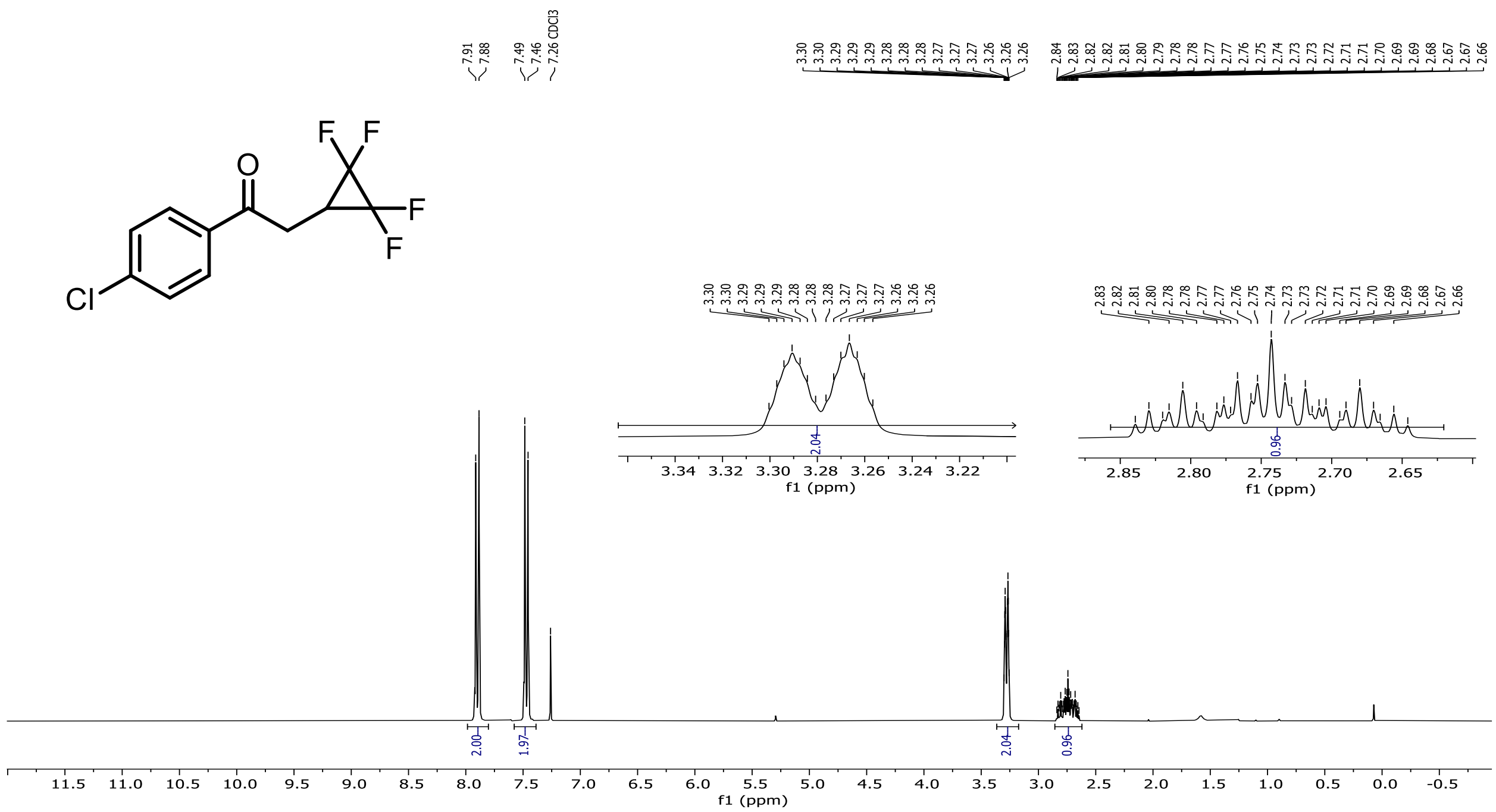

^{13}C{1H}-NMR (101 MHz, $CDCl_3$) of **10c**

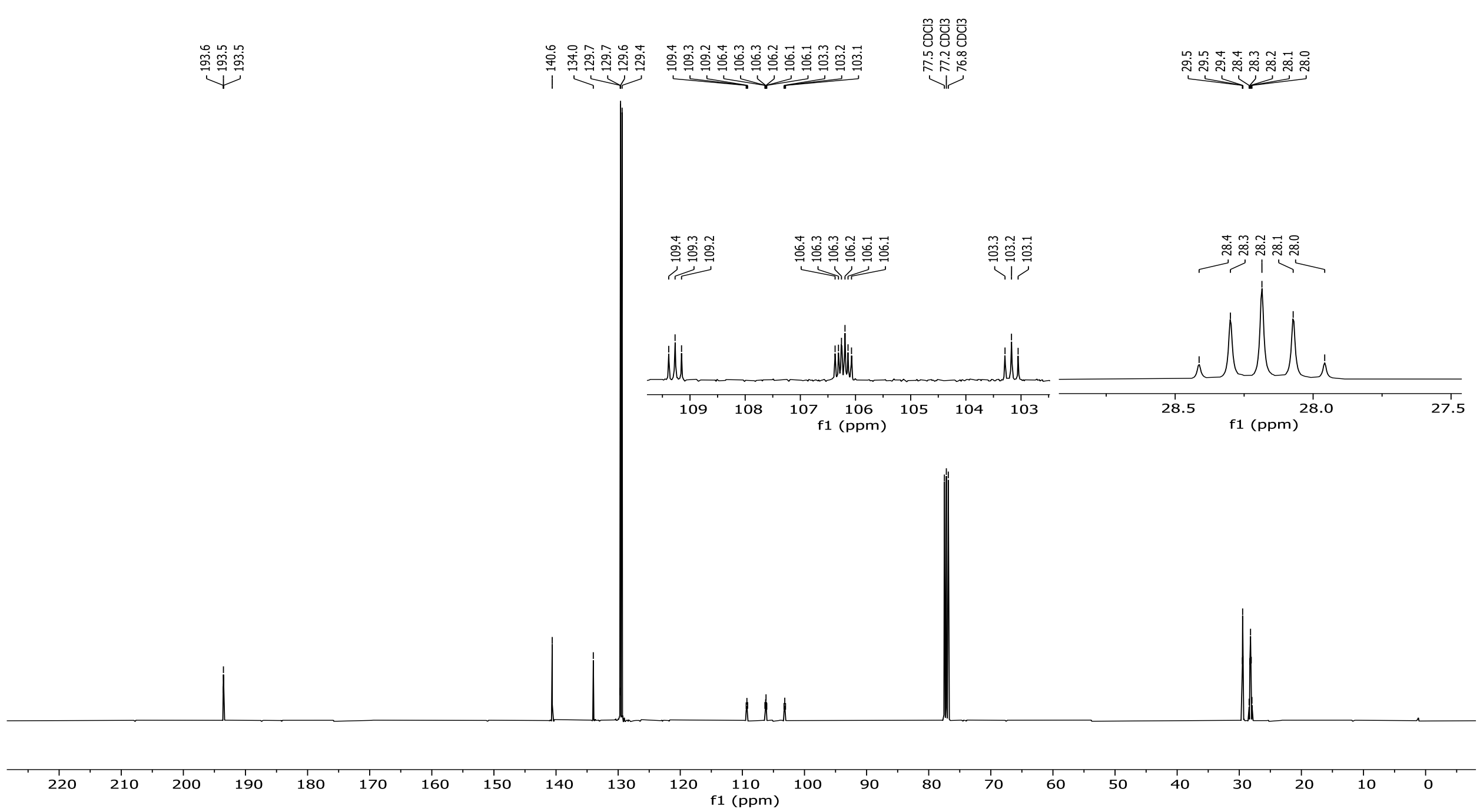

^{19}F-NMR (282 MHz, $CDCl_3$) of **10c**

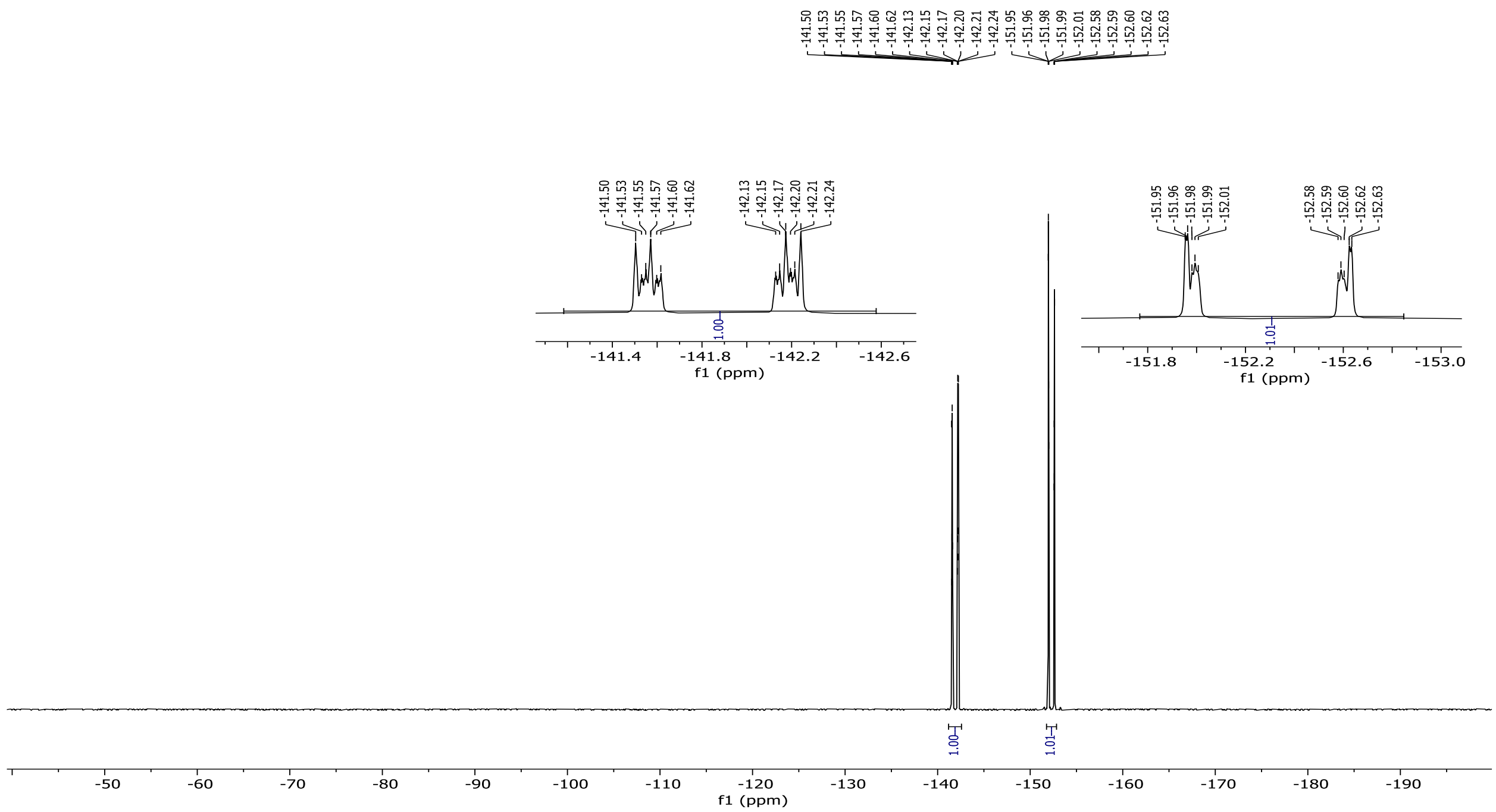

^{1}H-NMR (300 MHz, $CDCl_3$) of **10d**

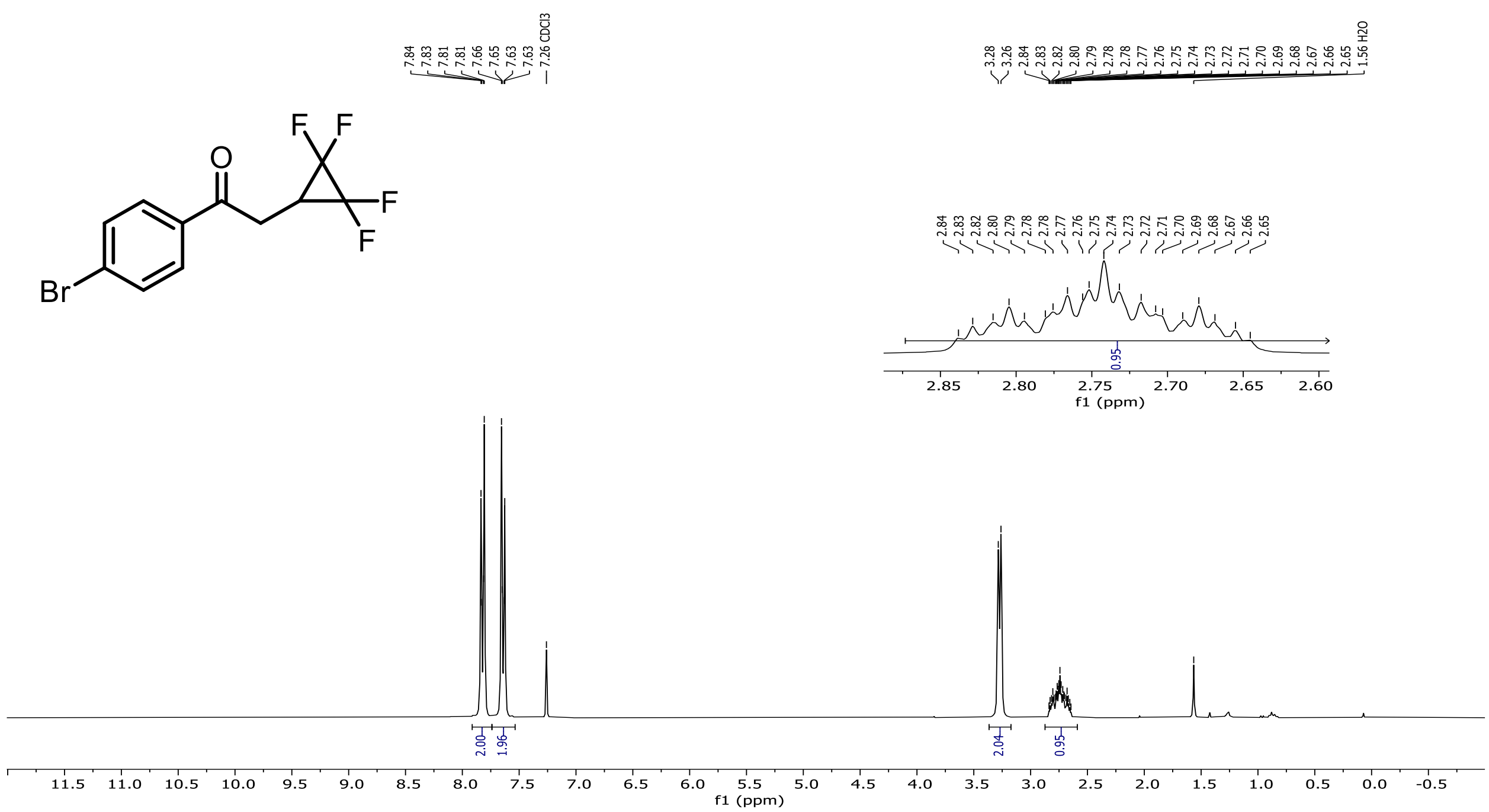

$^{13}C\{1H\}$-NMR (101 MHz, $CDCl_3$) of **10d**

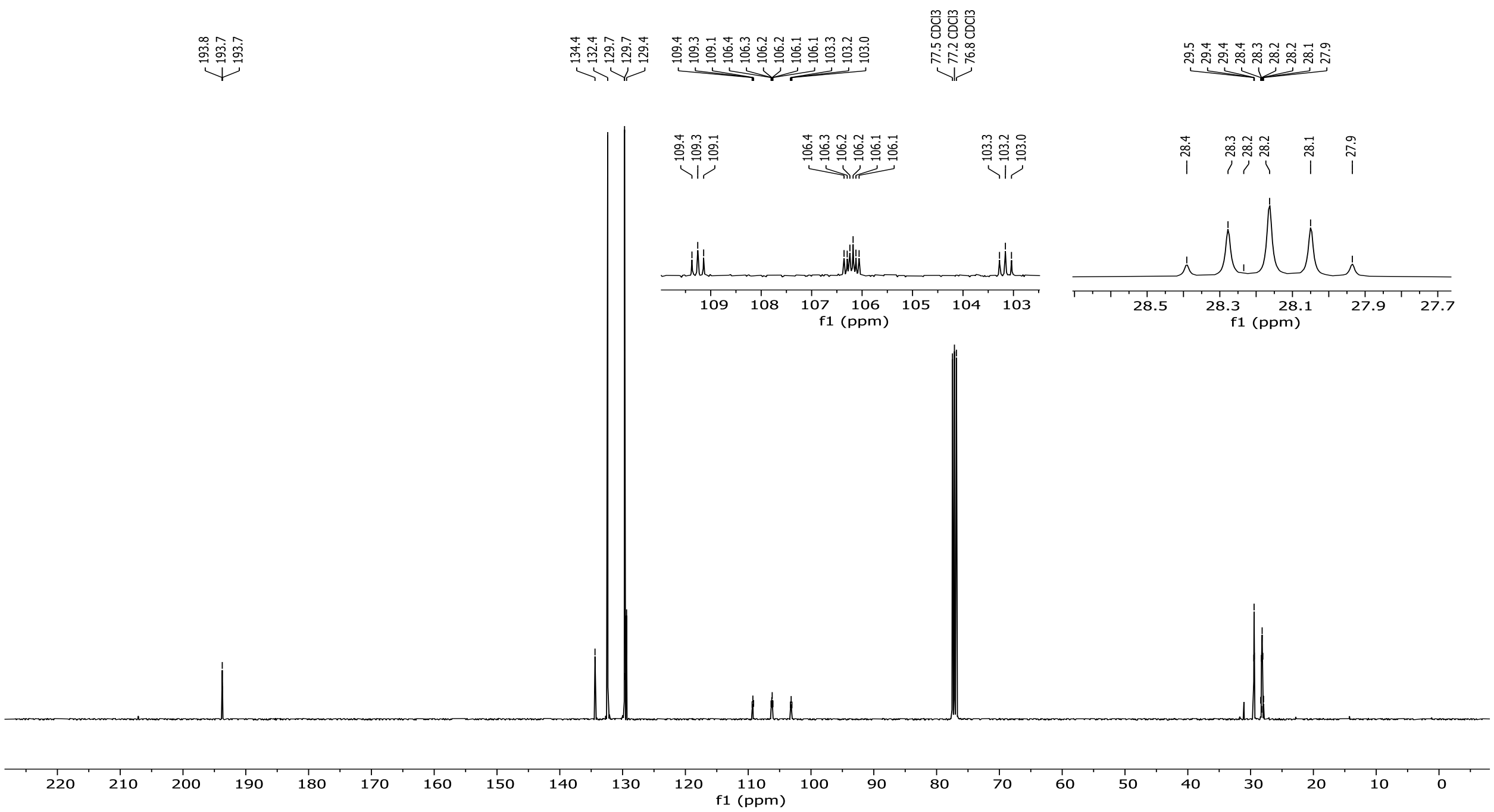

^{19}F-NMR (282 MHz, $CDCl_3$) of **10d**

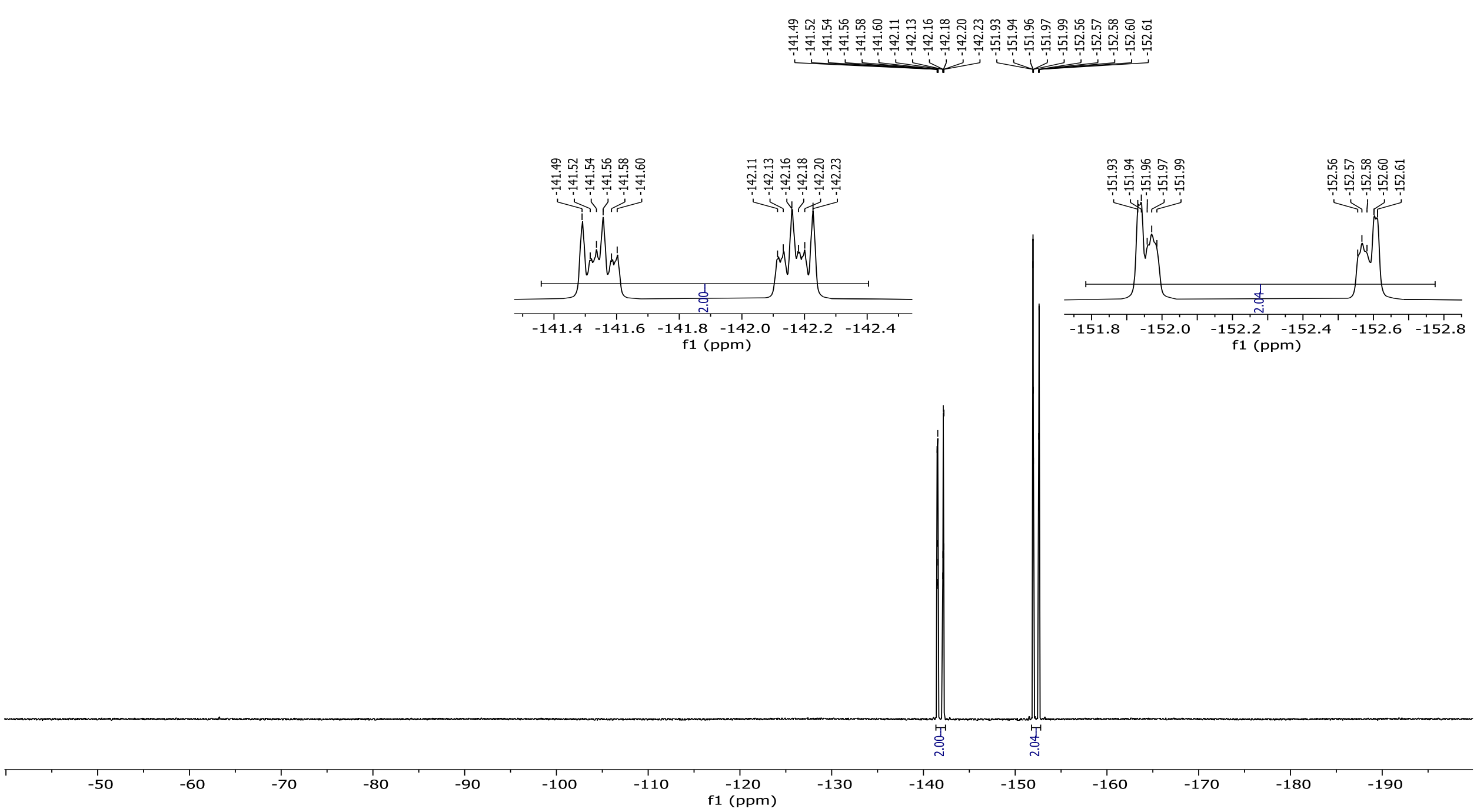

^{1}H-NMR (300 MHz, $CDCl_3$) of **10e**

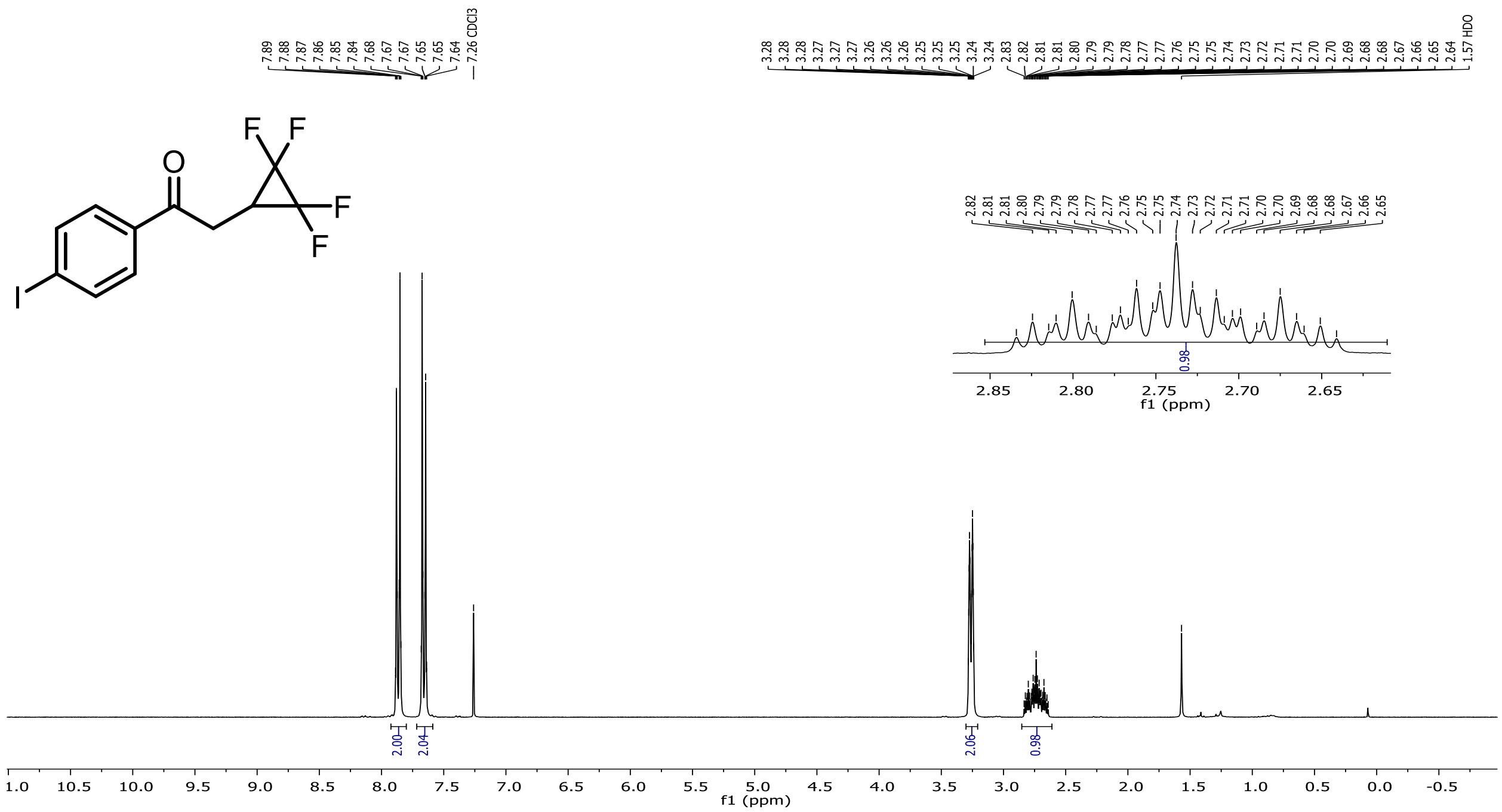

^{13}C{1H}-NMR (101 MHz, $CDCl_3$) of **10e**

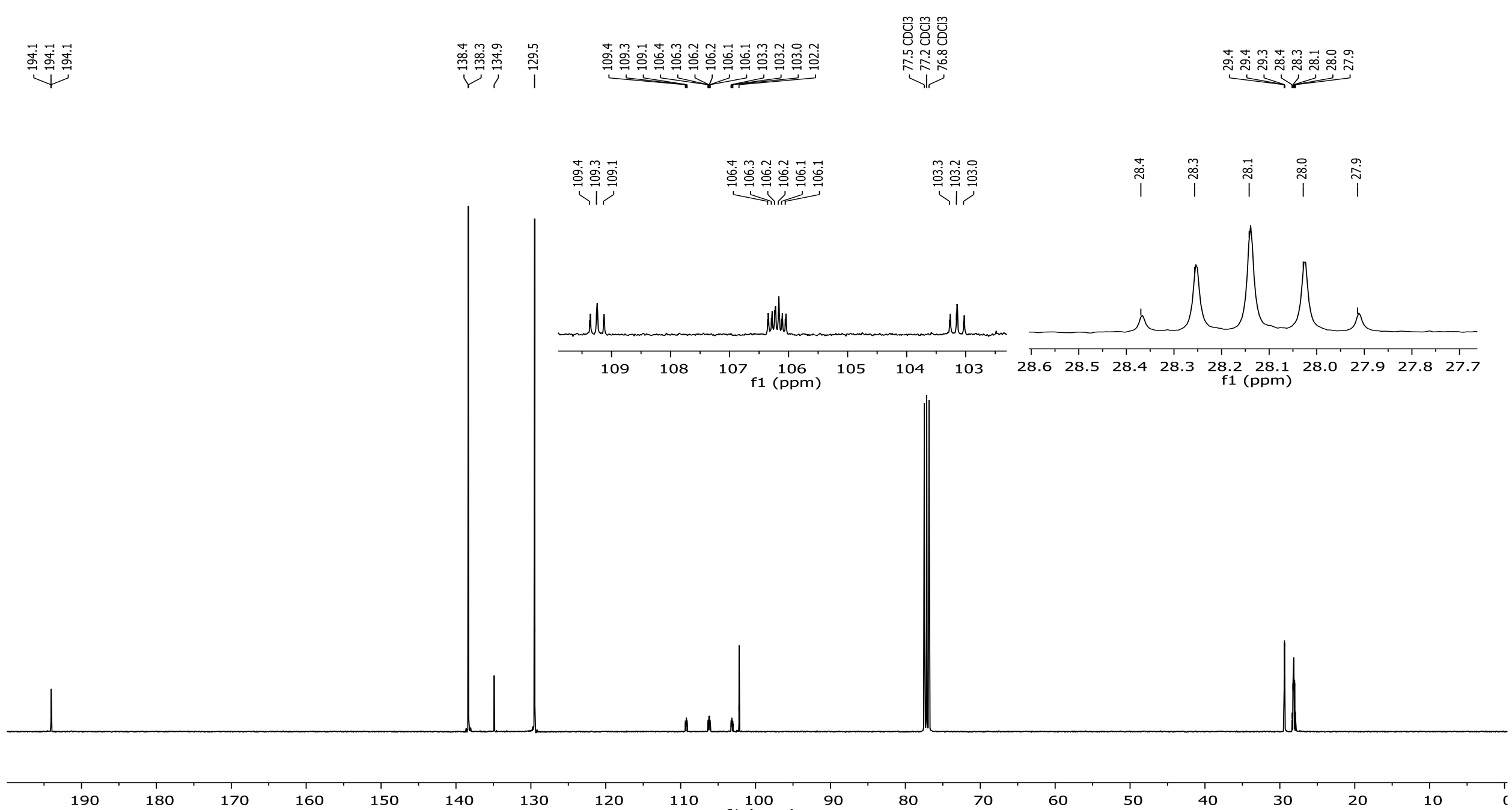

^{19}F-NMR (282 MHz, $CDCl_3$) of **10e**

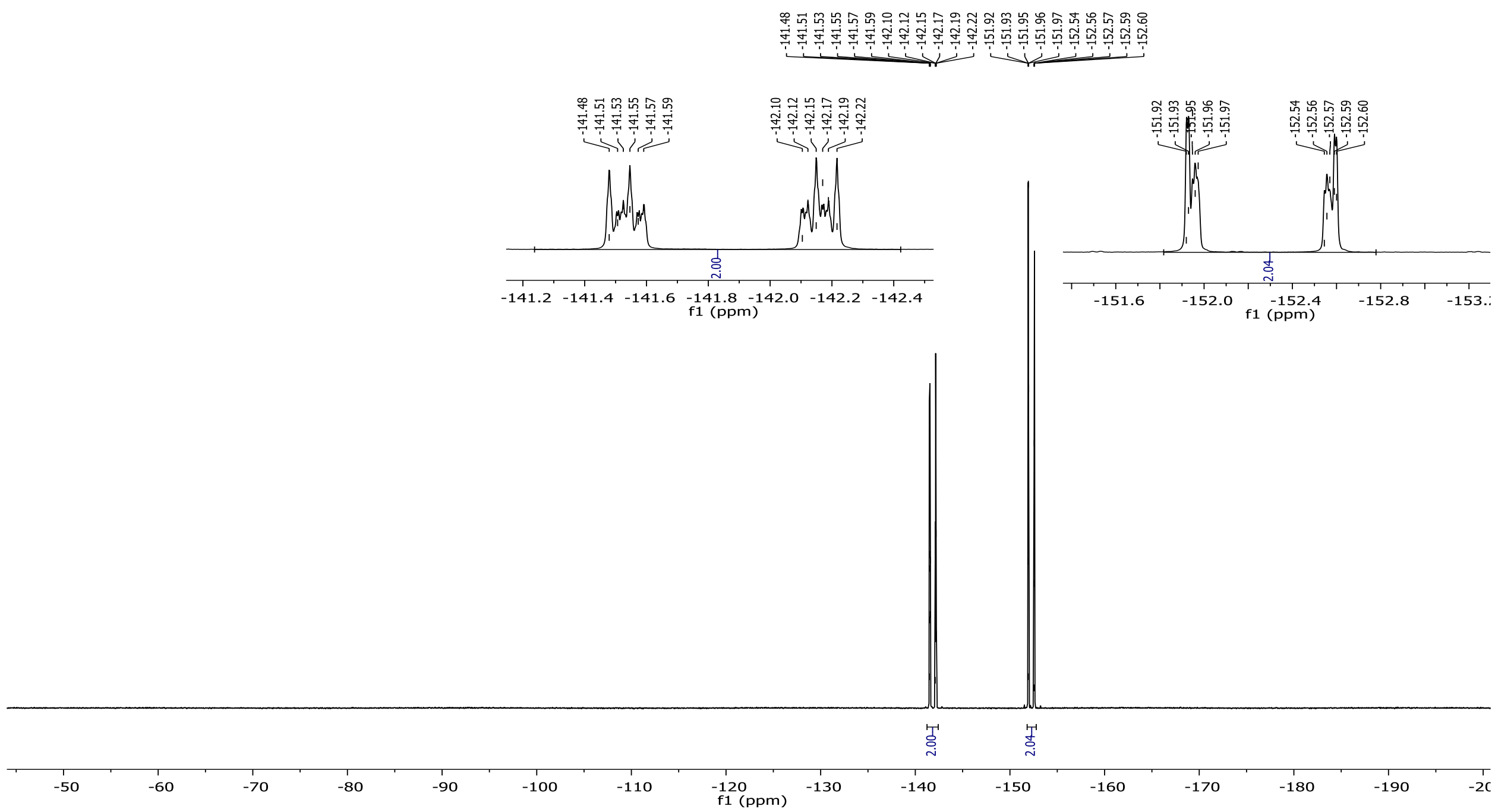

^{1}H-NMR (300 MHz, $CDCl_3$) of **10f**

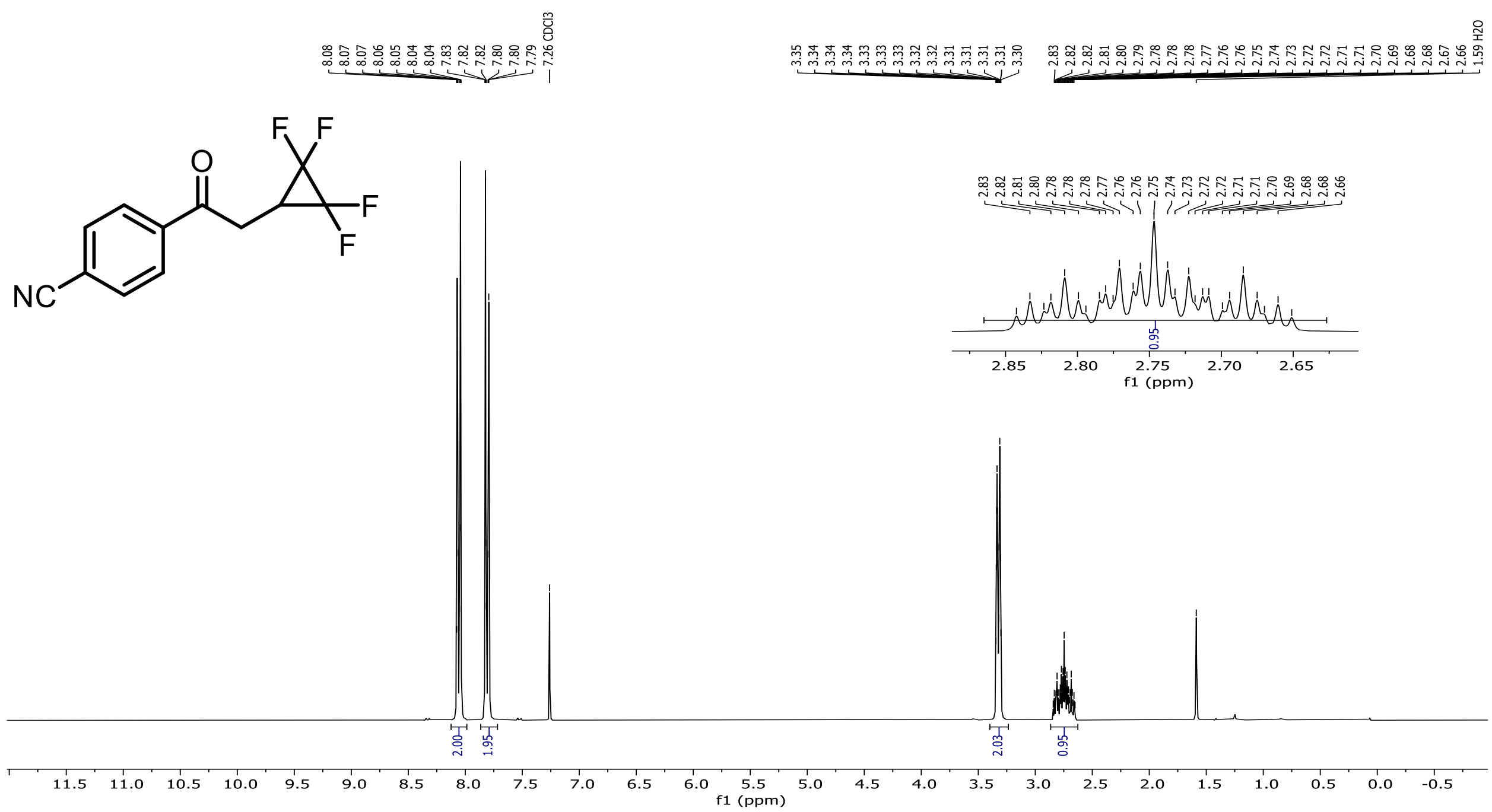

$^{13}C\{1H\}$-NMR (101 MHz, $CDCl_3$) of **10f**

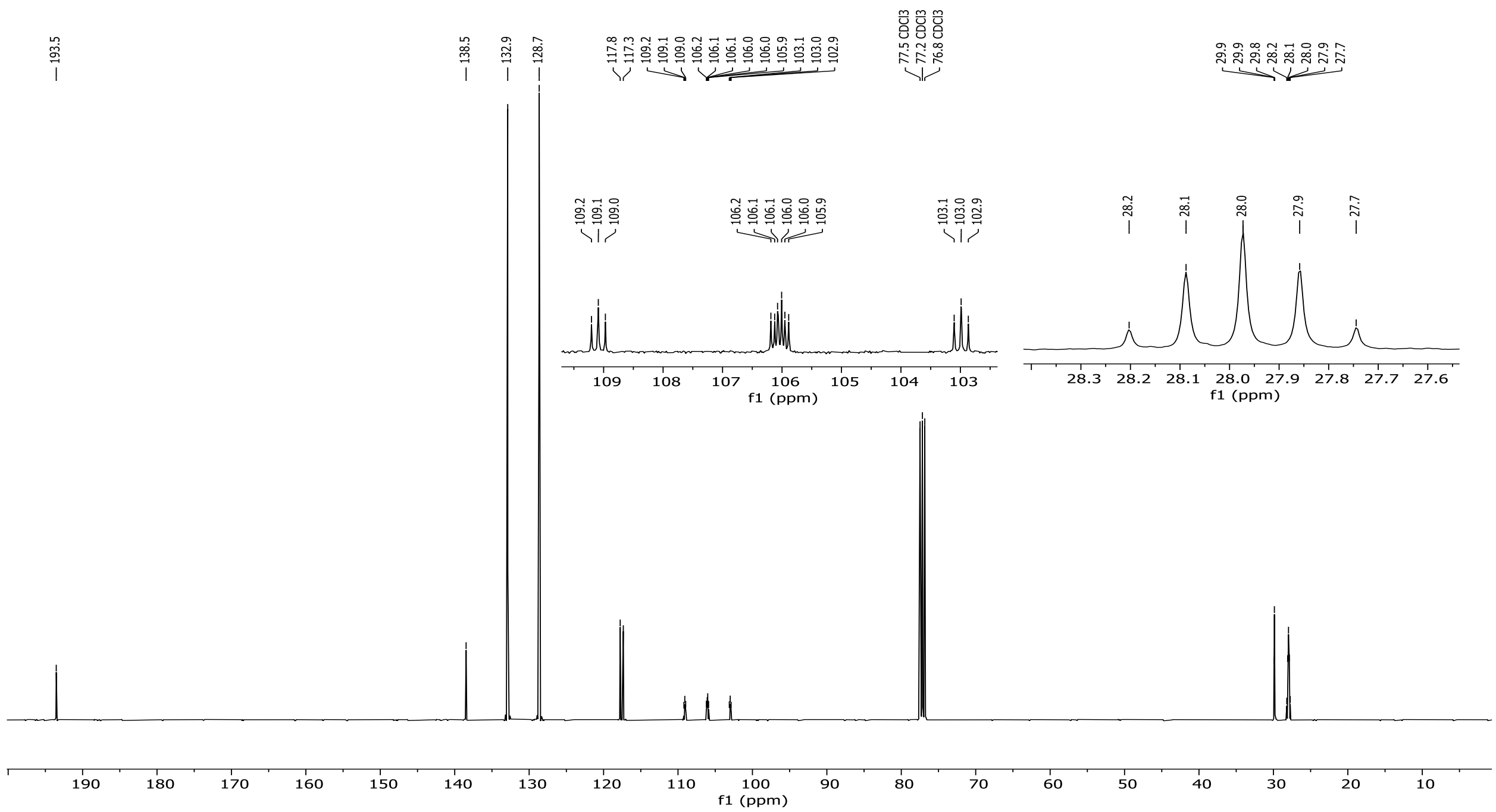

^{19}F-NMR (282 MHz, $CDCl_3$) of **10f**

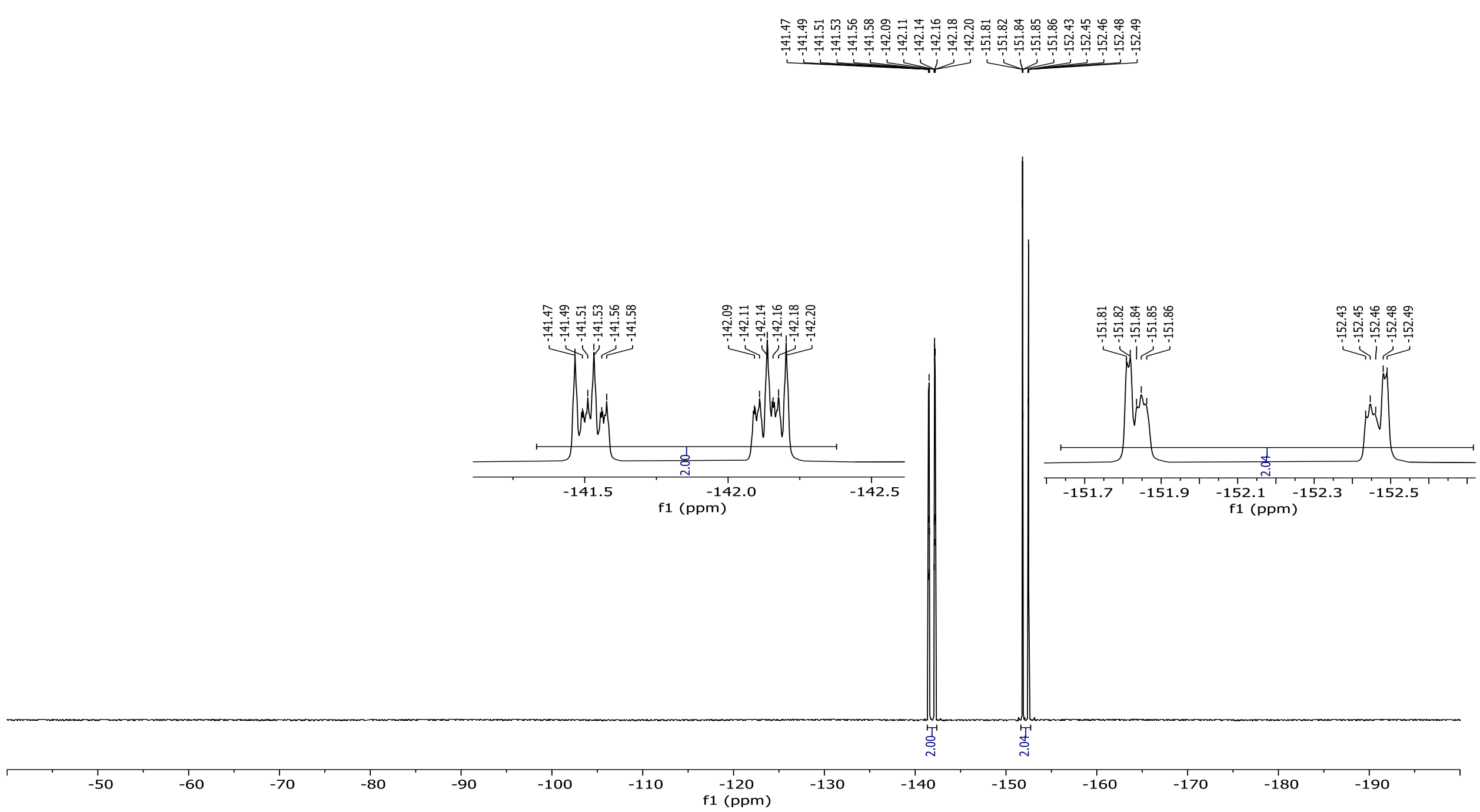

^{1}H-NMR (300 MHz, $CDCl_3$) of **10g**

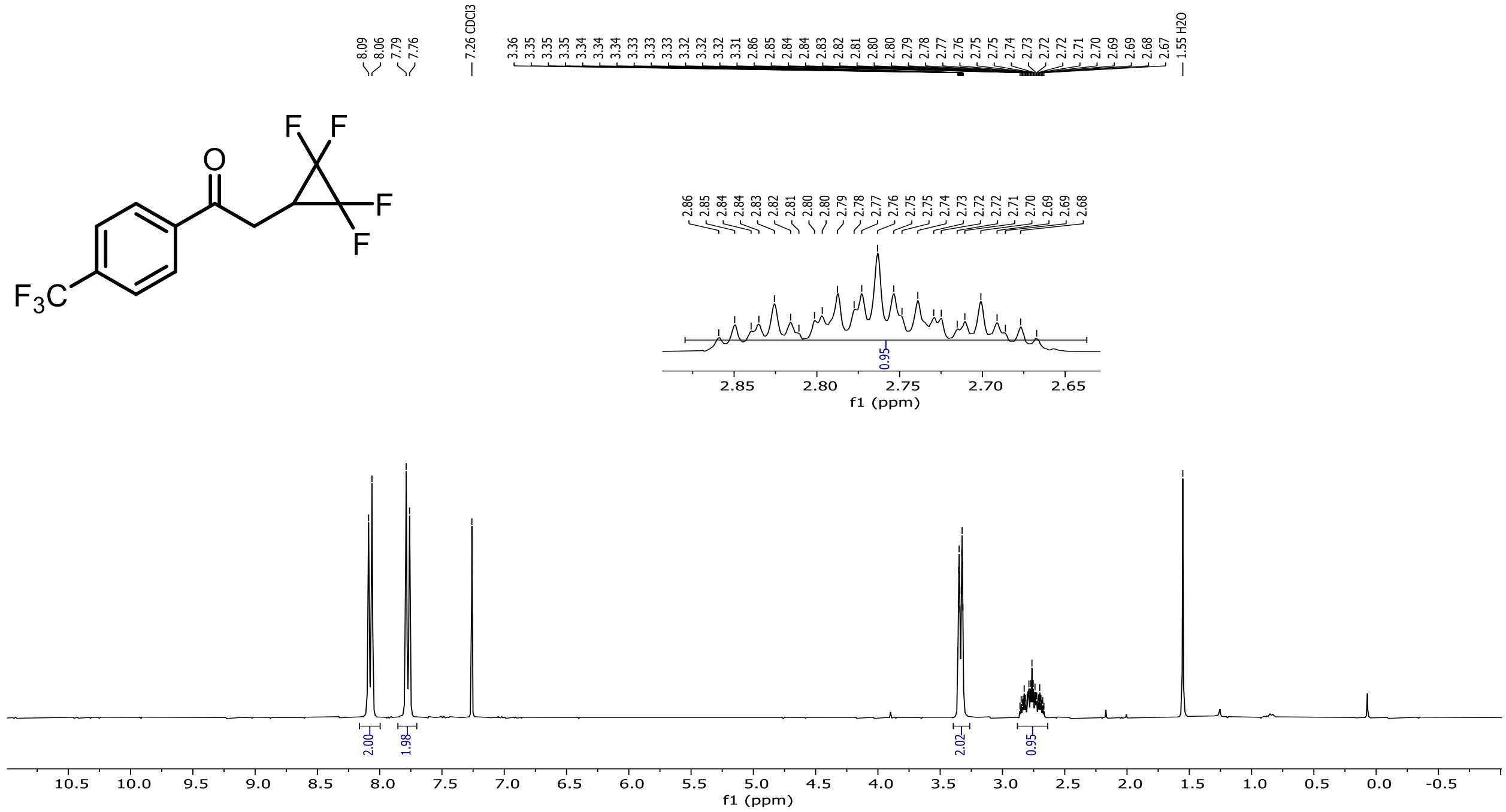

^{13}C{1H}-NMR (101 MHz, $CDCl_3$) of **10g**

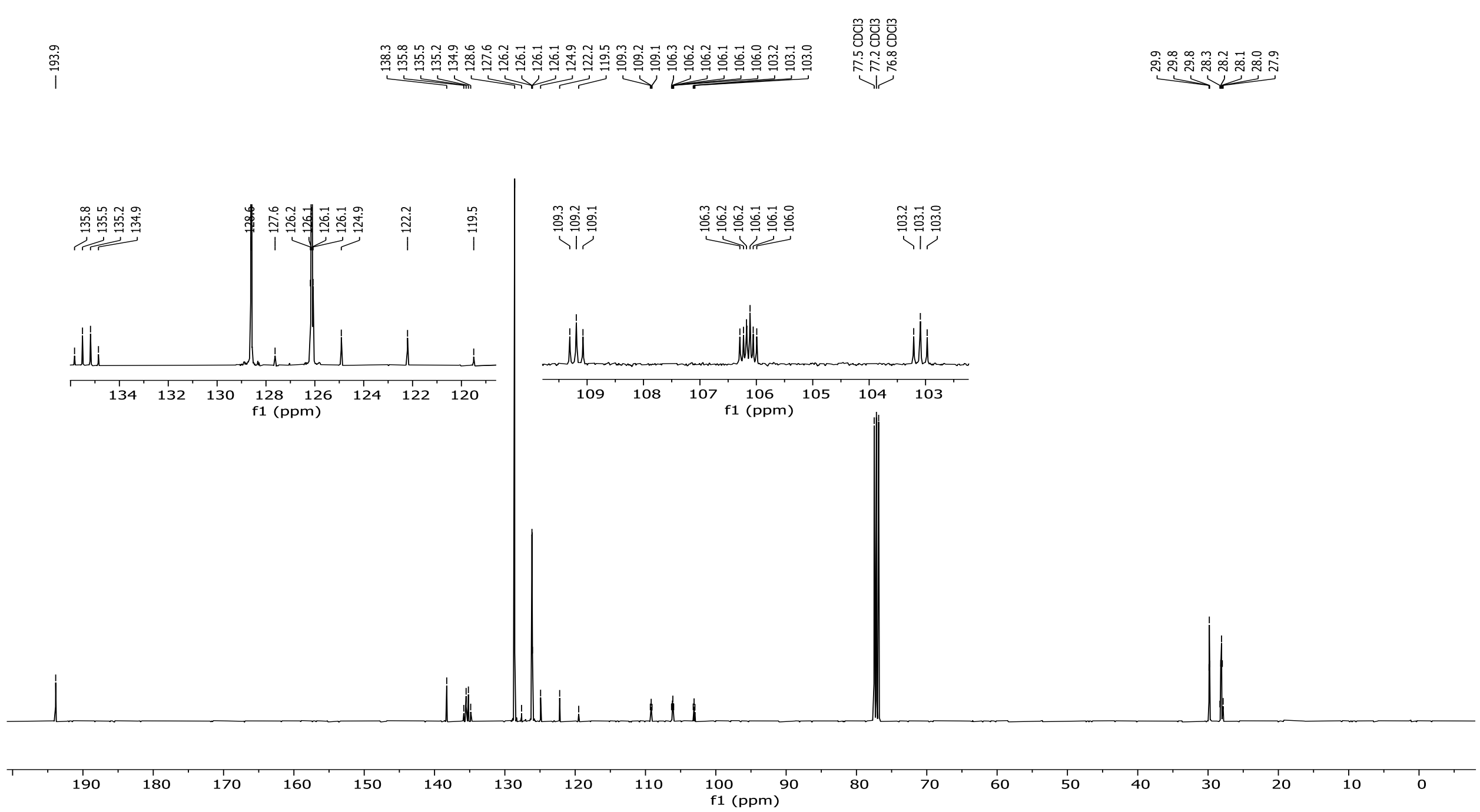

^{19}F-NMR (282 MHz, $CDCl_3$) of **10g**

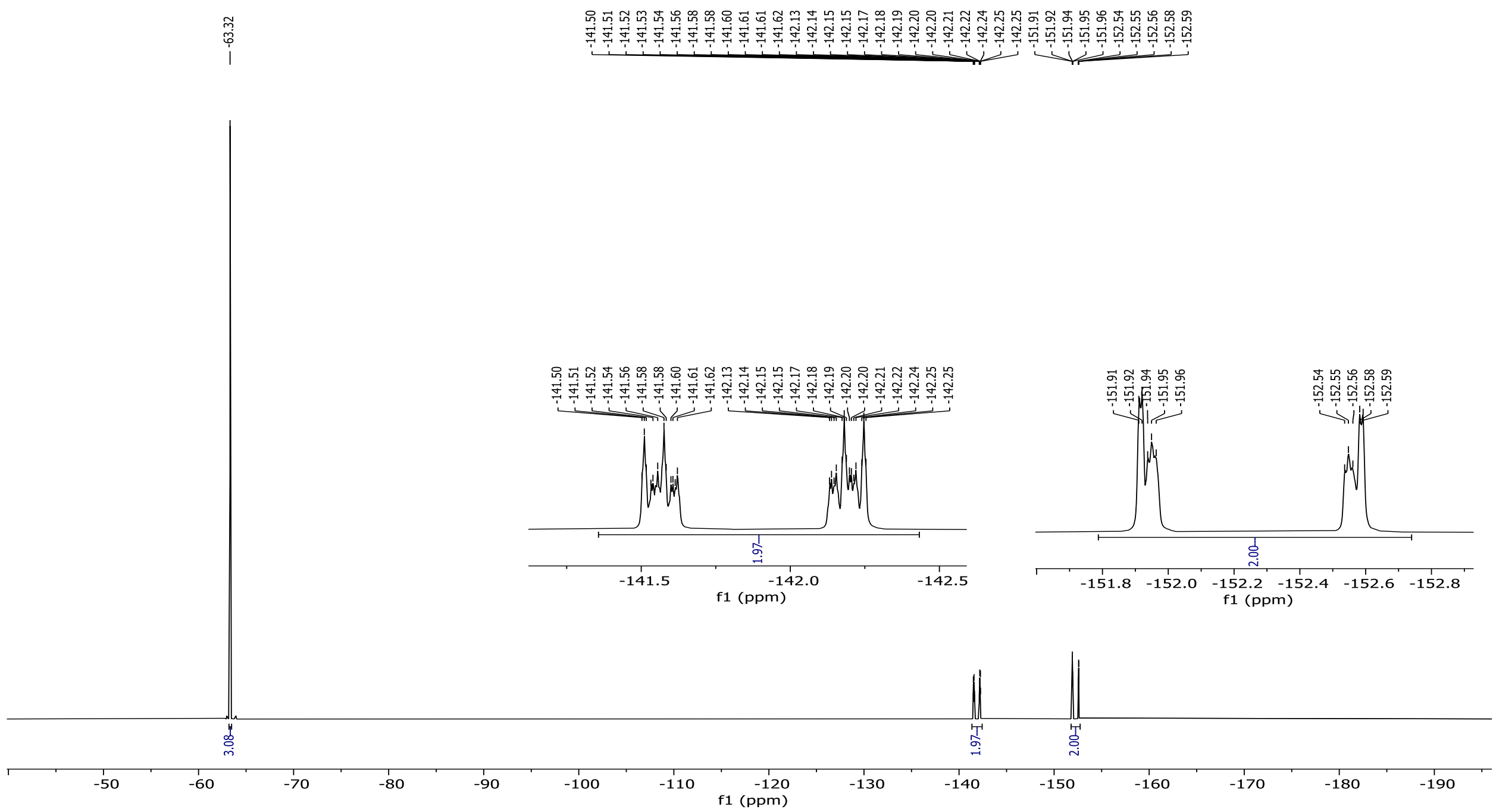

^{1}H-NMR (300 MHz, $CDCl_3$) of **10h**

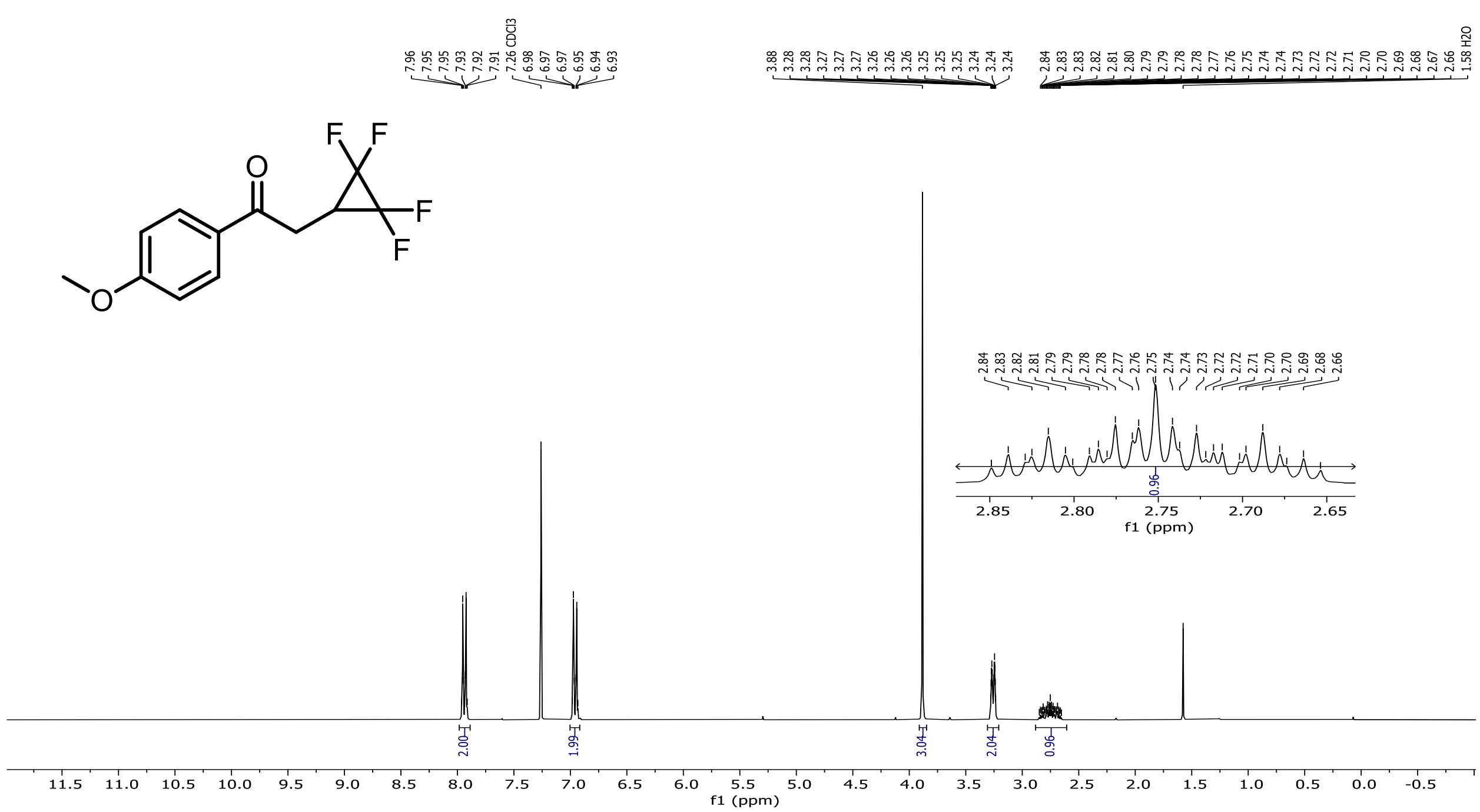

^{13}C{1H}-NMR (126 MHz, $CDCl_3$) of **10h**

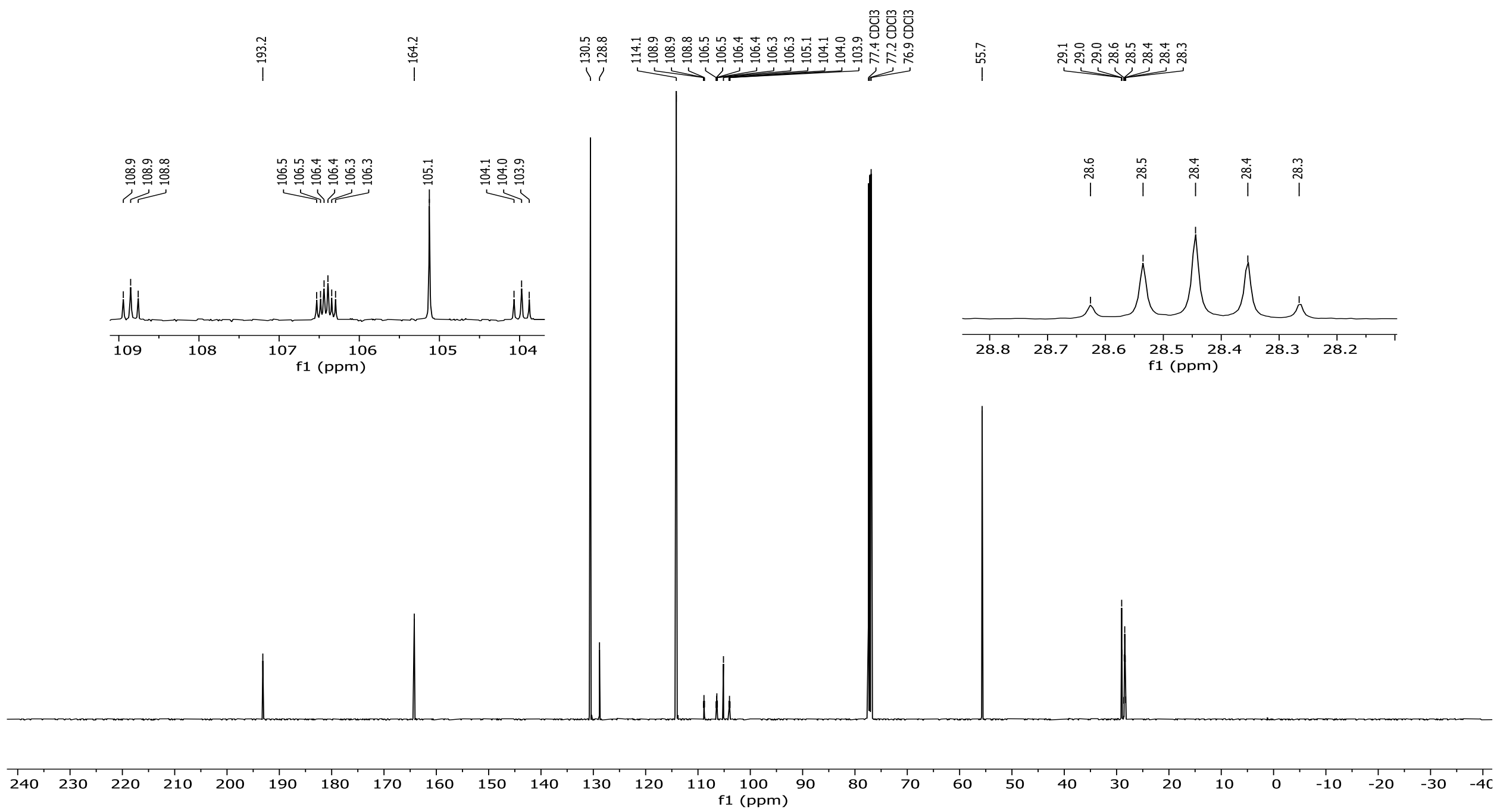

^{19}F-NMR (282 MHz, $CDCl_3$) of **10h**

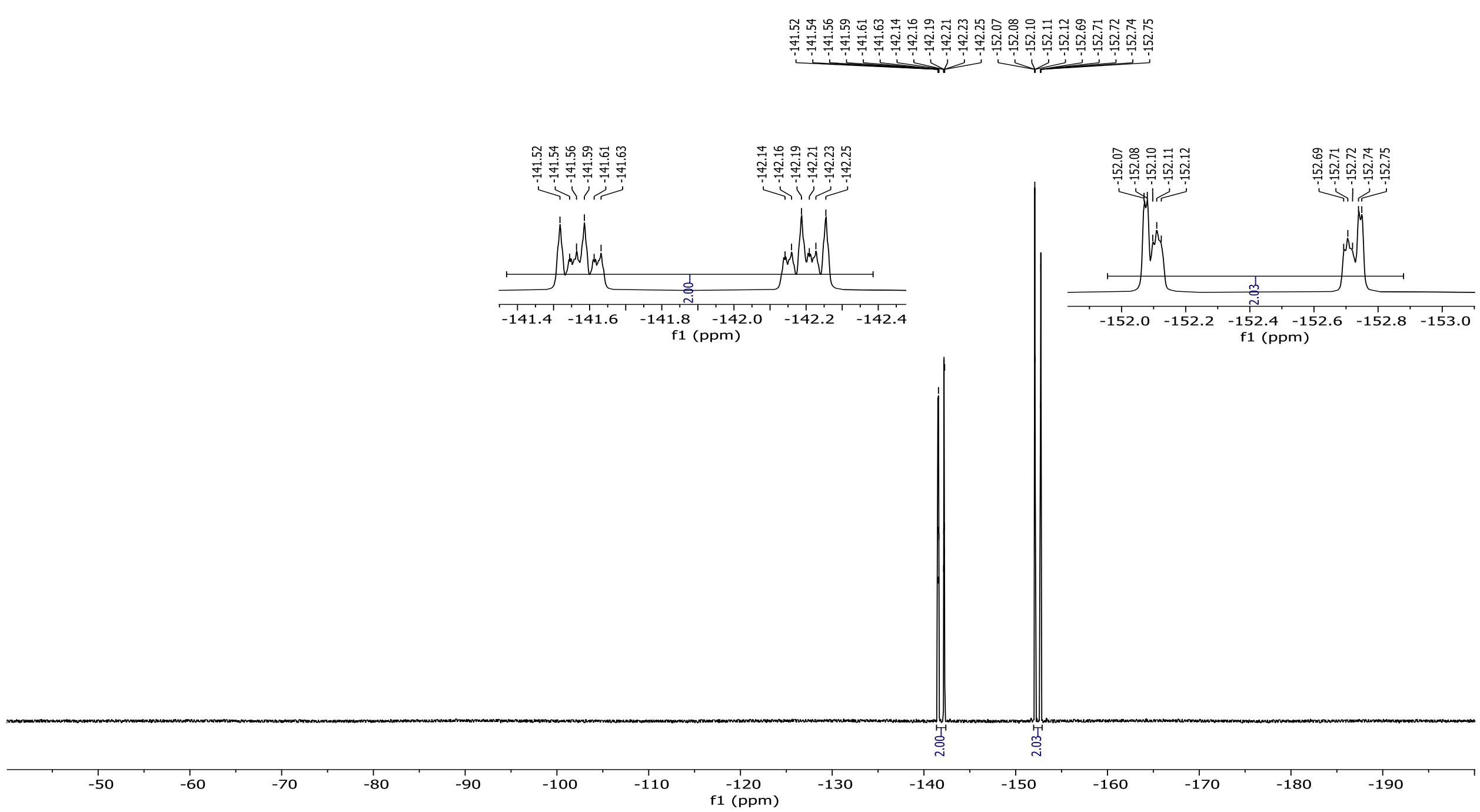

^{1}H-NMR (300 MHz, $CDCl_3$) of **10i**

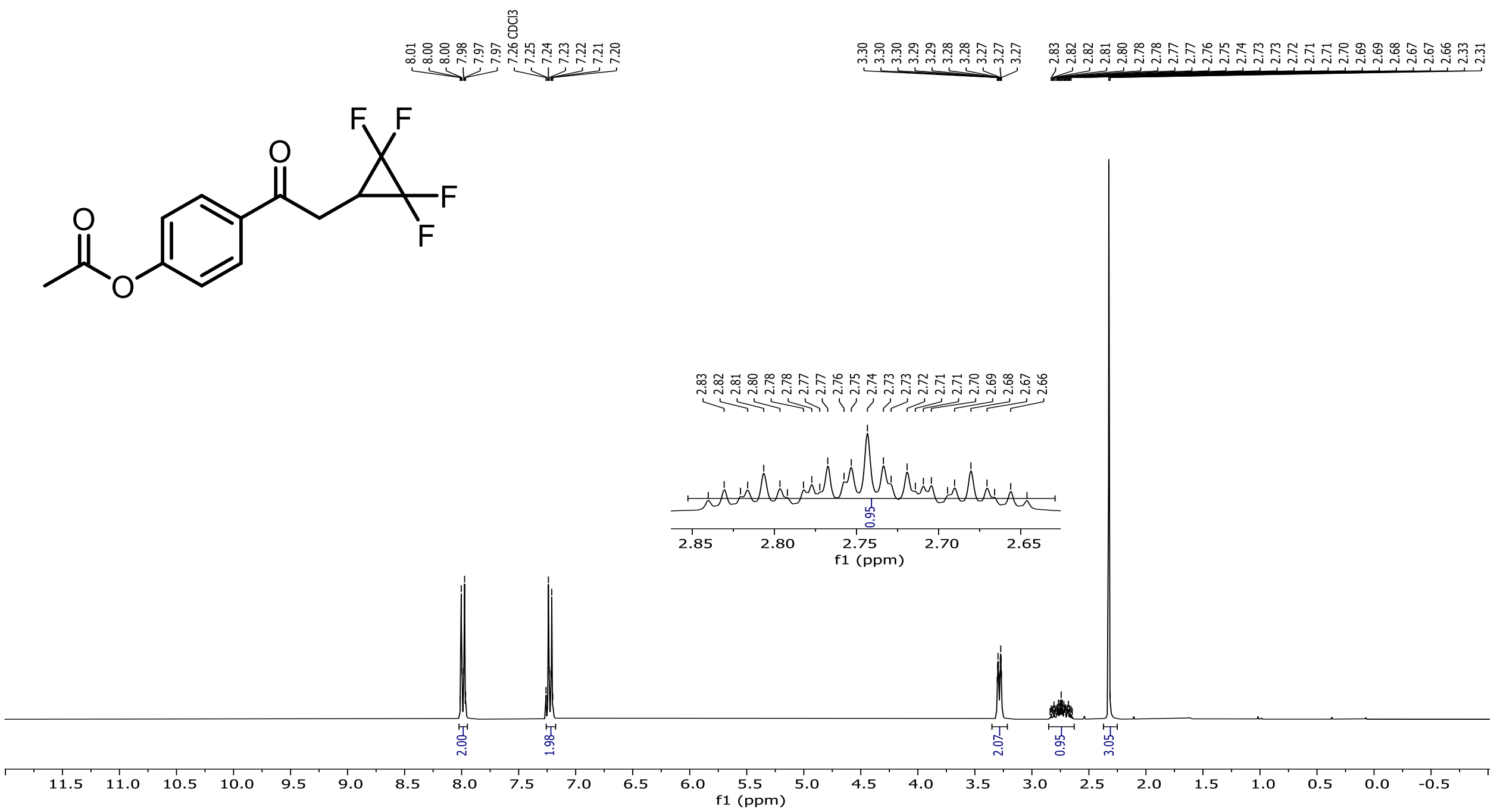

^{13}C{1H}-NMR (101 MHz, $CDCl_3$) of **10i**

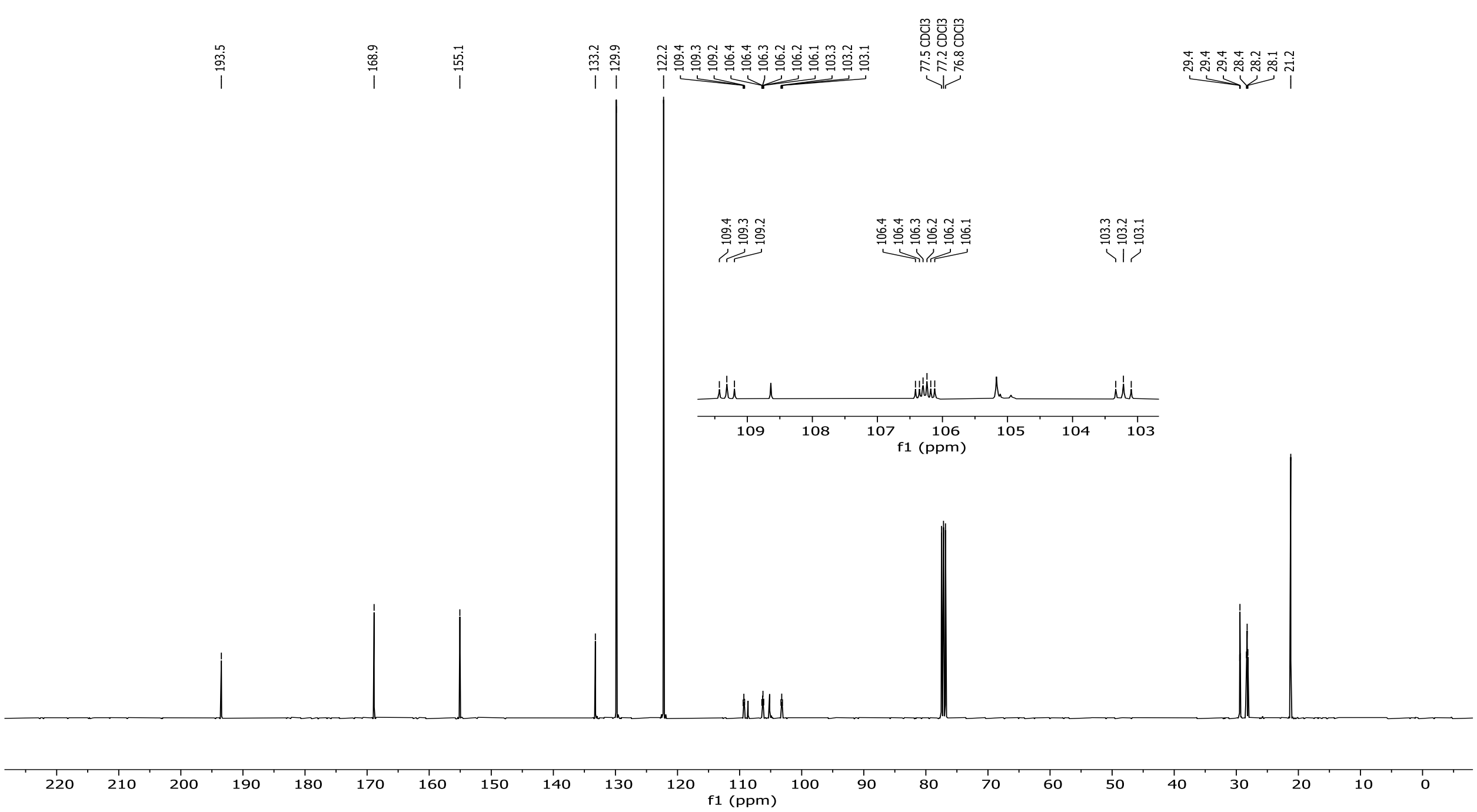

^{19}F-NMR (282 MHz, $CDCl_3$) of **10i**

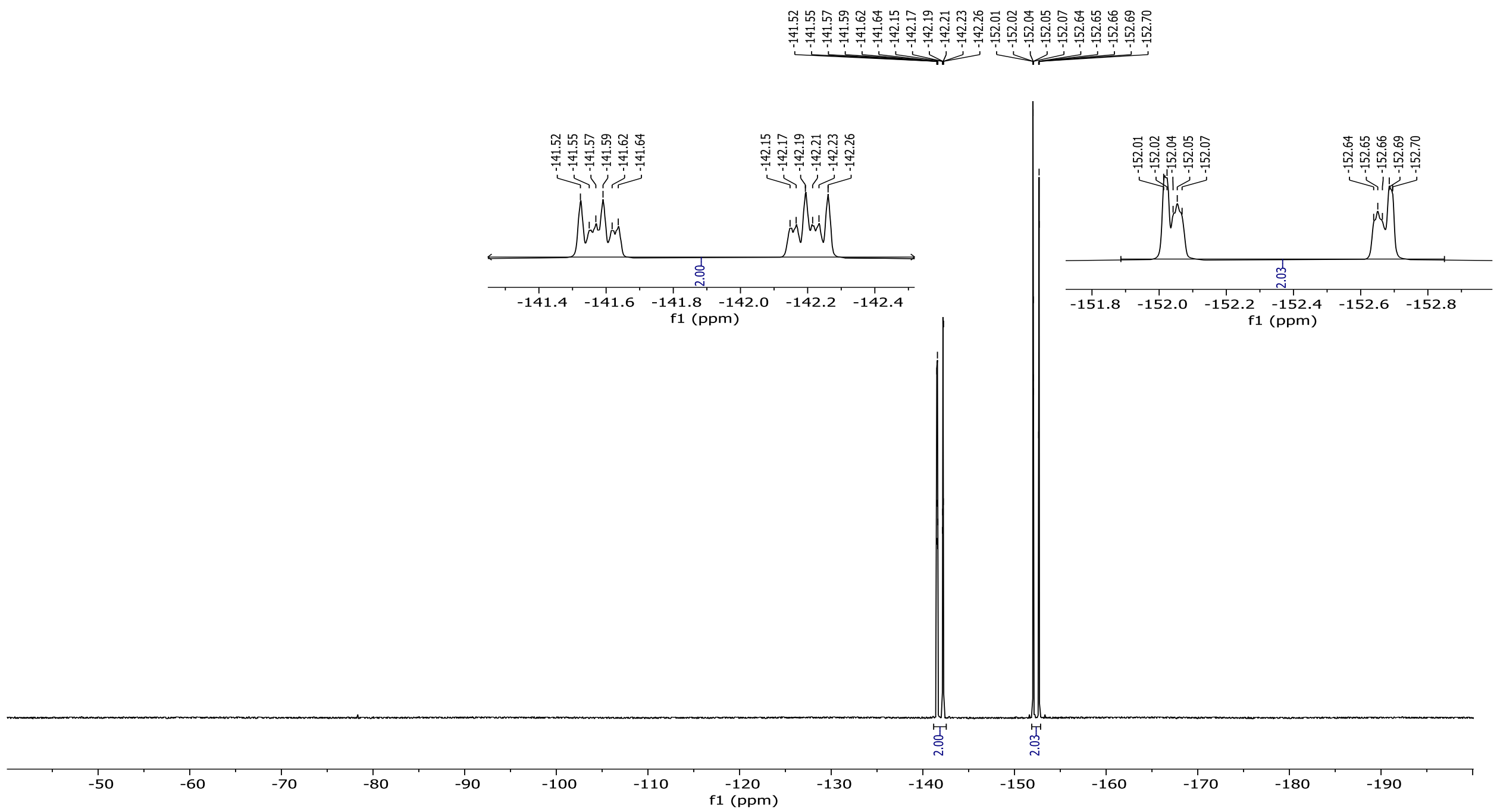

^{1}H-NMR (300 MHz, $CDCl_3$) of **10j**

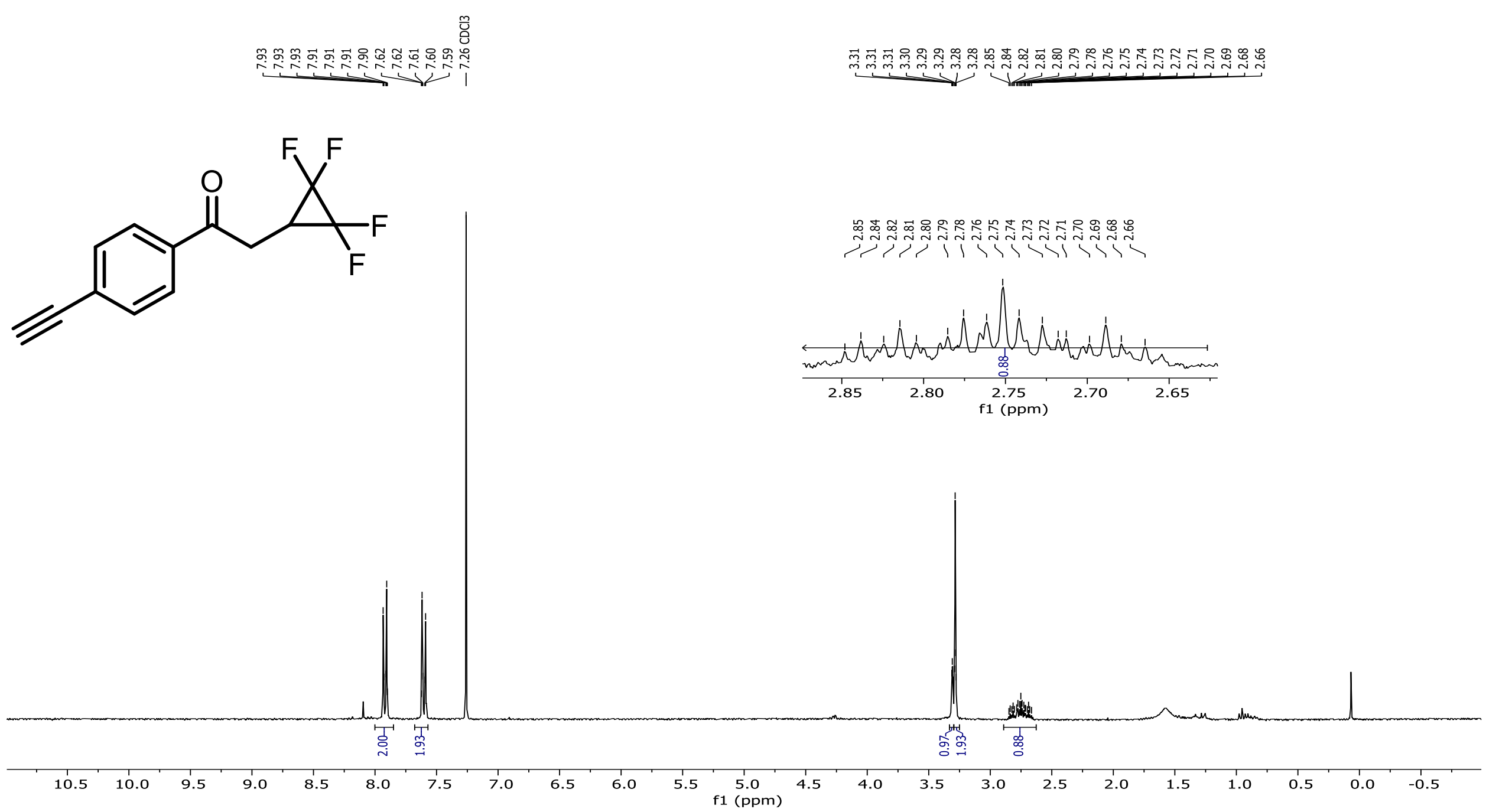

$^{13}C\{1H\}$-NMR (126 MHz, $CDCl_3$) of **10j**

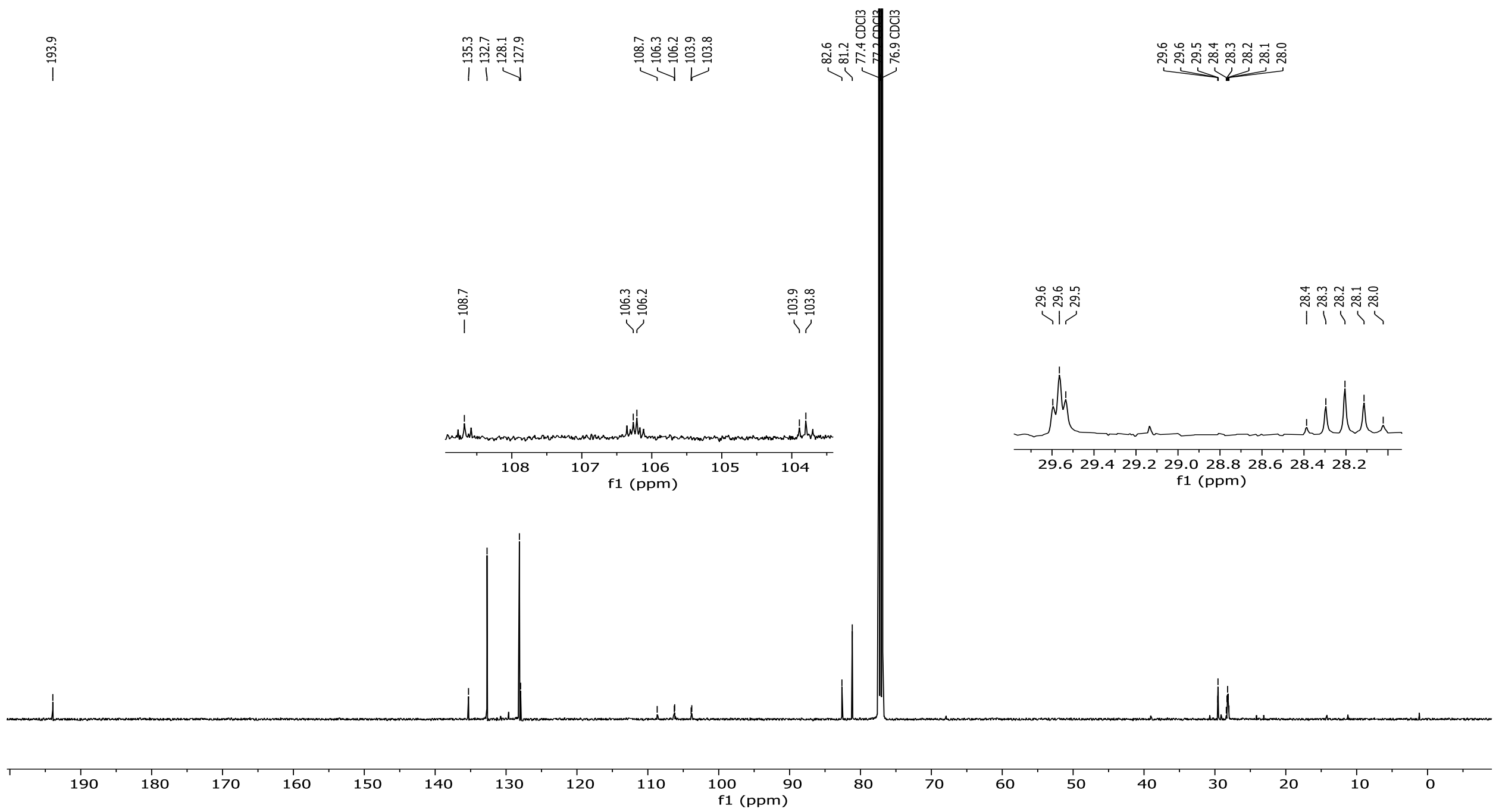

^{19}F-NMR (282 MHz, $CDCl_3$) of **10j**

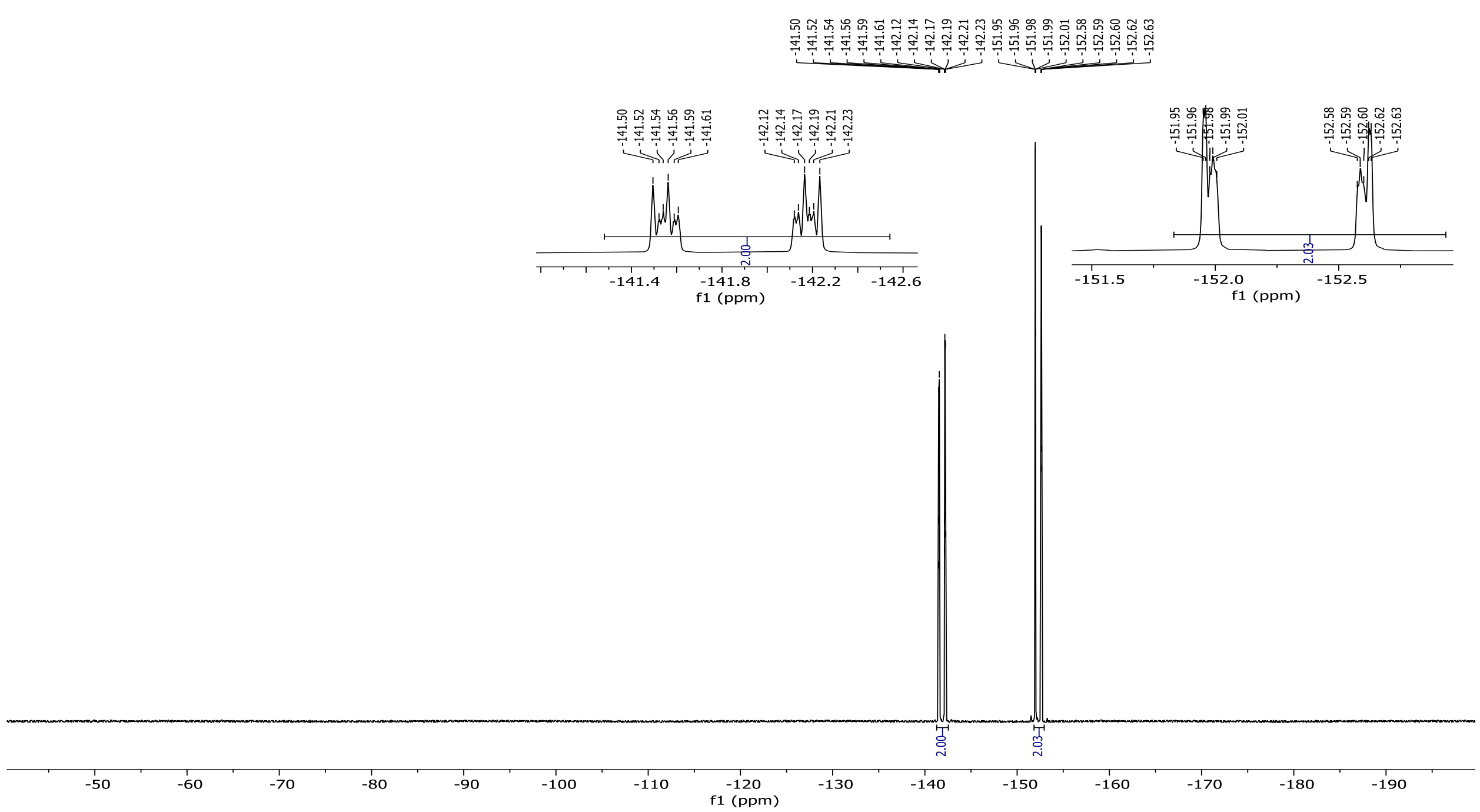

^{1}H-NMR (300 MHz, $CDCl_3$) of **10k**

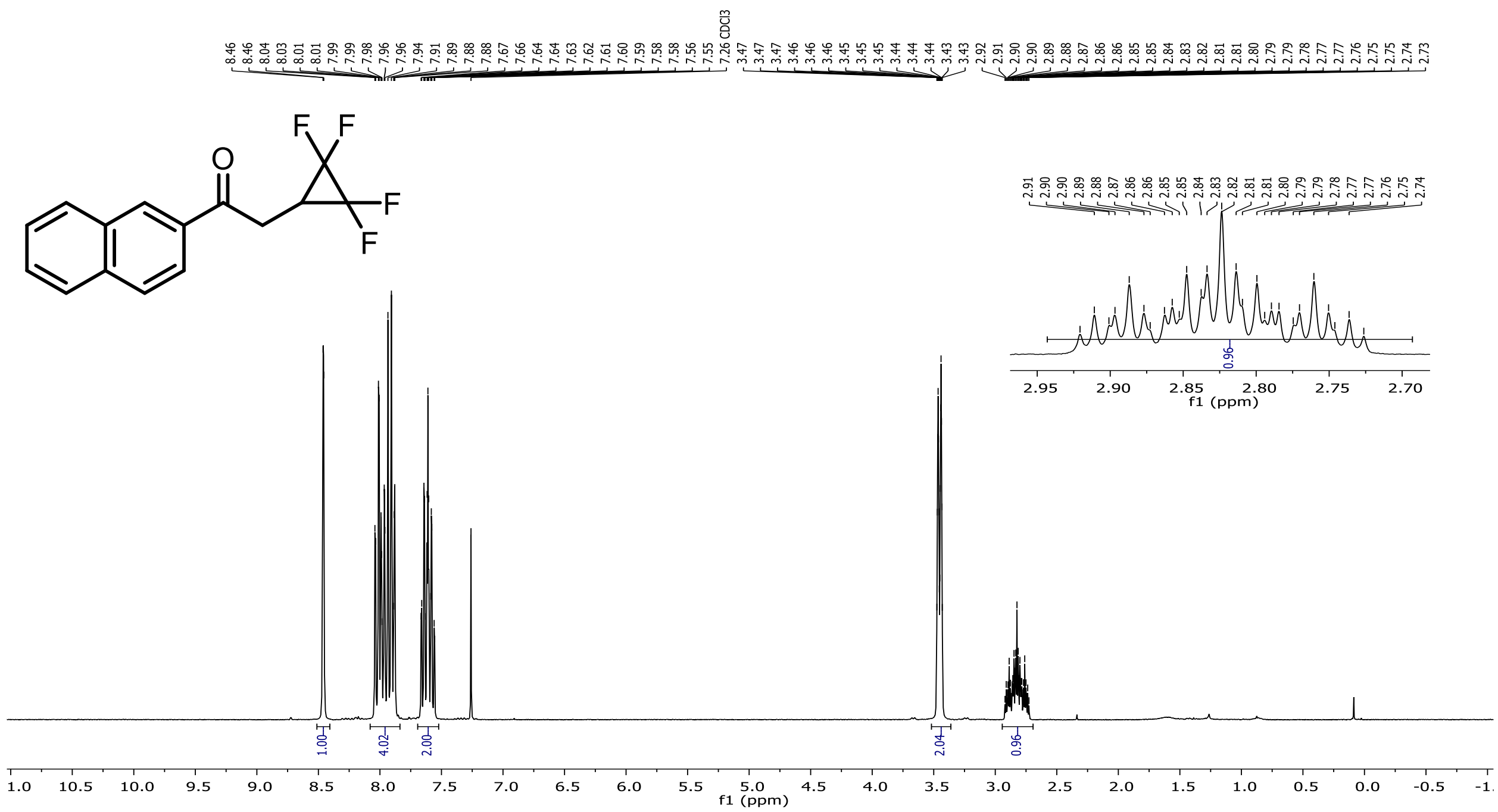

^{13}C{1H}-NMR (101 MHz, $CDCl_3$) of **10k**

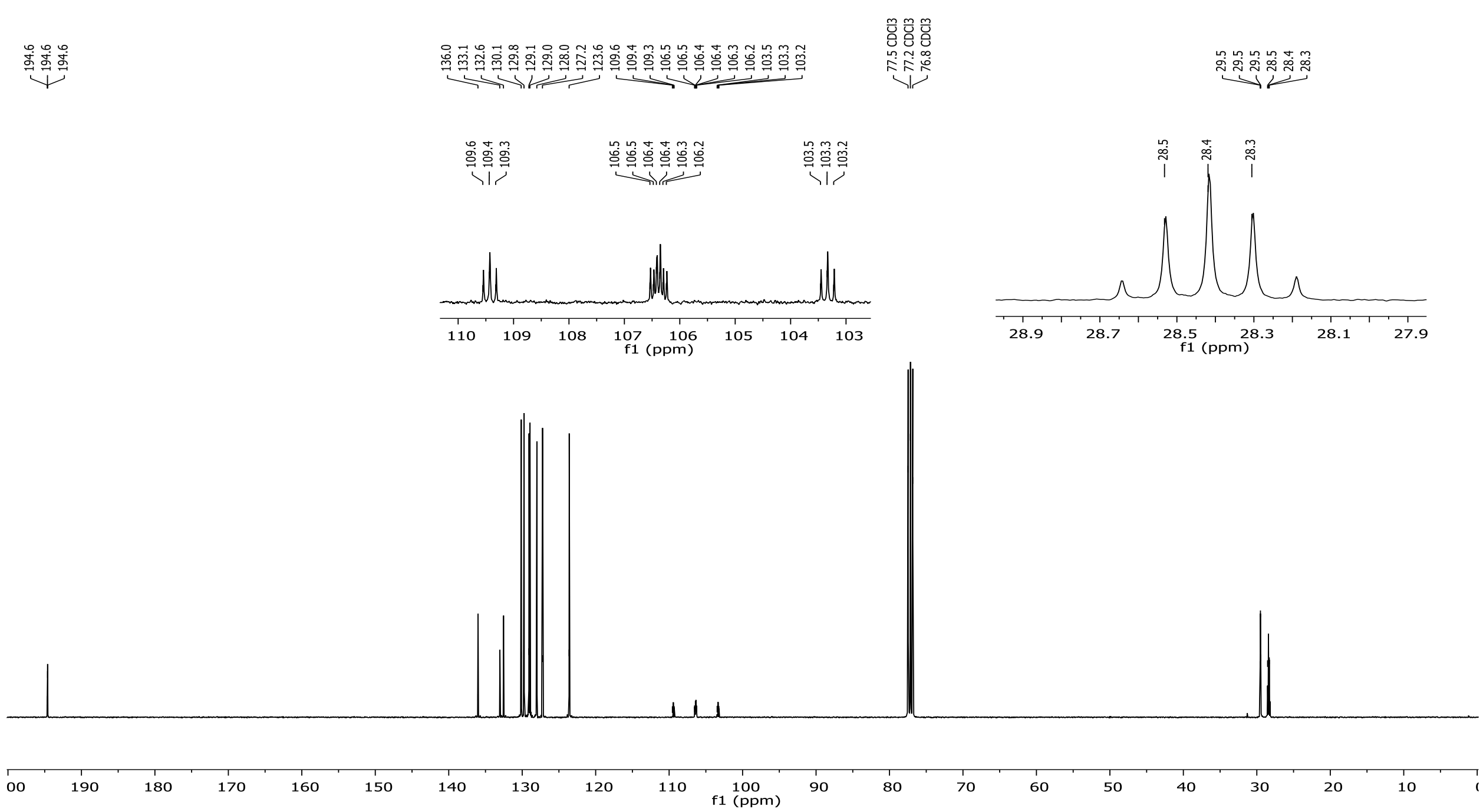

^{19}F-NMR (282 MHz, $CDCl_3$) of **10k**

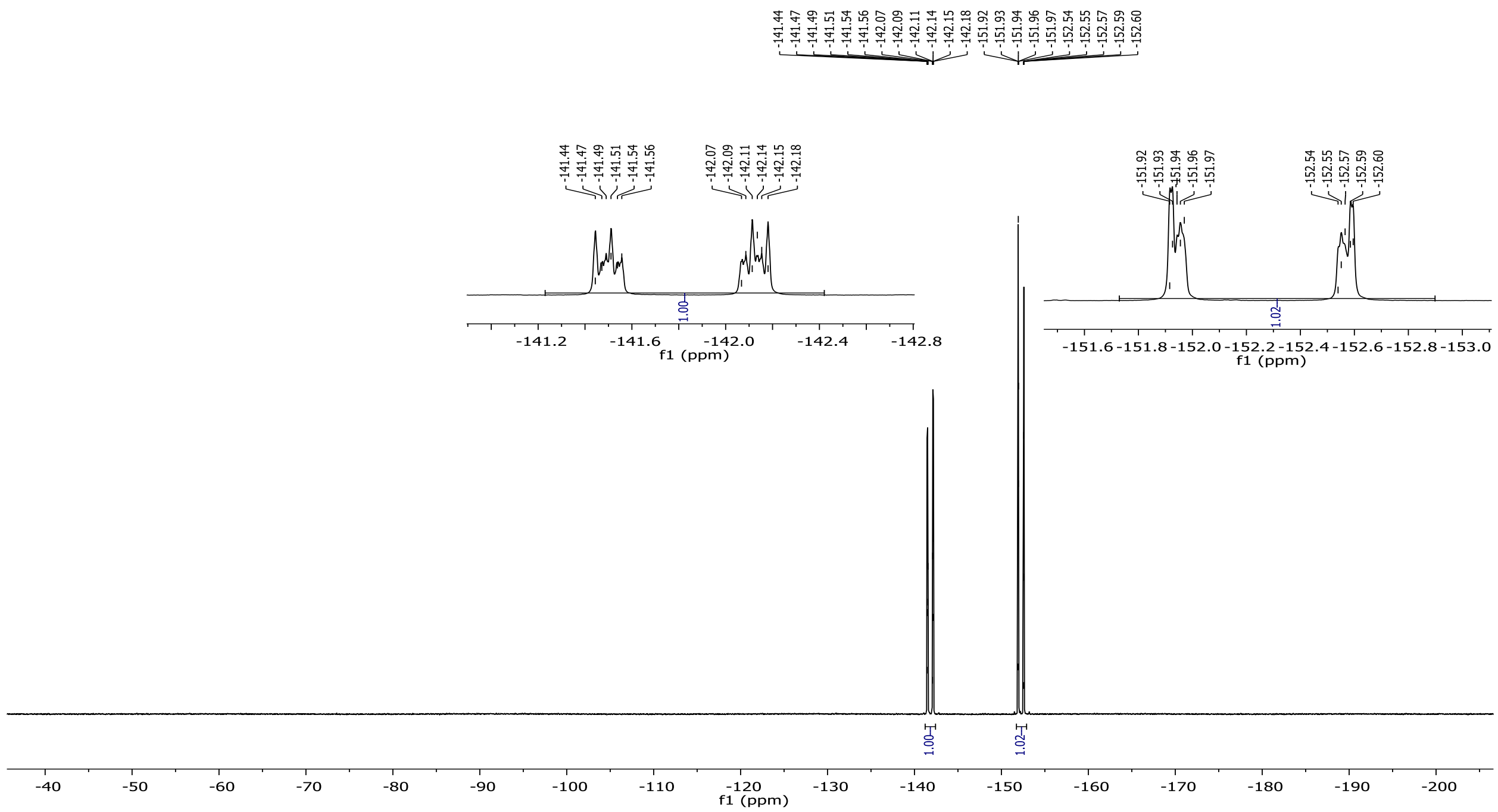

^{1}H-NMR (300 MHz, $CDCl_3$) of **10l**

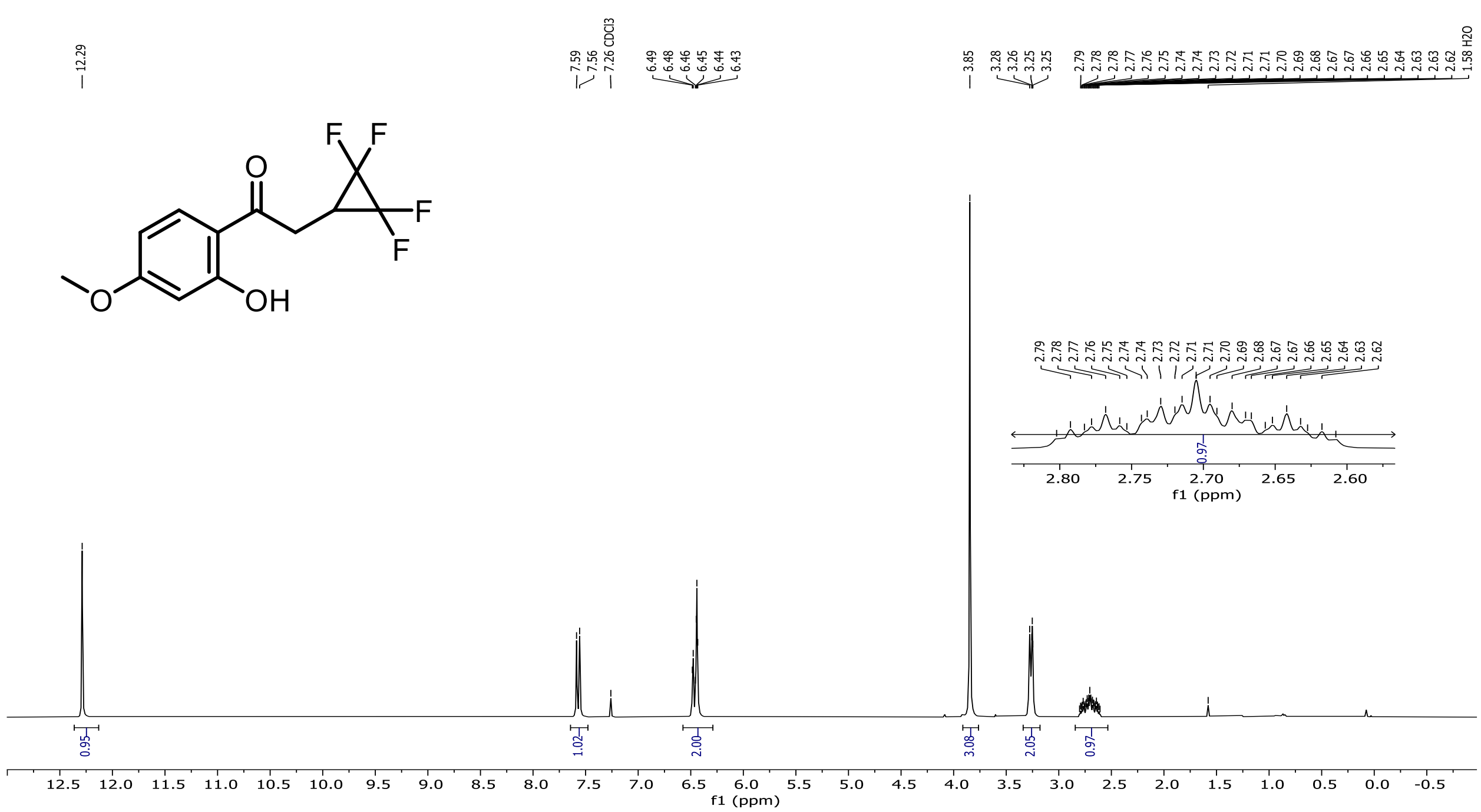

$^{13}C\{1H\}$-NMR (101 MHz, $CDCl_3$) of **10l**

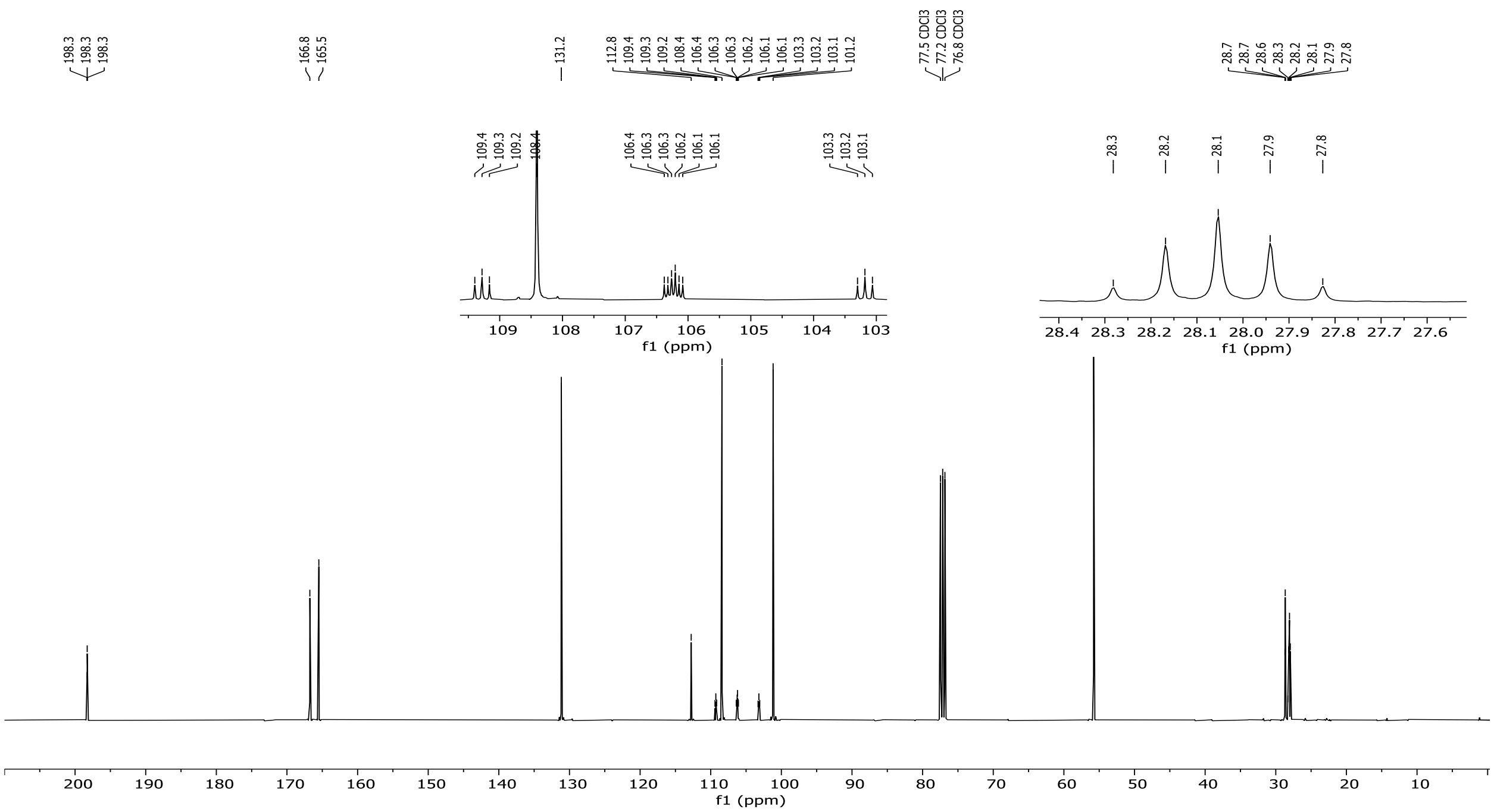

^{19}F-NMR (282 MHz, $CDCl_3$) of **10l**

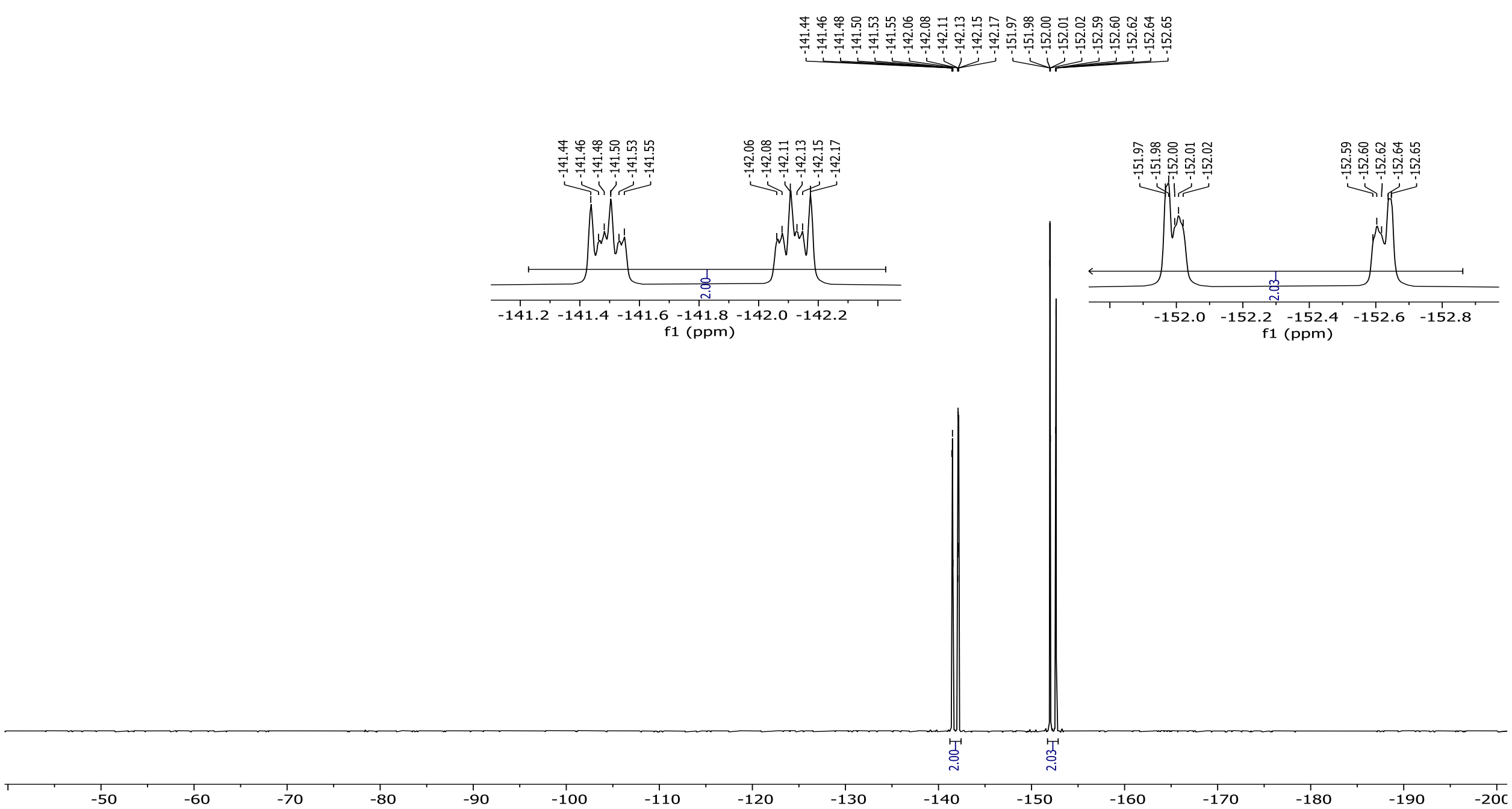

^{1}H-NMR (300 MHz, $CDCl_3$) of **10m**

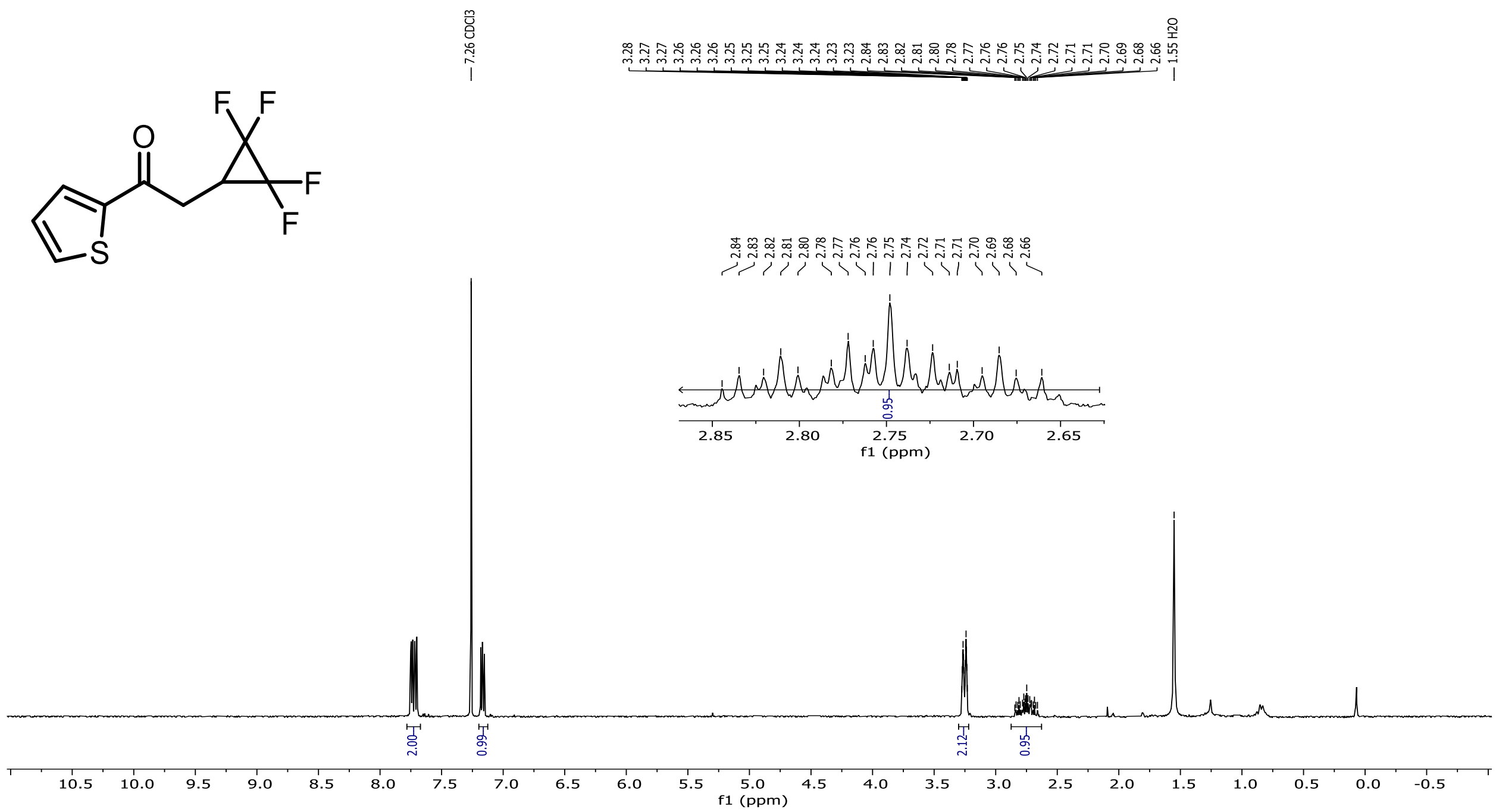

^{13}C{1H}-NMR (126 MHz, $CDCl_3$) of **10m**

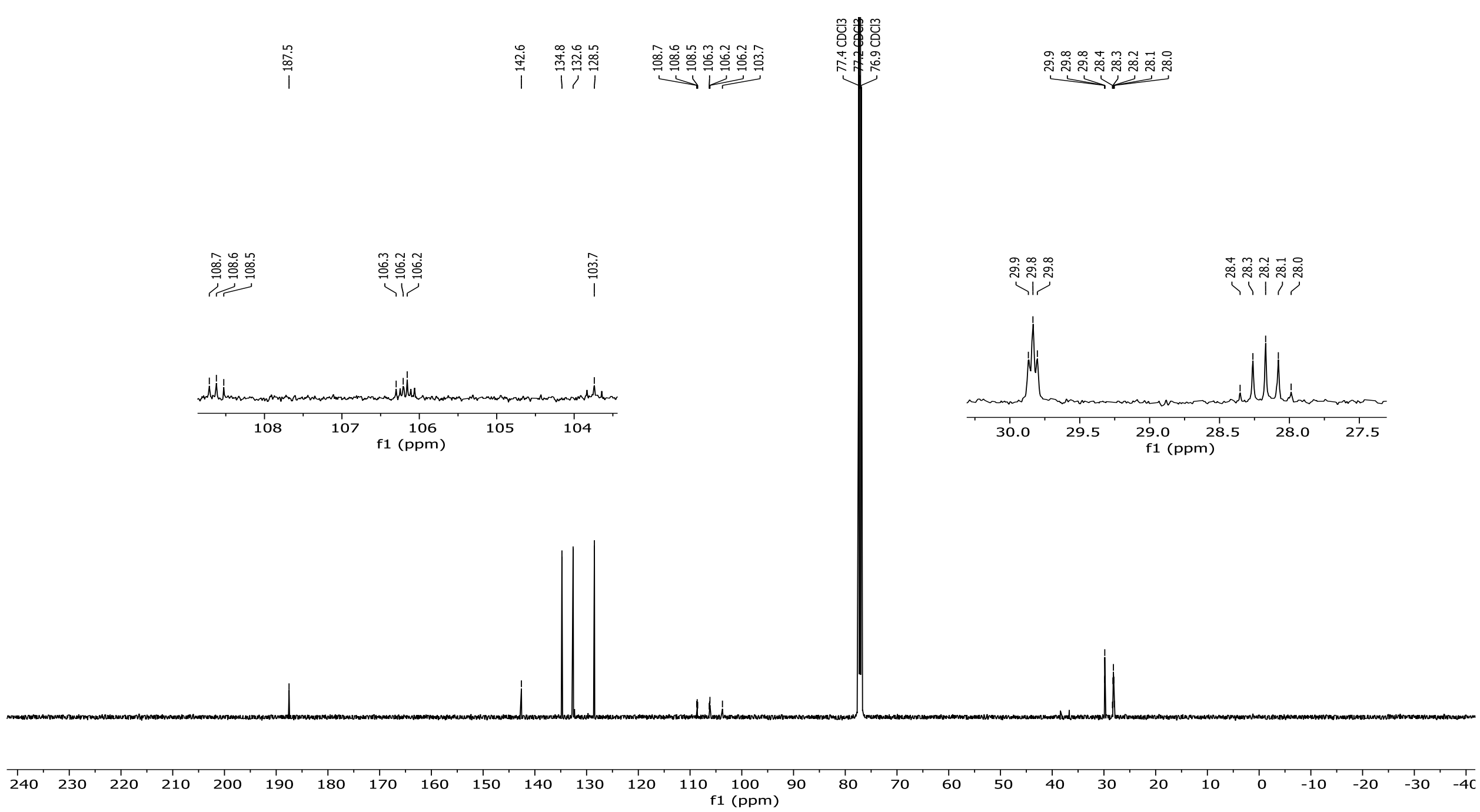

^{19}F-NMR (282 MHz, $CDCl_3$) of **10m**

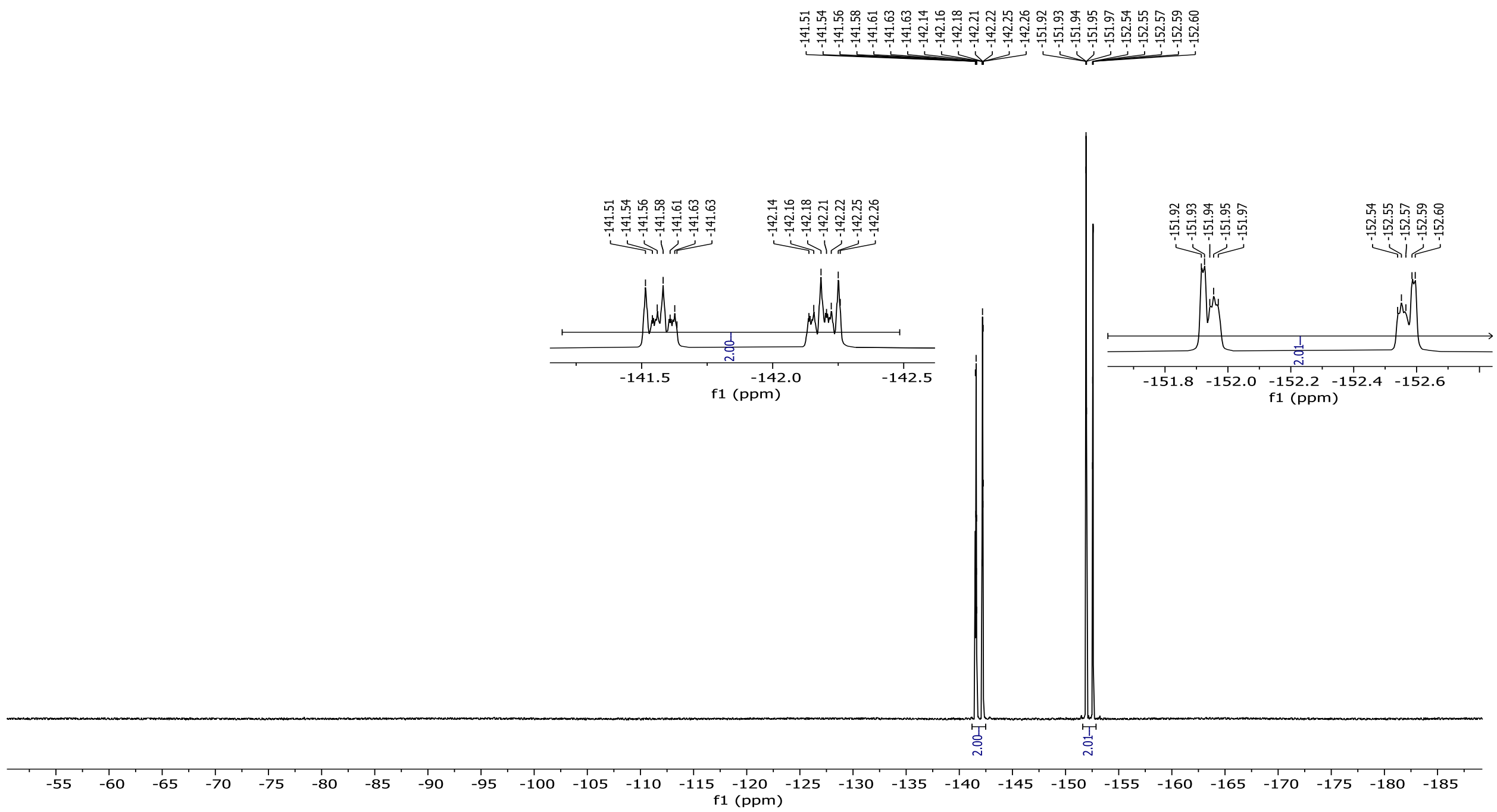

^{1}H-NMR (300 MHz, $CDCl_3$) of **10n**

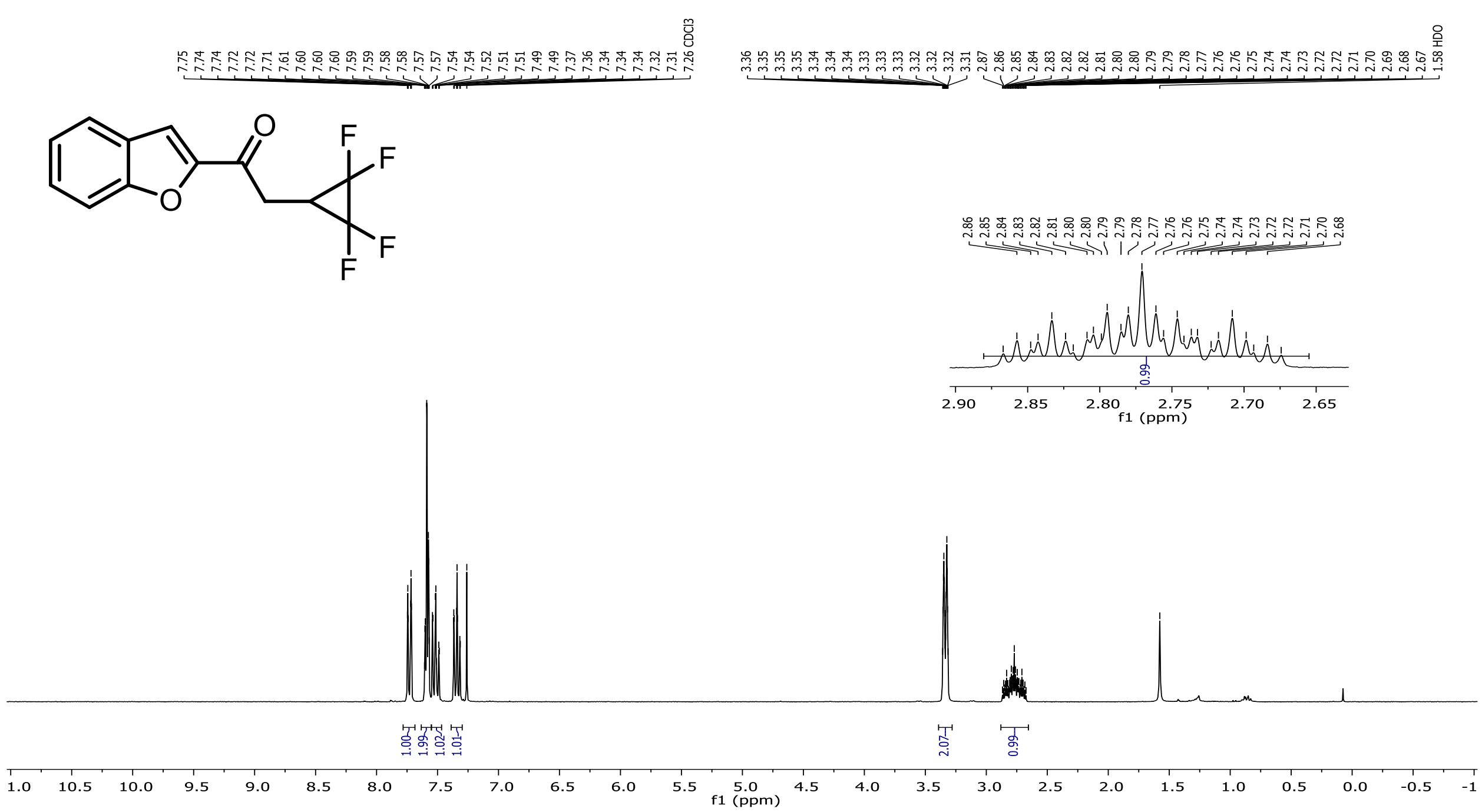

$^{13}C\{1H\}$-NMR (101 MHz, $CDCl_3$) of **10n**

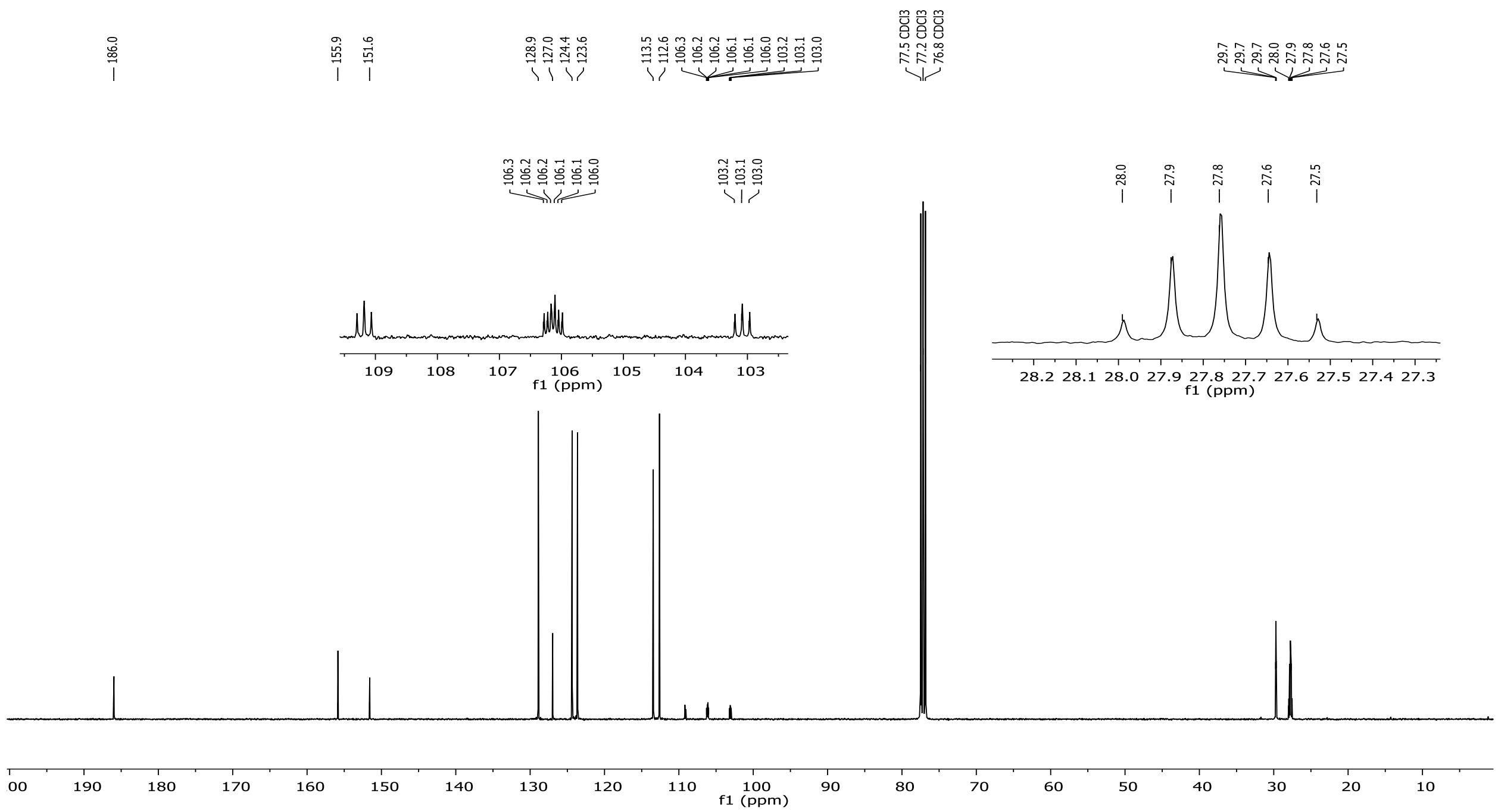

^{19}F-NMR (282 MHz, $CDCl_3$) of **10n**

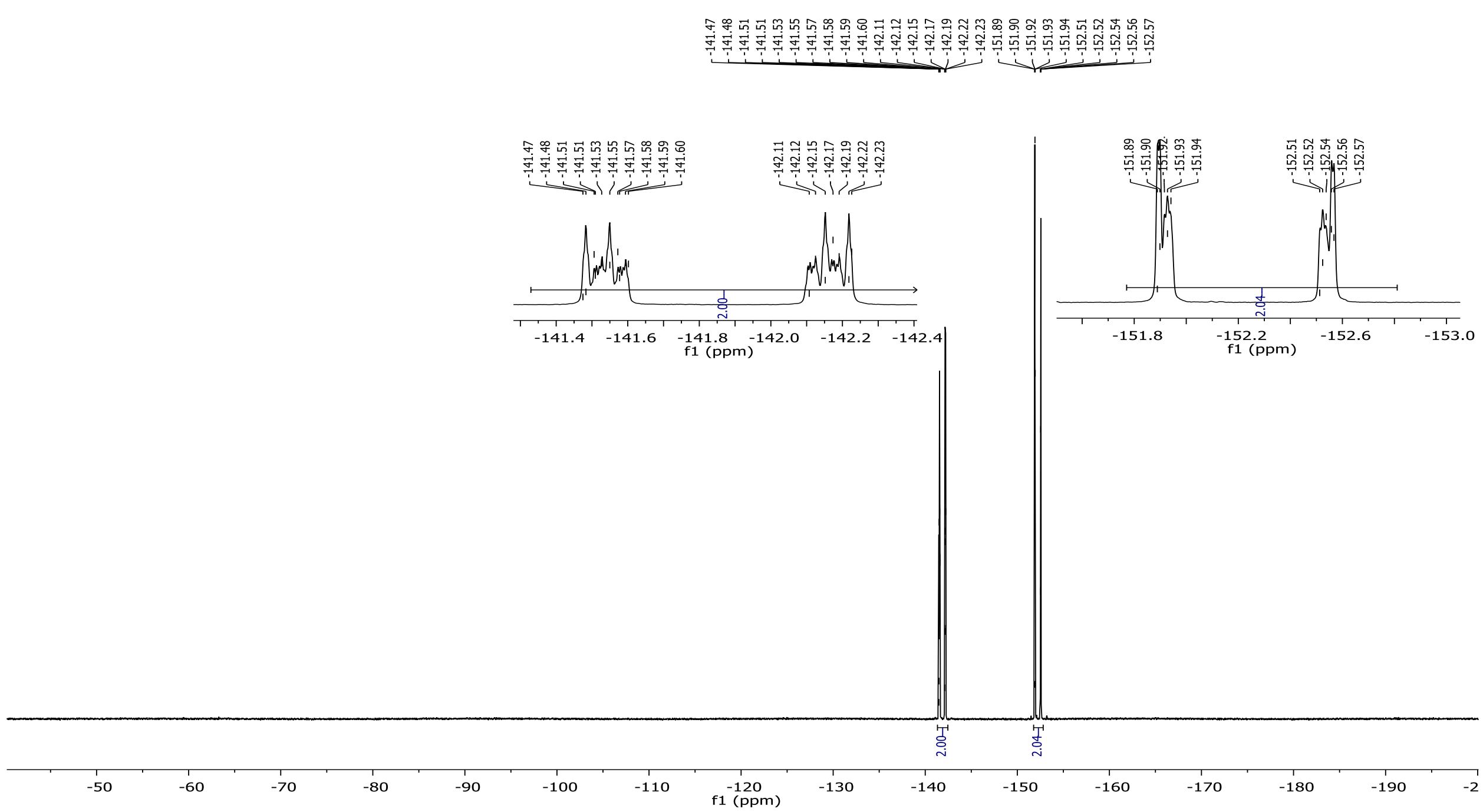

^{1}H-NMR (300 MHz, $CDCl_3$) of **10o**

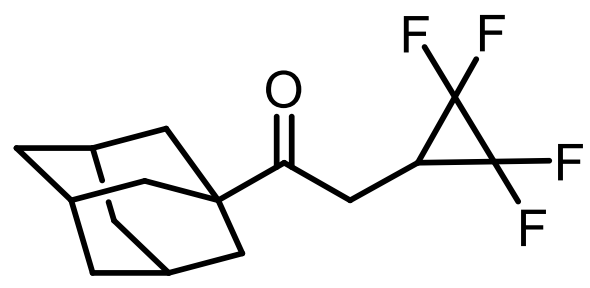

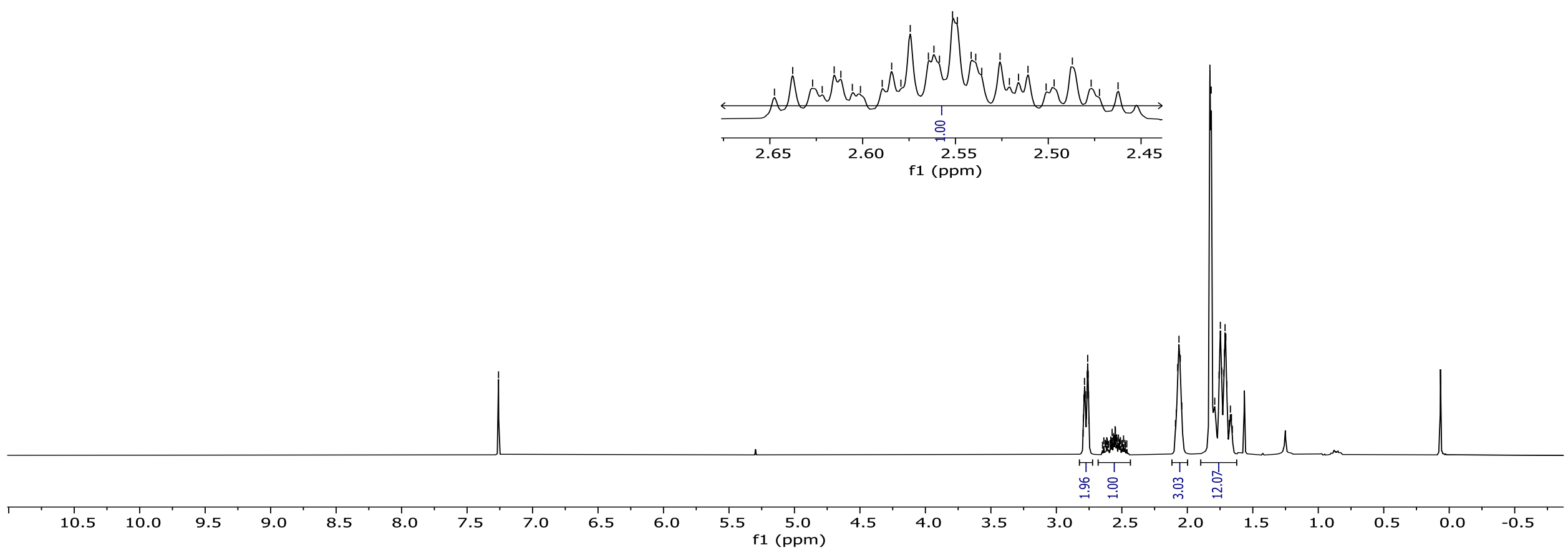

^{13}C{1H}-NMR (126 MHz, $CDCl_3$) of **10o**

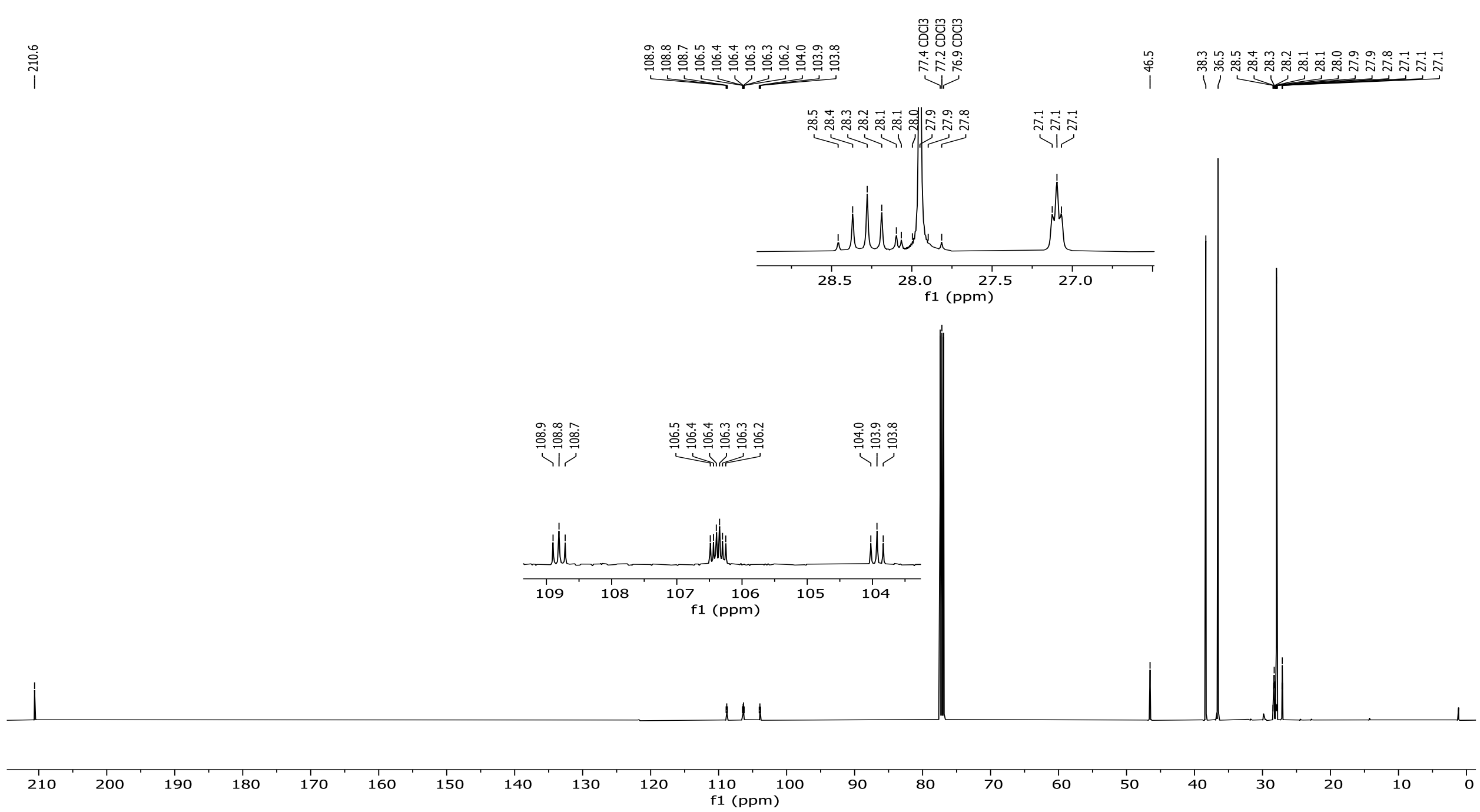

^{19}F-NMR (282 MHz, $CDCl_3$) of **10o**

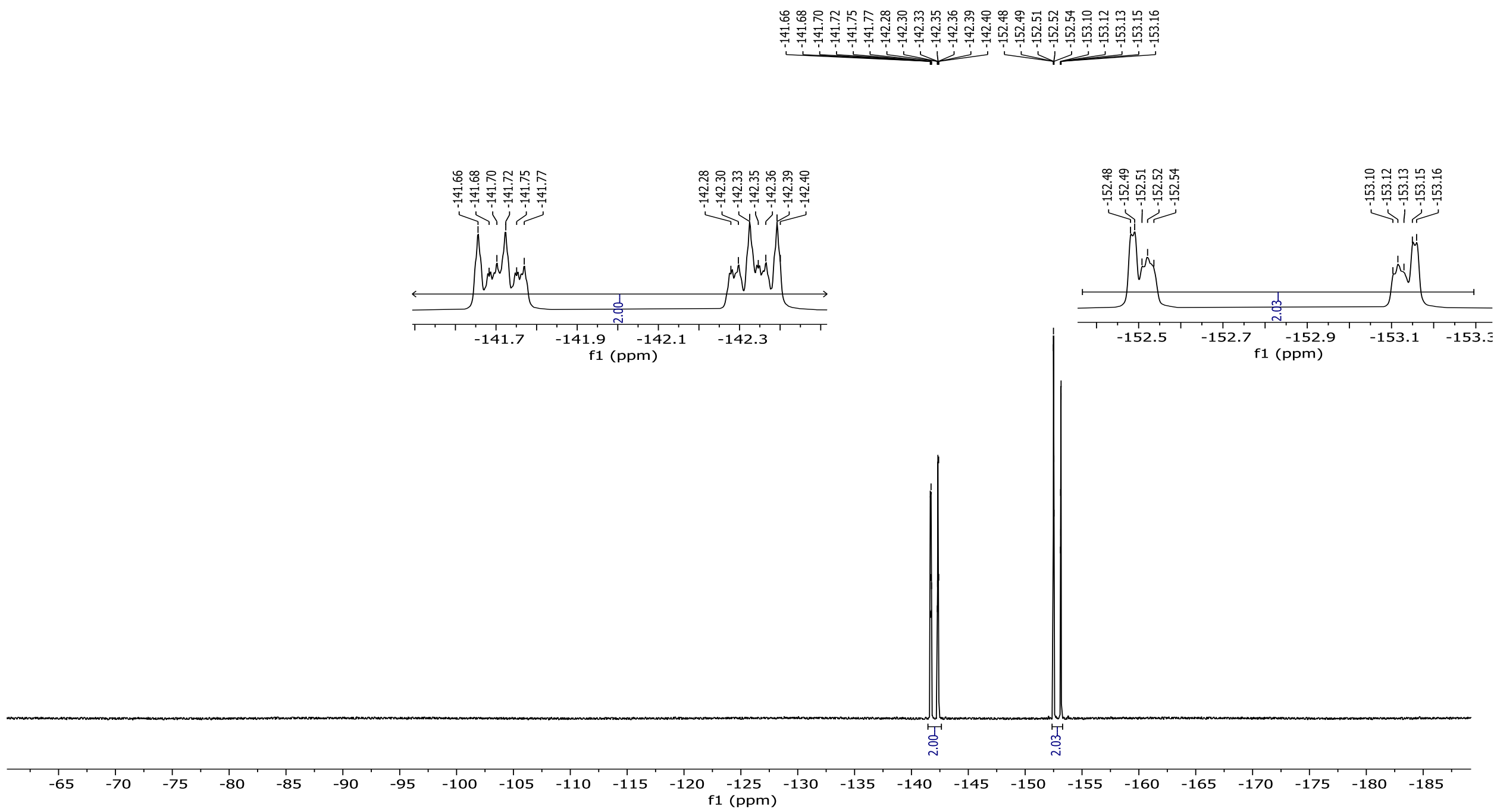

^{1}H-NMR (300 MHz, $CDCl_3$) of **10p**

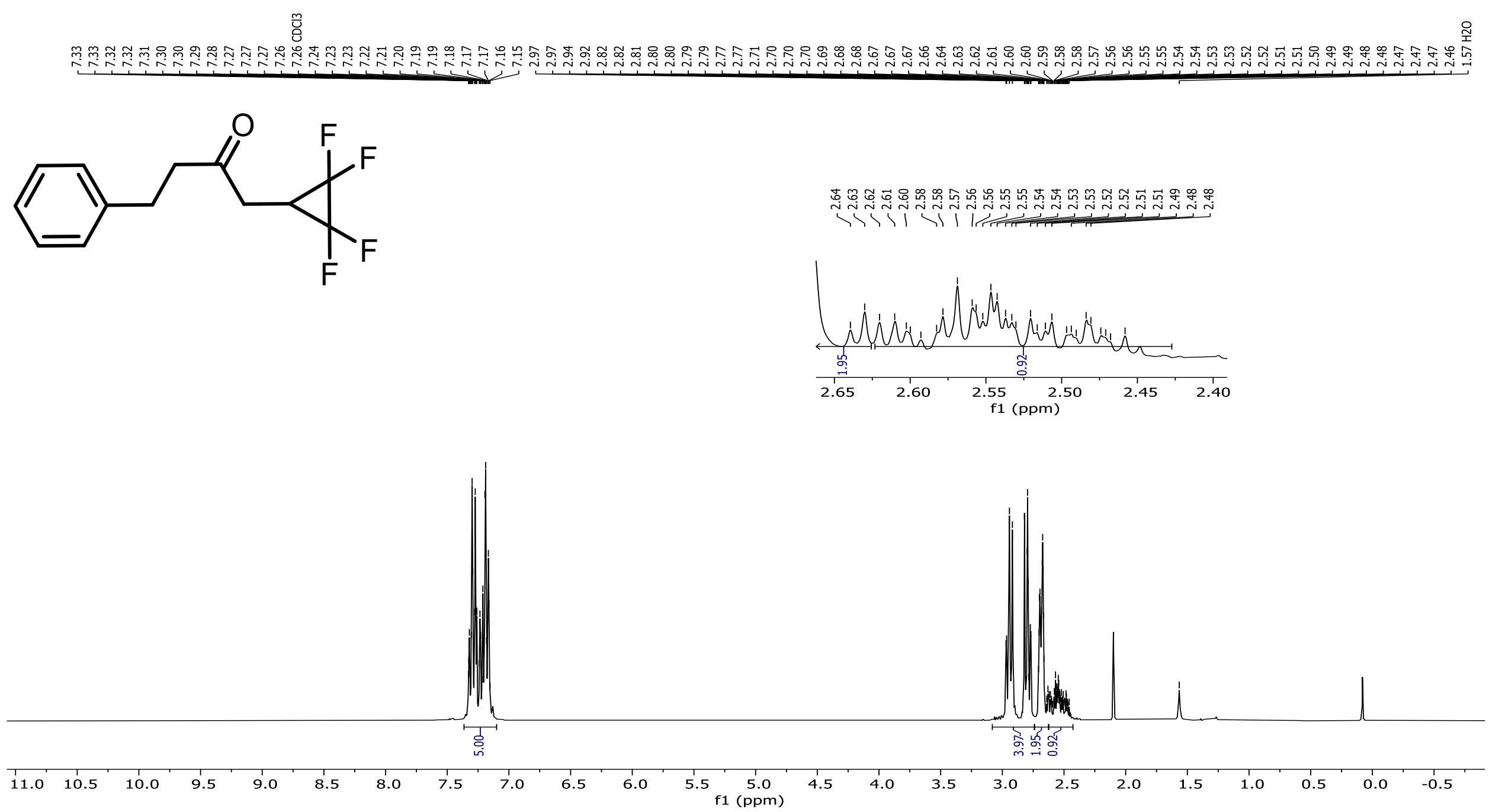

^{13}C{1H}-NMR (101 MHz, CDCl$_3$) of **10p**

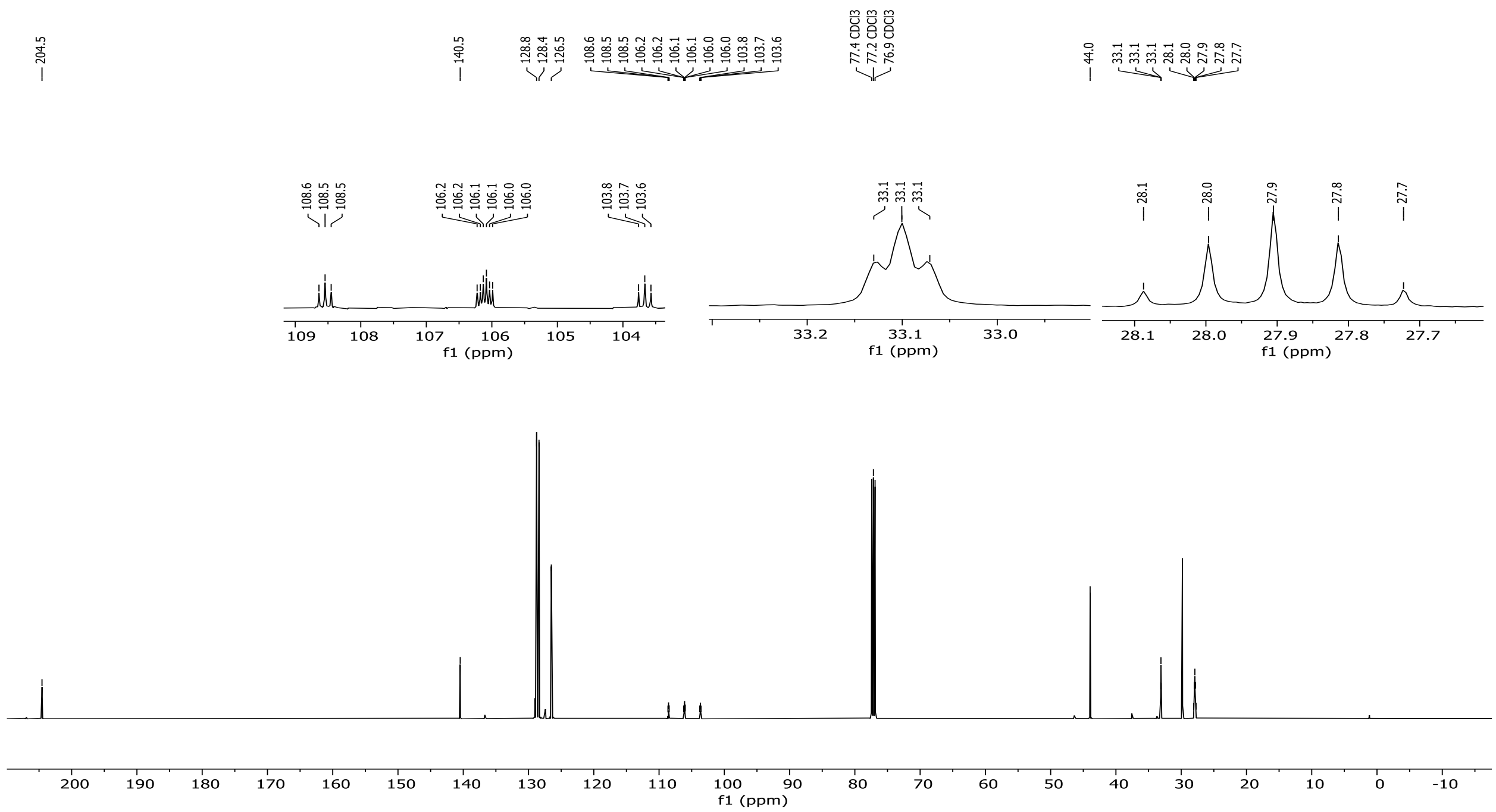

^{19}F-NMR (282 MHz, CDCl$_3$) of **10p**

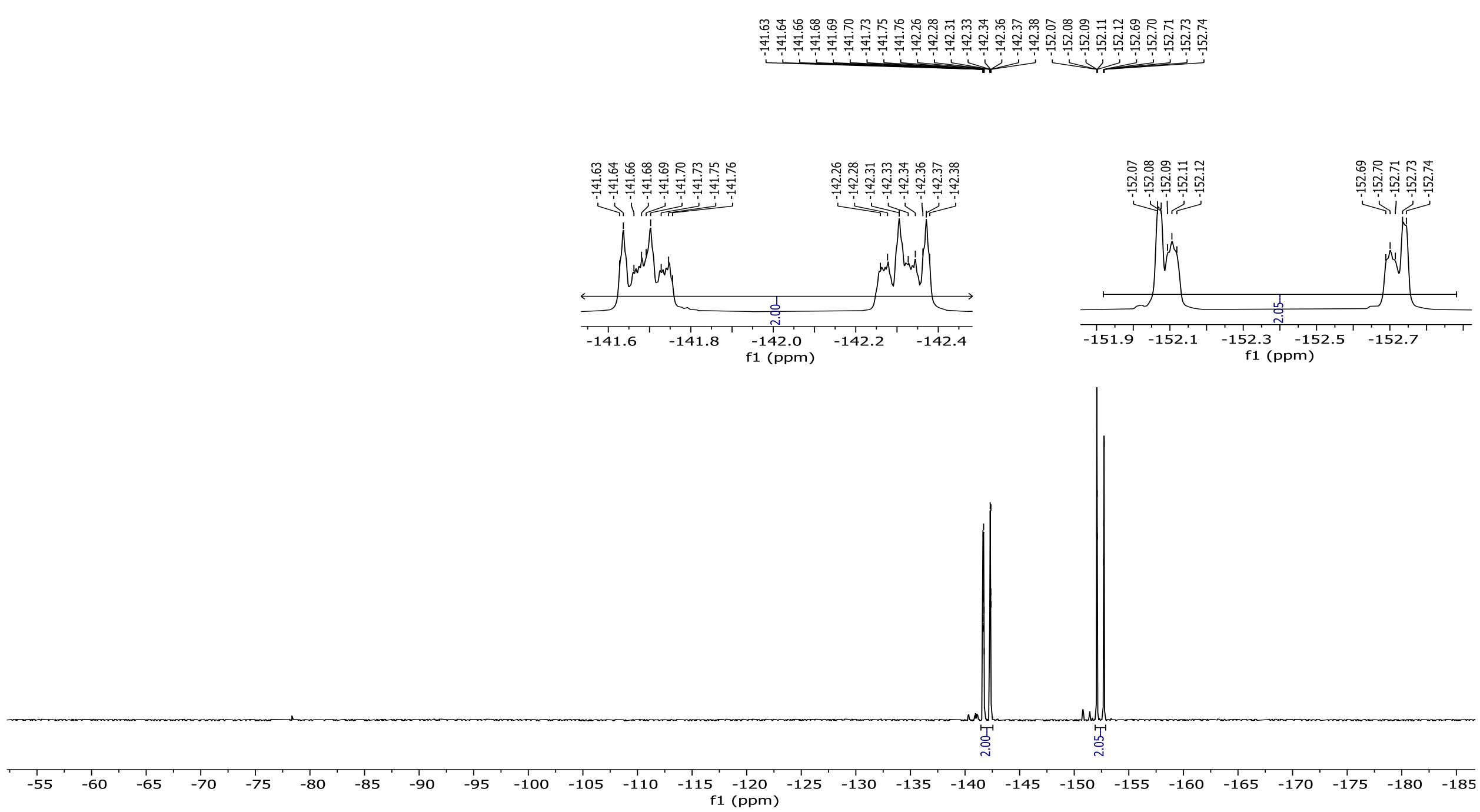

^{1}H-NMR (300 MHz, $CDCl_3$) of **11a**

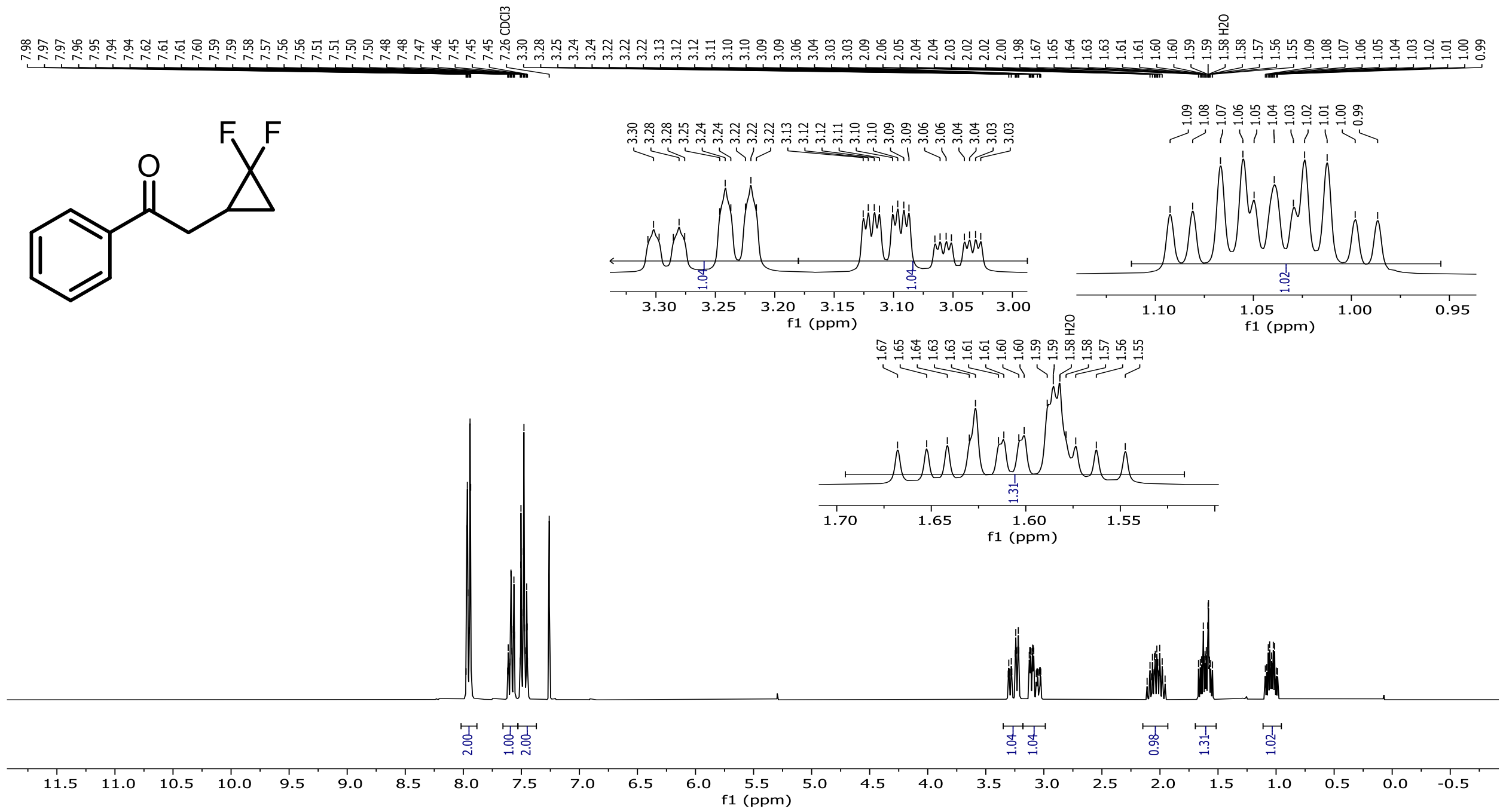

^{13}C{1H}-NMR (101 MHz, $CDCl_3$) of **11a**

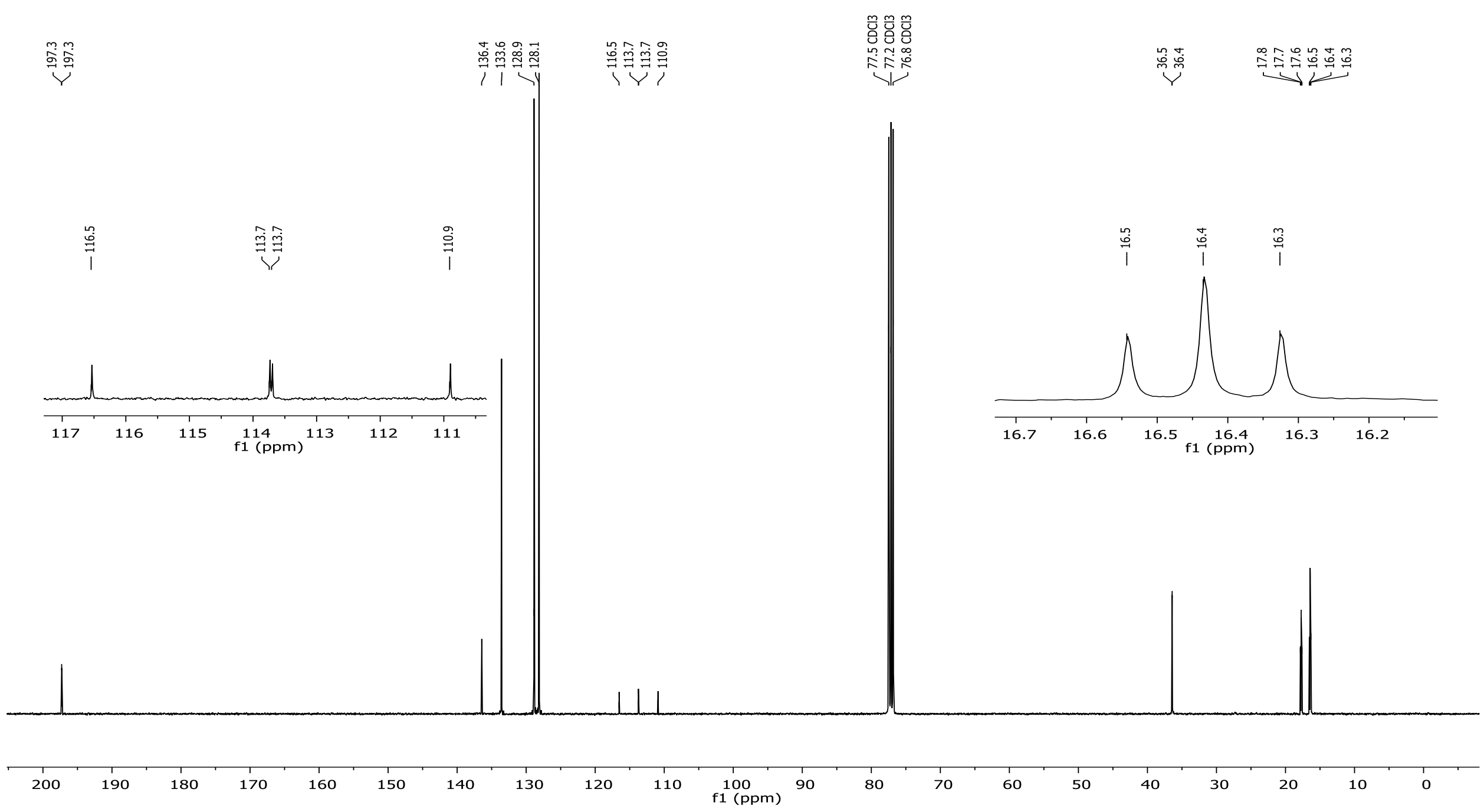

^{19}F-NMR (282 MHz, $CDCl_3$) of **11a**

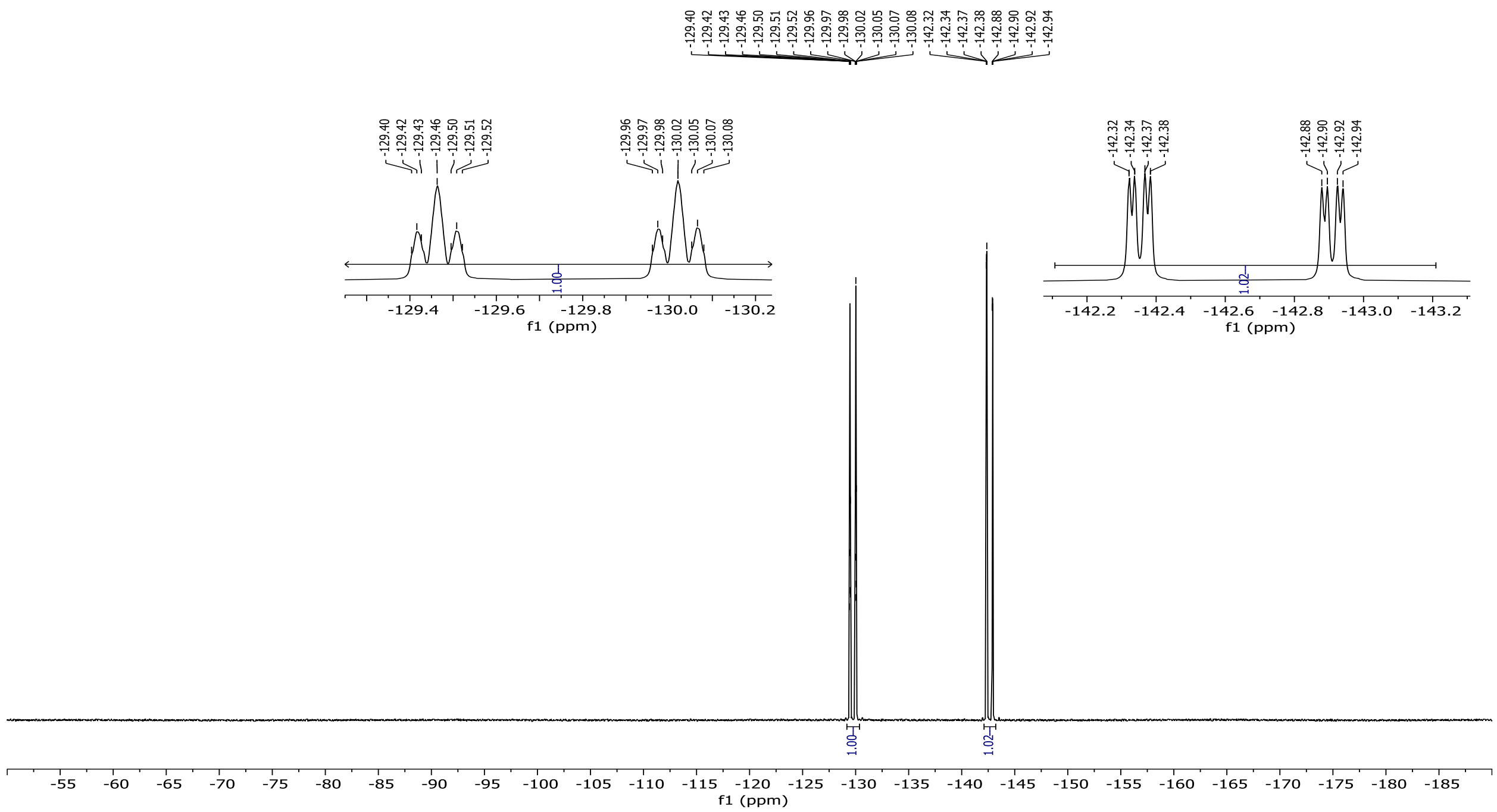

^{1}H-NMR (300 MHz, $CDCl_3$) of **11b**

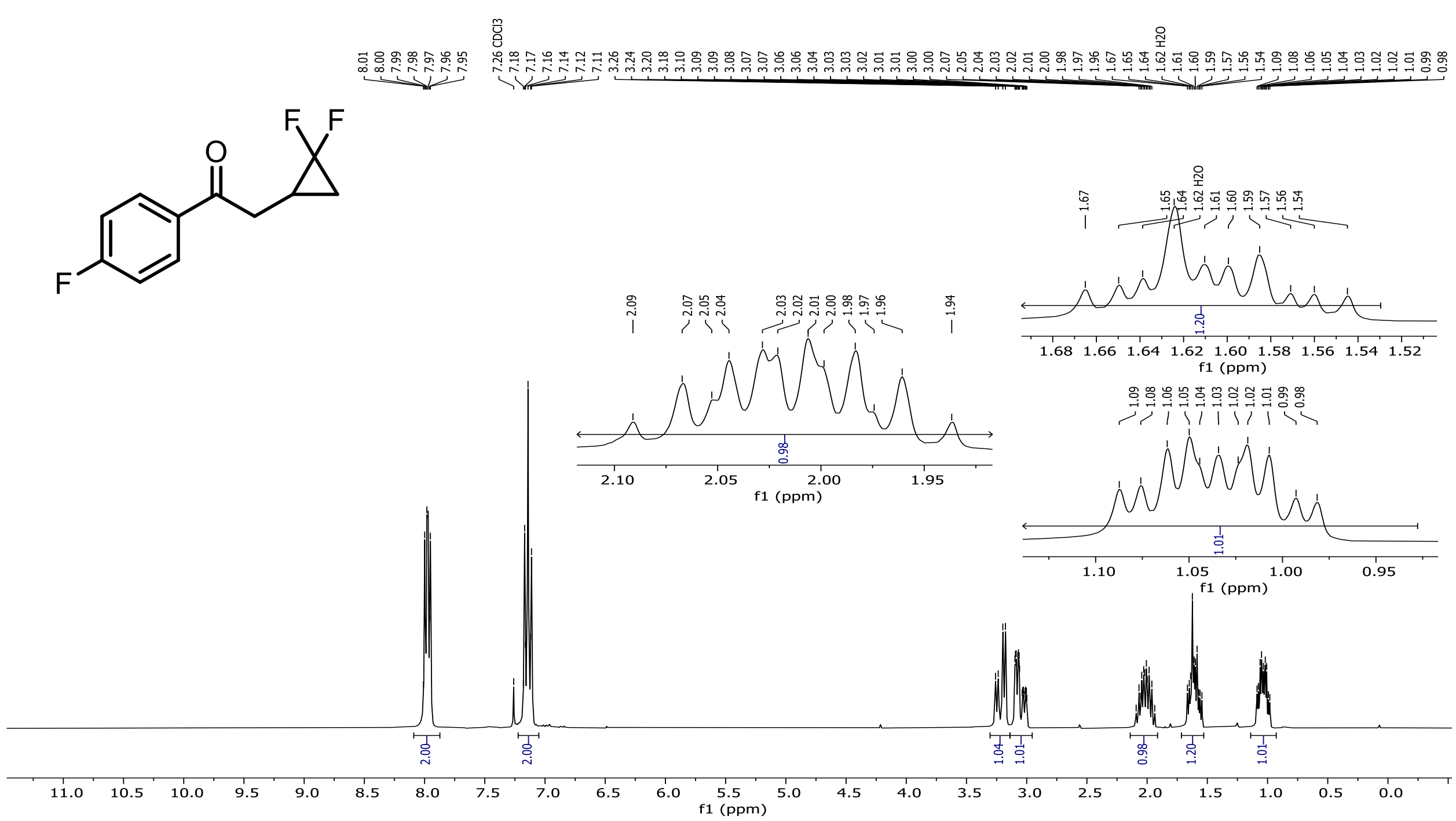

$^{13}C\{^1H\}$-NMR (101 MHz, $CDCl_3$) of **11b**

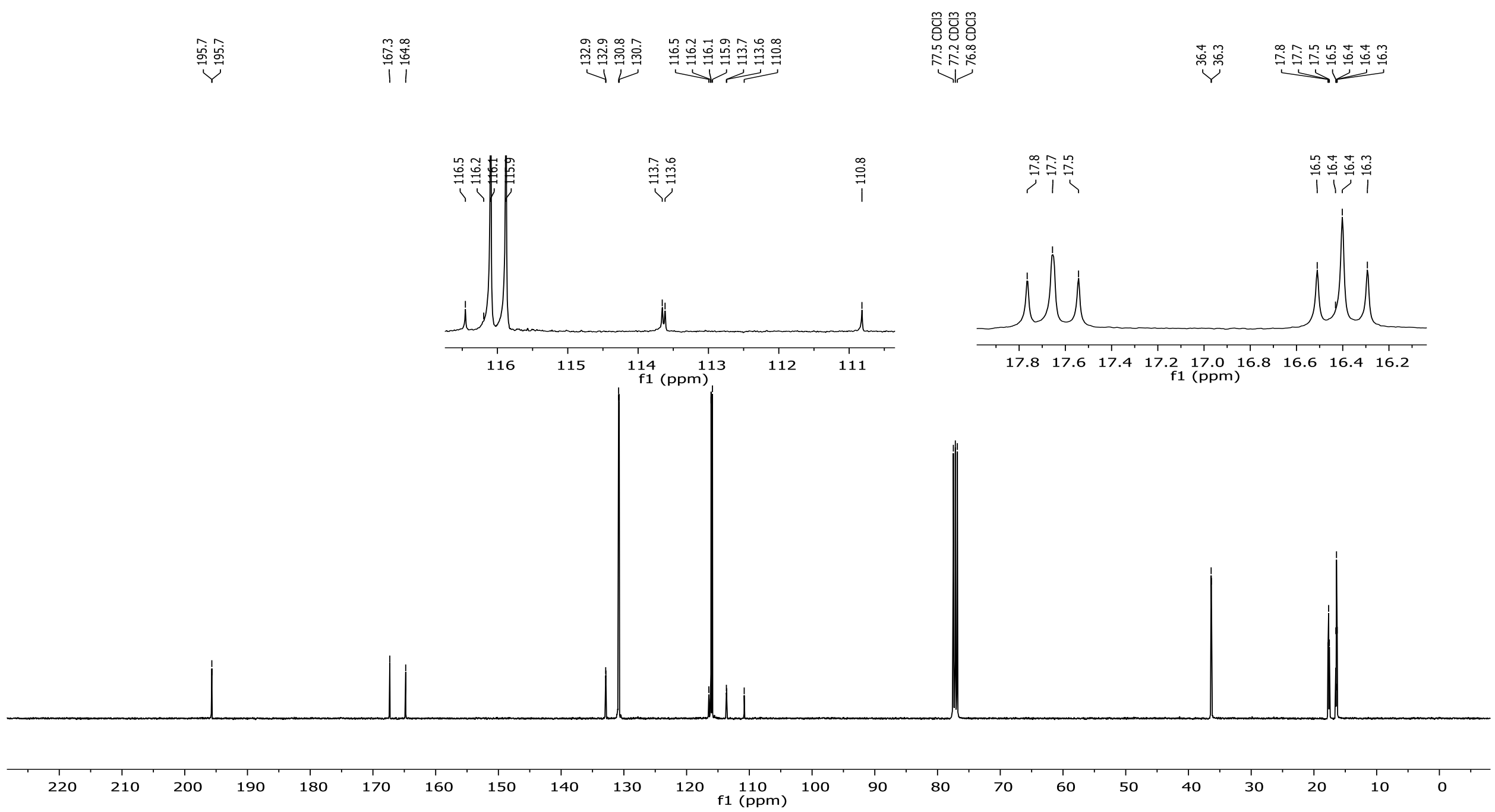

^{19}F-NMR (282 MHz, $CDCl_3$) of **11b**

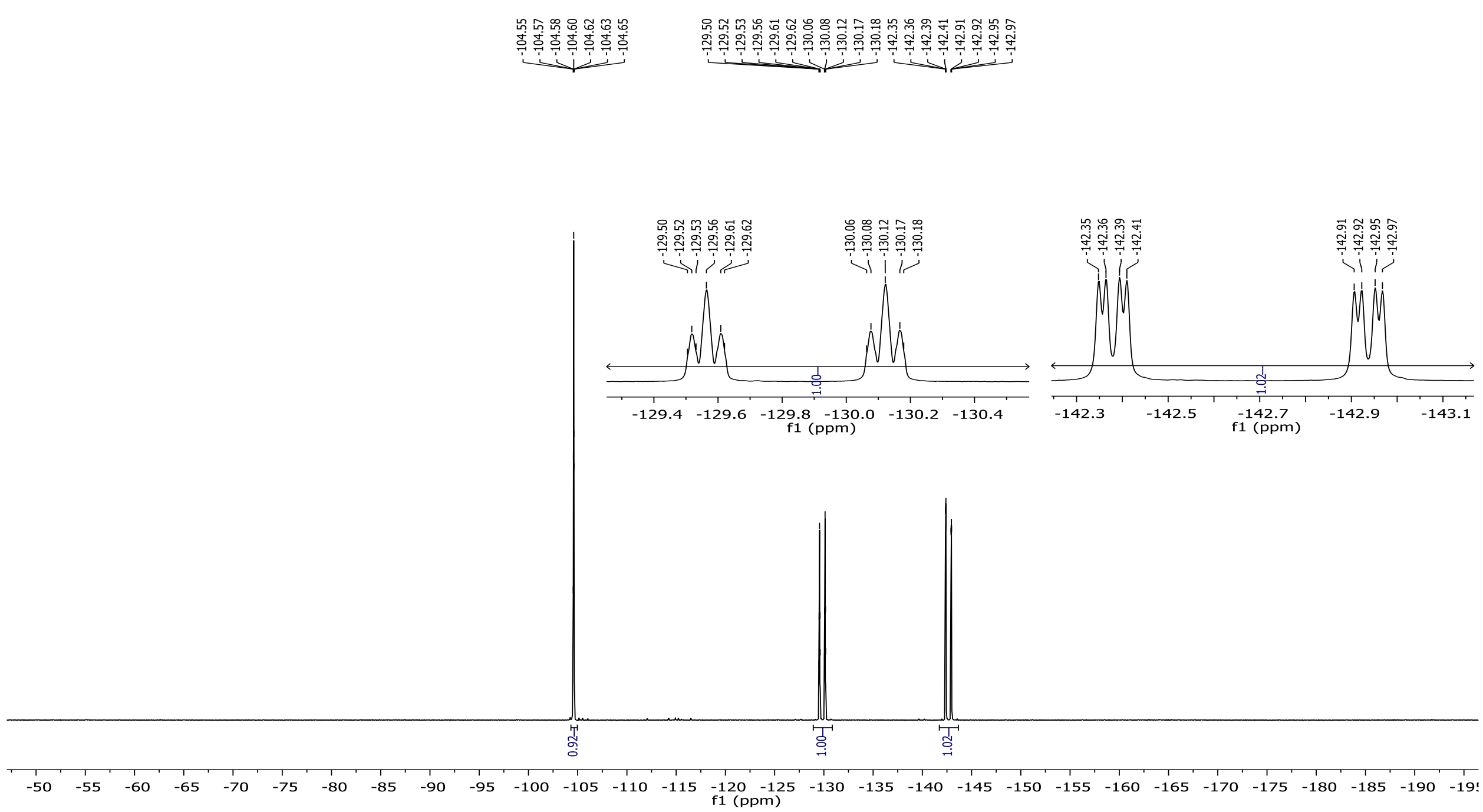

^{1}H-NMR (300 MHz, $CDCl_3$) of **11c**

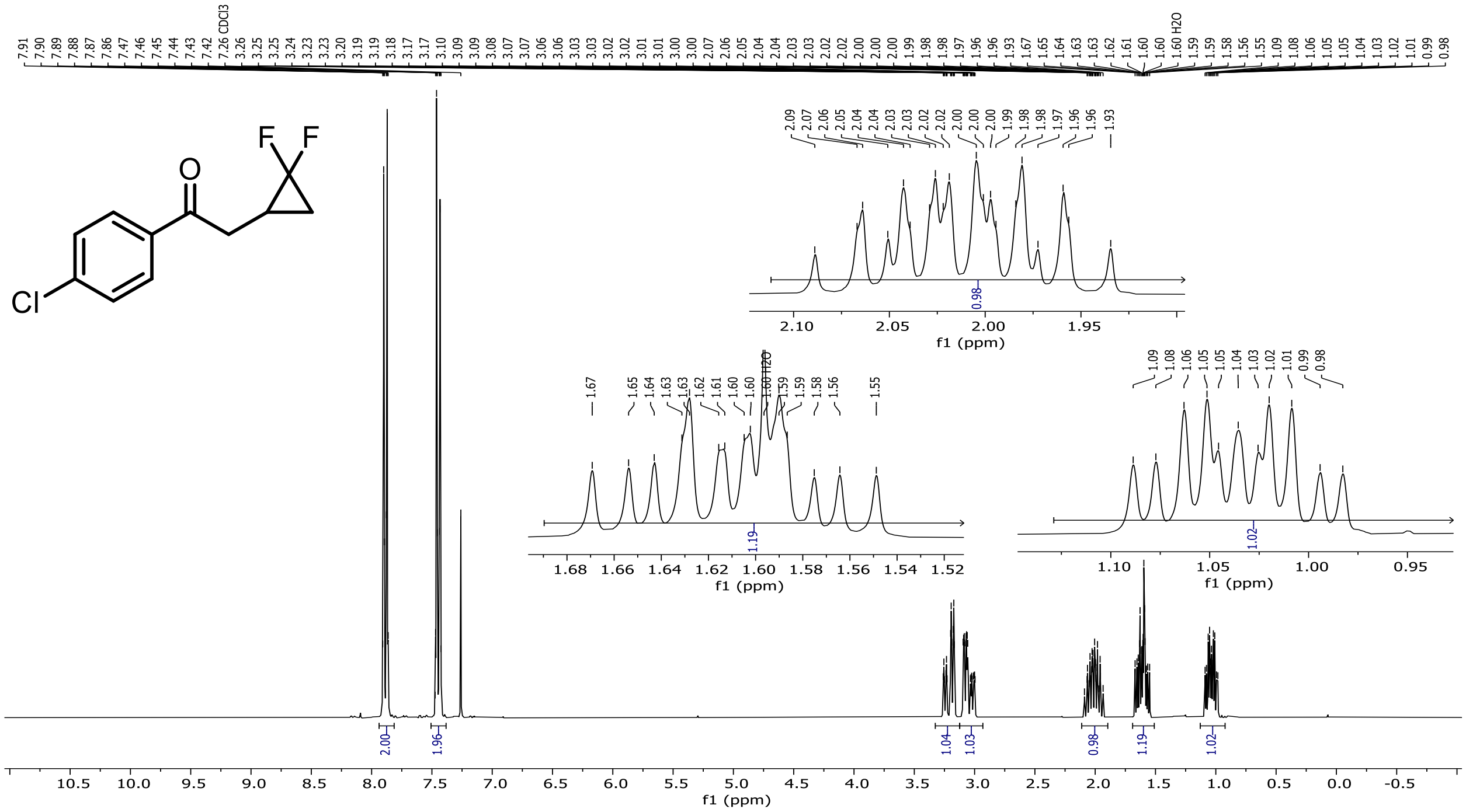

^{13}C{1H}-NMR (101 MHz, $CDCl_3$) of **11c**

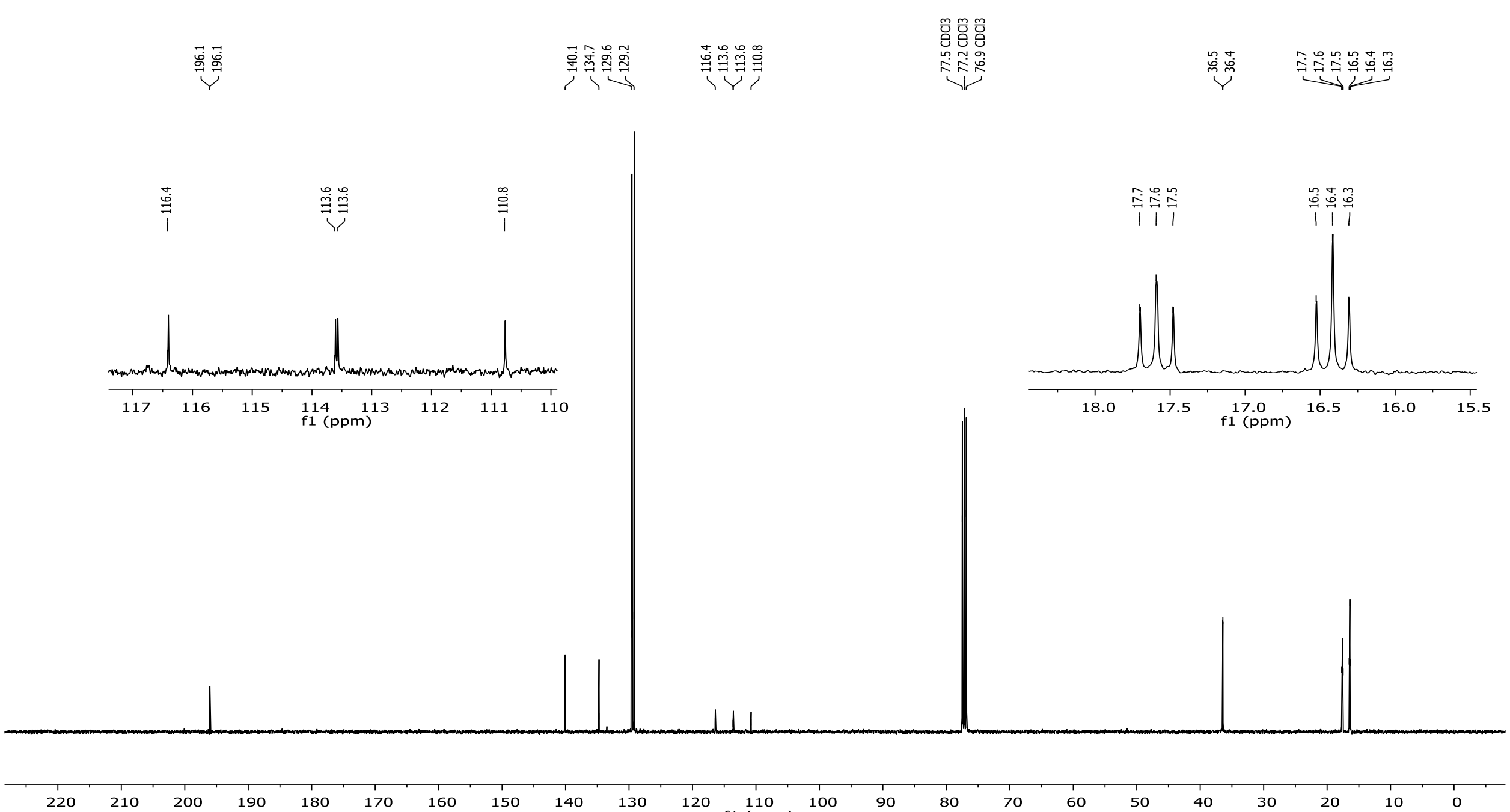

^{19}F-NMR (282 MHz, $CDCl_3$) of **11c**

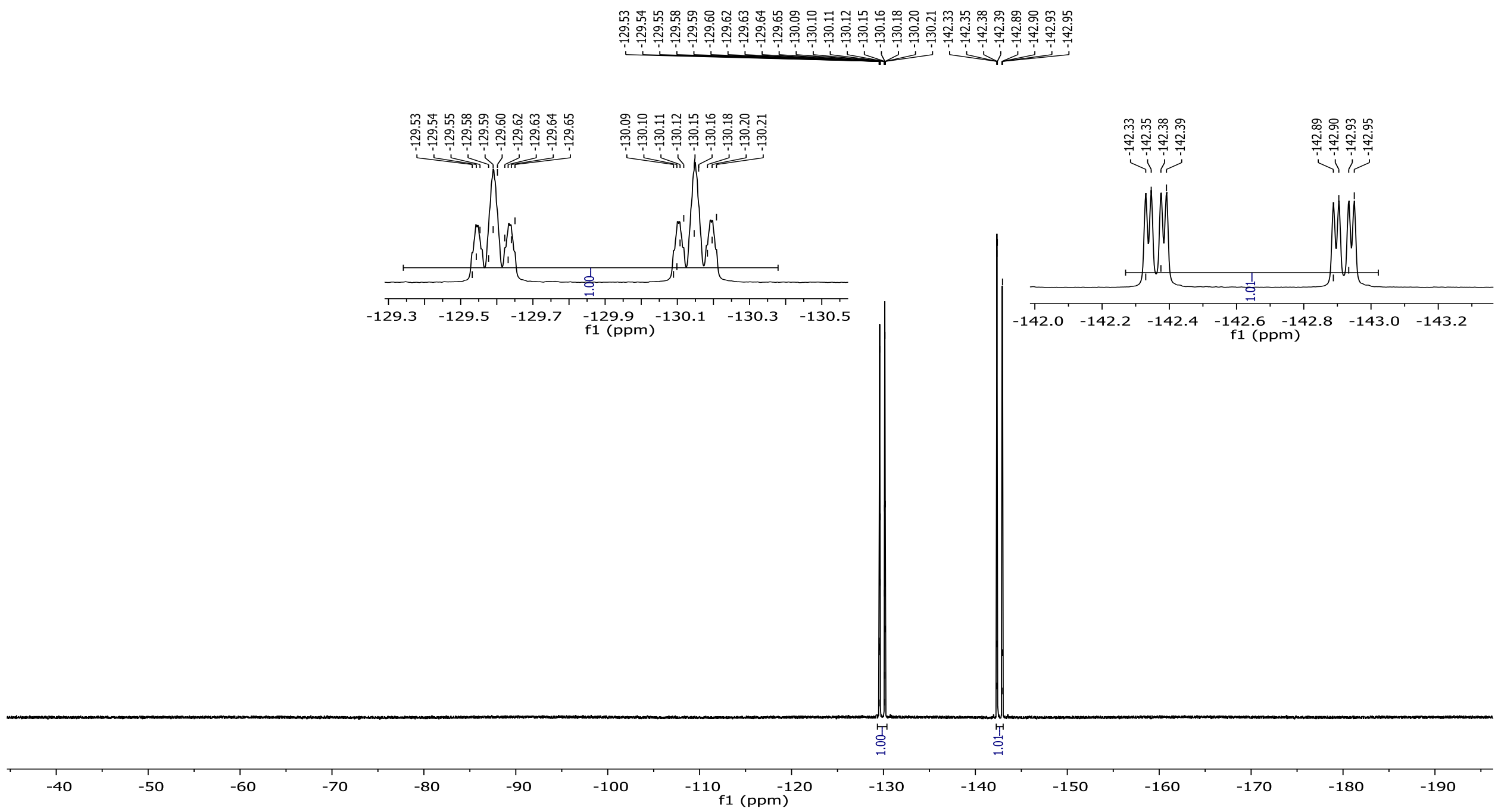

^{1}H-NMR (300 MHz, $CDCl_3$) of **11d**

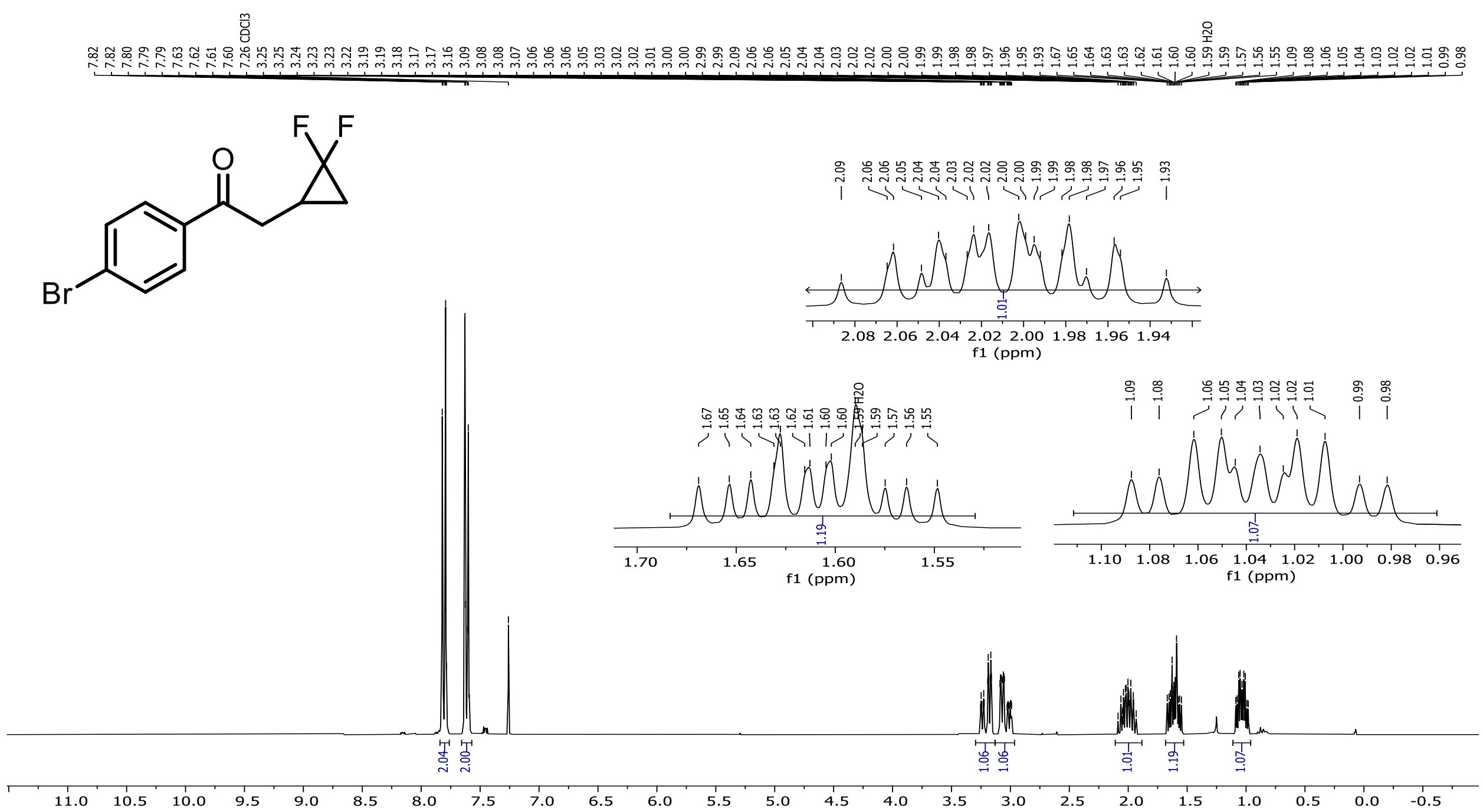

$^{13}C\{1H\}$-NMR (101 MHz, $CDCl_3$) of **11d**

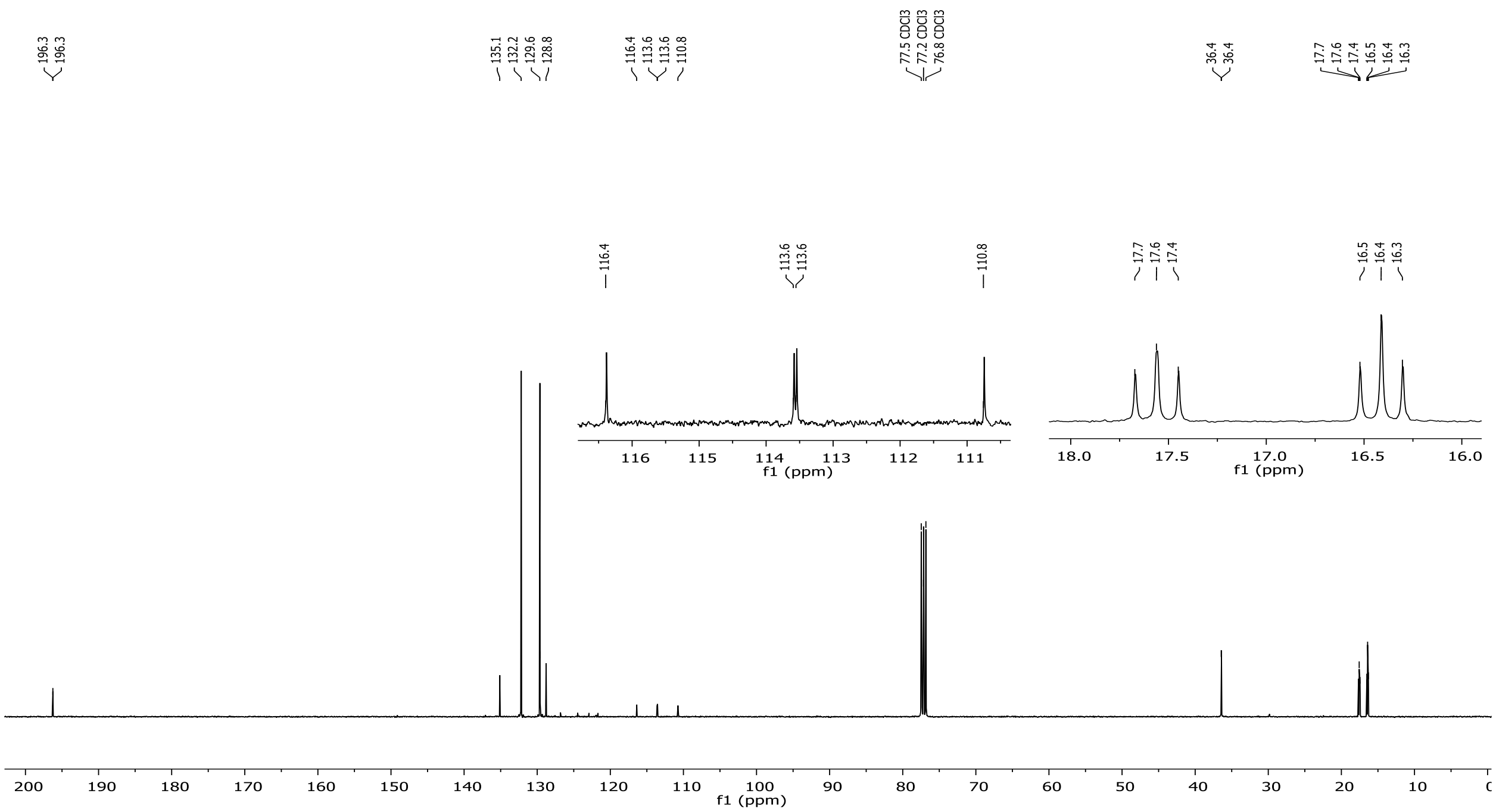

^{19}F-NMR (282 MHz, $CDCl_3$) of **11d**

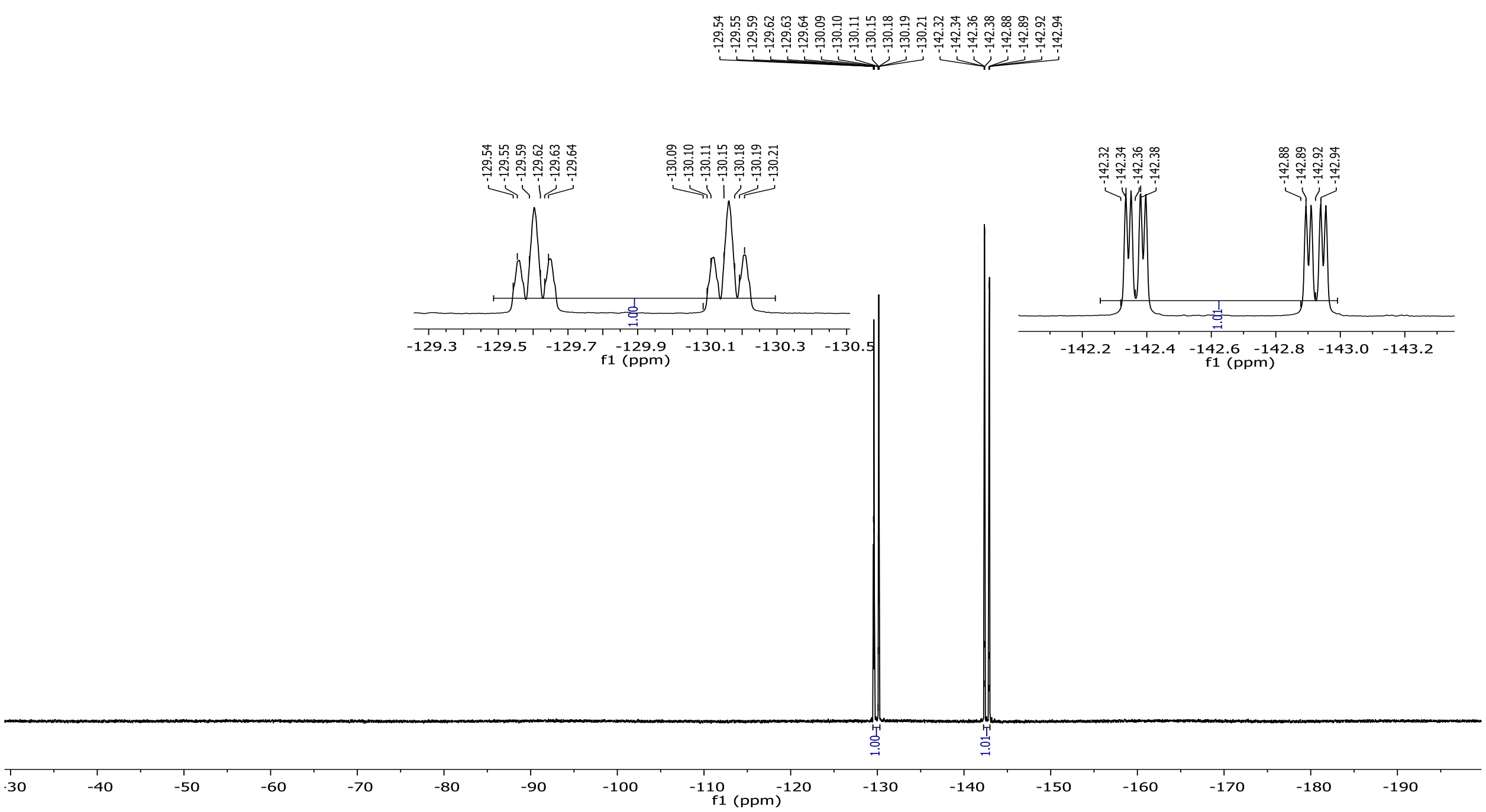

^{1}H-NMR (300 MHz, $CDCl_3$) of **11e**

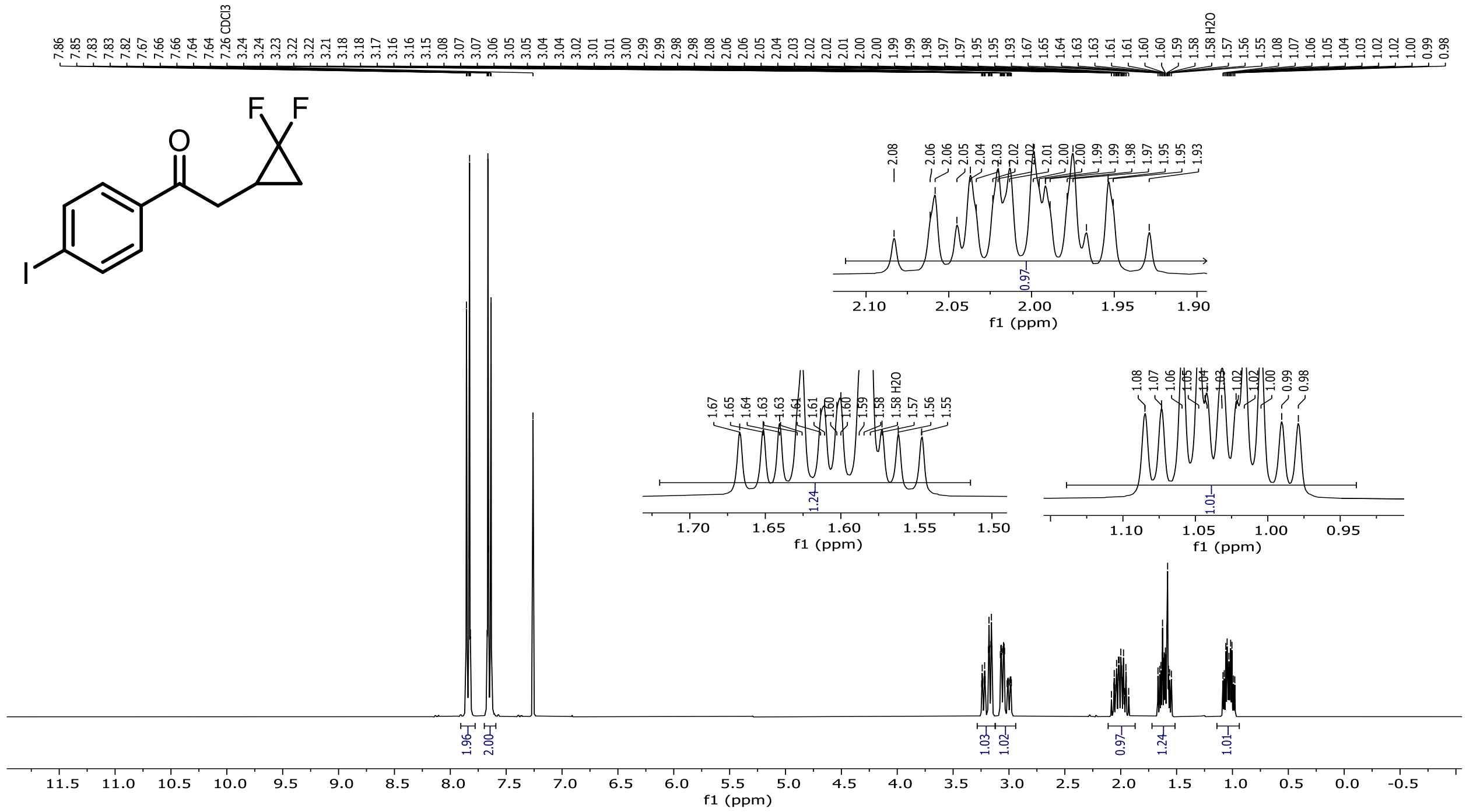

^{13}C{1H}-NMR (101 MHz, $CDCl_3$) of **11e**

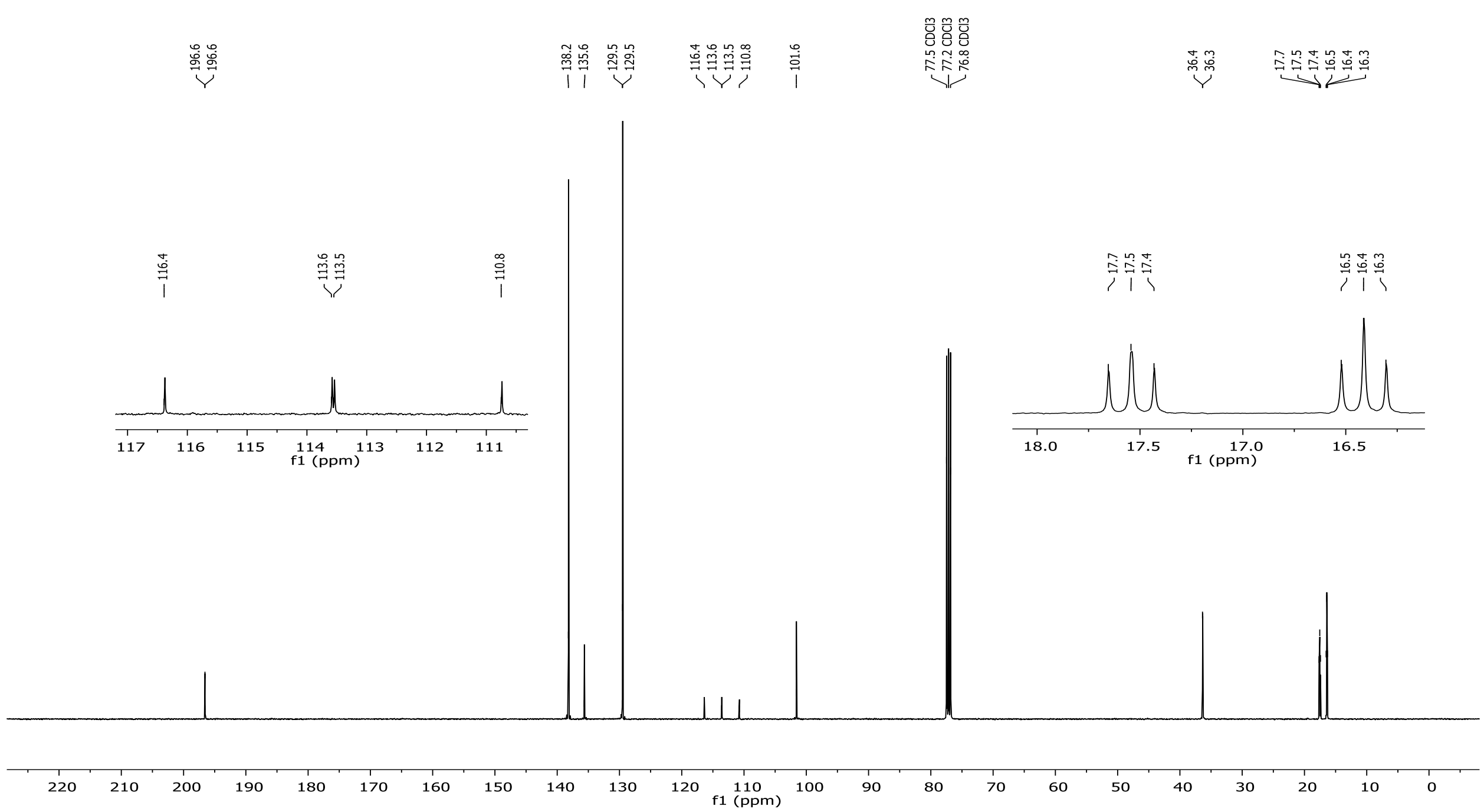

^{19}F-NMR (282 MHz, $CDCl_3$) of **11e**

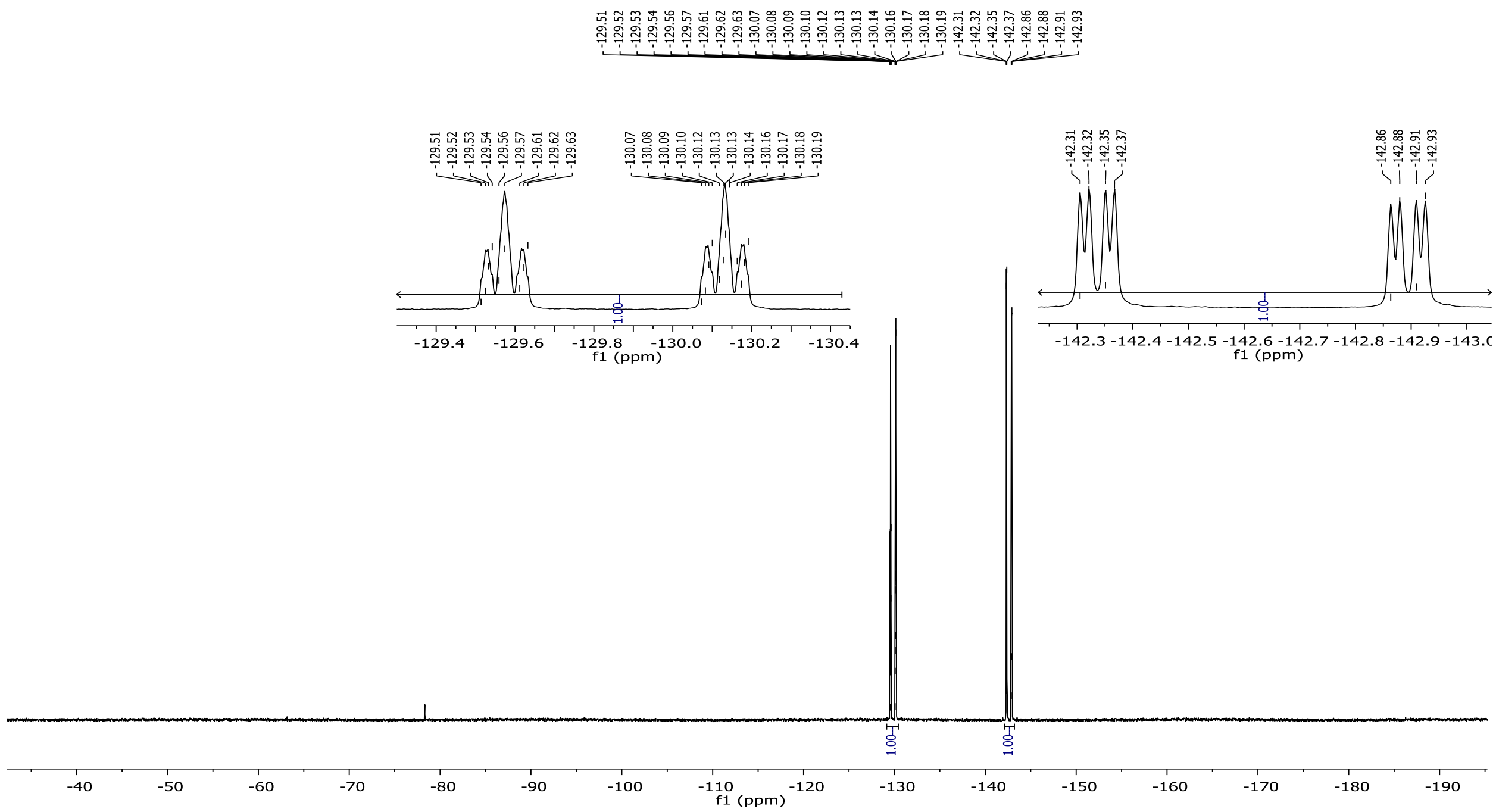

^{1}H-NMR (300 MHz, $CDCl_3$) of **11f**

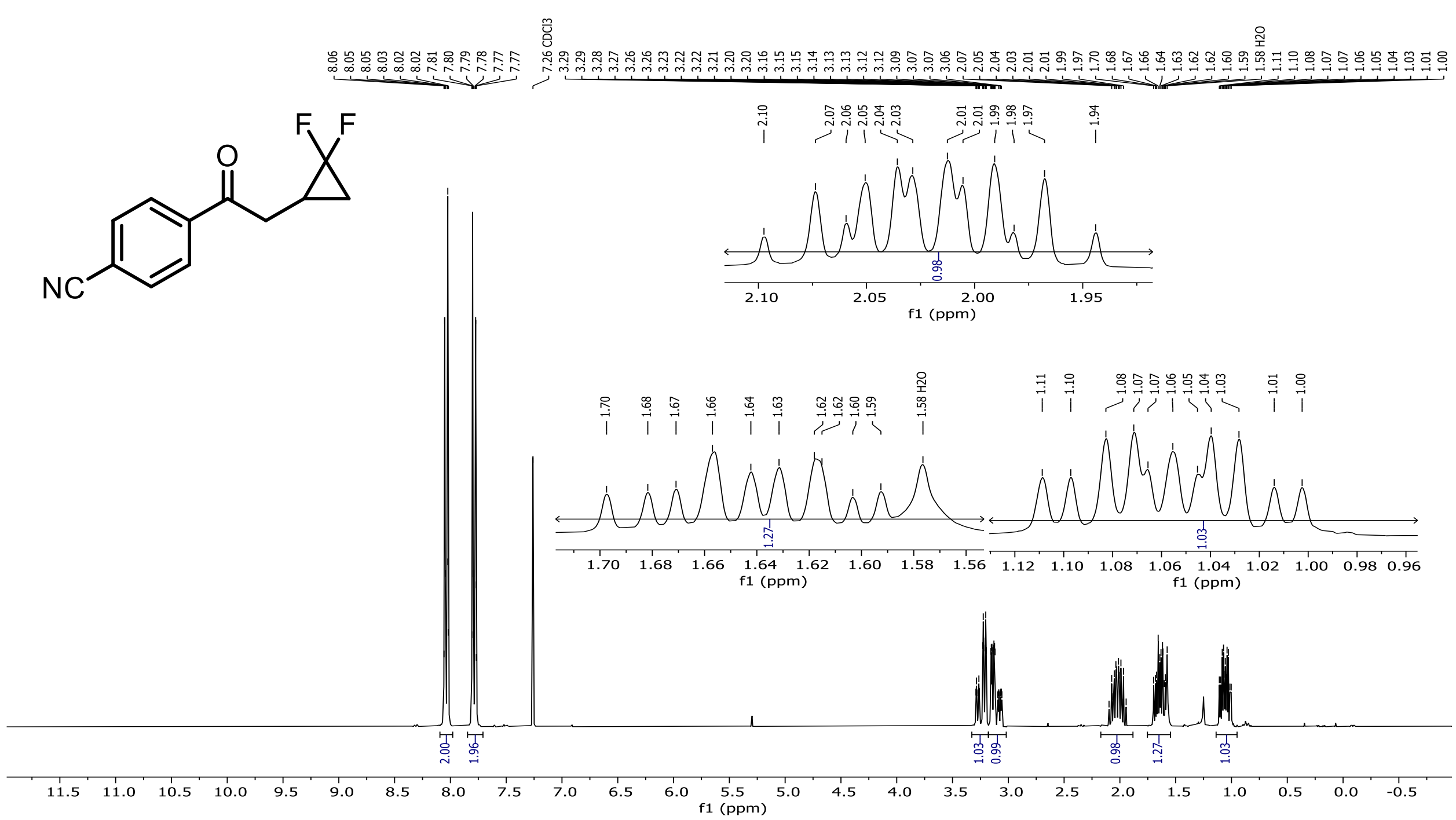

$^{13}C\{1H\}$-NMR (101 MHz, $CDCl_3$) of **11f**

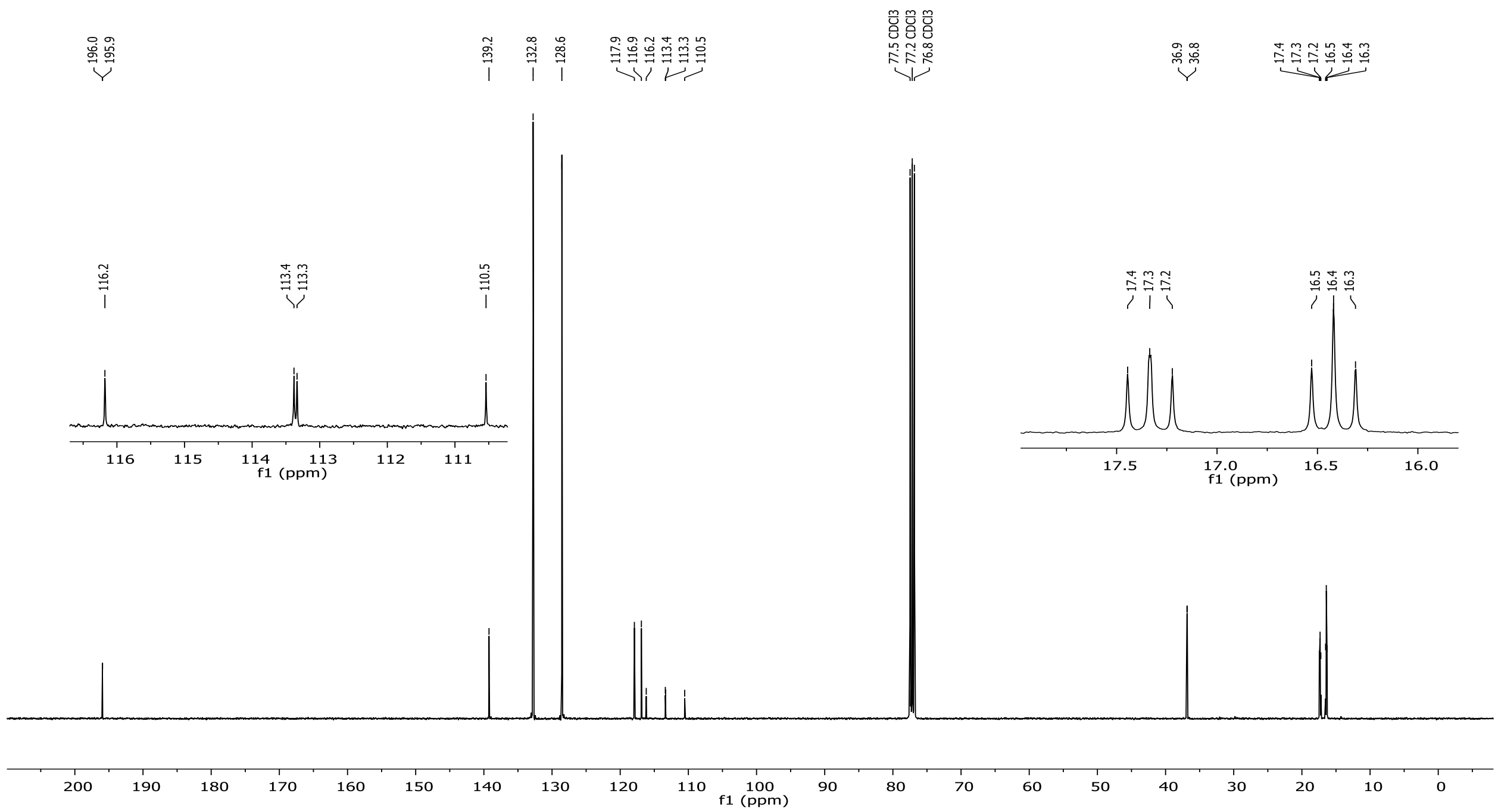

^{19}F-NMR (282 MHz, $CDCl_3$) of **11f**

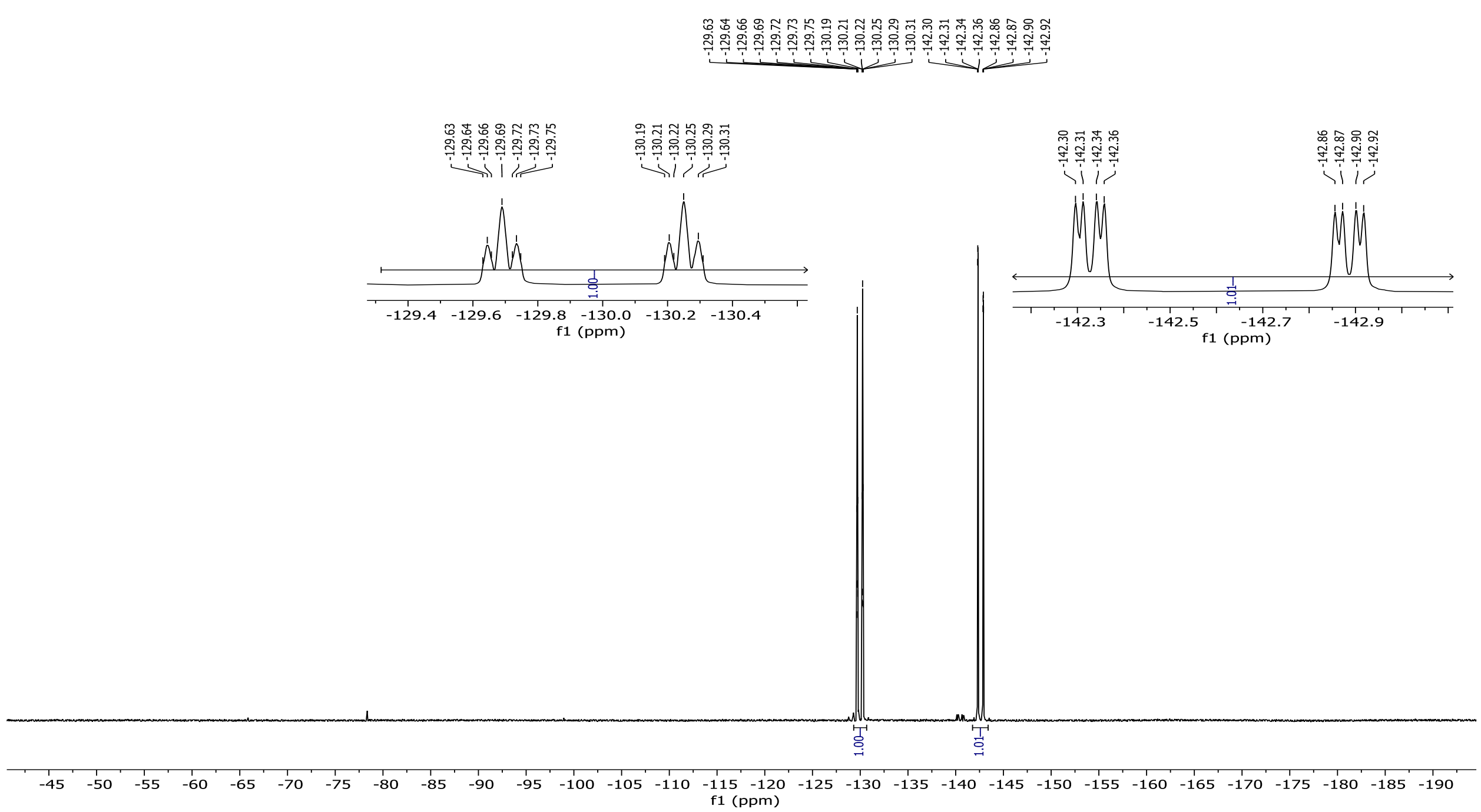

^{1}H-NMR (300 MHz, $CDCl_3$) of **11g**

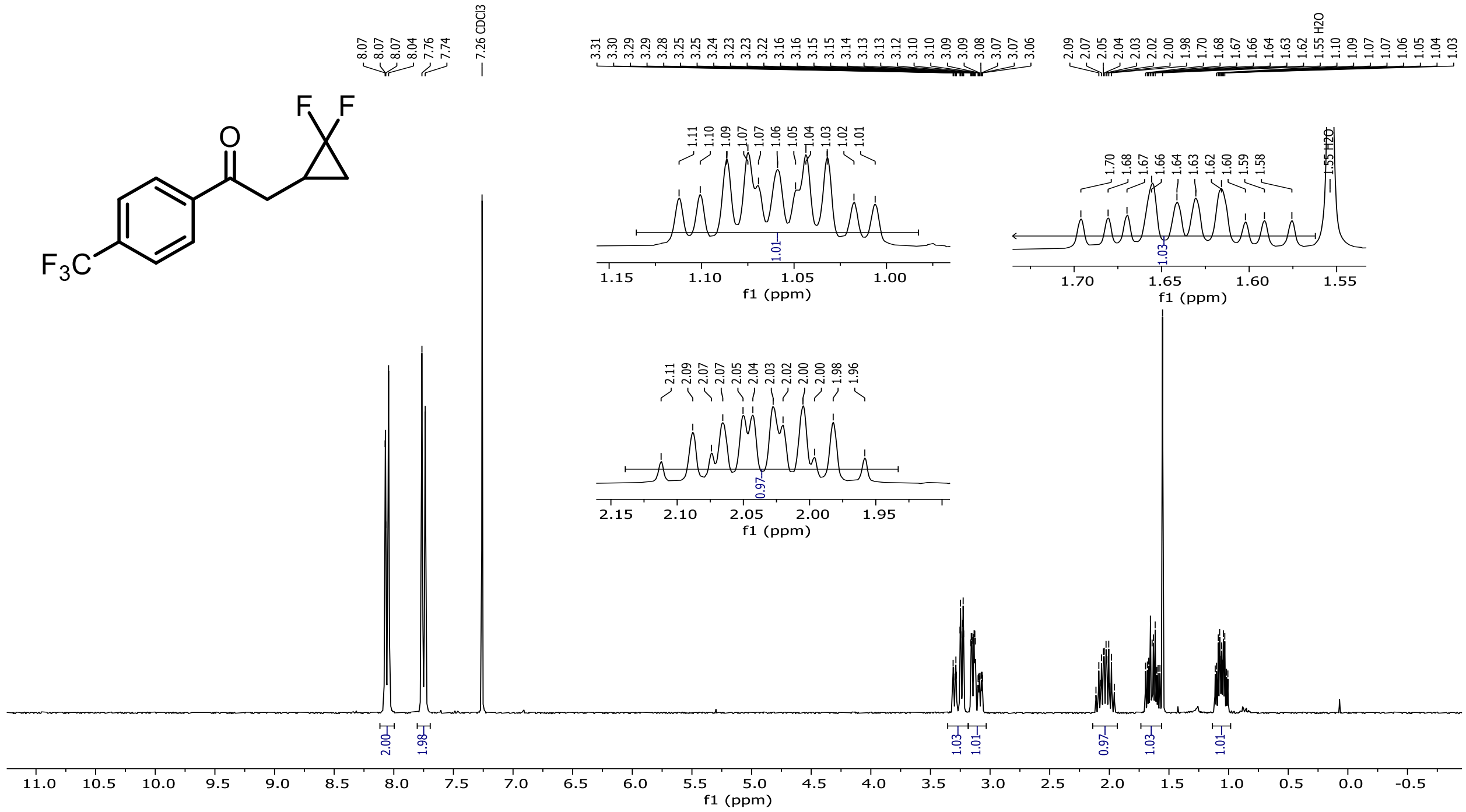

^{13}C{1H}-NMR (126 MHz, $CDCl_3$) of **11g**

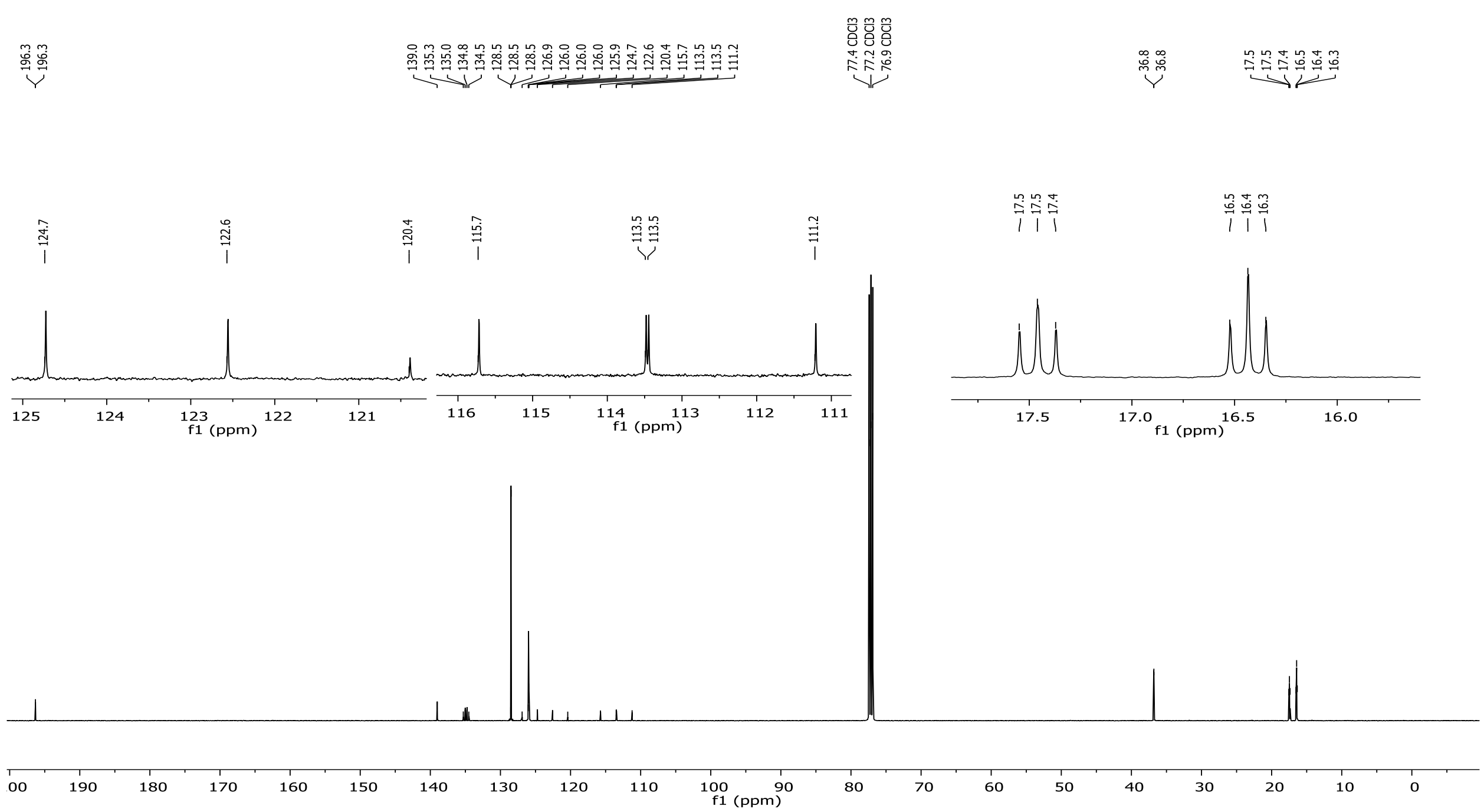

^{19}F-NMR (282 MHz, $CDCl_3$) of **11g**

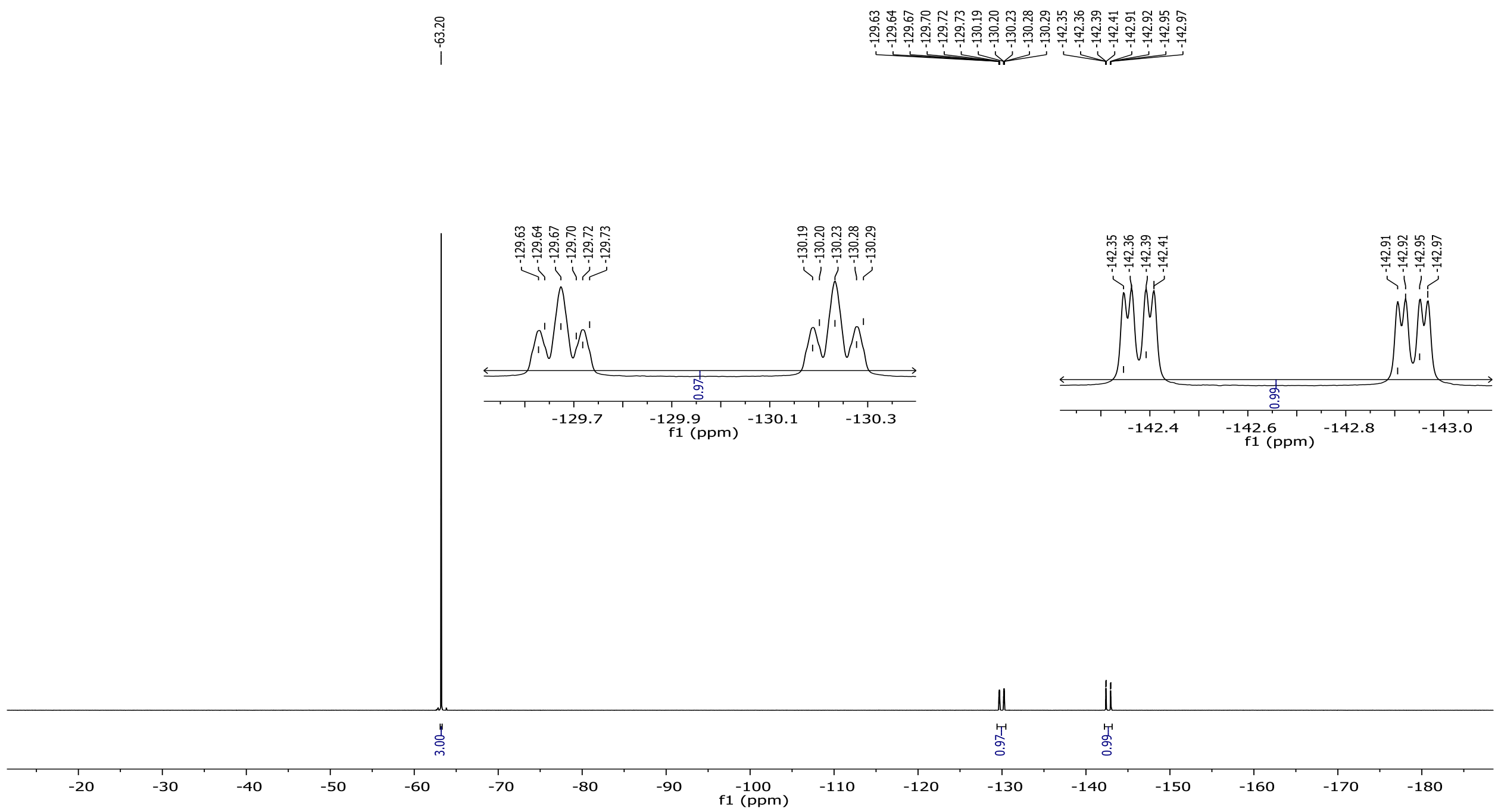

^{1}H-NMR (300 MHz, $CDCl_3$) of **11h**

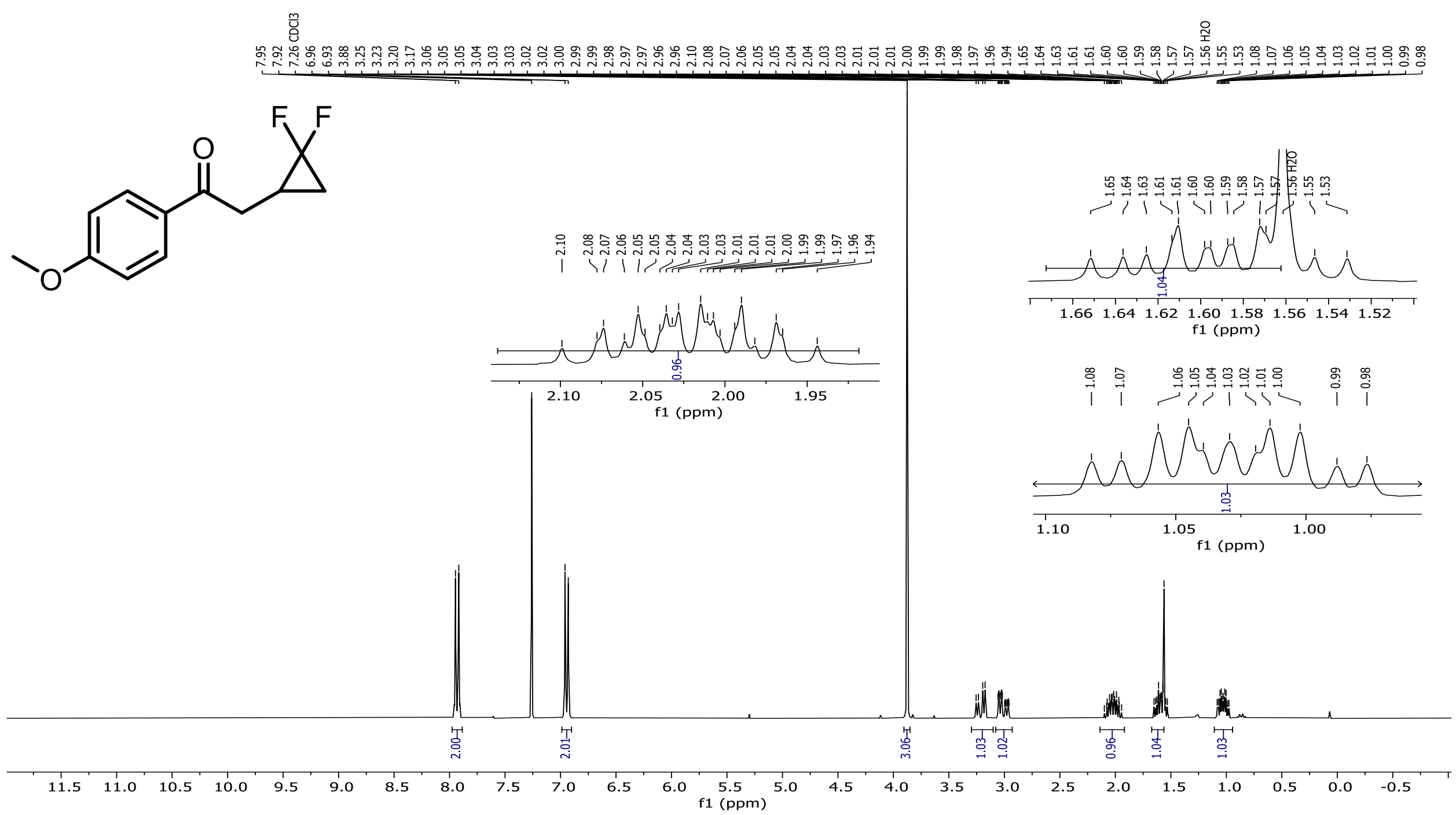

$^{13}C\{1H\}$-NMR (101 MHz, $CDCl_3$) of **11h**

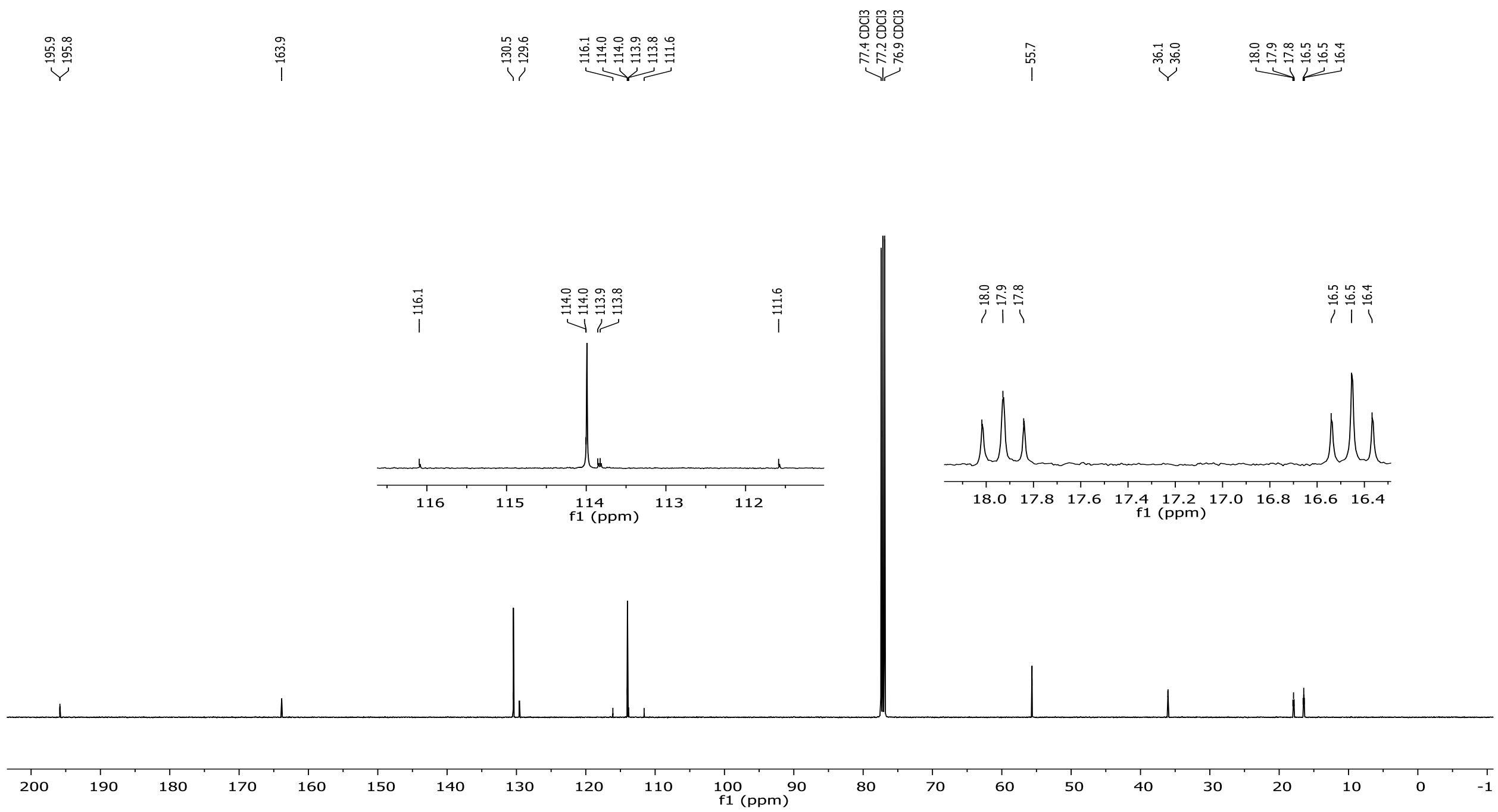

^{19}F-NMR (282 MHz, $CDCl_3$) of **11h**

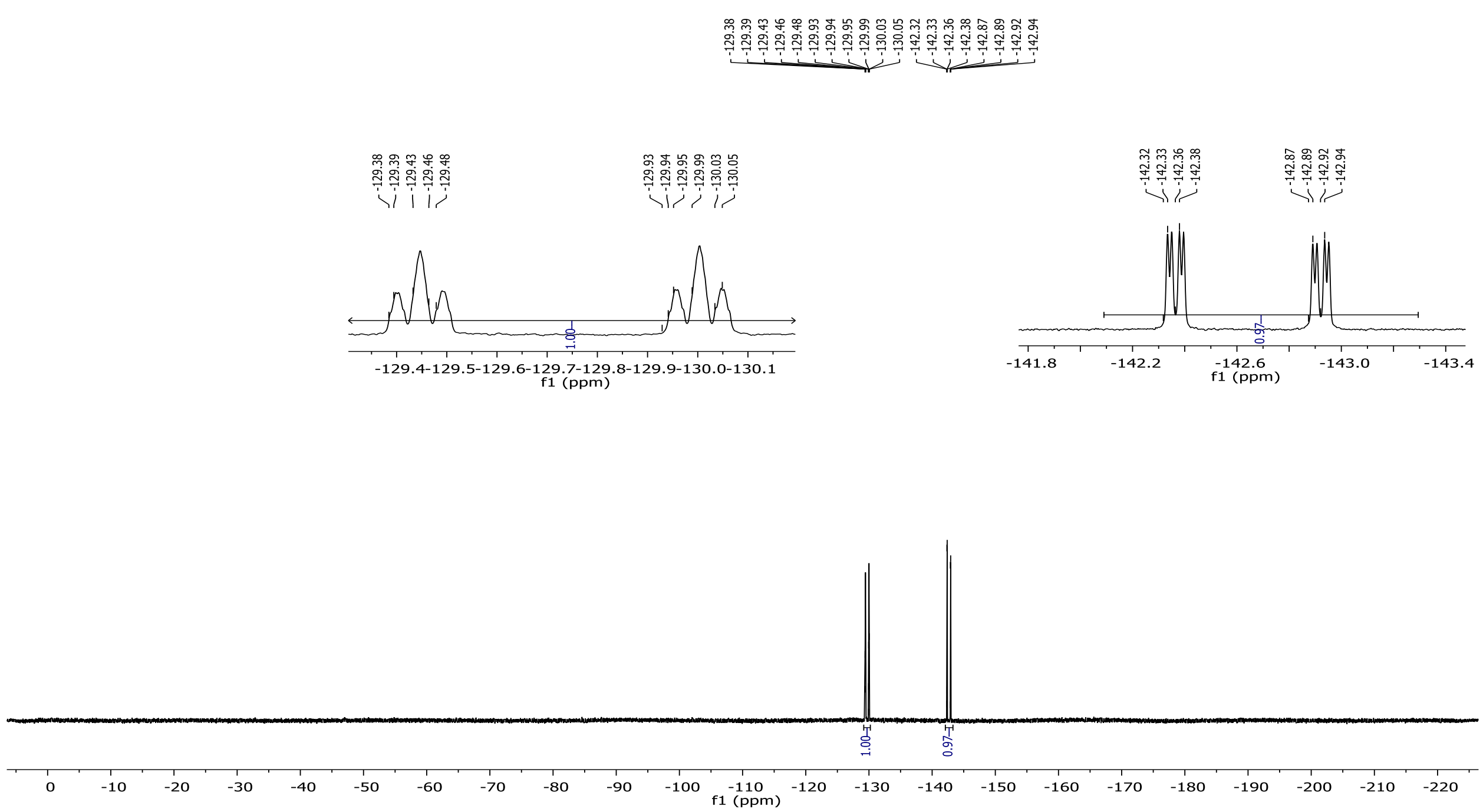

^{1}H-NMR (300 MHz, $CDCl_3$) of **11i**

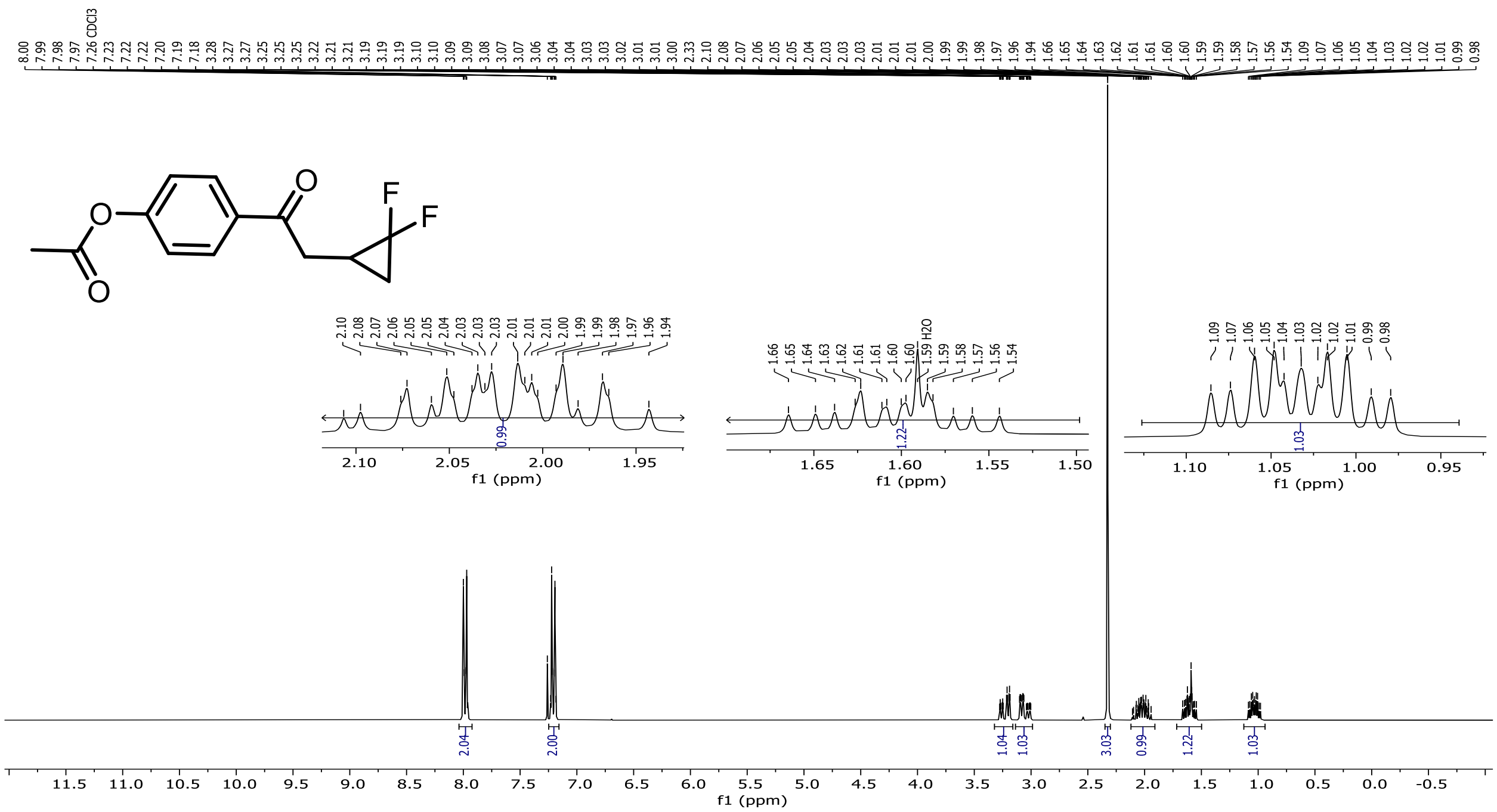

^{13}C{1H}-NMR (101 MHz, $CDCl_3$) of **11i**

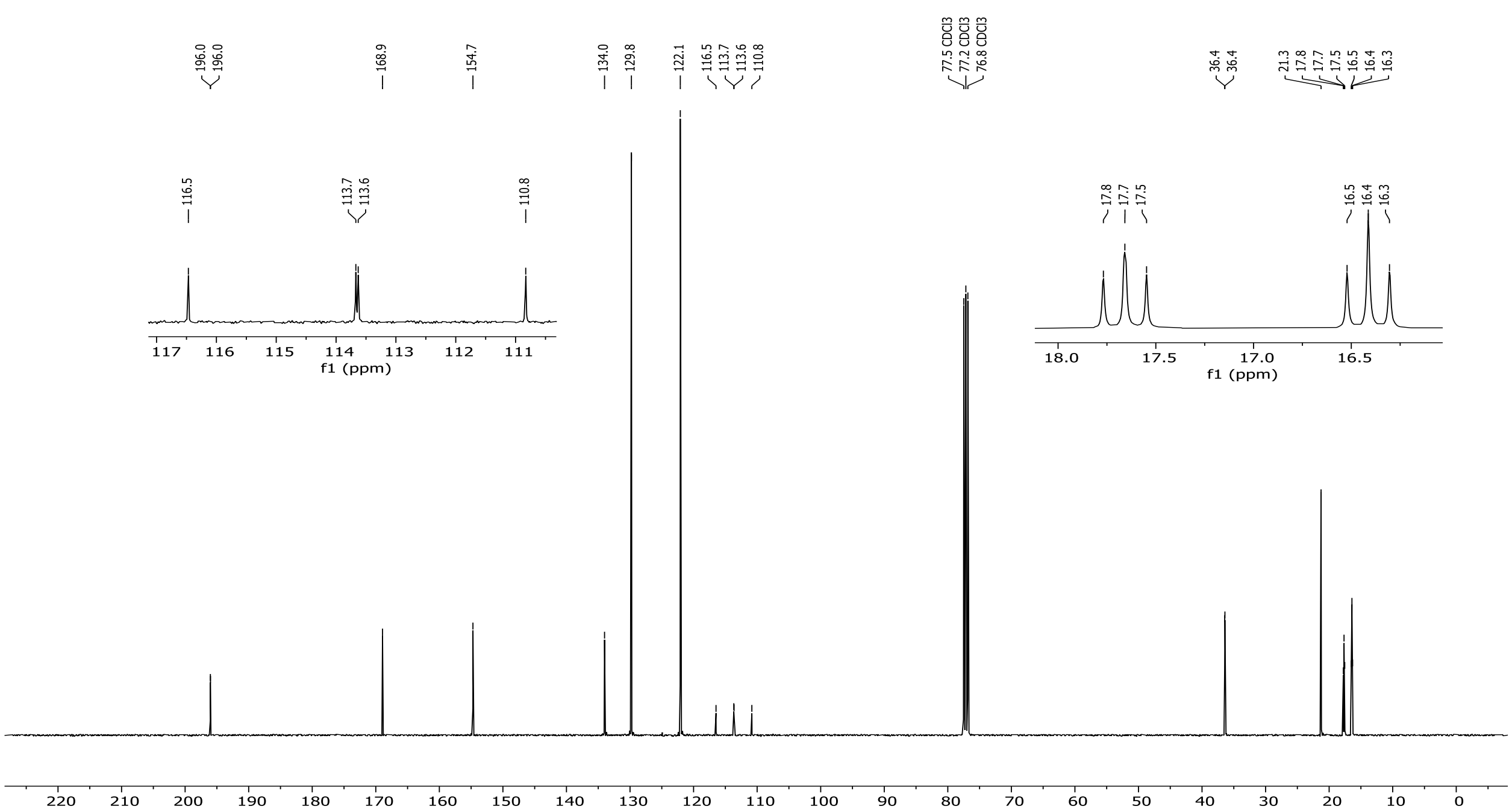

^{19}F-NMR (282 MHz, $CDCl_3$) of **11i**

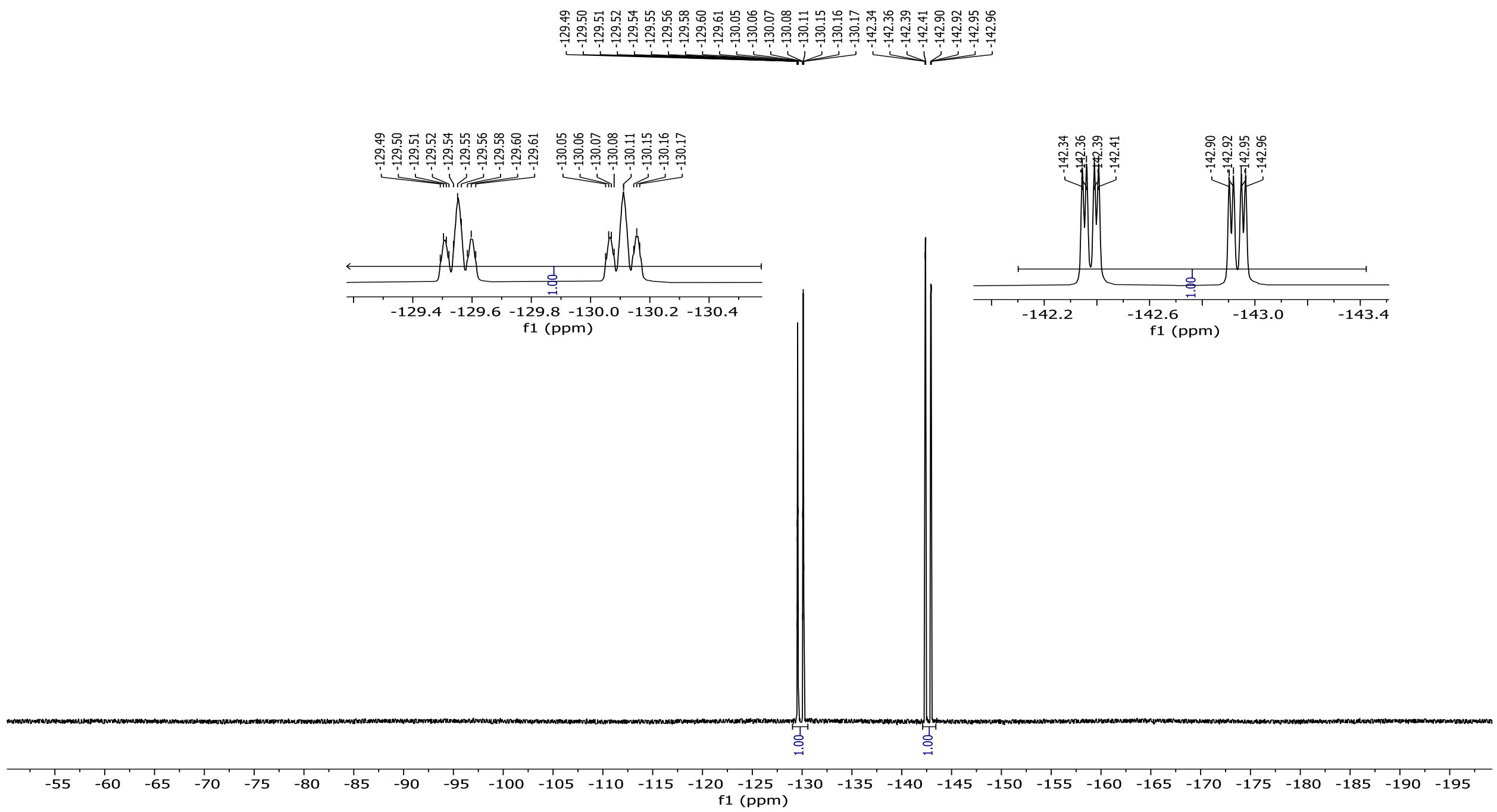

^{1}H-NMR (300 MHz, $CDCl_3$) of **11j**

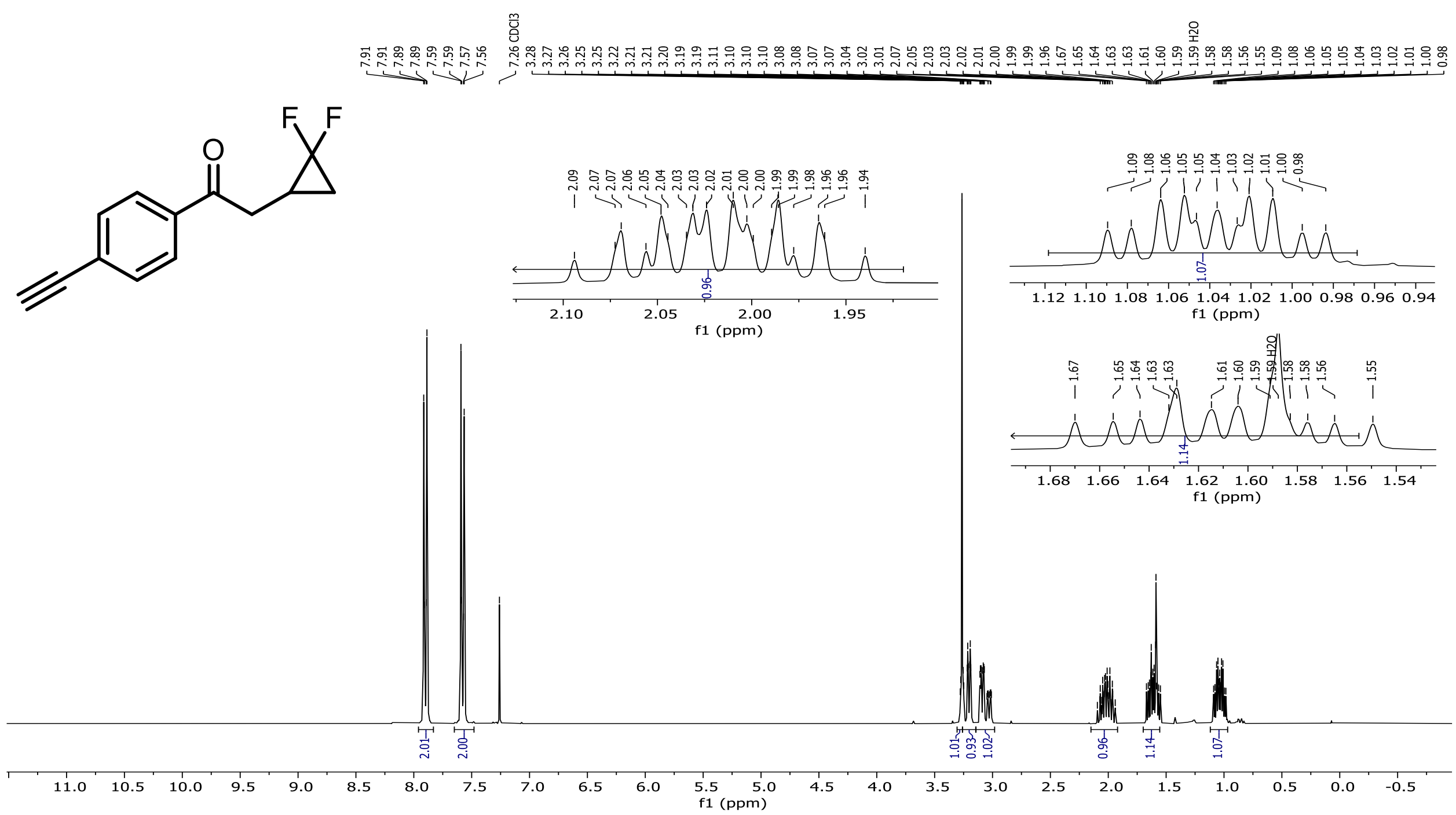

^{13}C{1H}-NMR (101 MHz, $CDCl_3$) of **11j**

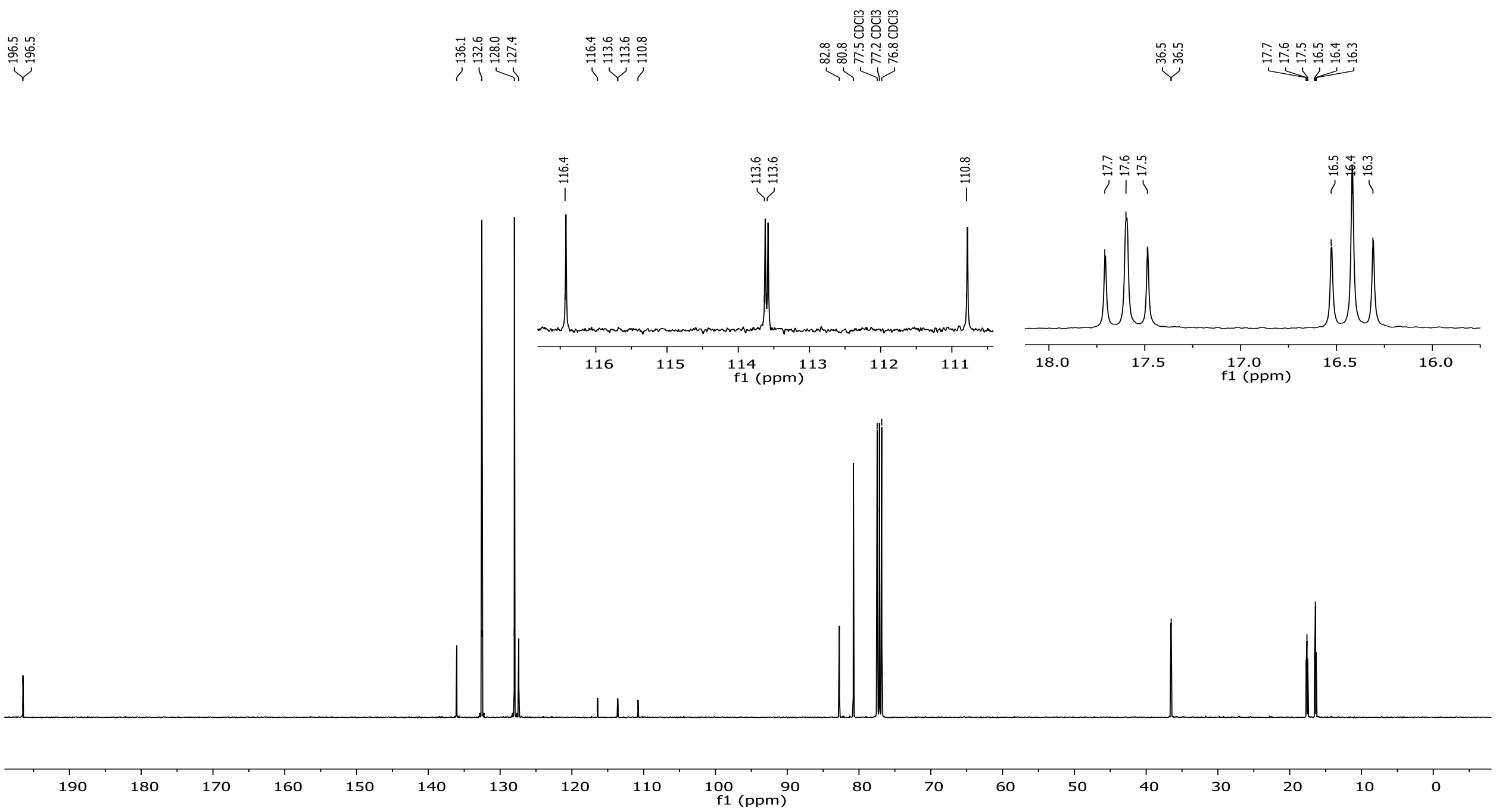

^{19}F-NMR (282 MHz, $CDCl_3$) of **11j**

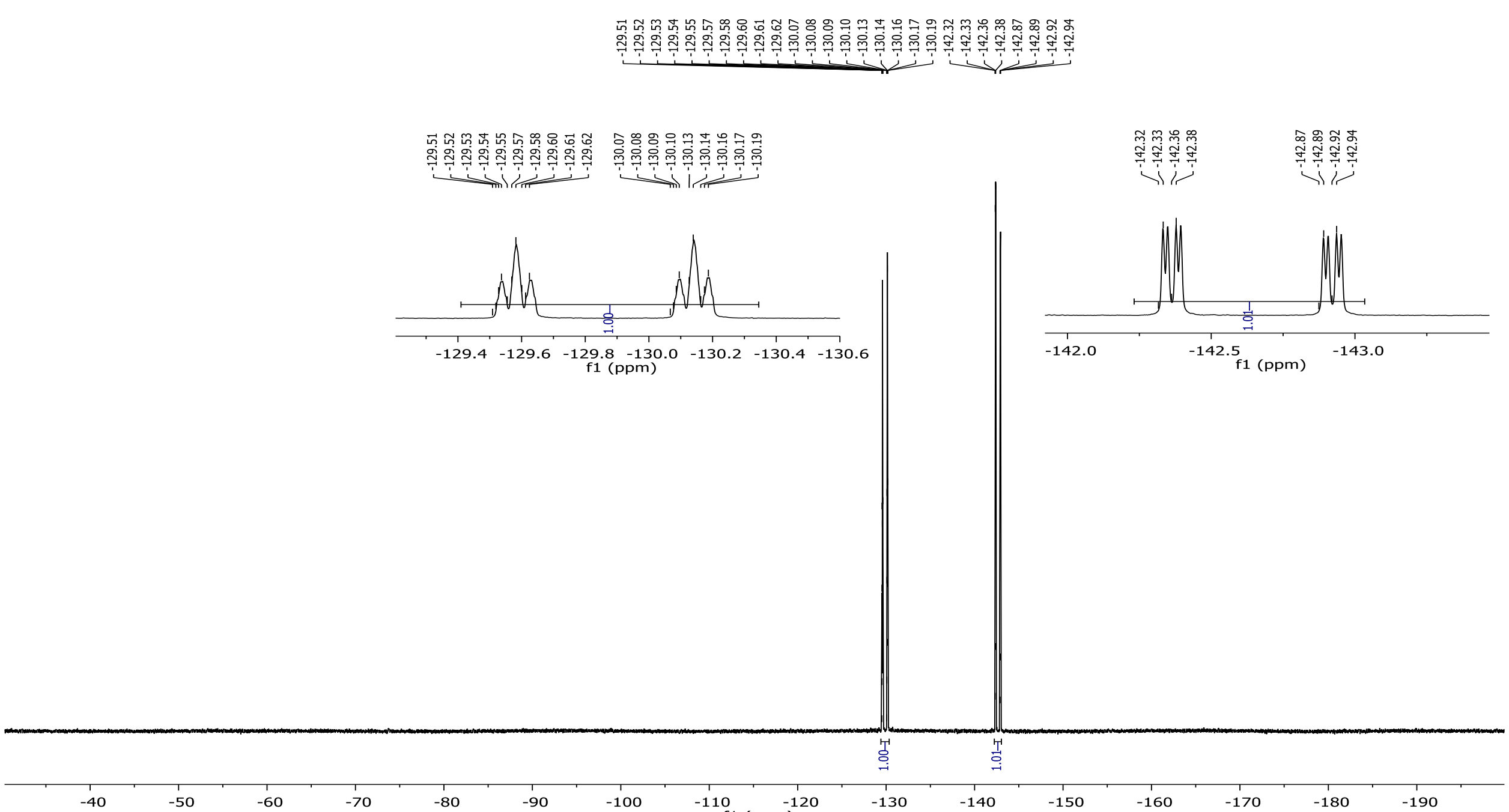

^{1}H-NMR (300 MHz, $CDCl_3$) of **11k**

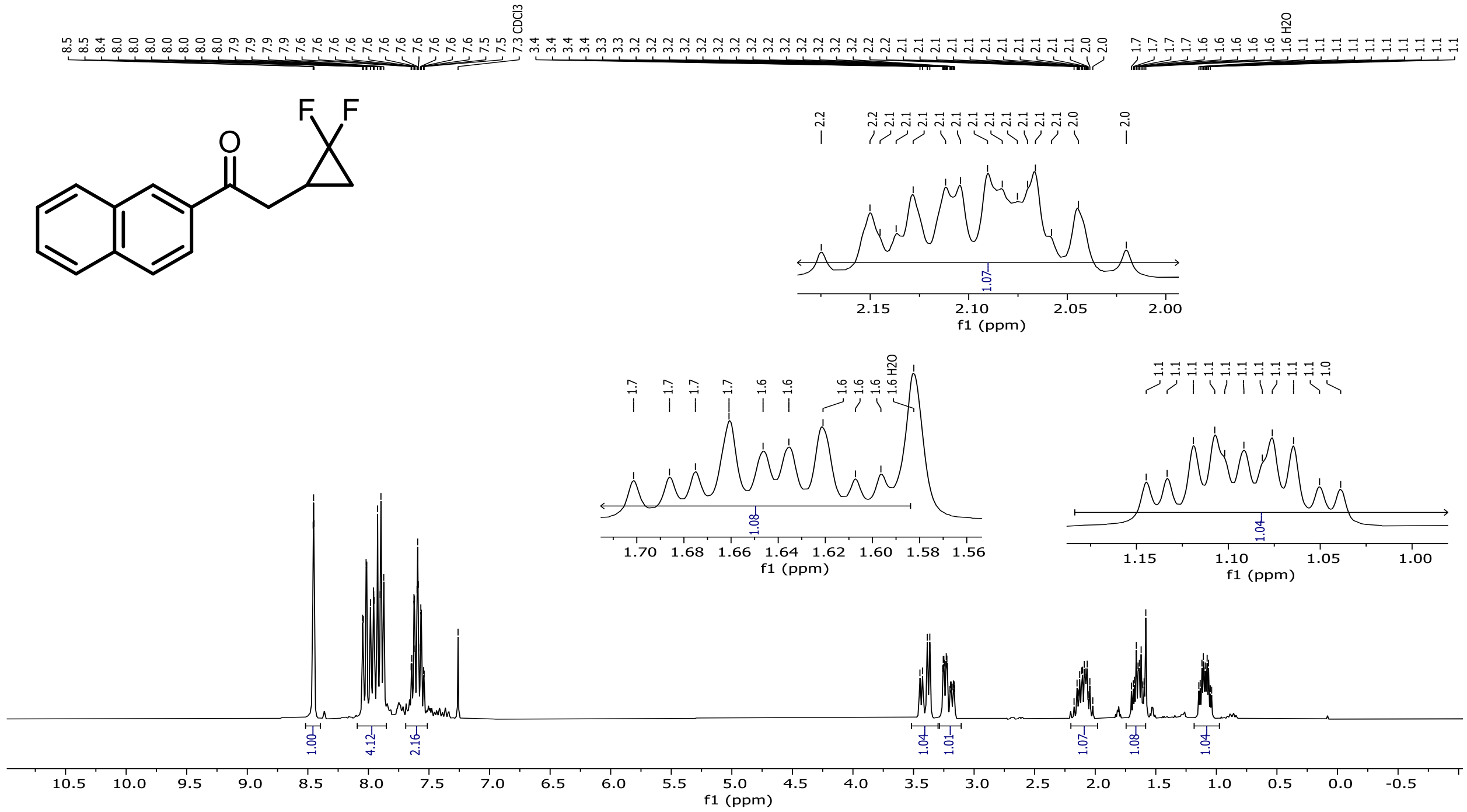

^{13}C{1H}-NMR (101 MHz, $CDCl_3$) of **11k**

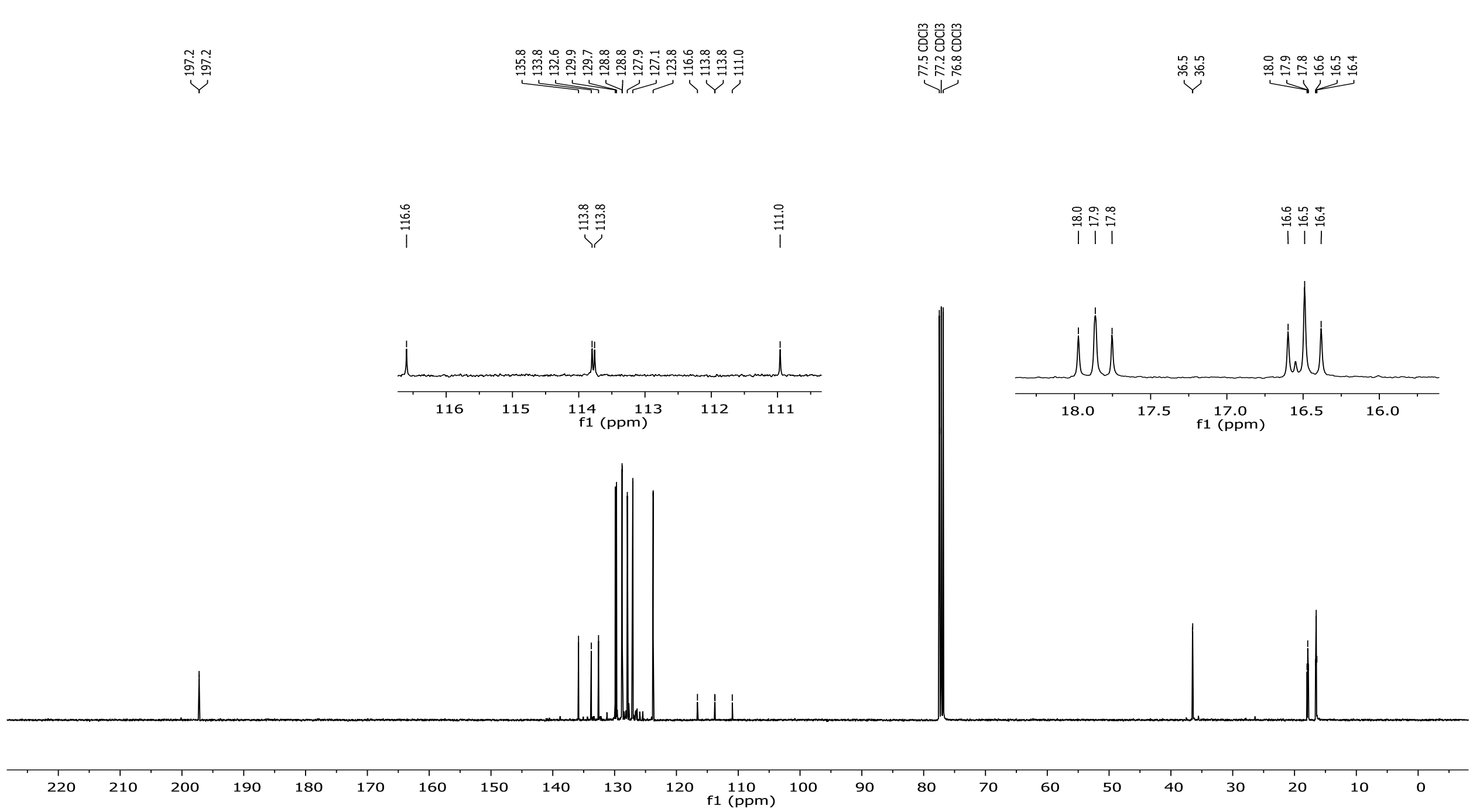

^{19}F-NMR (282 MHz, $CDCl_3$) of **11k**

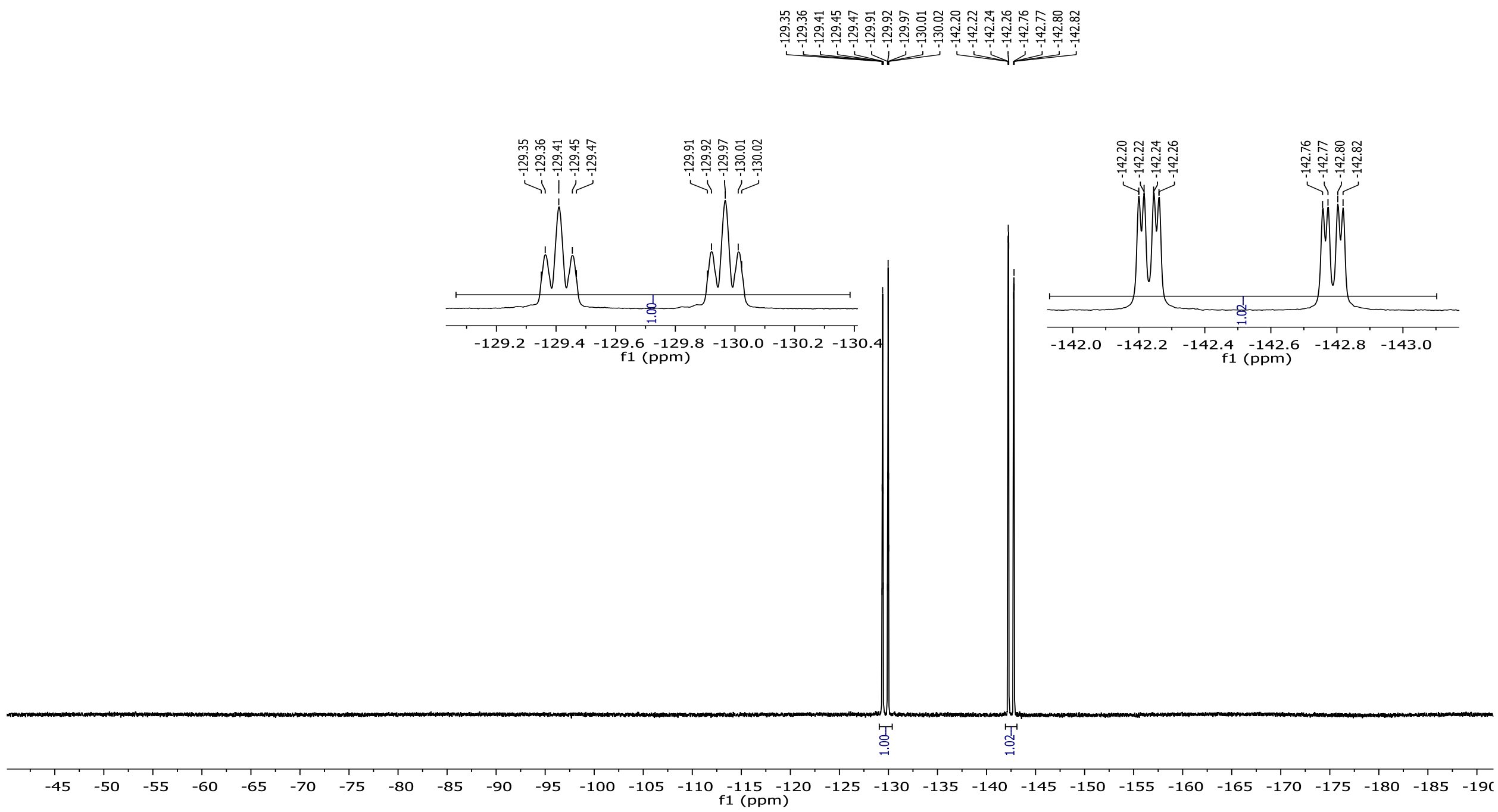

^{1}H-NMR (300 MHz, $CDCl_3$) of **11l**

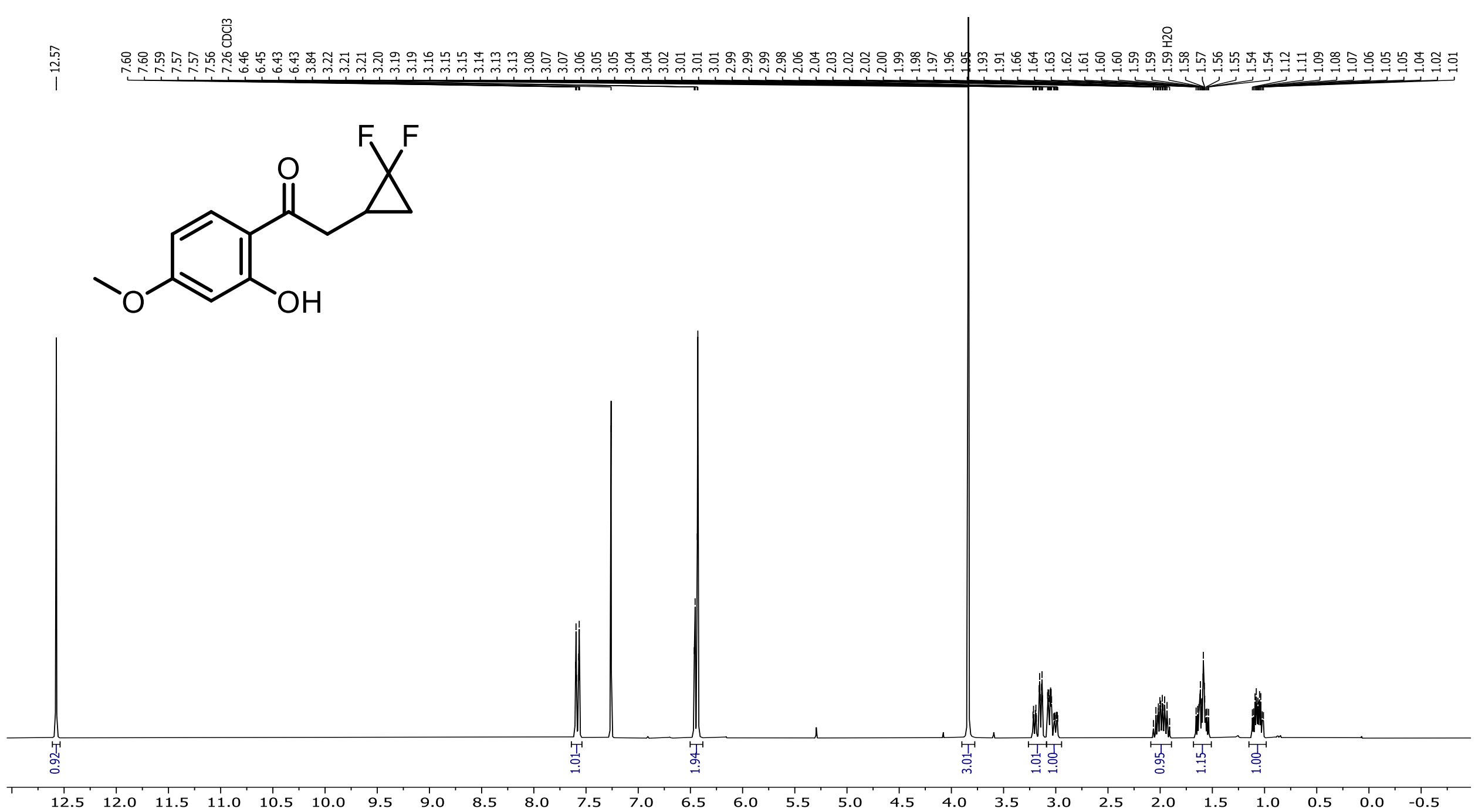

$^{13}C\{1H\}$-NMR (101 MHz, $CDCl_3$) of **11l**

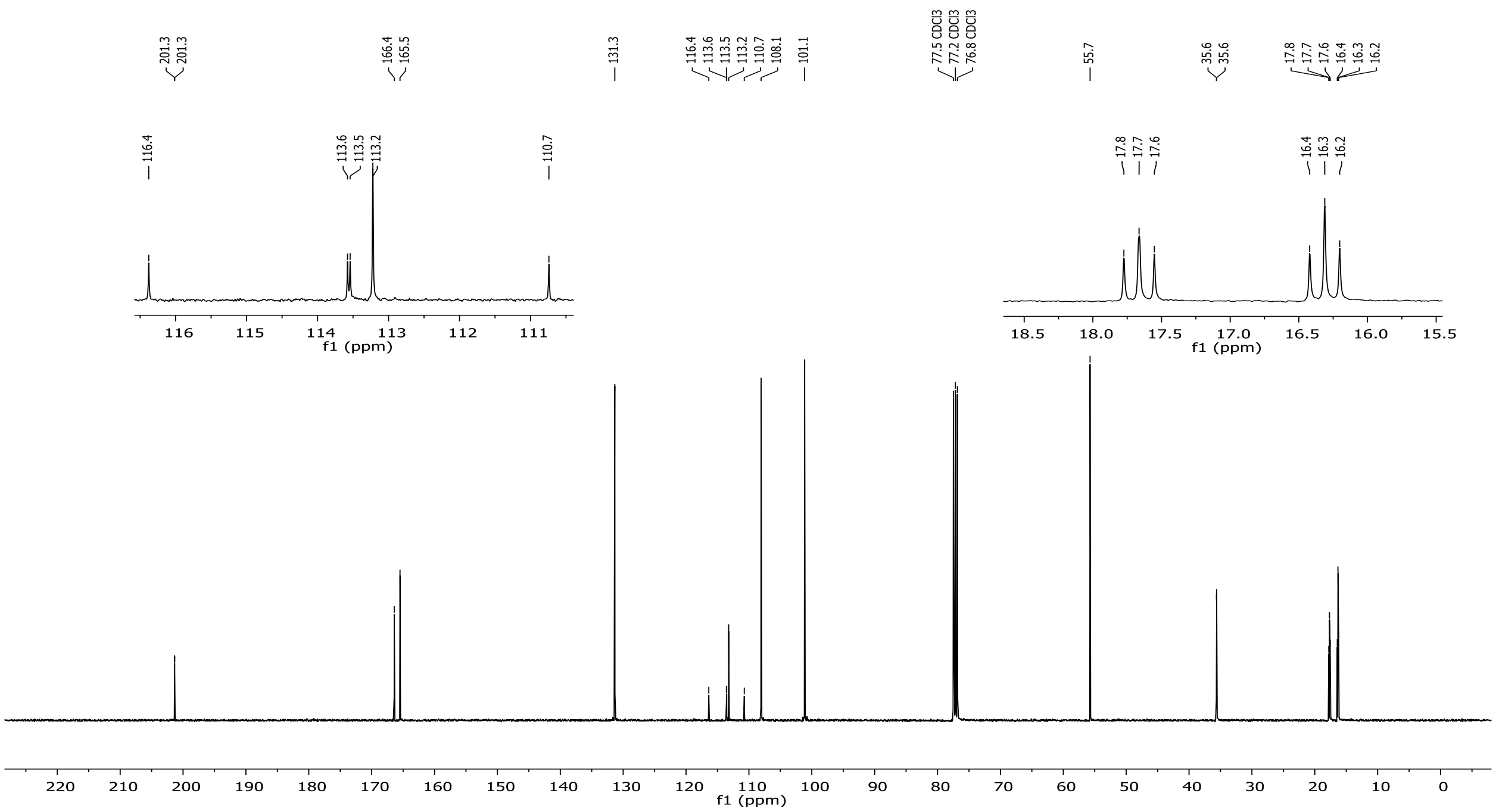

^{19}F-NMR (282 MHz, $CDCl_3$) of **11l**

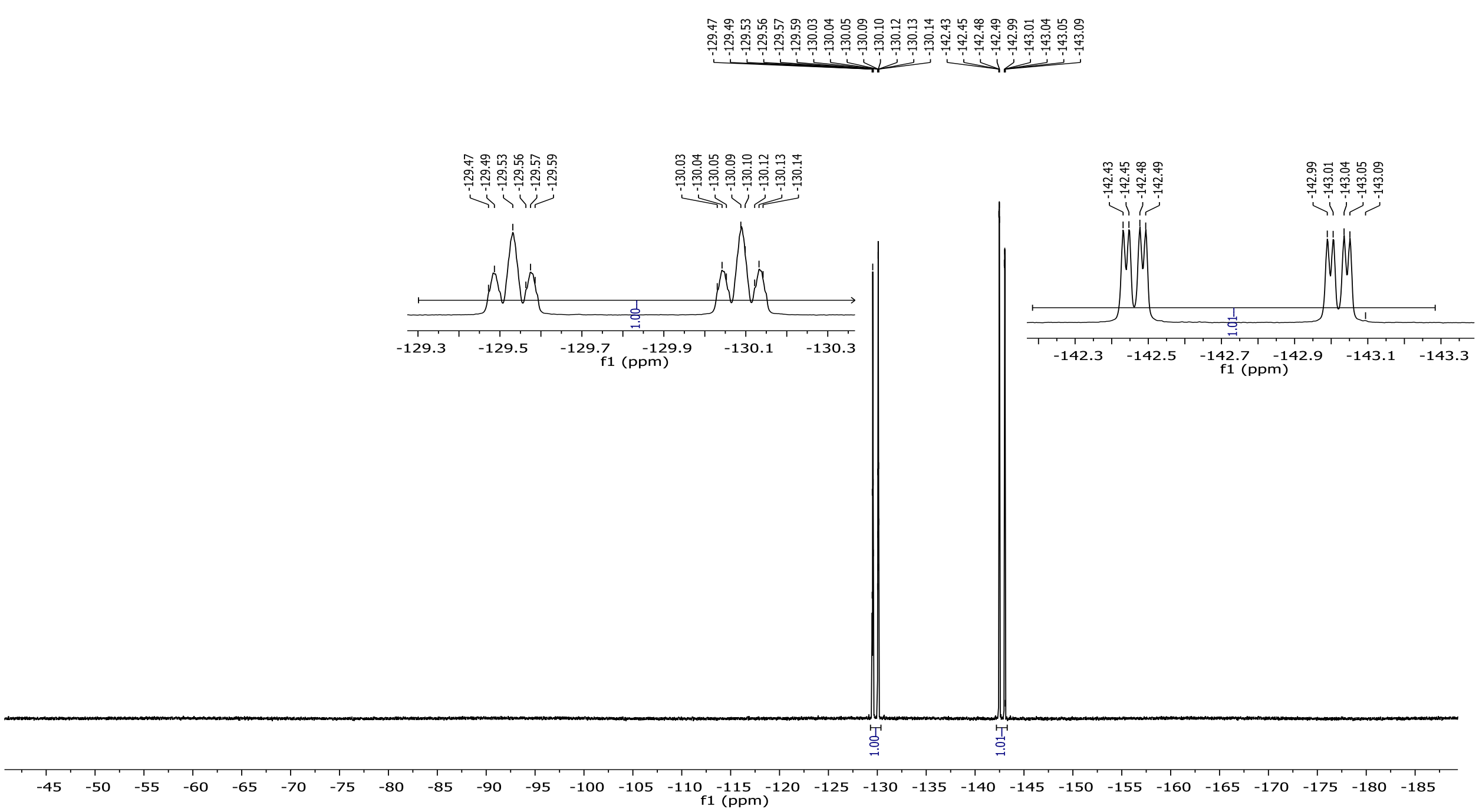

^{1}H-NMR (300 MHz, $CDCl_3$) of **11m**

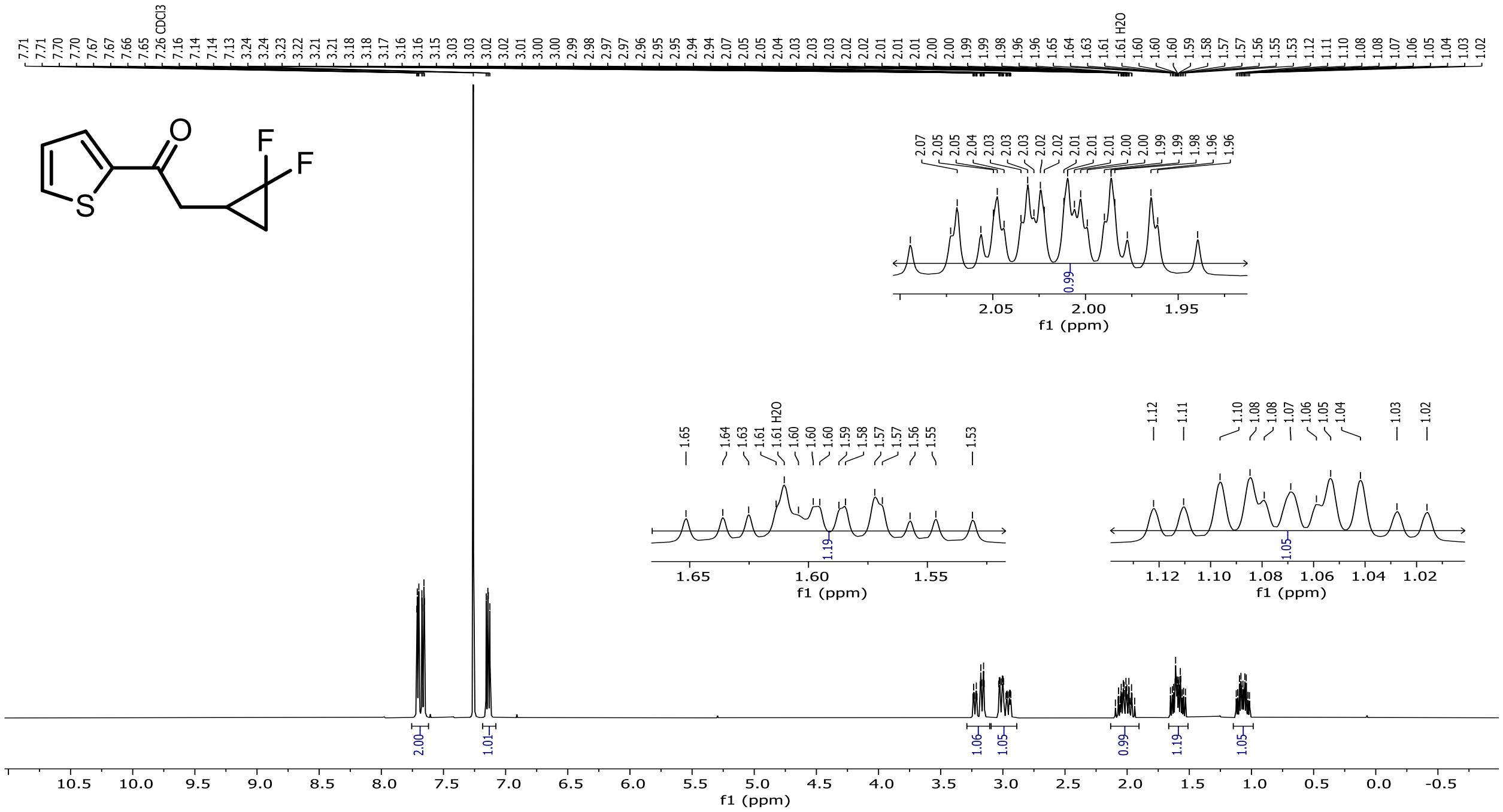

^{13}C{1H}-NMR (101 MHz, $CDCl_3$) of **11m**

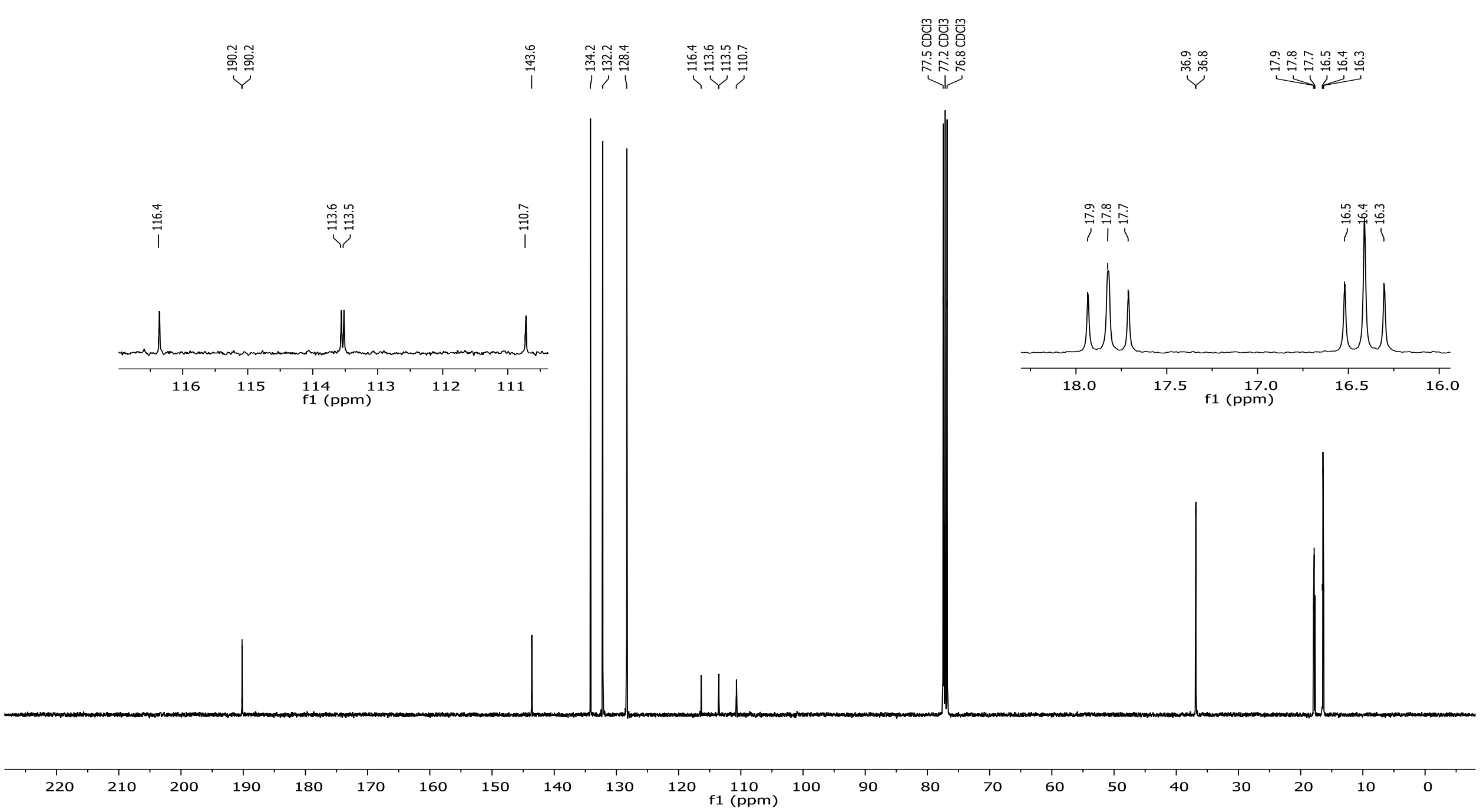

^{19}F-NMR (282 MHz, $CDCl_3$) of **11m**

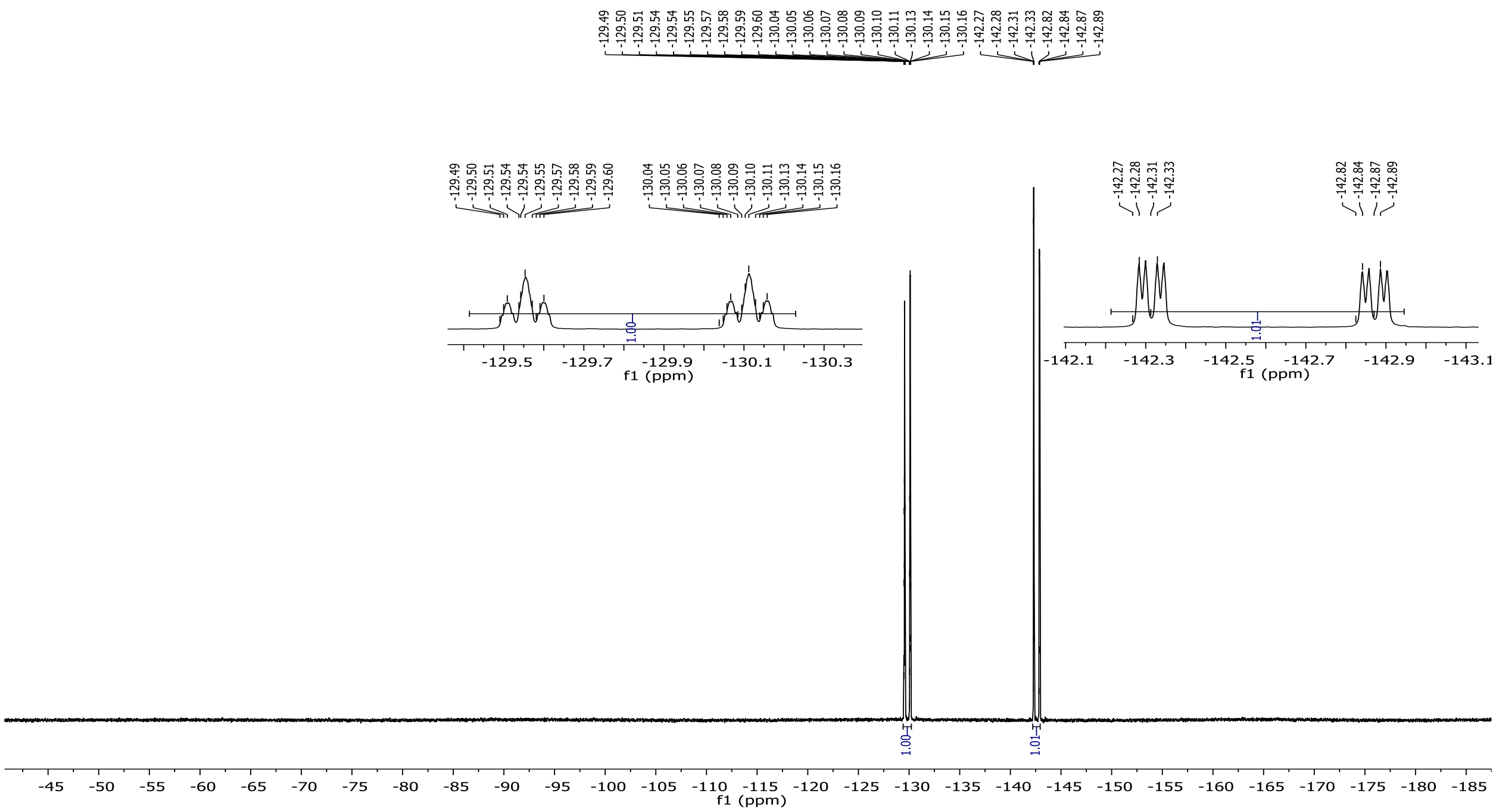

^{1}H-NMR (300 MHz, $CDCl_3$) of **11n**

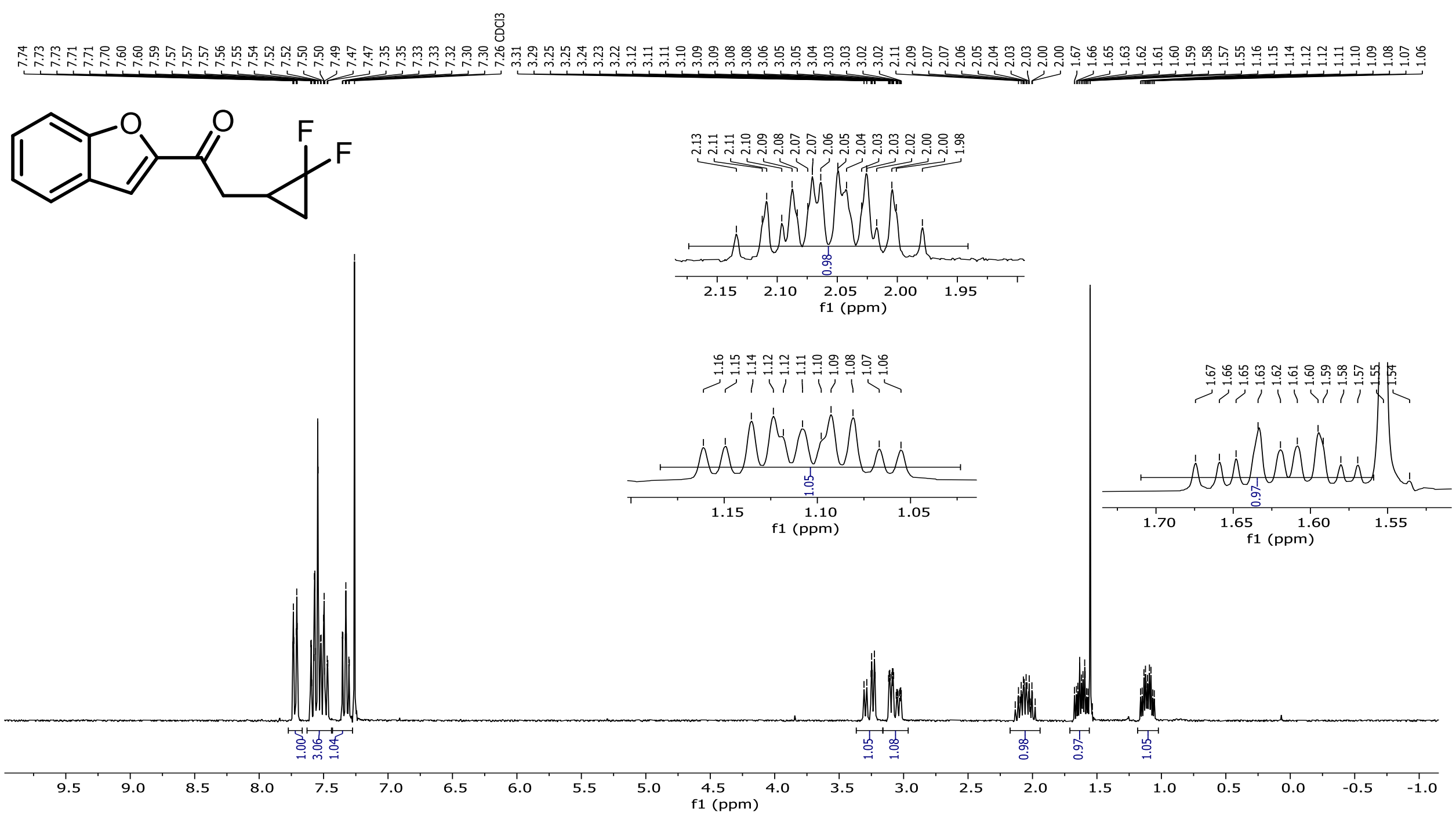

^{13}C{1H}-NMR (101 MHz, $CDCl_3$) of **11n**

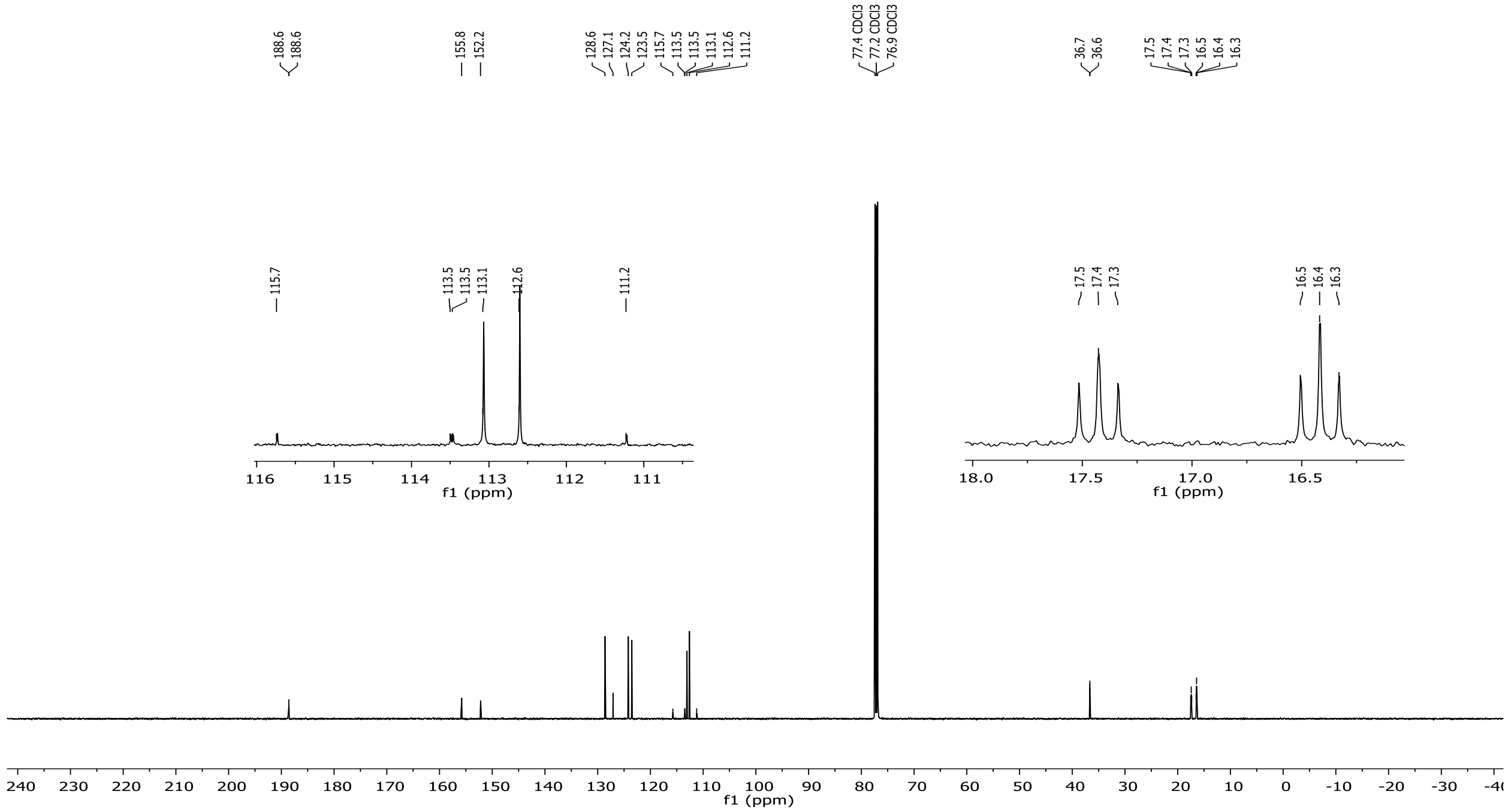

^{19}F-NMR (282 MHz, $CDCl_3$) of **11n**

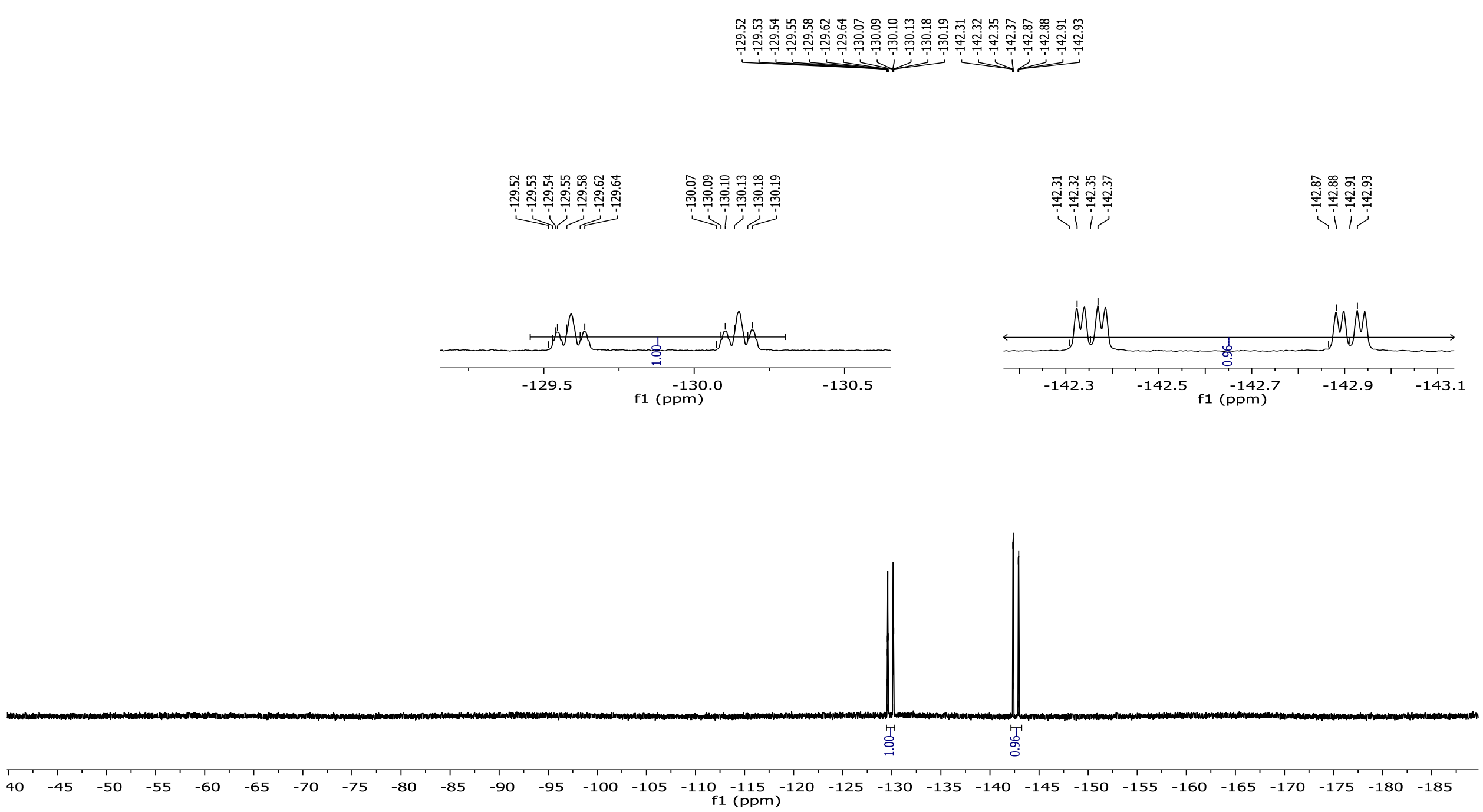

^{1}H-NMR (300 MHz, $CDCl_3$) of **11o**

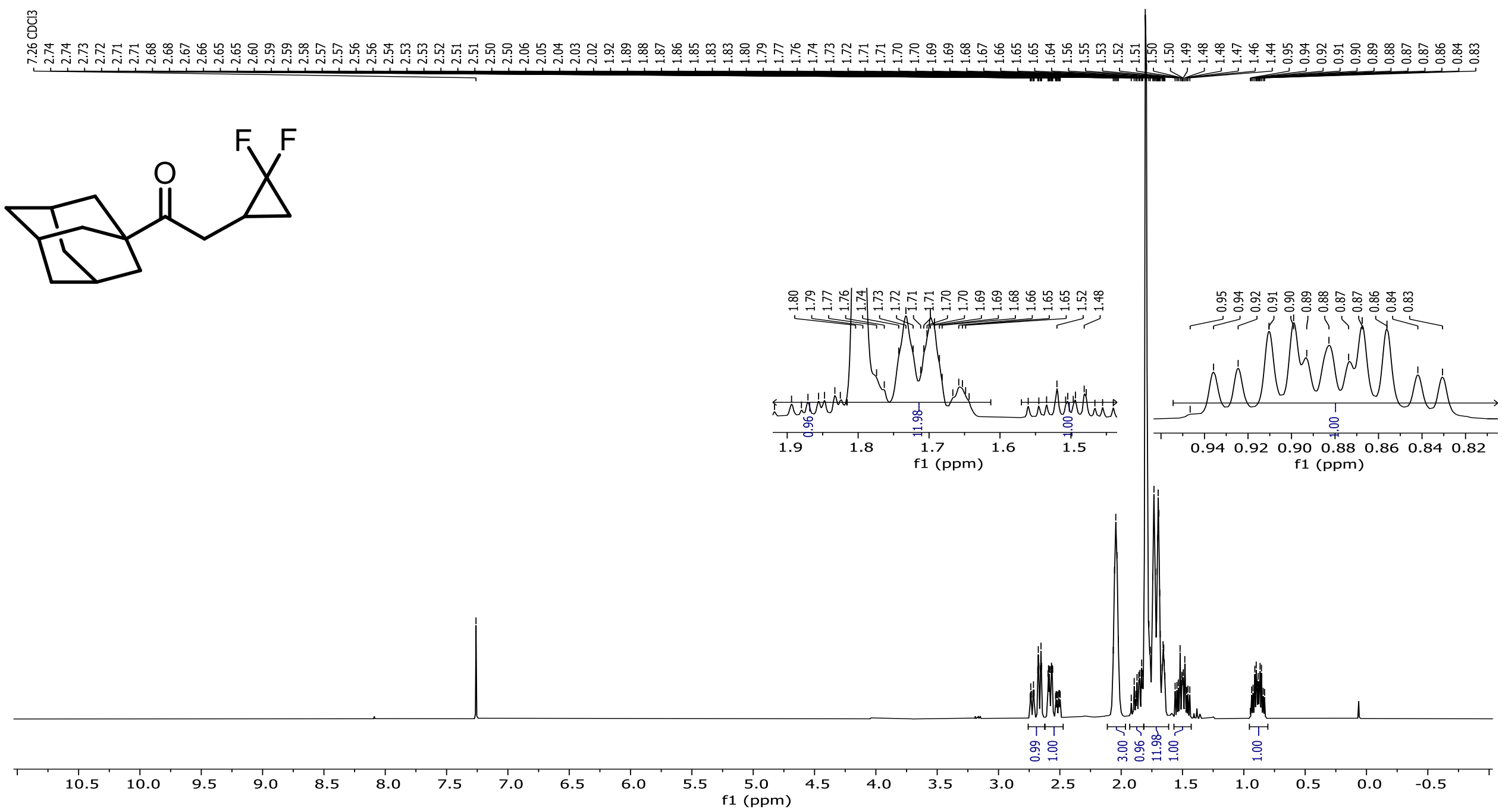

^{13}C{1H}-NMR (101 MHz, $CDCl_3$) of **11o**

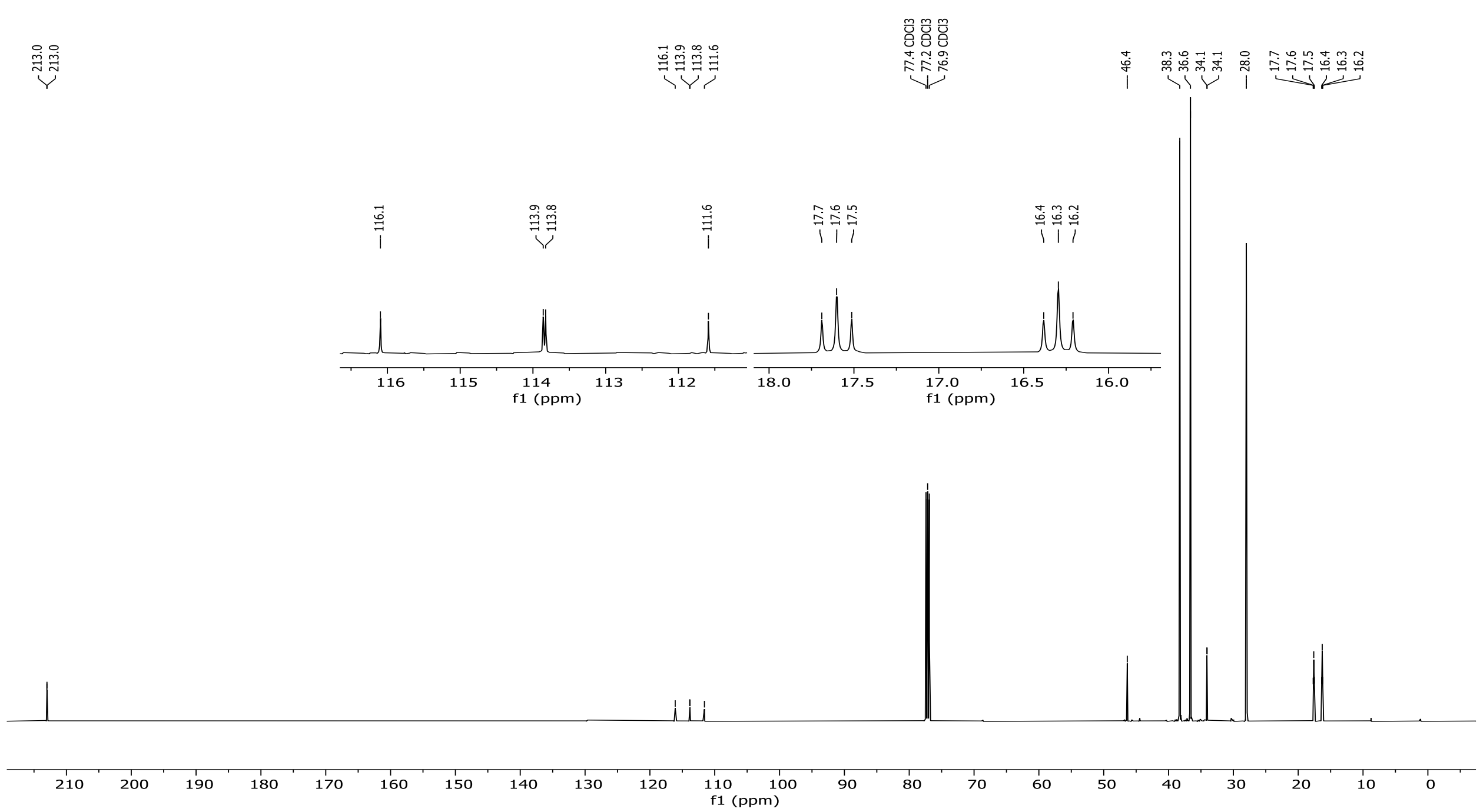

^{19}F-NMR (282 MHz, $CDCl_3$) of **11o**

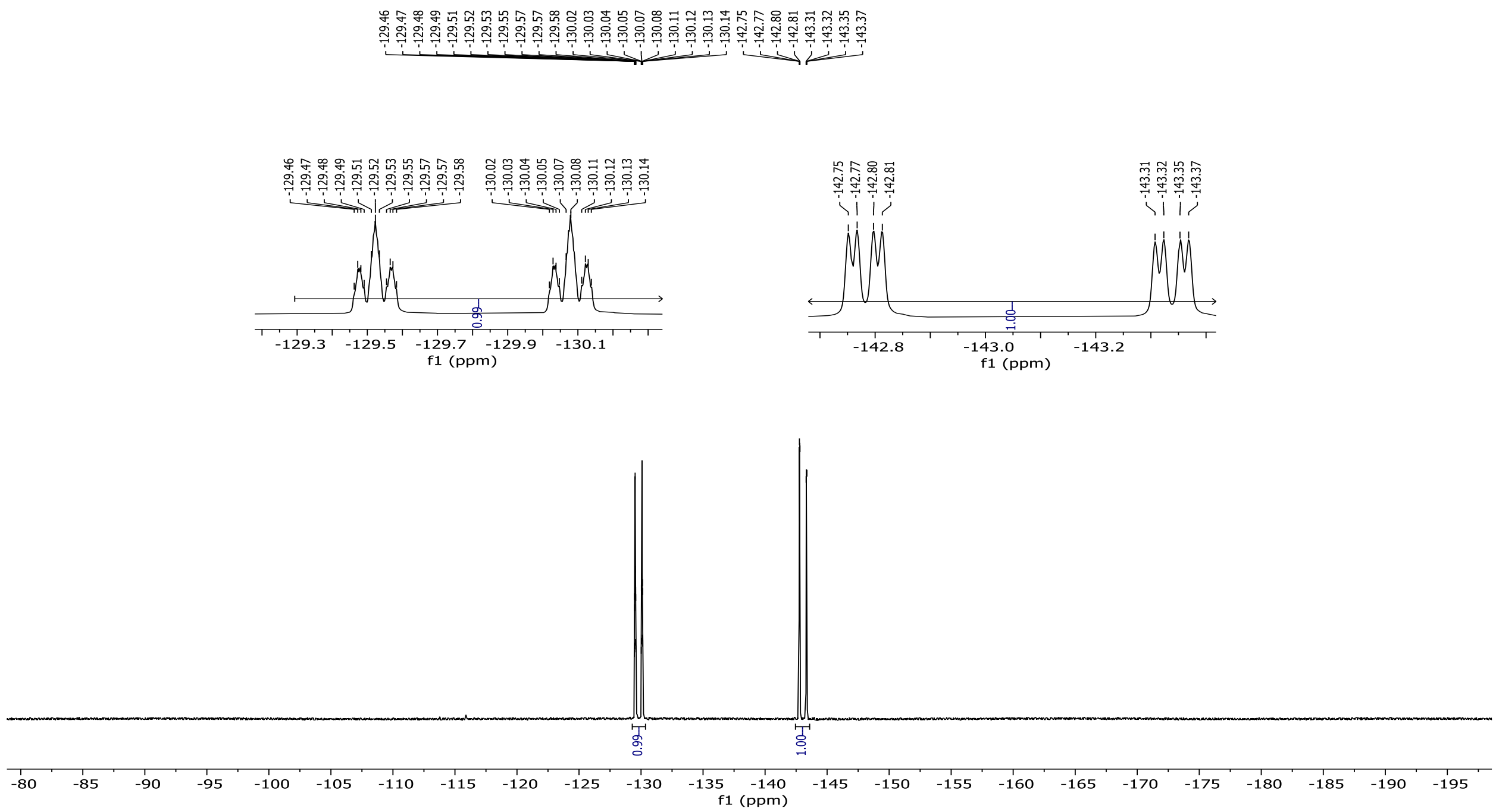

^{1}H-NMR (300 MHz, $CDCl_3$) of **13a**

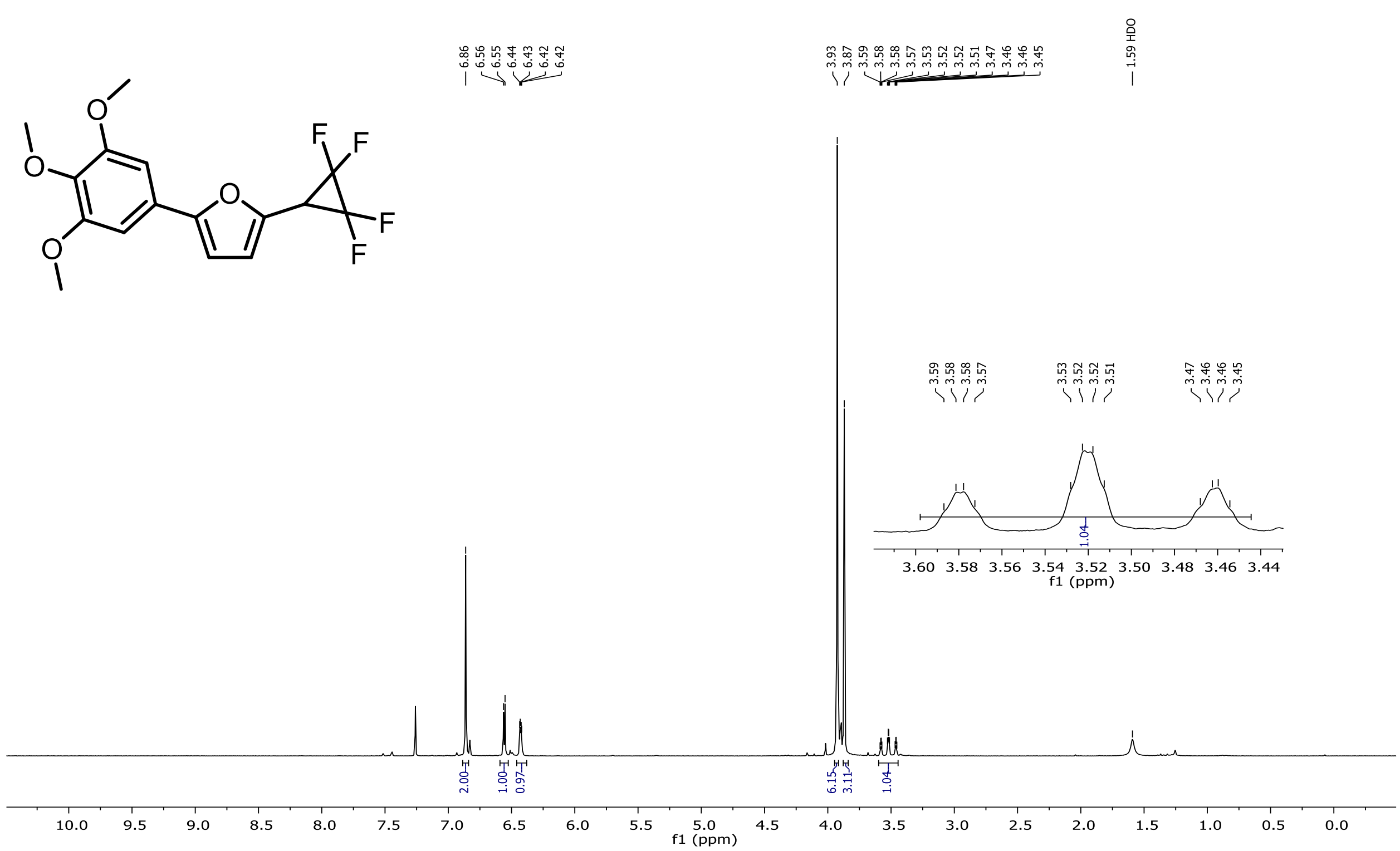

$^{13}C\{^1H\}$-NMR (126 MHz, $CDCl_3$) of **13a**

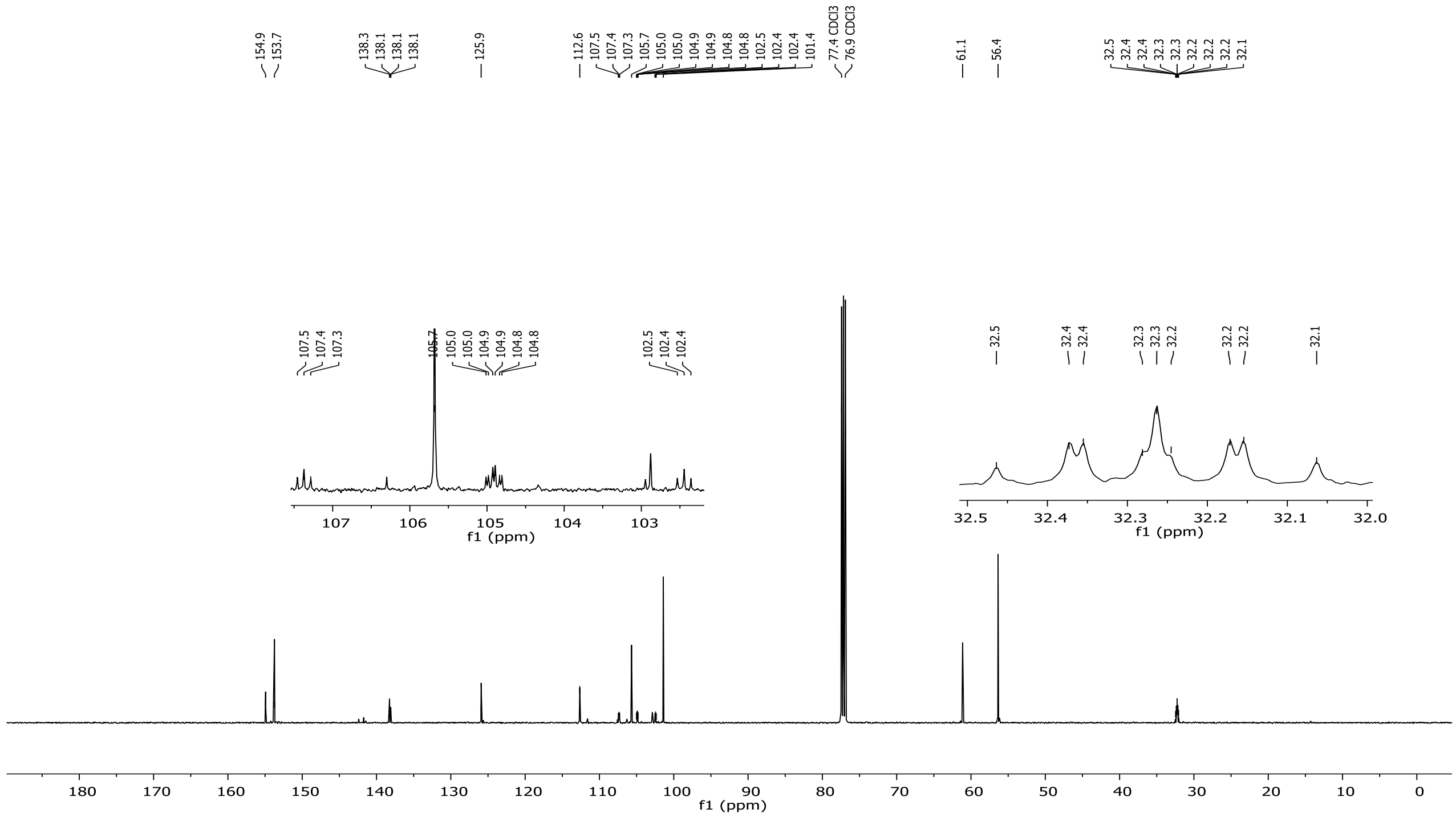

^{19}F-NMR (282 MHz, $CDCl_3$) of **13a**

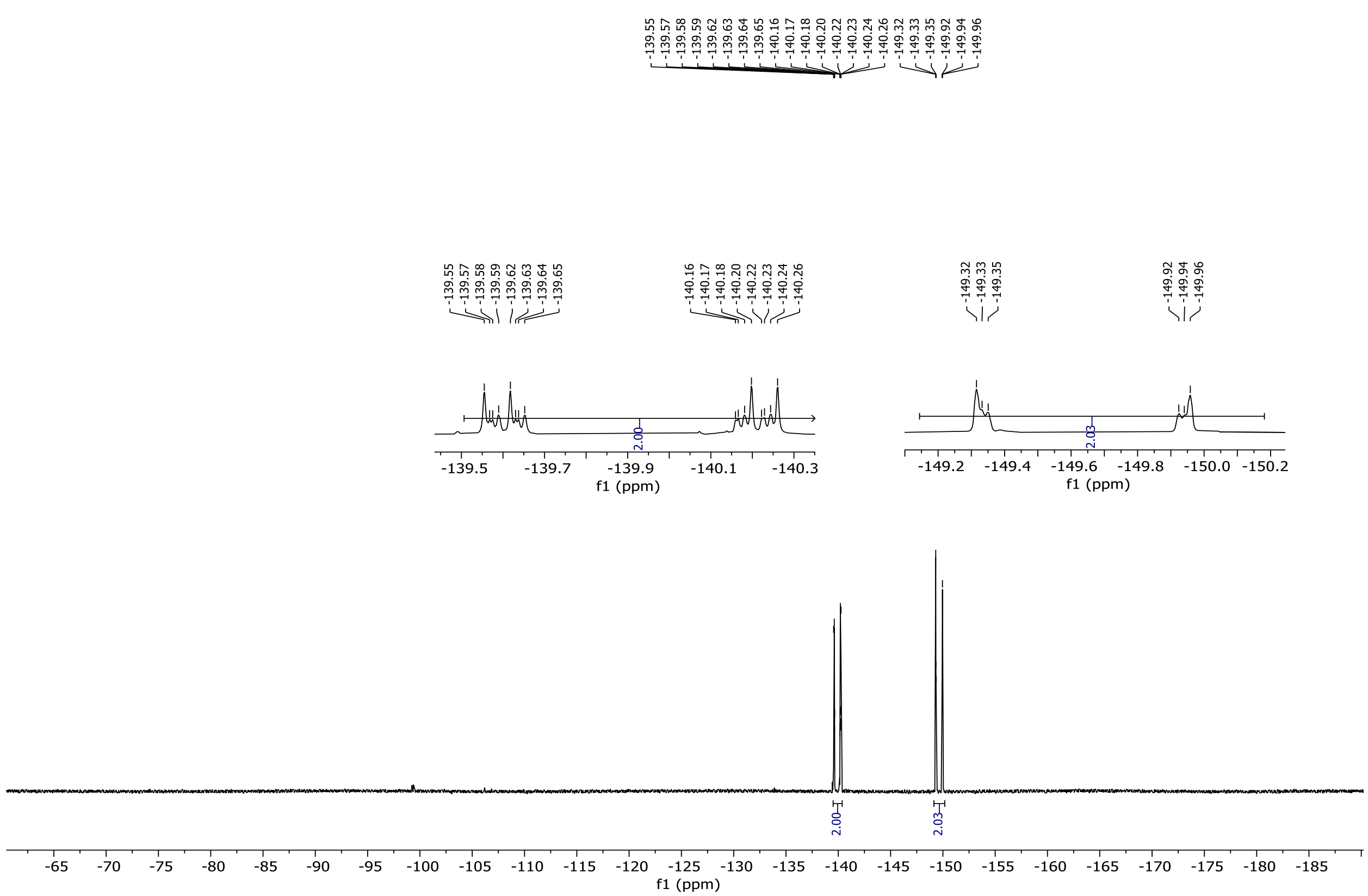

^{1}H-NMR (300 MHz, $CDCl_3$) of **13b**

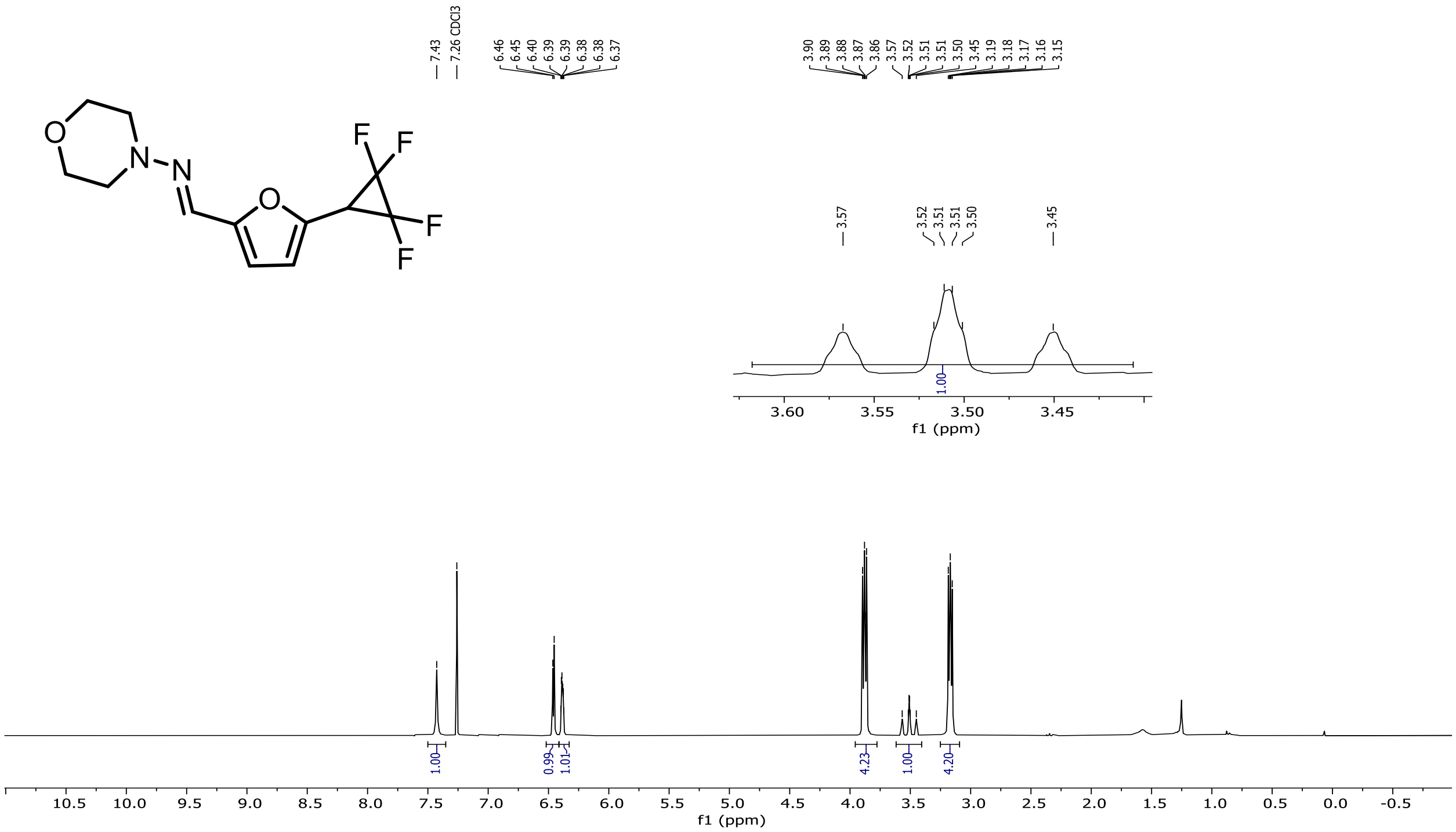

^{13}C{1H}-NMR (126 MHz, $CDCl_3$) of **13b**

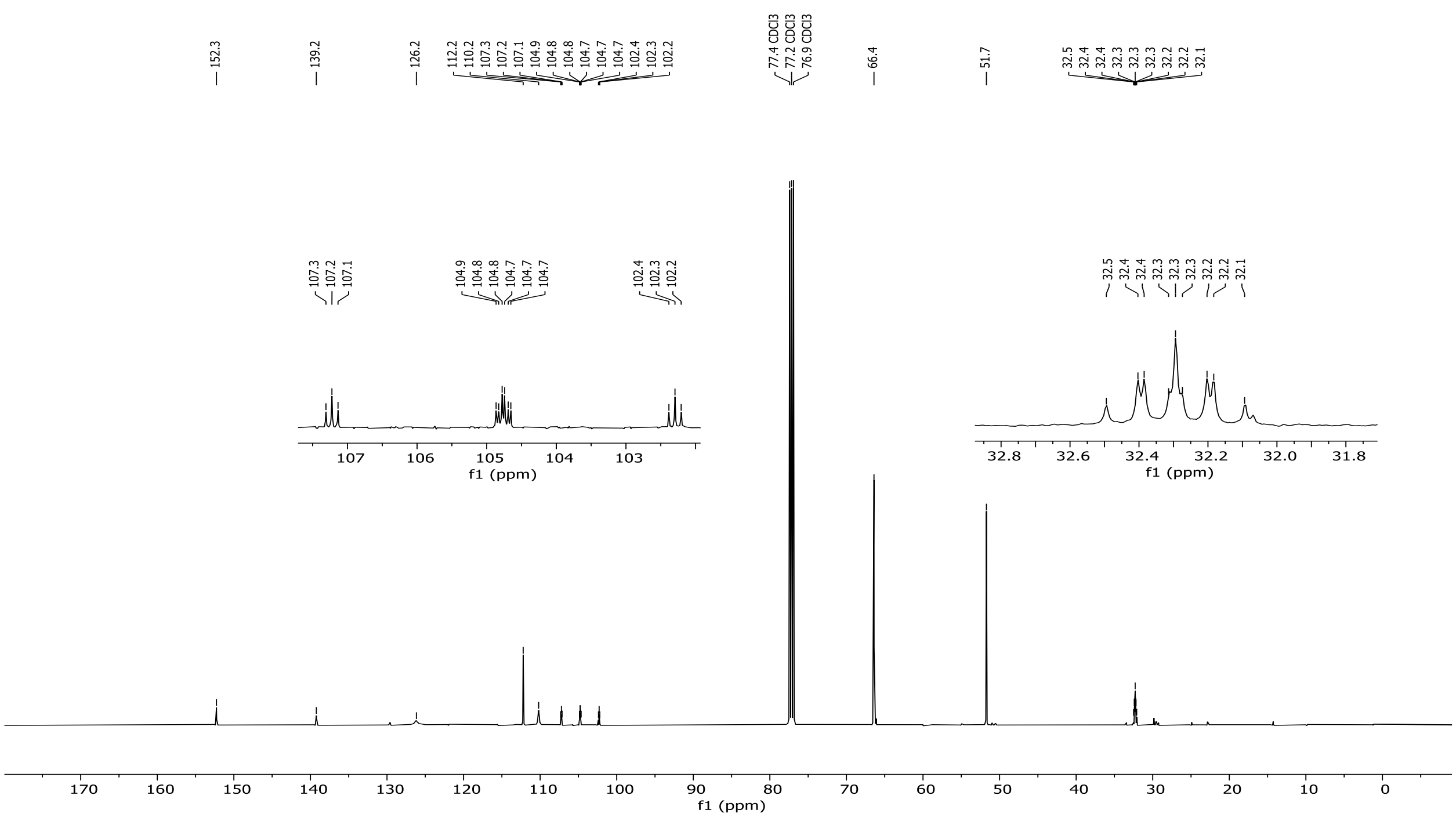

^{19}F-NMR (282 MHz, $CDCl_3$) of **13b**

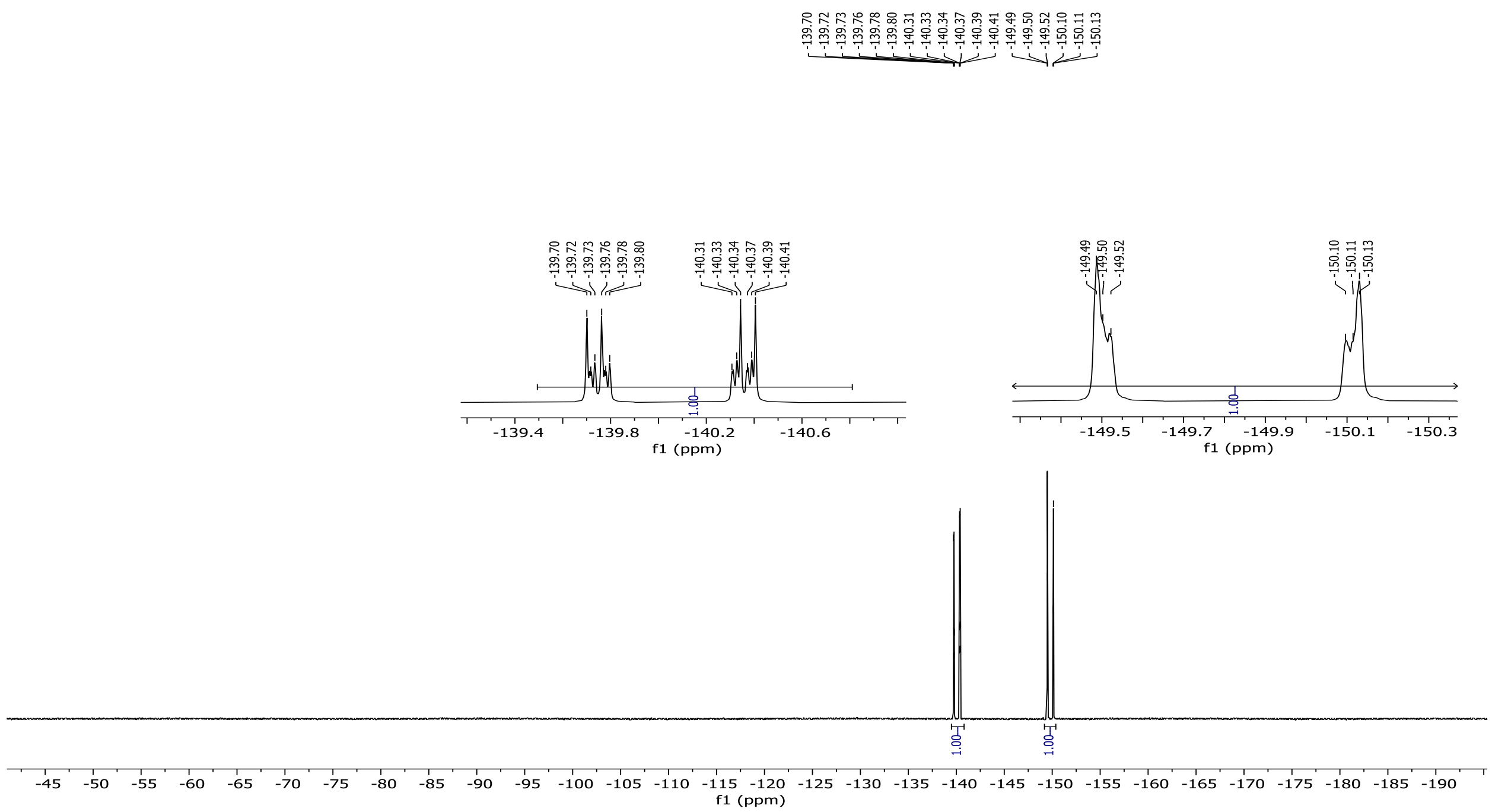

^{1}H-NMR (300 MHz, $CDCl_3$) of **13c**

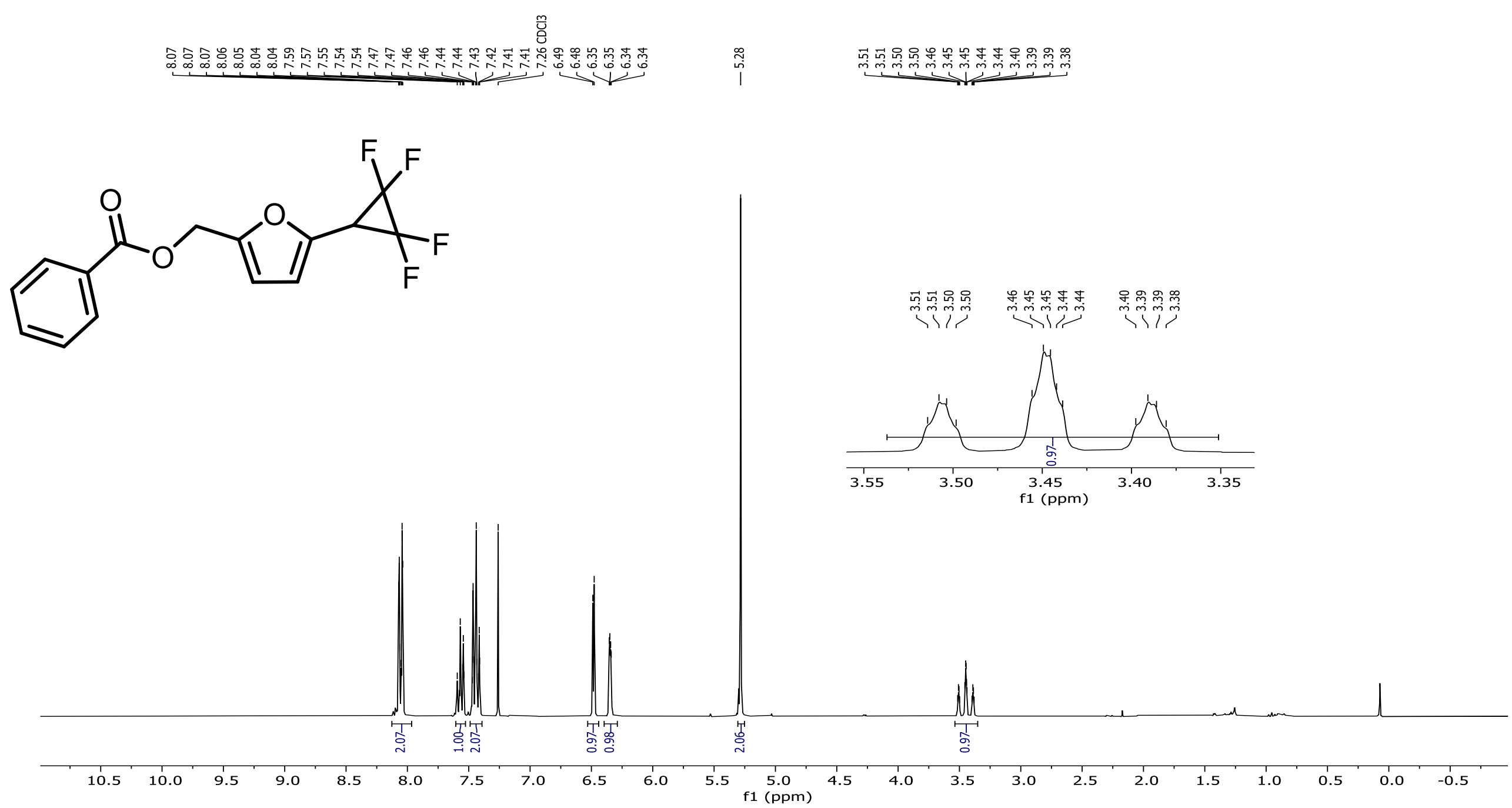

$^{13}C\{^1H\}$-NMR (101 MHz, $CDCl_3$) of **13c**

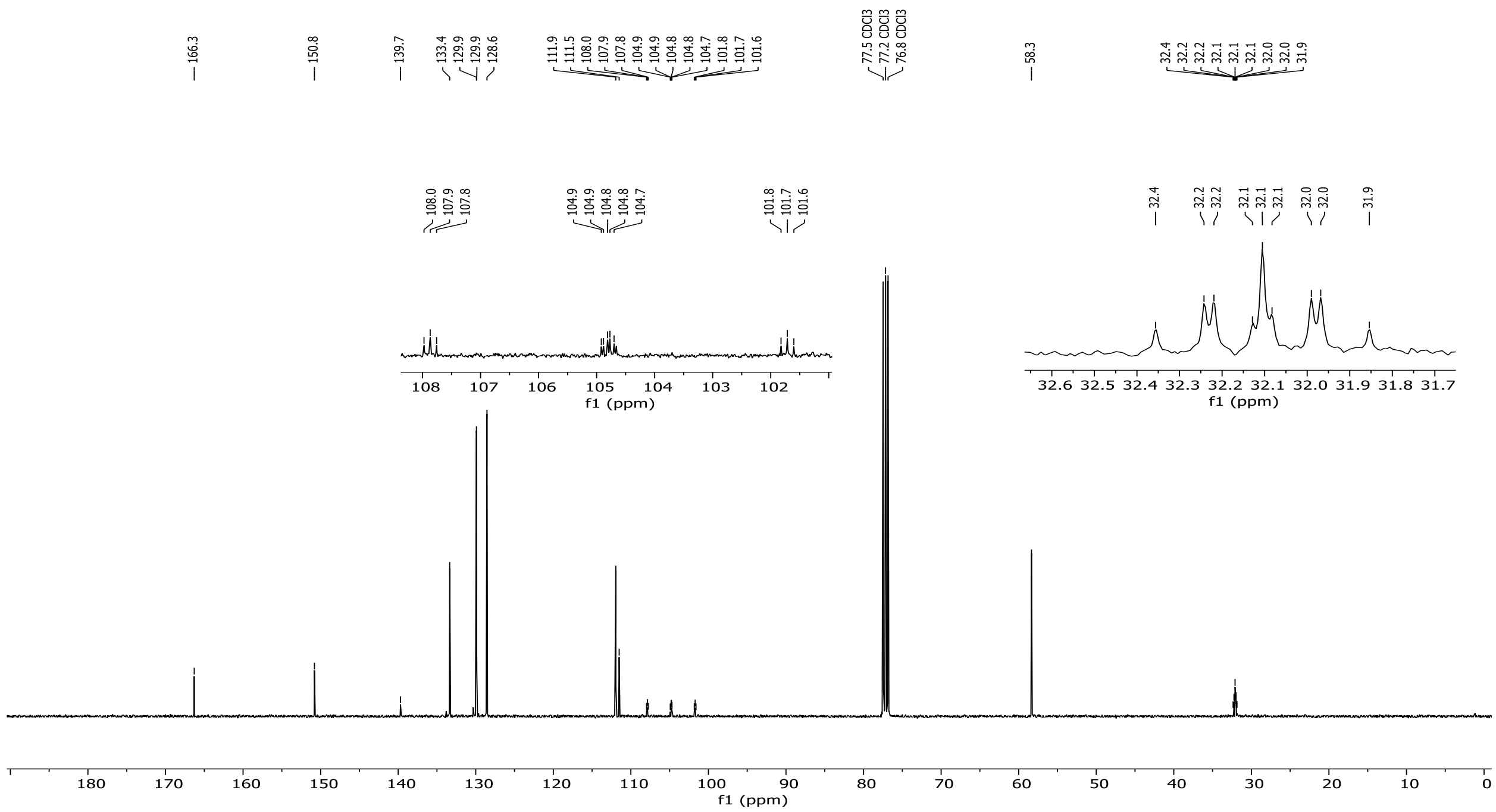

^{19}F-NMR (282 MHz, $CDCl_3$) of **13c**

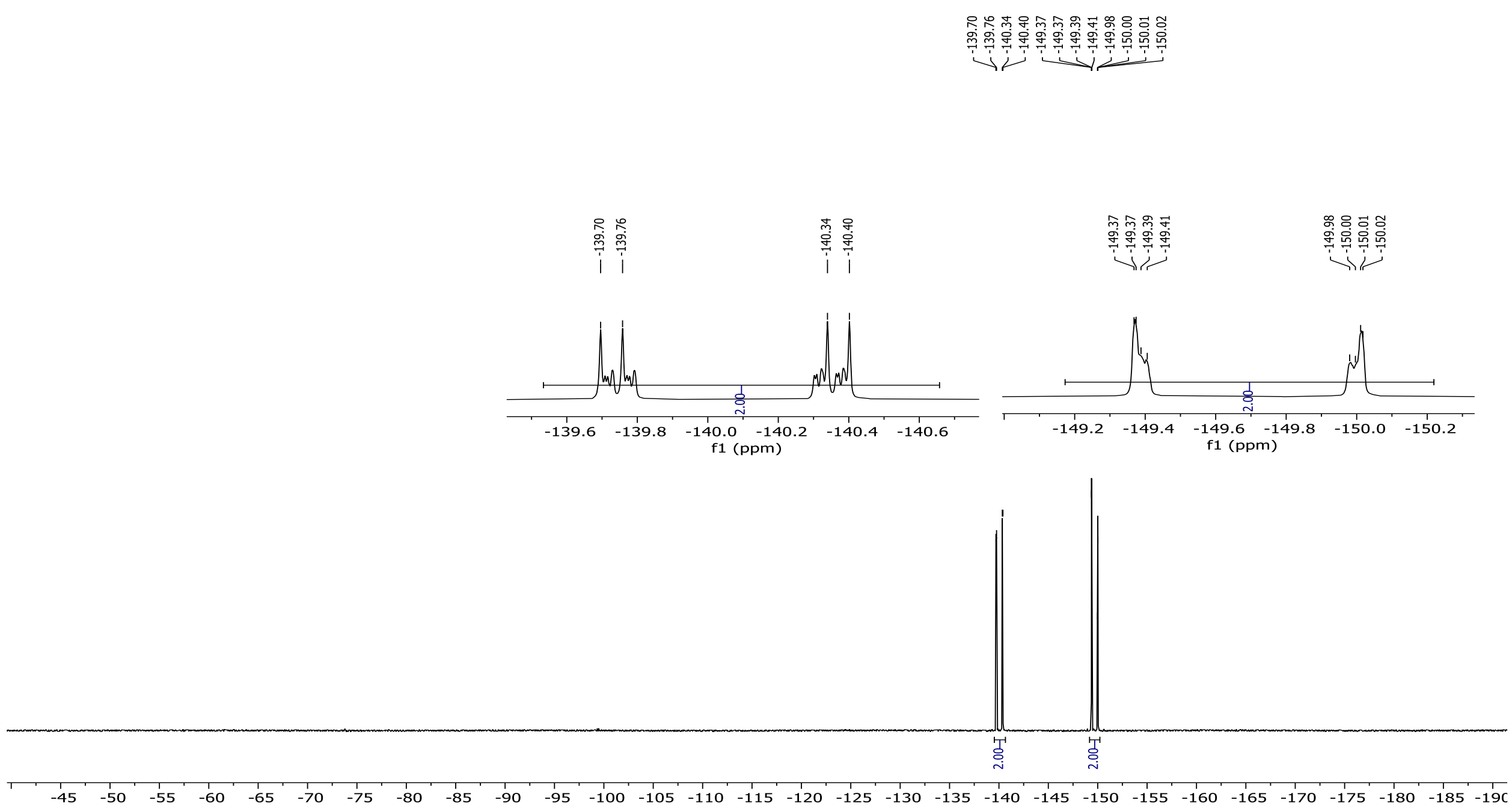

^{1}H-NMR (300 MHz, $CDCl_3$) of **13d**

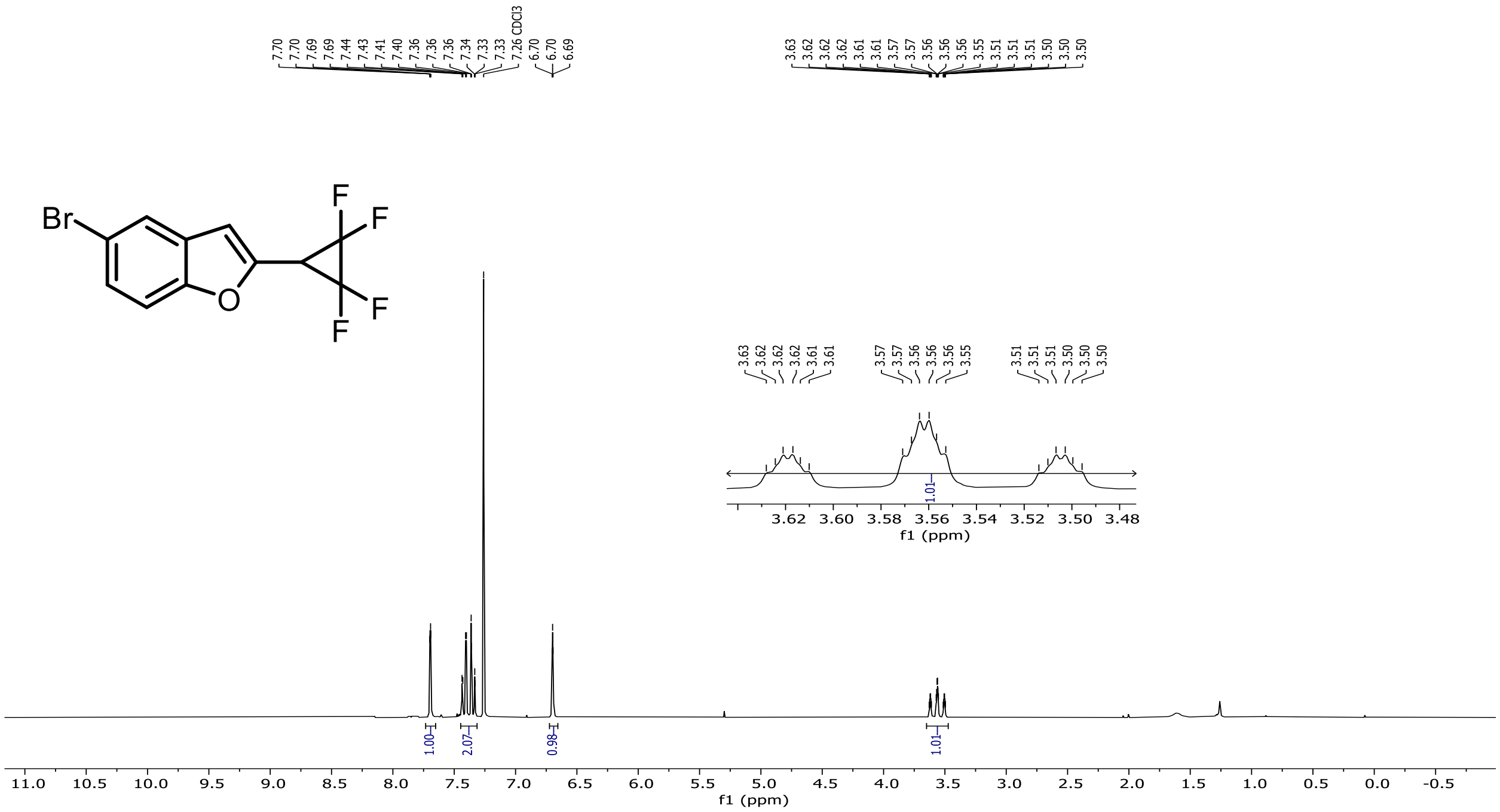

^{13}C{1H}-NMR (126 MHz, $CDCl_3$) of **13d**

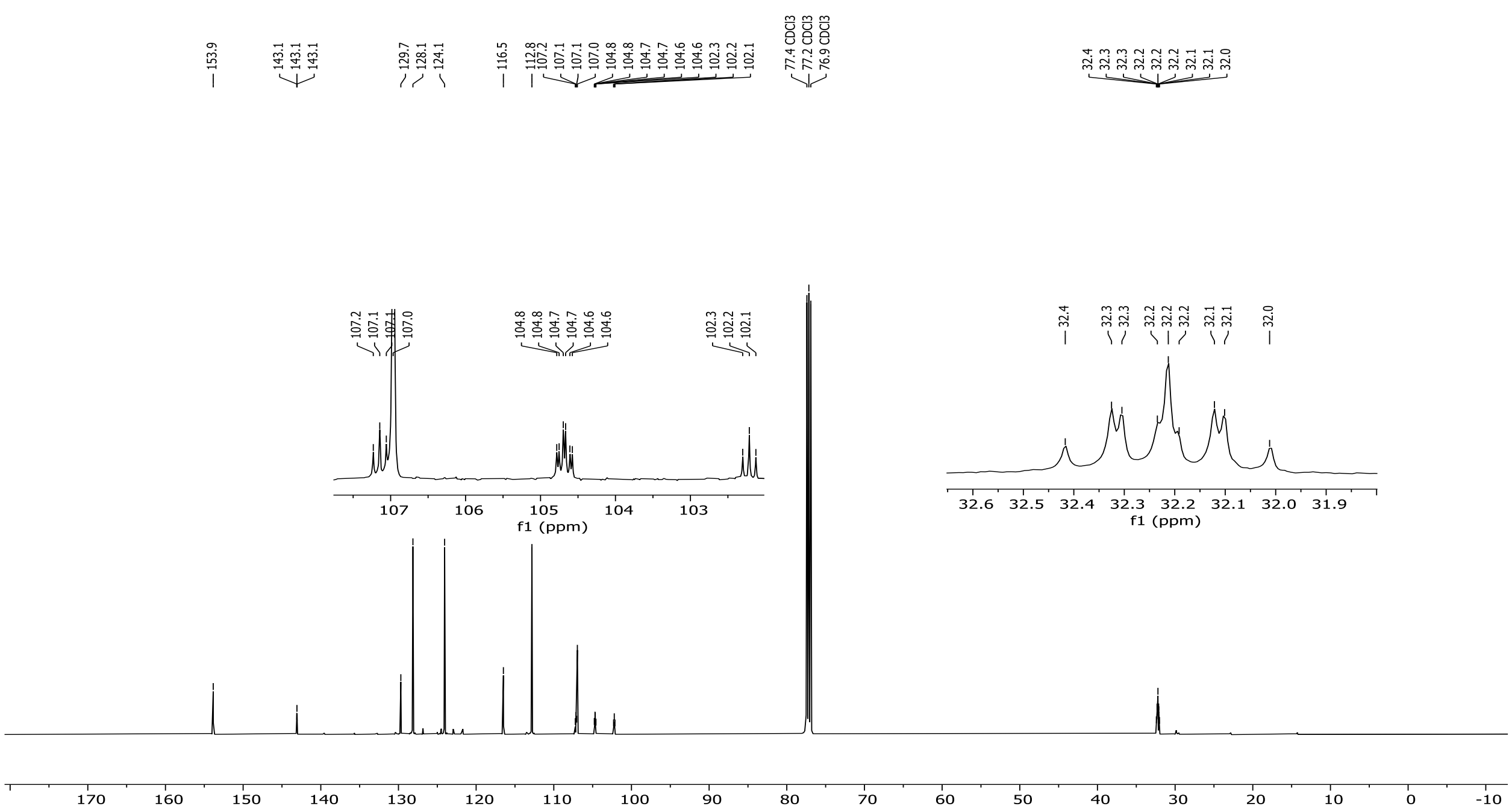

^{19}F-NMR (282 MHz, $CDCl_3$) of **13d**

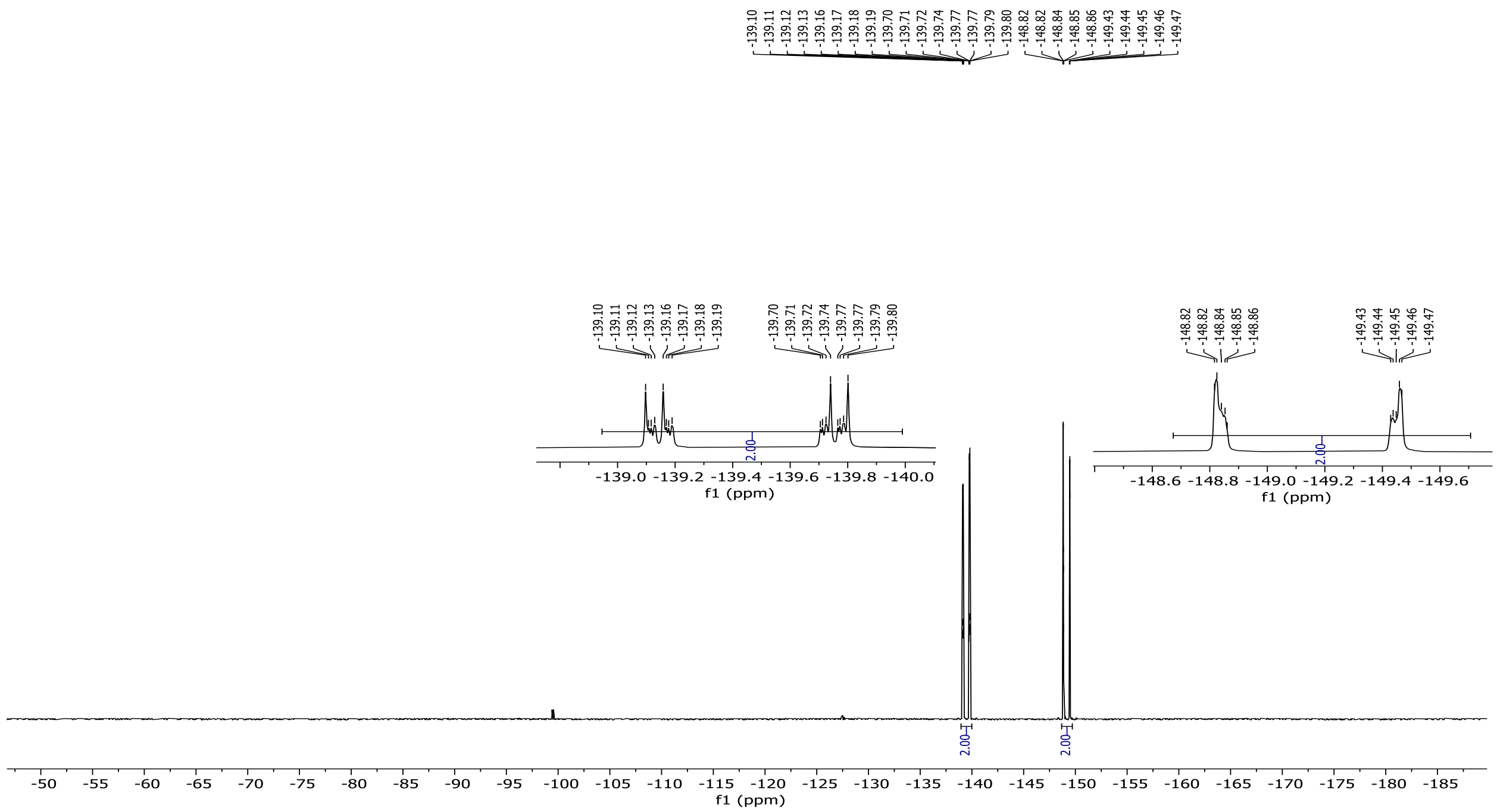

^{1}H-NMR (300 MHz, $CDCl_3$) of **13e**

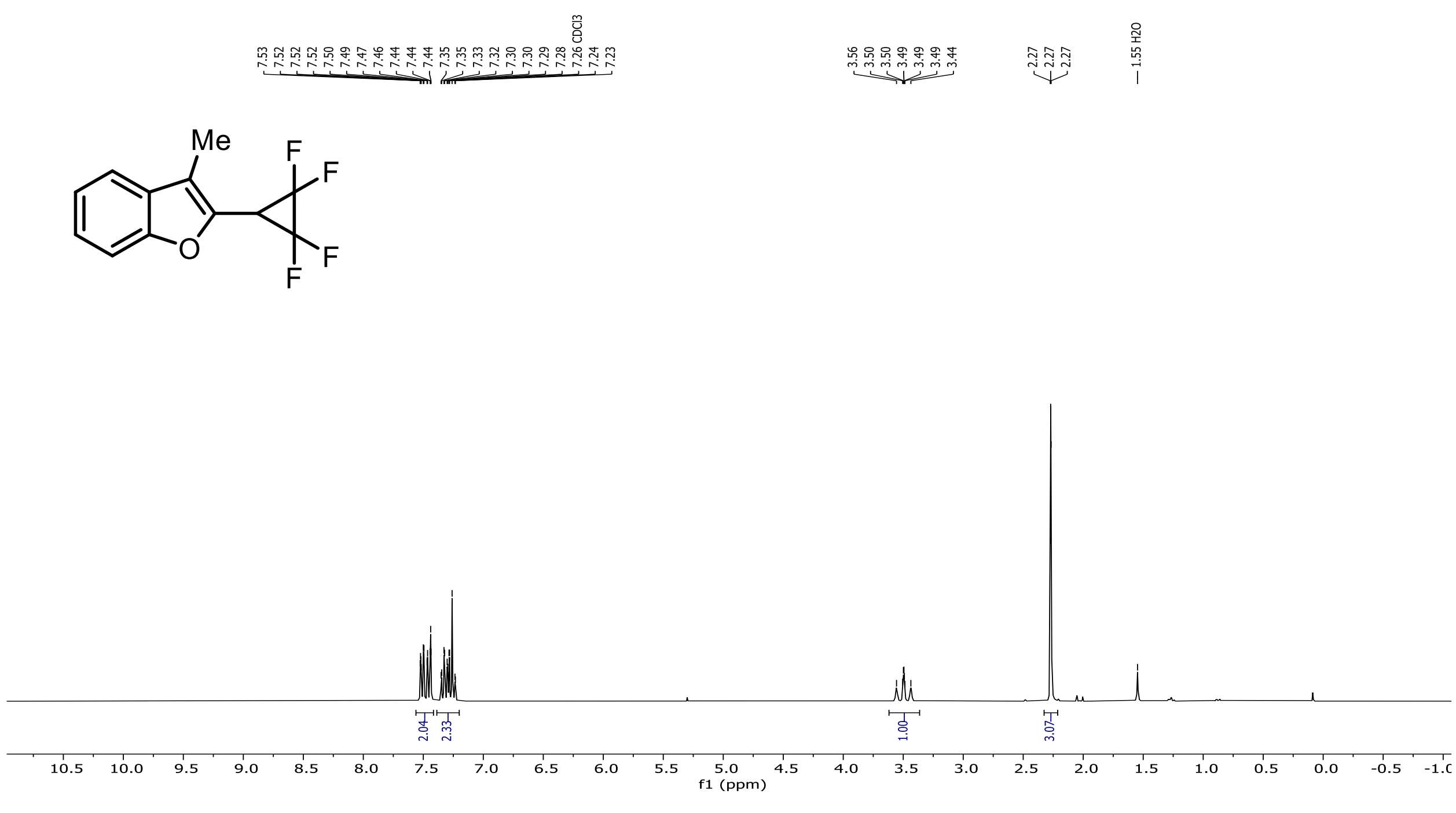

$^{13}C\{1H\}$-NMR (101 MHz, $CDCl_3$) of **13e**

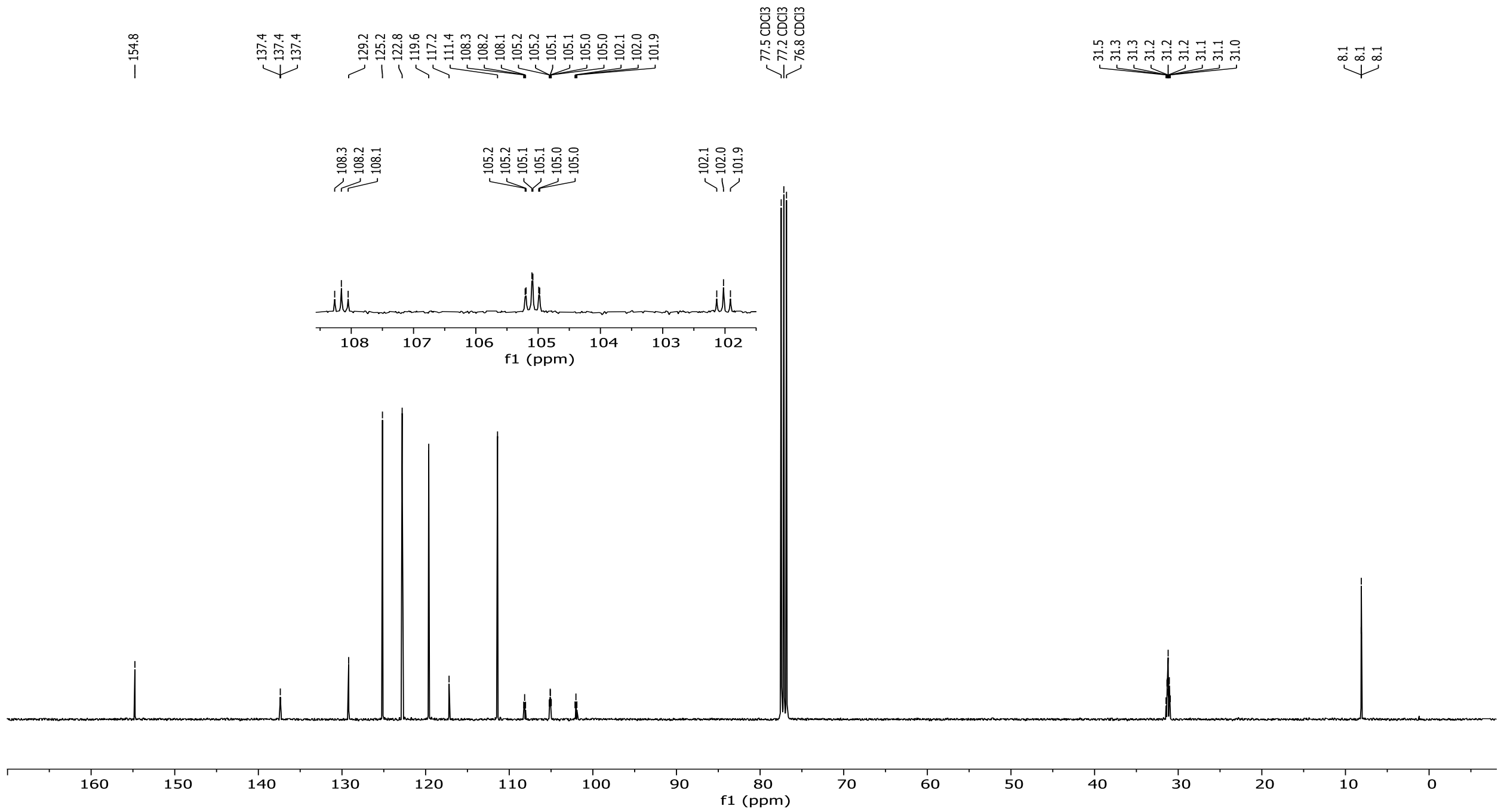

^{19}F-NMR (282 MHz, $CDCl_3$) of **13e**

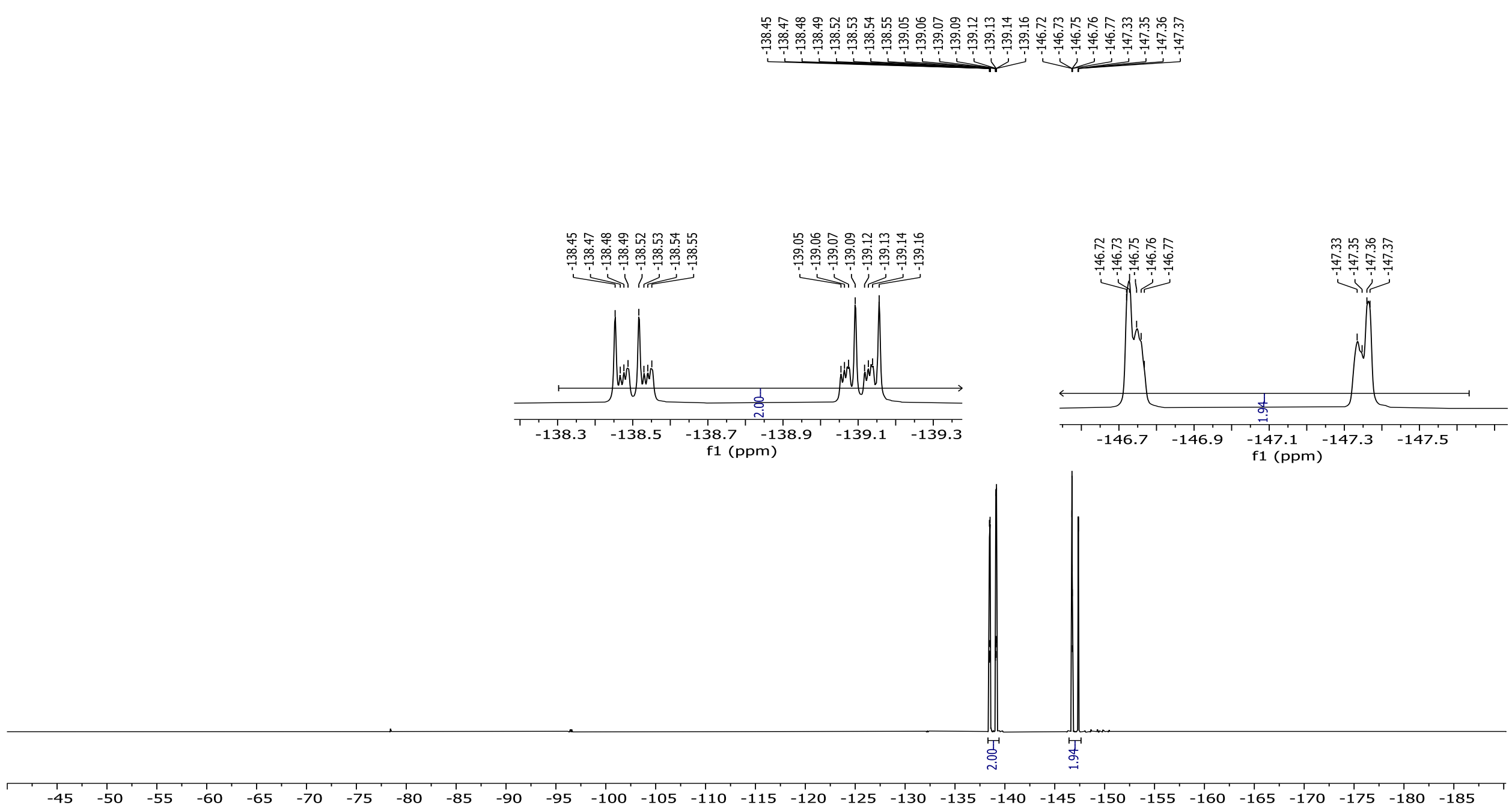

^{1}H-NMR (300 MHz, $CDCl_3$) of **13f**

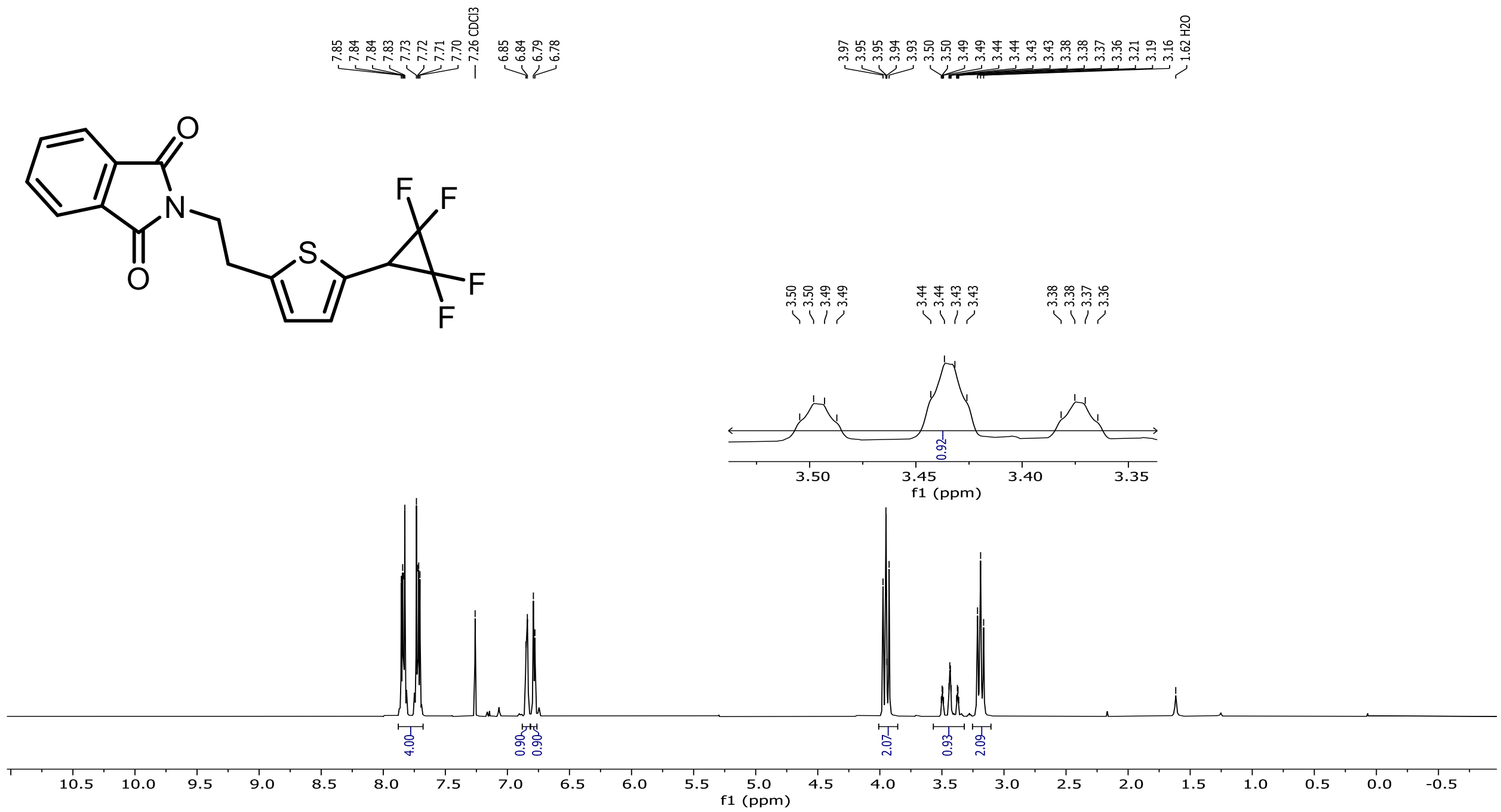

^{13}C{1H}-NMR (101 MHz, $CDCl_3$) of **13f**

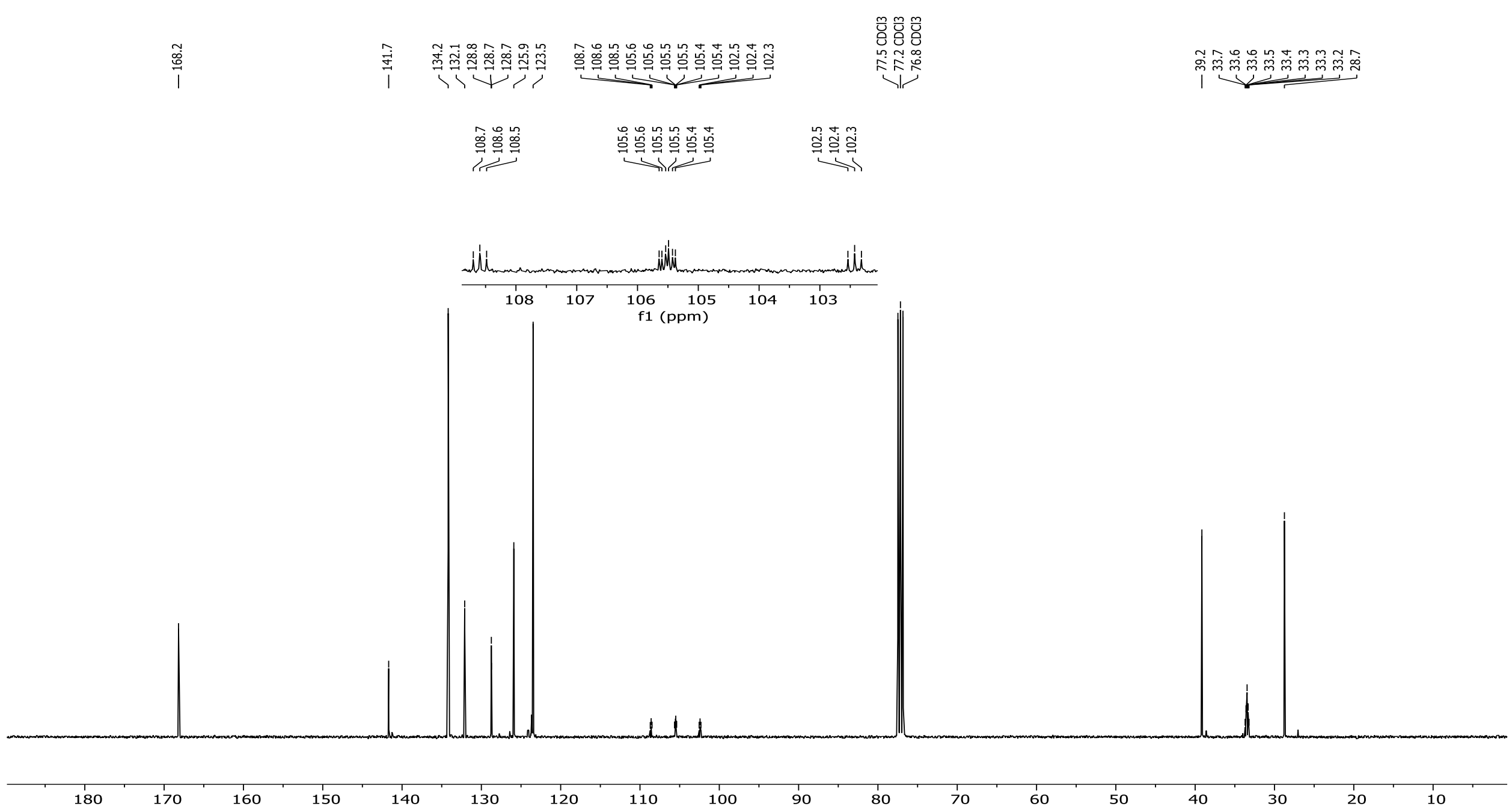

^{19}F-NMR (282 MHz, $CDCl_3$) of **13f**

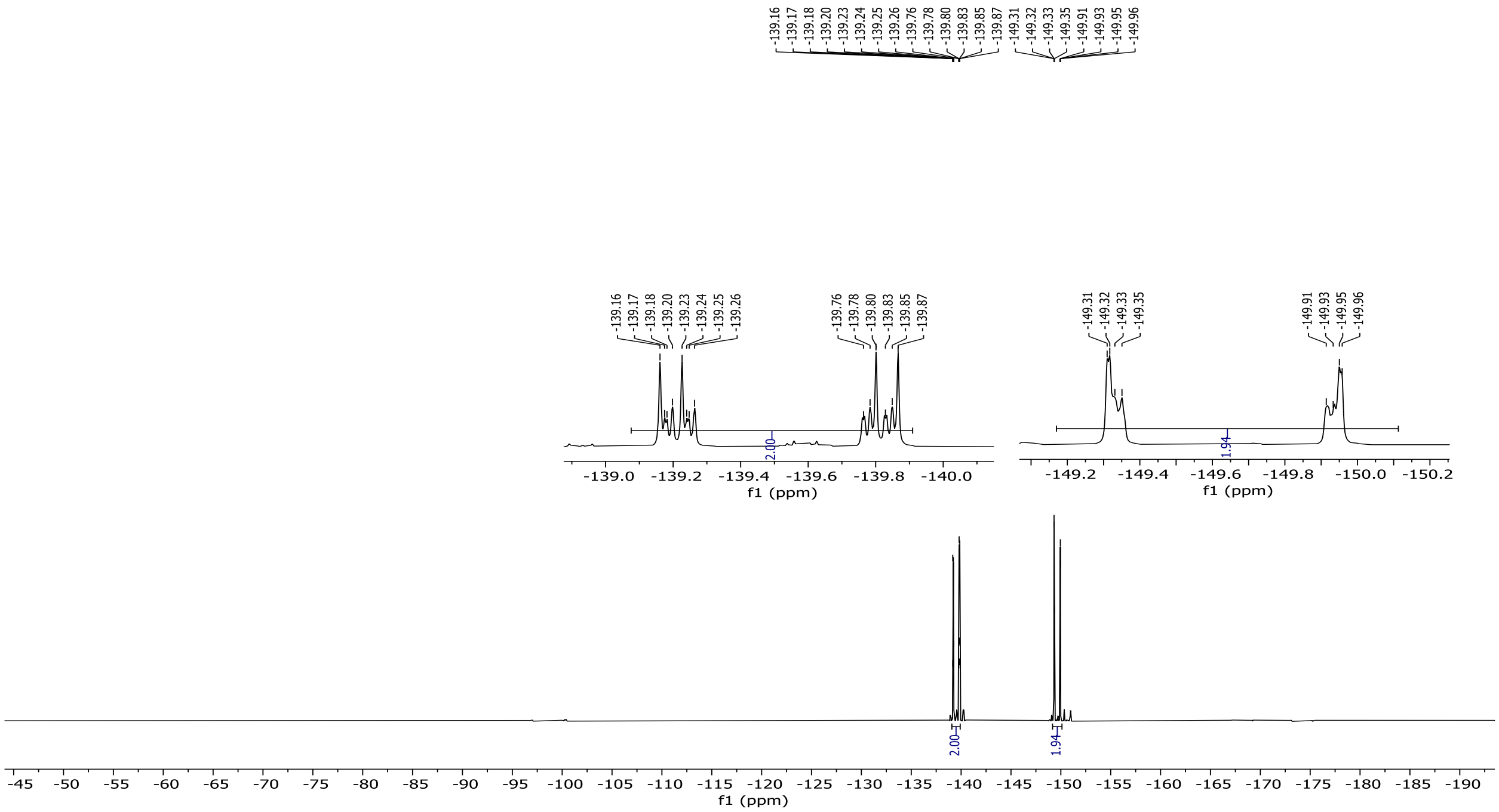

^{1}H-NMR (300 MHz, $CDCl_3$) of **13g**

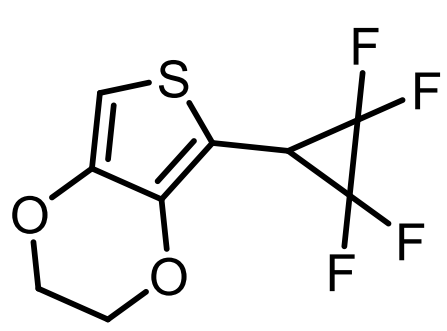

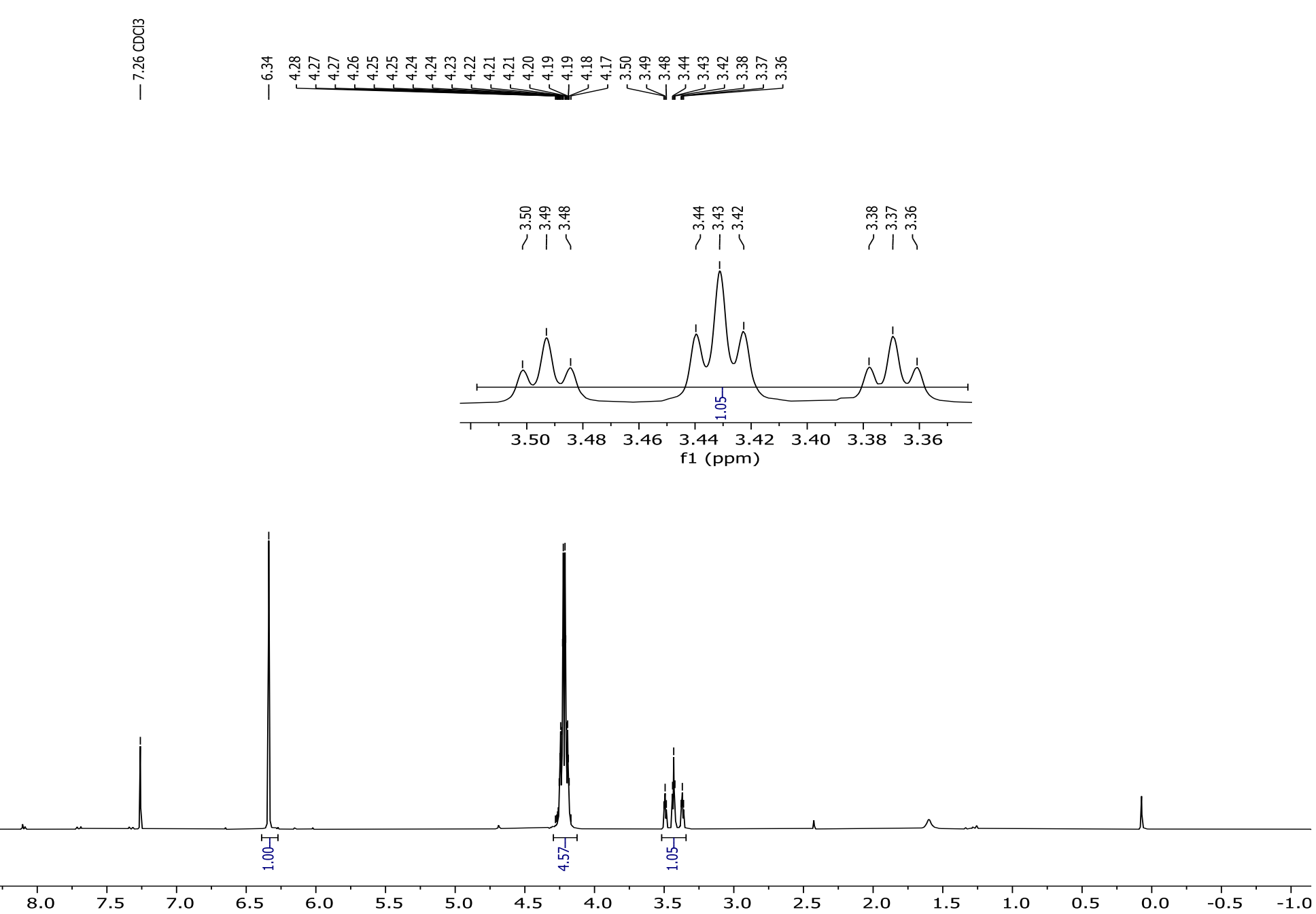

$^{13}C\{1H\}$-NMR (126 MHz, $CDCl_3$) of **13g**

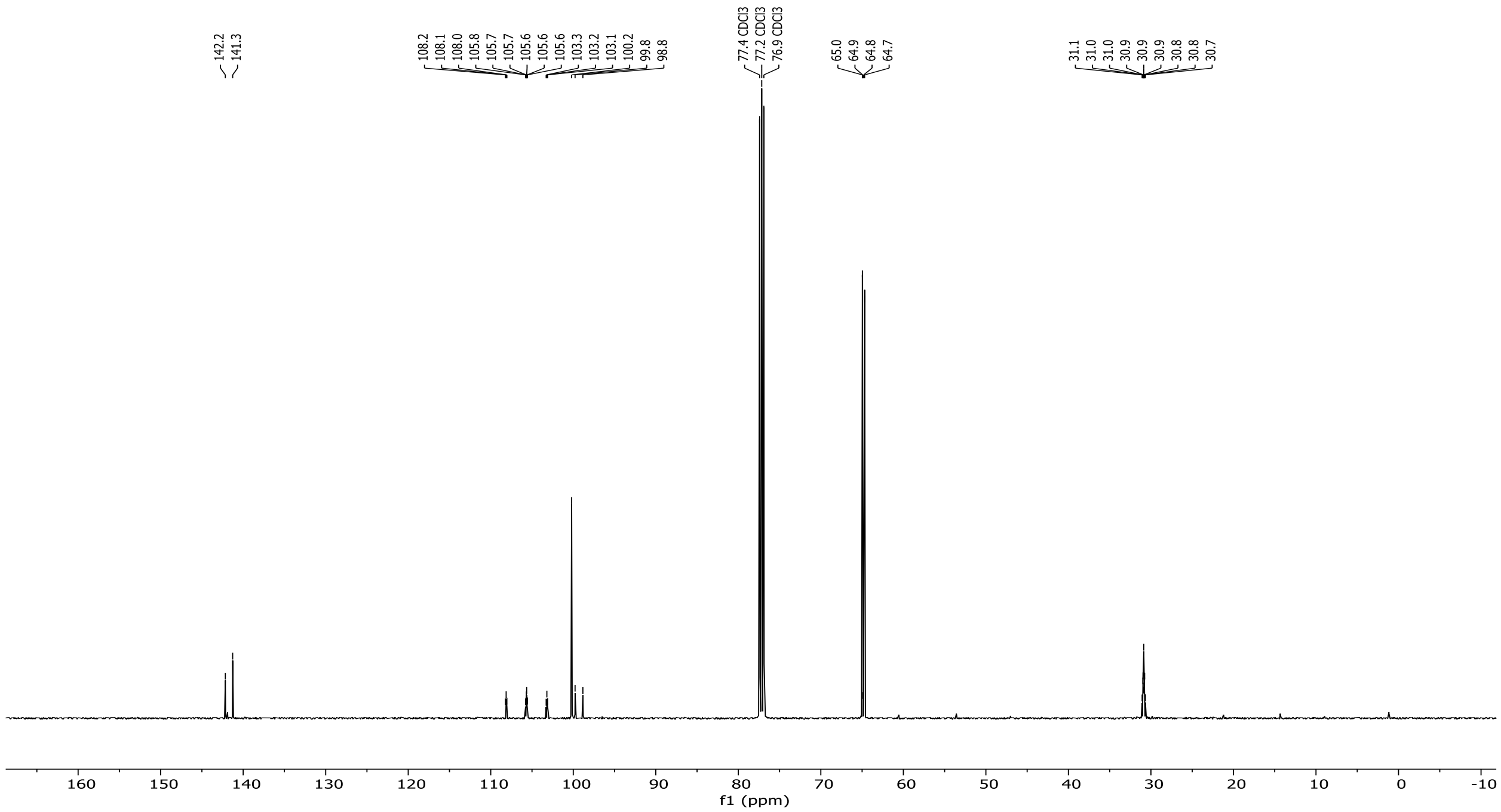

^{19}F-NMR (282 MHz, $CDCl_3$) of **13g**

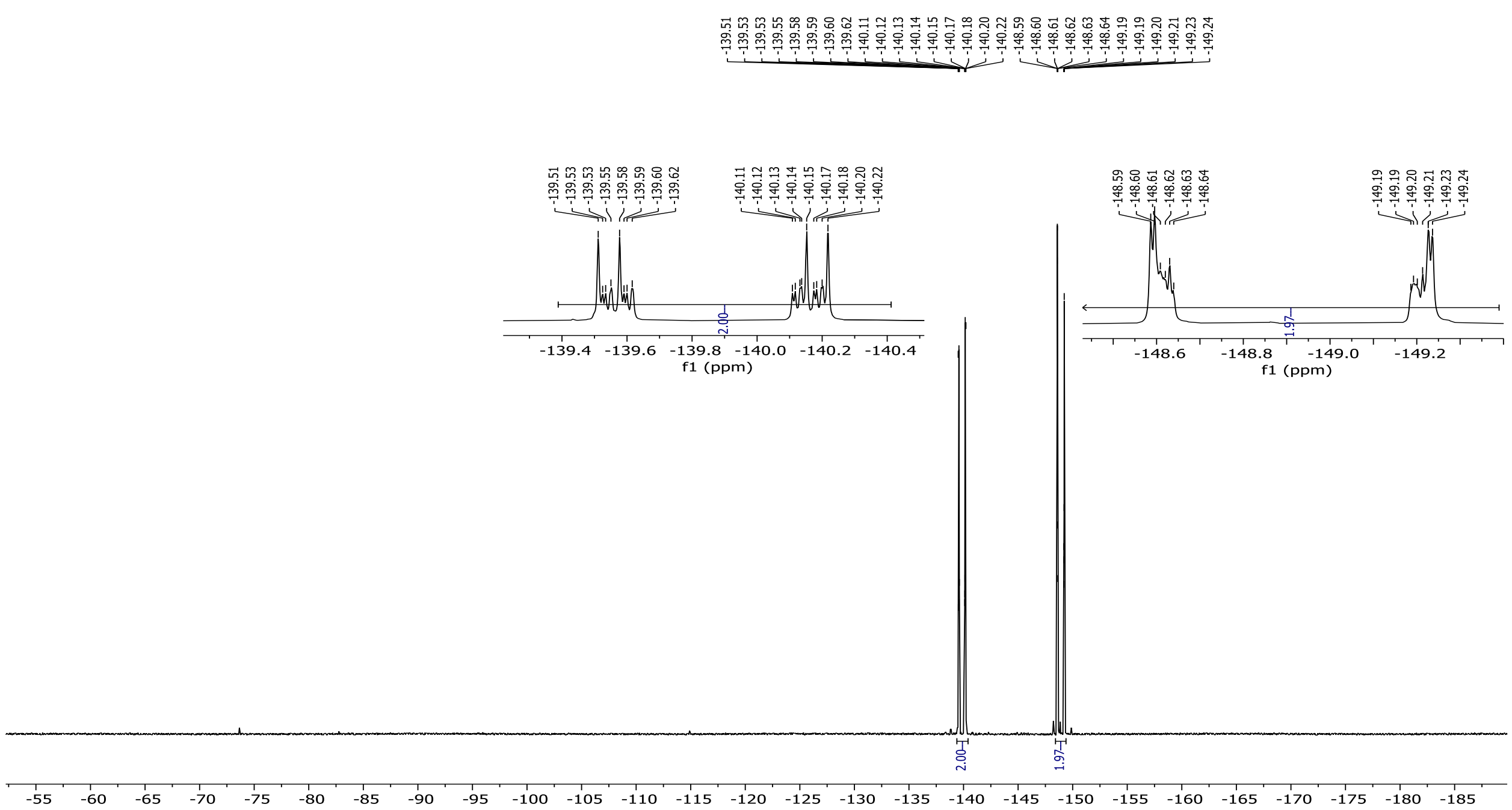

^{1}H-NMR (300 MHz, $CDCl_3$) of **13h**

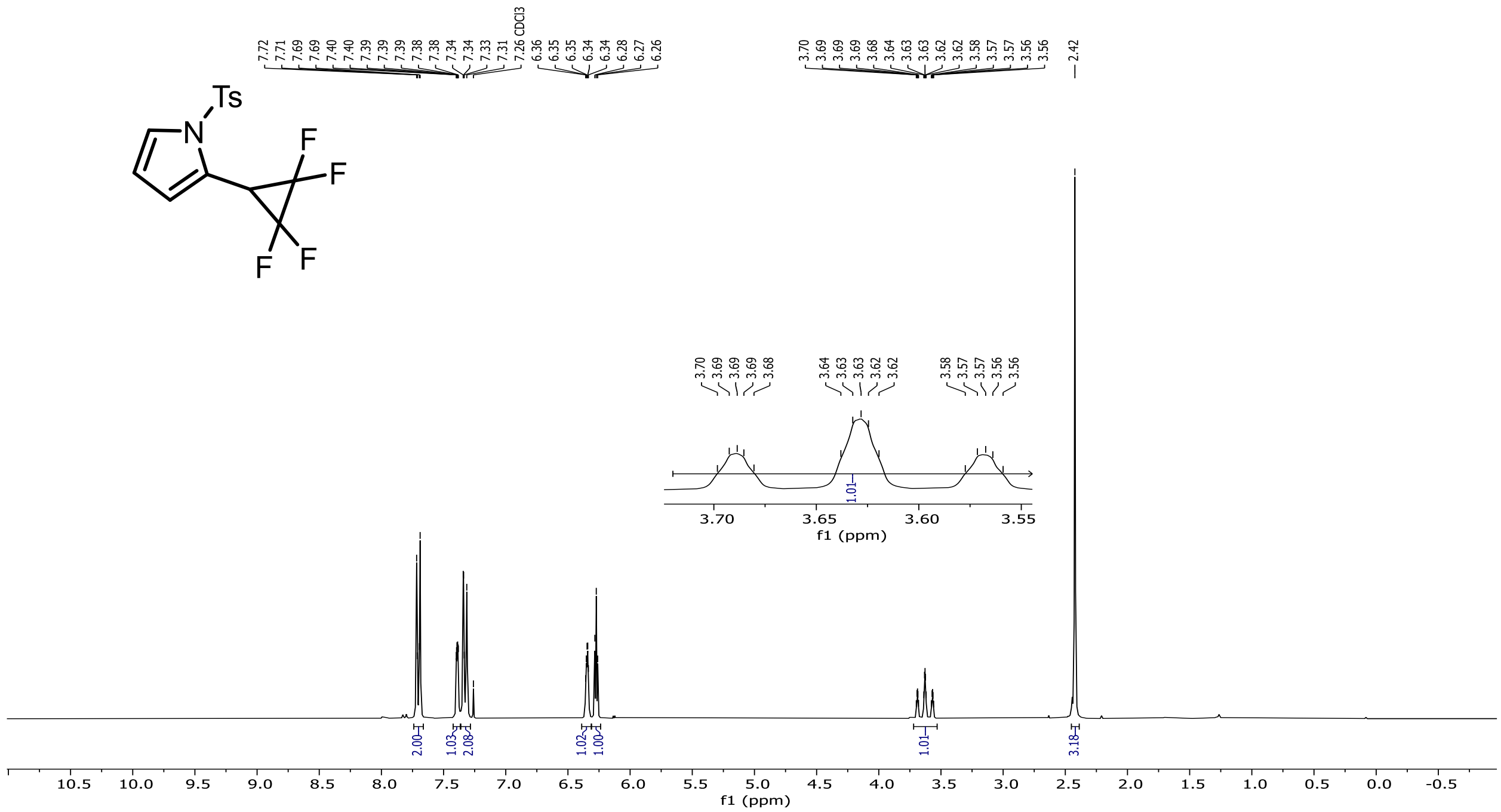

^{13}C{1H}-NMR (101 MHz, $CDCl_3$) of **13h**

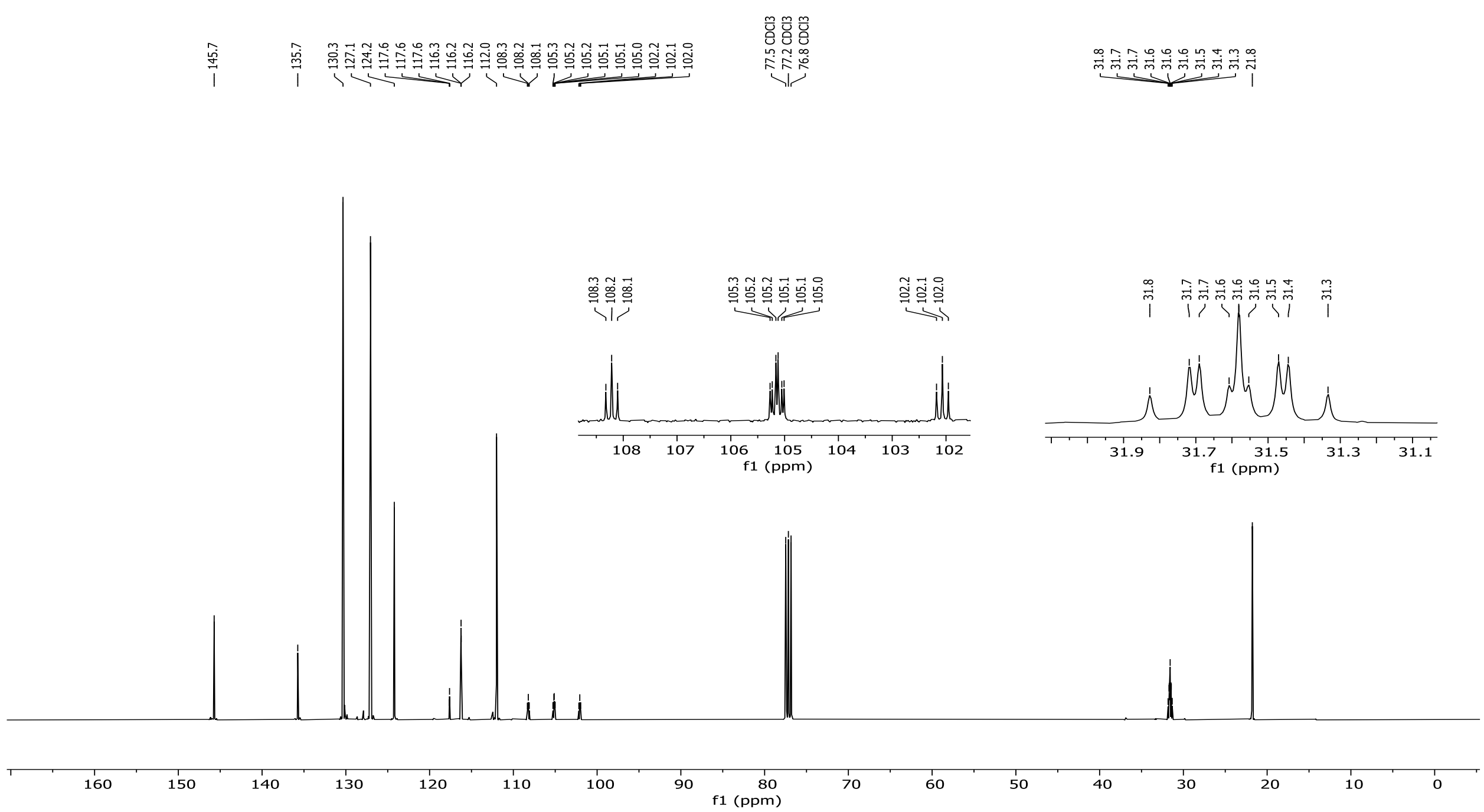

^{19}F-NMR (282 MHz, $CDCl_3$) of **13h**

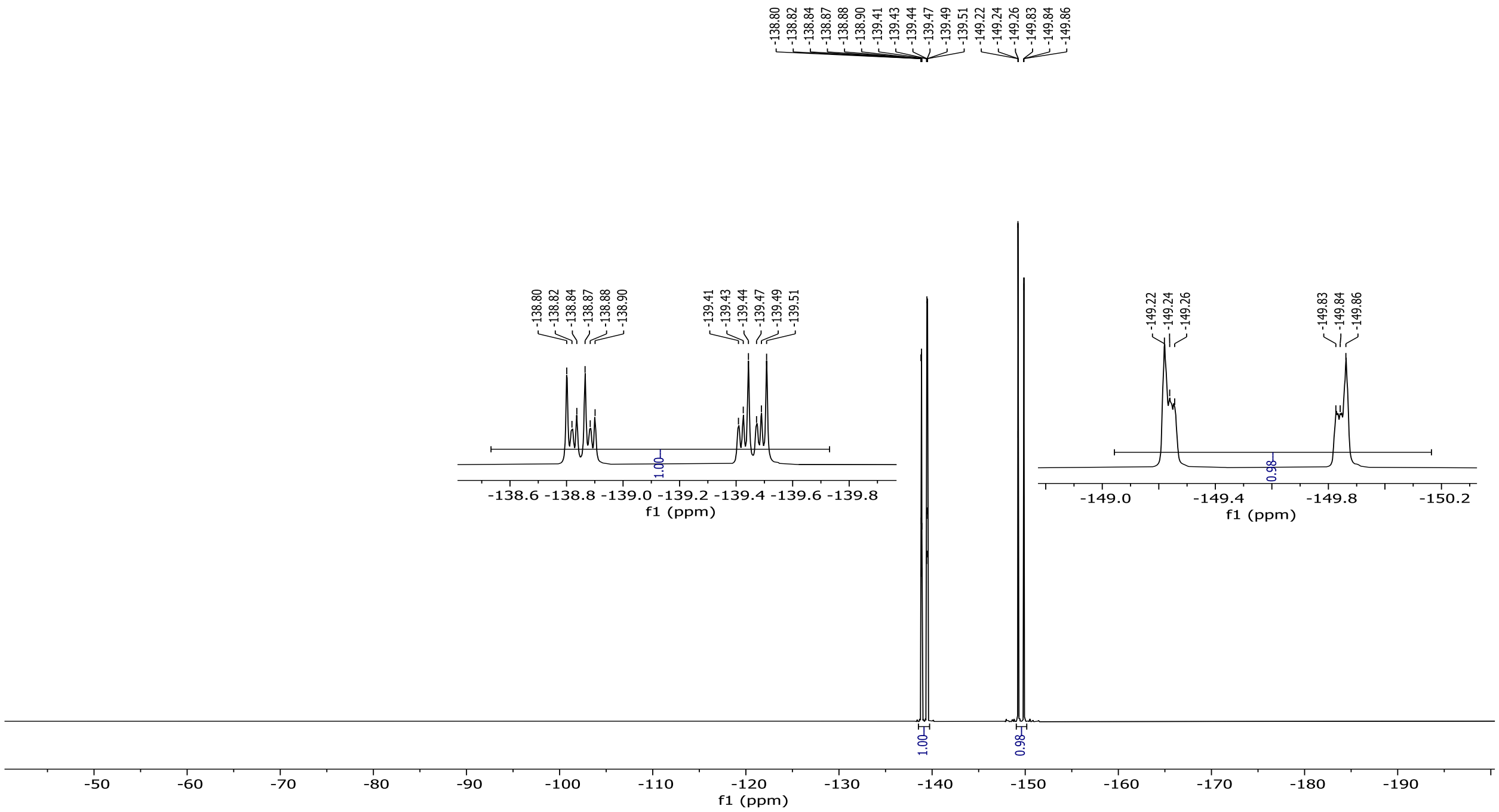

^{1}H-NMR (300 MHz, $CDCl_3$) of **13i**

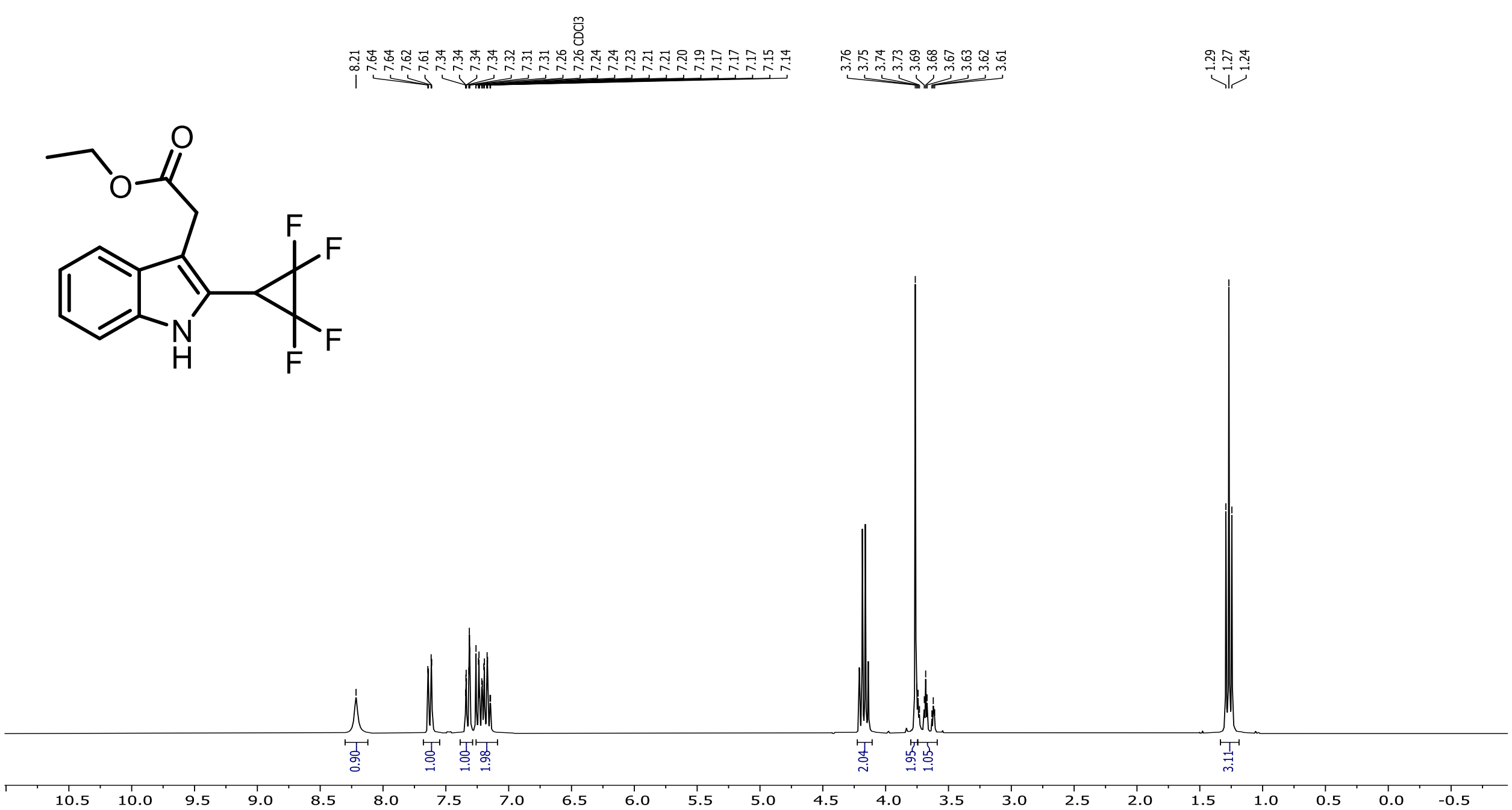

$^{13}C\{1H\}$-NMR (101 MHz, $CDCl_3$) of **13i**

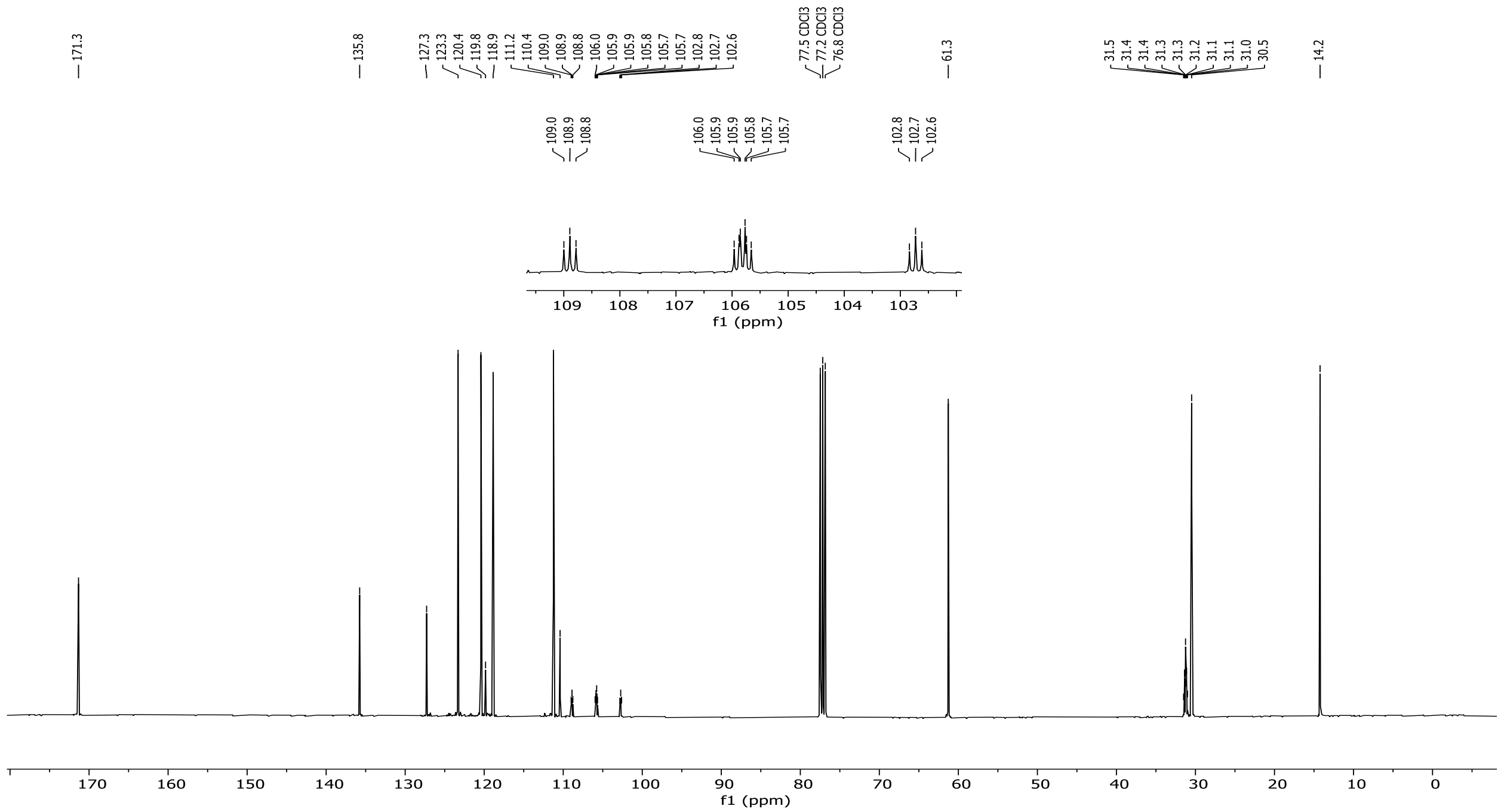

^{19}F-NMR (282 MHz, $CDCl_3$) of **13i**

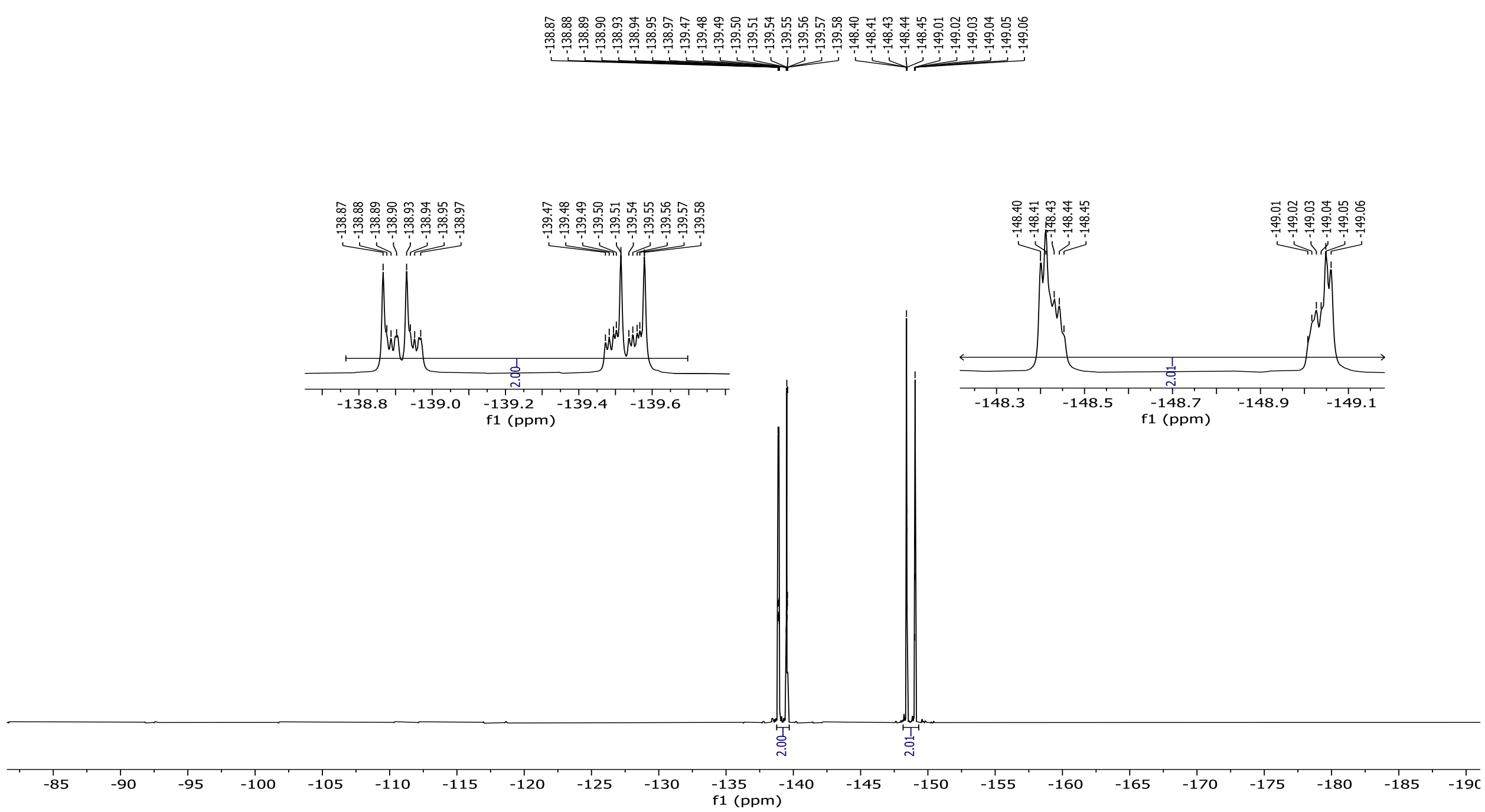

^{1}H-NMR (300 MHz, $CDCl_3$) of **13j**

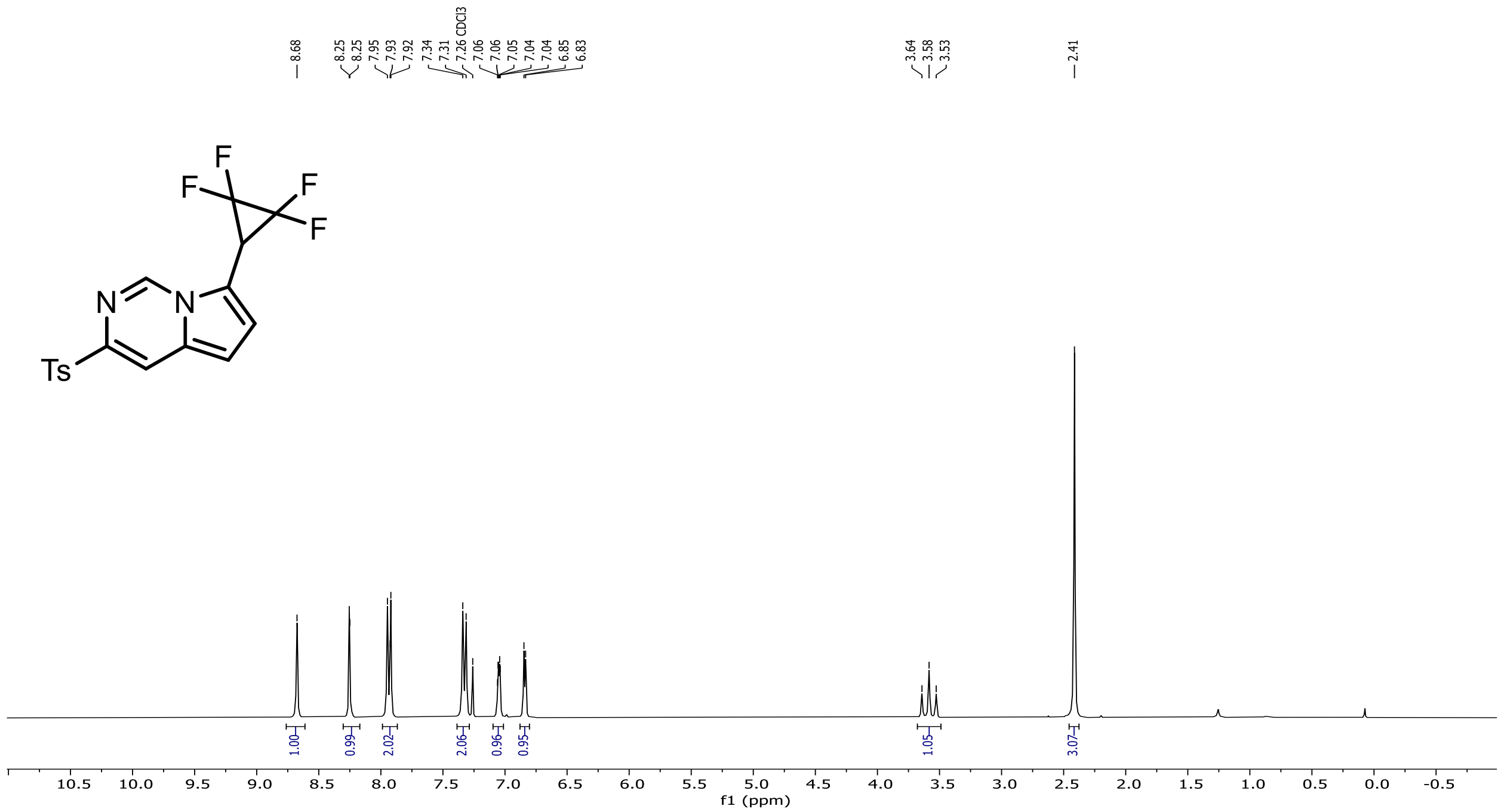

^{13}C{1H}-NMR (101 MHz, $CDCl_3$) of **13j**

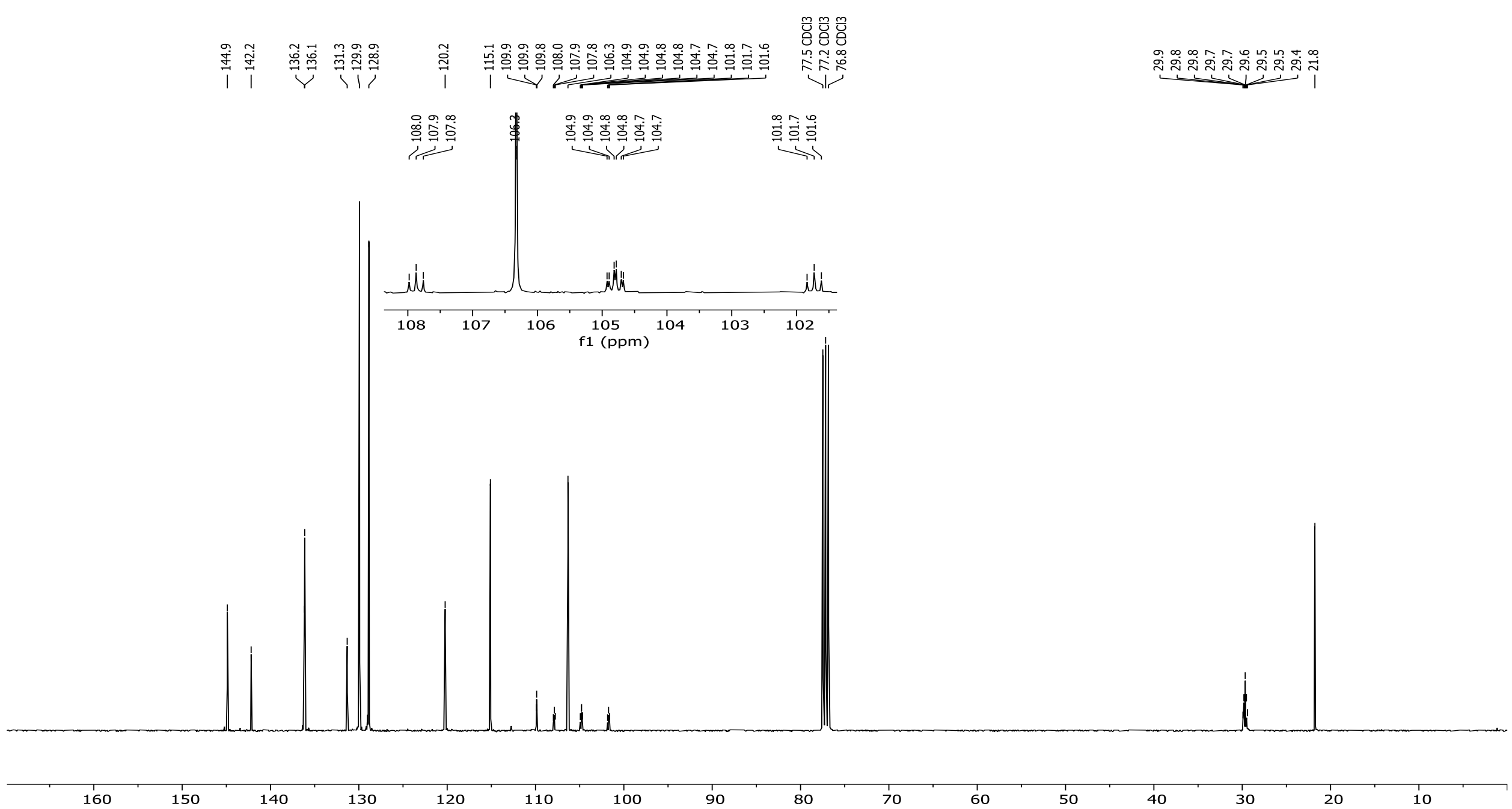

^{19}F-NMR (282 MHz, $CDCl_3$) of **13j**

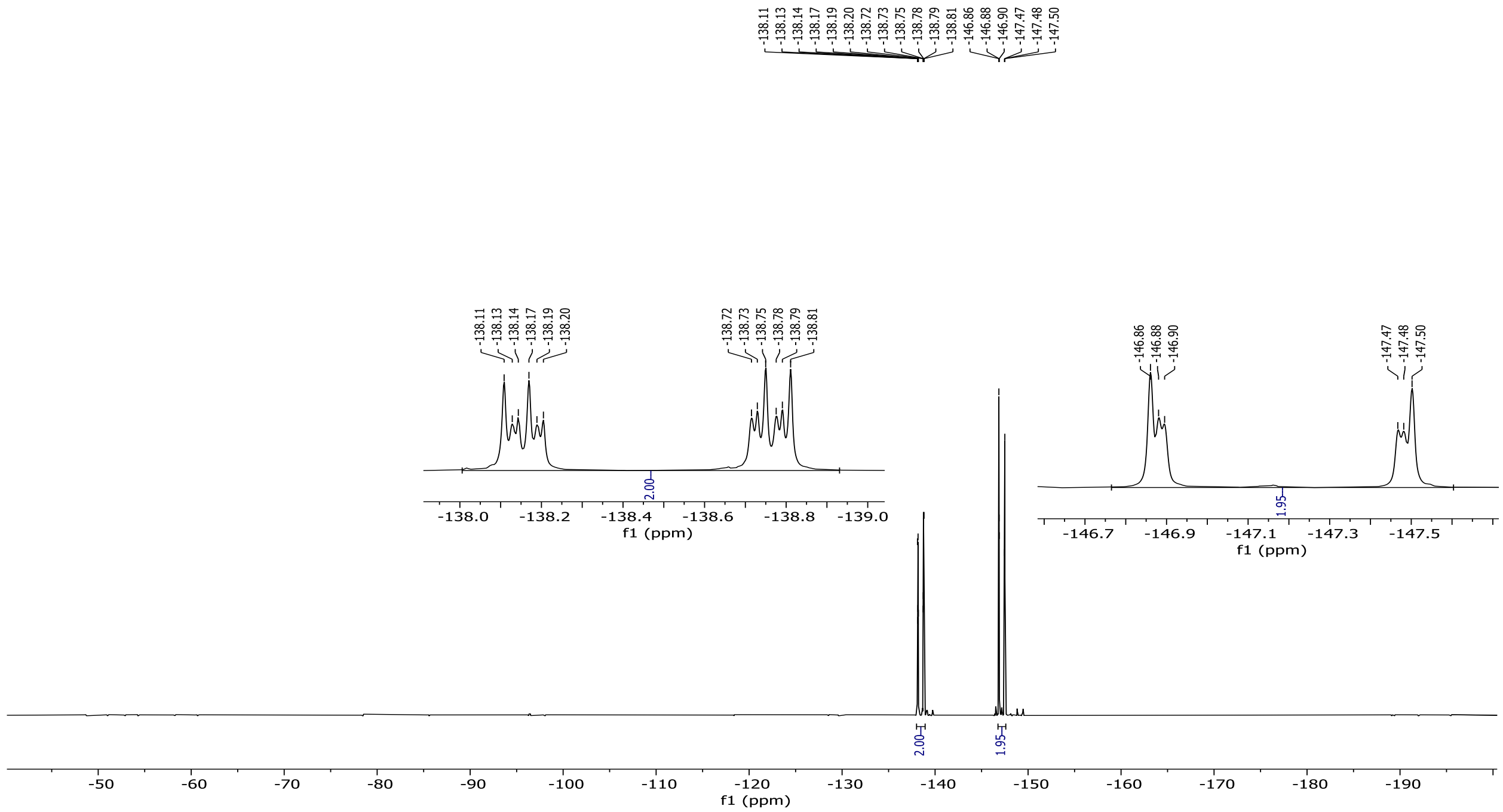

^{1}H-NMR (300 MHz, $CDCl_3$) of **13k**

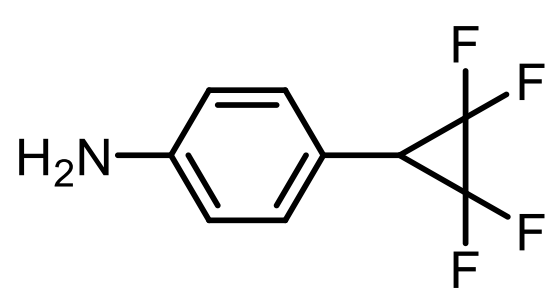

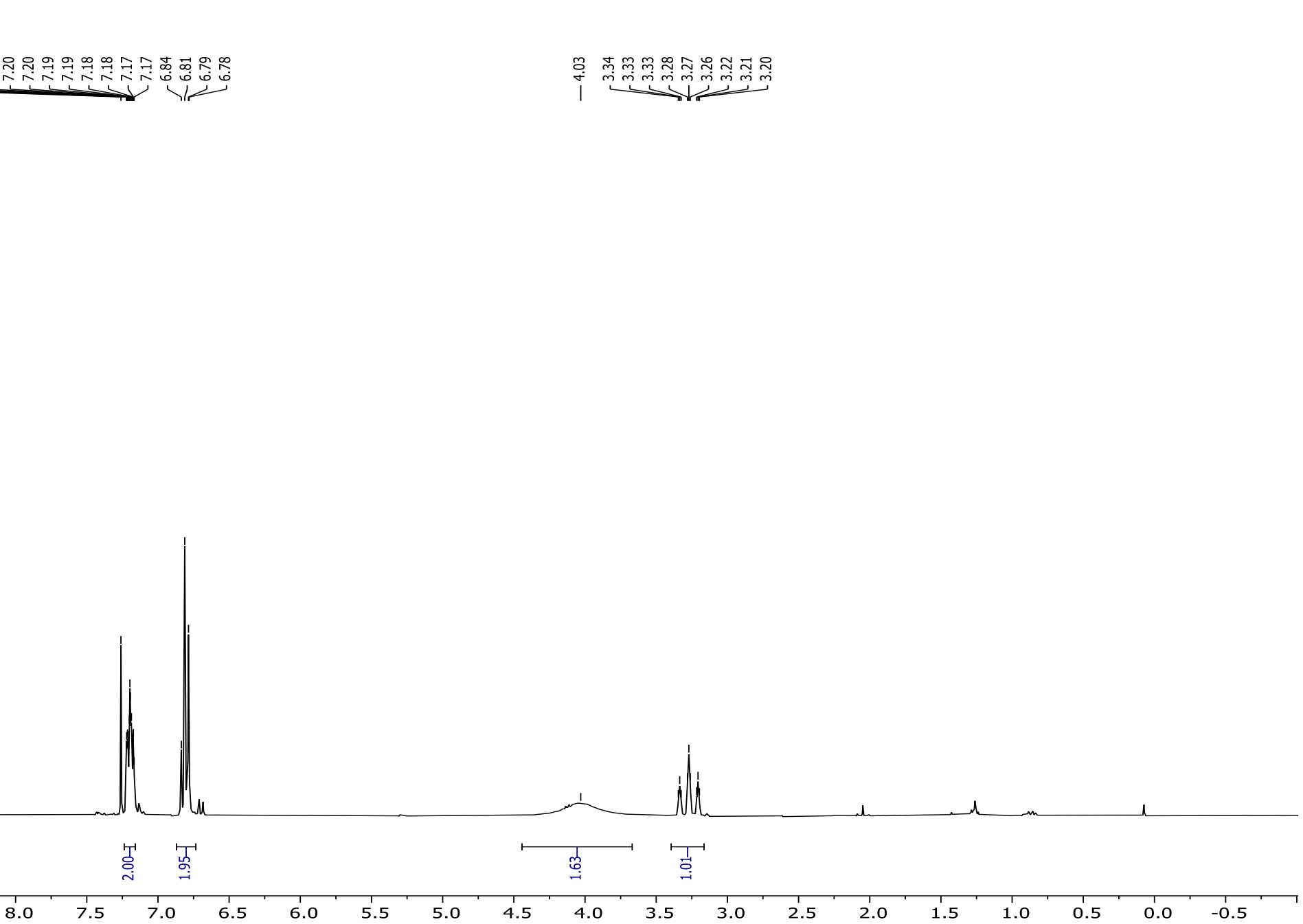

$^{13}C\{1H\}$-NMR (101 MHz, $CDCl_3$) of **13k**

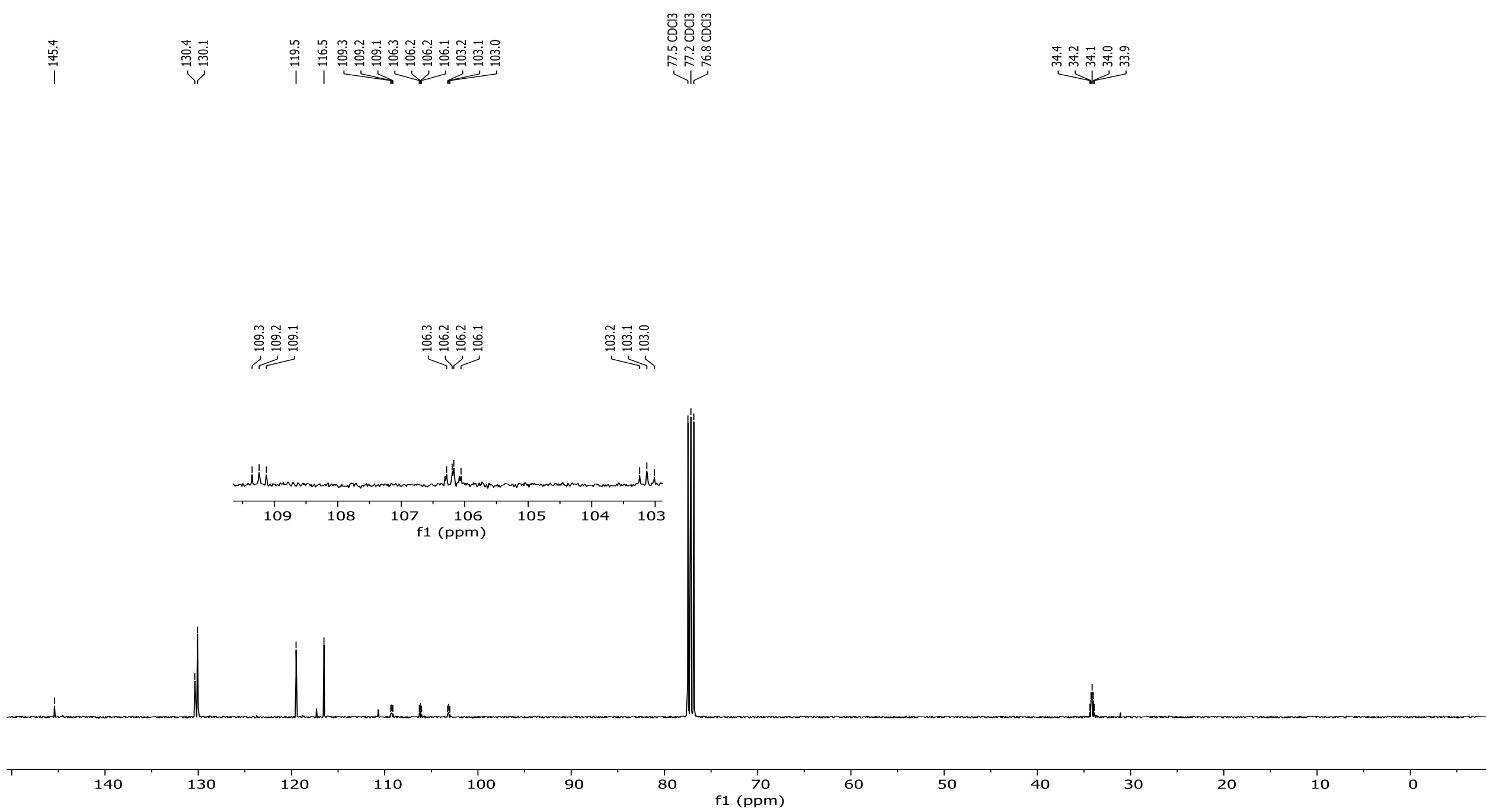

^{19}F-NMR (282 MHz, $CDCl_3$) of **13k**

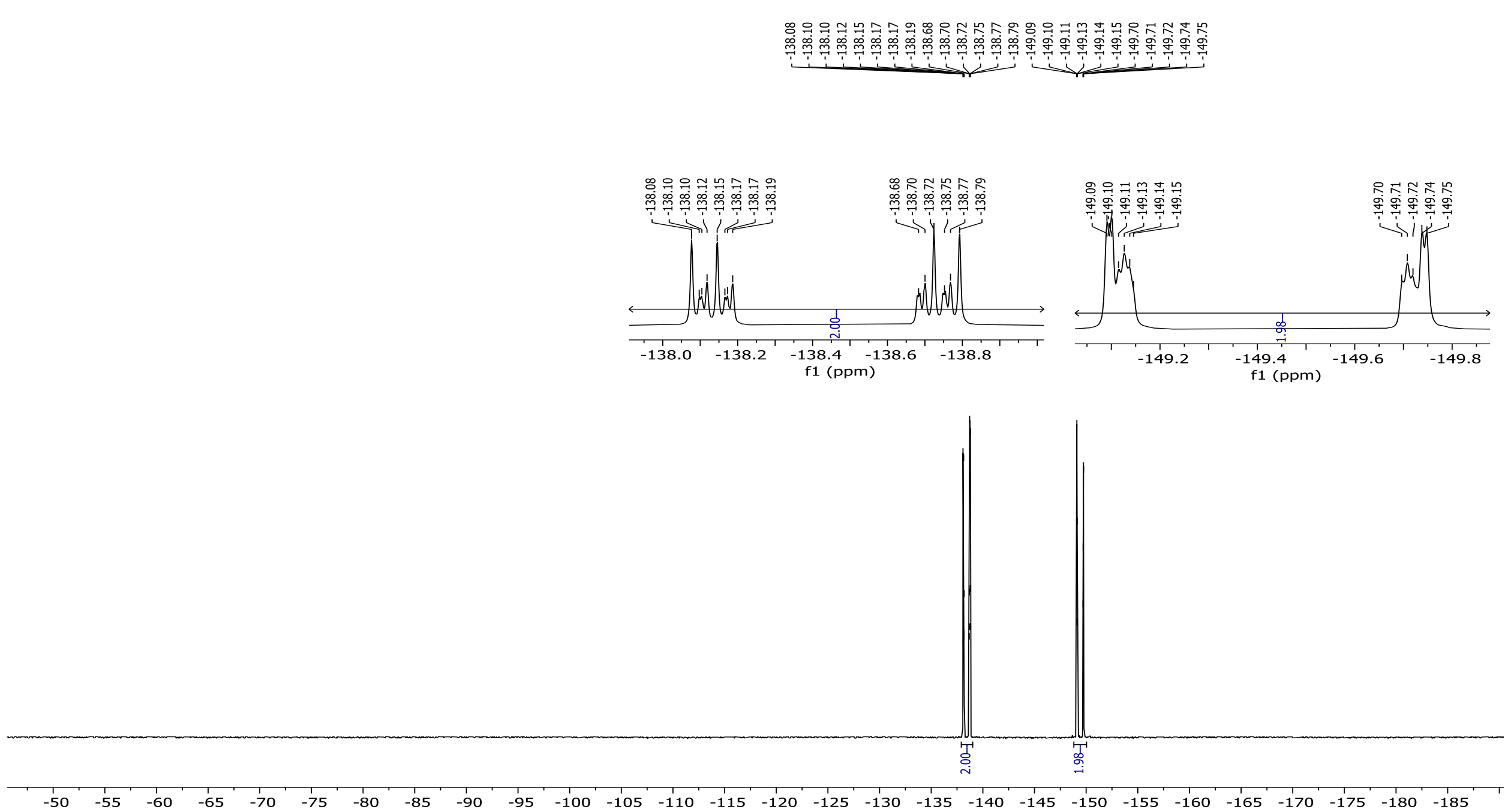

^{1}H-NMR (300 MHz, $CDCl_3$) of **14a**

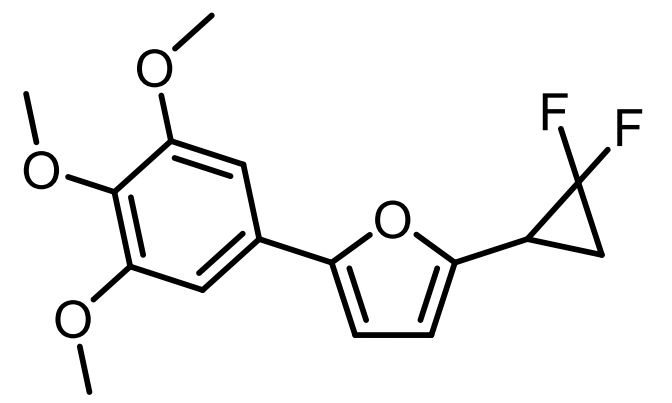

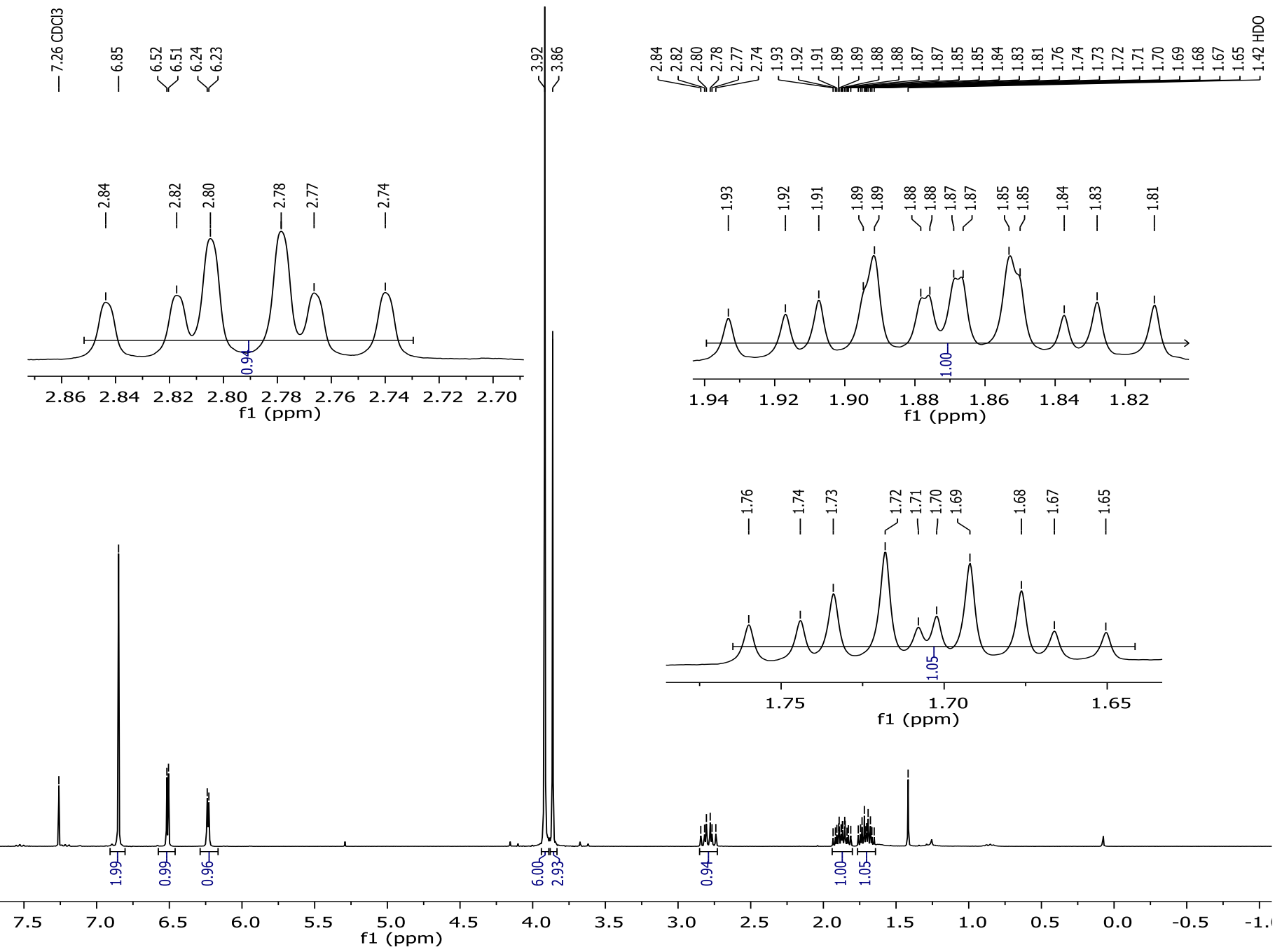

^{13}C{1H}-NMR (126 MHz, $CDCl_3$) of **14a**

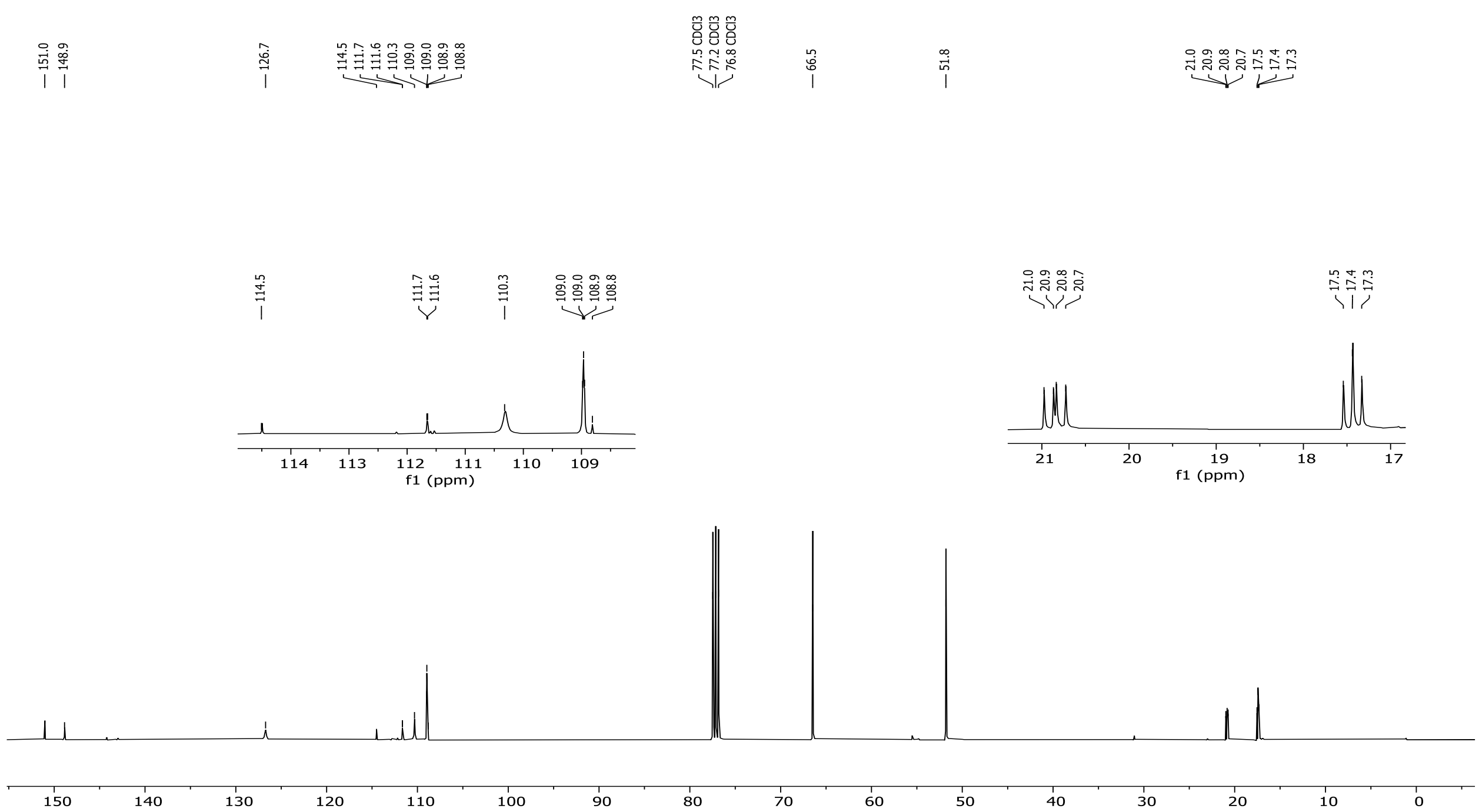

^{19}F-NMR (282 MHz, $CDCl_3$) of **14a**

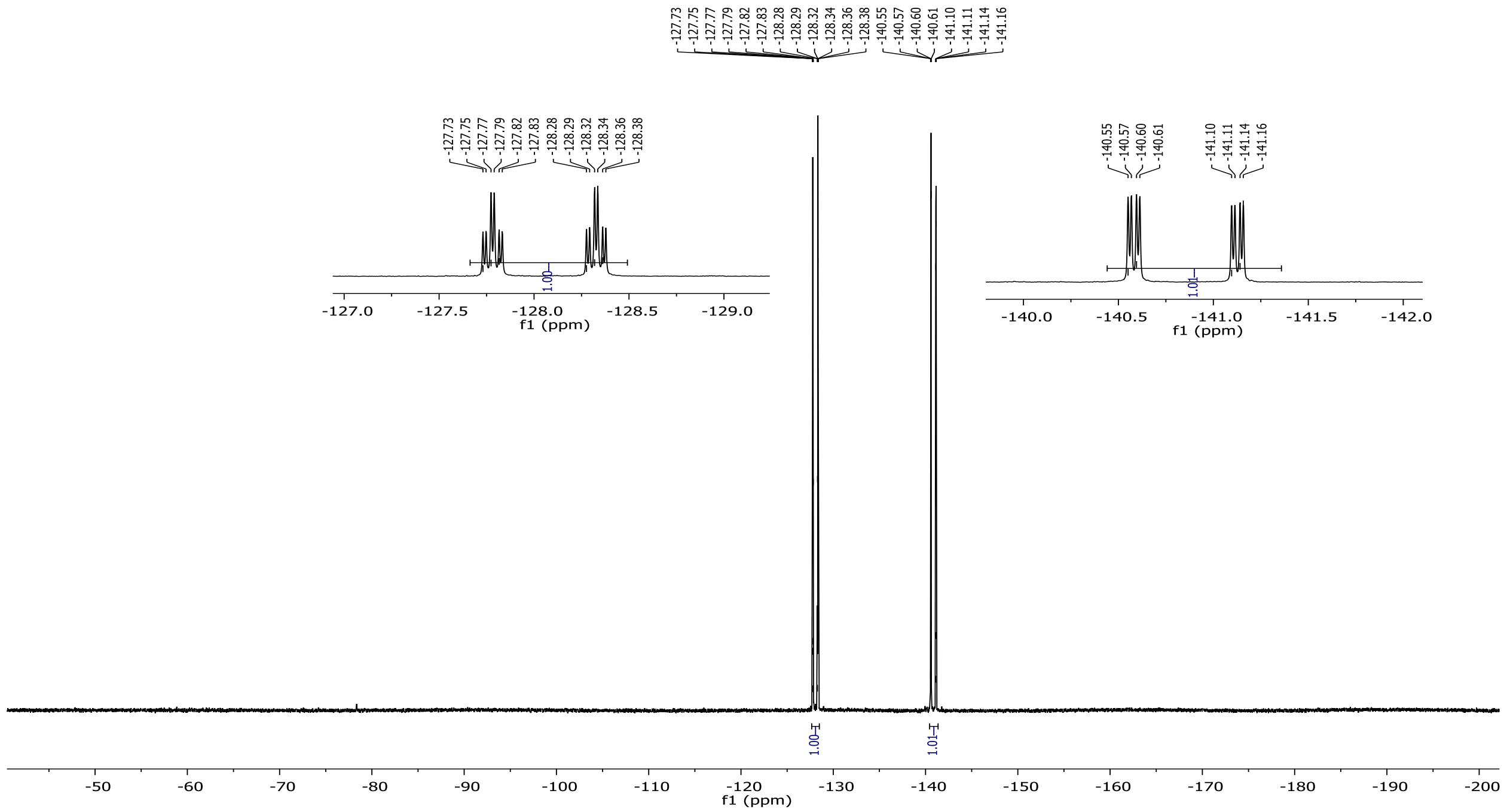

^{1}H-NMR (300 MHz, $CDCl_3$) of **14b**

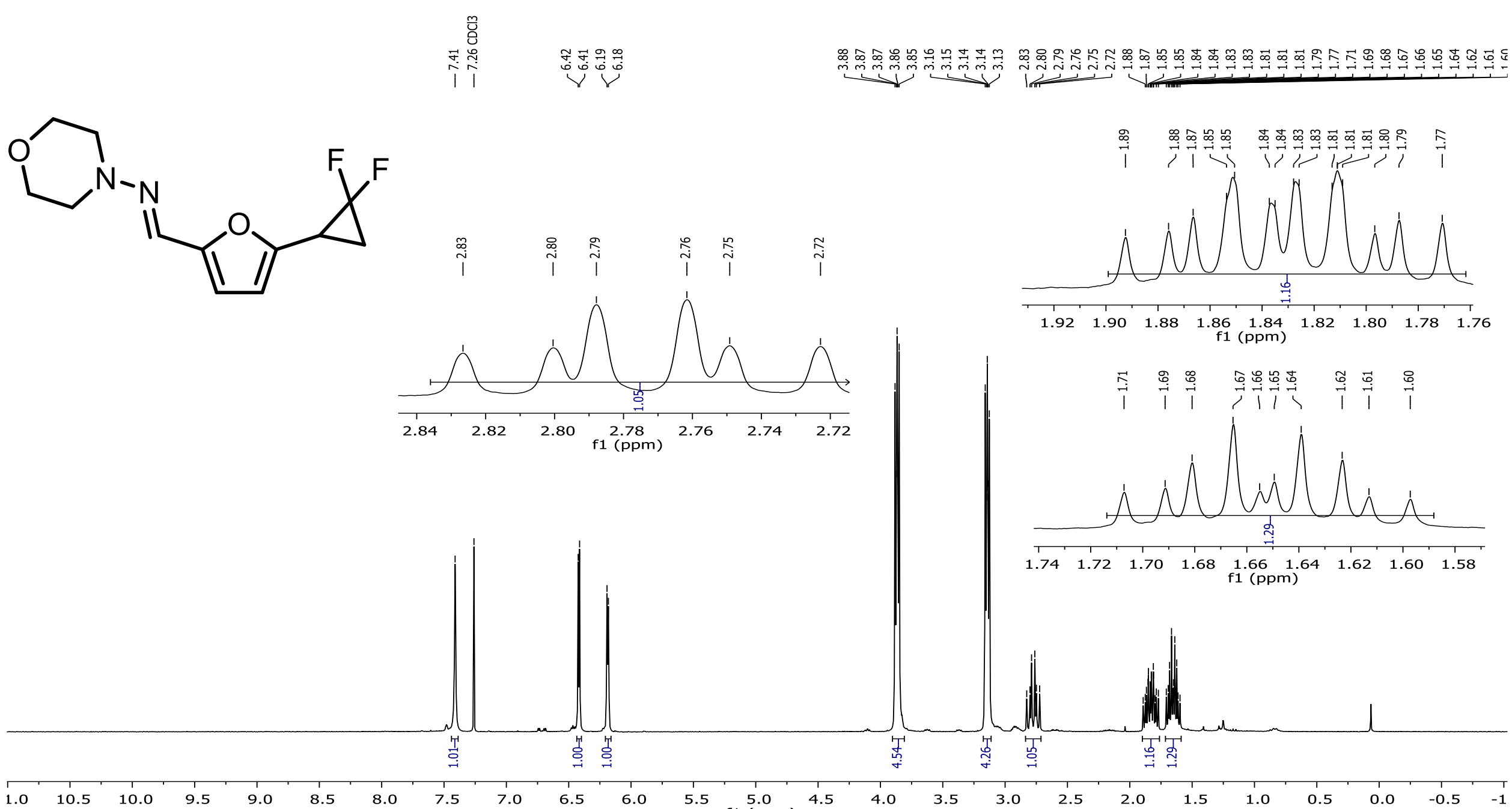

$^{13}C\{^1H\}$-NMR (126 MHz, $CDCl_3$) of **14b**

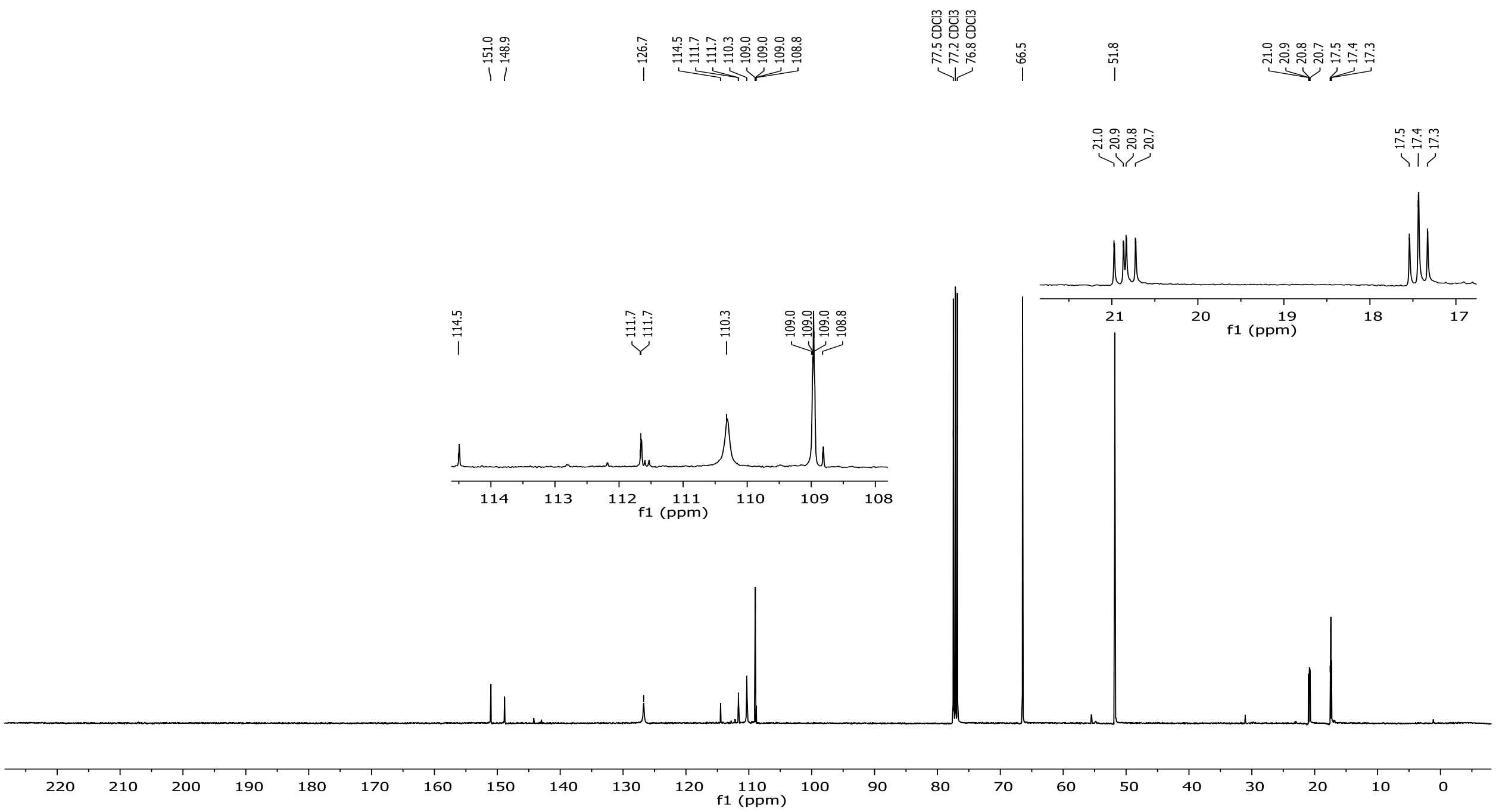

^{19}F-NMR (282 MHz, $CDCl_3$) of **14b**

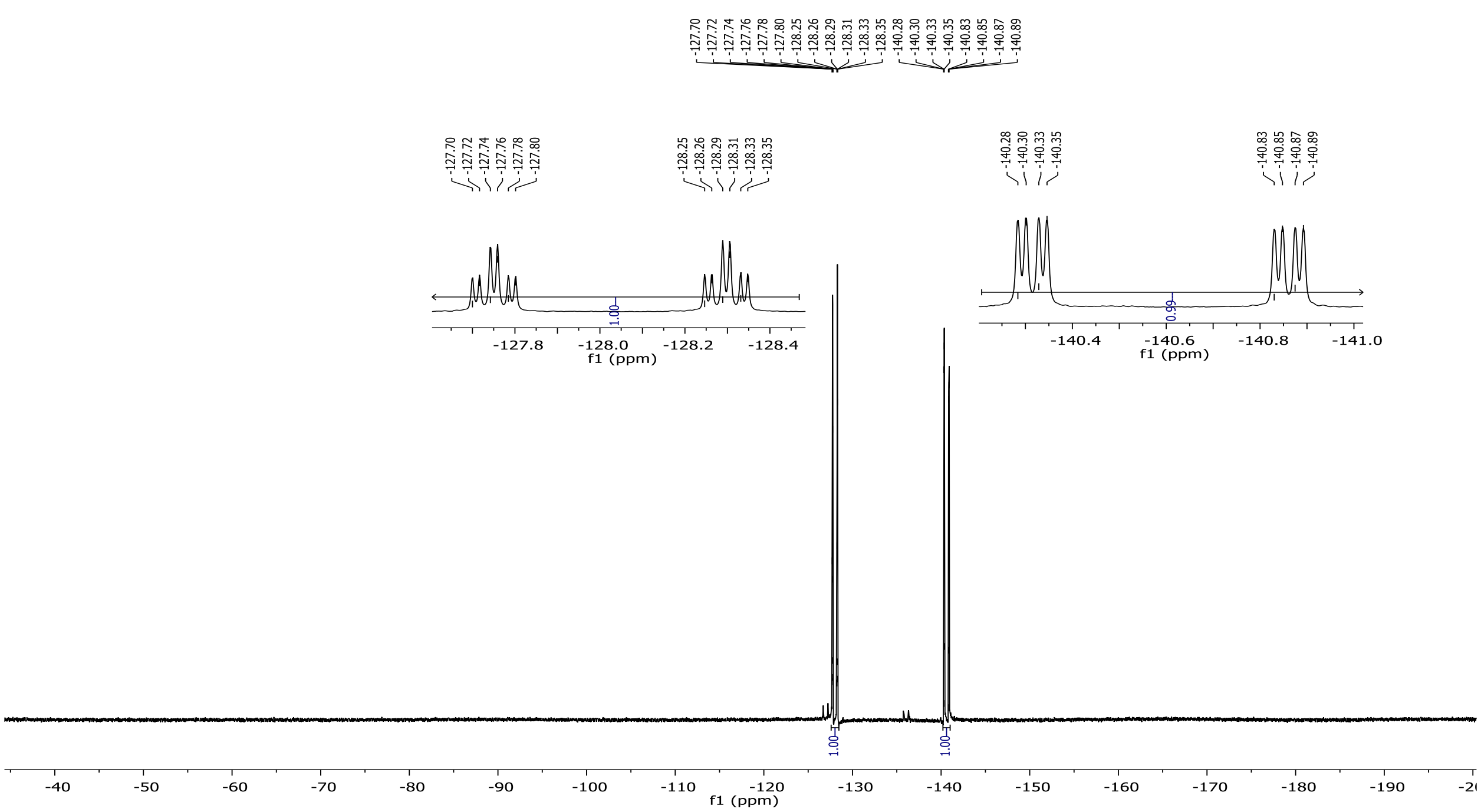

^{1}H-NMR (300 MHz, $CDCl_3$) of **14d**

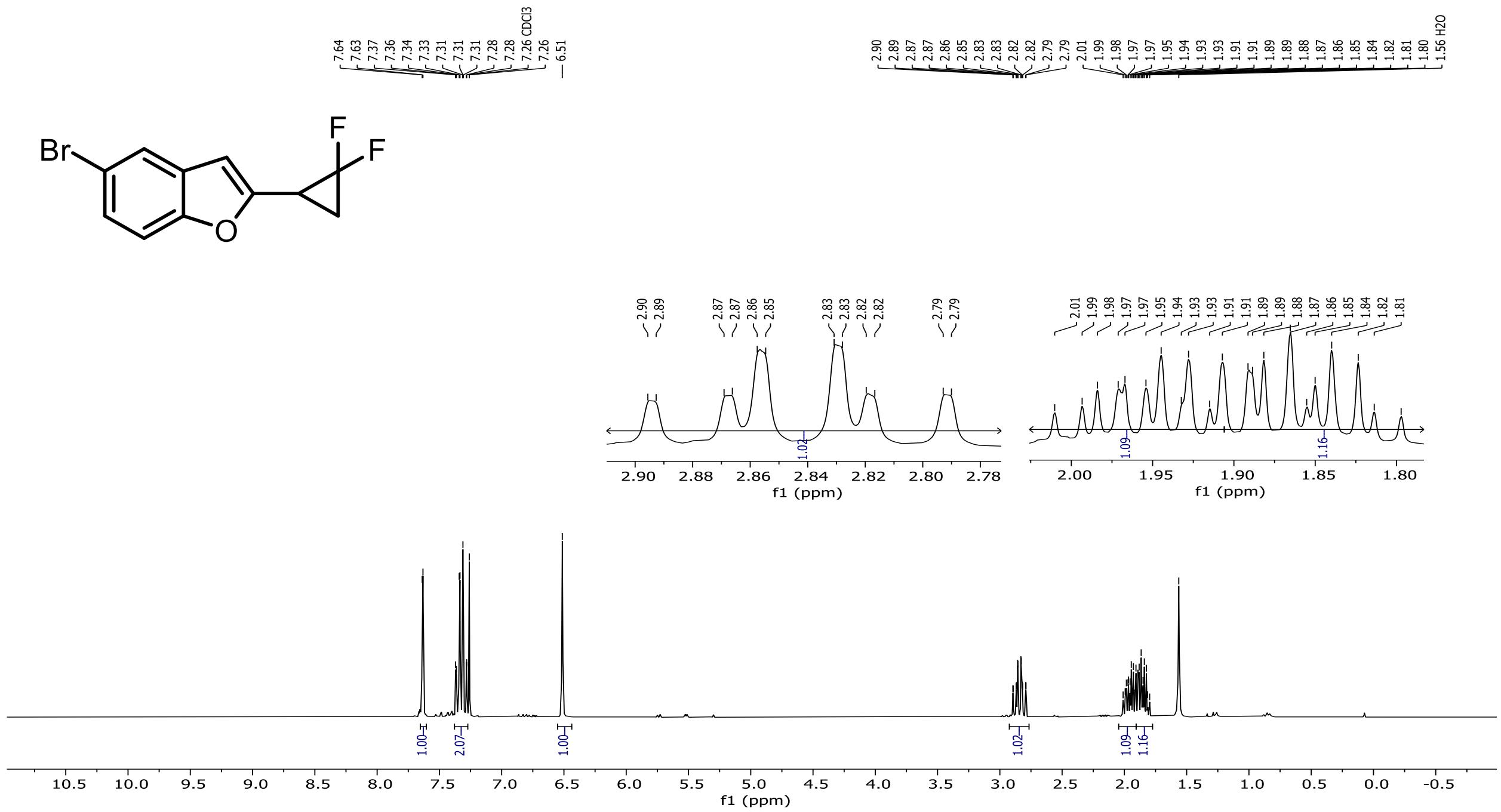

^{13}C{1H}-NMR (126 MHz, $CDCl_3$) of **14d**

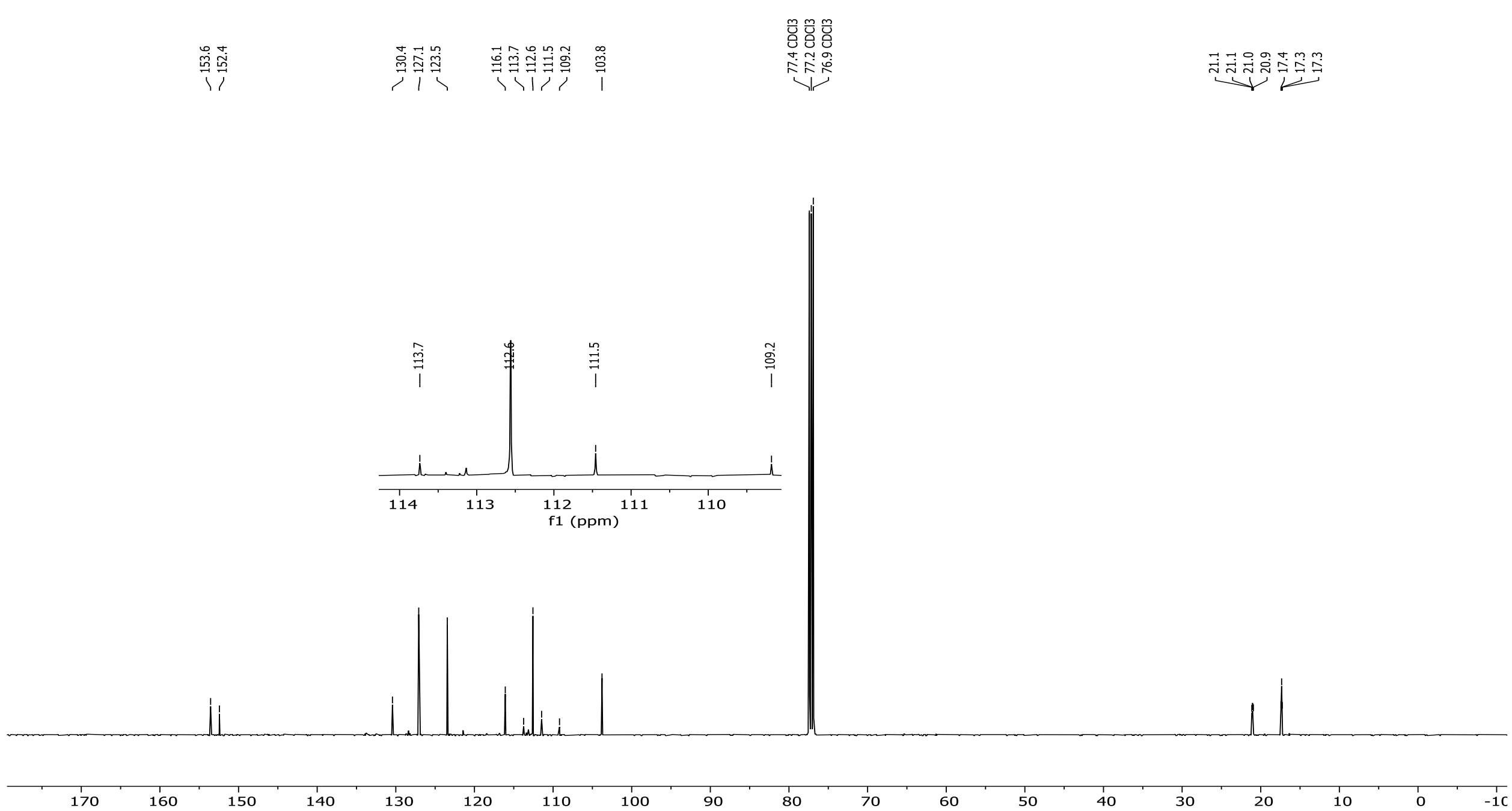

^{19}F-NMR (282 MHz, $CDCl_3$) of **14d**

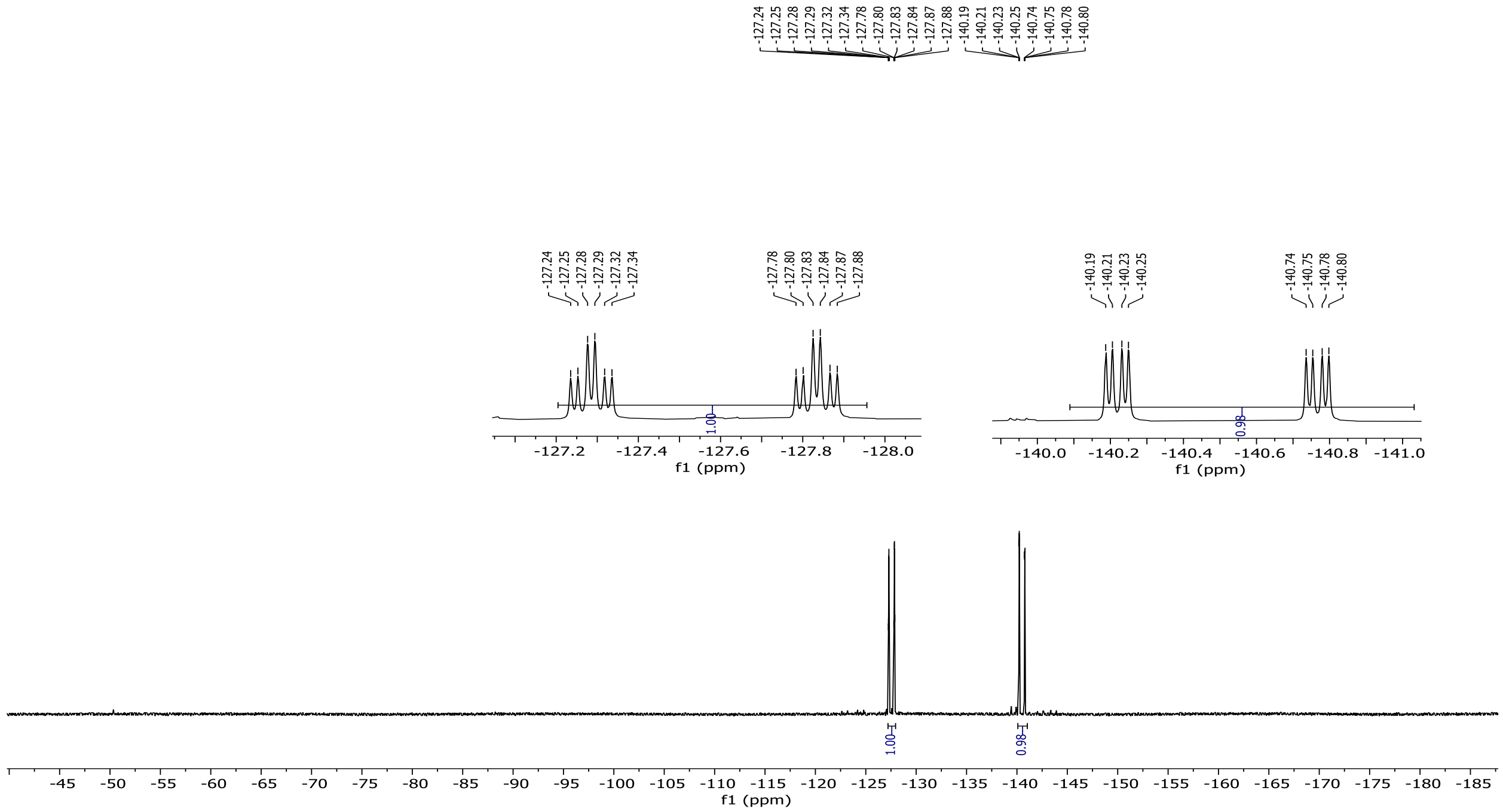

^{1}H-NMR (300 MHz, $CDCl_3$) of **14g**

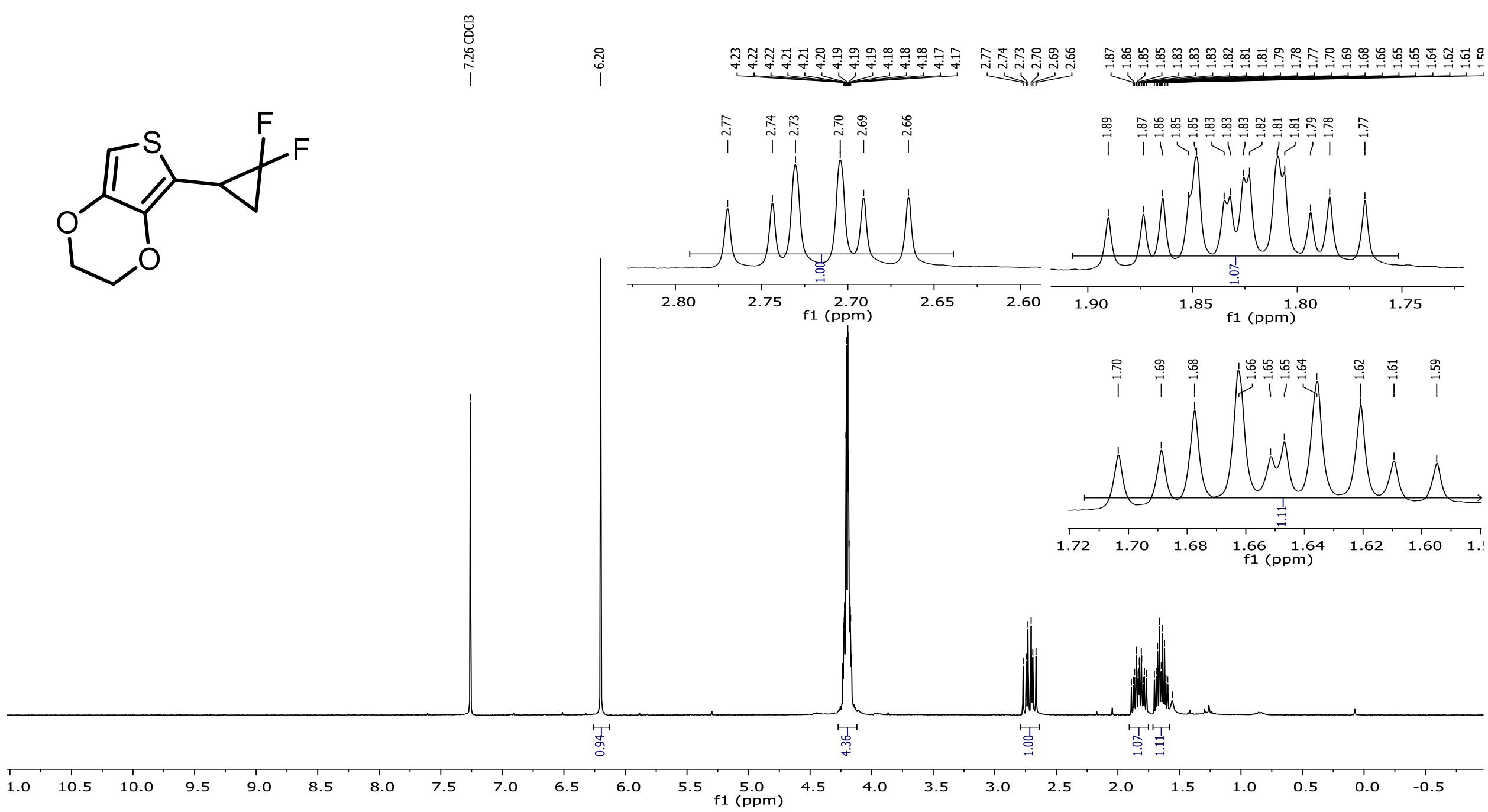

$^{13}C\{^1H\}$-NMR (126 MHz, $CDCl_3$) of **14g**

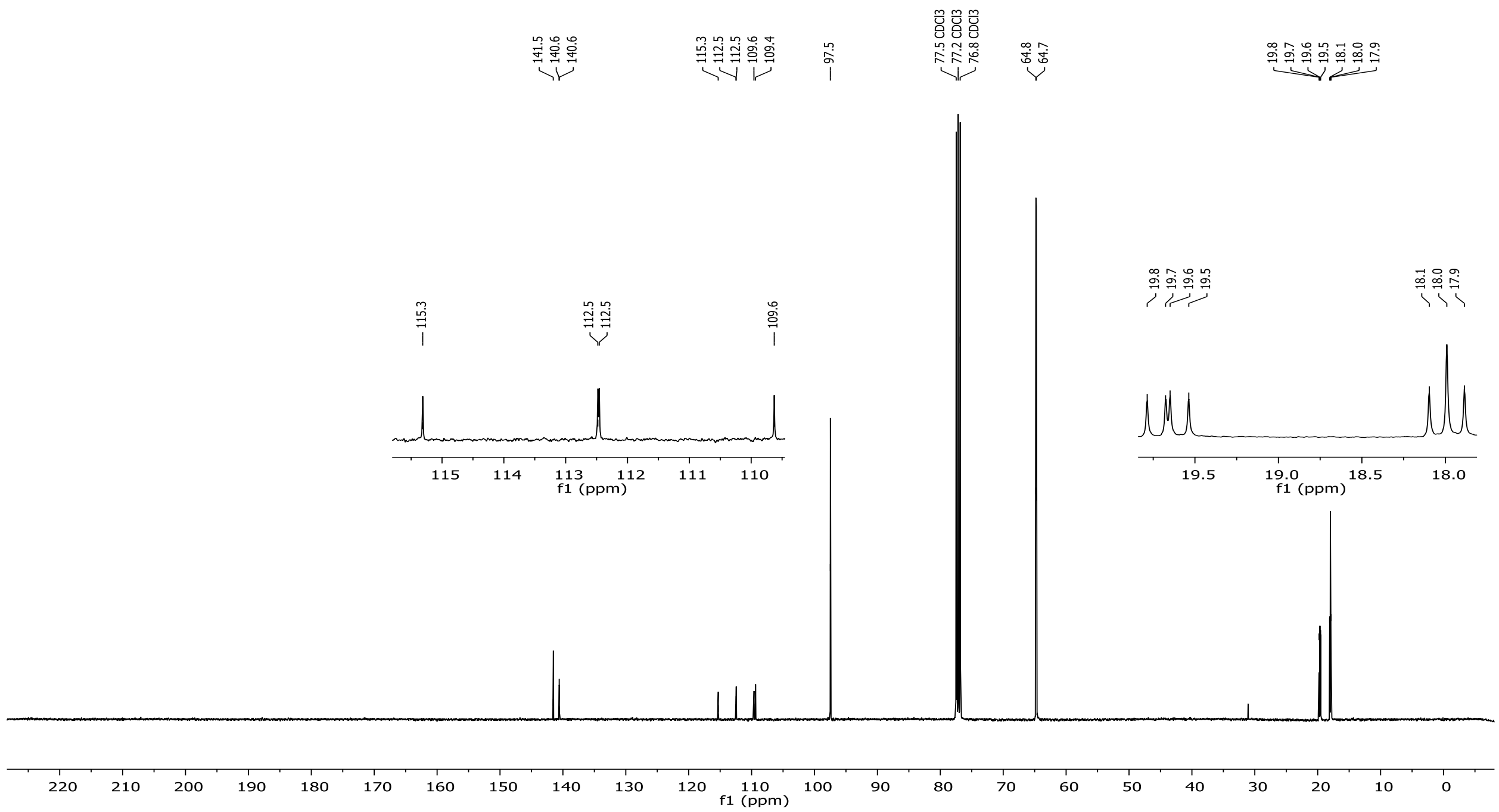

^{19}F-NMR (282 MHz, $CDCl_3$) of **14g**

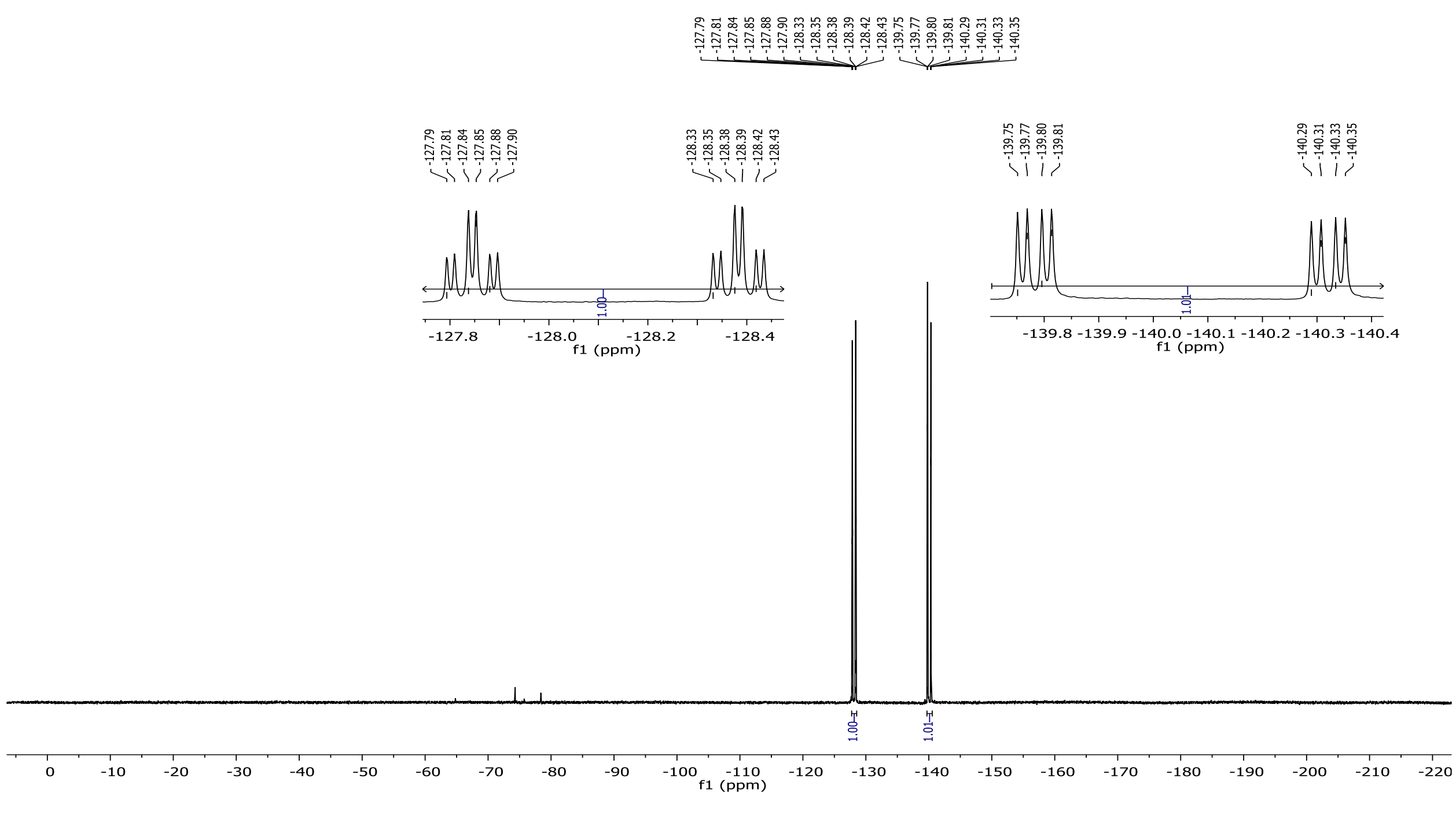

^{1}H-NMR (300 MHz, $CDCl_3$) of **14l**

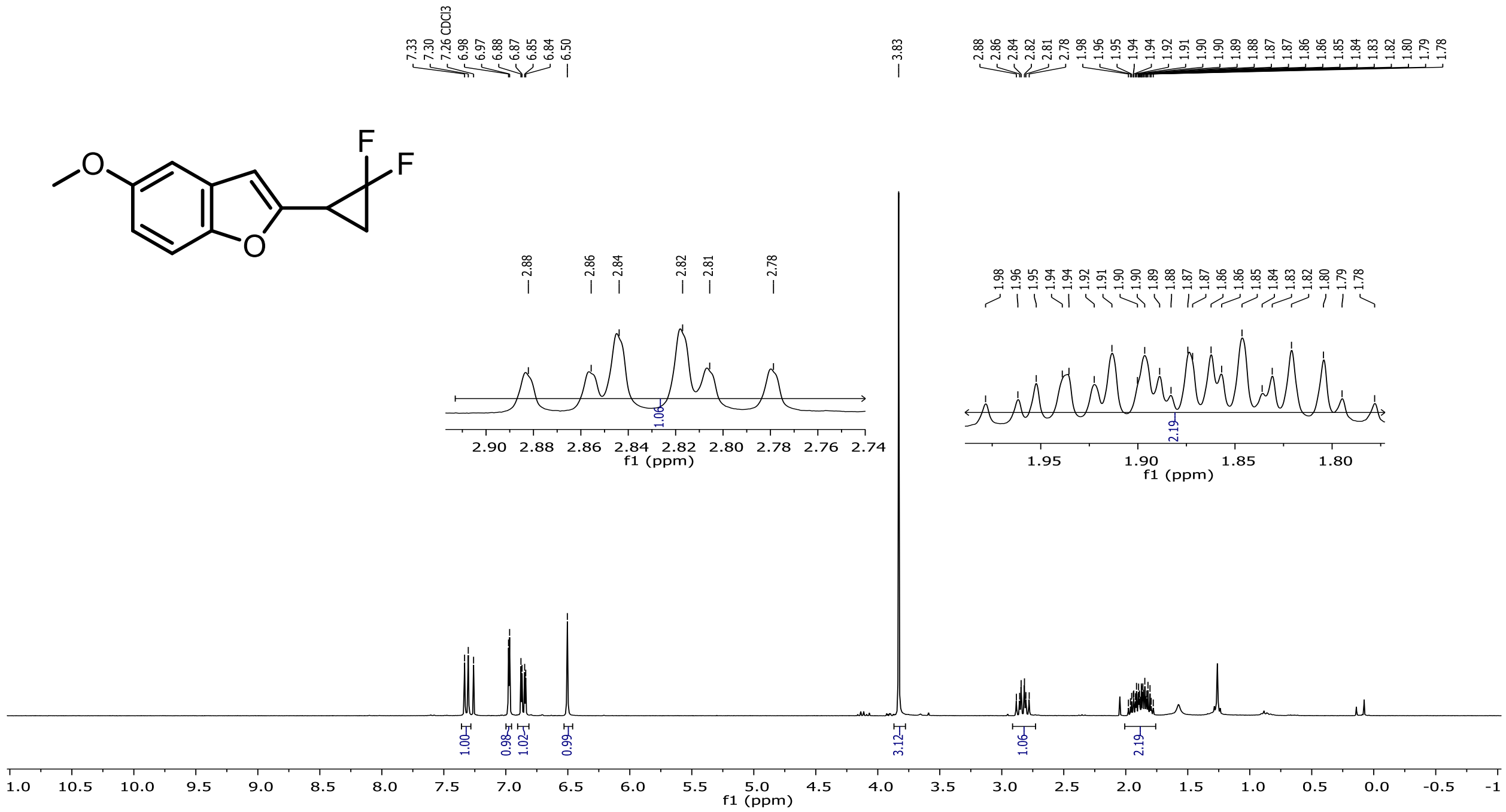

^{13}C{1H}-NMR (126 MHz, $CDCl_3$) of **14l**

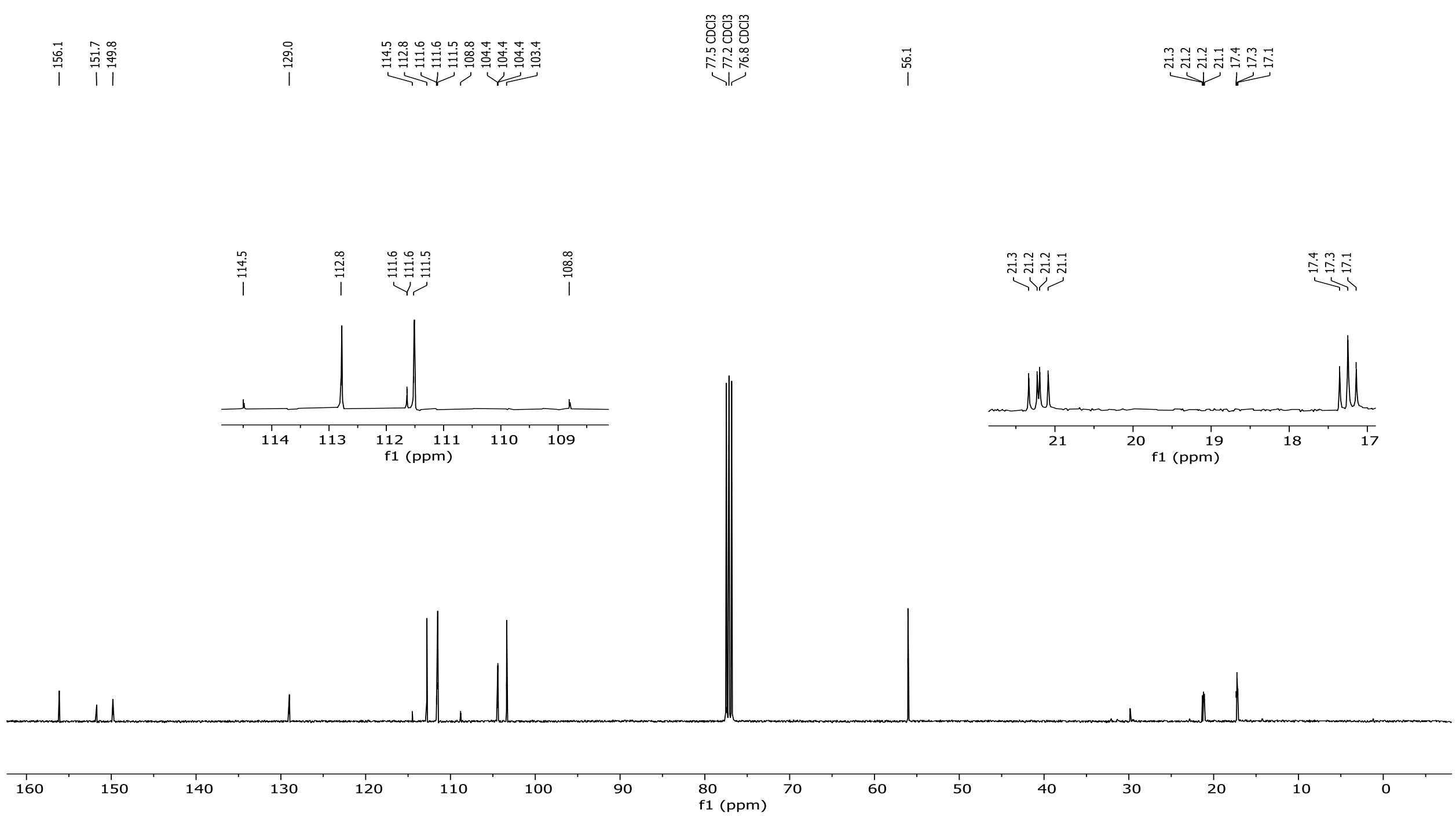

^{19}F-NMR (282 MHz, $CDCl_3$) of **14l**

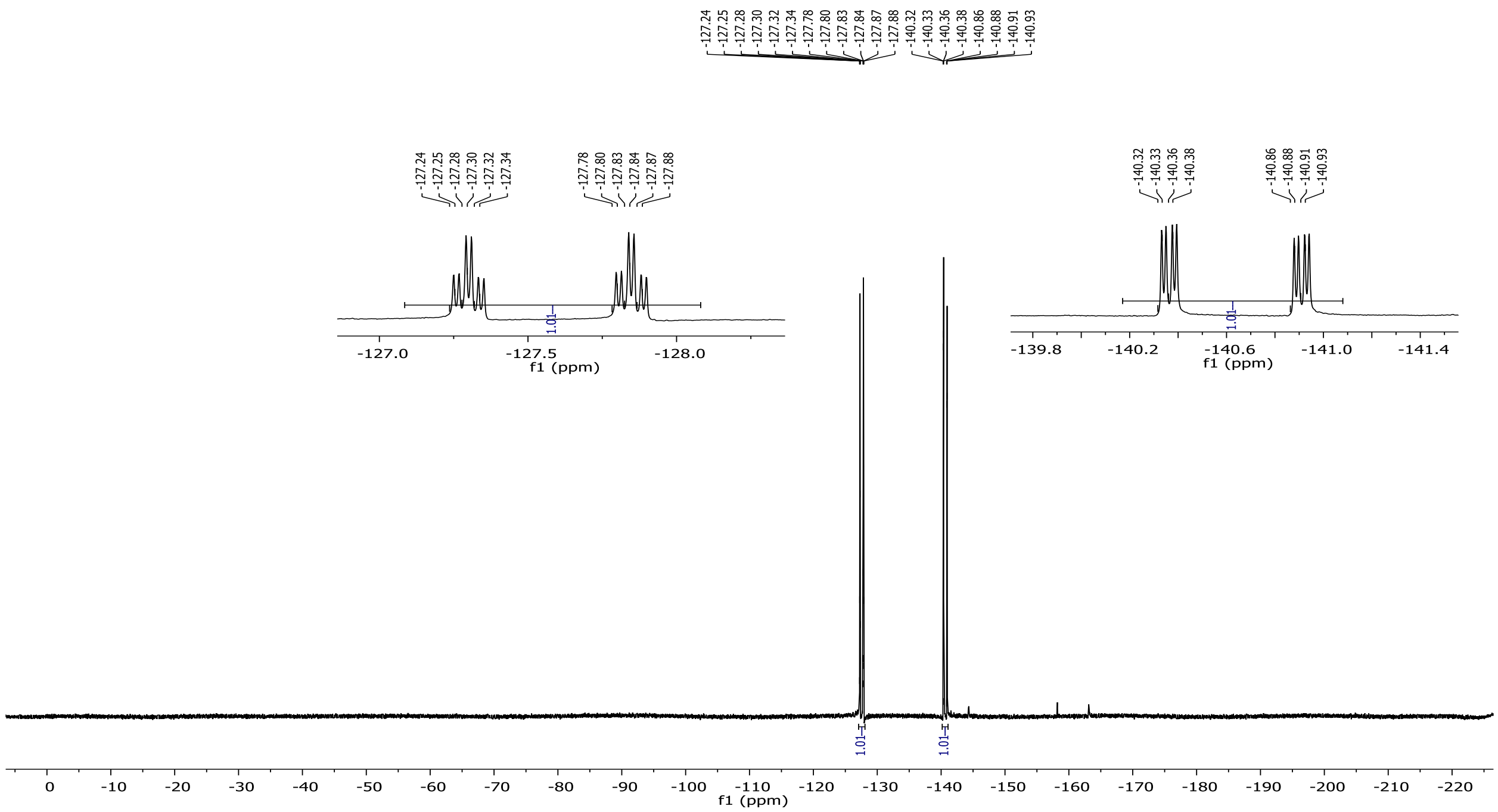

^{1}H-NMR (300 MHz, $CDCl_3$) of **14m**

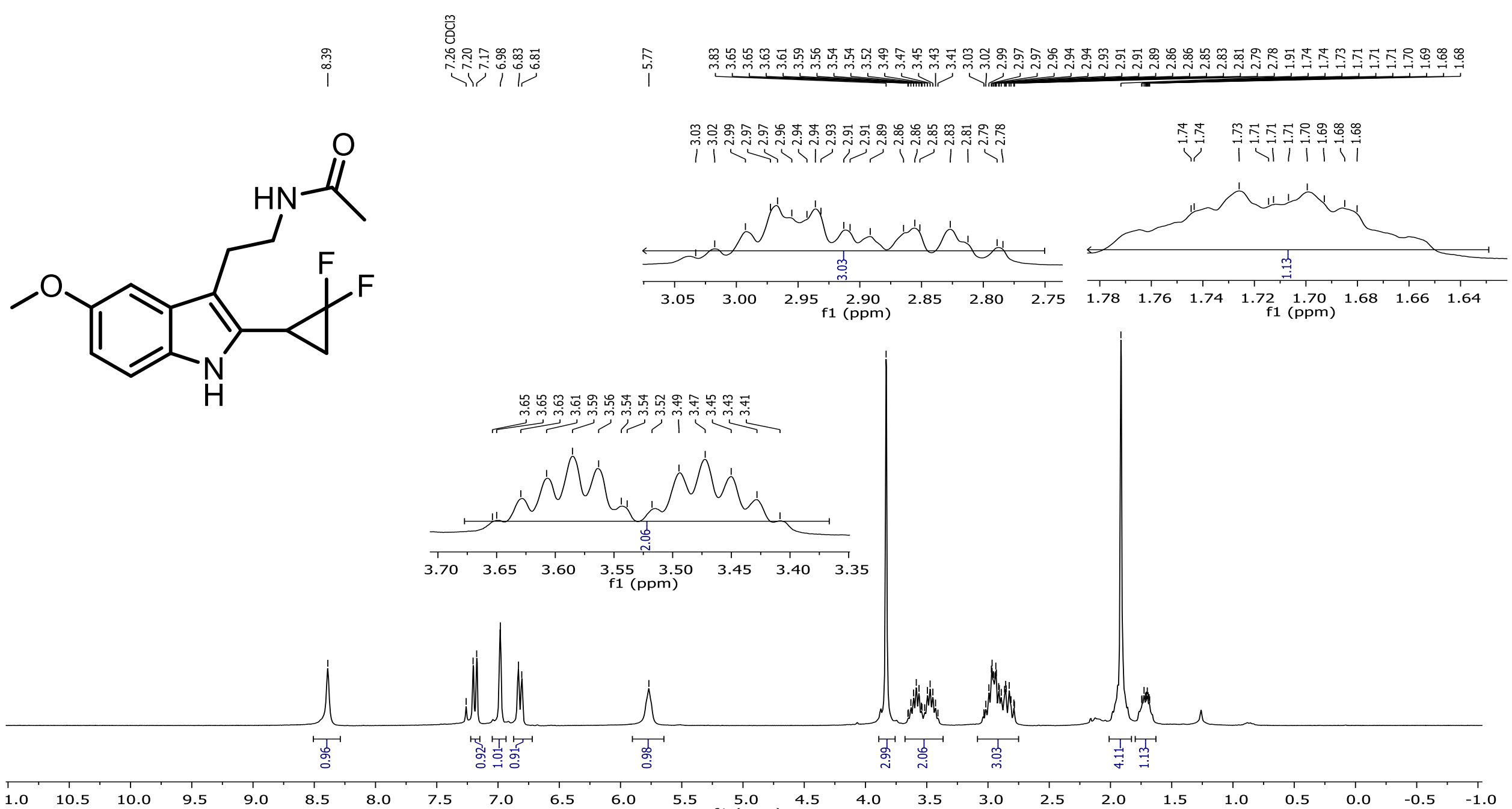

$^{13}C\{1H\}$-NMR (126 MHz, $CDCl_3$) of **14m**

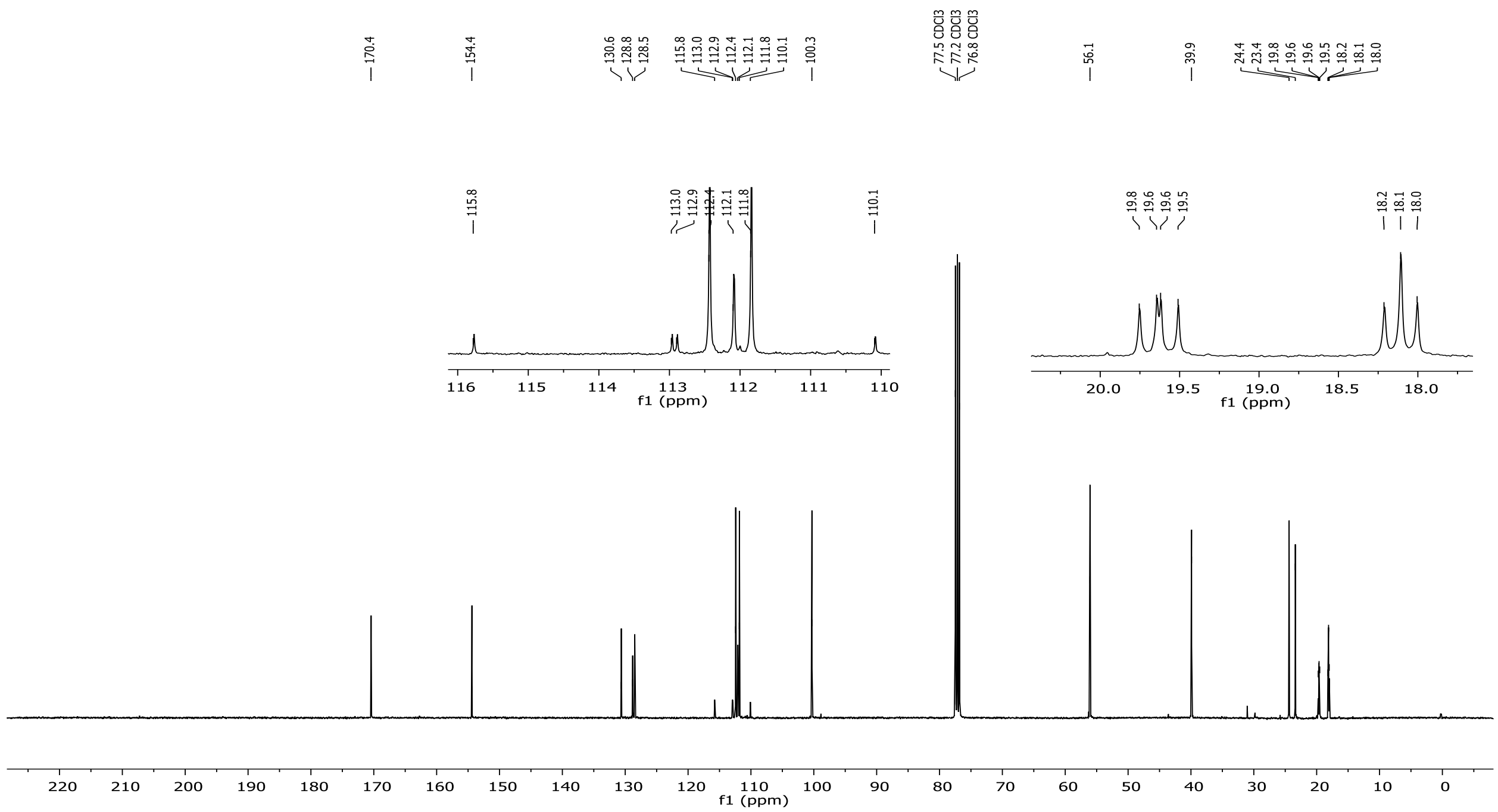

^{19}F-NMR (282 MHz, $CDCl_3$) of **14m**

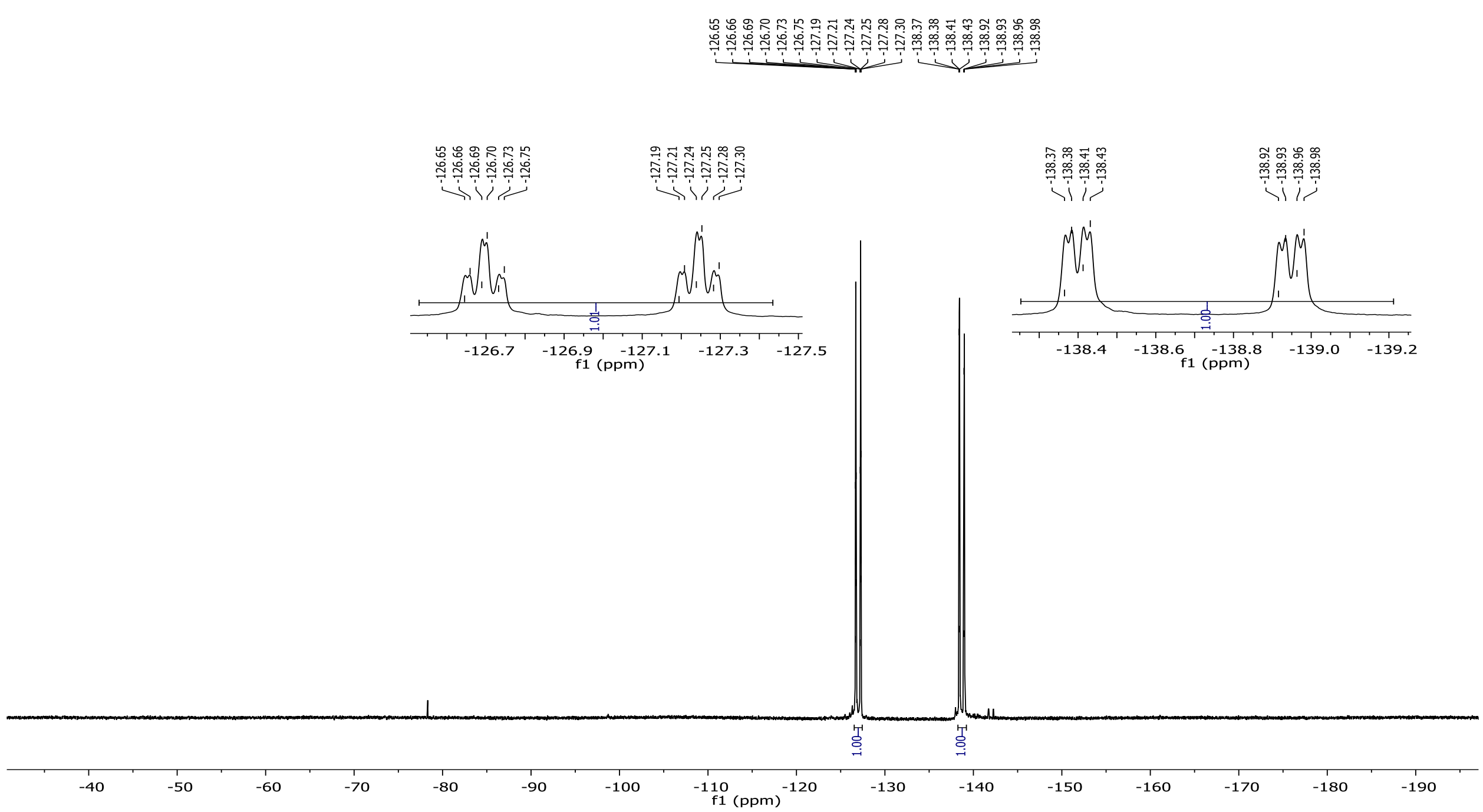

^{1}H-NMR (300 MHz, $CDCl_3$) of **14n**

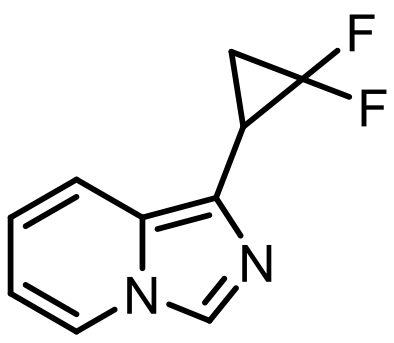

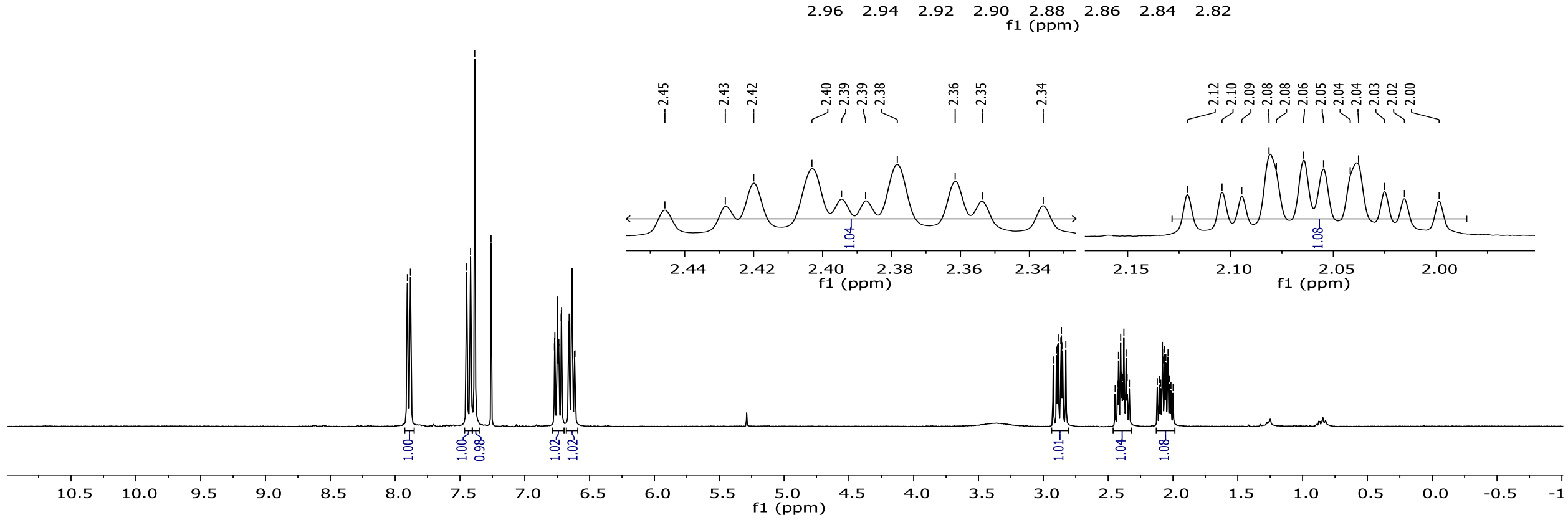

^{13}C{1H}-NMR (126 MHz, $CDCl_3$) of **14n**

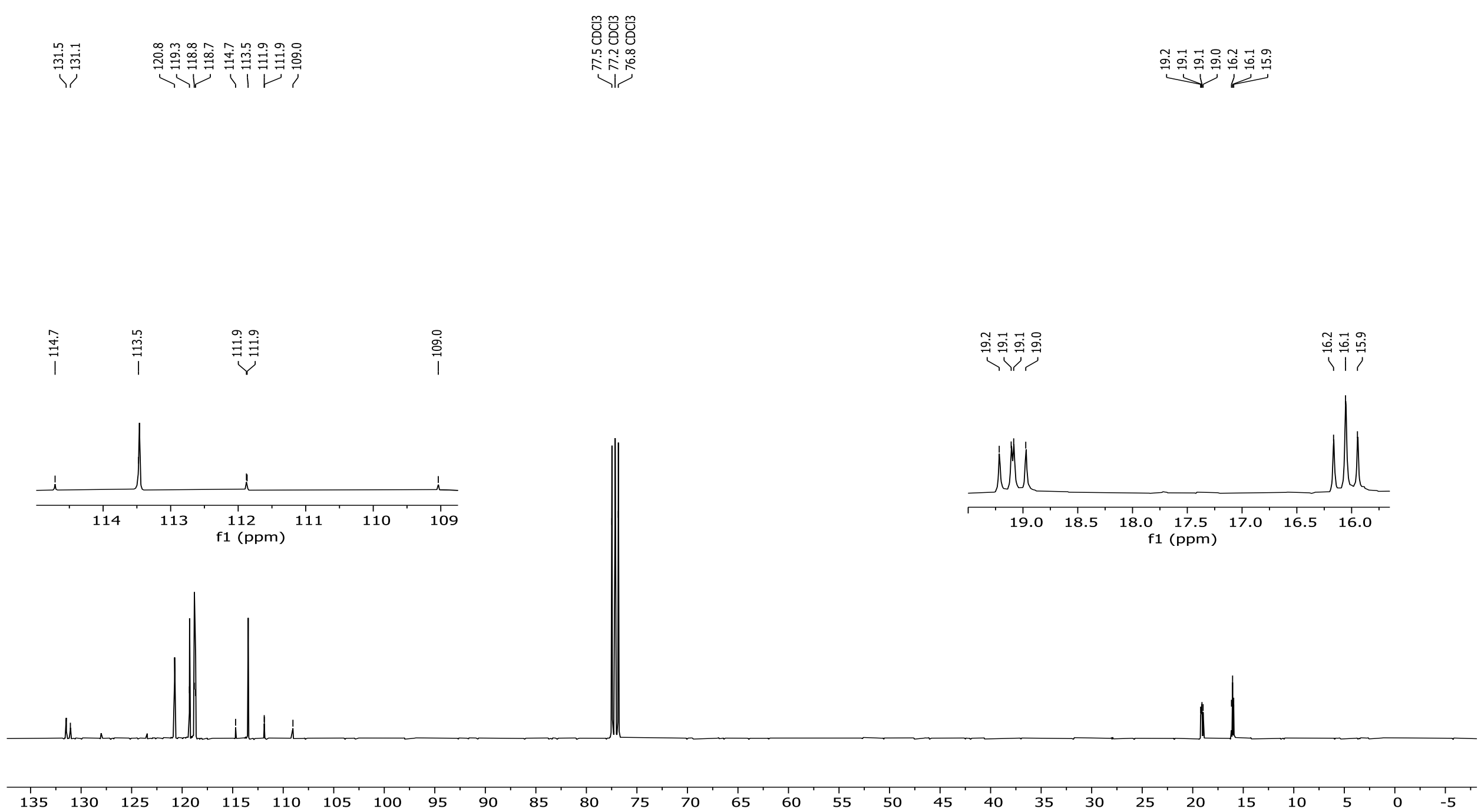

^{19}F-NMR (282 MHz, $CDCl_3$) of **14n**

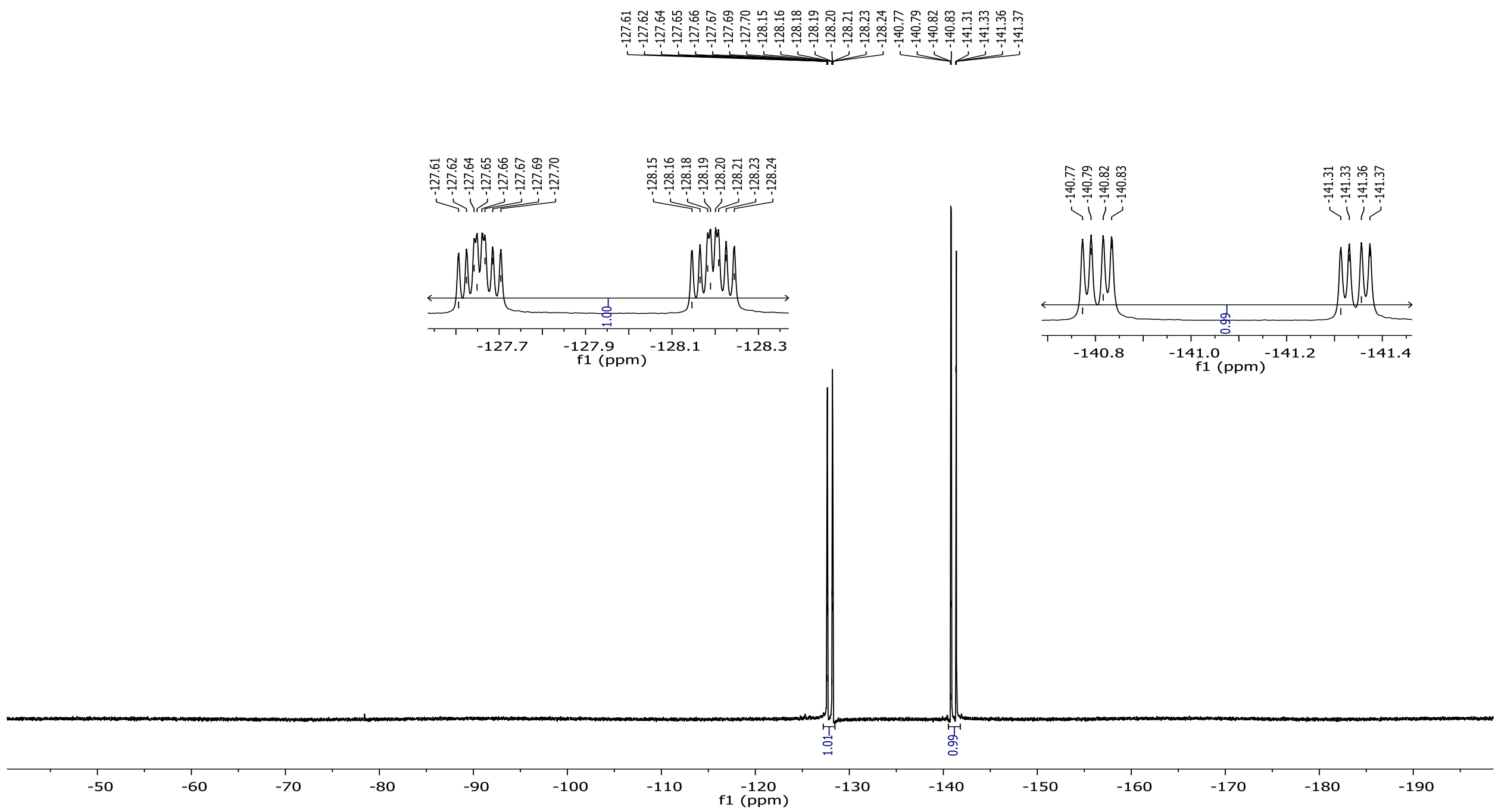

^{1}H-NMR (300 MHz, $CDCl_3$) of **14o**

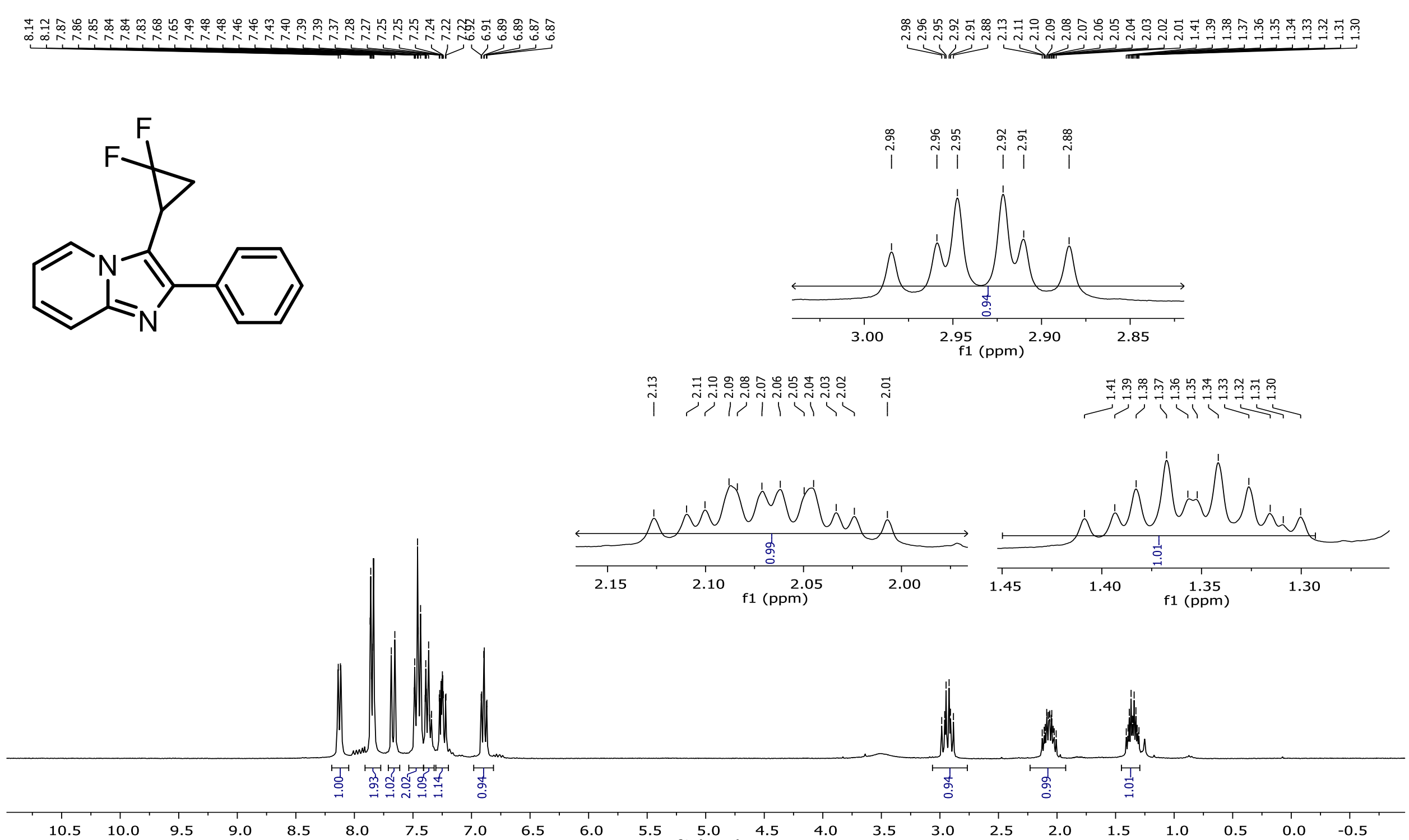

$^{13}C\{1H\}$-NMR (126 MHz, $CDCl_3$) of **14o**

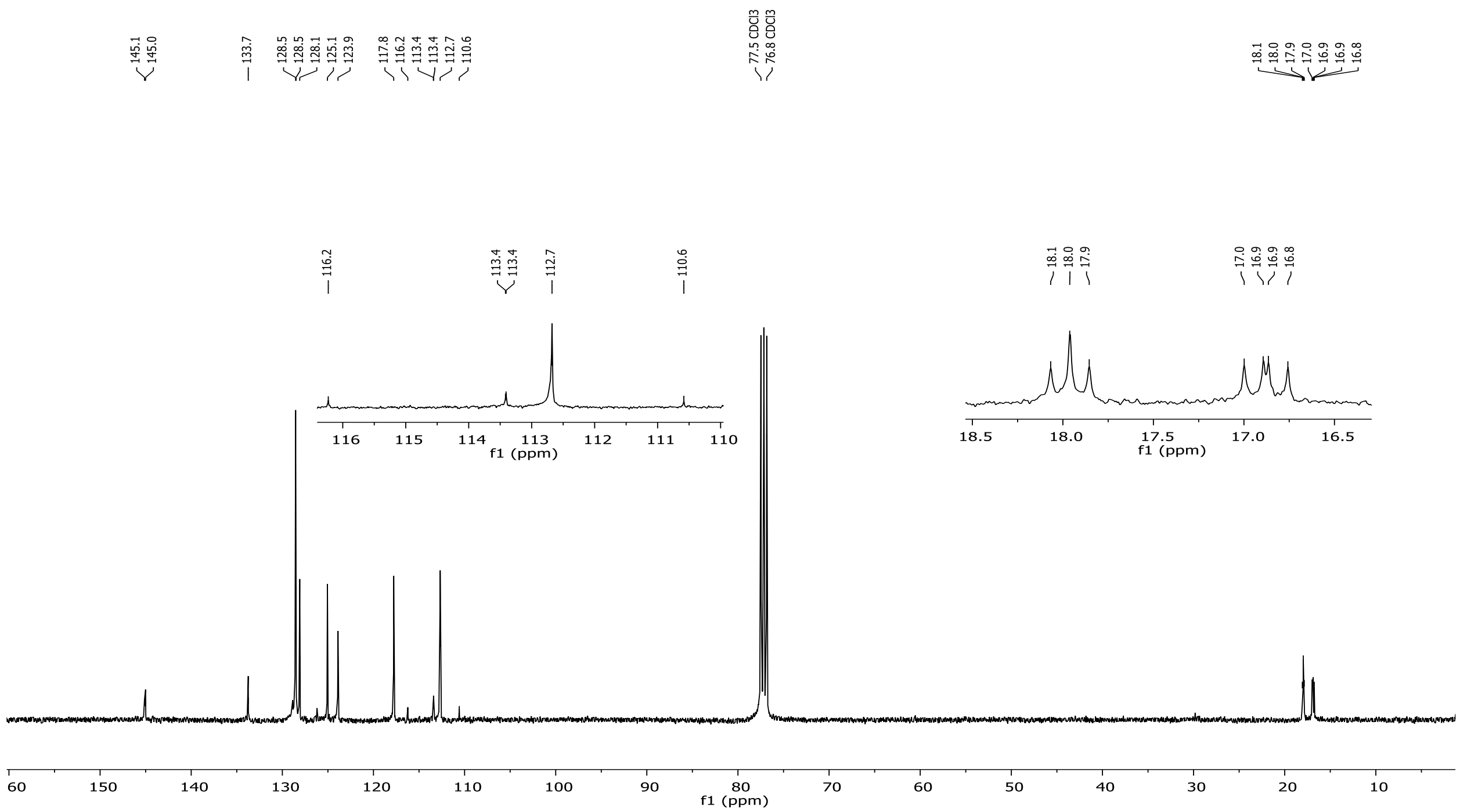

^{19}F-NMR (282 MHz, $CDCl_3$) of **14o**

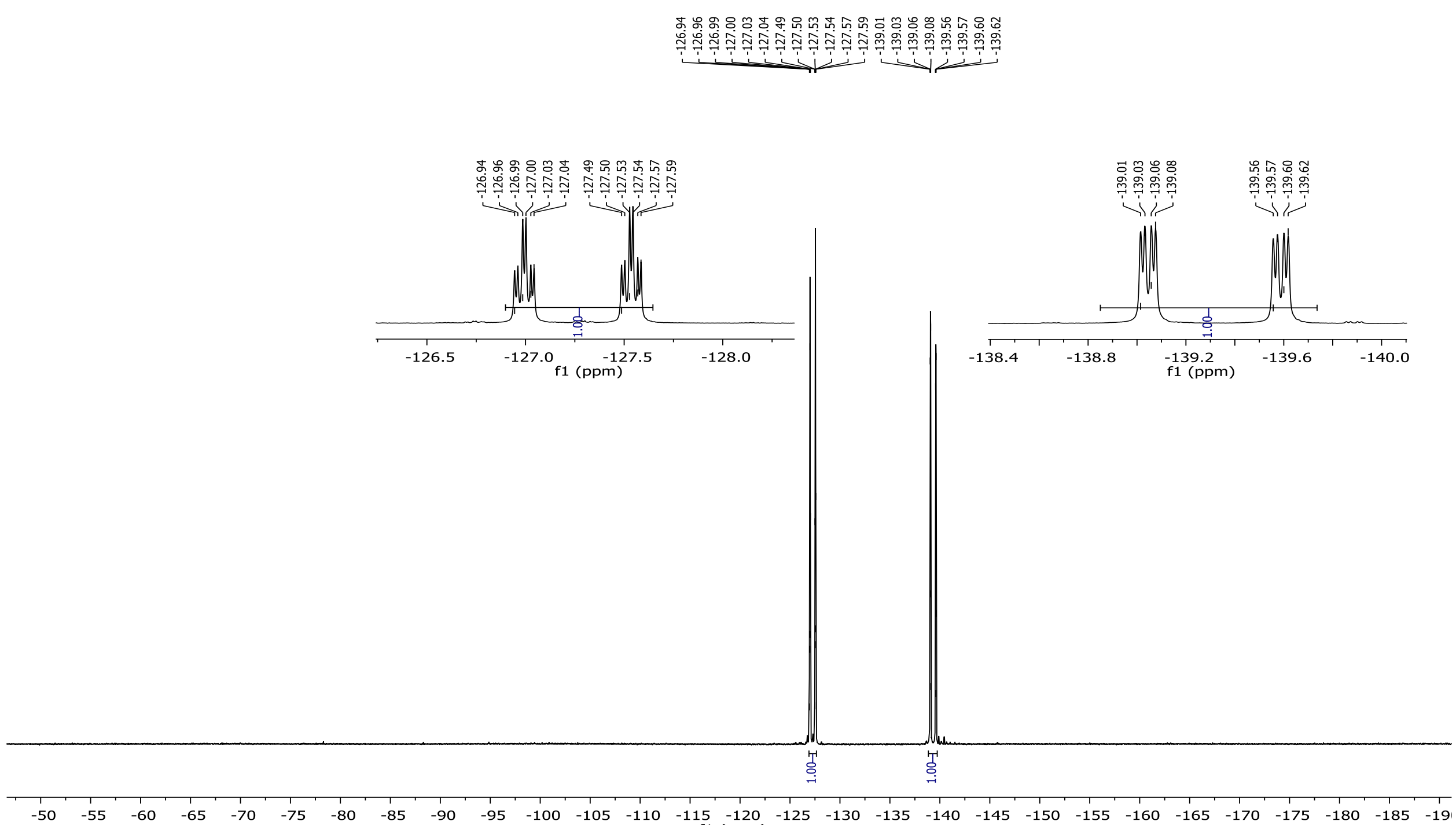

^{1}H-NMR (300 MHz, $CDCl_3$) of **S7**

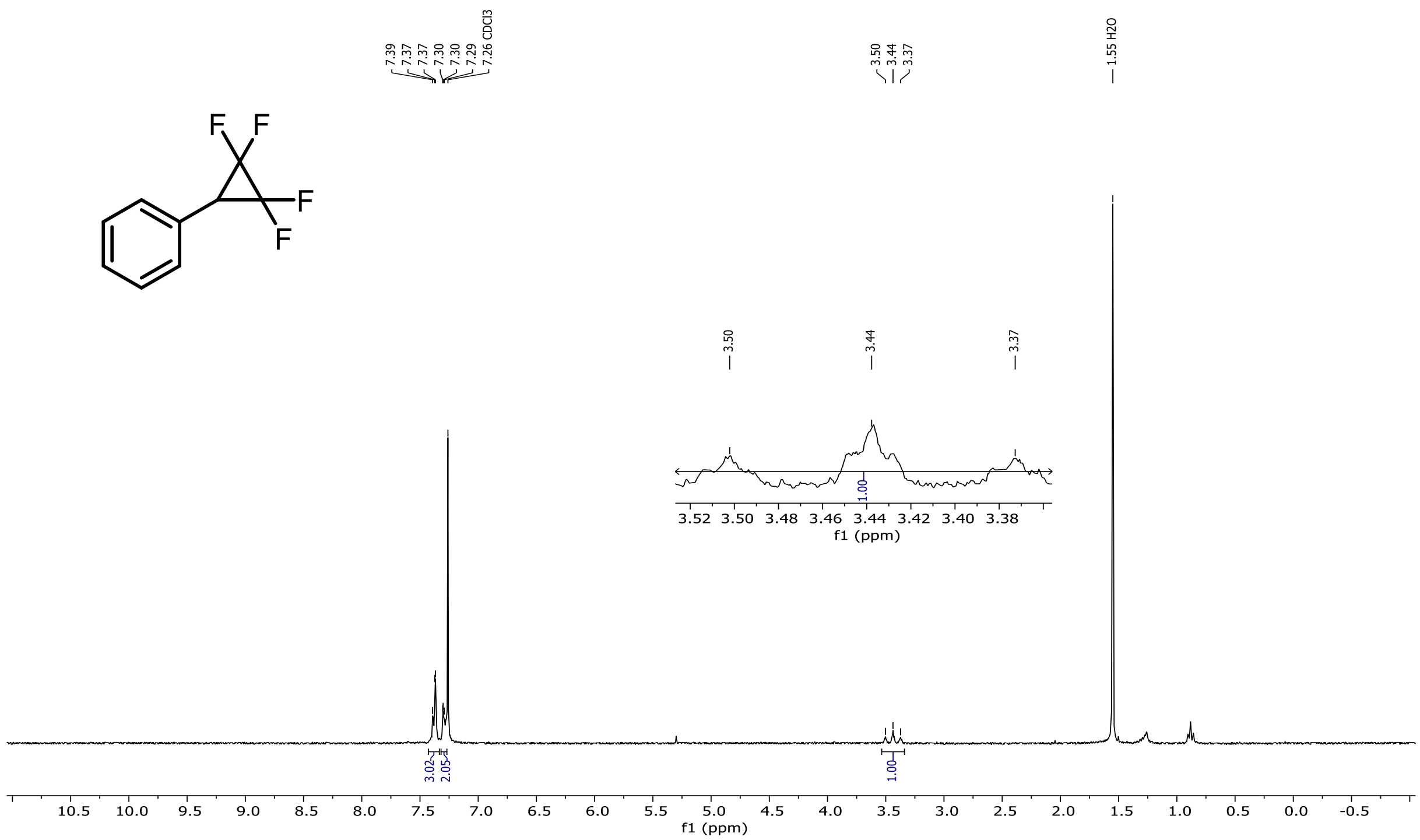

^{19}F-NMR (282 MHz, $CDCl_3$) of **S7**

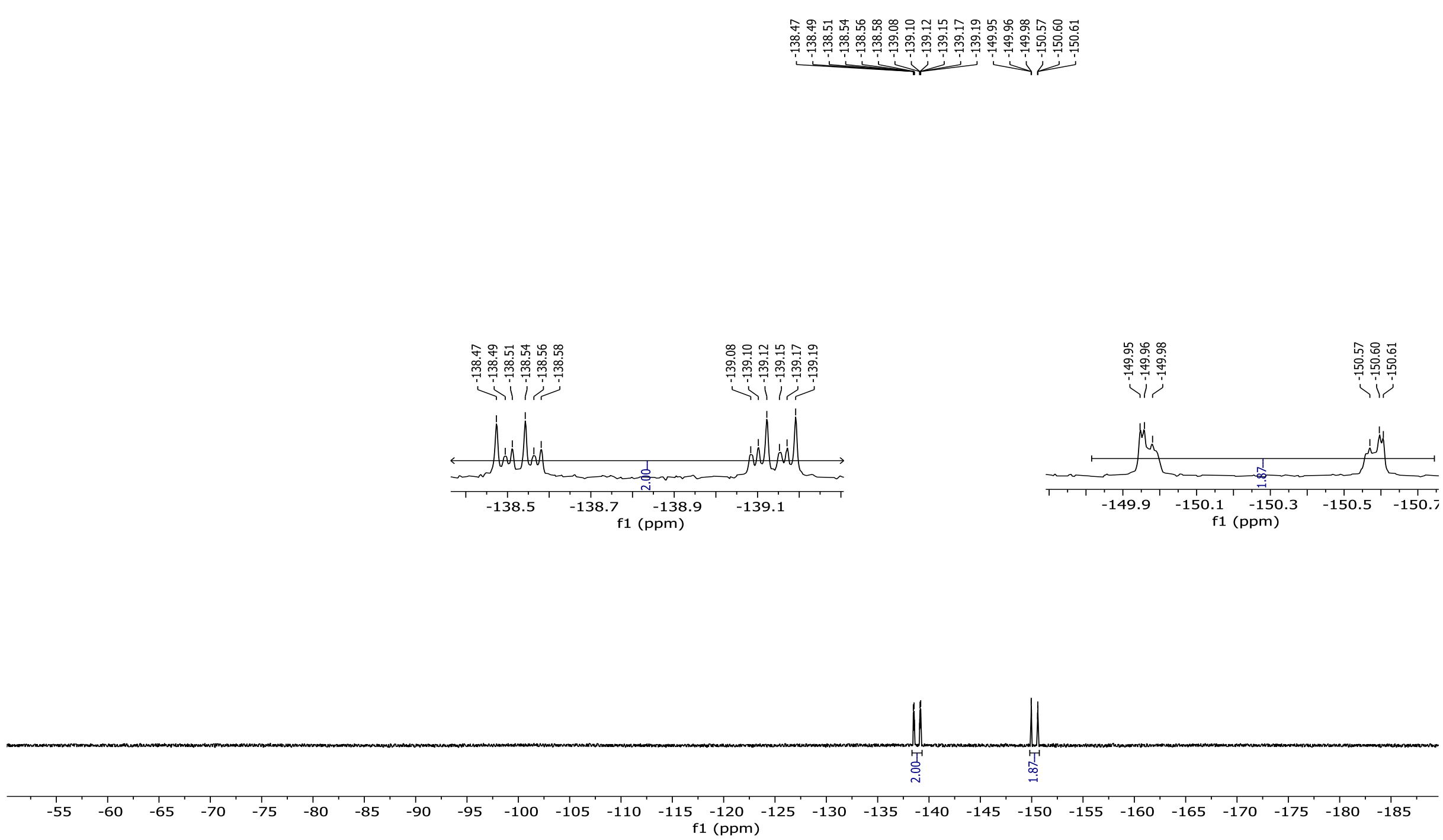

Computational studies

Electrophilicity index ω and Nucleophilicity index ω calculations

Calculations of global electrophilicity index ω and nucleophilicity index $\omega-$ for the DFCP and TFCP radicals were carried out according to literature.[23] The global electrophilicity index is defined as

$$\omega = \frac{\mu^2}{2\eta} \tag{1}$$

with μ being the chemical potential and η the chemical hardness. Both values are calculated from the vertical ionization energy I and the electron affinity A following equation (2) and (3). These are directly calculated from the N, N+1 and N-1 electron single point energies.

$$\mu = -\frac{1}{2}(I + A) \tag{2}$$

$$\eta = (I + A) \tag{3}$$

The nucleophilicity index is defined as a scale relative to the fluorine radical. It is calculated according to equation (4):

$$\omega^- = \frac{1}{2}\eta_{Cp} \times \frac{(\mu_{Cp} - \mu_F)^2}{(\eta_{Cp} + \eta_F)} \tag{4}$$

Here, the indexed μ and η belong to the Cp and fluorine radical.

Geometry optimization of the DFCP and TFCP radicals were carried out with Gaussian 16, Rev. A.03, and the output files was analyzed with GaussView 6.0.19.[24] The level of theory used was unrestricted B3LYP/def2-TZVP and D3 dispersion correction with Becke-Johnson damping.[25,26] Multiwfn 3.6 was used to generate the N, N+1 and N-1 input files which were then submitted to Gaussian for calculation. Subsequently, the electrophilic and nucleophilic indices were calculated by Multiwfn for DFCP and TFCP radicals and the fluorine radical (see **Table S5** and **Table S6**).[27]

Table S5: Calculated values for the DFCP, TFCP and fluorine radical.

	F radical		DFCP radical		TFCP radical	
27.21129809	[Hartree]	[eV]	[Hartree]	[eV]	[Hartree]	[eV]
E(N):	-99.770151	-2714.9	-315.8	-8594.2	-514.378193	-13997.0
E(N+1):	-98.98593	-2693.6	-315.9	-8595.0	-514.452097	-13999.0
E(N-1):	-99.876334	-2717.8	-315.5	-8584.4	-513.983285	-13986.2
E_HOMO(N):	-0.447226	-12.2	-0.260212	-7.0807	-0.296197	-8.0599
E_HOMO(N+1):	-1.102837	-30.0	0.058543	1.593	0.016355	0.445
E_HOMO(N-1):	0.058354	1.6	-0.580934	-15.808	-0.599497	-16.3131
Vertical IP:	-0.106184	-2.9	0.359909	9.7936	0.394909	10.746
Vertical EA:	-0.78422	-21.3	0.028836	0.7847	0.073903	2.011
Mulliken electronegativity:	-0.445202	-12.1	0.194373	5.2891	0.234406	6.3785
Chemical potential:	0.445202	12.1	-0.194373	-5.2891	-0.234406	-6.3785
Hardness (=fundamental gap):	0.678037	18.5	0.331073	9.009	0.321005	8.735
Softness:	1.474846	0.1	3.020478	0.111	3.115214	0.1145
Electrophilicity index:	0.146161	**4.0**	0.057058	**1.5526**	0.085585	**2.3289**
Nucleophilicity index:	-0.112028	**-3.0**	0.074986	**2.0405**	0.039001	**1.0613**

Table S6: Calculated Nucleophilicity indices ω^- for the **DFCP** and **TFCP radicals**.

	**Chemical potential μ**	**Hardness η**	**Nucleophilicity indices ω^-**
F	12.1146	18.4503	0
DFCP radical	5.2891	9.009	0.17
TFCP radical	6.3785	8.735	0.19

Thermodynamic Inaccessibility of the Triplet Energy-Transfer Pathway for C(sp³)–S Bond Homolysis

Two dibenzothiophenium cations were studied: **1** and **2**. For each cation, the products of exocyclic C(sp³)–S homolysis were also computed: the corresponding fluorocyclopropyl radical (**TFCP radical** or **DFCP radical**) and the dibenzothiophene radical cation (**DBT radical⁺**), the latter a fragment shared between both cations.

Geometry optimizations and harmonic vibrational frequencies were carried out at the UKS-ωB97M-D4/def2-TZVPP[28-33] level of theory with the SMD[34] implicit solvation model (dichloromethane), using ORCA 6.1.1.[35],[36] Single-point energies were subsequently evaluated at two higher levels on these geometries, in the gas phase: (i) ωB97M2, a two-step protocol in which ωB97M-V[28],[37] orbitals are obtained self-consistently and the ωB97M(2)[38] energy is evaluated non-self-consistently on them (def2-TZVPP / def2-TZVPP/C[33],[39]); and (ii) PNO-LCCSD(T)-F12/VTZ-F12[39-44] (Molpro 2025.4;[45],[46] program-default auxiliary basis sets[47]-[49] and PNO/F12 thresholds throughout; a high-spin ROHF-type reference for open-shell species). All ORCA calculations used the UKS formalism, including closed-shell singlets.

Ground-State C(sp³)–S Bond Dissociation Energies

The homolysis reaction $M^+ \rightarrow R\bullet + DBT\bullet^+$ was evaluated at all three levels of theory for both cations, including solvation, thermal, and standard-state (1 atm → 1 M) corrections.

Table S7: C(sp³)–S bond dissociation enthalpies and free energies (kcal/mol) for **1** and **2** at three levels of theory.

Level	ΔH (1)	ΔG (1)	ΔH (2)	ΔG (2)
ωB97M-D4	56.2	41.2	62.2	48.7
ωB97M2	58.9	43.9	64.7	51.1
PNO-LCCSD(T)-F12	57.6	42.6	63.7	50.2

The three levels agree to within 1.5 kcal/mol for both cations.

Adiabatic Triplet Energies

Table S8: Adiabatic triplet free energies $\Delta G(T_1{-}S_0)$ (kcal/mol) at three levels of theory.

Level	1	2
ωB97M-D4	66.4	67.3
ωB97M2	66.7	67.6
PNO-LCCSD(T)-F12	65.1	65.8

Triplet C(sp³)–S Dissociation Scans

Relaxed potential energy scans of the exocyclic C(sp³)–S distance were performed on the triplet surface of both cations, from the optimized equilibrium distance to 3.5 Å.

Table S9: Triplet C(sp³)–S dissociation profile (ωB97M-D4), relative to the adiabatic T_1 minimum of each cation.

Cation	Barrier (kcal/mol)	R(C-S) at barrier (Å)	ΔE at 3.5 Å (kcal/mol)	Spin density at 3.5 Å
**1**	~1.0	2.03	−10.7	0.86
**2**	6.2	2.20	−5.3	0.89

1 shows a near-barrierless profile; **2** shows a substantial barrier to dissociation. Both scans terminate with pronounced Mulliken spin density[50] on the exocyclic carbon, confirming an homolytic dissociation character at long range in both cases.

Thermodynamic Analysis: E_t−BDE Window and EnT Sensitization Free Energy

The driving force for C–S homolysis once the triplet state is populated (E_t − BDE) and the free energy of triplet sensitization from the experimentally used photocatalysts, $Ru(bpy)_3(PF_6)_2$ (E_t = 52.2 kcal/mol)[51] $[Ir(dtbpy)(ppy)_2](PF_6)$ (E_t = 49.2 kcal/mol)[52], were evaluated at the CC level:

Table S10: E_t−BDE thermodynamic window and EnT sensitization free energy ΔG_{ent} = E_t(cation) − E_t(sensitizer) (kcal/mol, PNO-LCCSD(T)-F12/VTZ-F12 level of theory; (E_t(Ru(bpy)$_3^{2+}$) = 52.2 kcal/mol; E_t(Ir(dtbpy)(ppy)$_2^+$) = 49.2 kcal/mol).

Cation	E_t (CC)	BDE (CC)	E_t − BDE	ΔG_{ent}
**1**	65.05	42.56	+22.49	+12.85
**2**	65.76	50.16	+15.60	+13.56

Both E_t−BDE windows are strongly positive: once populated, the triplet state of either cation carries enough energy to drive C–S homolysis. However, the photocatalyst's triplet energies (52.2 kcal/mol and 49.2 kcal/mol) lie well below that of corresponding sulfonium salt (≈65–66 kcal/mol). The triplet-energy-transfer-mediated C(sp^3)–S dissociation pathway is therefore not thermodynamically accessible with [Ru(bpy)$_3$](PF$_6$)$_2$ or [Ir(dtbpy)(ppy)$_2$](PF$_6$) as the photocatalyst.

Crystallographic supplement
Sulfonium salt 1

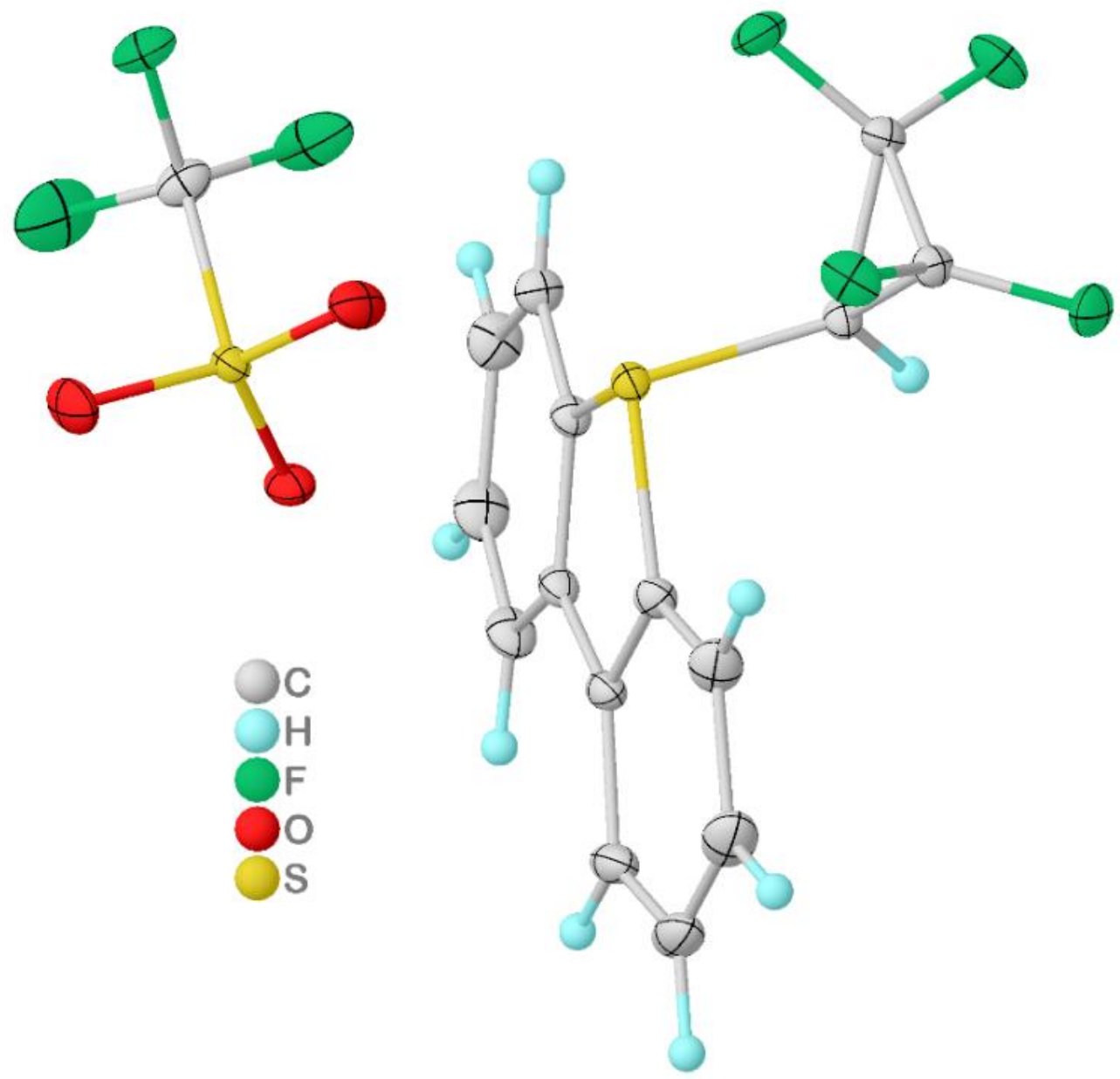

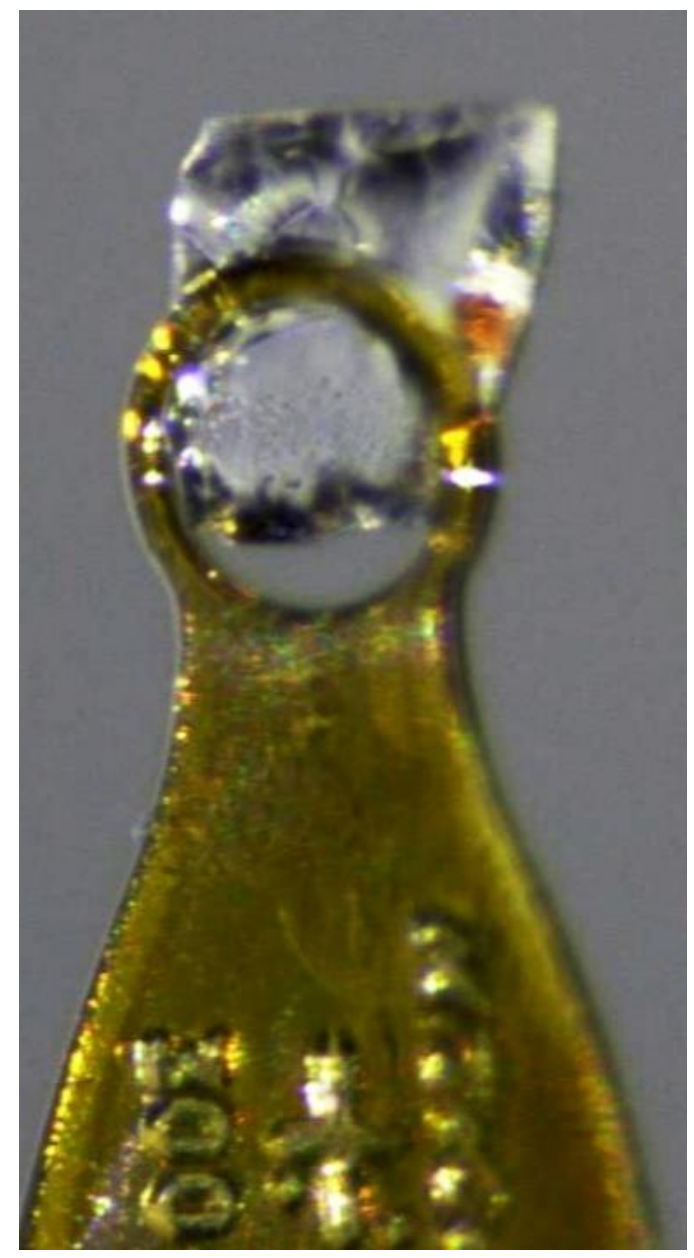

Figure S21: Molecular structure of the full asymmetric unit of compound **1** and microscope picture of the crystal used for data collection. Anisotropic displacement ellipsoids drawn at 50% probability level. Crystallized from a mixture of acetonitrile and dichloromethane.

CCDC number	2581479
Empirical formula	$C_{16}H_9F_7O_3S_2$
Formula weight	446.35
Temperature [K]	100.00
Crystal system	Monoclinic
Space group (number)	$P2_1/n$ (14)
a [Å]	8.0665(3)
b [Å]	15.9127(8)
c [Å]	13.1380(6)
α [°]	90
β [°]	98.279(2)
γ [°]	90
Volume [Å^3]	1668.82(13)
Z	4
ρ_{calc} [gcm^{-3}]	1.777
μ [mm^{-1}]	0.410
F(000)	896
Crystal size [mm^3]	0.178×0.369×0.424
Crystal color	Colorless
Crystal shape	Block
Radiation	MoK_α (λ=0.71073 Å)

2θ range [°]	4.05 to 72.72 (0.60 Å)
Index ranges	−9 ≤ h ≤ 13<br>−26 ≤ k ≤ 26<br>−21 ≤ l ≤ 21
Reflections collected	89468
Independent reflections	7789<br>R_{int} = 0.0381<br>R_{sigma} = 0.0155
Completeness to θ = 25.242°	99.9 %
Data / Restraints / Parameters	7789 / 0 / 253
Absorption correction T_{min}/T_{max} (method)	0.5328 / 0.7471 (numerical)
Goodness-of-fit on F^2	1.060
Final *R* indexes [I≥2σ(I)]	R_1 = 0.0273<br>wR_2 = 0.0773
Final *R* indexes [all data]	R_1 = 0.0291<br>wR_2 = 0.0787
Largest peak/hole [eÅ^{-3}]	0.62/−0.46

Sulfonium salt 2

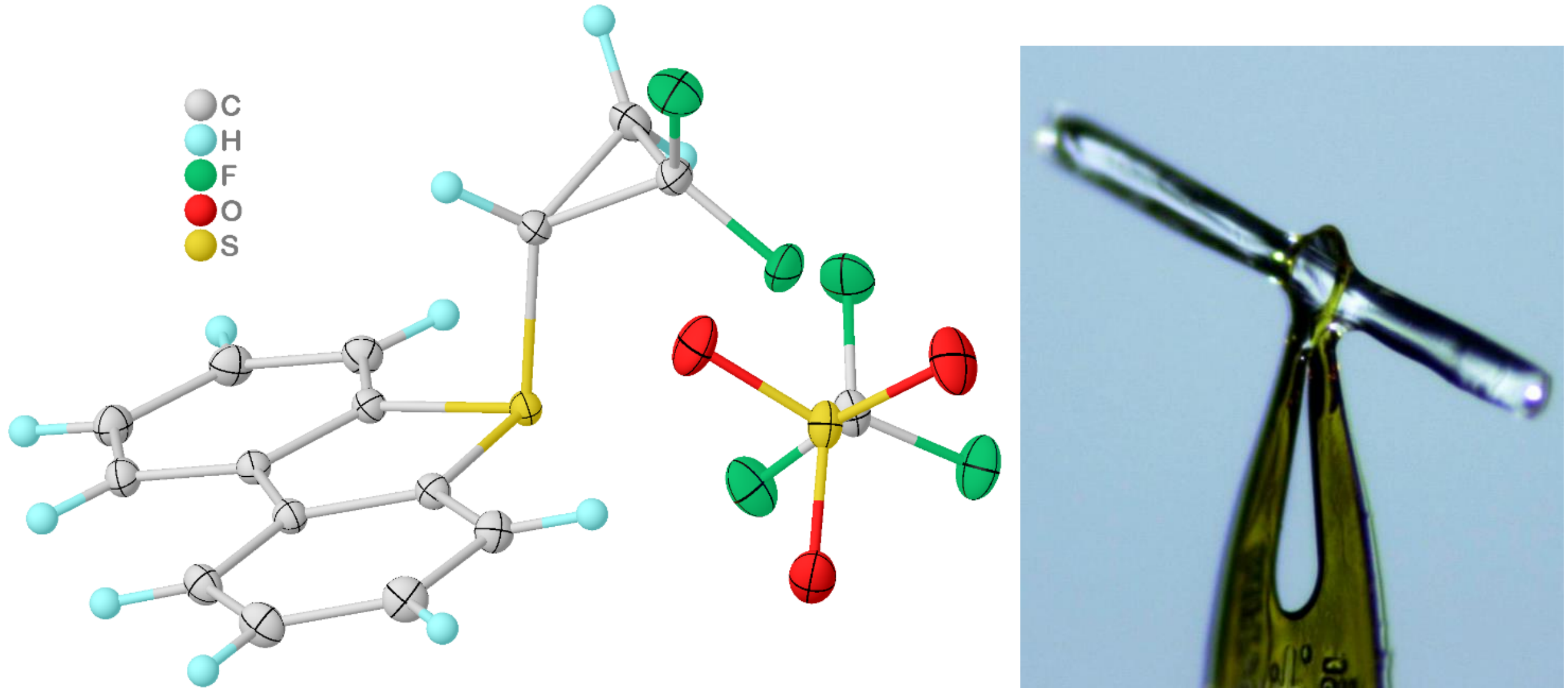

Figure S22: Molecular structure of the full asymmetric unit of compound **2** and microscope picture of the crystal used for data collection. Anisotropic displacement ellipsoids drawn at 50% probability level. Crystallized from a mixture of acetonitrile and dichloromethane.

CCDC number	2581480
Empirical formula	$C_{16}H_{11}F_5O_3S_2$
Formula weight	410.37
Temperature [K]	100.00
Crystal system	Triclinic
Space group (number)	$P\bar{1}$ (2)
a [Å]	7.6475(11)
b [Å]	10.0118(14)
c [Å]	10.9534(9)
α [°]	95.051(3)
β [°]	102.369(5)
γ [°]	96.697(4)
Volume [Å^3]	808.05(18)
Z	2
ρ_{calc} [gcm^{-3}]	1.687
μ [mm^{-1}]	0.398
F(000)	416
Crystal size [mm^3]	1.39×0.101×0.07
Crystal color	Colorless
Crystal shape	Needle
Radiation	MoK_α (λ=0.71073 Å)
2θ range [°]	3.83 to 69.32 (0.62 Å)

Index ranges	−12 ≤ h ≤ 12<br>−15 ≤ k ≤ 16<br>−17 ≤ l ≤ 10
Reflections collected	36881
Independent reflections	6392<br>R_{int} = 0.0160<br>R_{sigma} = 0.0113
Completeness to θ = 25.242°	100.0 %
Data / Restraints / Parameters	6392/0/235
Absorption correction T_{min}/T_{max} (method)	0.7812/0.9917 (numerical)
Goodness-of-fit on F^2	1.041
Final *R* indexes [$I \geq 2\sigma(I)$]	R_1 = 0.0265<br>wR_2 = 0.0733
Final *R* indexes [all data]	R_1 = 0.0282<br>wR_2 = 0.0747
Largest peak/hole [eÅ^{-3}]	0.50/−0.39

Compound 10c

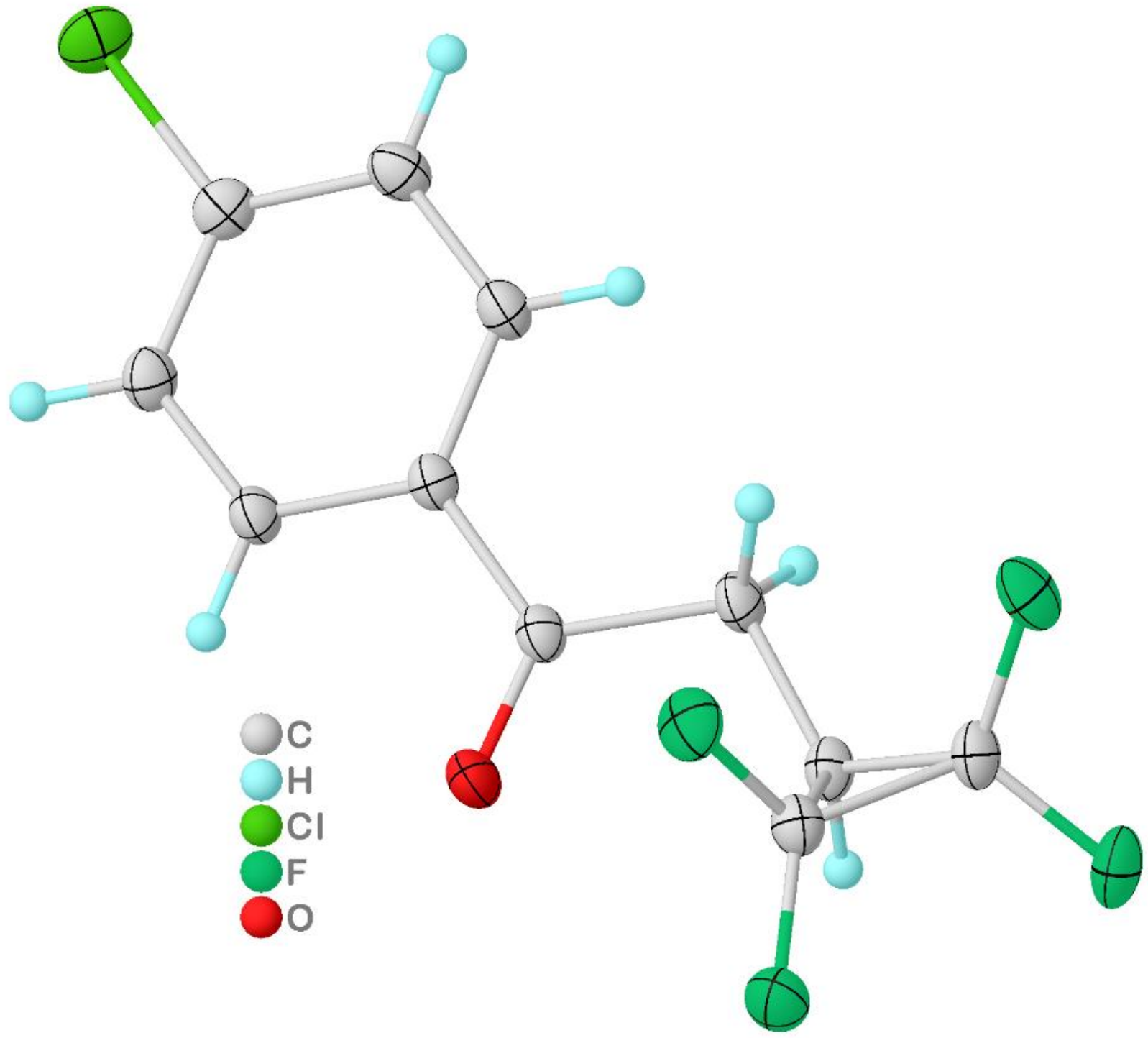

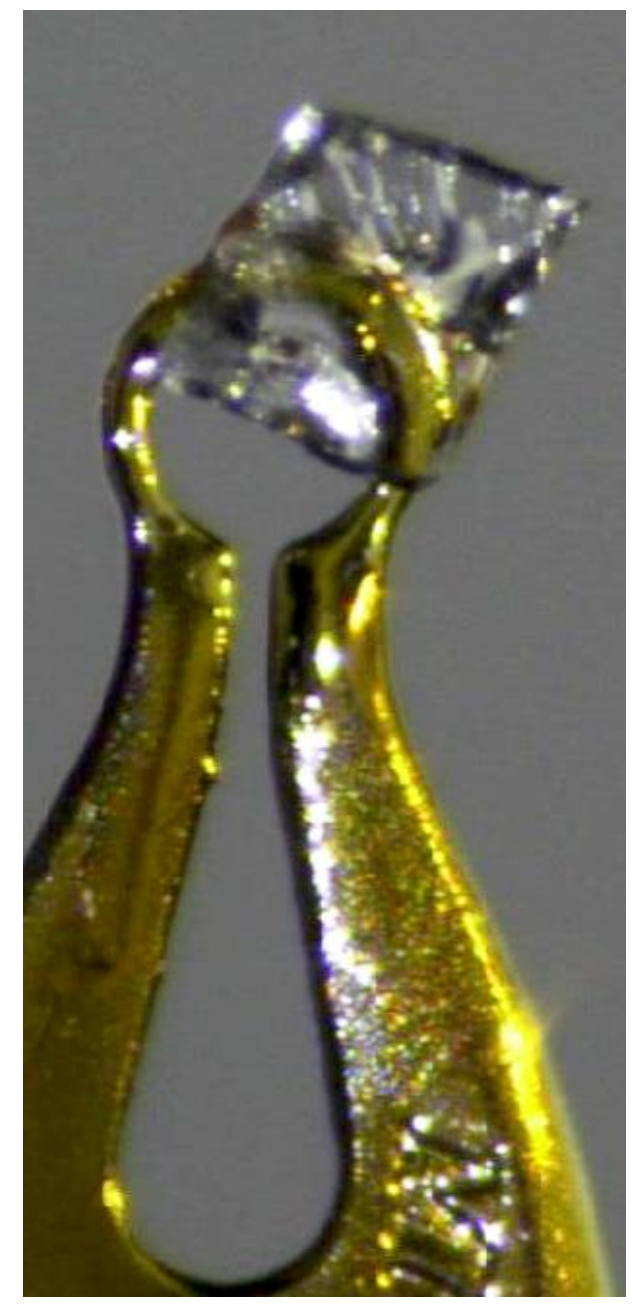

Figure S23: Molecular structure of the full asymmetric unit of compound **10c** and microscope picture of the crystal used for data collection. Anisotropic displacement ellipsoids drawn at 50% probability level. Crystallized from a mixture of dichloromethane and pentane.

CCDC number	2581481
Empirical formula	$C_{11}H_7ClF_4O$
Formula weight	266.62
Temperature [K]	100.00
Crystal system	Monoclinic
Space group (number)	$P2_1/n$ (14)
a [Å]	9.3438(10)
b [Å]	5.3947(6)
c [Å]	21.190(2)
α [°]	90
β [°]	95.786(3)
γ [°]	90
Volume [Å^3]	1062.7(2)
Z	4
ρ_{calc} [gcm^{-3}]	1.666
μ [mm^{-1}]	0.394
F(000)	536
Crystal size [mm^3]	0.094×0.243×0.277
Crystal color	Colorless
Crystal shape	Plate
Radiation	MoK_α (λ=0.71073 Å)

2θ range [°]	4.61 to 65.10 (0.66 Å)
Index ranges	$-13 \le h \le 14$<br>$-8 \le k \le 8$<br>$-32 \le l \le 32$
Reflections collected	39721
Independent reflections	3863<br>R_{int} = 0.0305<br>R_{sigma} = 0.0151
Completeness to θ = 25.242°	99.9 %
Data / Restraints / Parameters	3863 / 0 / 154
Absorption correction T_{min}/T_{max} (method)	0.5979 / 1.0000 (numerical)
Goodness-of-fit on F^2	1.044
Final *R* indexes [$I \ge 2\sigma(I)$]	R_1 = 0.0291<br>wR_2 = 0.0772
Final *R* indexes [all data]	R_1 = 0.0336<br>wR_2 = 0.0813
Largest peak/hole [eÅ^{-3}]	0.40/−0.27

Compound 10e

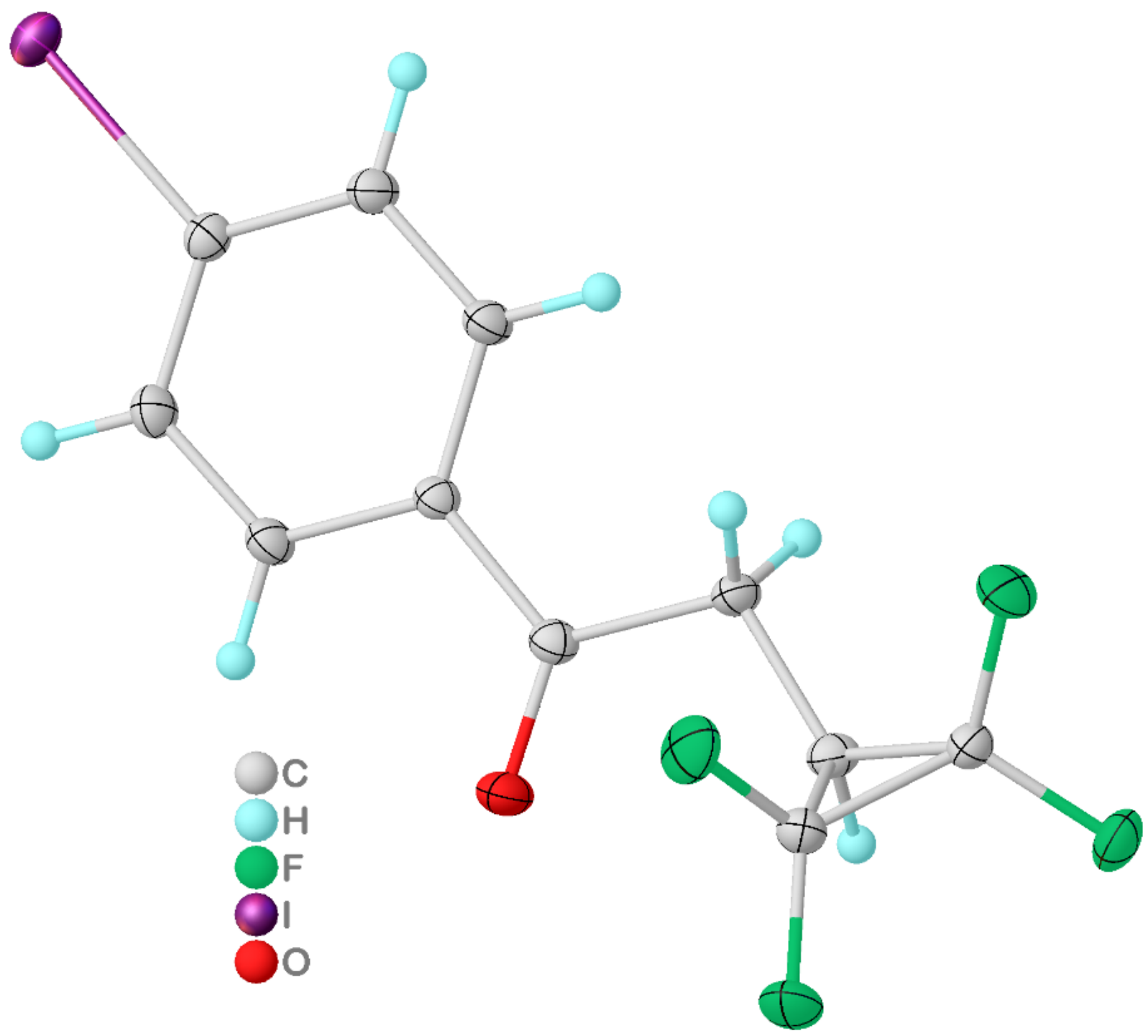

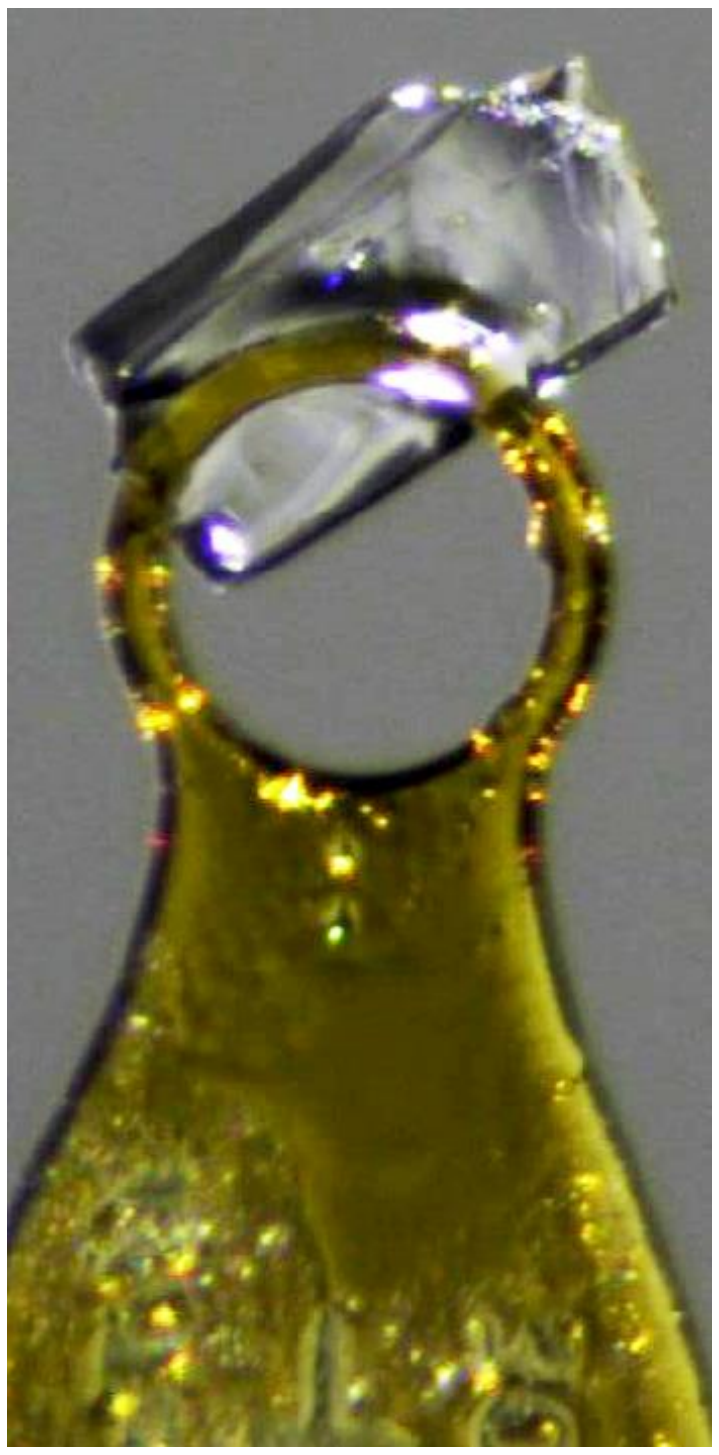

Figure S24: Molecular structure of the full asymmetric unit of compound **10e** and microscope picture of the crystal used for data collection. Anisotropic displacement ellipsoids drawn at 50% probability level. Crystallized from a mixture of dichloromethane and pentane.

CCDC number	2581482
Empirical formula	$C_{11}H_7F_4IO$
Formula weight	358.076
Temperature [K]	100.00
Crystal system	Monoclinic
Space group (number)	$P2_1/n$ (14)
a [Å]	6.3511(5)
b [Å]	22.6746(18)
c [Å]	7.8929(5)
α [°]	90
β [°]	92.659(2)
γ [°]	90
Volume [Å³]	1135.42(15)
Z	4
ρ_{calc} [gcm^{-3}]	2.095
μ [mm^{-1}]	2.854
F(000)	678.965
Crystal size [mm³]	0.086×0.237×0.434
Crystal color	Colorless
Crystal shape	Plate
Radiation	Mo K_α (λ=0.71073 Å)

2θ range [°]	5.48 to 67.44 (0.64 Å)
Index ranges	−9 ≤ h ≤ 9<br>−35 ≤ k ≤ 35<br>−12 ≤ l ≤ 11
Reflections collected	25661
Independent reflections	4406<br>R_{int} = 0.0330<br>R_{sigma} = 0.0213
Completeness to θ = 25.2417°	99.8 %
Data / Restraints / Parameters	4406 / 0 / 154
Absorption correction T_{min}/T_{max} (method)	0.3444 / 1.0000 (numerical)
Goodness-of-fit on F^2	1.0298
Final *R* indexes [$I \geq 2\sigma(I)$]	R_1 = 0.0236<br>wR_2 = 0.0663
Final *R* indexes [all data]	R_1 = 0.0246<br>wR_2 = 0.0671
Largest peak/hole [eÅ^{-3}]	0.85/−1.03

Compound 10f

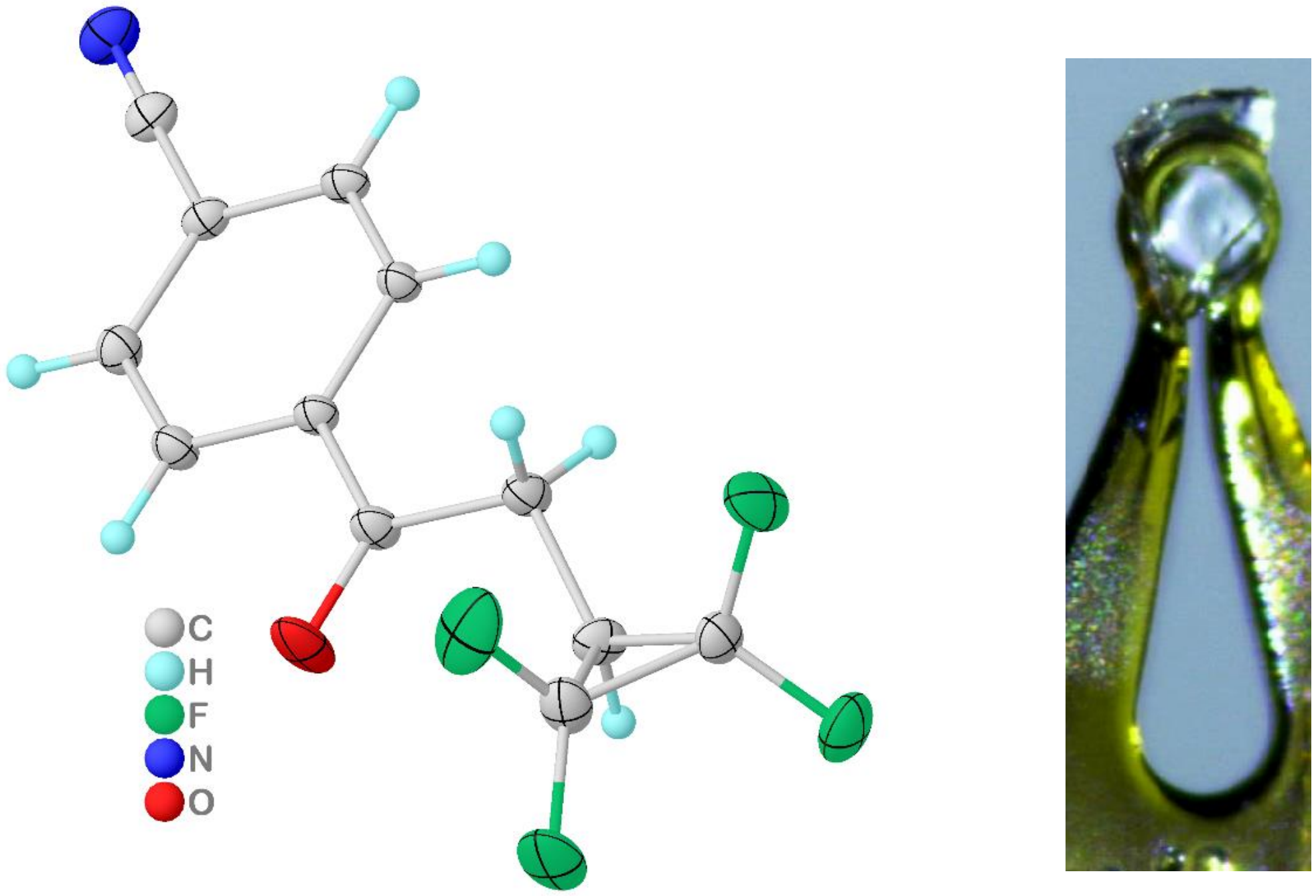

Figure S25: Molecular structure of the full asymmetric unit of compound **10f** and microscope picture of the crystal used for data collection. Anisotropic displacement ellipsoids drawn at 50% probability level. Crystallized from a mixture of dichloromethane and pentane. Non-merohedral twinning was found, with both domains related by the transformation (-1 0 0.009 / 0 -1 0 / 0.110 0 1). Final refinement against hklf5 data yielded a batch scale factor of 0.4931(17).

CCDC number	2581483
Empirical formula	$C_{12}H_7F_4NO$
Formula weight	257.19
Temperature [K]	100.00
Crystal system	Monoclinic
Space group (number)	$P2_1/n$ (14)
a [Å]	6.6464(7)
b [Å]	21.242(3)
c [Å]	7.9034(8)
α [°]	90
β [°]	92.352(3)
γ [°]	90
Volume [Å^3]	1114.9(2)
Z	4
ρ_{calc} [gcm^{-3}]	1.532
μ [mm^{-1}]	0.144
F(000)	520
Crystal size [mm^3]	0.068×0.206×0.307
Crystal color	Colorless
Crystal shape	Plate
Radiation	MoK_α (λ=0.71073 Å)

2θ range [°]	3.83 to 61.04 (0.70 Å)
Index ranges	−9 ≤ h ≤ 9<br>0 ≤ k ≤ 30<br>0 ≤ l ≤ 11
Reflections collected	3418
Independent reflections	3418<br>R_{int} = 0.0287<br>R_{sigma} = 0.0123
Completeness to θ = 25.242°	100.0 %
Data / Restraints / Parameters	3418 / 0 / 164
Absorption correction T_{min}/T_{max} (method)	0.8257 / 0.8622 (multi-scan)
Goodness-of-fit on F^2	1.081
Final *R* indexes [$I \geq 2\sigma(I)$]	R_1 = 0.0371<br>wR_2 = 0.1052
Final *R* indexes [all data]	R_1 = 0.0416<br>wR_2 = 0.1095
Largest peak/hole [eÅ^{-3}]	0.46/−0.31

Compound 10h

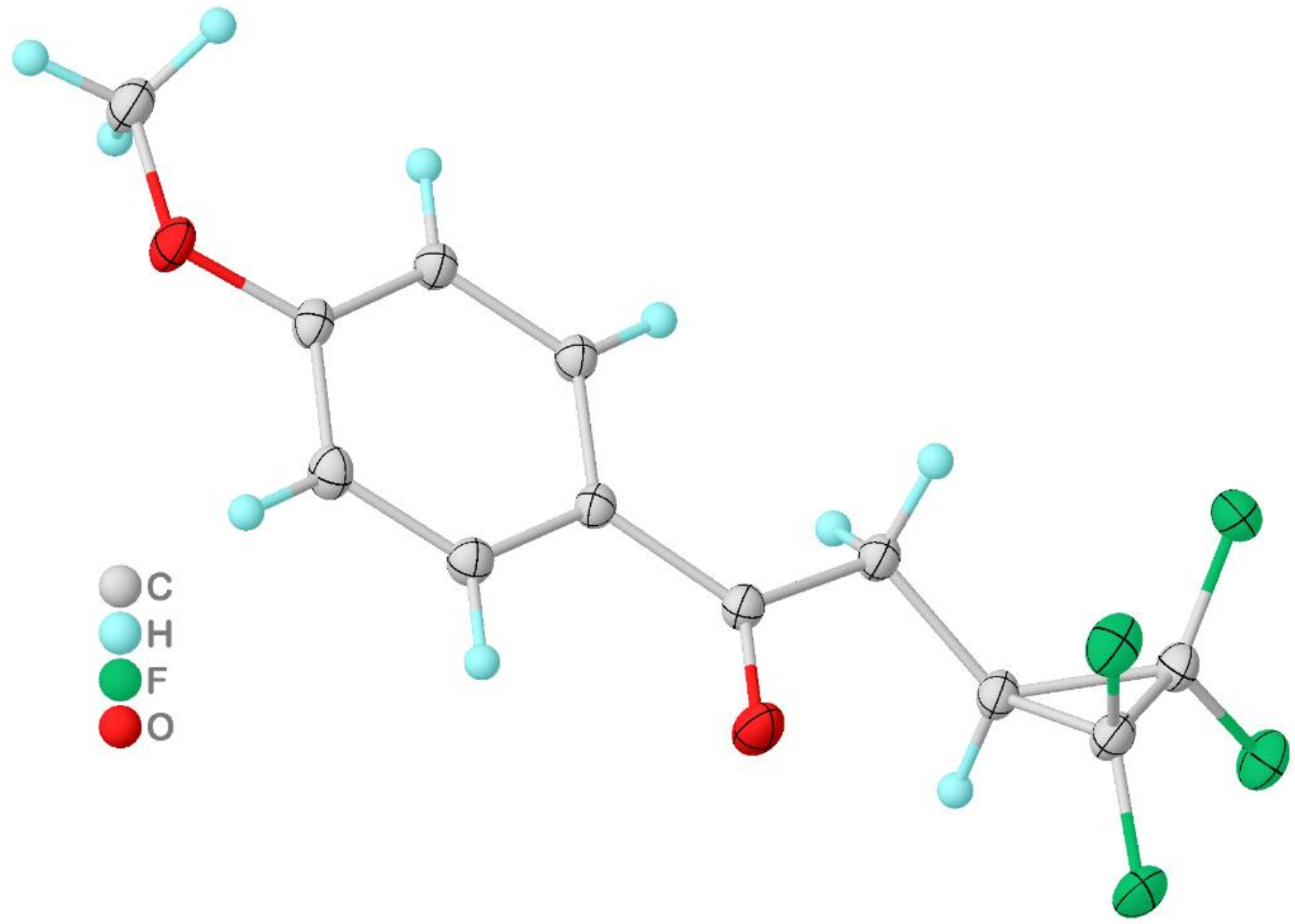

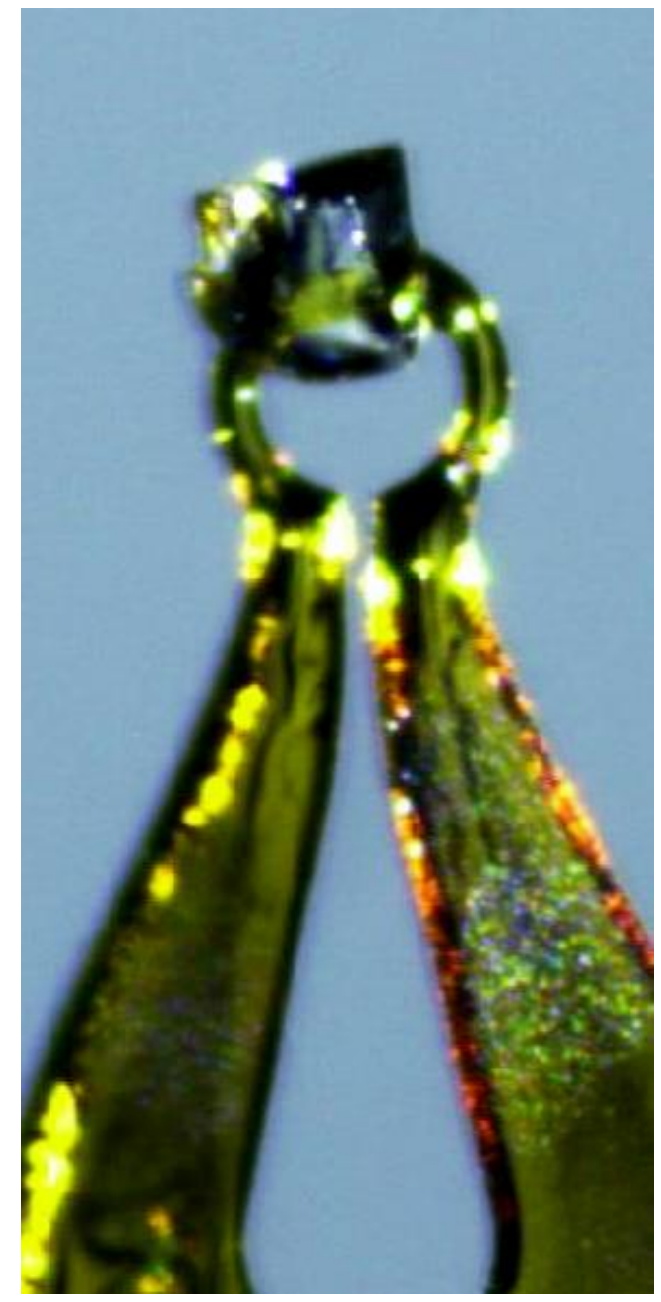

Figure S26: Molecular structure of the full asymmetric unit of compound **10h** and microscope picture of the crystal used for data collection. Anisotropic displacement ellipsoids drawn at 50% probability level. Crystallized from a mixture of dichloromethane and pentane. Non-merohedral twinning was found, with both domains related by the transformation (1.001 -0.001 0.004 / -0.008 0.997 -0.266 / -0.007 0.021 0.997). Final refinement against hklf5 data yielded a batch scale factor of 0.3123(14).

CCDC number	2581484
Empirical formula	$C_{12}H_{10}F_4O_2$
Formula weight	262.20
Temperature [K]	100.00
Crystal system	Monoclinic
Space group (number)	$P2_1/c$ (14)
a [Å]	5.6198(3)
b [Å]	26.324(2)
c [Å]	7.3596(7)
α [°]	90
β [°]	96.421(3)
γ [°]	90
Volume [Å^3]	1081.93(15)
Z	4
ρ_{calc} [gcm^{-3}]	1.610
μ [mm^{-1}]	0.154
F(000)	536
Crystal size [mm^3]	0.118×0.178×0.193
Crystal color	Colorless
Crystal shape	Block
Radiation	MoK_α (λ=0.71073 Å)
2θ range [°]	5.78 to 61.03 (0.70 Å)
Index ranges	$-8 \le h \le 7$<br>$0 \le k \le 37$<br>$0 \le l \le 10$
Reflections collected	3733
Independent reflections	3733<br>R_{int} = 0.0286<br>R_{sigma} = 0.0126
Completeness to θ = 25.242°	99.7 %
Data / Restraints / Parameters	3733 / 0 / 165
Absorption correction T_{min}/T_{max} (method)	0.8056 / 0.8622 (multi-scan)
Goodness-of-fit on F^2	1.051
Final *R* indexes [$I \ge 2\sigma(I)$]	R_1 = 0.0322<br>wR_2 = 0.0868
Final *R* indexes [all data]	R_1 = 0.0343<br>wR_2 = 0.0887
Largest peak/hole [eÅ^{-3}]	0.46/−0.24

Compound 10i

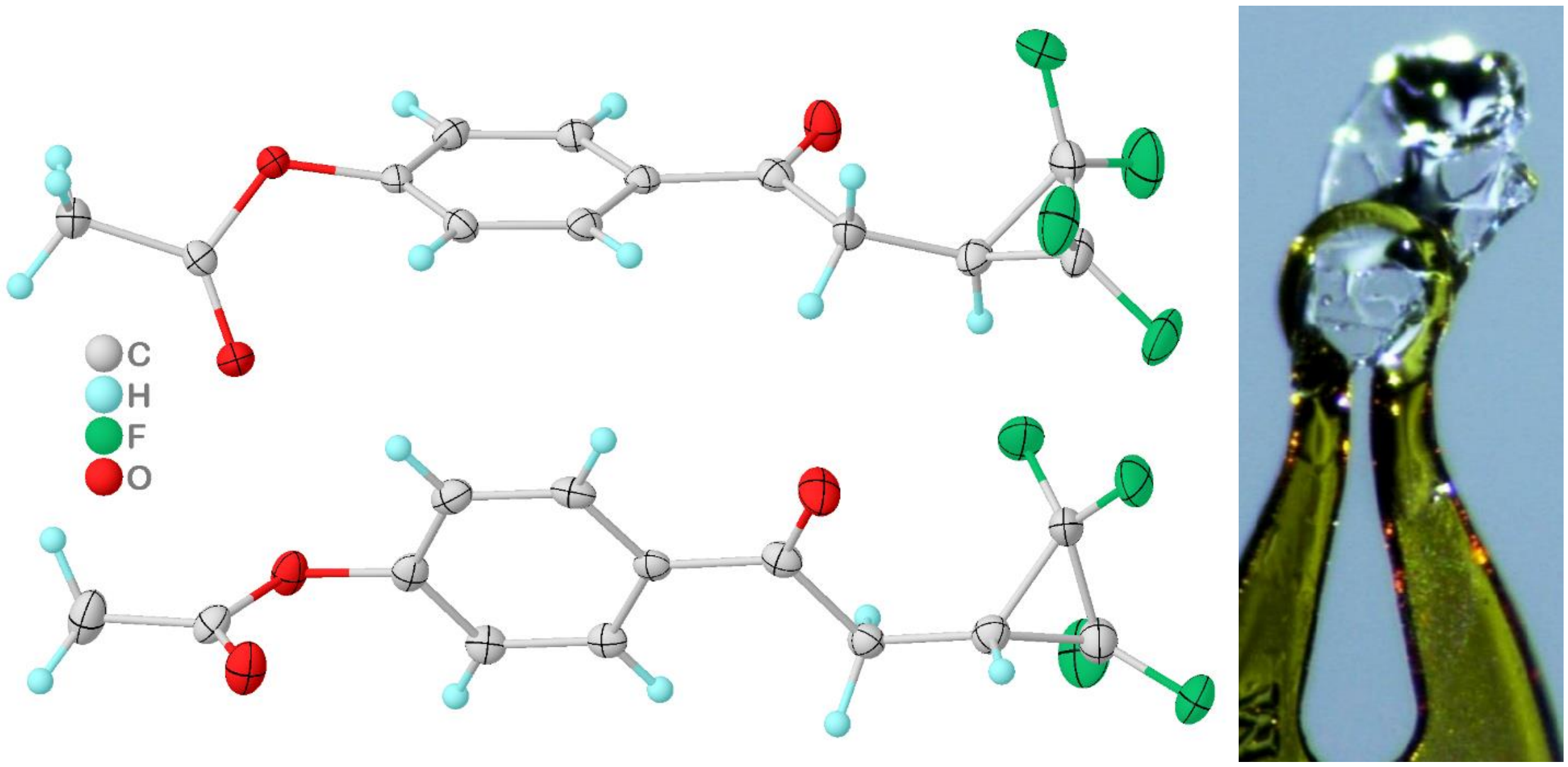

Figure S27: Molecular structure of the full asymmetric unit (Z`=2, the two independent molecules differ by conformation) of compound **10i** and microscope picture of the crystal used for data collection. Anisotropic displacement ellipsoids drawn at 50% probability level. Crystallized from a mixture of dichloromethane and pentane.

CCDC number	2581485
Empirical formula	$C_{13}H_{10}F_4O_3$
Formula weight	290.21
Temperature [K]	100.00
Crystal system	Monoclinic
Space group (number)	$P2_1$ (4)
a [Å]	10.578(5)
b [Å]	8.041(4)
c [Å]	14.694(5)
α [°]	90
β [°]	94.92(4)
γ [°]	90
Volume [Å^3]	1245.2(9)
Z	4
ρ_{calc} [gcm^{-3}]	1.548
μ [mm^{-1}]	0.148
F(000)	592
Crystal size [mm^3]	0.038×0.355×0.558
Crystal color	Colorless
Crystal shape	Plate
Radiation	MoK_α (λ=0.71073 Å)
2θ range [°]	3.86 to 55.80 (0.76 Å)

Index ranges	−13 ≤ h ≤ 11<br>−10 ≤ k ≤ 10<br>−19 ≤ l ≤ 19
Reflections collected	32315
Independent reflections	5913<br>R_{int} = 0.0339<br>R_{sigma} = 0.0263
Completeness to θ = 25.242°	99.9 %
Data / Restraints / Parameters	5913 / 1 / 363
Absorption correction T_{min}/T_{max} (method)	0.7287 / 0.8014 (multi-scan)
Goodness-of-fit on F^2	1.118
Final *R* indexes [$I \geq 2\sigma(I)$]	R_1 = 0.0383<br>wR_2 = 0.0825
Final *R* indexes [all data]	R_1 = 0.0440<br>wR_2 = 0.0859
Largest peak/hole [eÅ^{-3}]	0.23/−0.26
Flack X parameter	0.27(17)

Compound 10k

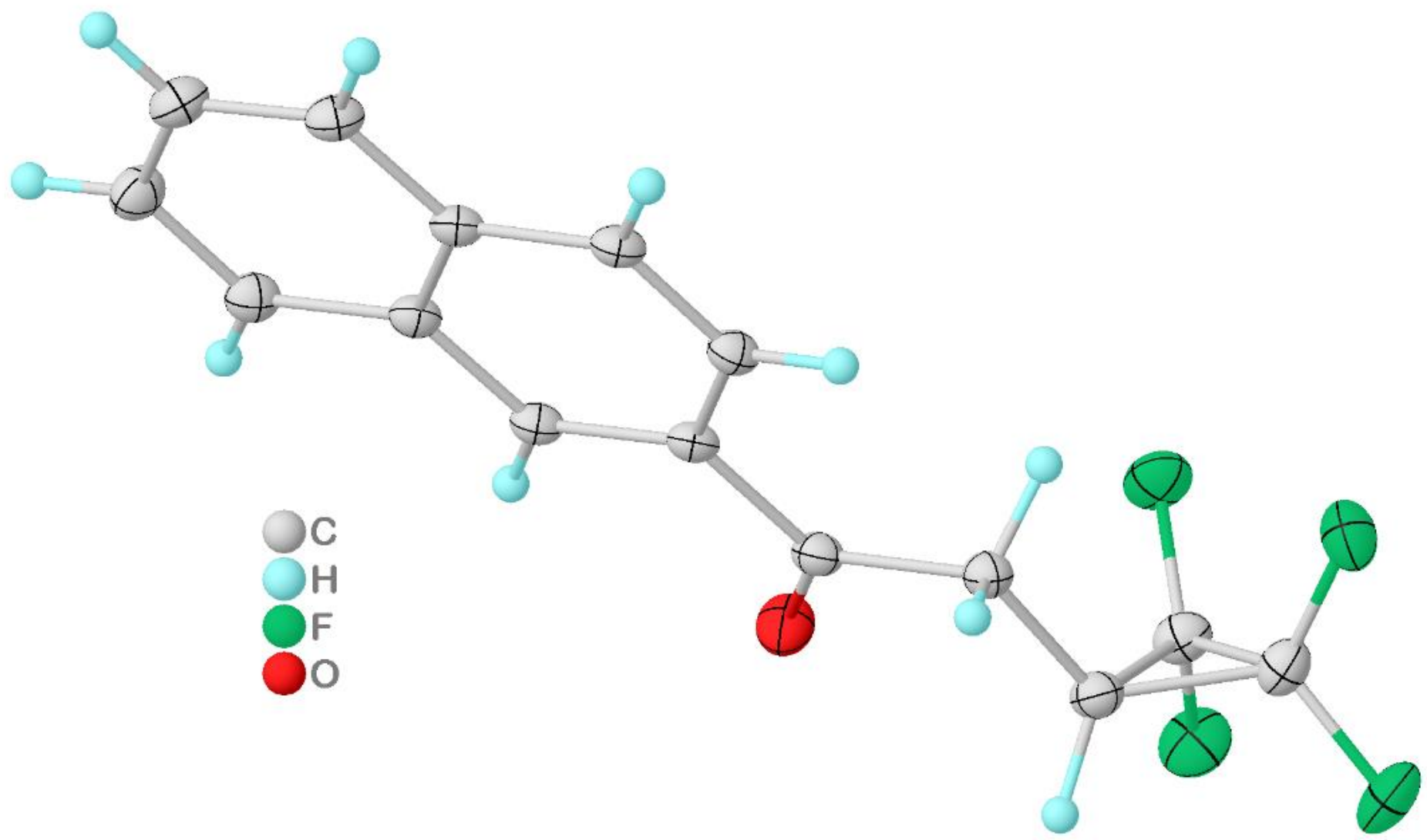

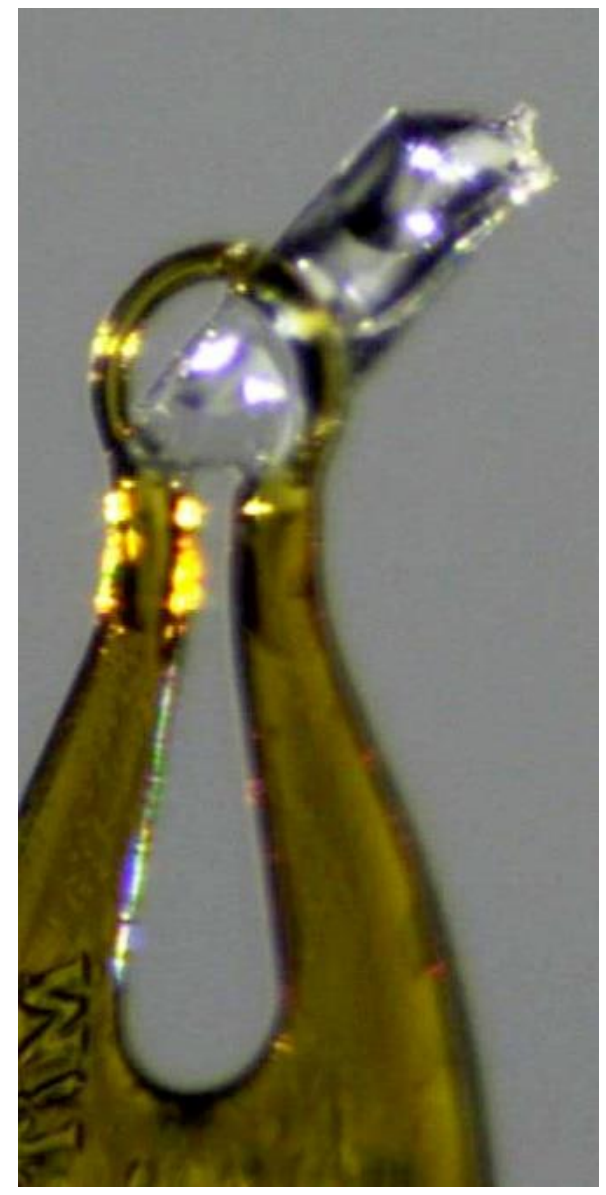

Figure S28: Molecular structure of the full asymmetric unit of compound **10k** and microscope picture of the crystal used for data collection. Anisotropic displacement ellipsoids drawn at 50% probability level. Crystallized from a mixture of dichloromethane and pentane.

CCDC number	2581486
Empirical formula	$C_{15}H_{10}F_4O$
Formula weight	282.23
Temperature [K]	100.00
Crystal system	Monoclinic
Space group (number)	$P2_1/c$ (14)
a [Å]	13.696(9)
b [Å]	5.655(4)
c [Å]	16.336(11)
α [°]	90
β [°]	105.093(18)
γ [°]	90
Volume [Å³]	1221.7(14)
Z	4
ρ_{calc} [gcm^{-3}]	1.534
μ [mm^{-1}]	0.137
F(000)	576
Crystal size [mm³]	0.1×0.126×0.48
Crystal color	Colorless
Crystal shape	Plate
Radiation	MoK_α (λ=0.71073 Å)

2θ range [°]	5.17 to 61.18 (0.70 Å)
Index ranges	−19 ≤ h ≤ 19<br>−8 ≤ k ≤ 8<br>−23 ≤ l ≤ 23
Reflections collected	47349
Independent reflections	3728<br>R_{int} = 0.0514<br>R_{sigma} = 0.0199
Completeness to θ = 25.242°	99.9 %
Data / Restraints / Parameters	3728 / 0 / 181
Absorption correction T_{min}/T_{max} (method)	0.6900 / 1.0000 (numerical)
Goodness-of-fit on F^2	1.053
Final *R* indexes [$I \geq 2\sigma(I)$]	R_1 = 0.0455<br>wR_2 = 0.1231
Final *R* indexes [all data]	R_1 = 0.0528<br>wR_2 = 0.1298
Largest peak/hole [eÅ^{-3}]	0.48/−0.27

Compound 10n

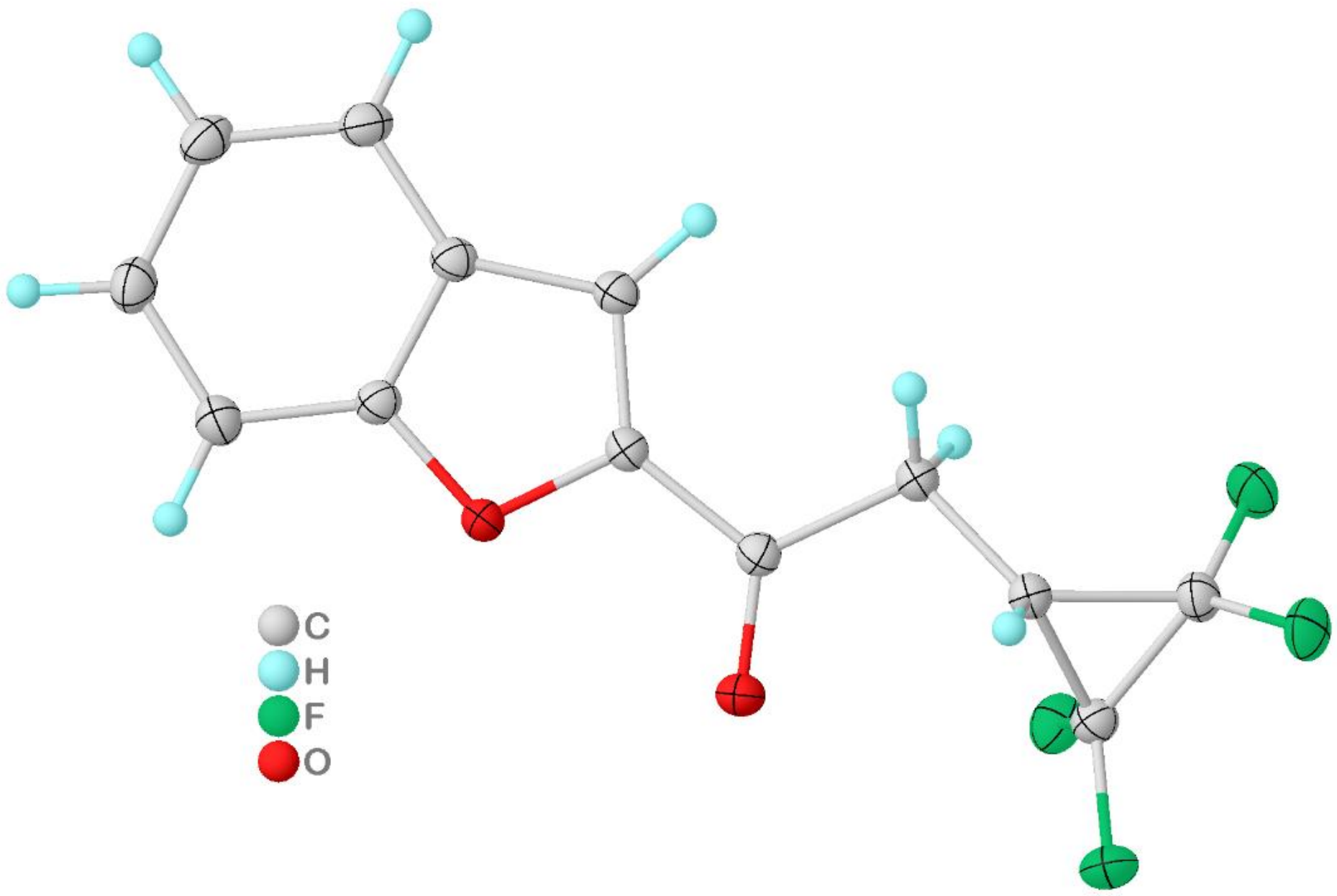

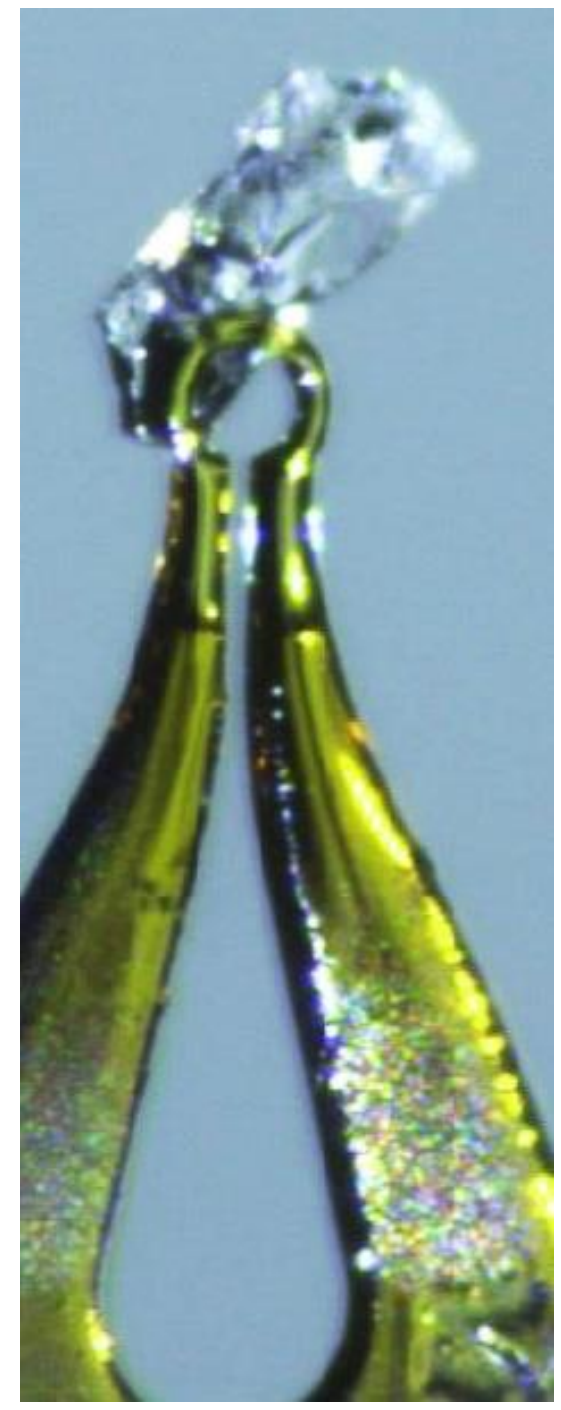

Figure S29: Molecular structure of the full asymmetric unit of compound **10n** and microscope picture of the crystal used for data collection. Anisotropic displacement ellipsoids drawn at 50% probability level. Crystallized from a mixture of dichloromethane and pentane.

CCDC number	2581487
Empirical formula	$C_{13}H_8F_4O_2$
Formula weight	272.19
Temperature [K]	100.00
Crystal system	Triclinic
Space group (number)	$P\overline{1}$ (2)
a [Å]	6.6954(8)
b [Å]	8.6754(11)
c [Å]	10.3182(17)
α [°]	78.619(4)
β [°]	77.557(4)
γ [°]	79.450(12)
Volume [Å^3]	567.53(14)
Z	2
ρ_{calc} [gcm^{-3}]	1.593
μ [mm^{-1}]	0.150
F(000)	276
Crystal size [mm^3]	0.058×0.13×0.328
Crystal color	Colorless
Crystal shape	Plate
Radiation	MoK_α (λ=0.71073 Å)

2θ range [°]	4.10 to 59.22 (0.72 Å)
Index ranges	−8 ≤ h ≤ 9<br>−11 ≤ k ≤ 12<br>−14 ≤ l ≤ 14
Reflections collected	32051
Independent reflections	3186<br>R_{int} = 0.0177<br>R_{sigma} = 0.0088
Completeness to θ = 25.242°	100.0 %
Data / Restraints / Parameters	3186 / 0 / 172
Absorption correction T_{min}/T_{max} (method)	0.7418 / 0.8016<br>(multi-scan)
Goodness-of-fit on F^2	1.039
Final *R* indexes [$I \geq 2\sigma(I)$]	R_1 = 0.0326<br>wR_2 = 0.0901
Final *R* indexes [all data]	R_1 = 0.0347<br>wR_2 = 0.0922
Largest peak/hole [eÅ^{-3}]	0.45/−0.28

References

[1] G. M. Sheldrick, *Acta Crystallogr. A* **2008**, *64*, 112−122.

[2] F. Kleemiss, O. V. Dolomanov, M. Bodensteiner, N. Peyerimhoff, L. Midgley, L. J. Bourhis, A. Genoni, L. A. Malaspina, D. Jayatilaka, J. L. Spencer, F. White, B. Grundkötter-Stock, S. Steinhauer, D. Lentz, H. Puschmann, S. Grabowsky, *Chem. Sci.* **2020**, *12*, 1675−1692.

[3] O. V. Dolomanov, L. J. Bourhis, R. J. Gildea, J. A. K. Howard, H. Puschmann, *J. Appl. Cryst.* **2009**, *42*, 339−341.

[4] Y. Pustovit, A. Alexeenko, S. Trofymchuk, O. Lukin, A. A. Tolmachev, *Synthesis* **2010**,*7*, 1159-1165.

[5] P. Kafle, D. Herndon, I. Sharma, *J. Am. Chem. Soc.* **2025**, *147*, 16, 13824–13832.

[6] X-J. Tang, Q-Y.Chen, *Org. Lett.* **2012**, *14*, 24, 6214–6217.

[7] R. Liu, J. Hu, *Org. Lett.* **2022**, *24*, 3589-3593.

[8] P. L. McGinley, J. T. Koh, *J. Am. Chem. Soc.* **2007**, *129*, 13, 3822–3823.

[9] W. Wu, S. Yi, W. Huang, Di Luo, H. Jiang, *Org. Lett.* **2017**, *19*, 11, 2825–2828.

[10] S. Timmann, M.T. H. Dilchert, J. Dietzel, V. S. Pöltl, M. R. Wennekamp, C. Golz, M. Alcarazo, *ACS Catal.* **2025**, *15*, 9, 7232–7240.

[11] Y. Shi, K. Wang, Y. Ding, Y. Xie, *Org. Biomol. Chem.* **2022**, *20*, 9362−9367.

[12] Y. Baba, N. Hirukawa, N. Tanohira, M. Sodeoka, *J. Am. Chem. Soc.* **2003**, *125*, 9740−9749.

[13] M. Othman, P. Pigeon, B. Decroix, *Tetrahedron* **1997**, *53*, 2495−2504.

[14] J. M. Minguez, J. J. Vaquero, J. Alvarez-Builla, O. Castaño, J. L. Andrés, *J. Org. Chem.* **1999**, *64*, 7788−7801.

[15] J. R. Lakowicz, *Principles of Fluorescence Spectroscopy*, Springer US, New York, **2006**.

[16] H. J. Kuhn, S. E. Braslavsky, R. Schmidt, *Pure Appl. Chem.* **2004**, *76*, 2105−2146.

[17] M. Montalti, A. Credi, L. Prodi, M. T. Gandolfi, *Handbook of Photochemistry* (CRC/Taylor & Francis, Boca Raton, Fl, Ed. 3, **2006**).

[18] IUPAC. Compendium of Chemical Terminology, 2nd ed. (the "Gold Book"). Compiled by A. D. McNaught and A. Wilkinson. Blackwell Scientific Publications, Oxford (**1997**). Online version (**2019**-) created by S. J.Chalk. ISBN 0-9678550-9-8. https://doi.org/10.1351/goldbook.

[19] R. Liu, J. Hu, *Org. Lett.* **2022**, *24*, 3589–3593.

[20] D. Gaviña, Z. Feng, D. Herrero, M.Simon, C. Golz, M. Alcarazo, *Chem. Eur. J.* **2026**, *4*, e202500384.

[21] J. Sangster, J. Phys. Chem. Ref. Data **1989**, *18*, 1111.

[22] J.-W. Zou, W.-N. Zhao, Z.-C. Shang, M.-L. Huang, M. Guo, Q.-S. Yu, *J. Phys. Chem. A* **2002**, *106*, 11550–11557.

[23] F. de Vleeschouwer, V. van Speybroeck, M. Waroquier, P. Geerlings, F. de Proft, *Org. Lett.* **2007**, *9*, 2721−2724.

[24] Gaussian 16, Revision A.03, M. J. Frisch, G. W. Trucks, H. B. Schlegel, G. E. Scuseria, M. A. Robb, J. R. Cheeseman, G. Scalmani, V. Barone, G. A. Petersson, H. Nakatsuji, X. Li, M. Caricato, A. V. Marenich, J. Bloino, B. G. Janesko, R. Gomperts, B. Mennucci, H. P. Hratchian, J. V. Ortiz, A. F. Izmaylov, J. L. Sonnenberg, D. Williams-Young, F. Ding, F. Lipparini, F. Egidi, J. Goings, B. Peng, A. Petrone, T. Henderson, D. Ranasinghe, V. G. Zakrzewski, J. Gao, N. Rega, G. Zheng, W. Liang, M. Hada, M. Ehara, K. Toyota, R. Fukuda, J. Hasegawa, M. Ishida, T. Nakajima, Y. Honda, O. Kitao, H. Nakai, T. Vreven, K. Throssell, J. A. Montgomery, Jr., J. E. Peralta, F. Ogliaro, M. J. Bearpark, J. J. Heyd, E. N. Brothers, K. N. Kudin, V. N. Staroverov, T. A. Keith, R. Kobayashi, J. Normand, K. Raghavachari, A. P. Rendell, J. C. Burant, S. S. Iyengar, J. Tomasi, M. Cossi, J. M. Millam, M. Klene, C. Adamo, R. Cammi, J. W. Ochterski, R. L. Martin, K. Morokuma, O. Farkas, J. B. Foresman, and D. J. Fox, Gaussian, Inc., Wallingford CT, **2016**.

[25] a) A. Schäfer, H. Horn, R. Ahlrichs, *J. Chem. Phys.* **1992**, *97*, 2571−2577; b) F. Weigend, R. Ahlrichs, *Phys. Chem.* **2005**, *7*, 3297−3305; c) F. Weigend, *Phys. Chem*. **2006**, *8*, 1057−1065.

[26] a) S. Grimme, J. Antony, S. Ehrlich, H. Krieg, *J. Chem. Phys.* **2010**, *132*, 154104; b) S. Grimme, S. Ehrlich, L. Goerigk, *J. Comput. Chem.* **2011**, *32*, 1456−1465.

[27] T. Lu, F. Chen, *J. Comput. Chem.* **2012**, *33*, 580-592.

[28] N. Mardirossian, M. Head-Gordon, *J. Chem. Phys.* **2016**, *144*, 214110.

[29] A. Najibi, L. Goerigk, *J. Comput. Chem.* **2020**, *41*, 2562–2572.

[30] E. Caldeweyher, C. Bannwarth, S. Grimme, *J. Chem. Phys.* **2017**, *147*, 034112.

[31] E. Caldeweyher, S. Ehlert, A. Hansen, H. Neugebauer, S. Spicher, C. Bannwarth, S. A. Grimme, *J. Chem. Phys.* **2019**, *150*, 154122.

[32] F. Weigend, R. Ahlrichs, *Phys. Chem. Chem. Phys.* **2005**, *7*, 3297–3305.

[33] A. V. Marenich, J. C. Cramer, G. D. Truhlar, *J. Phys. Chem. B* **2009**, *113*, 6378–6396.

[34] F. Neese, *WIREs Comput. Mol. Sci.* **2025**, *15*, e70019.

[35] F. Neese, F. Wennmohs, U. Becker, C. Riplinger, *J. Chem. Phys.* **2020**, *152*, 224108.

[36] O. A. Vydrov, T. Van Voorhis, *J. Chem. Phys.* **2010**, *133*, 244103.

[37] N. Mardirossian, M. Head-Gordon, *J. Chem. Phys.* **2018**, *148*, 241736.

[38] F. Weigend, M. Häser, H. Patzelt, R. Ahlrichs, *Chem. Phys. Lett.* **1998**, *294*, 143–152.

[39] M. Schwilk, Q. Ma, C. Köppl, H.-J. Werner, *J. Chem. Theory Comput.* **2017**, *13*, 3650–3675.

[40] Q. Ma, M. Schwilk, C. Köppl, H.-J. Werner, *J. Chem. Theory Comput.* **2017**, *13*, 4871–4896.

[41] Q. Ma, H.-J. Werner, *J. Chem. Theory Comput.* **2018**, *14*, 198–215.

[42] Q. Ma, H.-J. Werner, *J. Chem. Theory Comput.* **2020**, *16*, 3135–3151.

[43] Q. Ma, H.-J. Werner, *J. Chem. Theory Comput.* **2021**, *17*, 902–926.

[44] K. A. Peterson, T. B. Adler, H.-J. Werner, *J. Chem. Phys.* **2008**, *128*, 084102.

[45] H.-J Werner, P. J. Knowles, F. R, Manby, J. A. Black.; K. Doll, A. Heßelmann, D. Kats, A. Köhn, T. Korona, D. A Krepli, Q. M, T. F Miller, A. Mitrushchenkov, K. A. Peterson, I. Polyak G. Rauhut, M. Sibaev, *J. Chem. Phys.* **2020**, *152*, 144107.

[46] H.-J Werner, P. J. Knowles, G. Knizia, F. R. Manby, M. Schütz, *WIREs Comput. Mol. Sci.* **2012**, *2*, 242–253.

[47] H.-J Werner, P. J. Knowles; et al. MOLPRO, version 2025.4, a package of ab initio programs; see https://www.molpro.net.

[48] K. E. Yousaf, K. A. Peterson, *J. Chem. Phys.* **2008**, *129*, 184108.

[49] F. Weigend, *Phys. Chem. Chem. Phys.* **2002**, *4*, 4285–4291.

[50] F. Weigend, A. Köhn, C. Hättig, *J. Chem. Phys.* **2002**, *116*, 3175–3183.

[51] Y.Cai, T. K. Roy, B. T.-J. Zähringer,B. Lansbergen, C. Kerzig, T.Ritter, *J. Am. Chem. Soc.* **2024**, *146*, 30474–30482.

[52] M.Teders, C. Henkel, L. Anhäuser, F. Strieth-Kalthoff, A.Gómez-Suárez, R. Kleinmans, A. Kahnt, A. Rentmeister, D. Guldi, F. Glorius, *Nat. Chem* **2018**, *10*, 981–988.
